# Supplementary material for: European farmhouse brewing yeasts form a distinct genetic group
Source: Appl Microbiol Biotechnol. 2024 Aug 2;108:430. doi: 10.1007/s00253-024-13267-3 (PMC11297104; doi:10.1007/s00253-024-13267-3)

Supplementary Material

Journal: Applied Microbiology and Biotechnology

European Farmhouse Brewing Yeasts Form a Distinct Genetic Group

Richard Preiss[1], Eugene Fletcher[1,a], Lars Marius Garshol[2], Barret Foster[3], Emine Ozsahin[3], Mark Lubberts[3], George van der Merwe[3], Kristoffer Krogerus[4*]

[1] Escarpment Laboratories, Guelph, ON, Canada

[2] Independent researcher, Rælingen, Norway

[3] Department of Molecular and Cellular Biology, University of Guelph, Guelph, ON, Canada

[4] VTT Technical Research Centre of Finland, Tekniikantie 21, 02150, Espoo, Finland

[a] Current affiliation: Carleton University, Canada

[*] Address correspondence to Kristoffer Krogerus, kristoffer.krogerus@vtt.fi

Supplementary Figure and Table legends

Supplementary Figure S1. Visualisation of phased haplotigs detected by nPhase in the 17 phased strains.

Supplementary Figure S2. Median coverage in 10 kbp windows across the genome of the 105 *S. cerevisiae* strains included in the genomic analysis.

Supplementary Figure S3. Allele frequency distributions across the genome of the 105 *S. cerevisiae* strains included in the genomic analysis.

Supplementary Figure S4. A neighbour-joining tree generated based on MinHash distances in the (**A**) assemblies of 1,011 yeast genomes (Peter et al. 2018), and (**B**) 142 assemblies of ScRAP (O'Donnell et al. 2023), together with long-read assemblies of 9 landrace and 6 control strains generated here. The branches containing the 'Mixed origin', 'European farmhouse' and 'Beer 1'/'Ale beer' populations are highlighted.

Supplementary Figure S5. (A) Maximum likelihood phylogenetic tree based on SNPs at 310688 sites in 1,011 yeast genome strains (Peter et al. 2018), the 35 landrace and five brewing control strains sequenced here, and the five sequenced NCYC 'kveik' strains. (**B**) A neighbour-joining tree based on SNPs at 1652813 biallelic sites in 1,011 yeast genome strains (Peter et al. 2018), the 35 landrace and five brewing control strains sequenced here, and the five sequenced NCYC 'kveik' strains. The branches containing the 'Mixed origin', 'European farmhouse' and 'Beer 1'/'Ale beer' populations are highlighted.

Supplementary Figure S6. Population structure of a merged set of 35 *S. cerevisiae* landrace brewing strains and the 1,011 yeast genomes strains estimated with ADMIXTURE based on SNPs at 31766 sites. Each strain along the x-axis is represented by a vertical bar partitioned into colors based on estimated membership fractions to the resolved populations for K = 8 to 20 assumed ancestral populations.

Supplementary Figure S7. Heatmaps visualising *D*, f_4-ratio and *f*-branch statistics between populations as calculated with DSuite. (**A**) The *f*-branch statistics between population tree tips and nodes. A warmer colour represents a higher estimated f-branch value, which indicates the proportion of the genome affected by admixture. Grey shading indicates tests that could not be made. The maximum (**B**) *D* and (**C**) f_4-ratio statistics between pairs of populations. A red color indicates a higher value, while a blue color a lower value.

Supplementary Figure S8. The network graph topology with highest posterior probability as derived by AdmixtureBayes from the merged data set of 1,011 yeast genome strains (Peter et al. 2018), the 35 landrace and five brewing control strains sequenced here, and the five sequenced NCYC 'kveik' strains. The data set converged to and was best described by 20 admixture events. Red squares indicate admixture events, while blue ovals indicate populations.

Supplementary Figure S9. Change in specific gravity over time in the wort fermentations with landrace and control brewing strains. Error bars show standard deviation of three replicates.

Supplementary Figure S10. Concentration of isoamyl, isobutyl and phenylethyl acetate in the beers fermented with landrace and control brewing strains. Error bars show standard deviation of three replicates. Different letters above bars indicate significant differences by one-way ANOVA ($p < 0.05$).

Supplementary Figure S11. Relative total ester formation by group among the landrace and control brewing strains during wort fermentations. Different letters above bars indicate significant differences by one-way ANOVA ($p < 0.05$).

Supplementary Figure S12. Average copy number of genes related to maltose and maltotriose transport and metabolism among the landrace strains sequenced here and the 1,011 yeast genome strains (Peter et al. 2018) grouped by population.

Supplementary Figure S13. Copy number of *RTM1* among the 1,011 yeast genomes strains (Peter et al. 2018) grouped by population.

Supplementary Figure S14. The *RTM1* cluster in the long-read assemblies of the landrace and control brewing strains. *RTM1* is highlighted in red, while *SUC2* is highlighted in blue.

Supplementary Table S1. A list of strains included in the genomic analysis, the number of homozygous and heterozygous SNPs detected, and their accession numbers.

Supplementary Table S2. Assembly statistics for the 15 long-read assemblies generated here.

Supplementary Figure S1

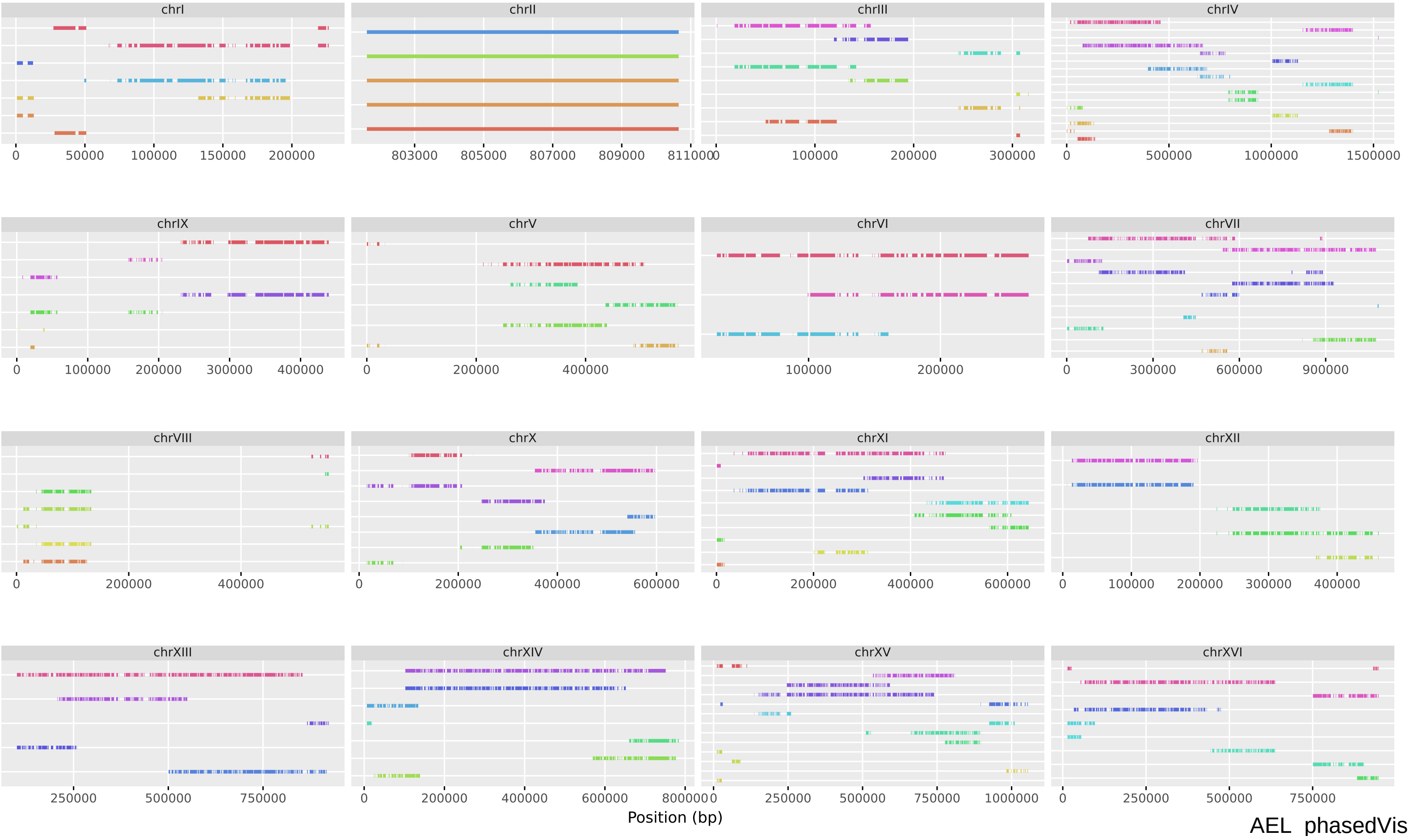

Supplementary Figure S1

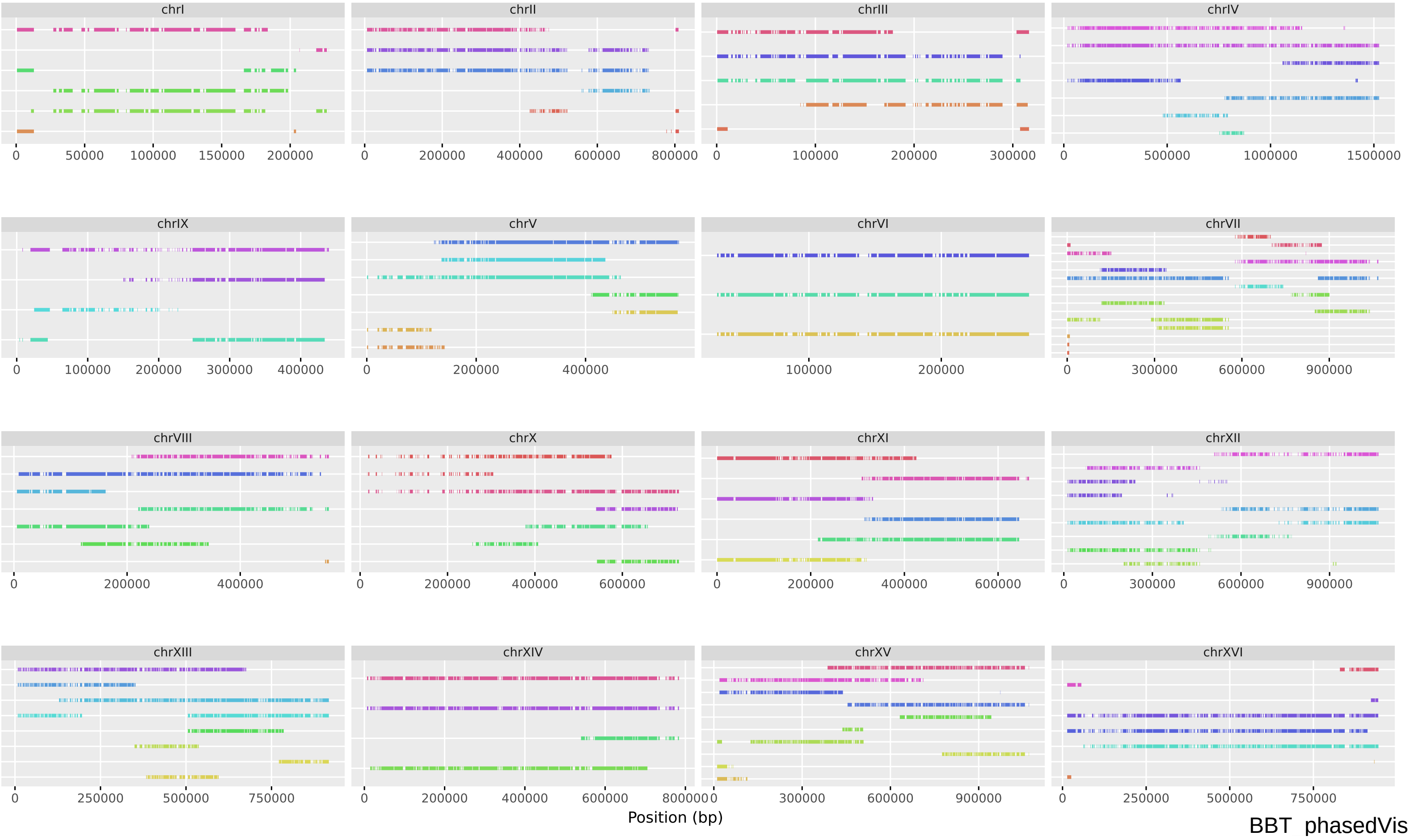

Supplementary Figure S1

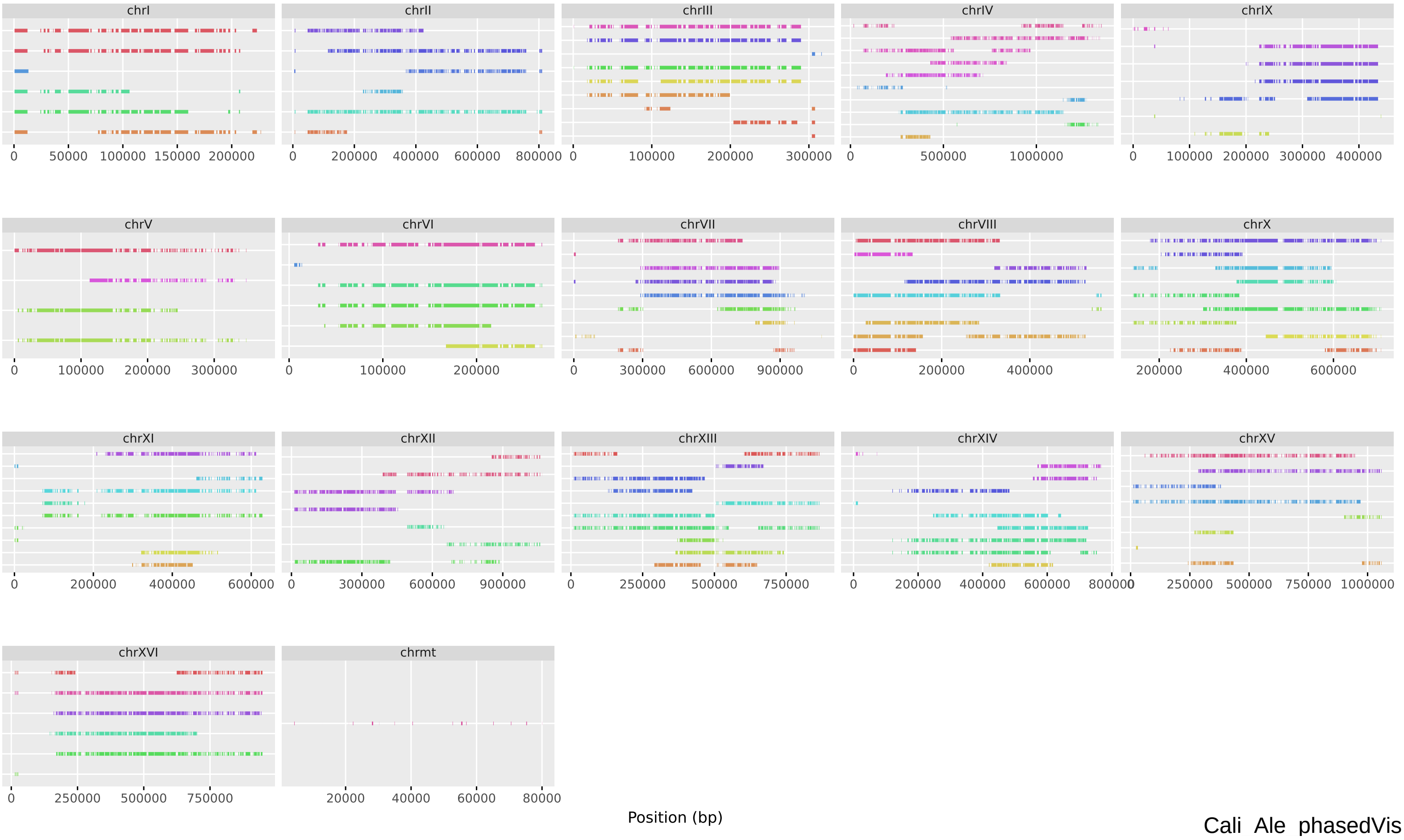

Supplementary Figure S1

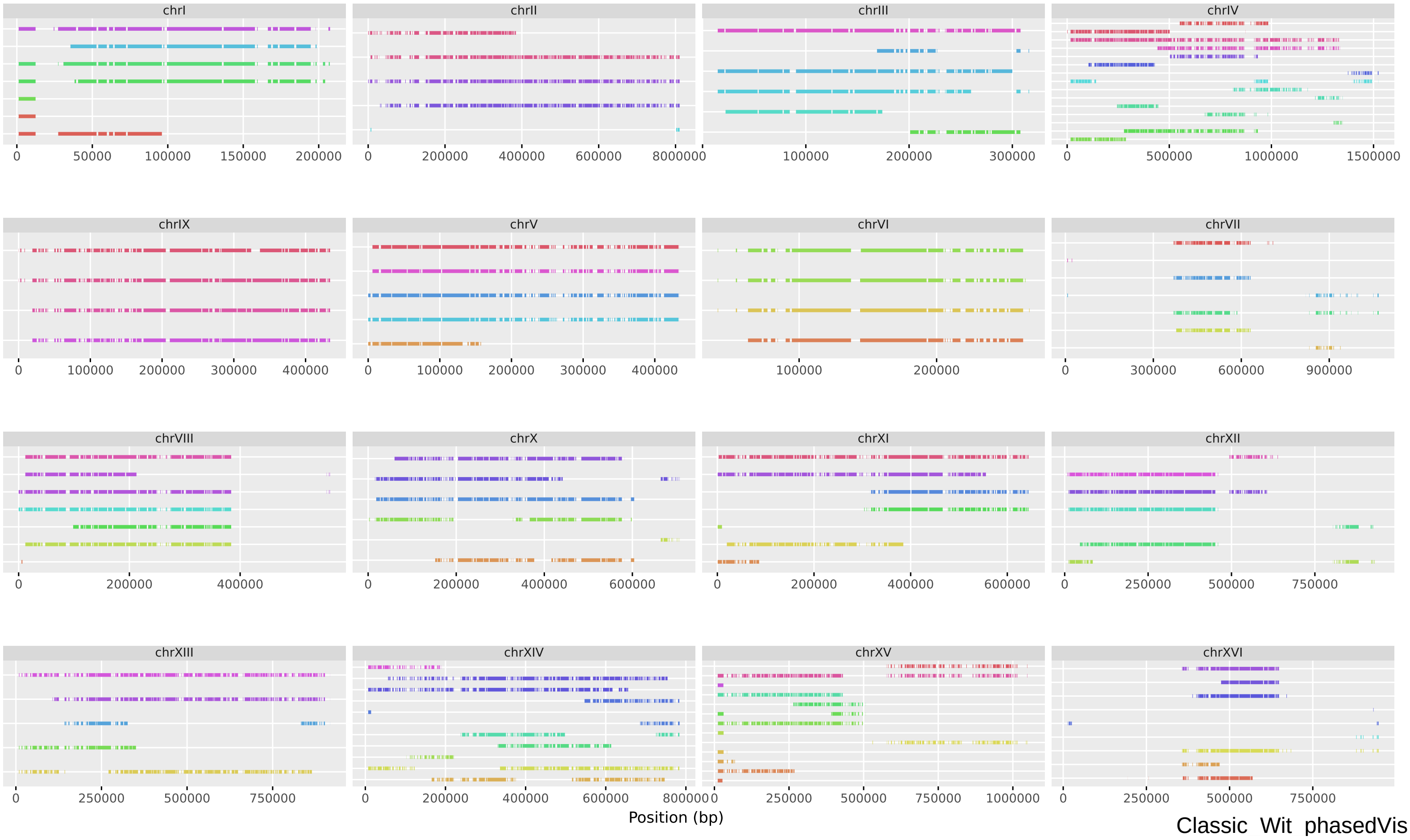

Supplementary Figure S1

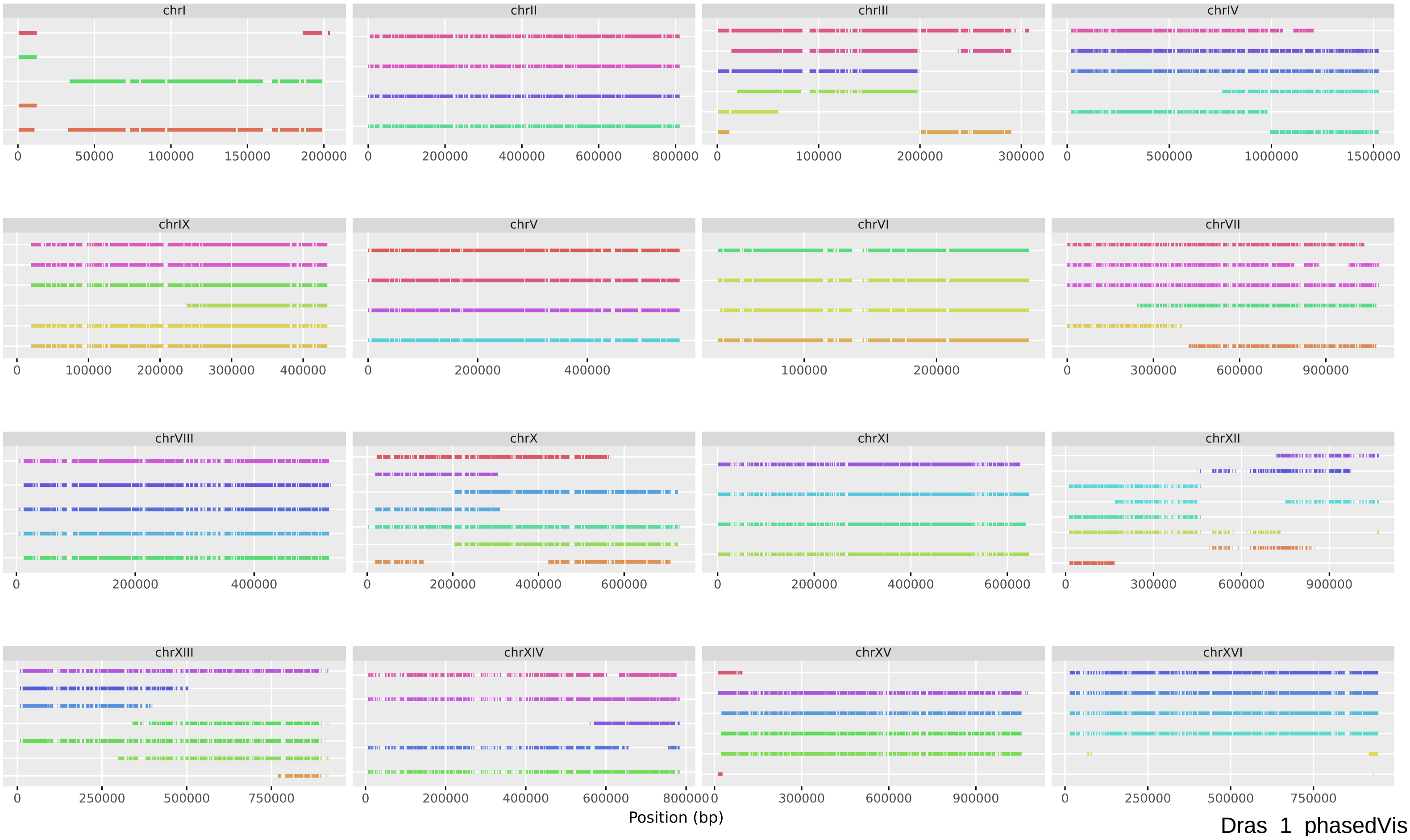

Supplementary Figure S1

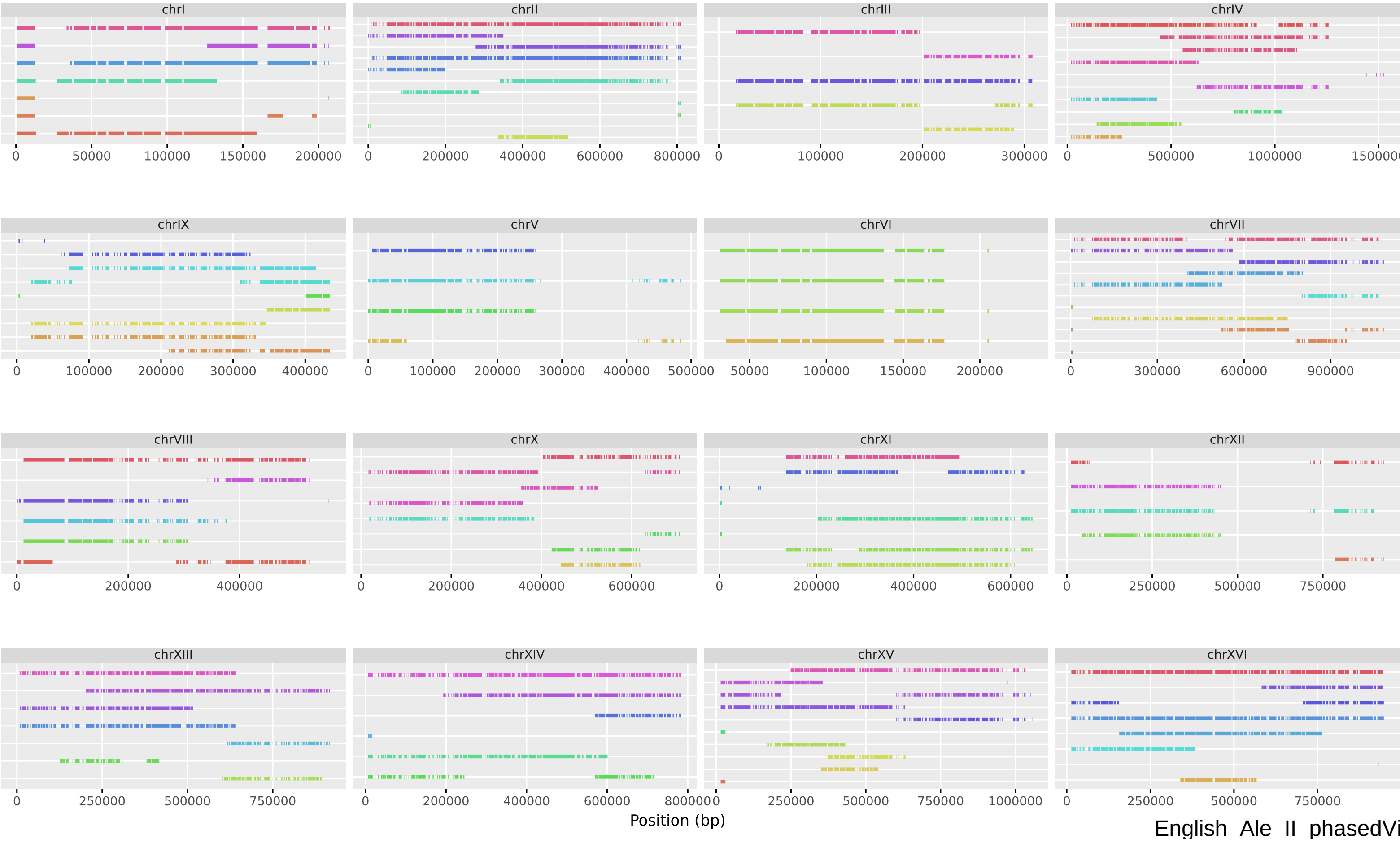

English Ale II phasedVis

Supplementary Figure S1

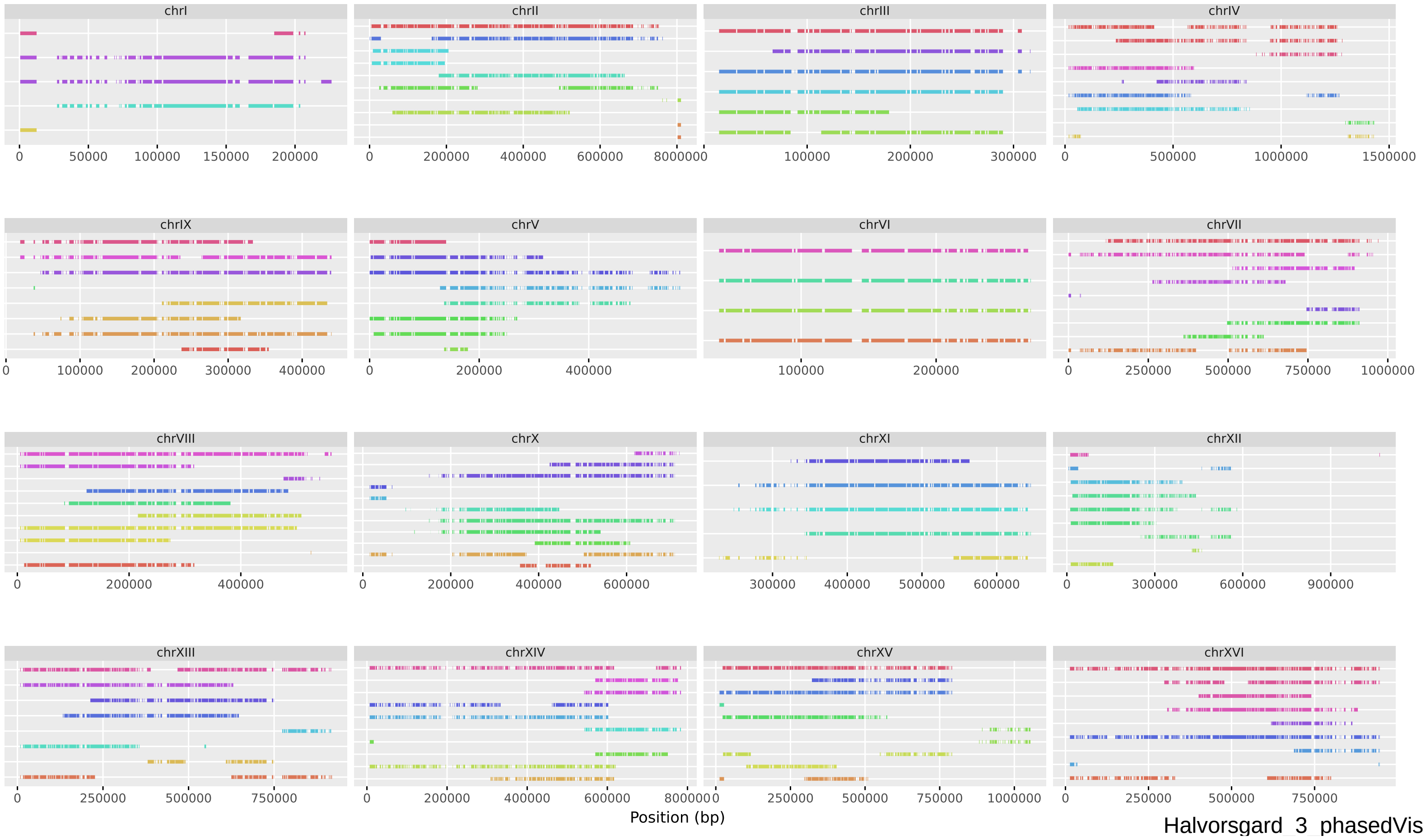

Supplementary Figure S1

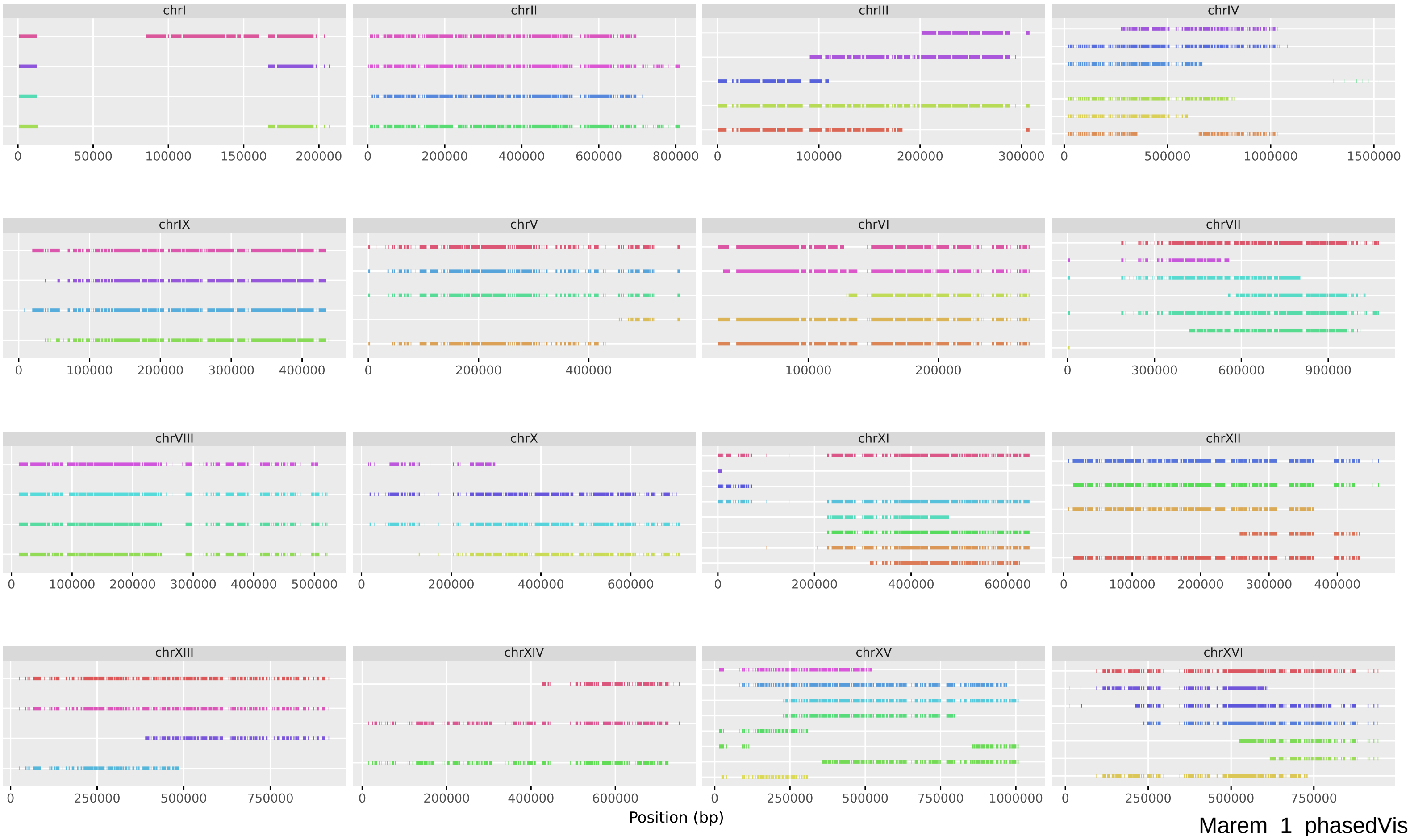

Supplementary Figure S1

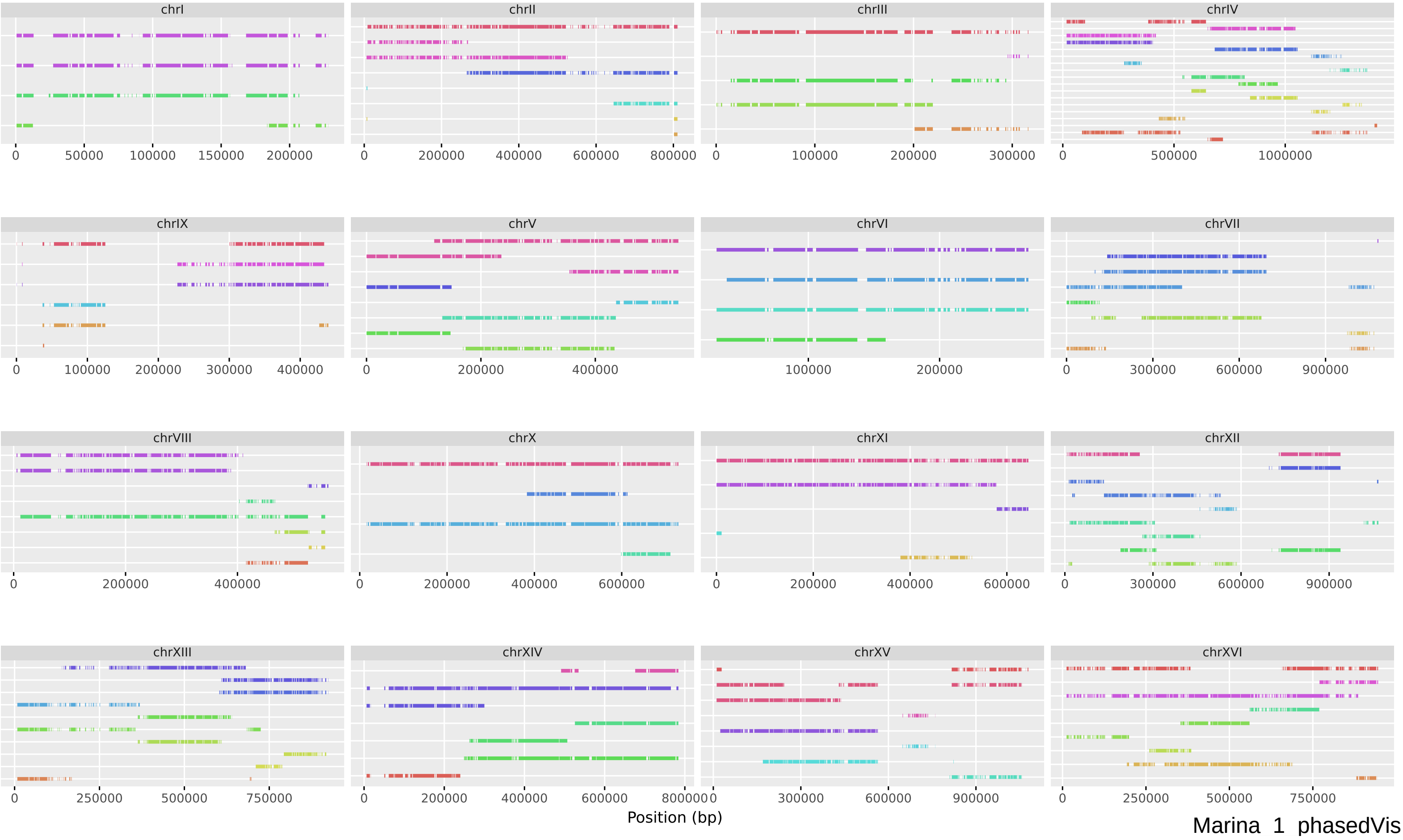

Supplementary Figure S1

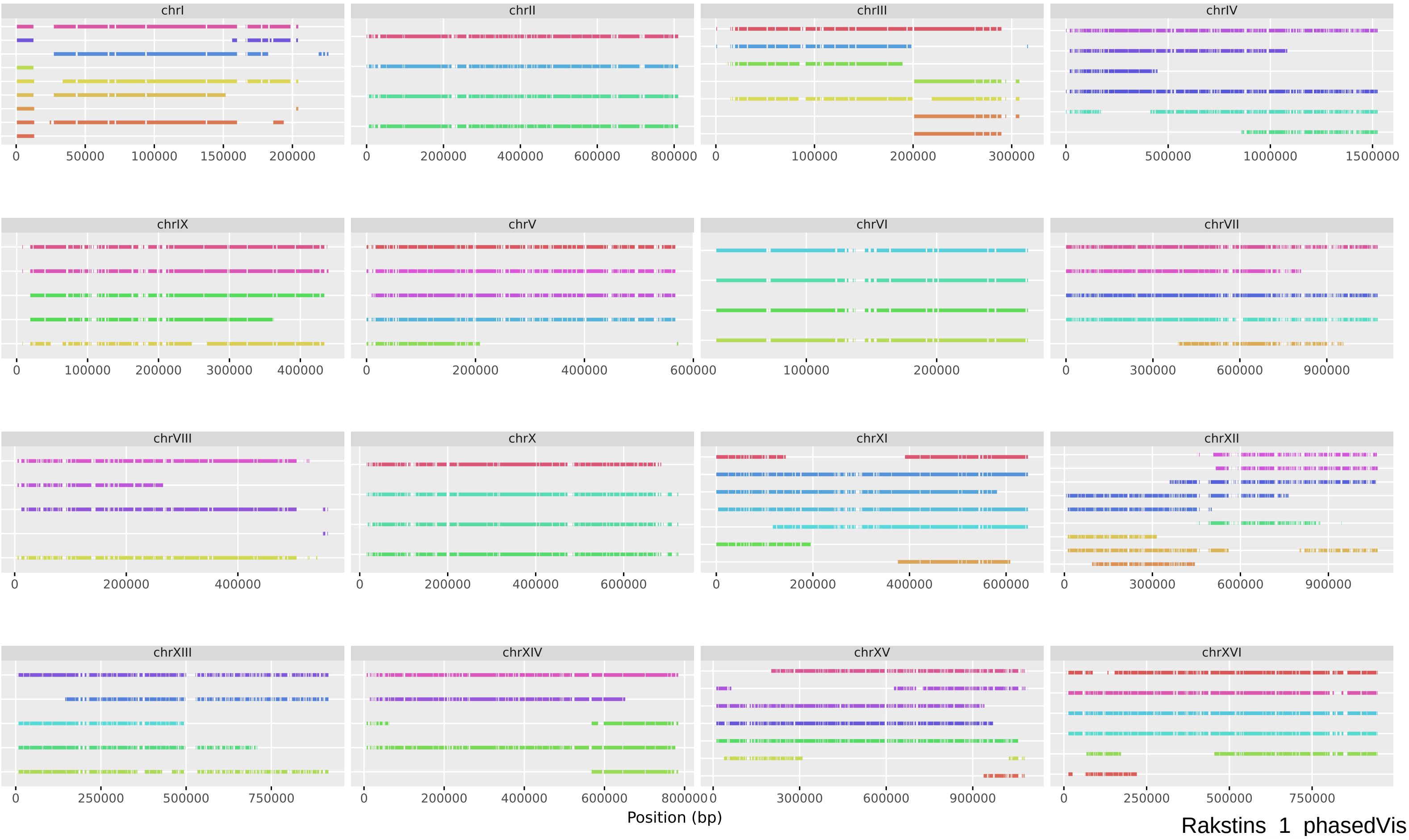

Supplementary Figure S1

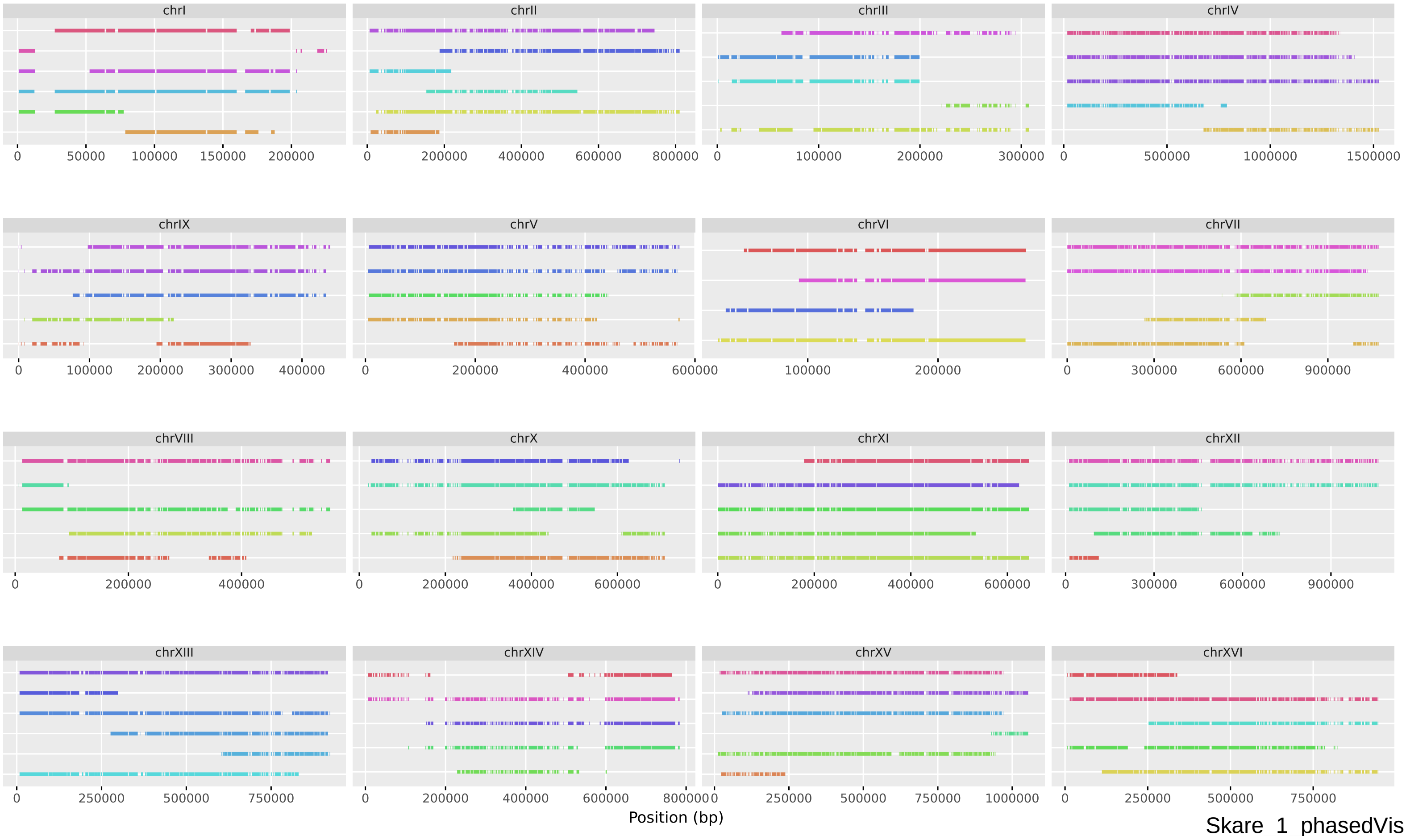

Skare 1 phasedVis

Supplementary Figure S1

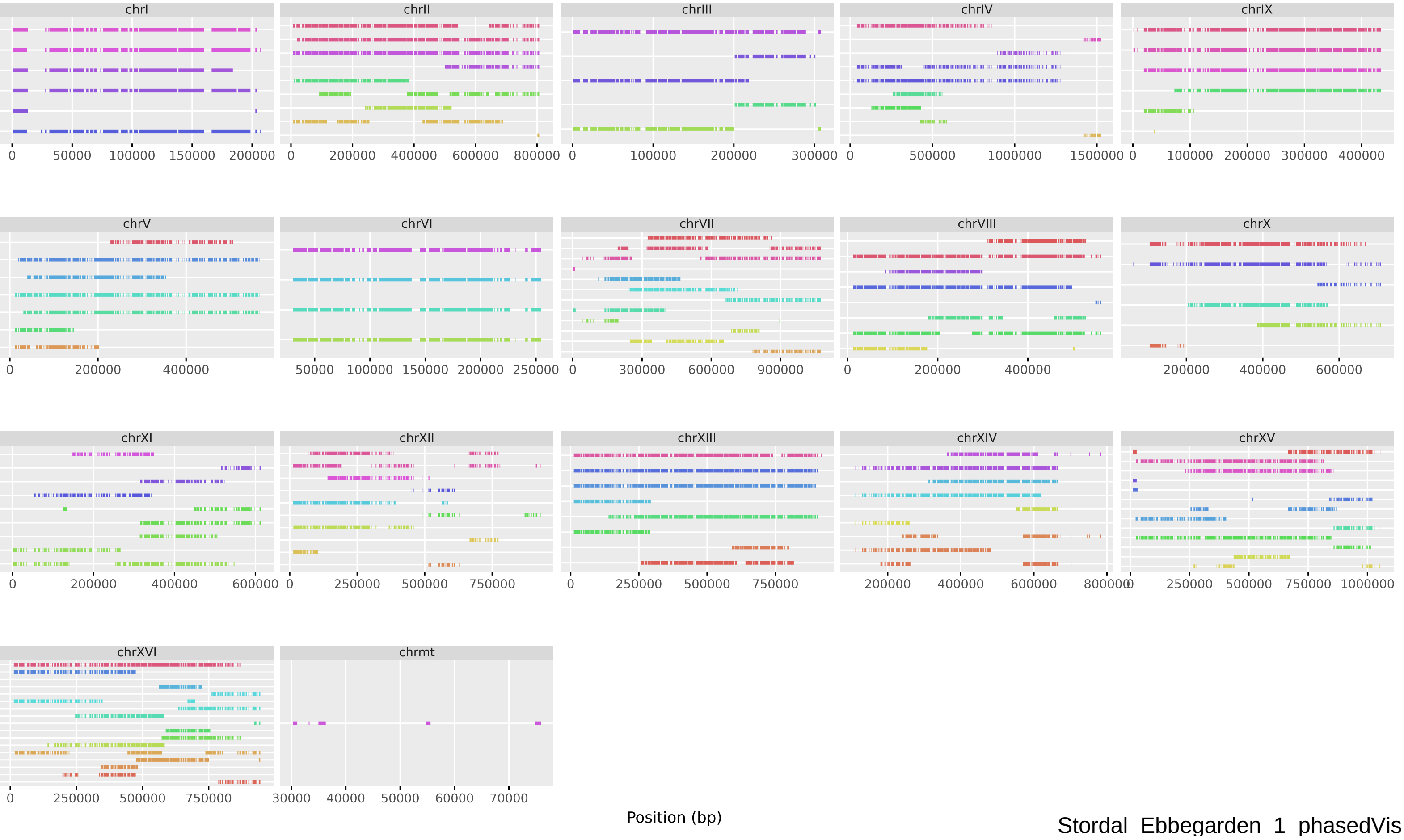

Supplementary Figure S1

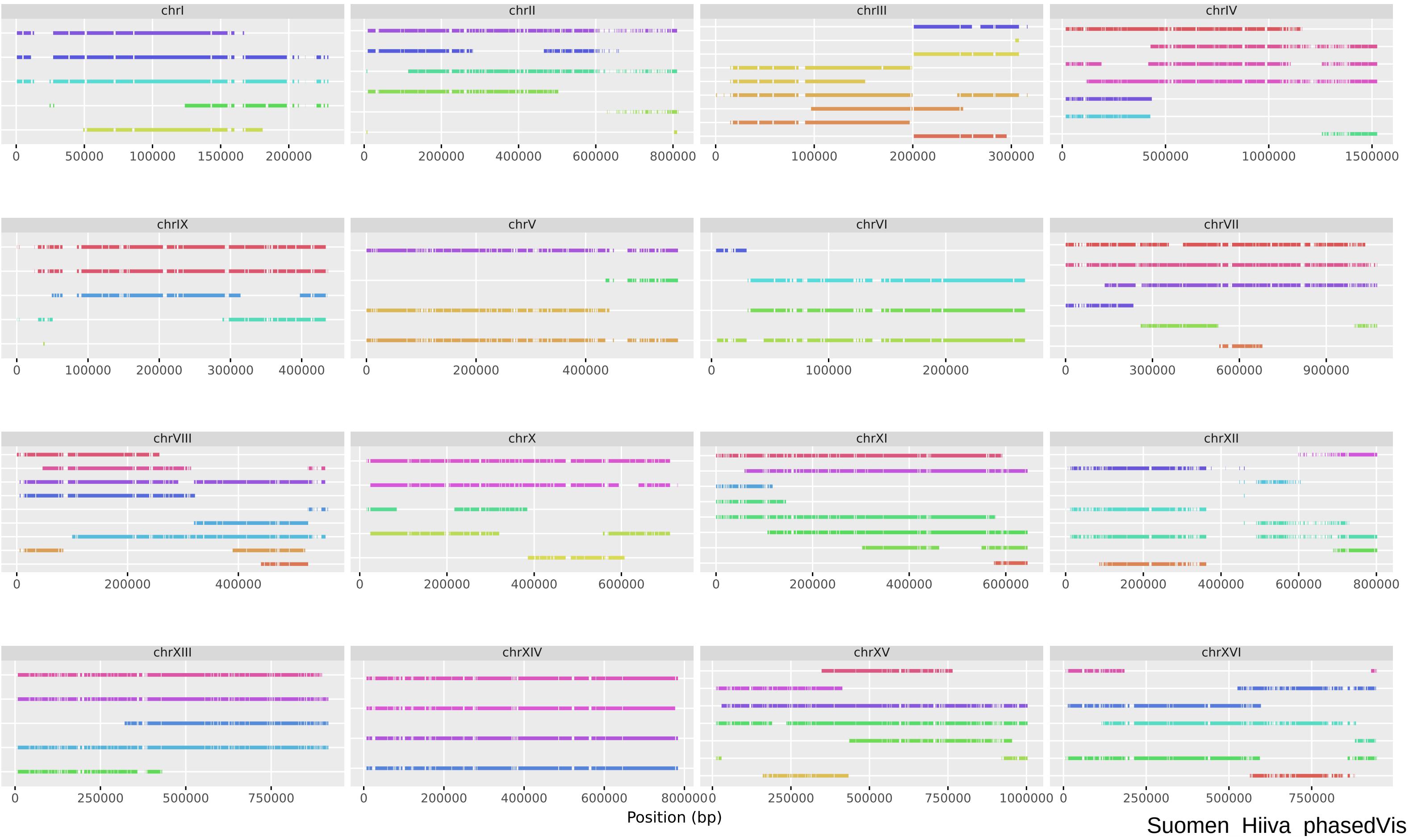

Supplementary Figure S1

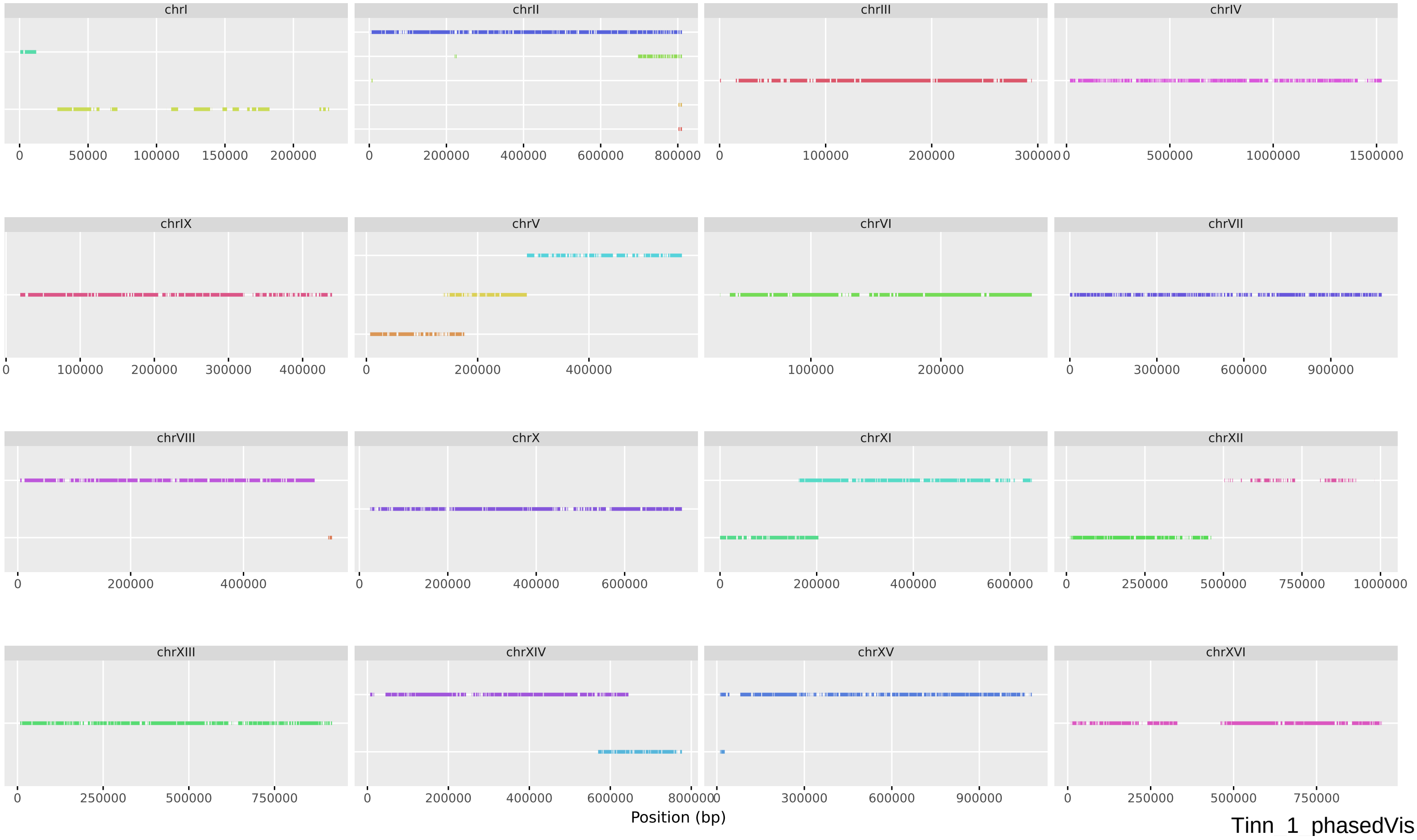

Tinn 1 phasedVis

Supplementary Figure S1

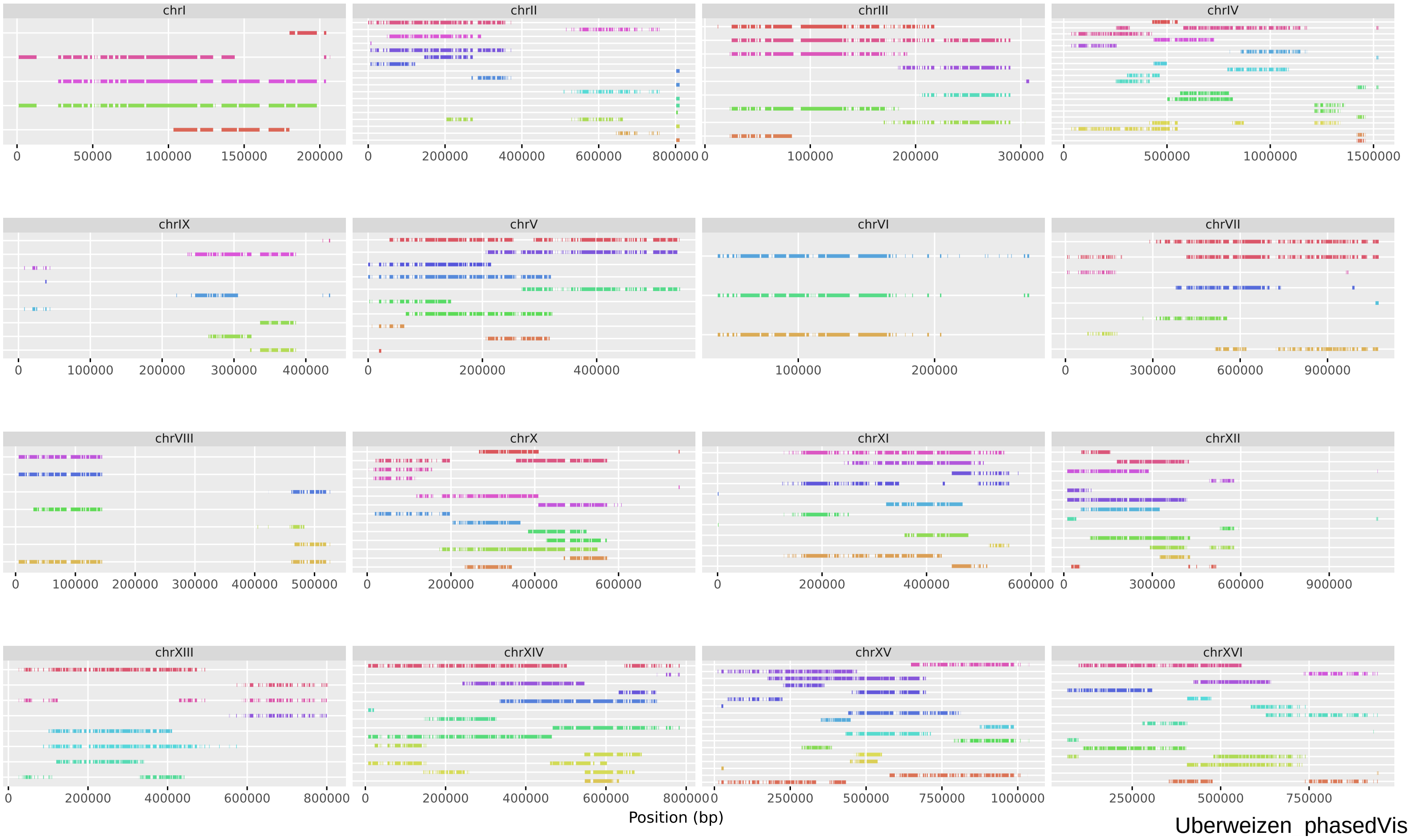

Supplementary Figure S1

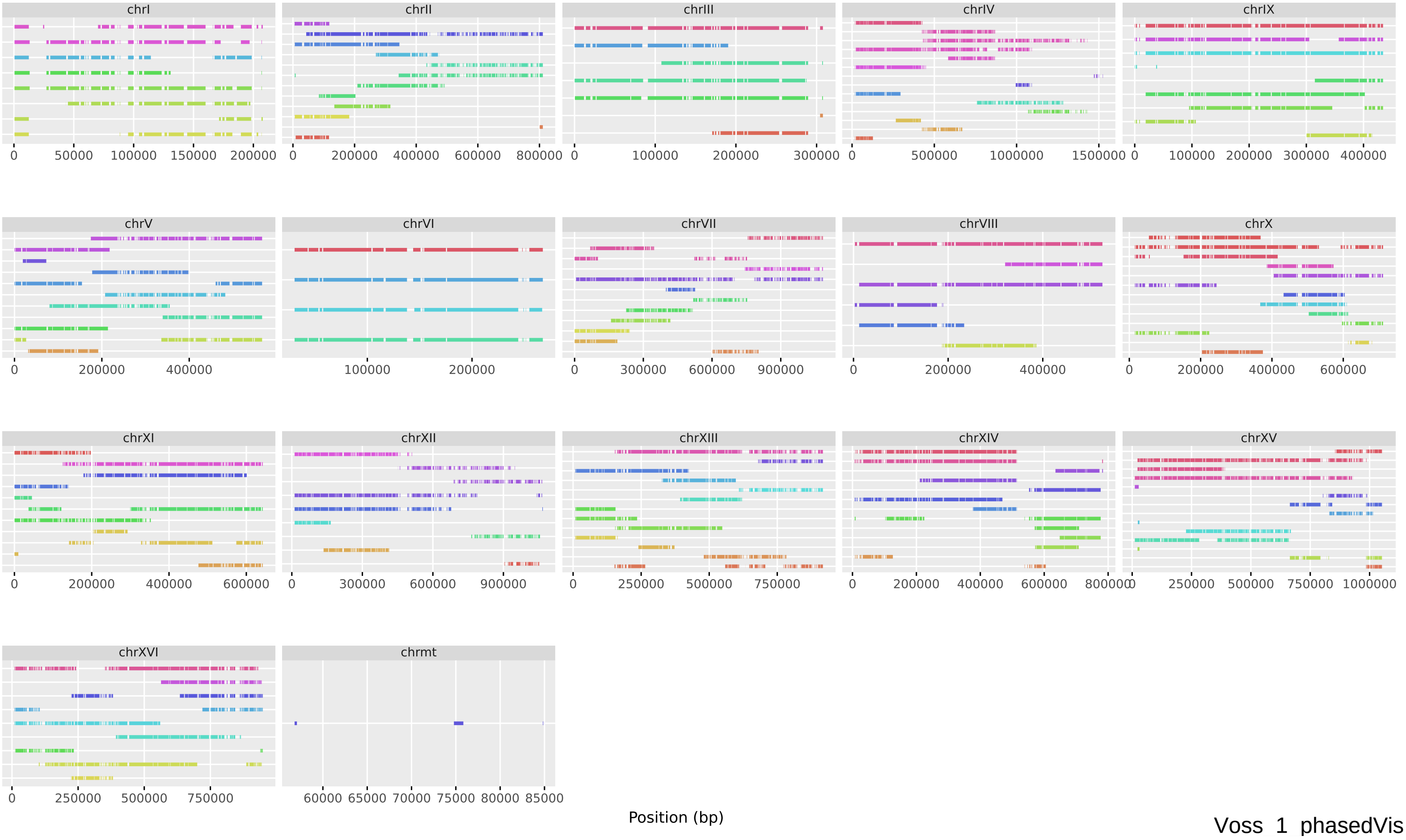

Voss_1_phasedVis

Supplementary Figure S1

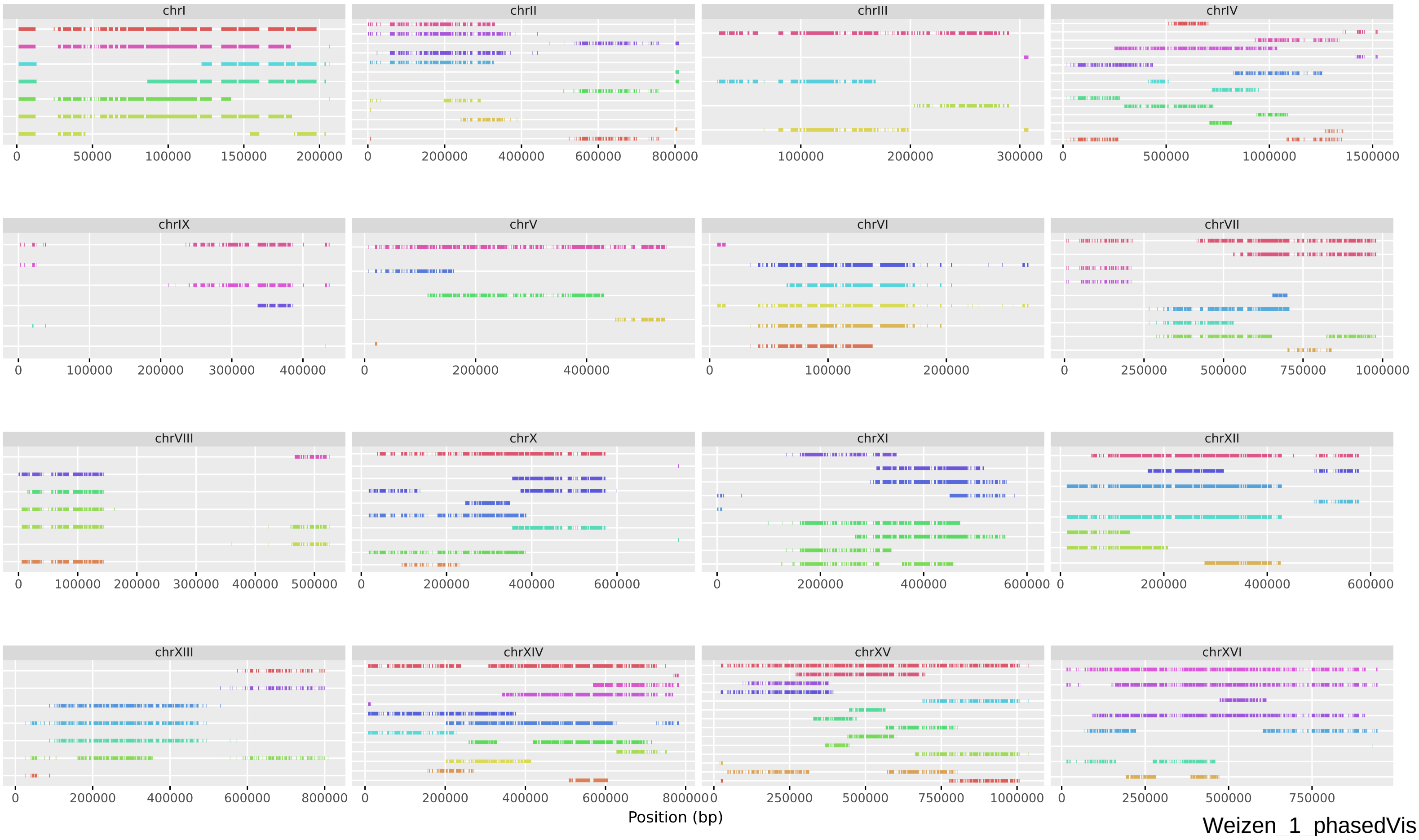

Supplementary Figure S2

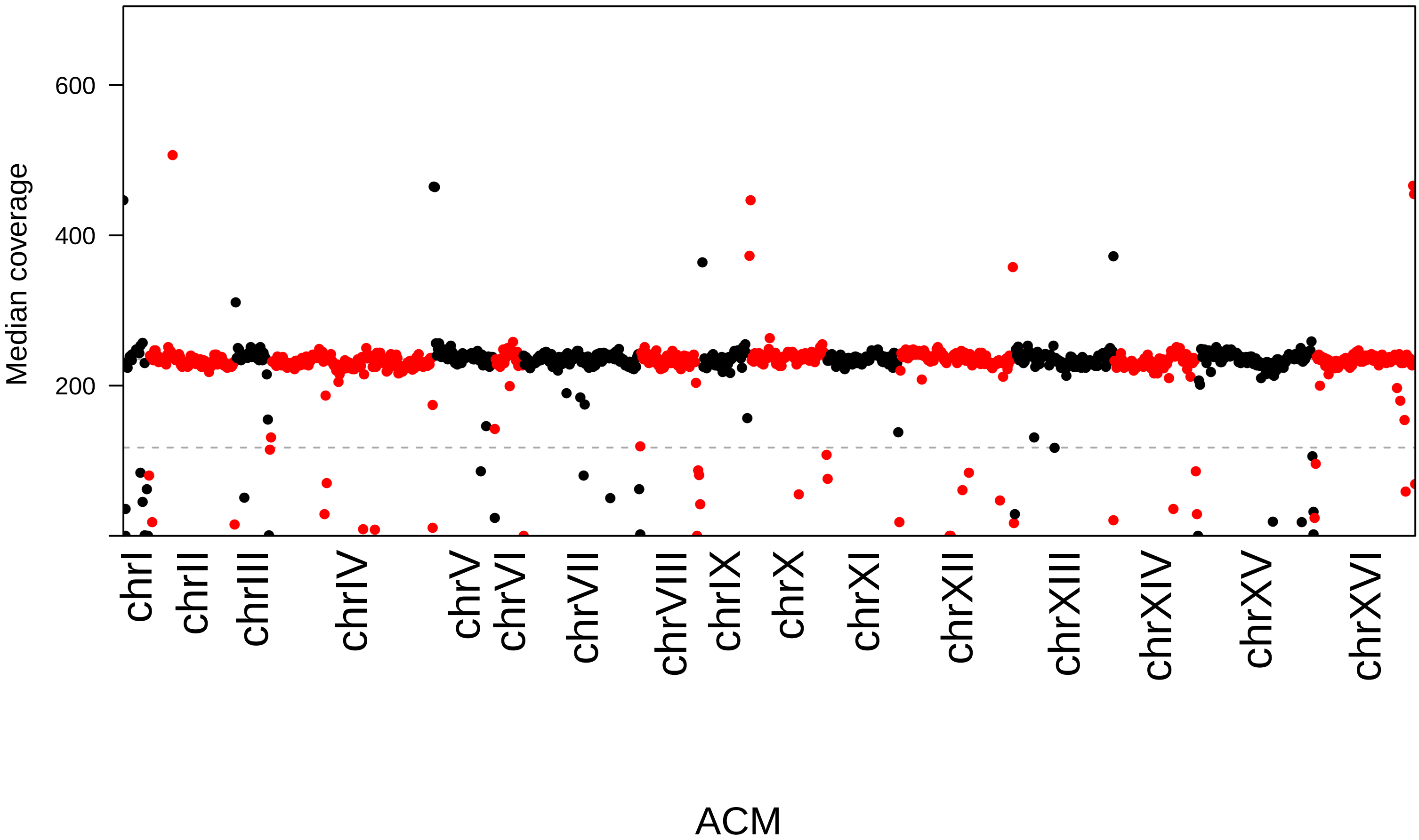

Supplementary Figure S2

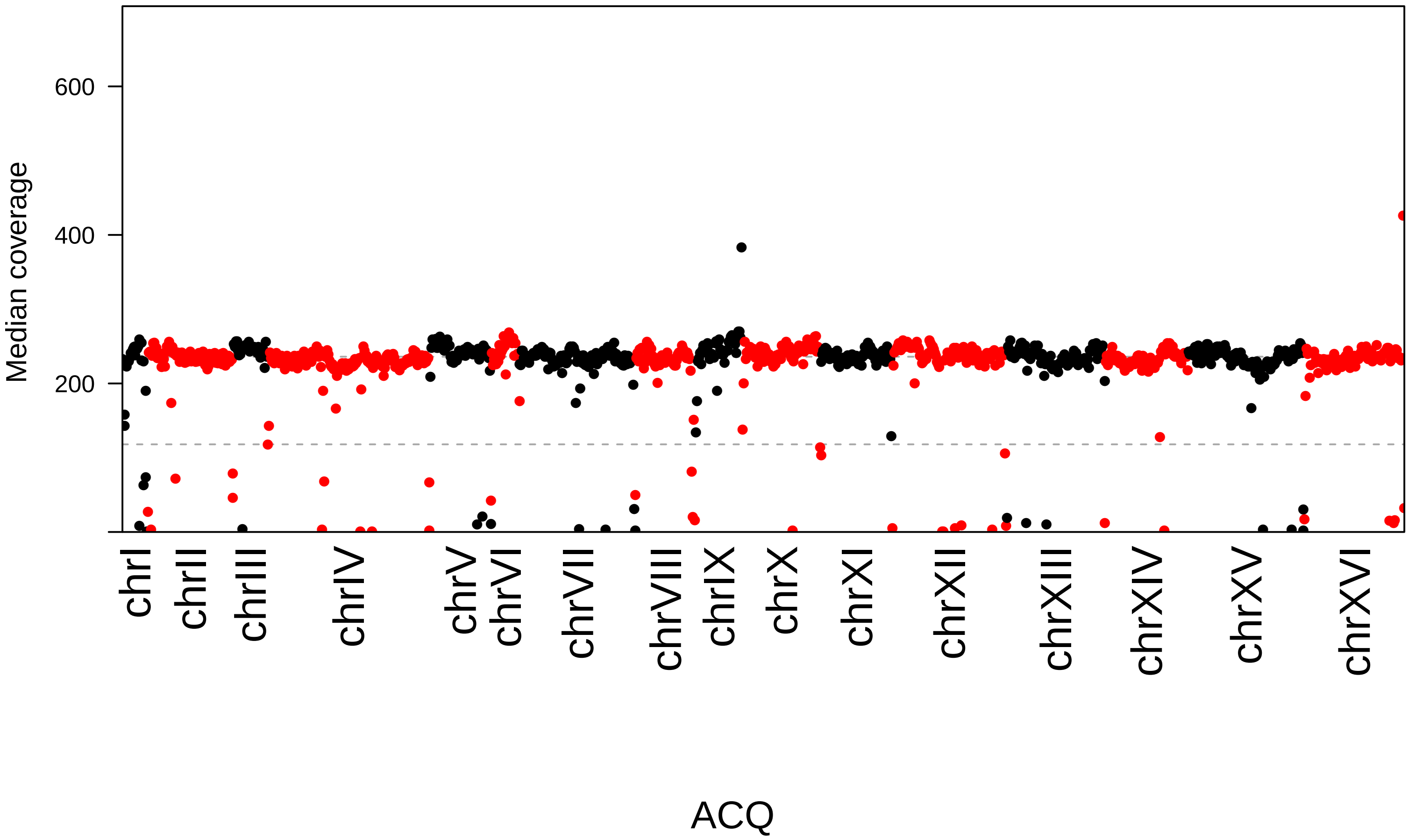

Supplementary Figure S2

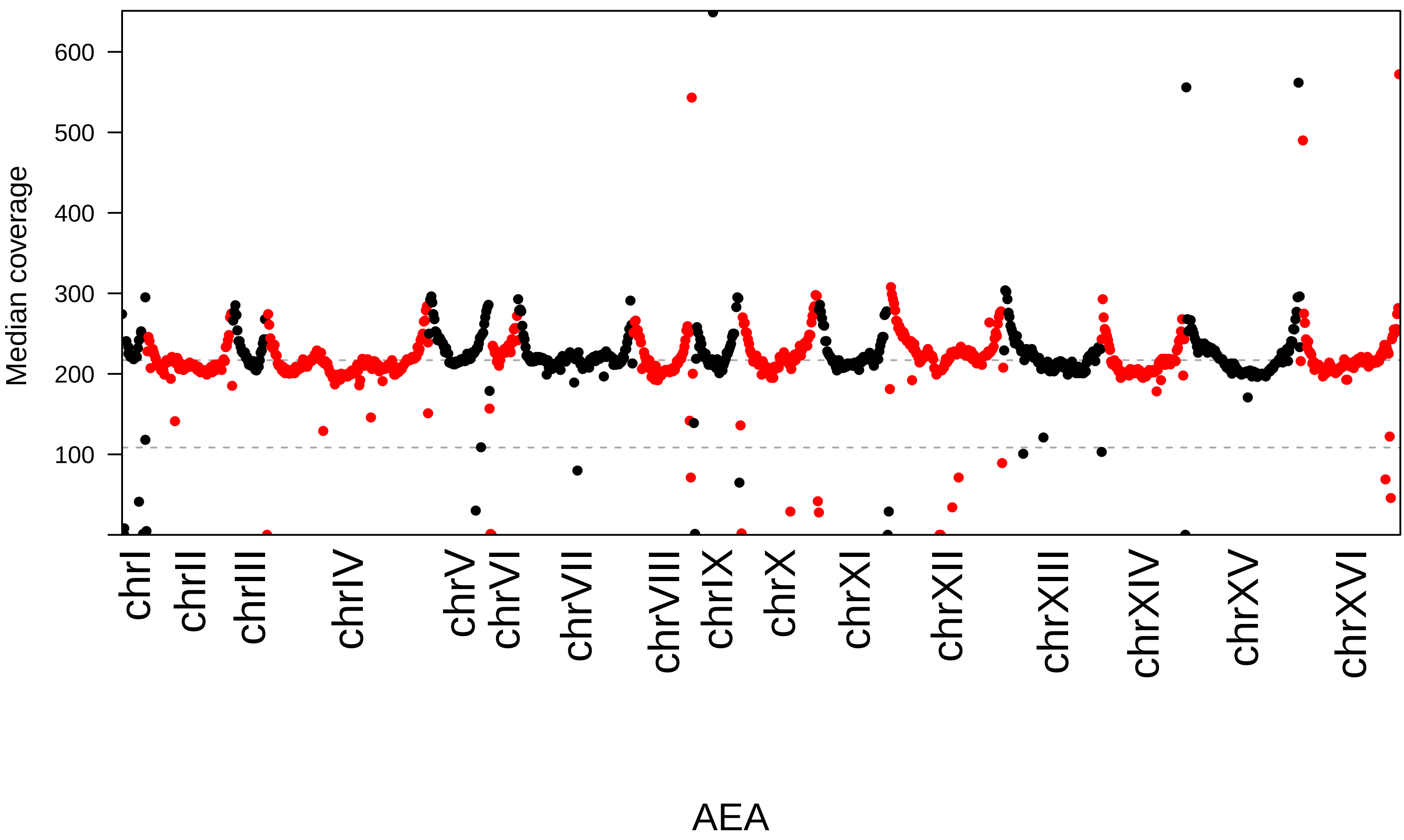

Supplementary Figure S2

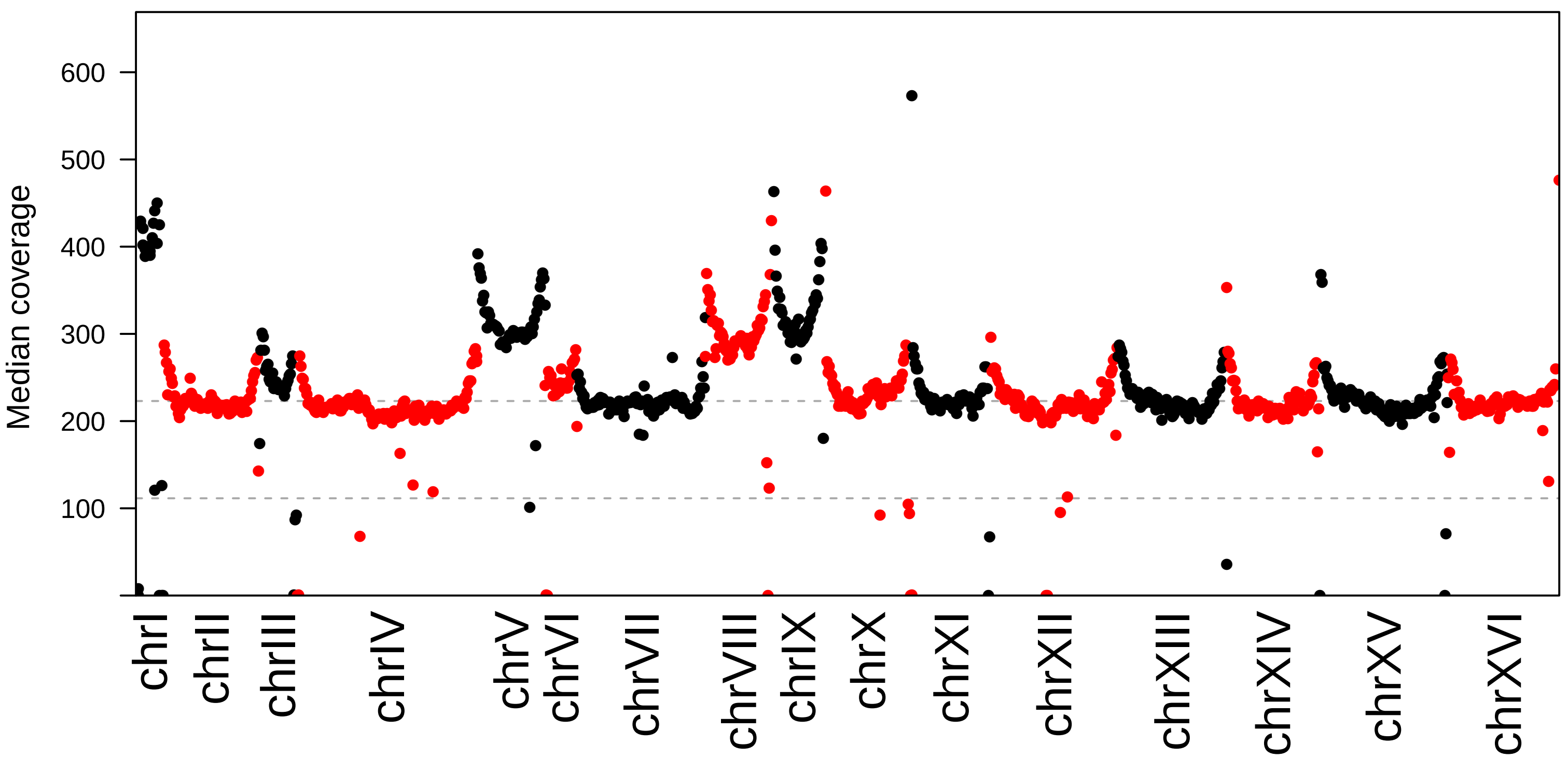

AEE

Supplementary Figure S2

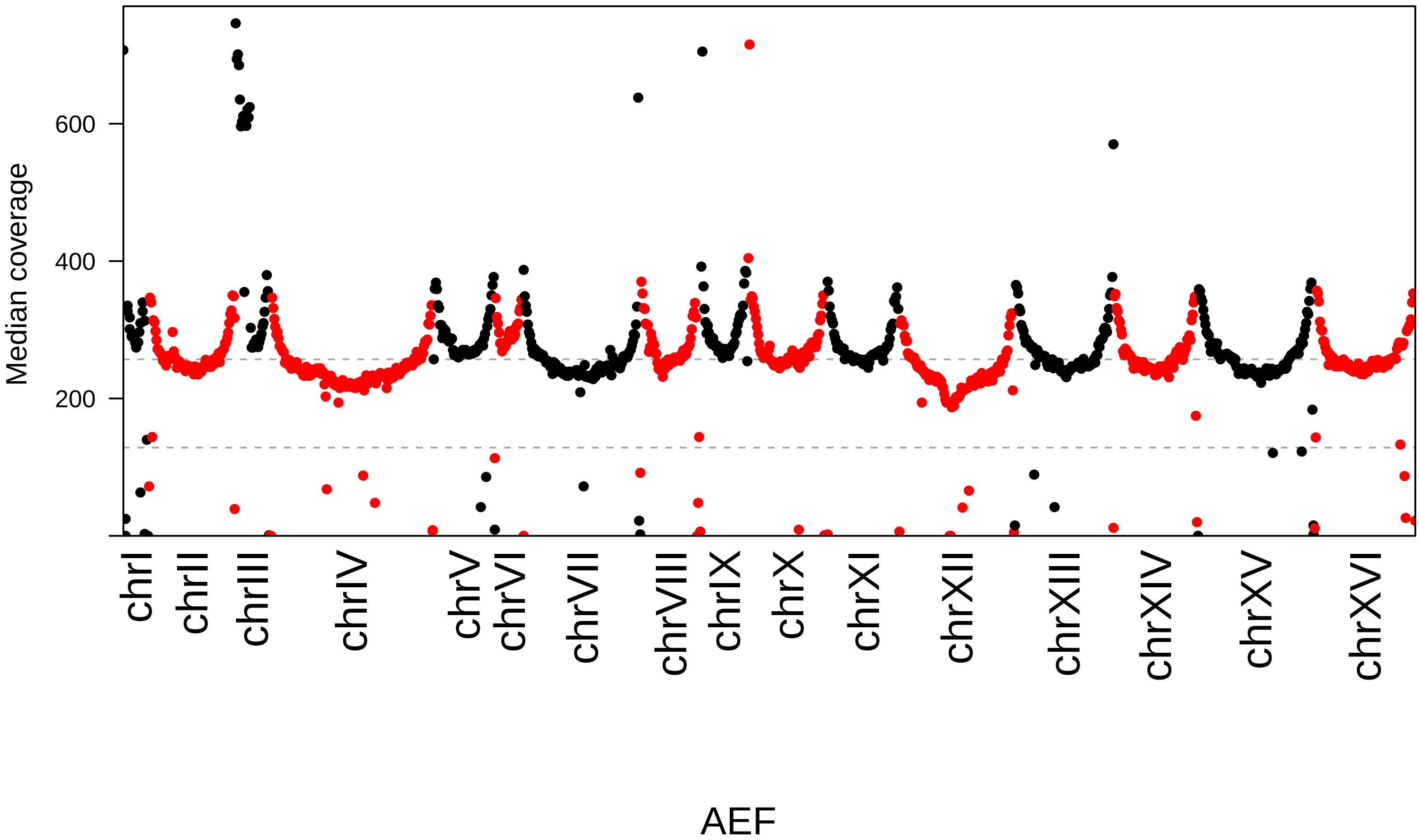

Supplementary Figure S2

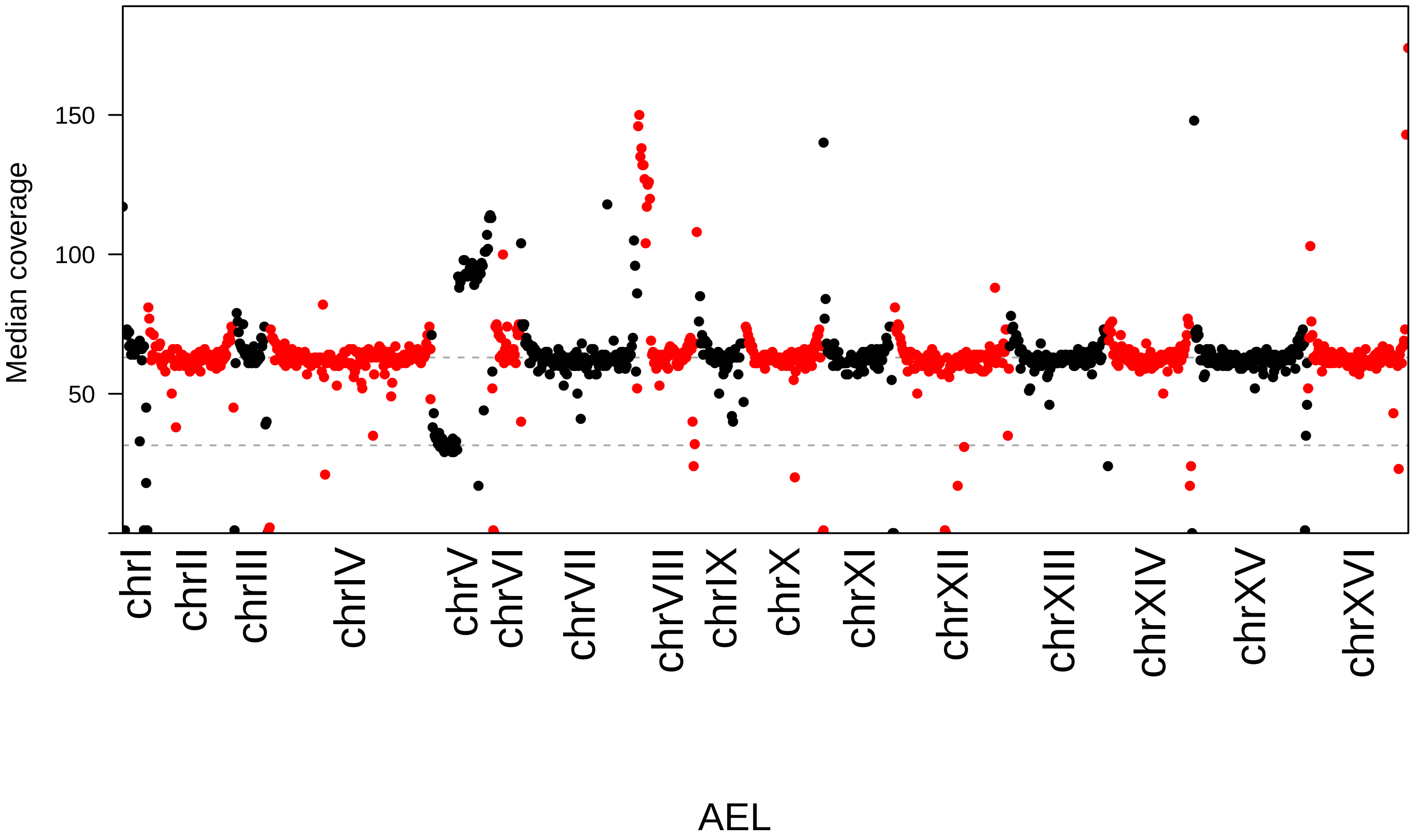

Supplementary Figure S2

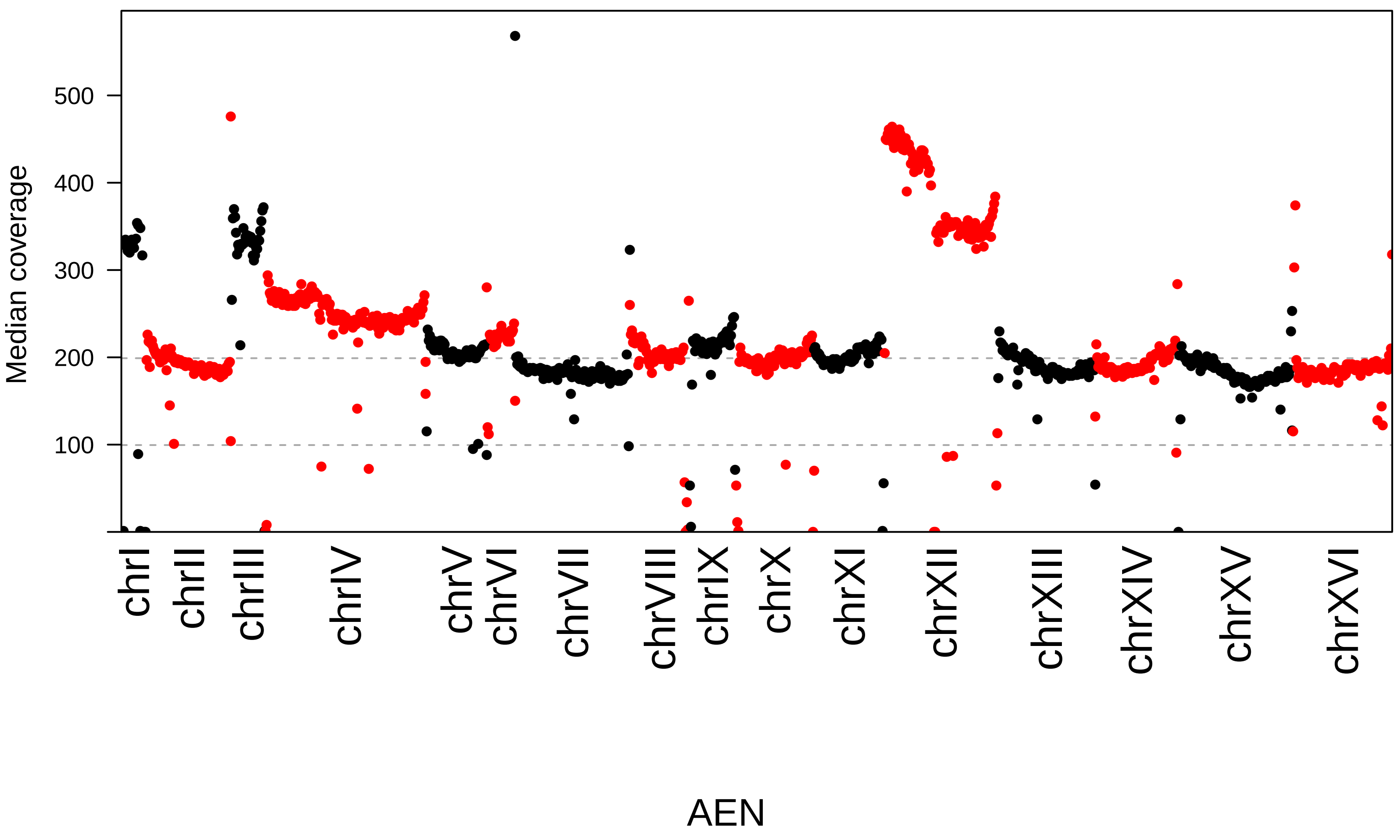

Supplementary Figure S2

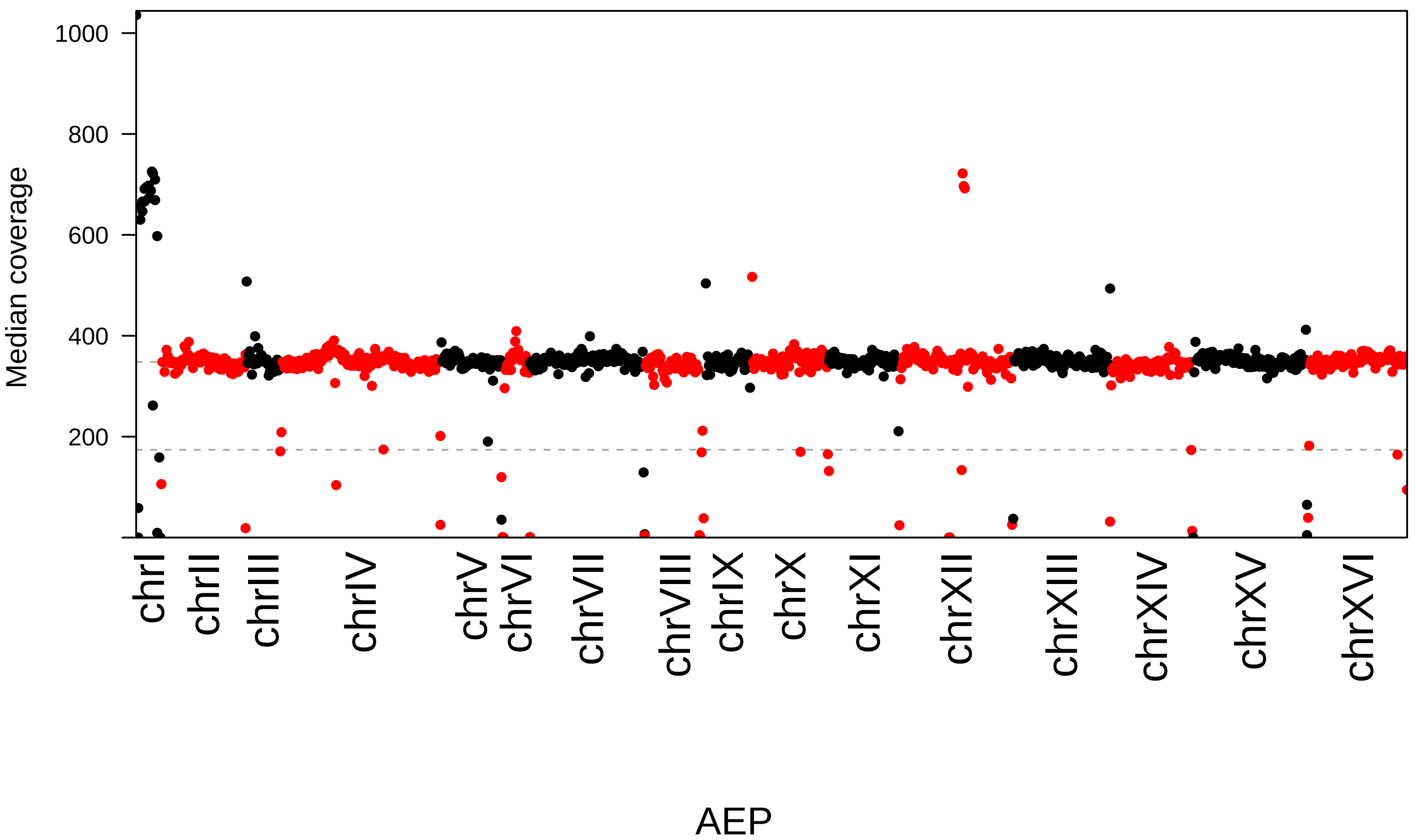

Supplementary Figure S2

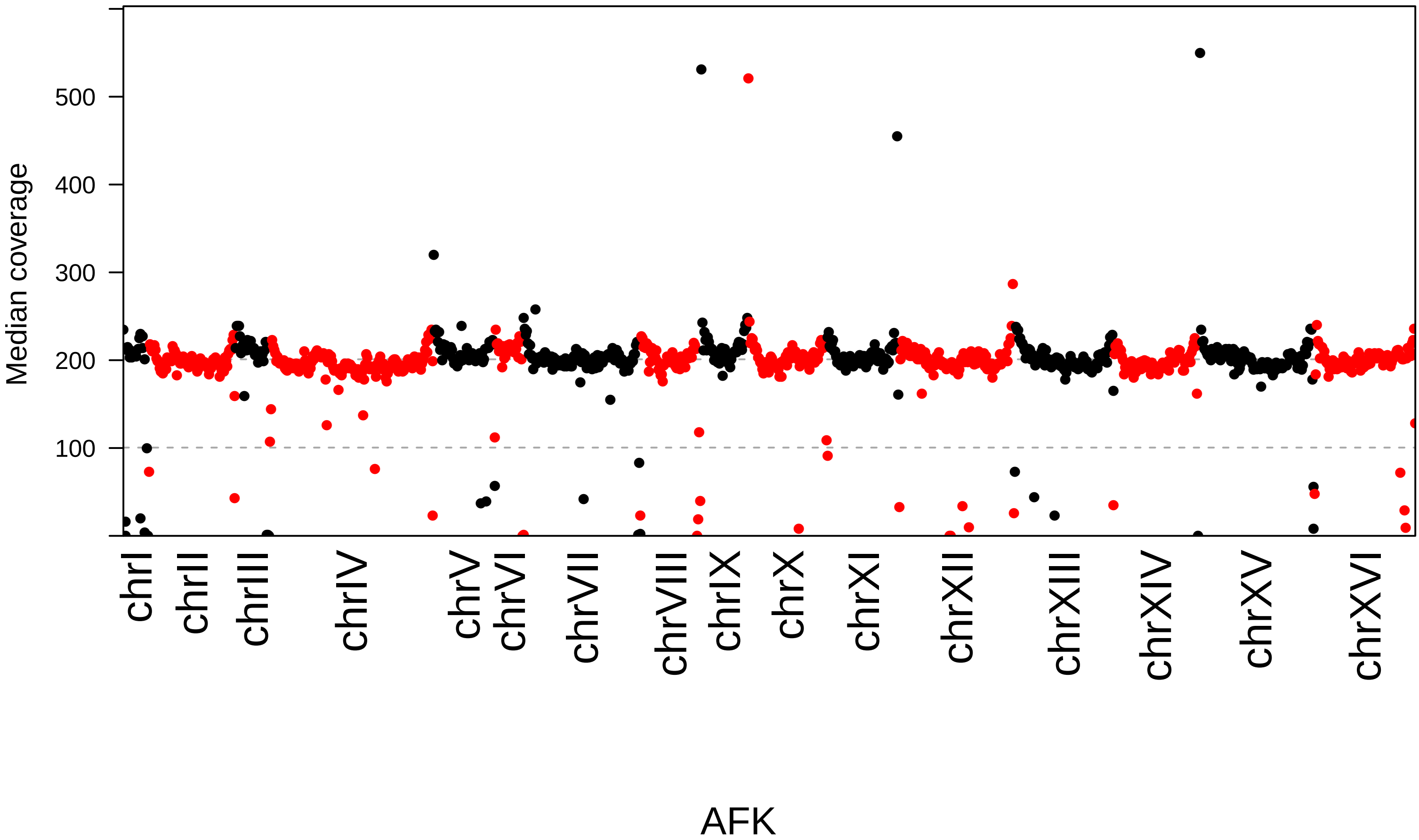

Supplementary Figure S2

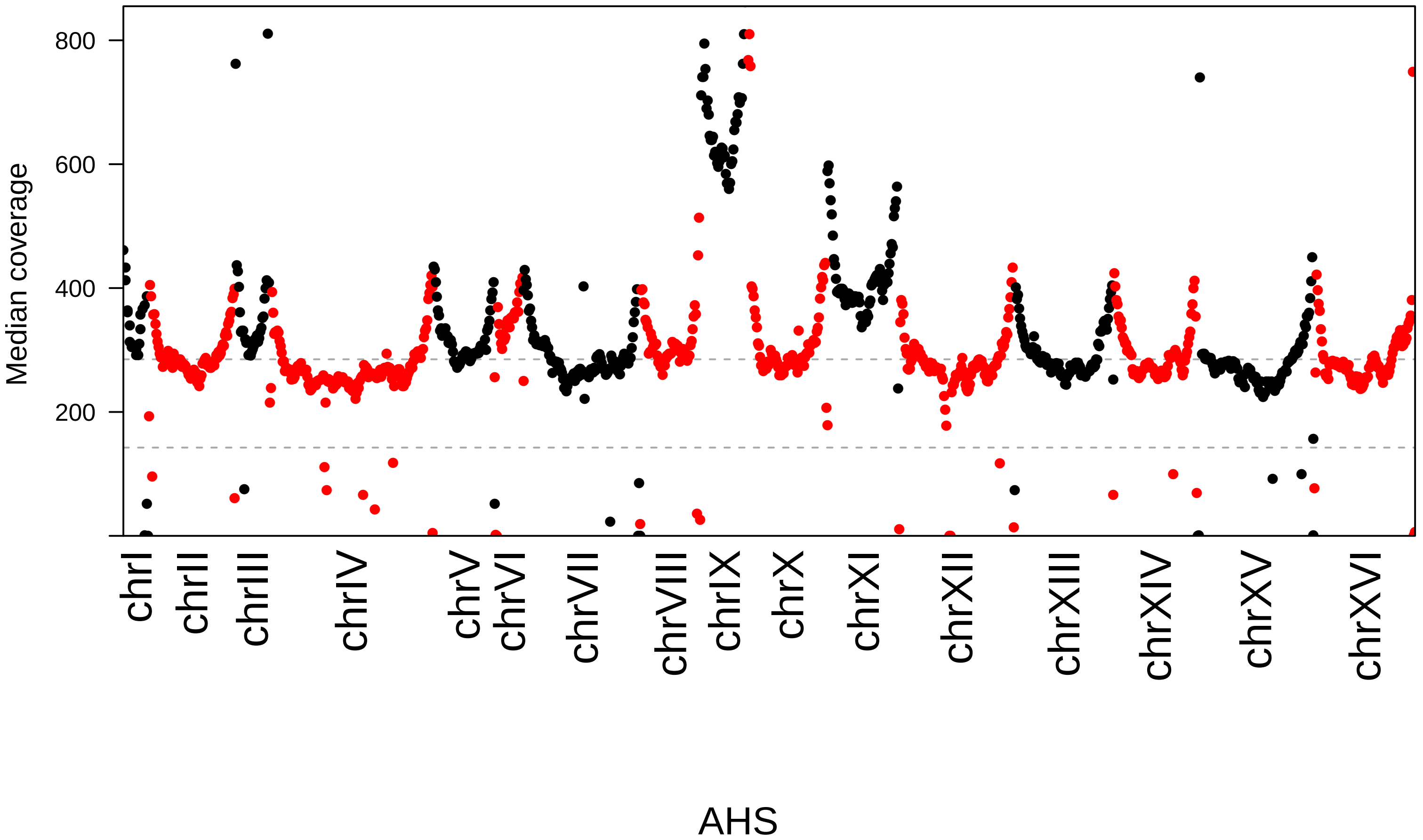

Supplementary Figure S2

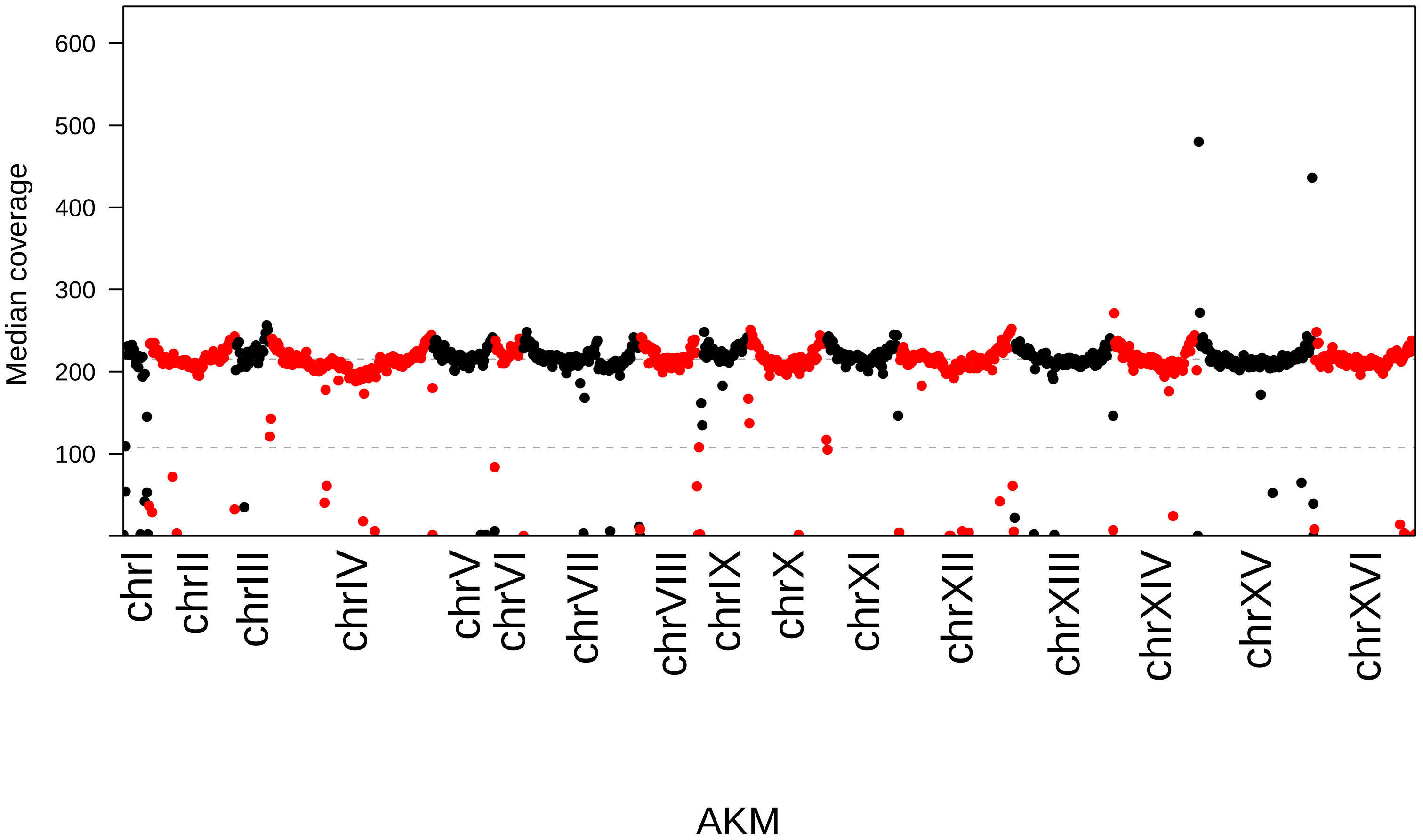

Supplementary Figure S2

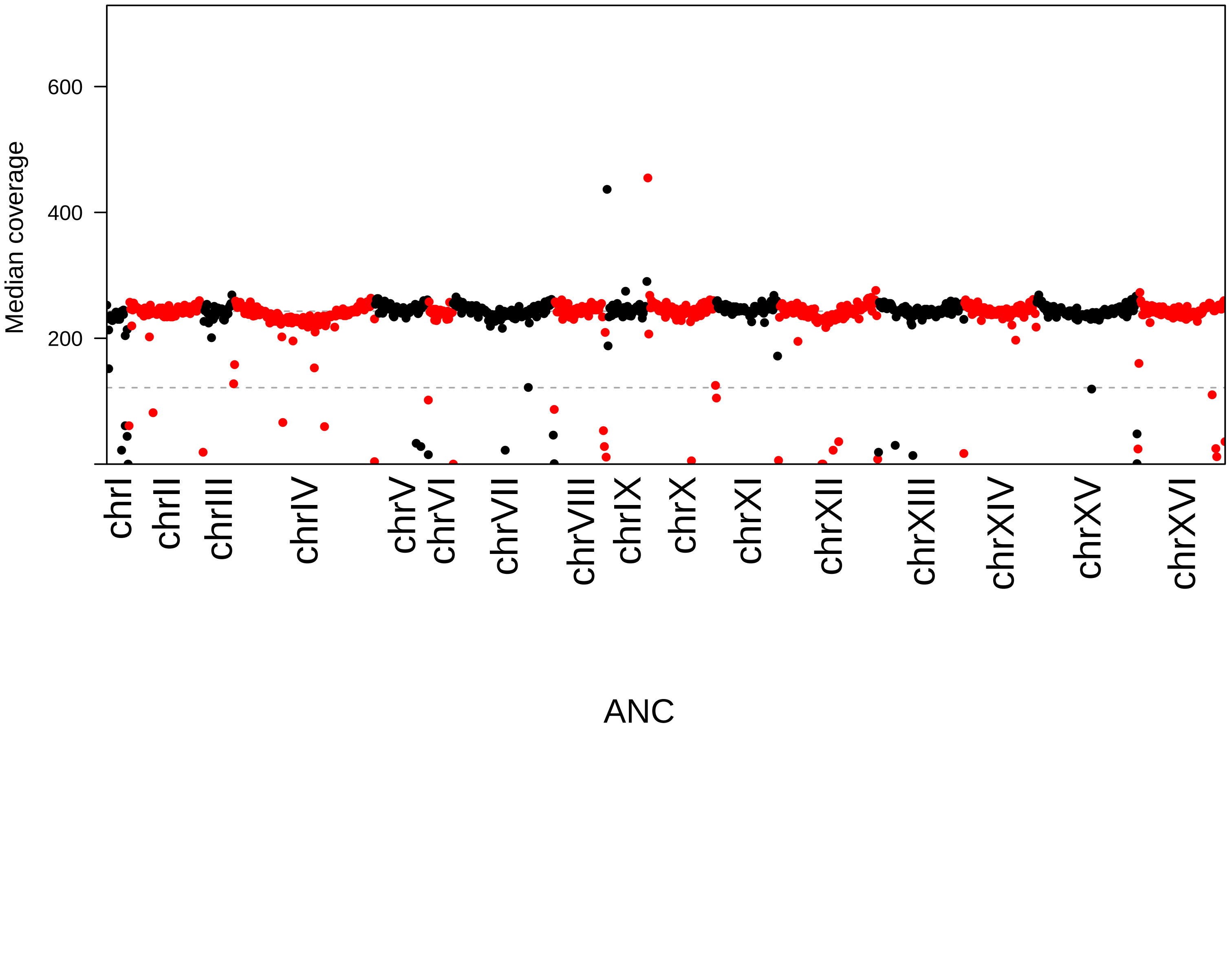

Supplementary Figure S2

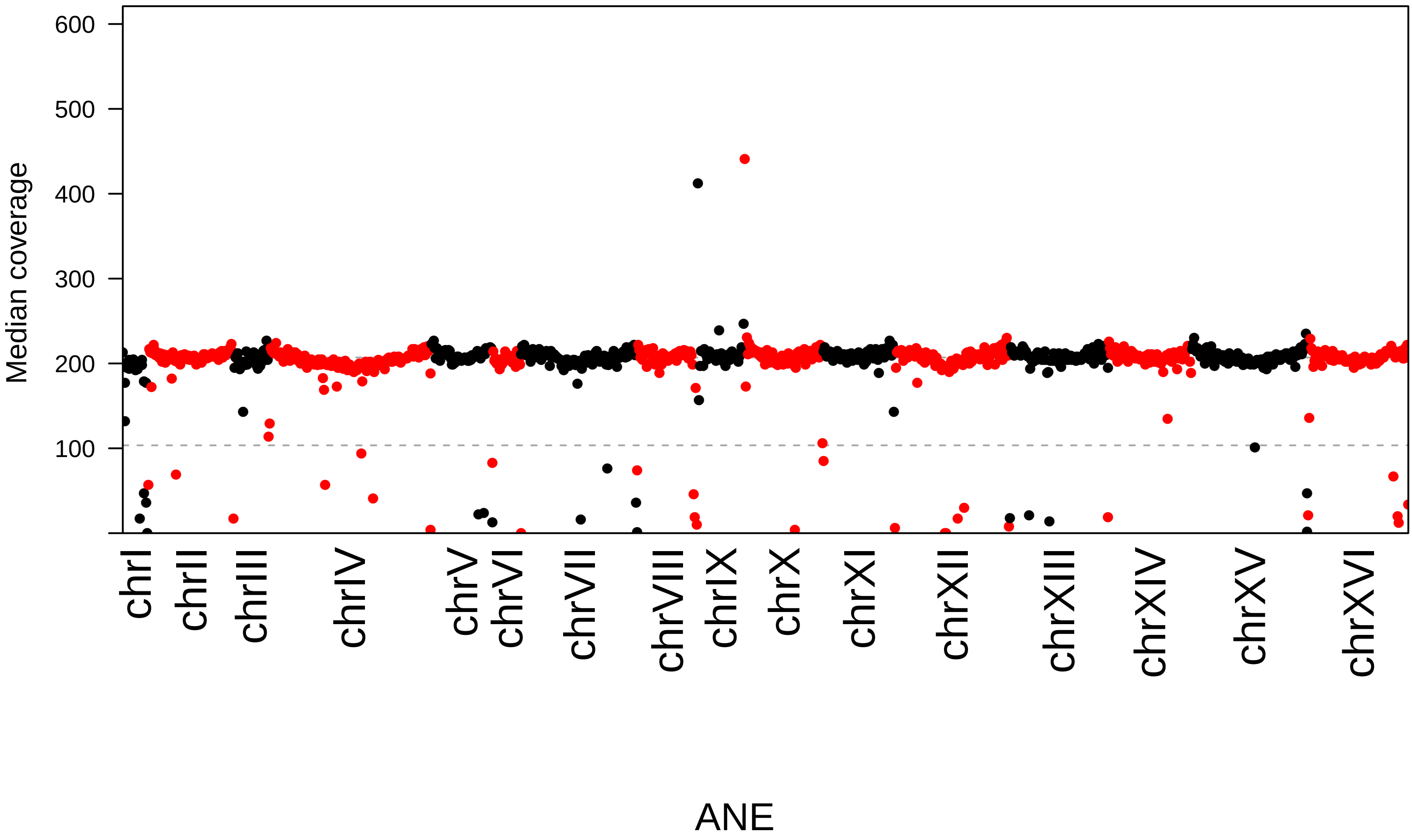

Supplementary Figure S2

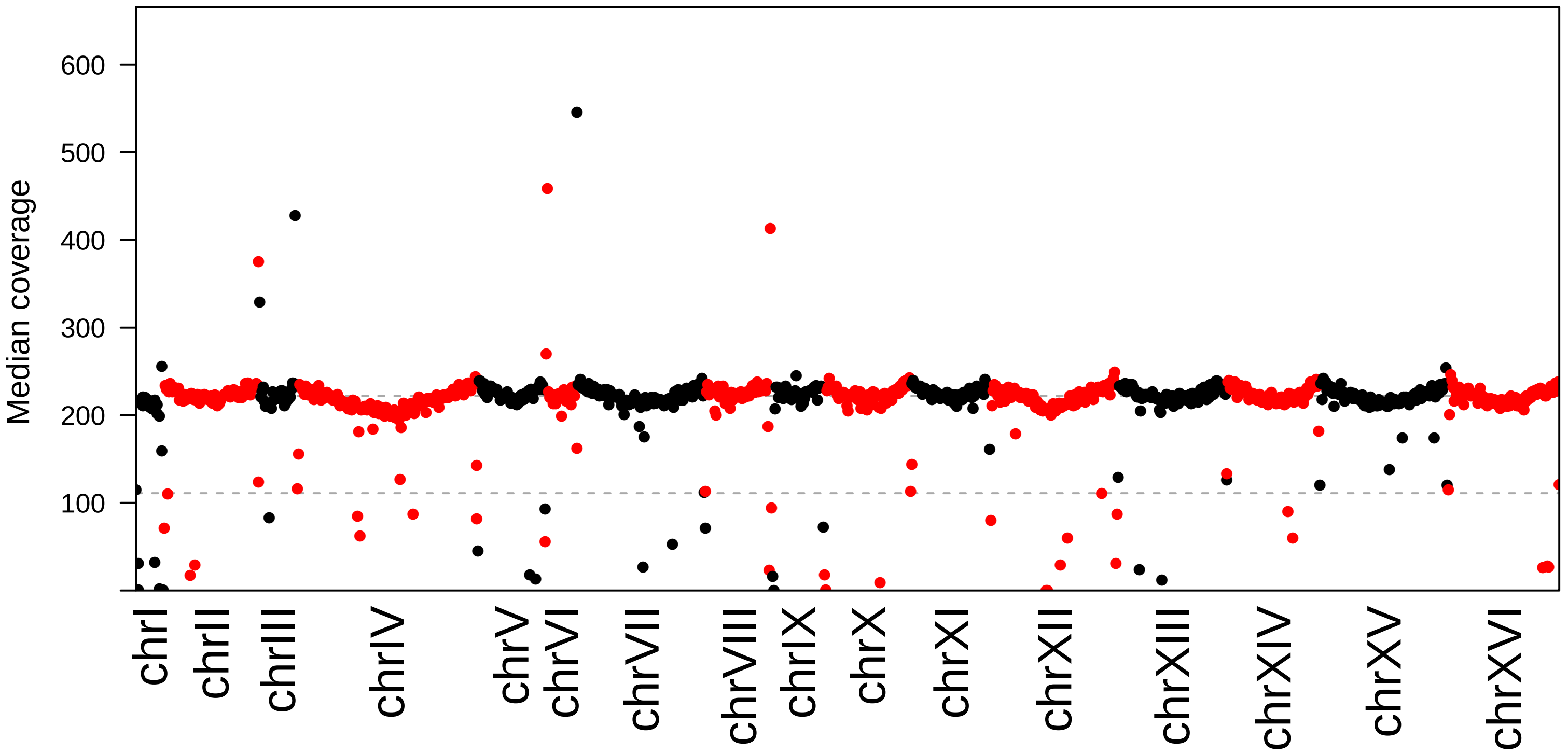

ANN

Supplementary Figure S2

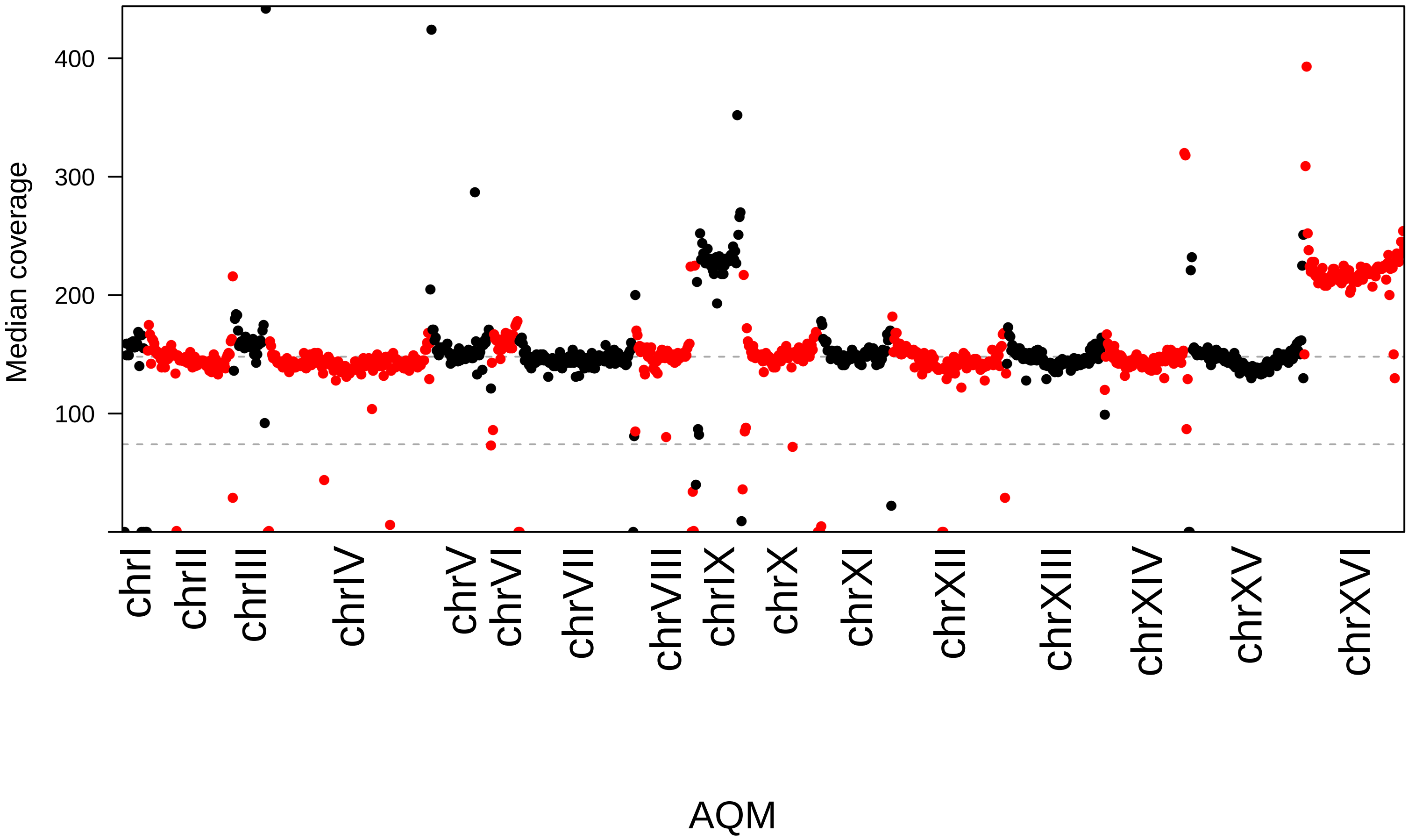

Supplementary Figure S2

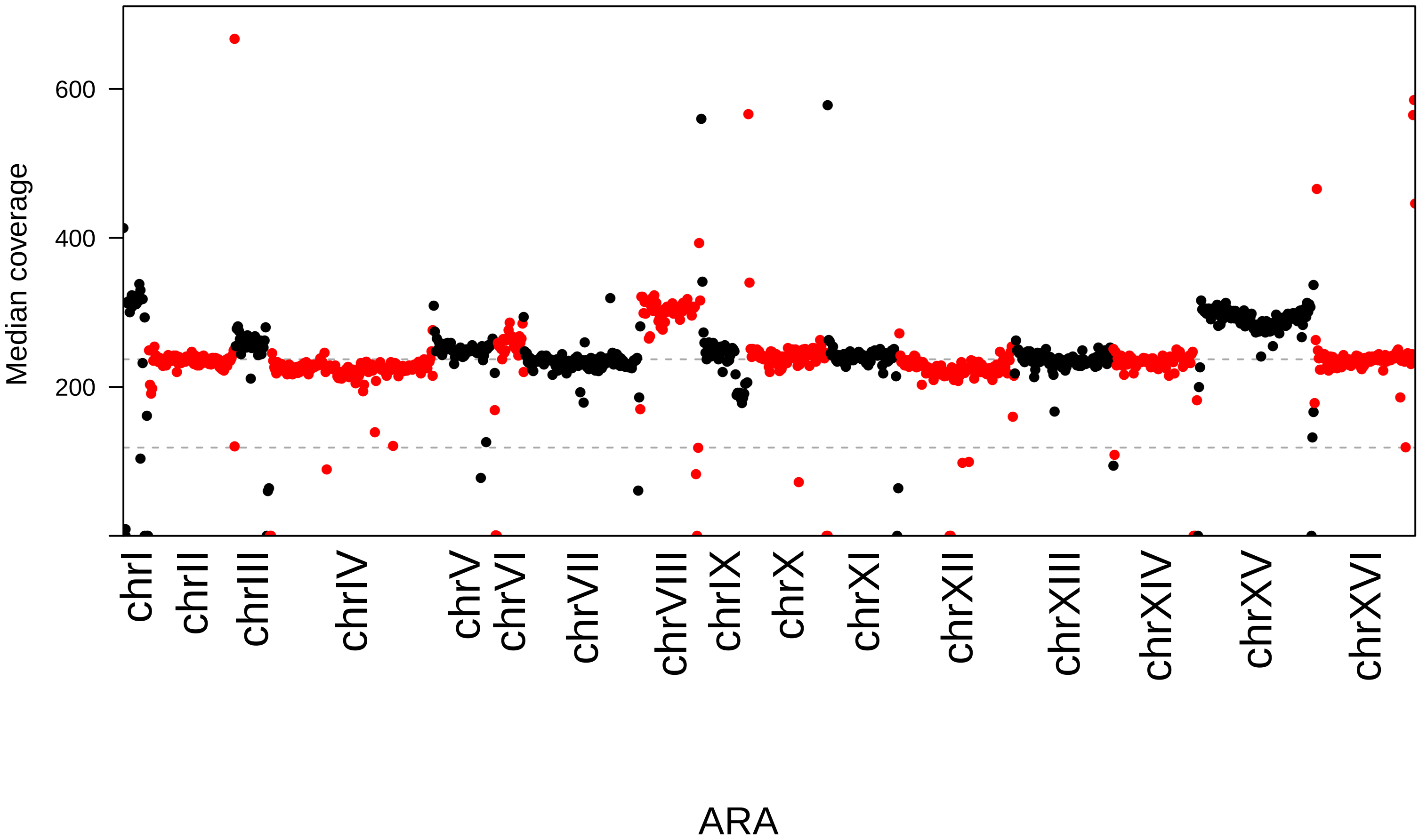

Supplementary Figure S2

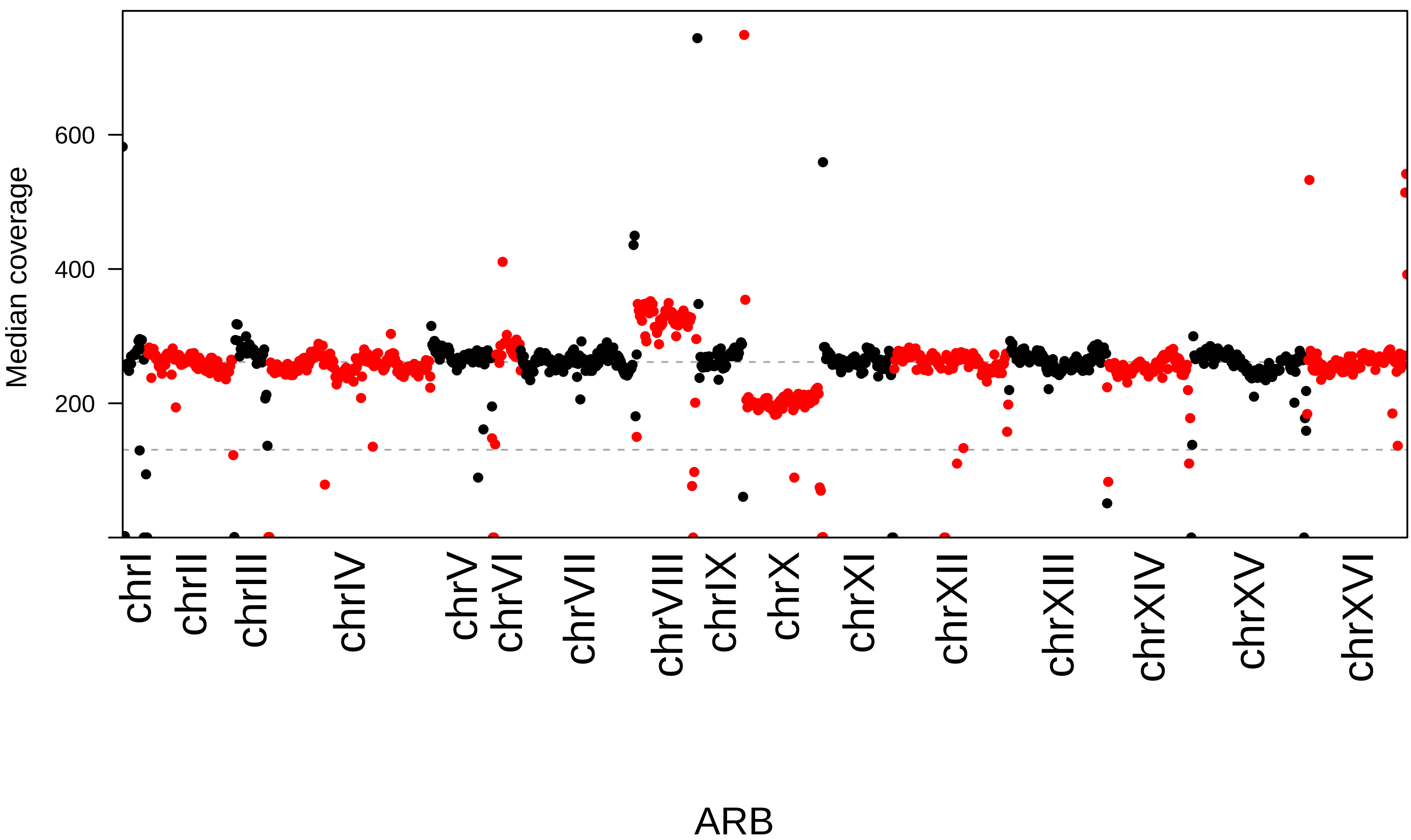

Supplementary Figure S2

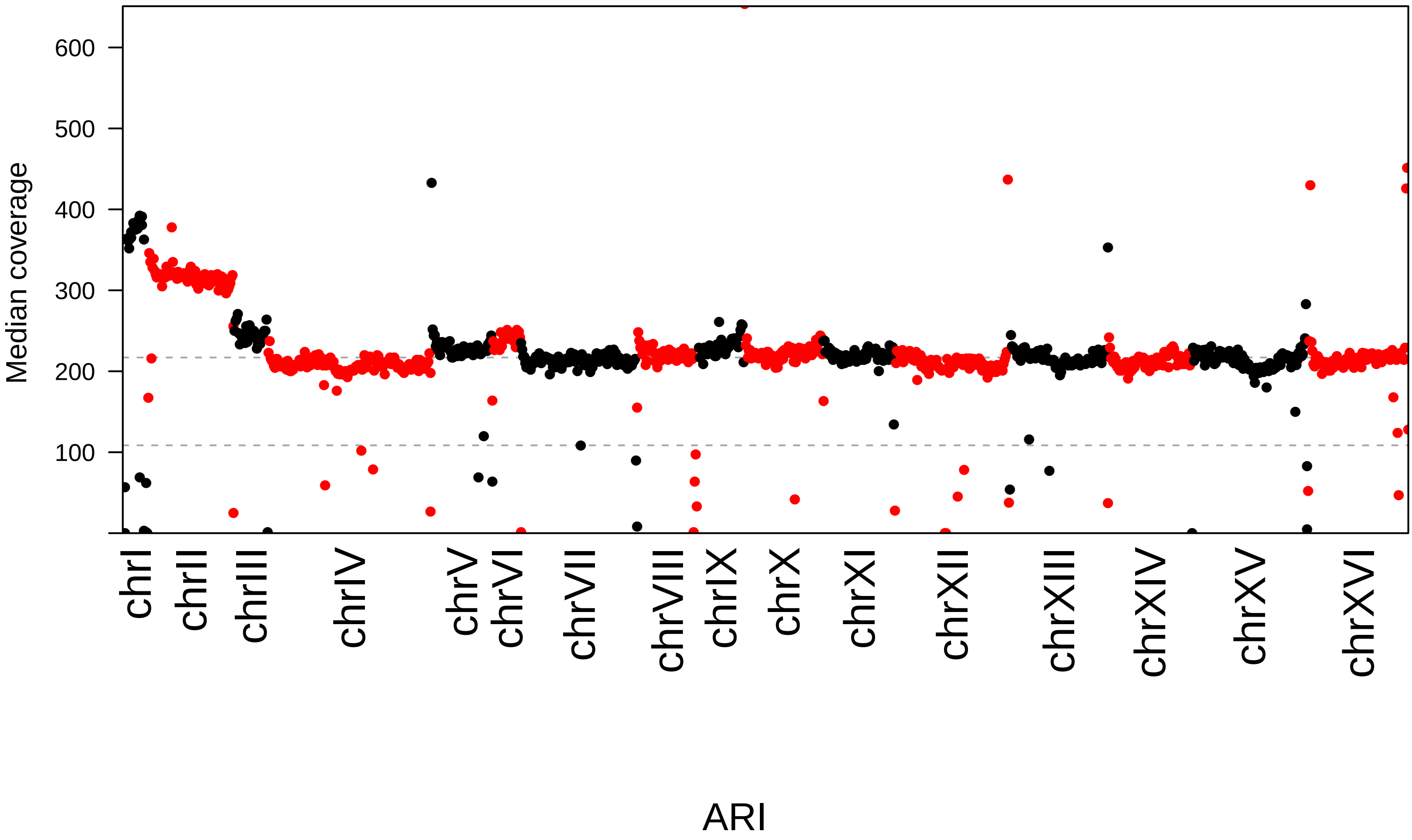

Supplementary Figure S2

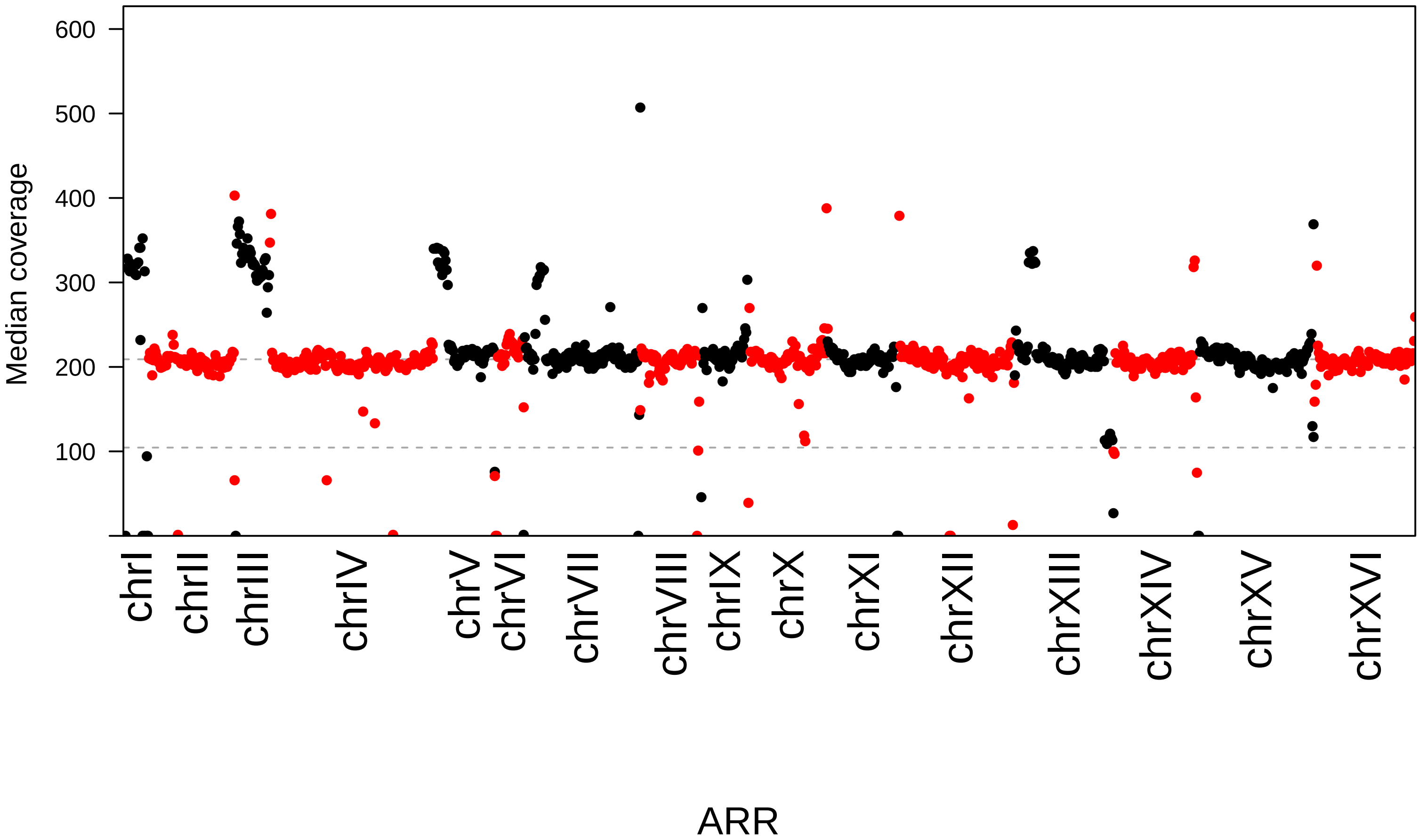

Supplementary Figure S2

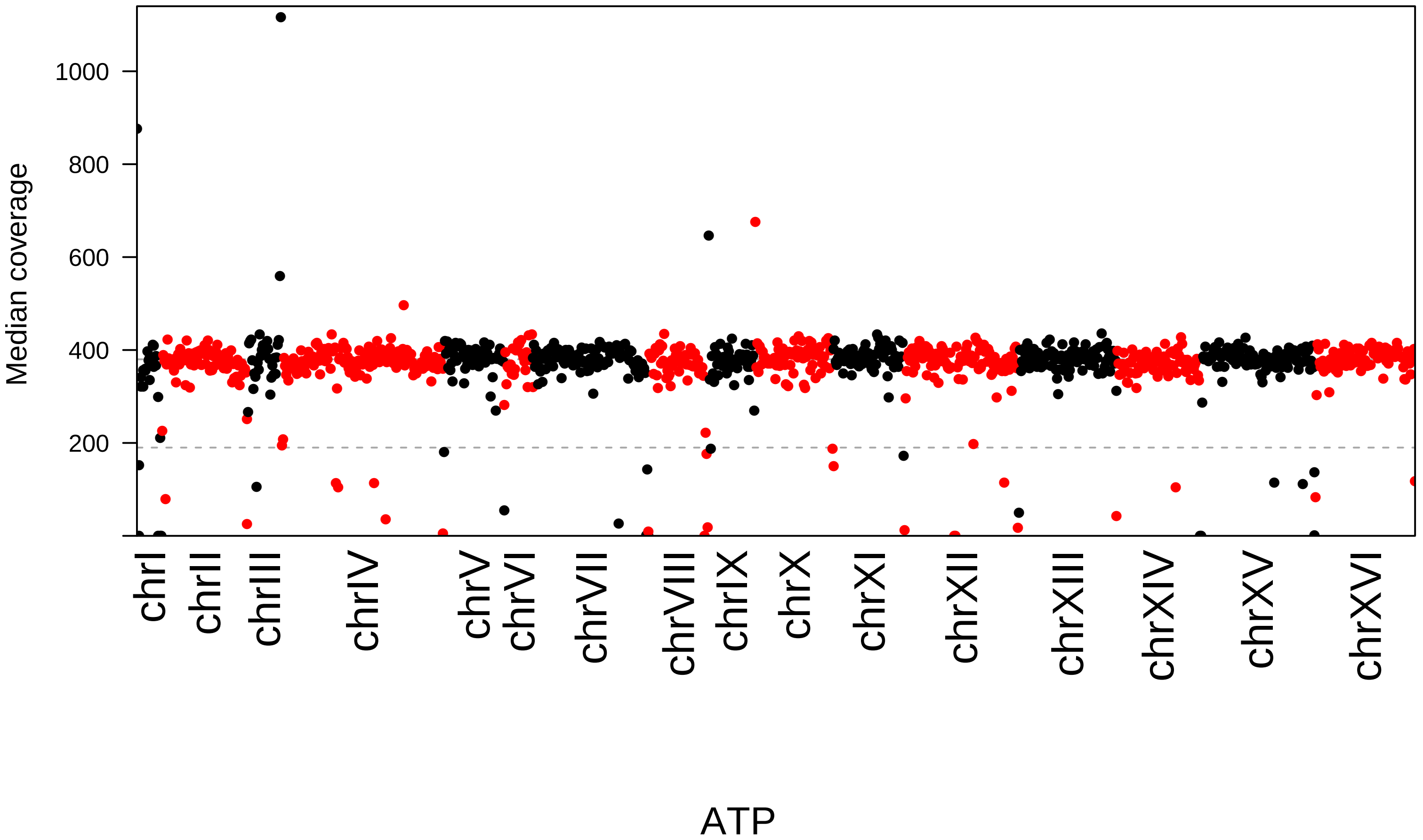

Supplementary Figure S2

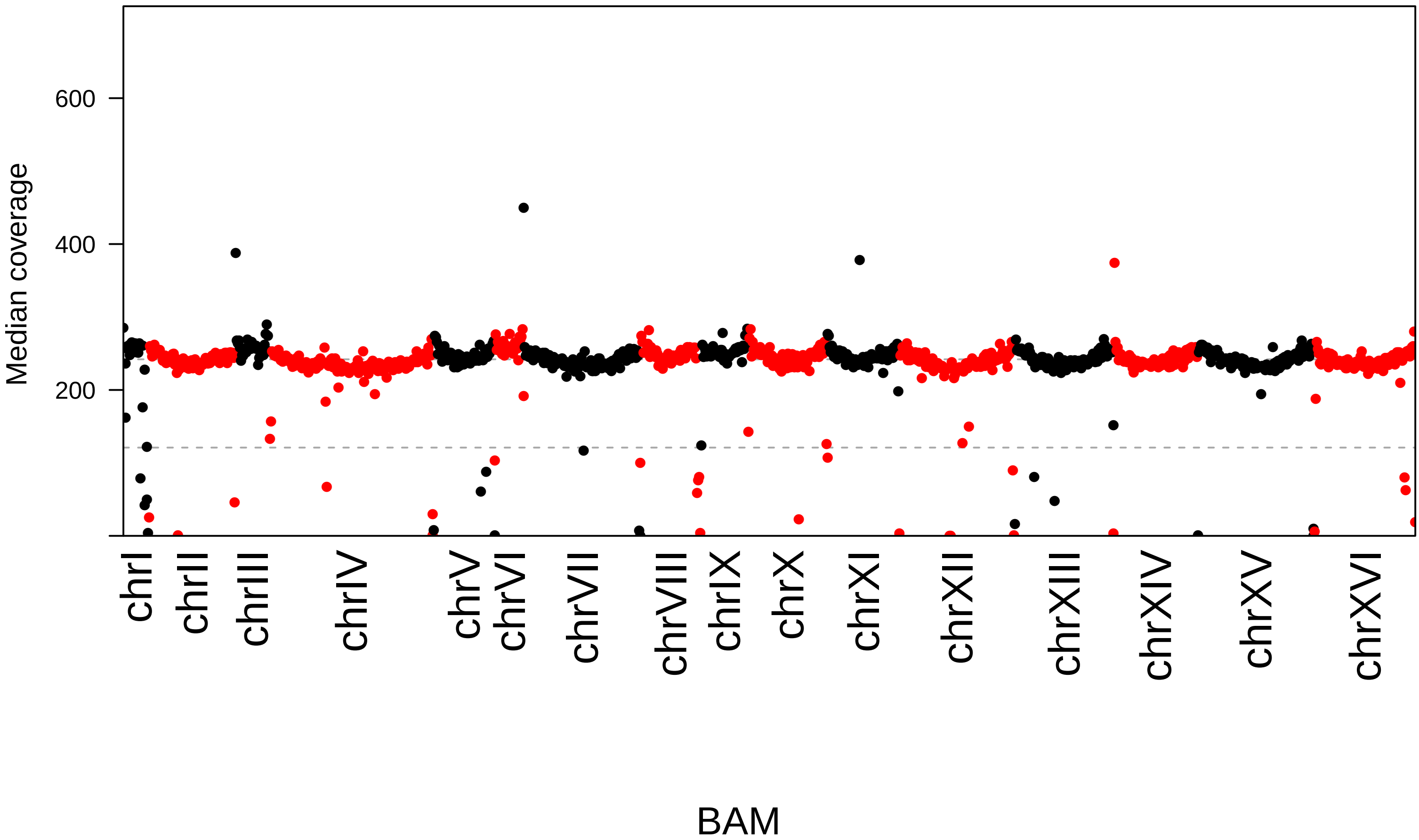

Supplementary Figure S2

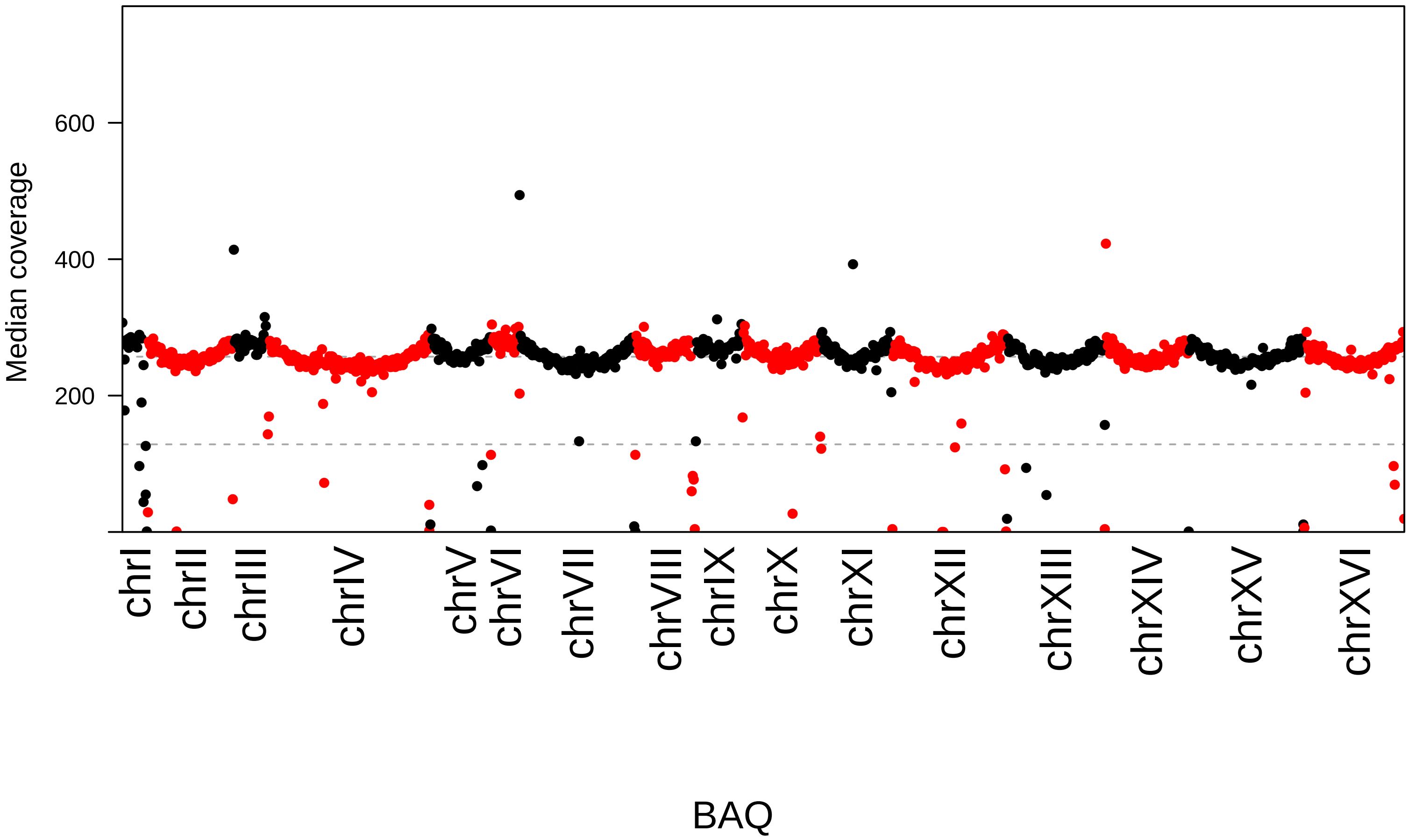

Supplementary Figure S2

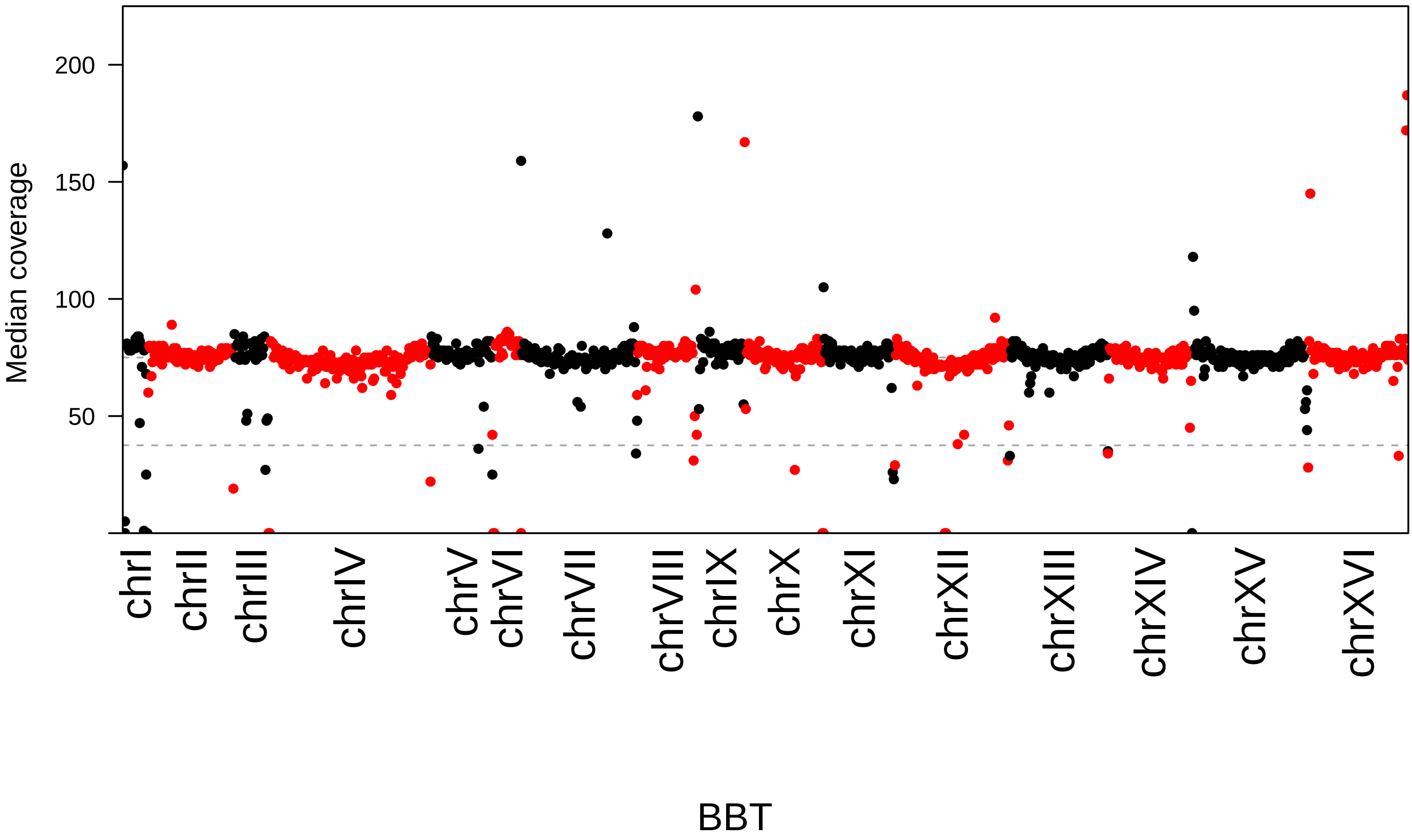

Supplementary Figure S2

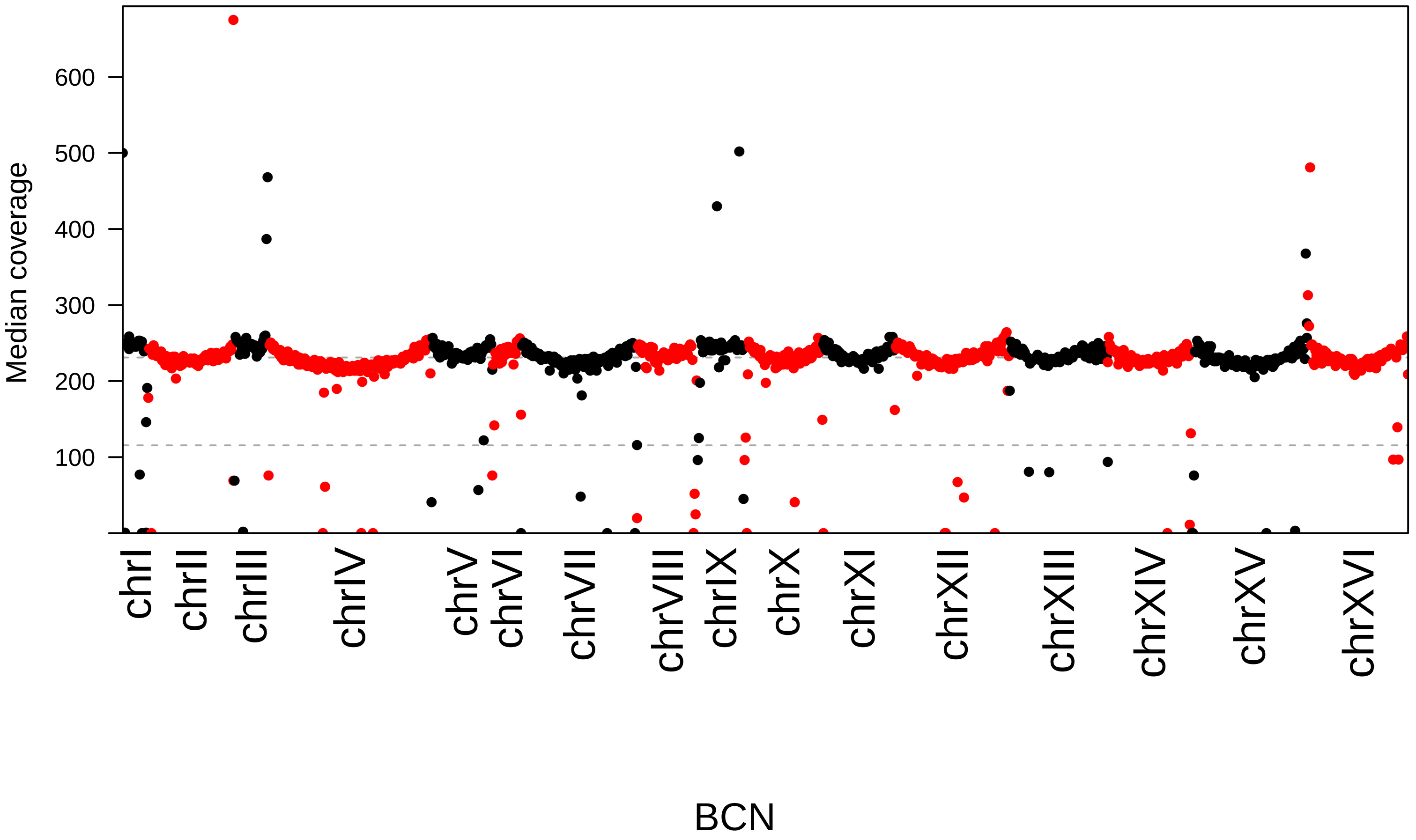

Supplementary Figure S2

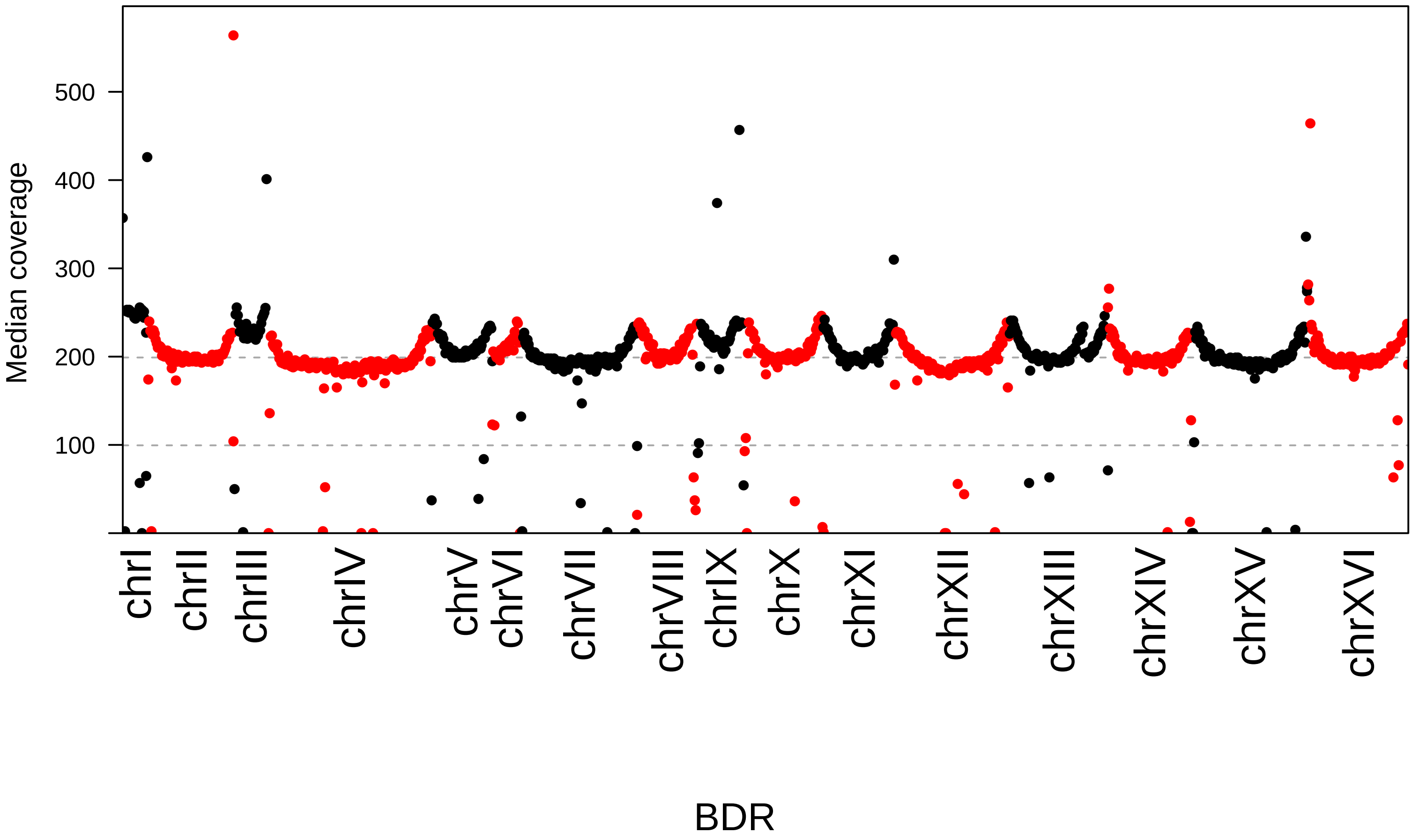

Supplementary Figure S2

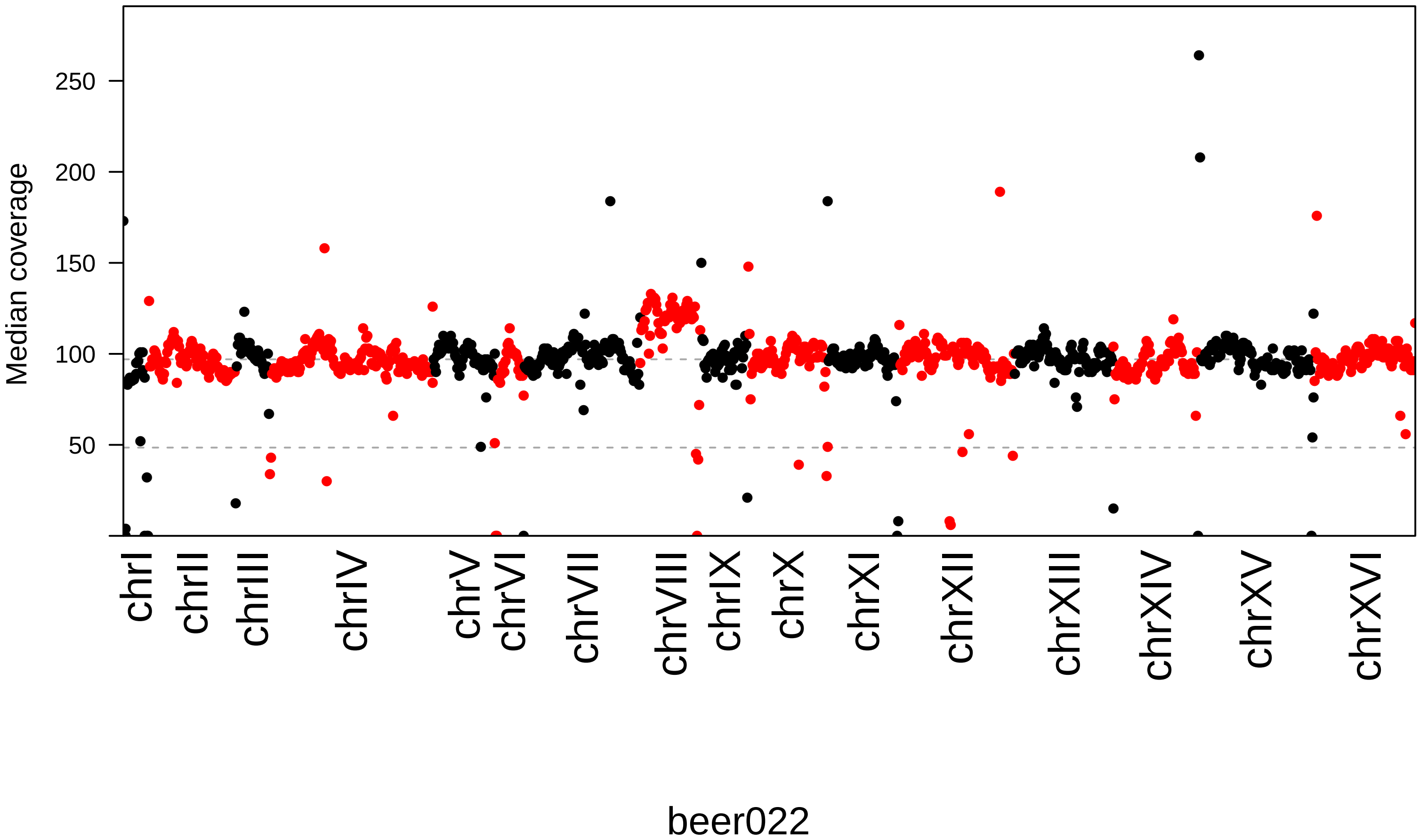

Supplementary Figure S2

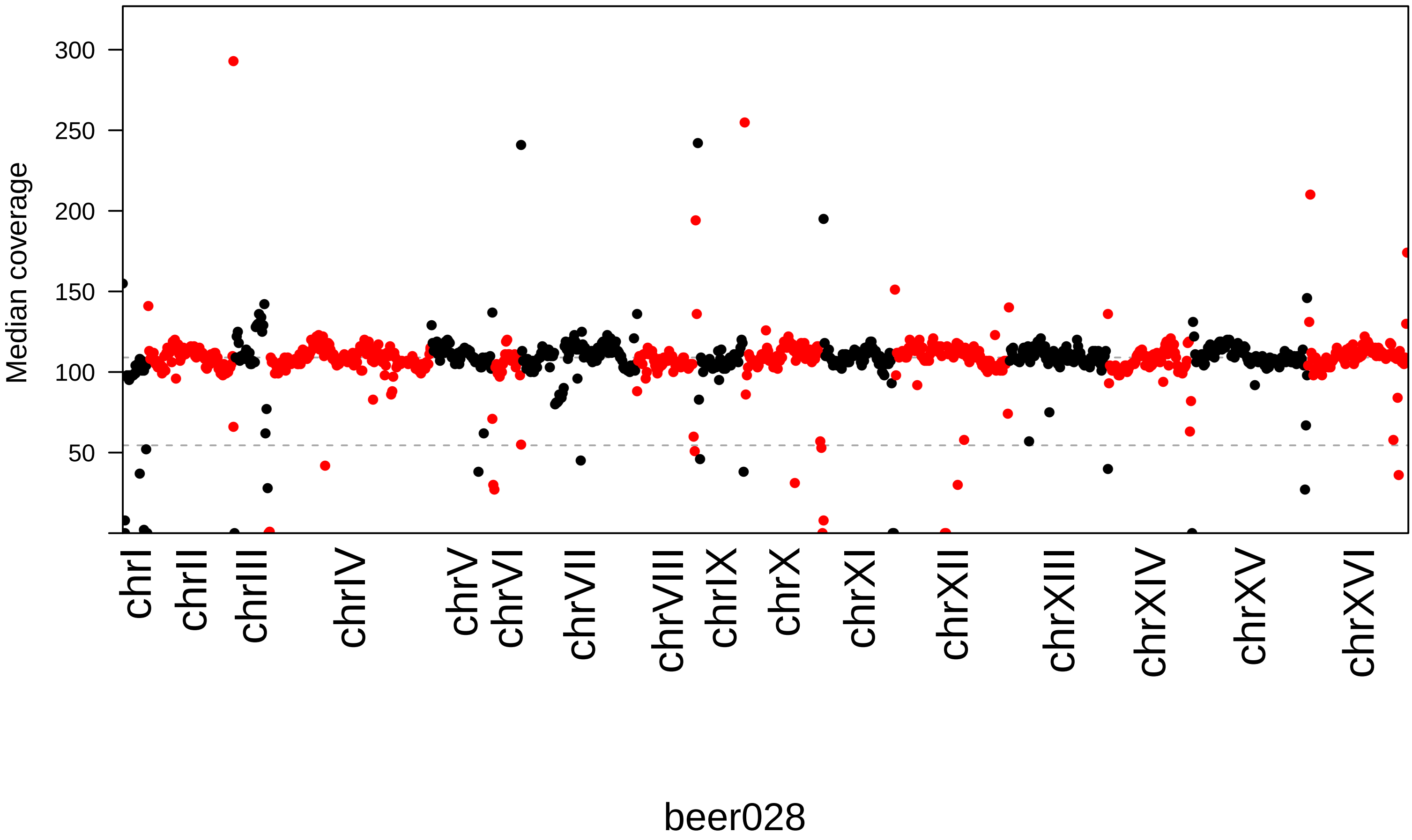

Supplementary Figure S2

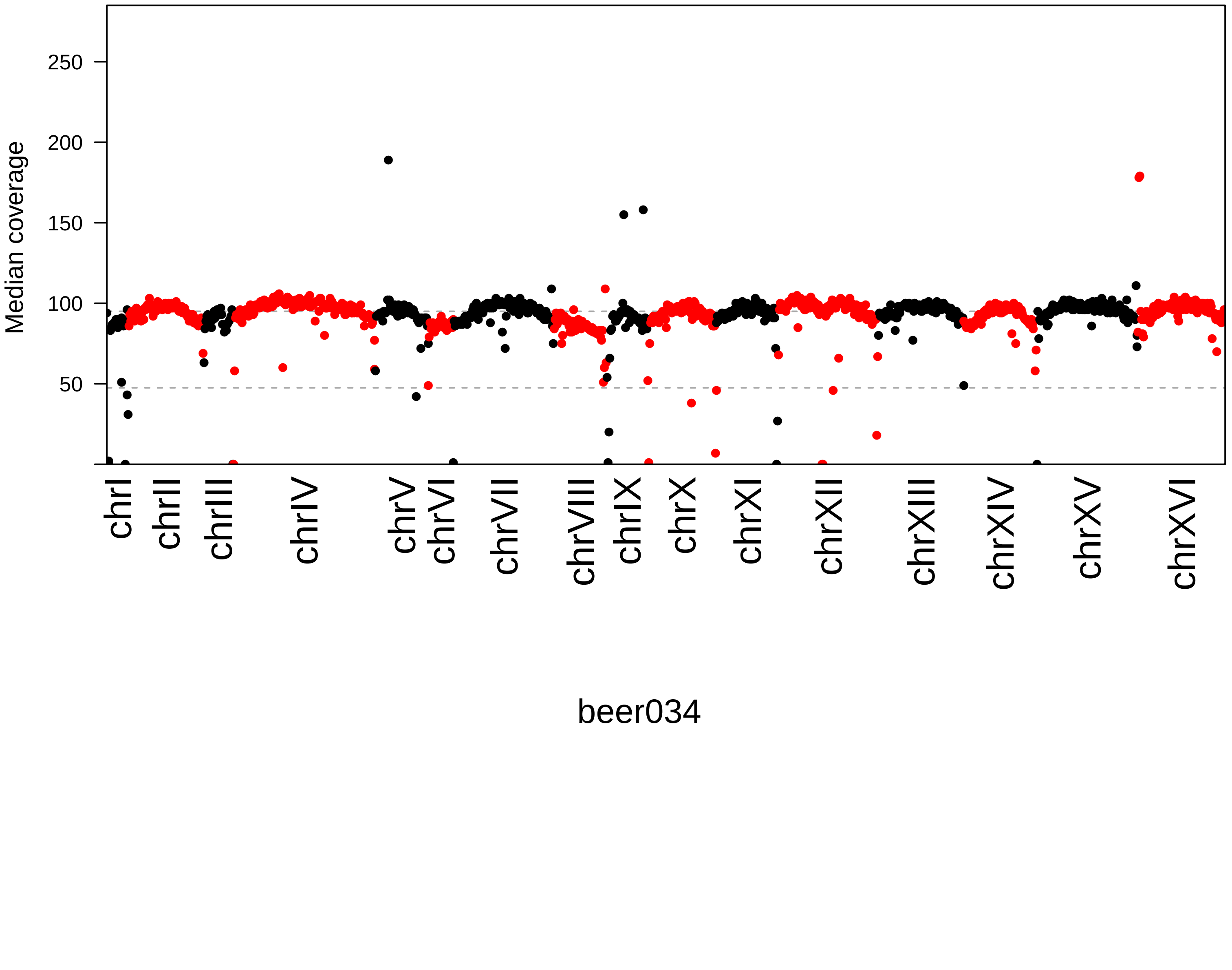

Supplementary Figure S2

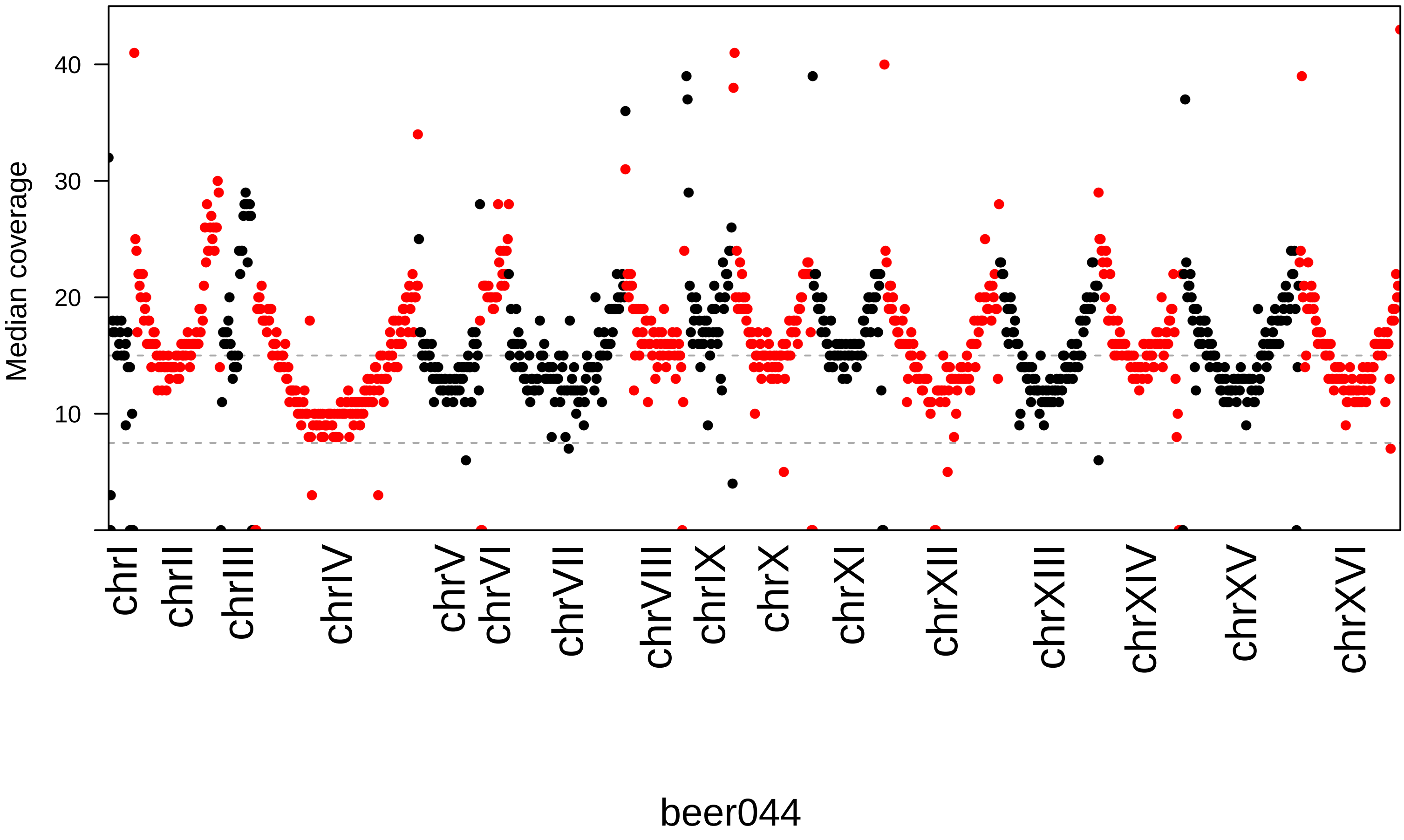

Supplementary Figure S2

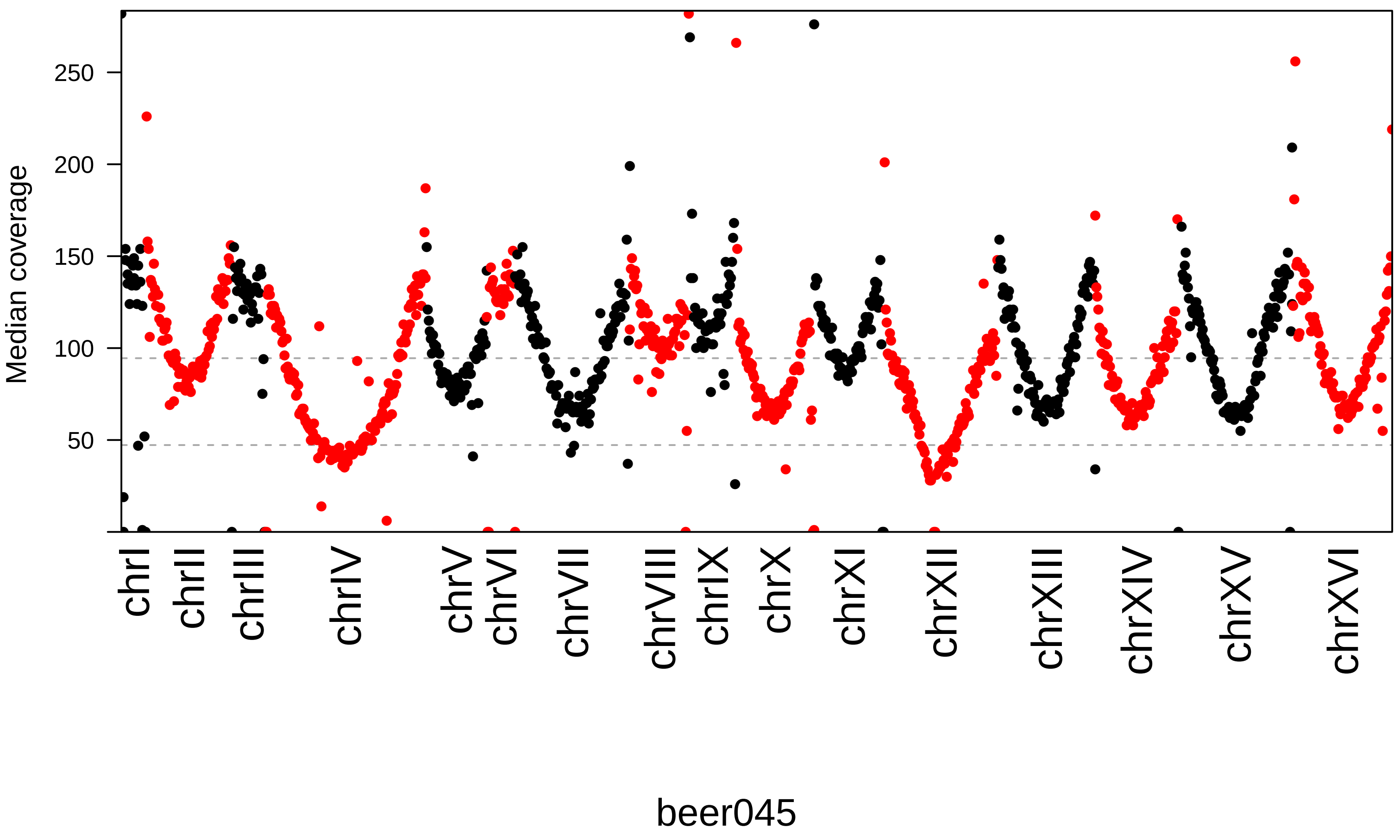

Supplementary Figure S2

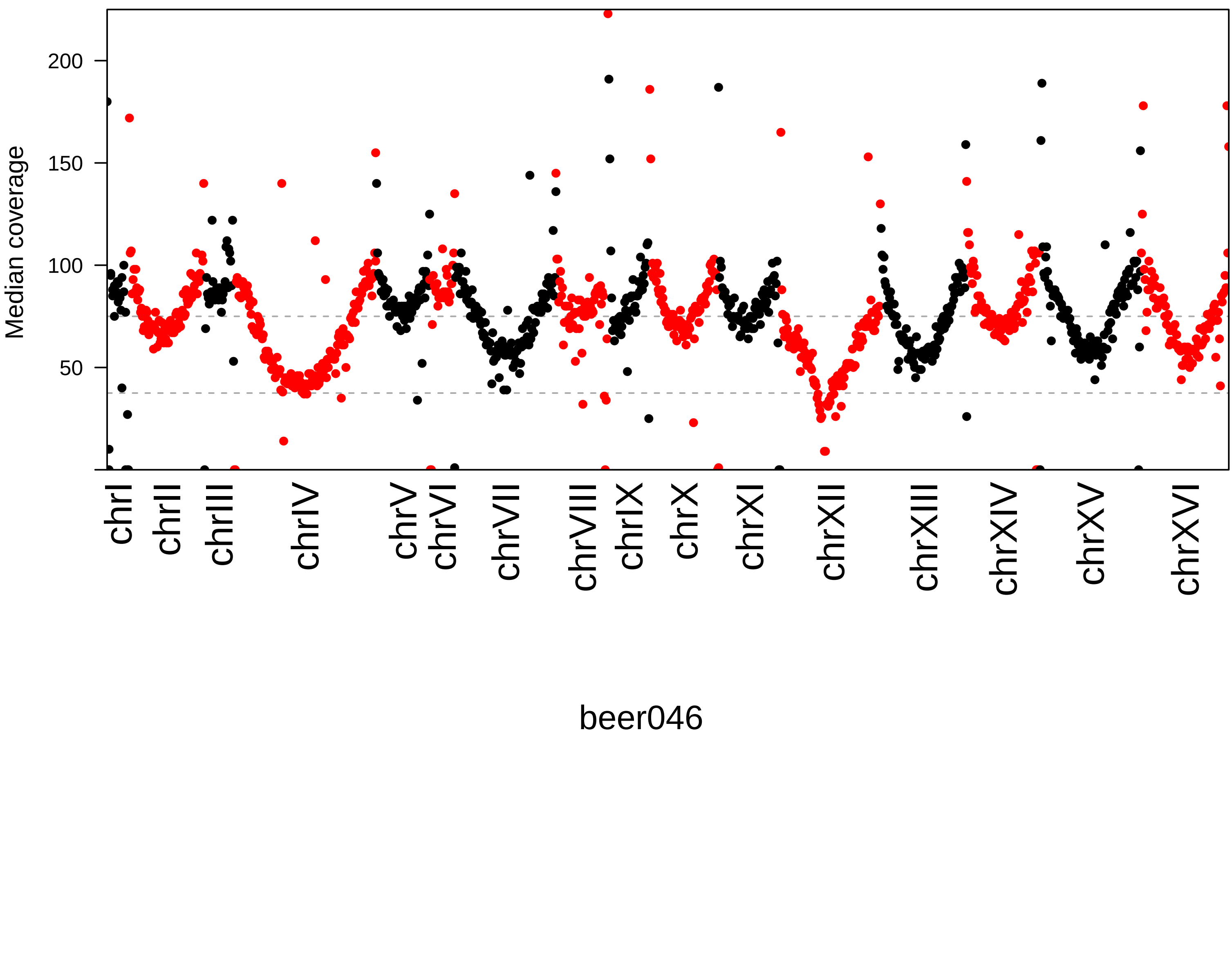

Supplementary Figure S2

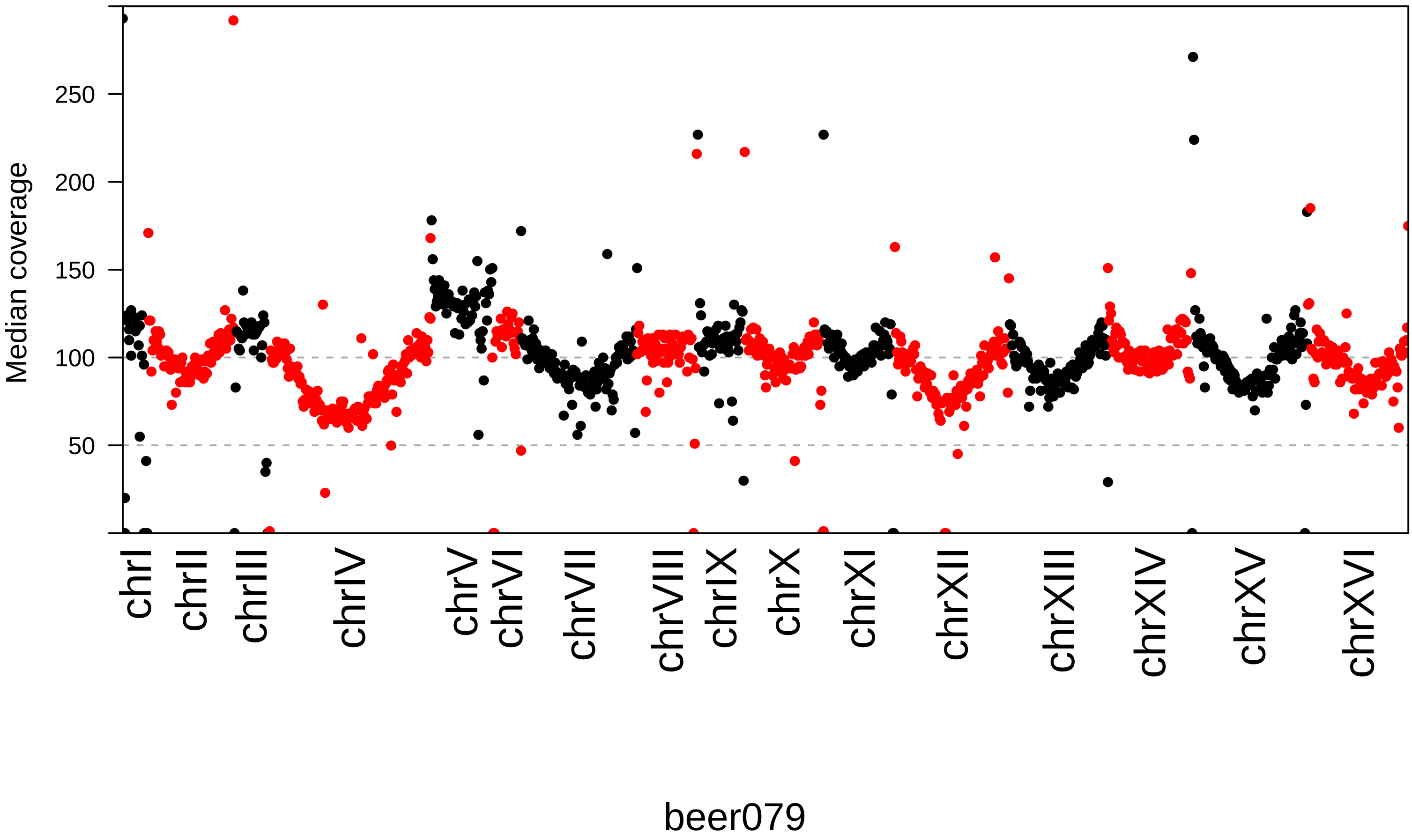

Supplementary Figure S2

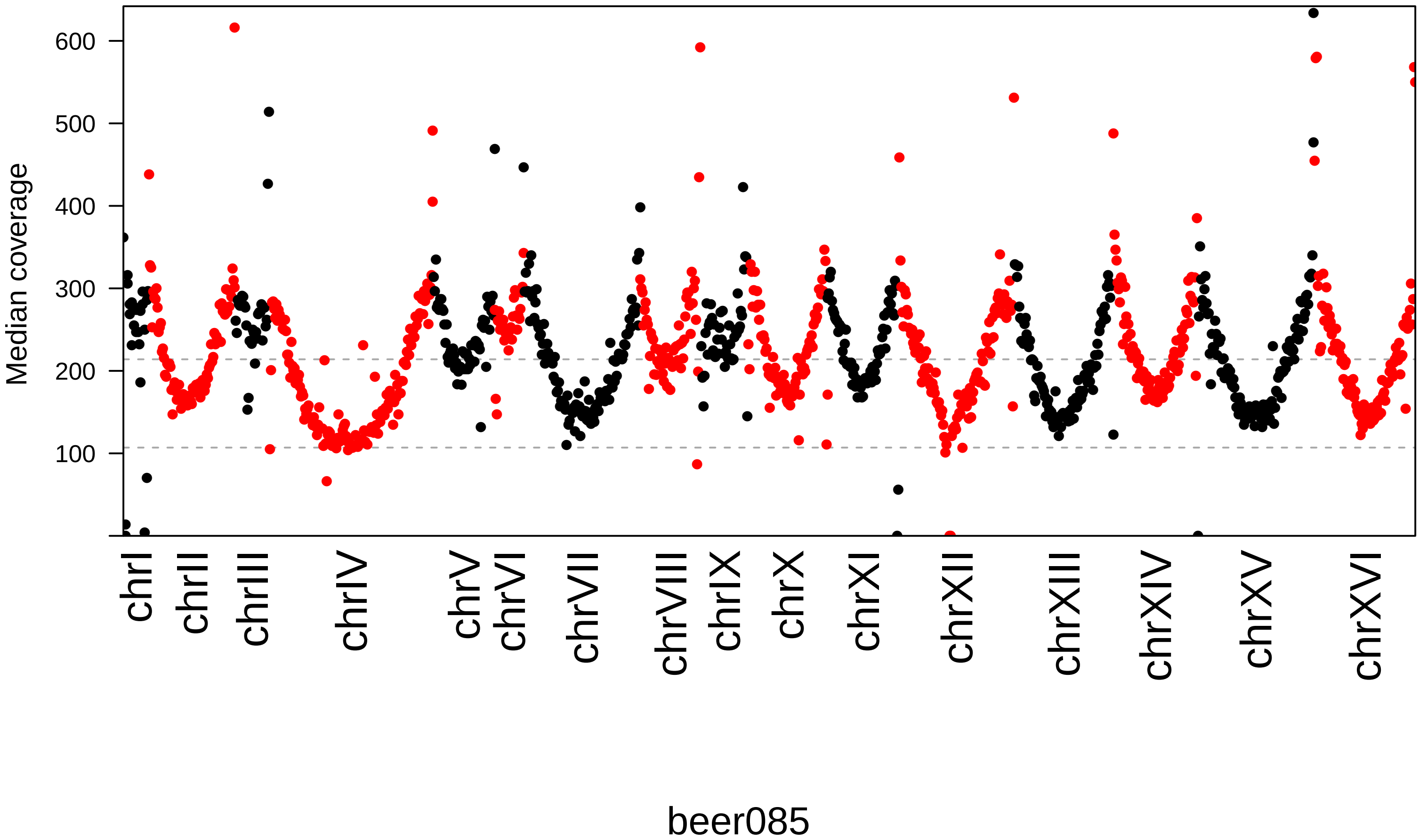

Supplementary Figure S2

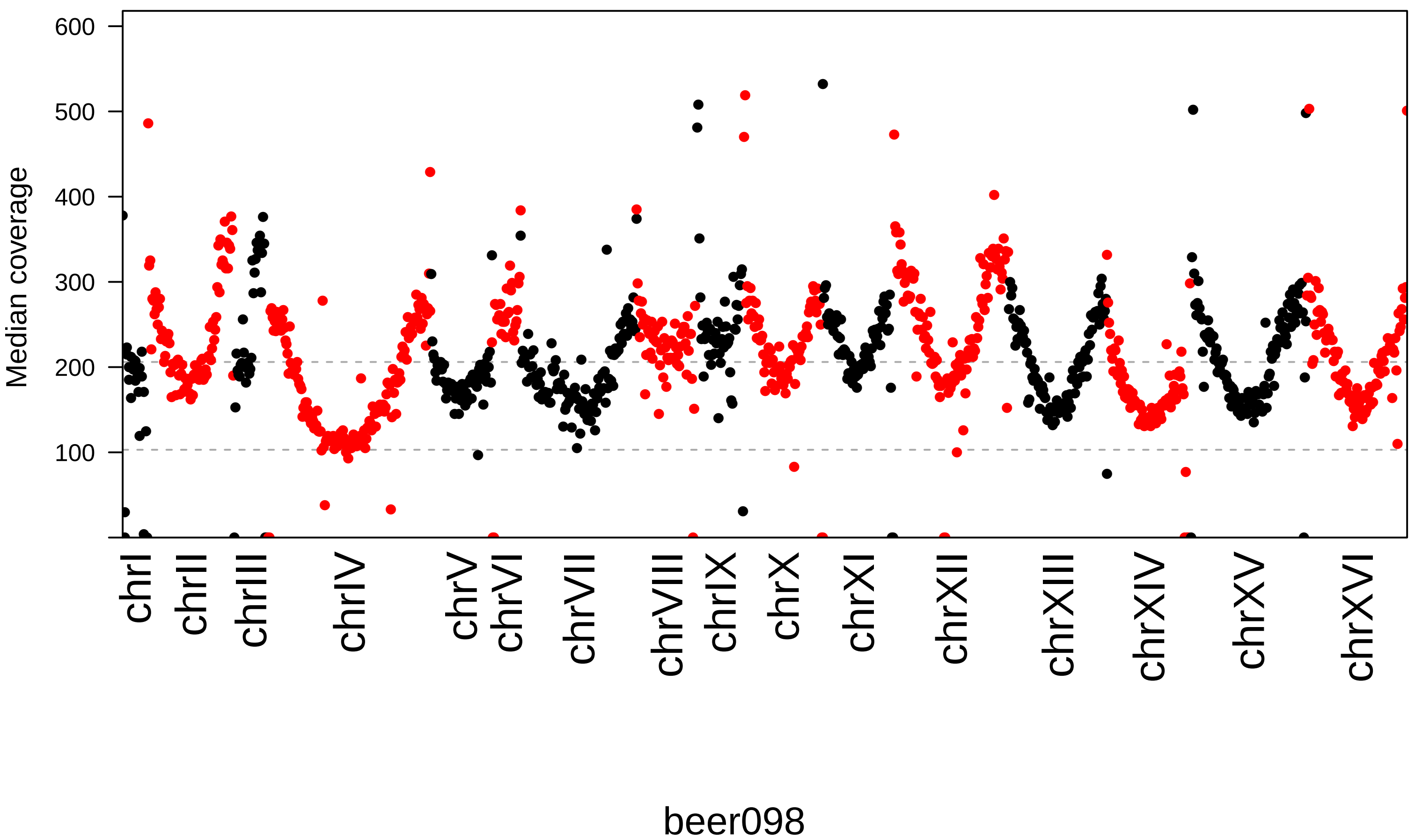

Supplementary Figure S2

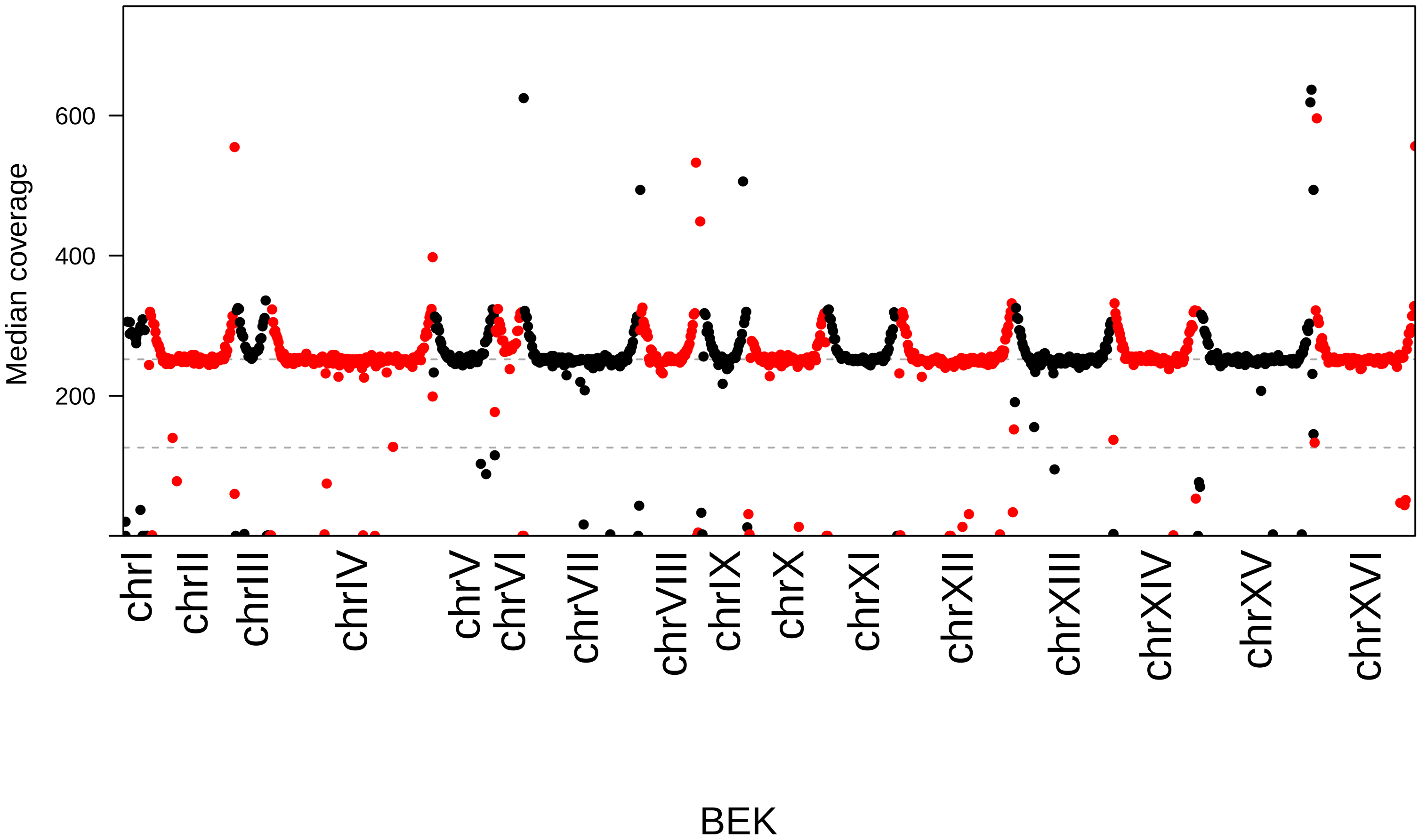

Supplementary Figure S2

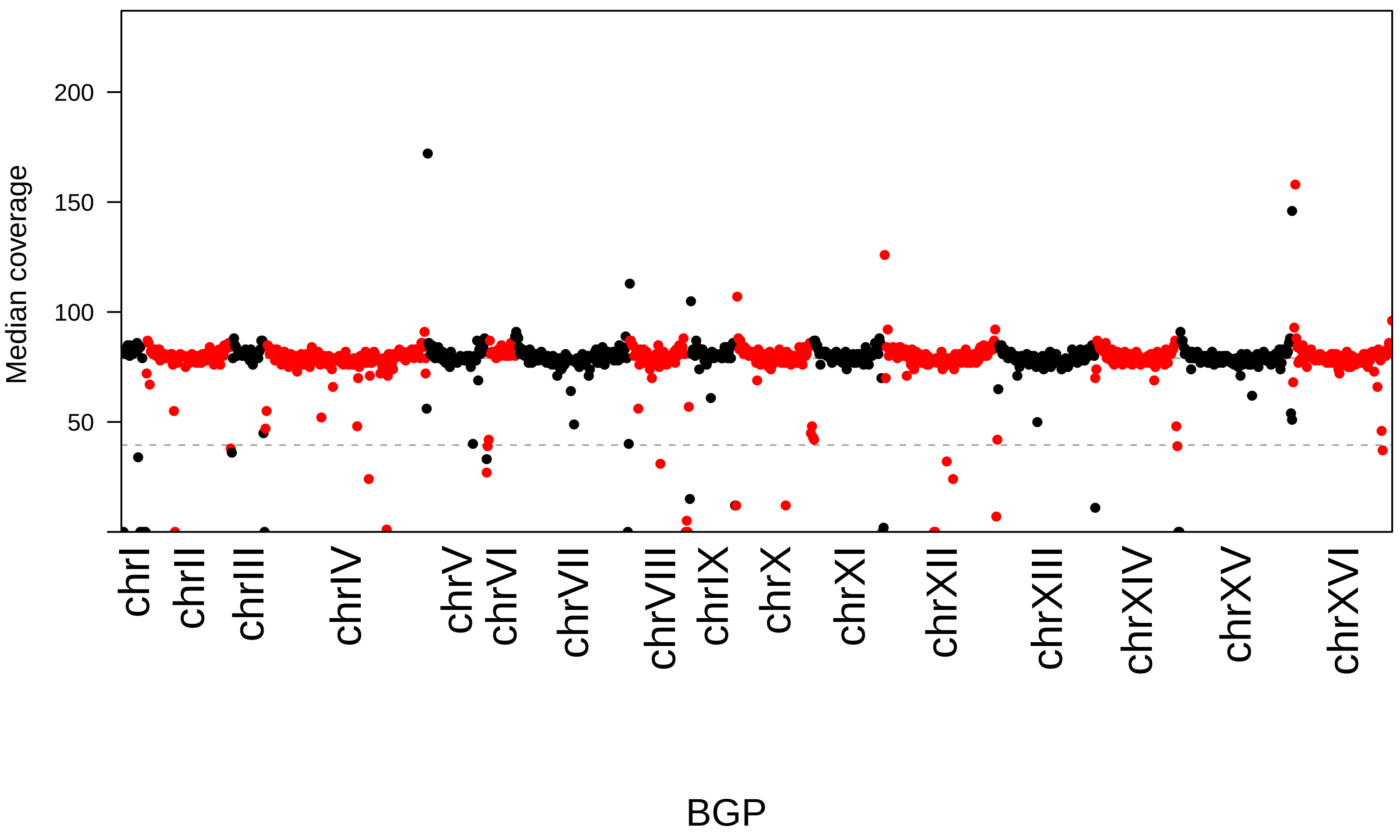

Supplementary Figure S2

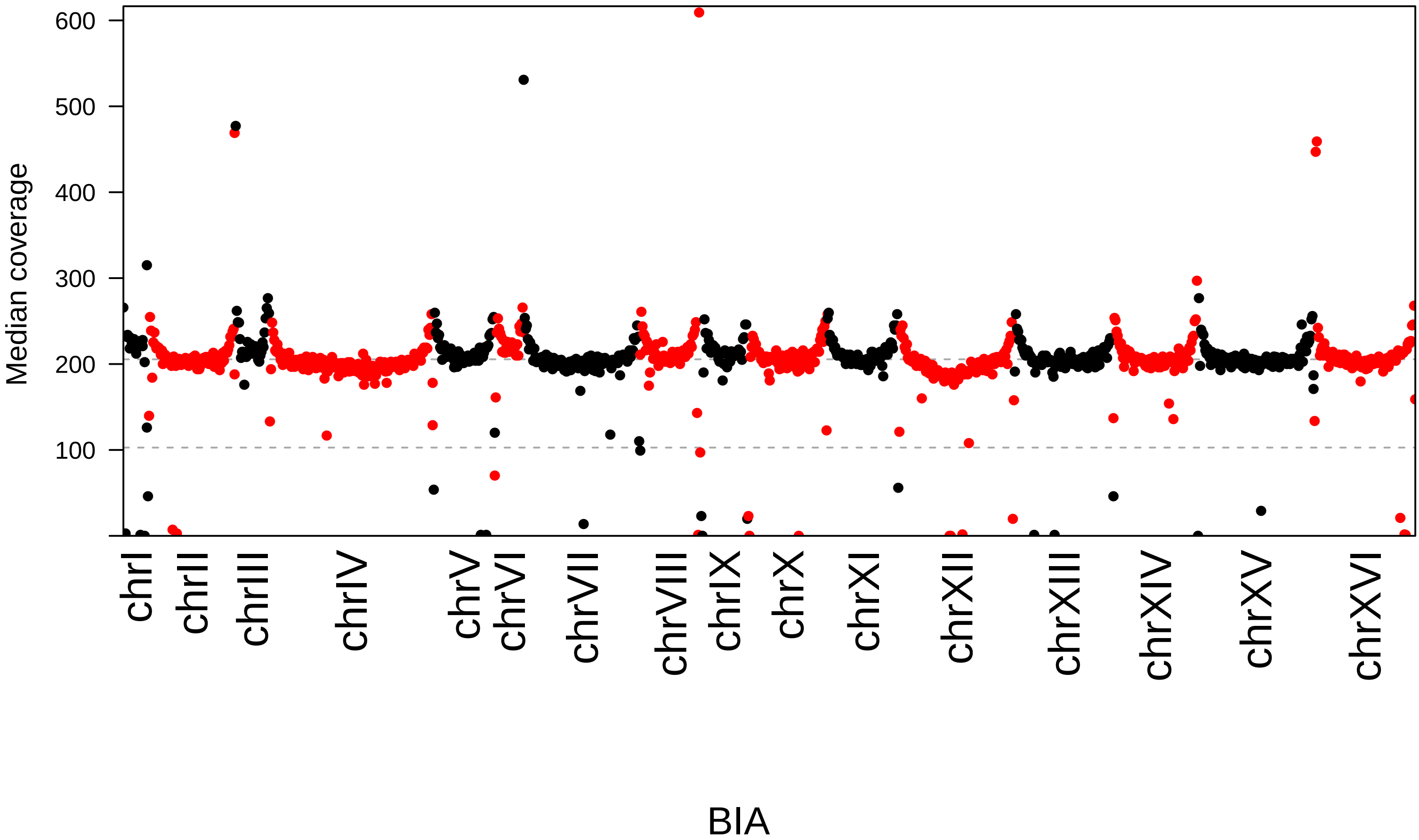

Supplementary Figure S2

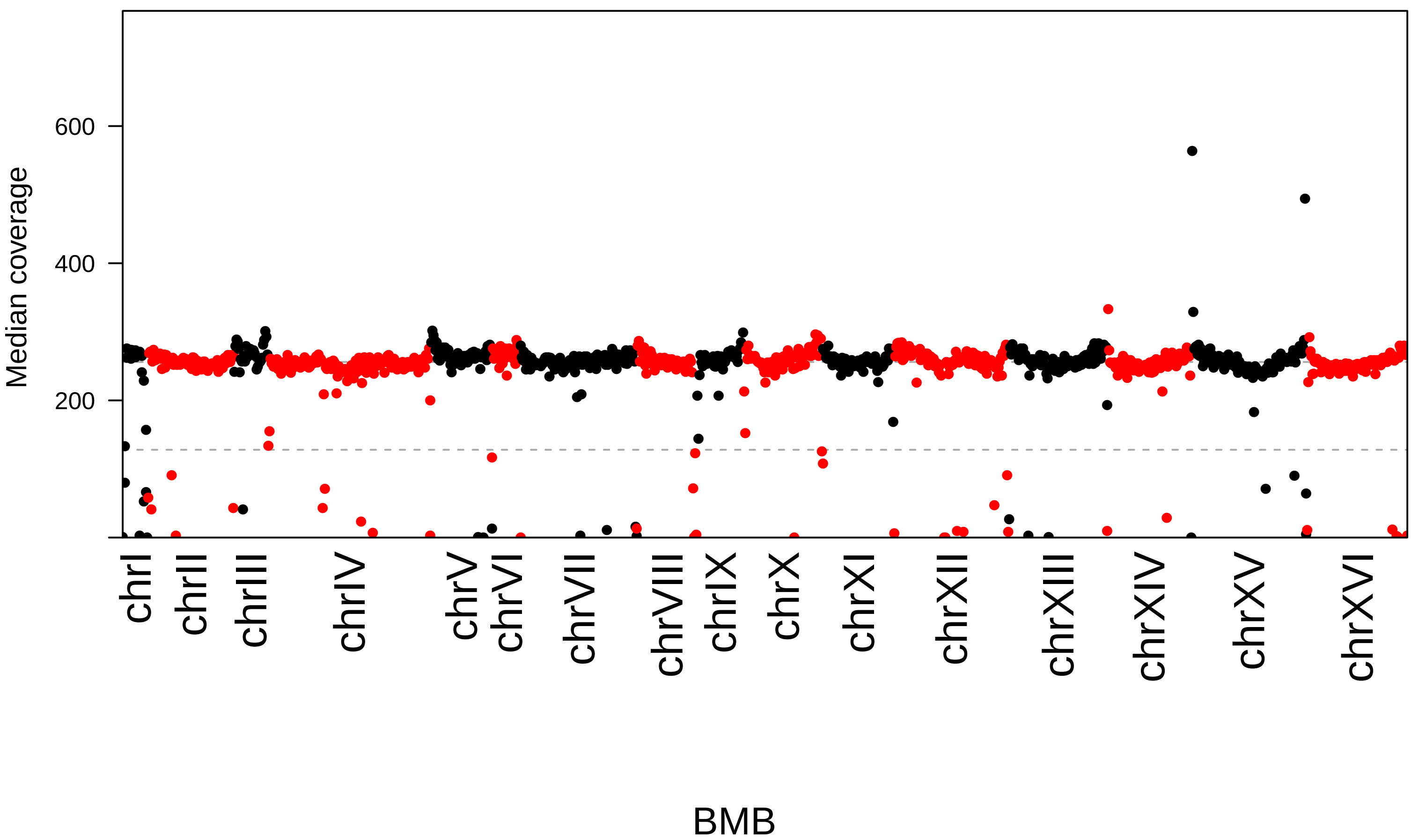

Supplementary Figure S2

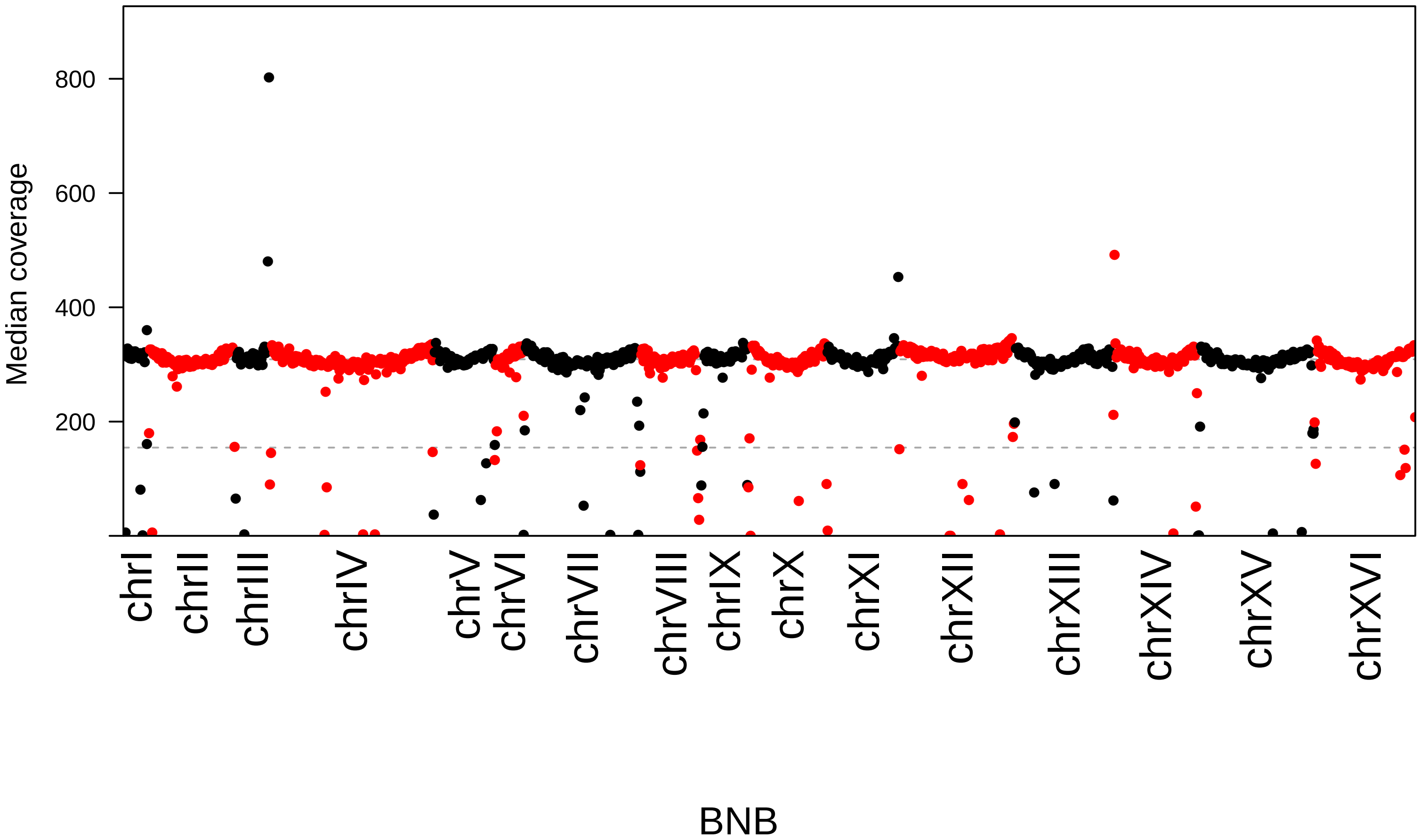

Supplementary Figure S2

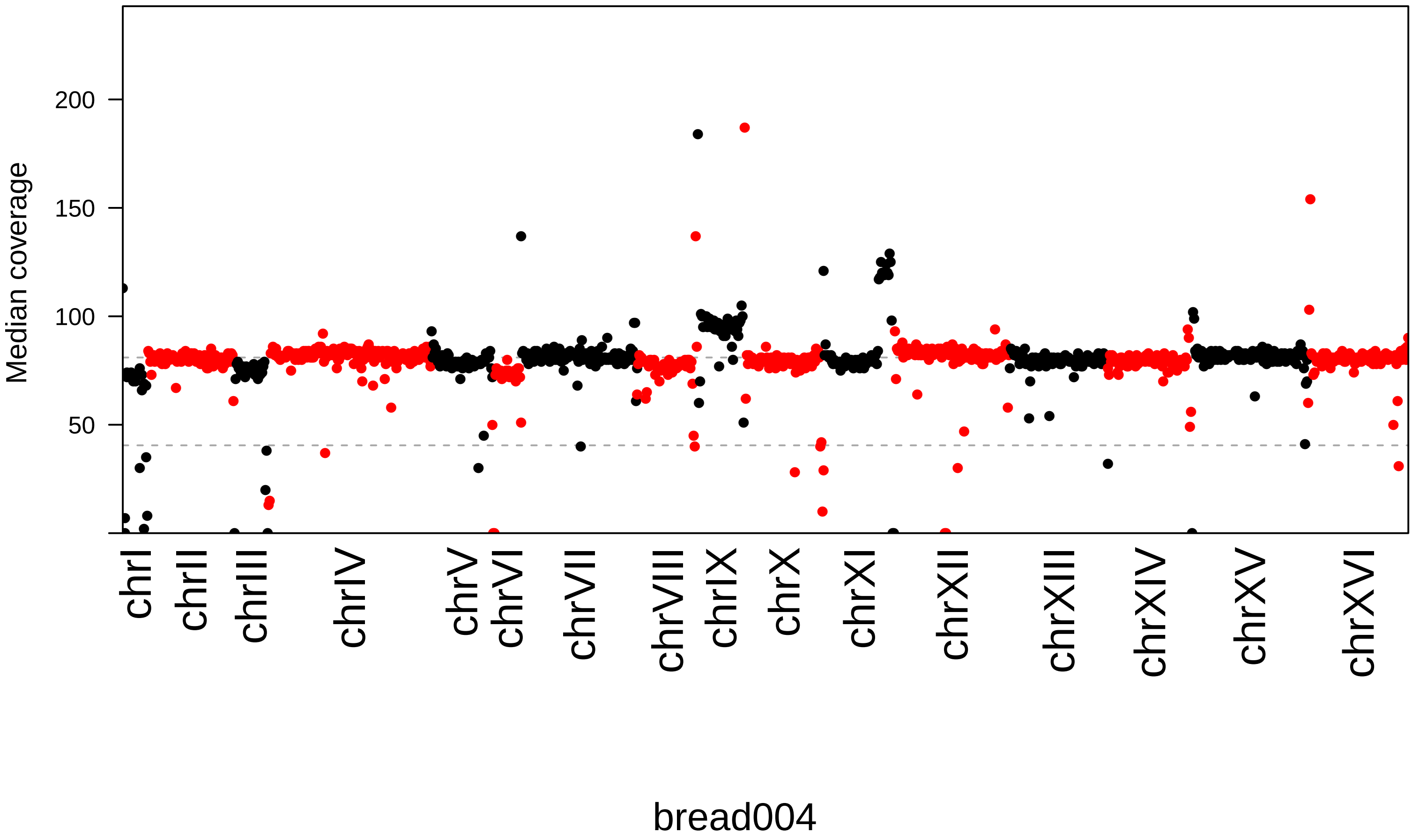

Supplementary Figure S2

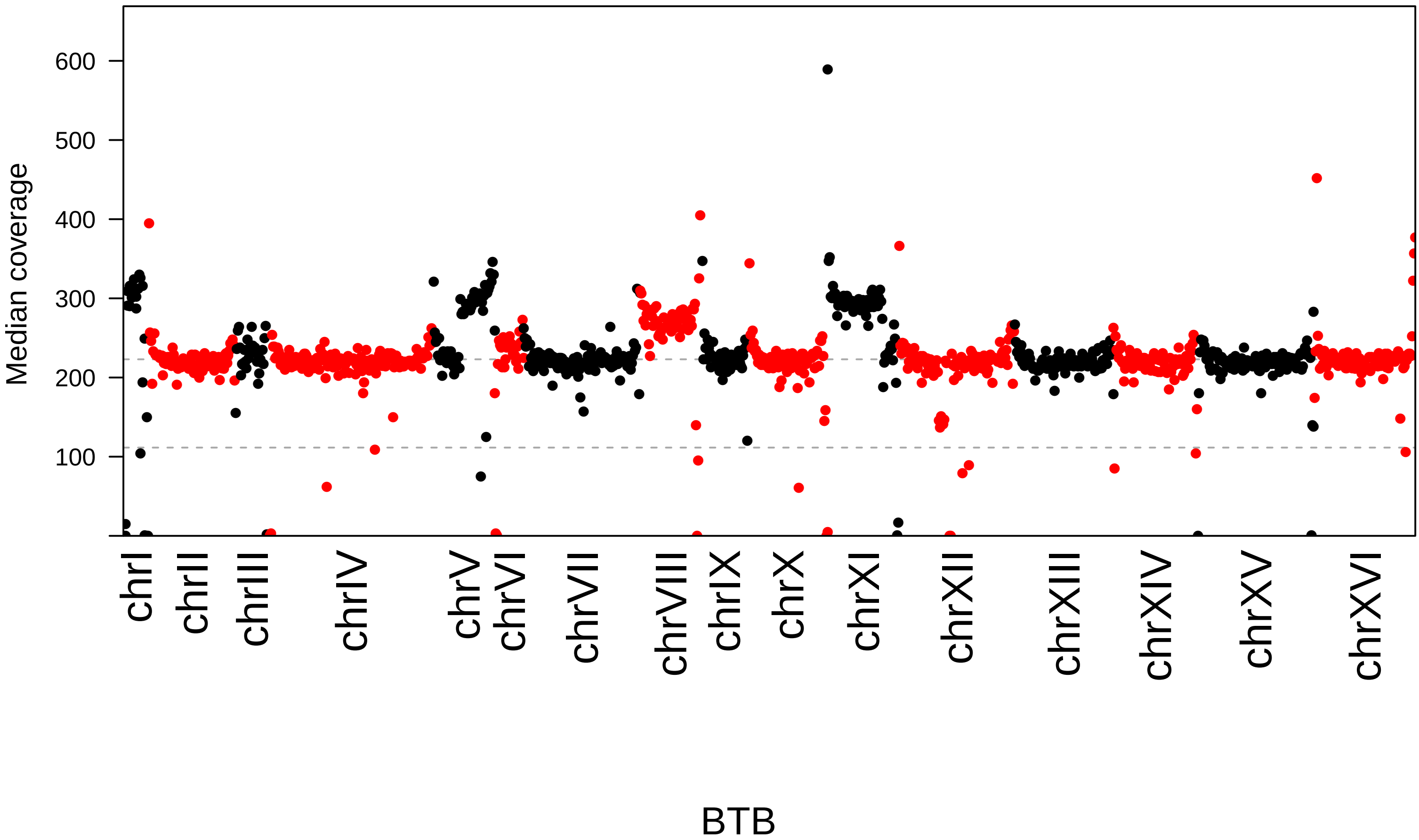

Supplementary Figure S2

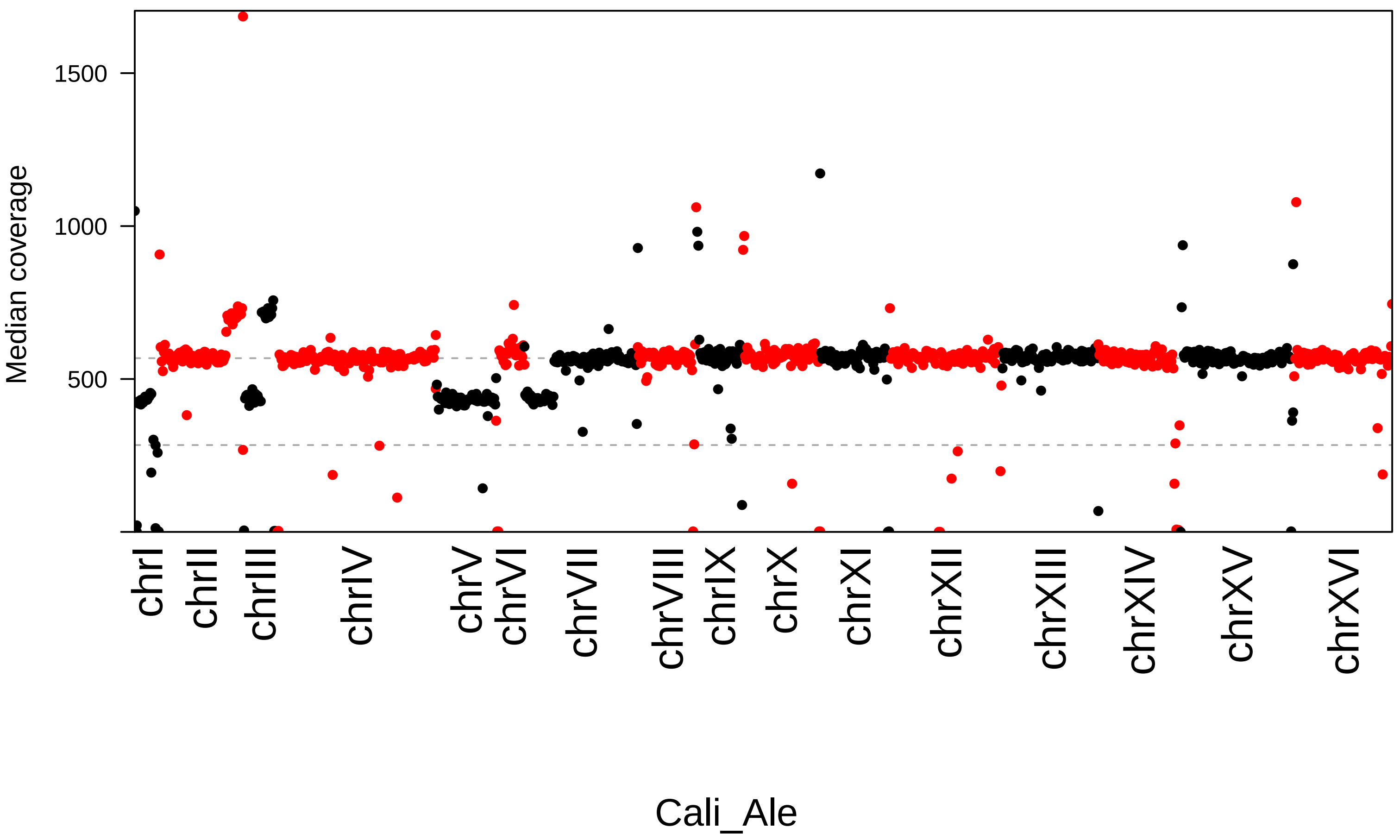

Supplementary Figure S2

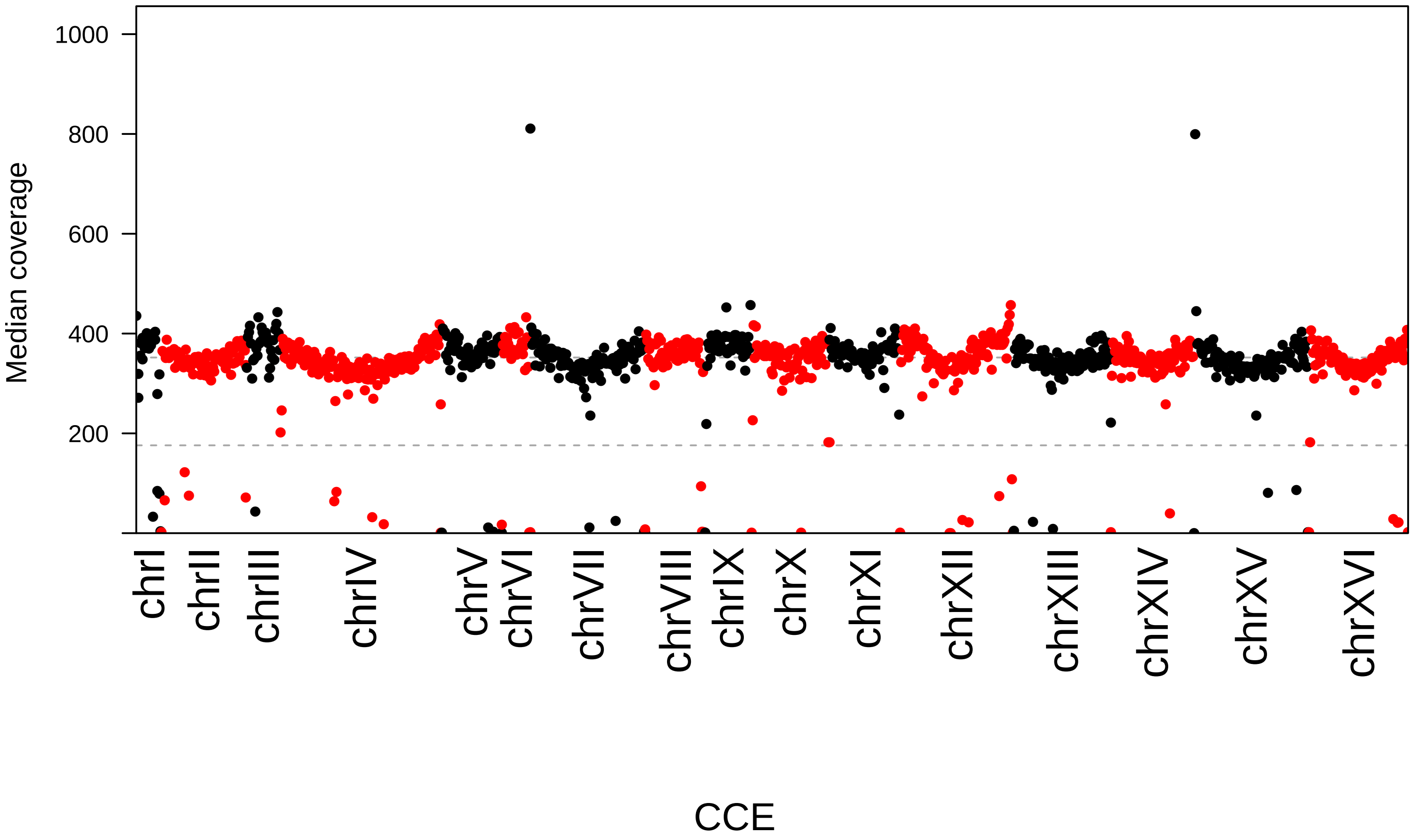

Supplementary Figure S2

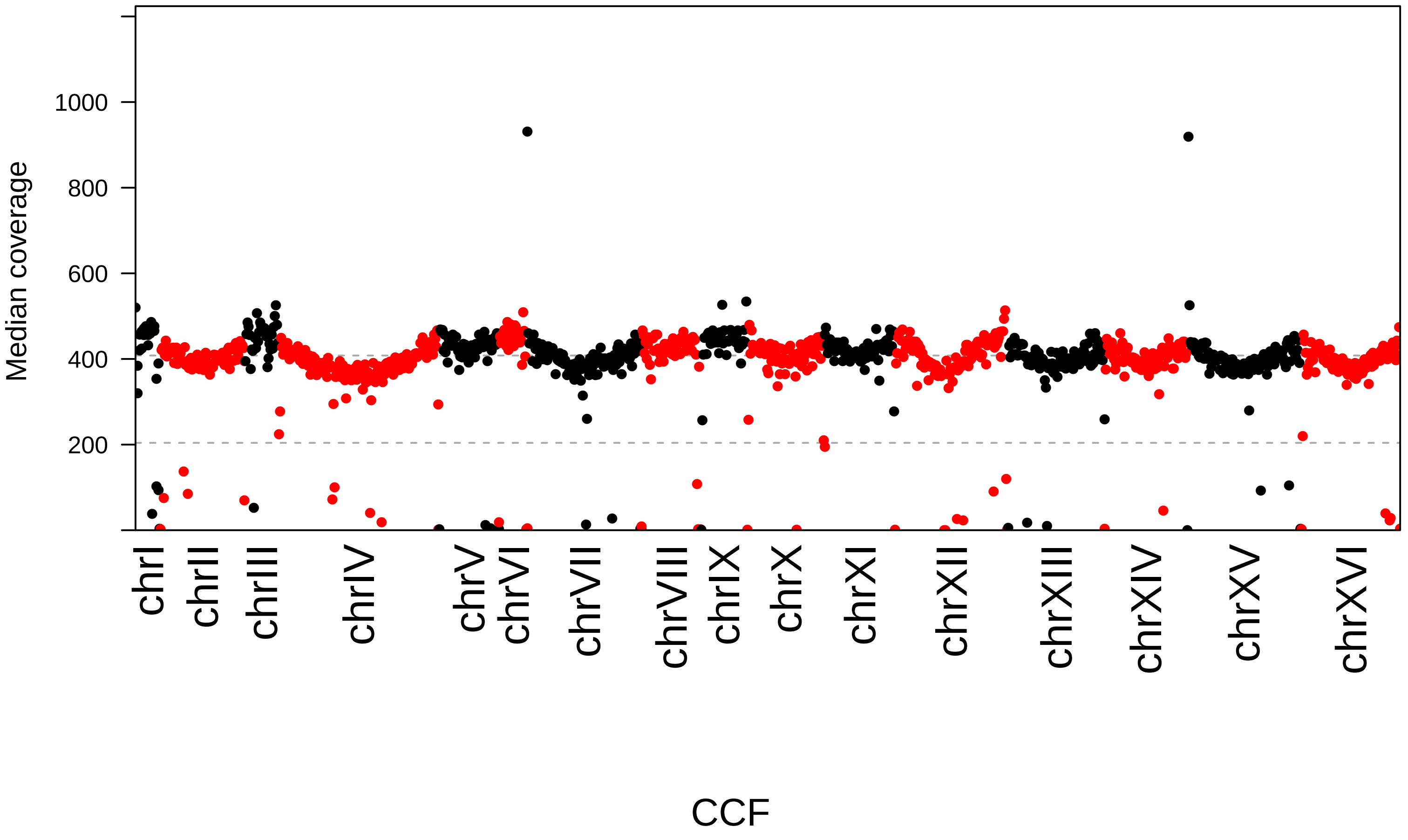

Supplementary Figure S2

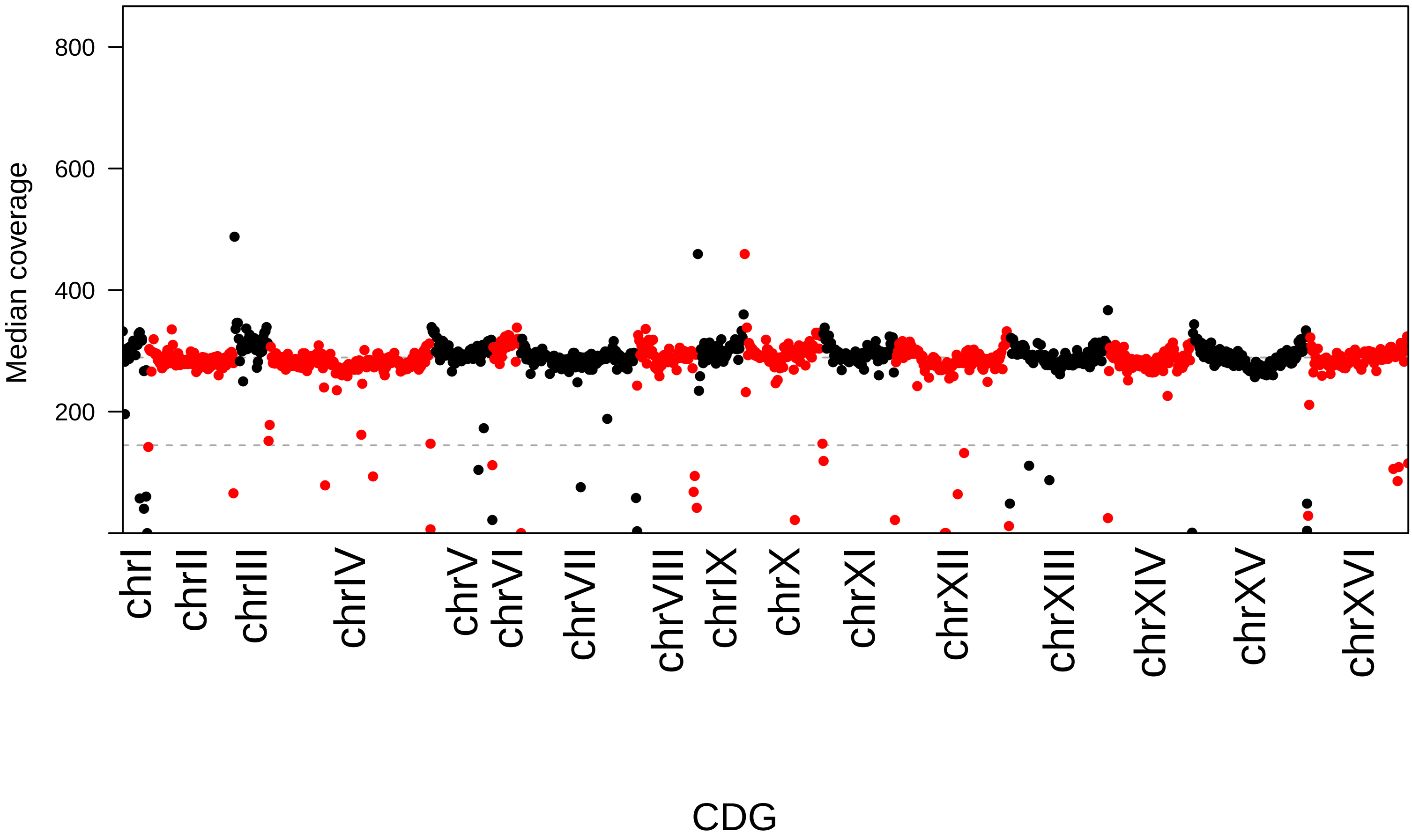

Supplementary Figure S2

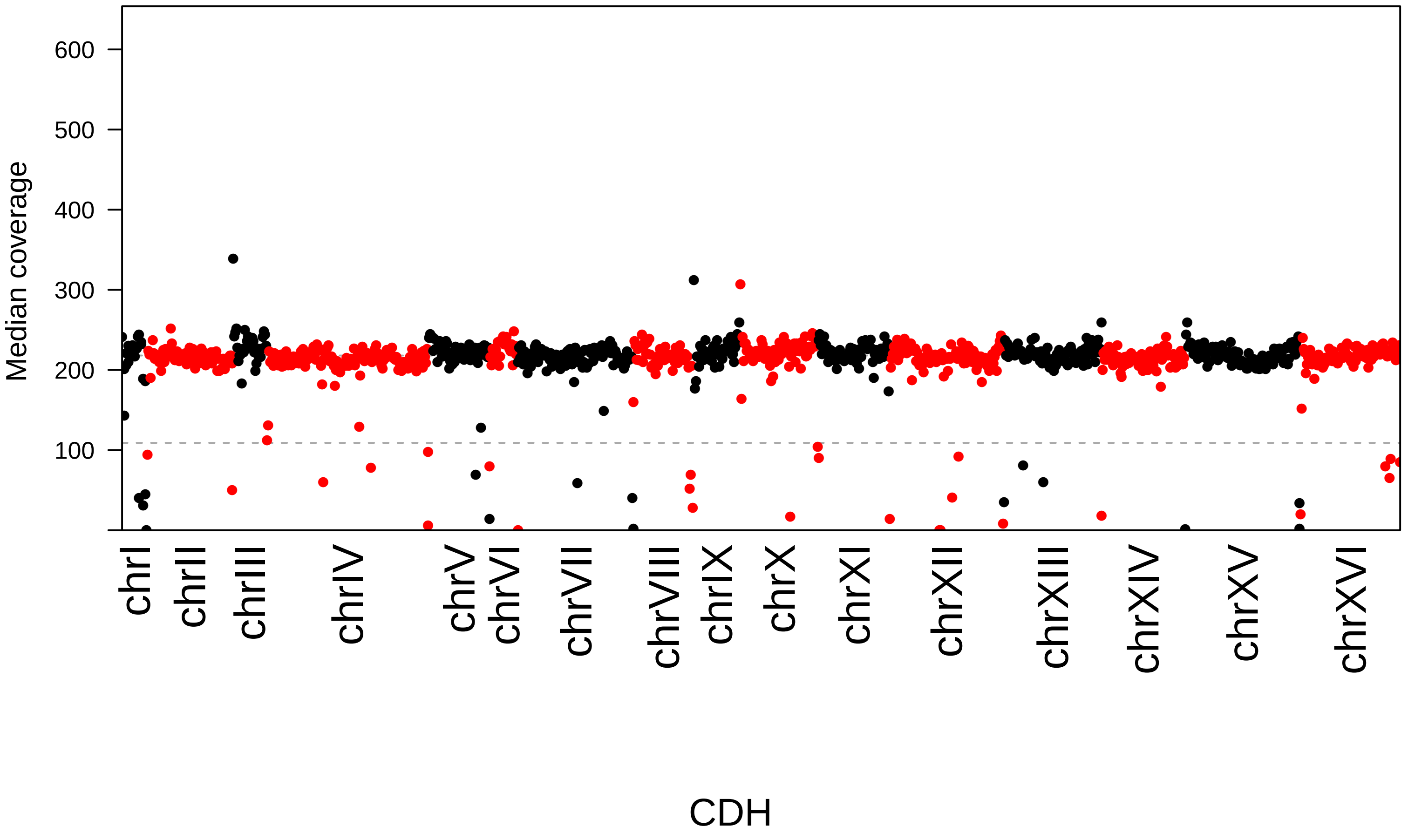

Supplementary Figure S2

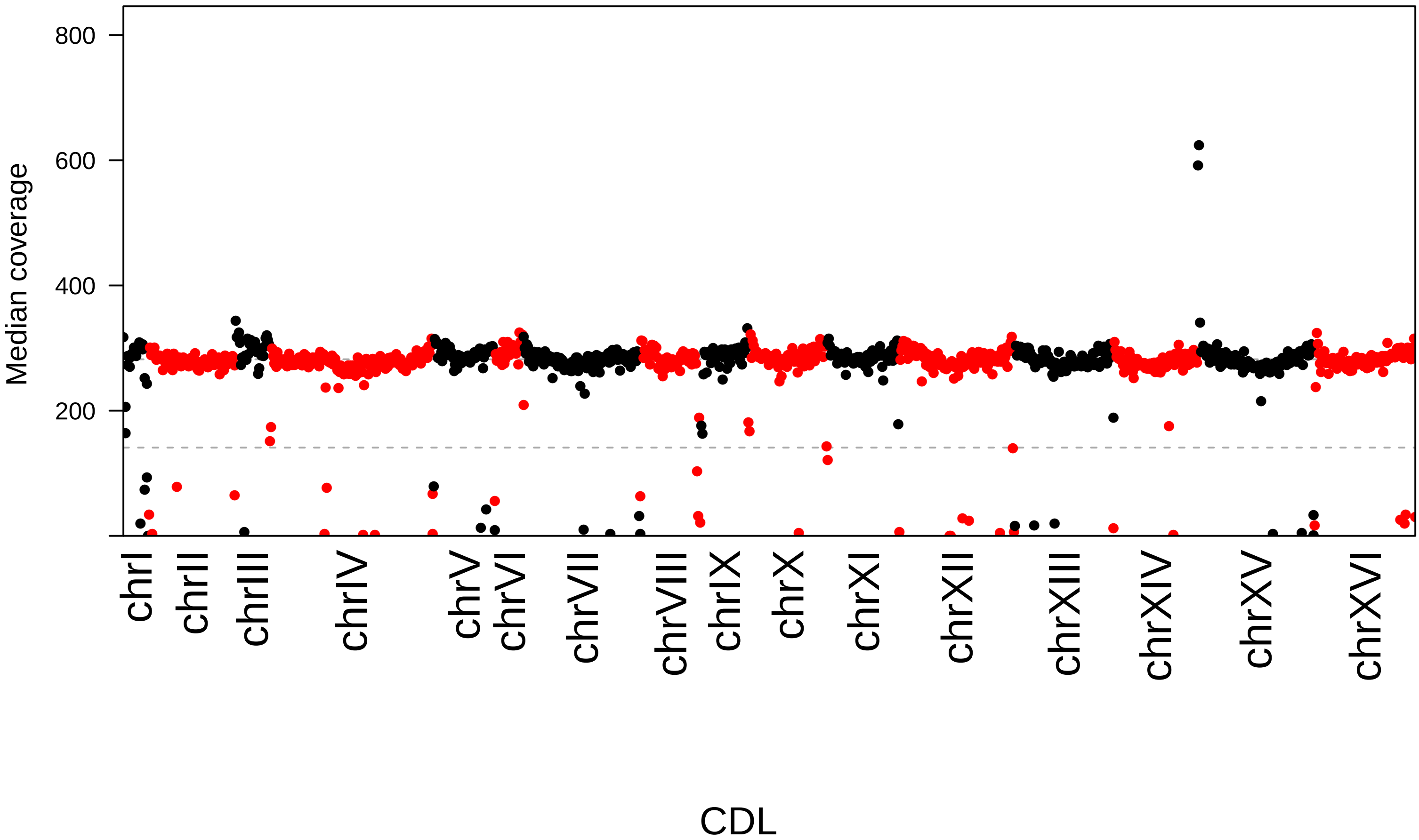

Supplementary Figure S2

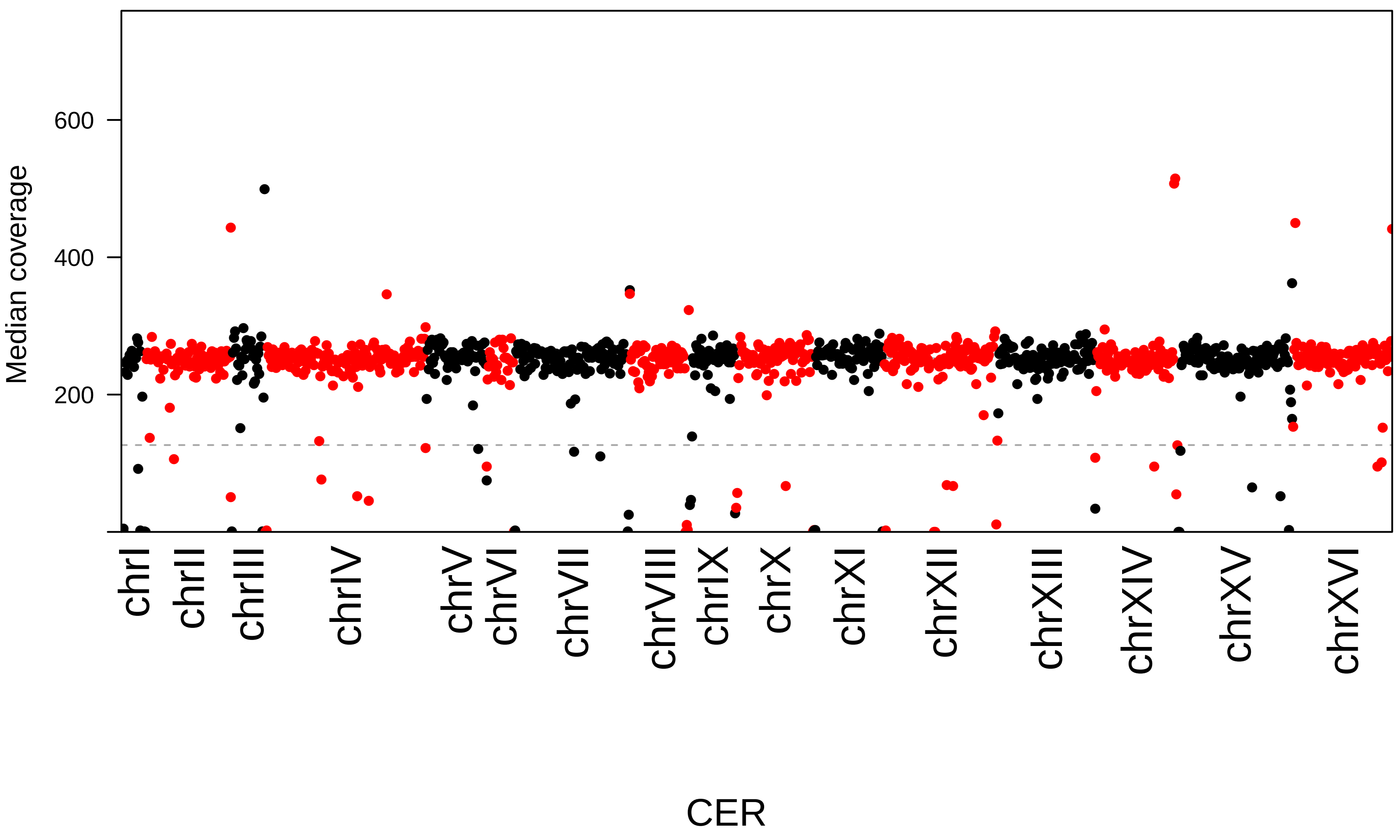

Supplementary Figure S2

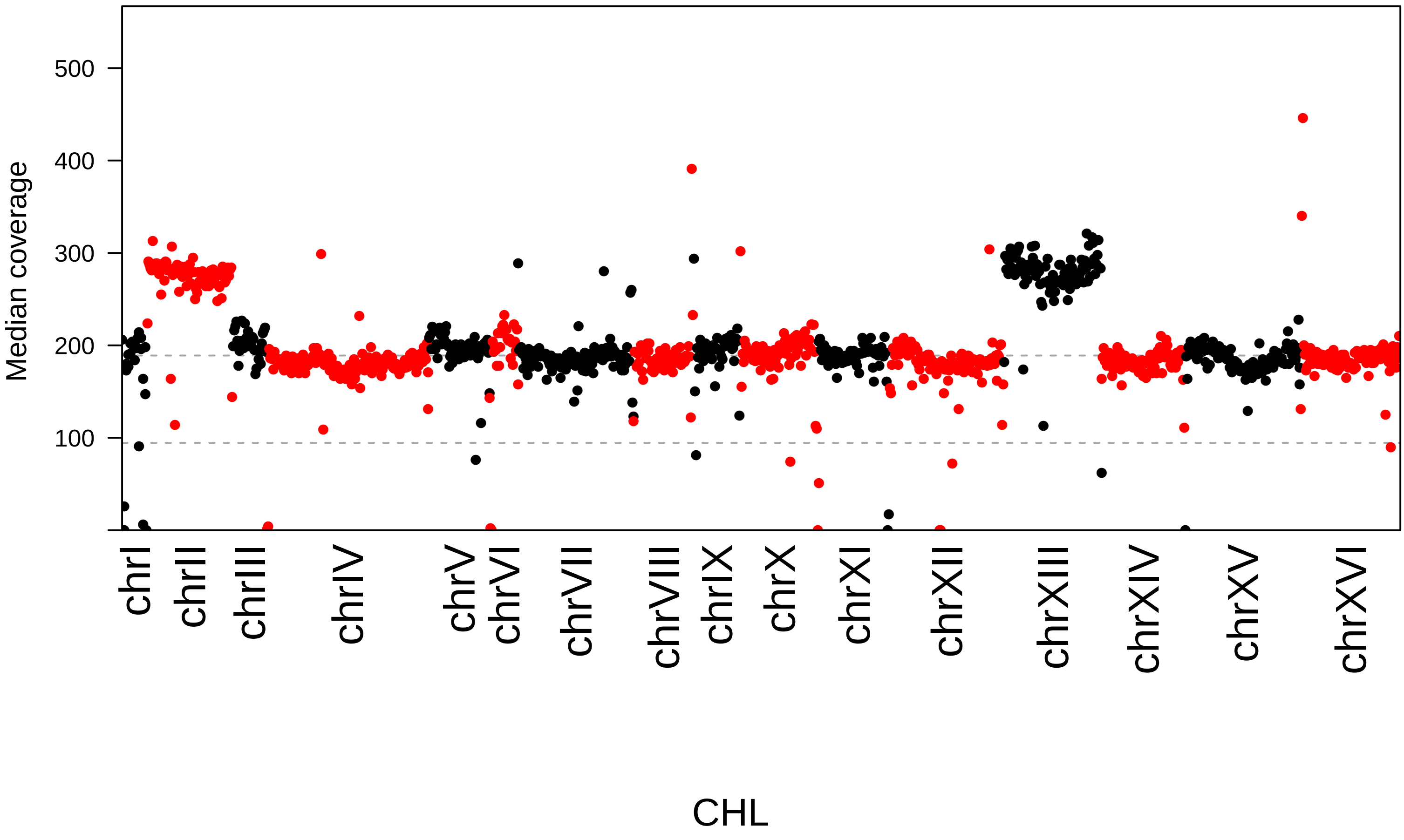

Supplementary Figure S2

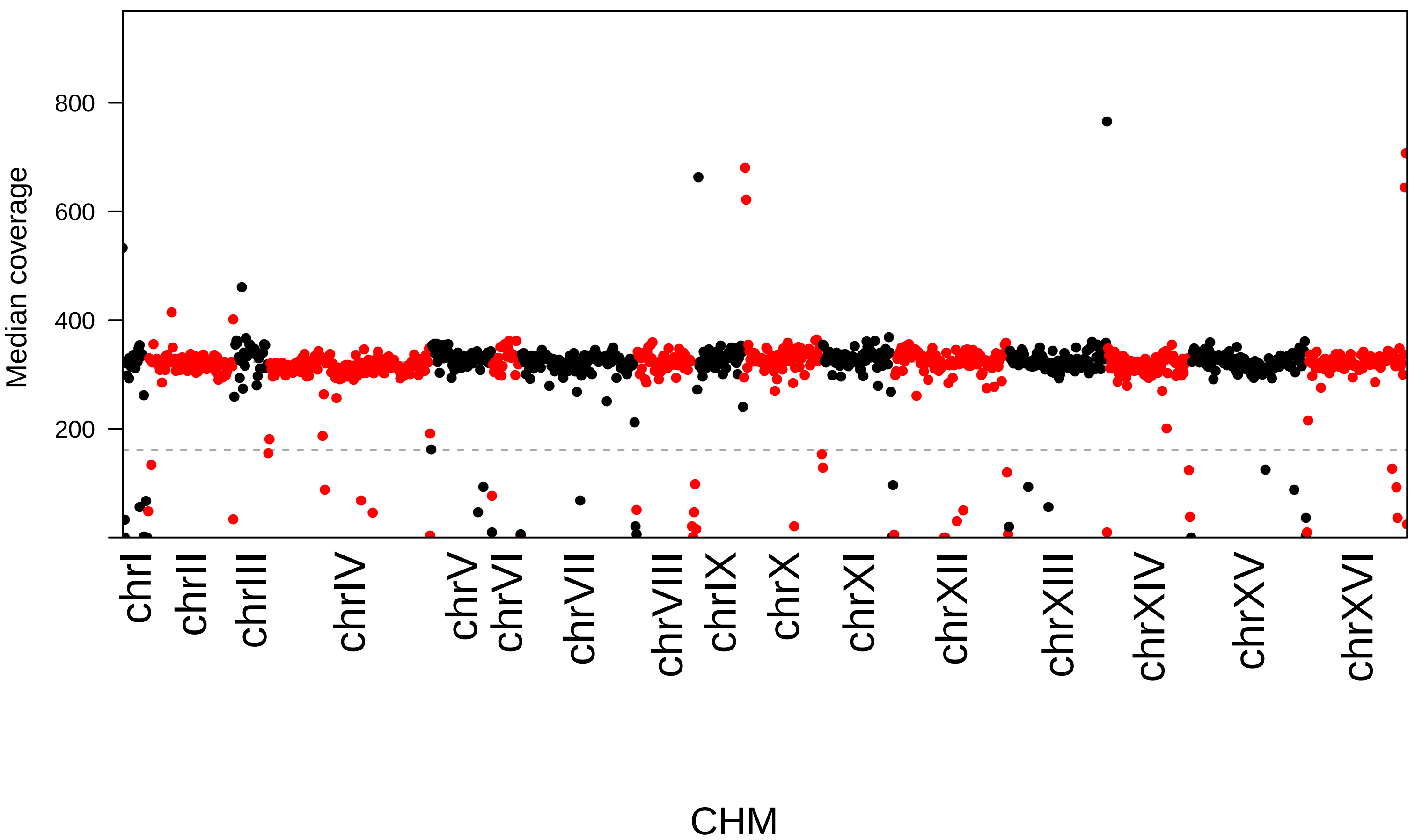

Supplementary Figure S2

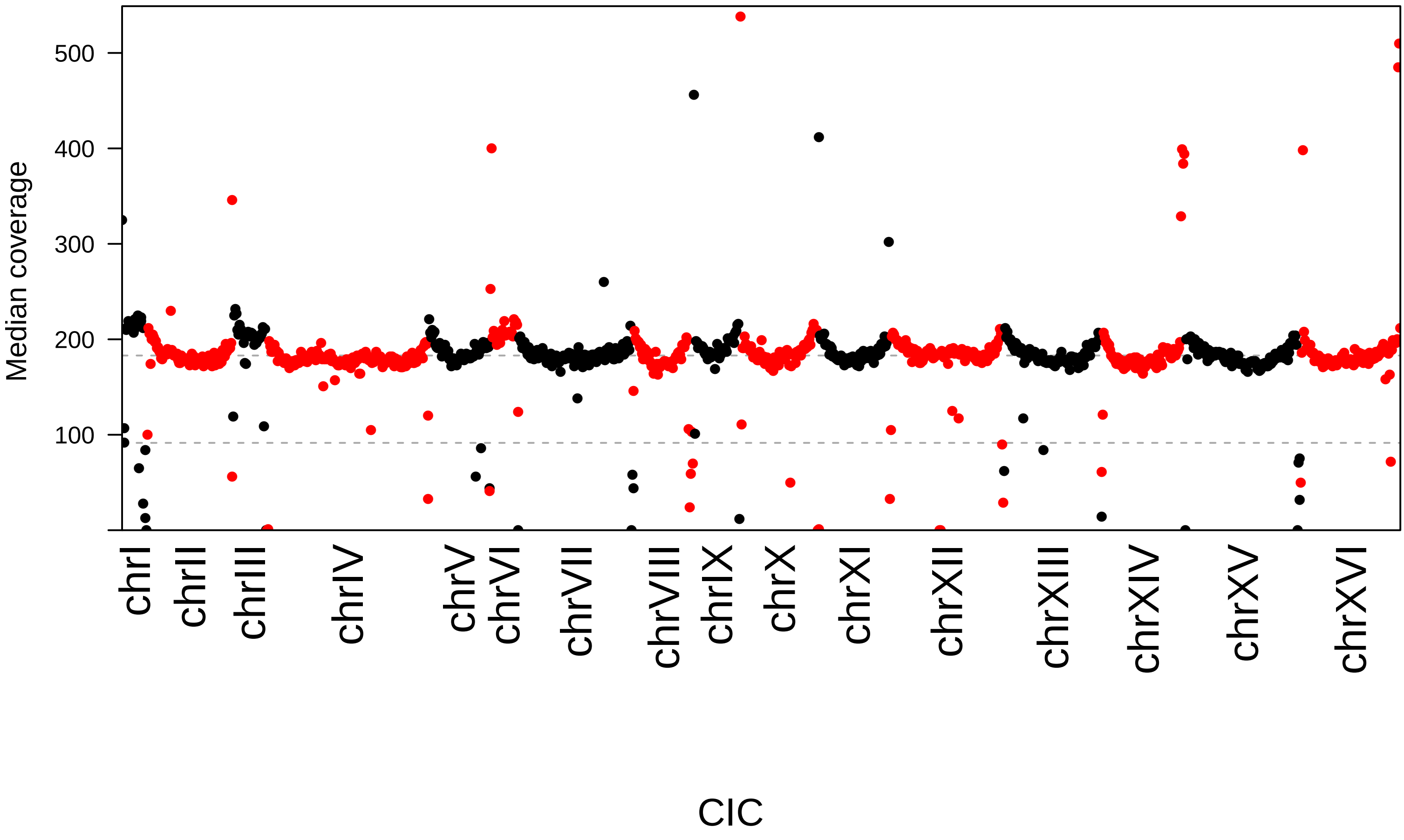

Supplementary Figure S2

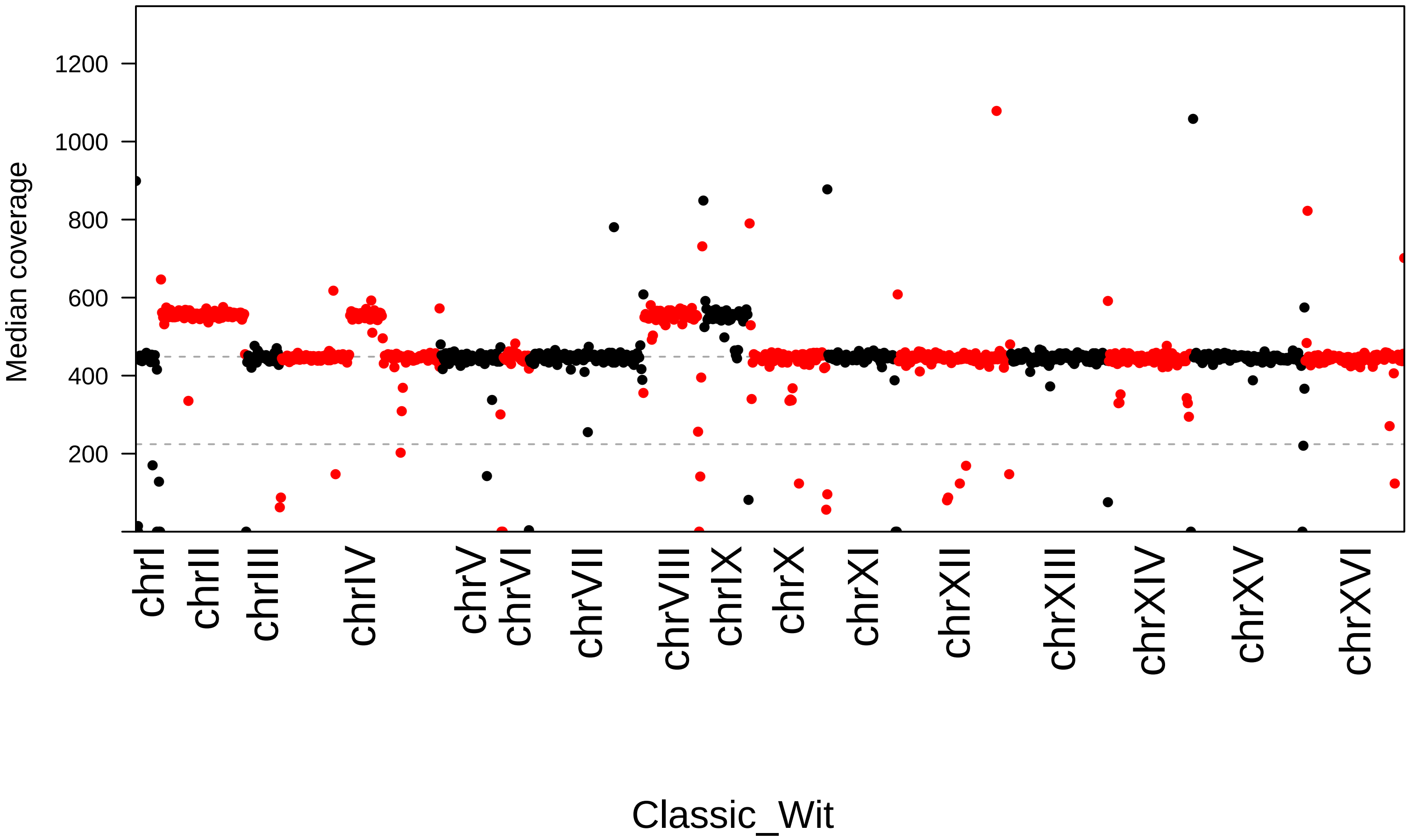

Supplementary Figure S2

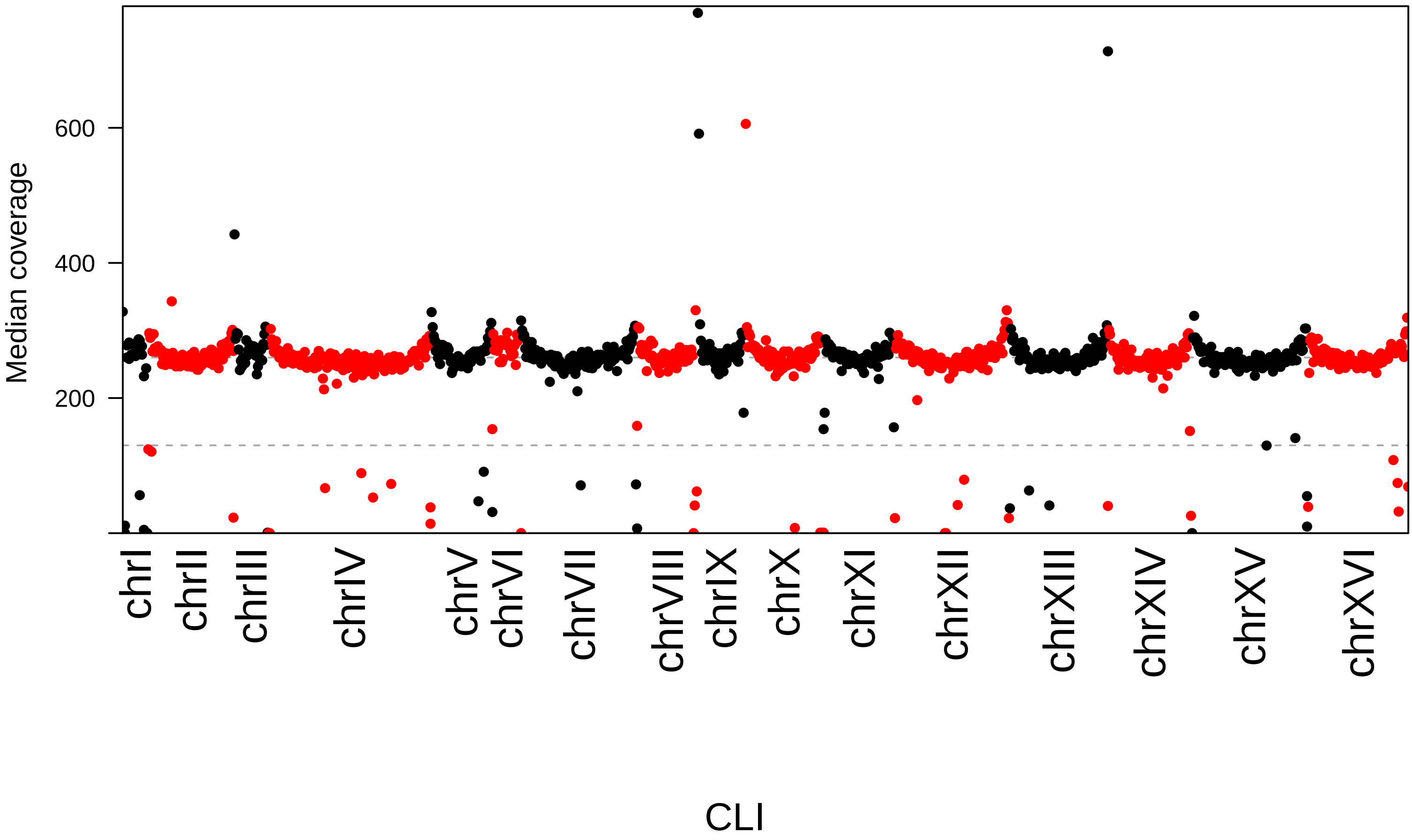

Supplementary Figure S2

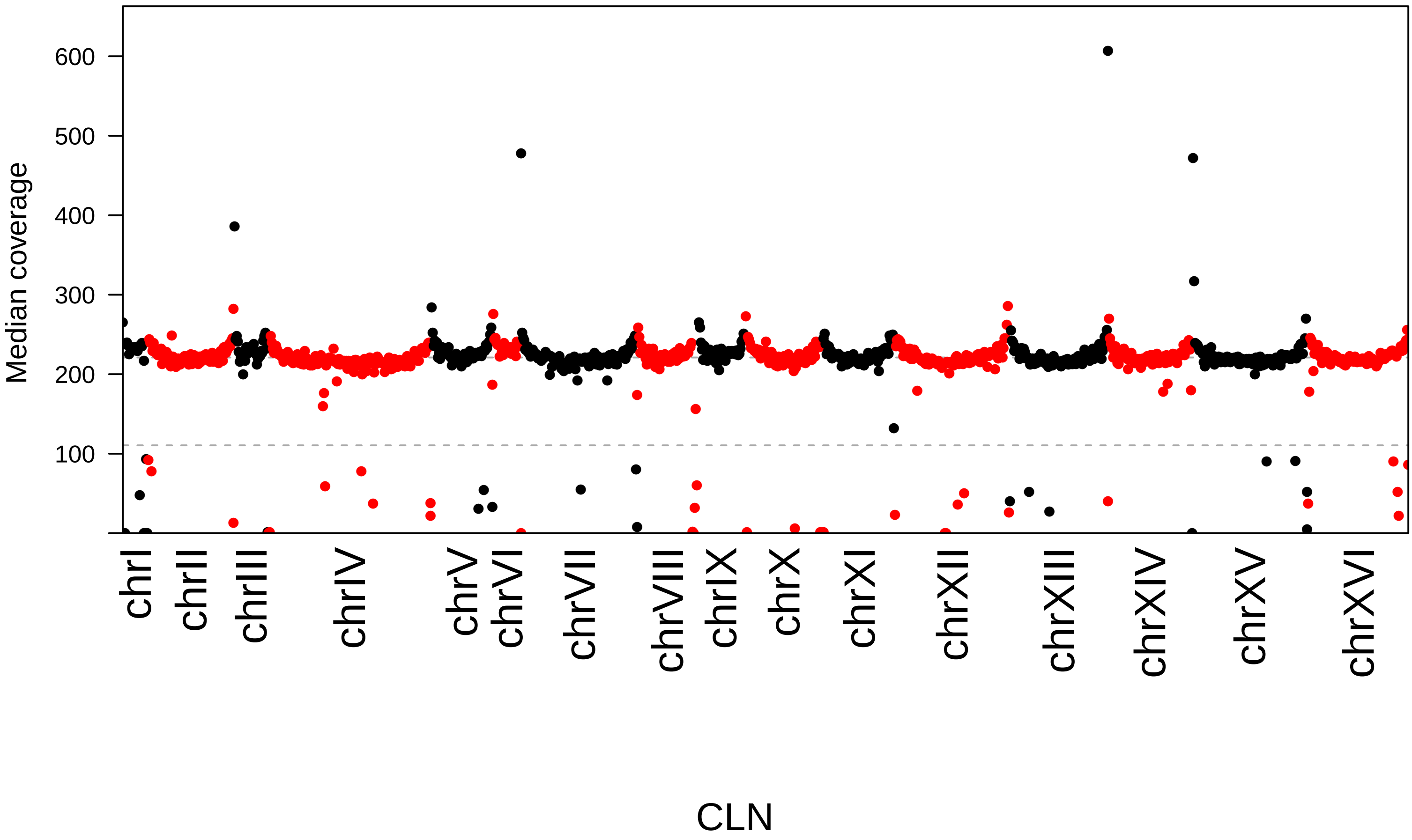

Supplementary Figure S2

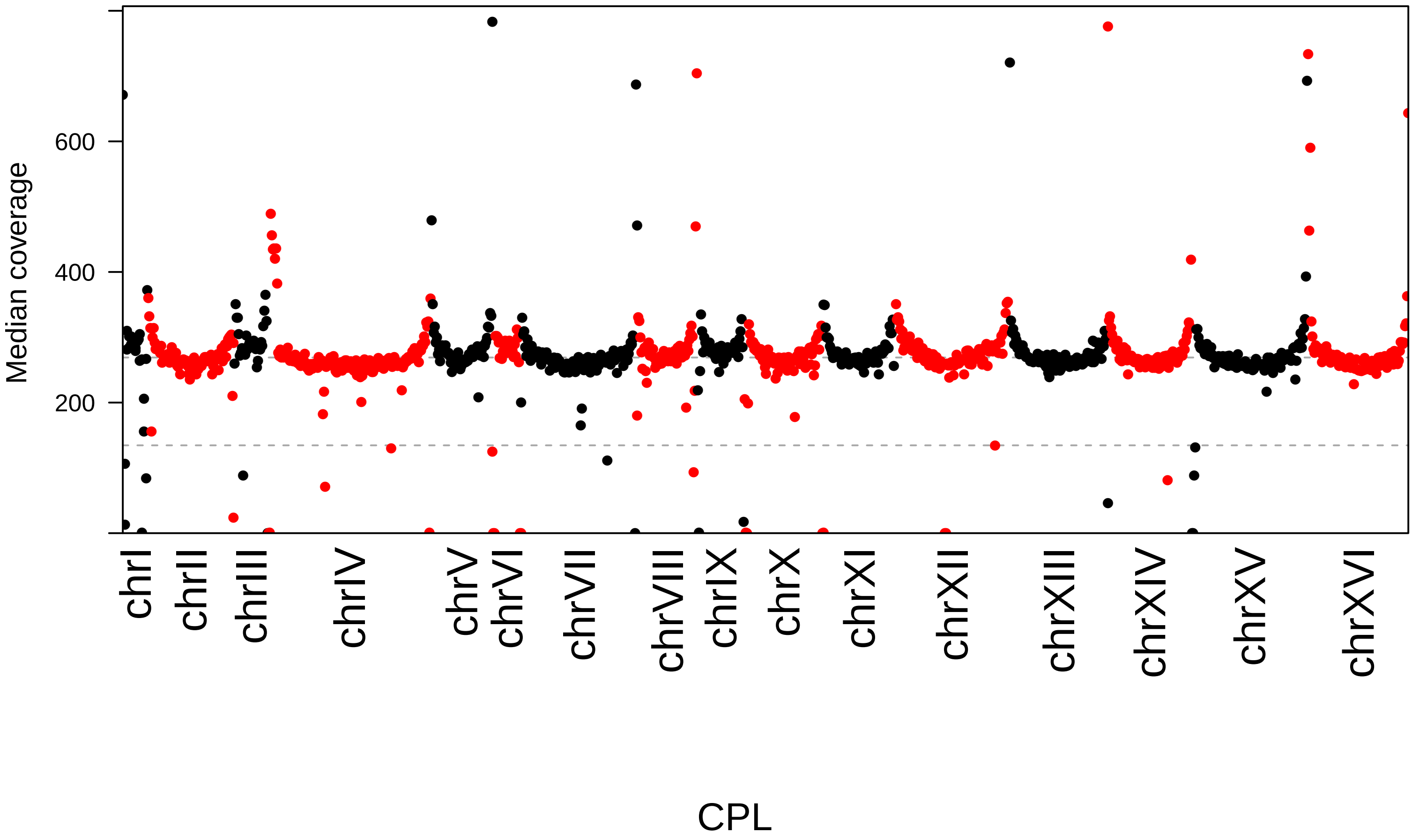

Supplementary Figure S2

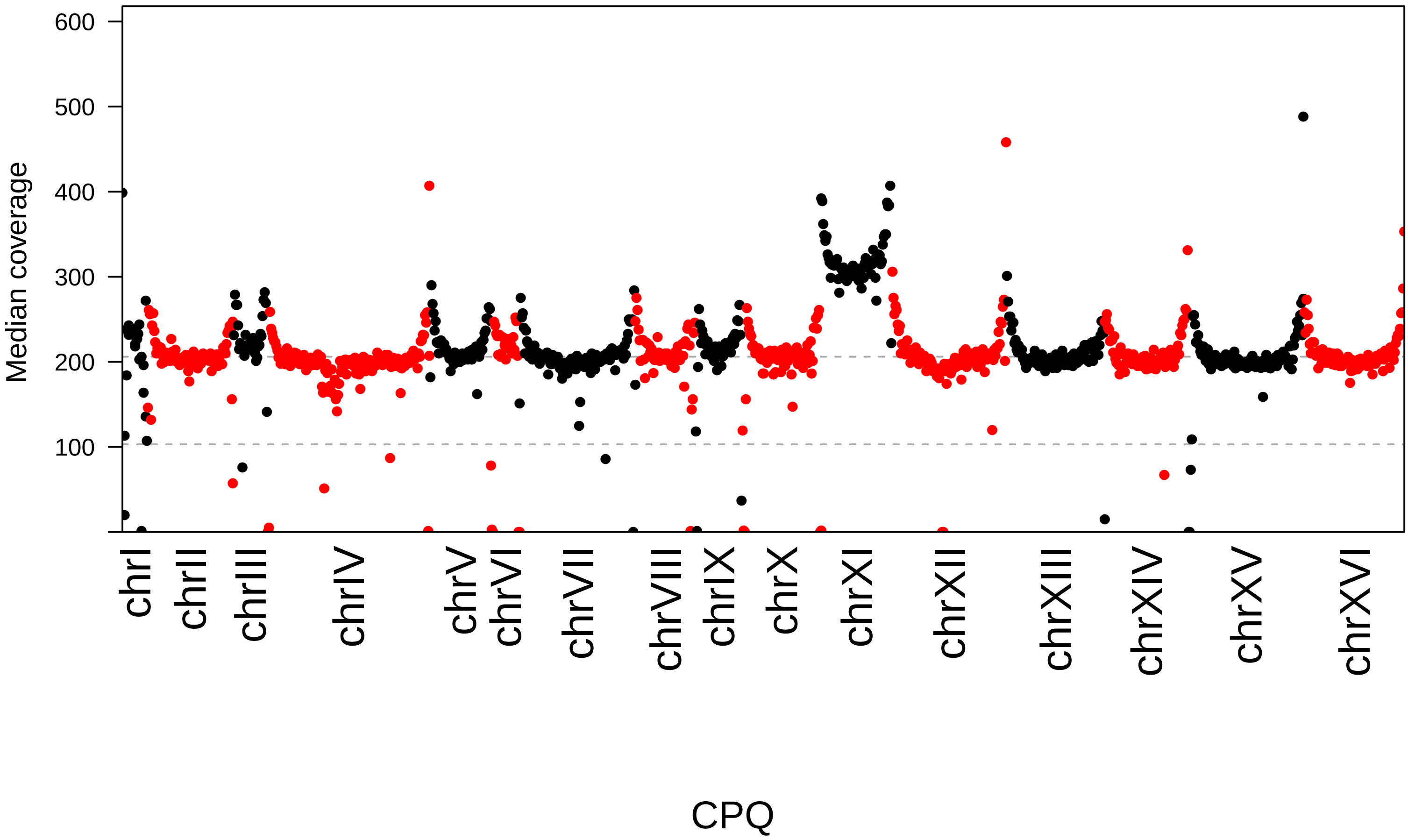

Supplementary Figure S2

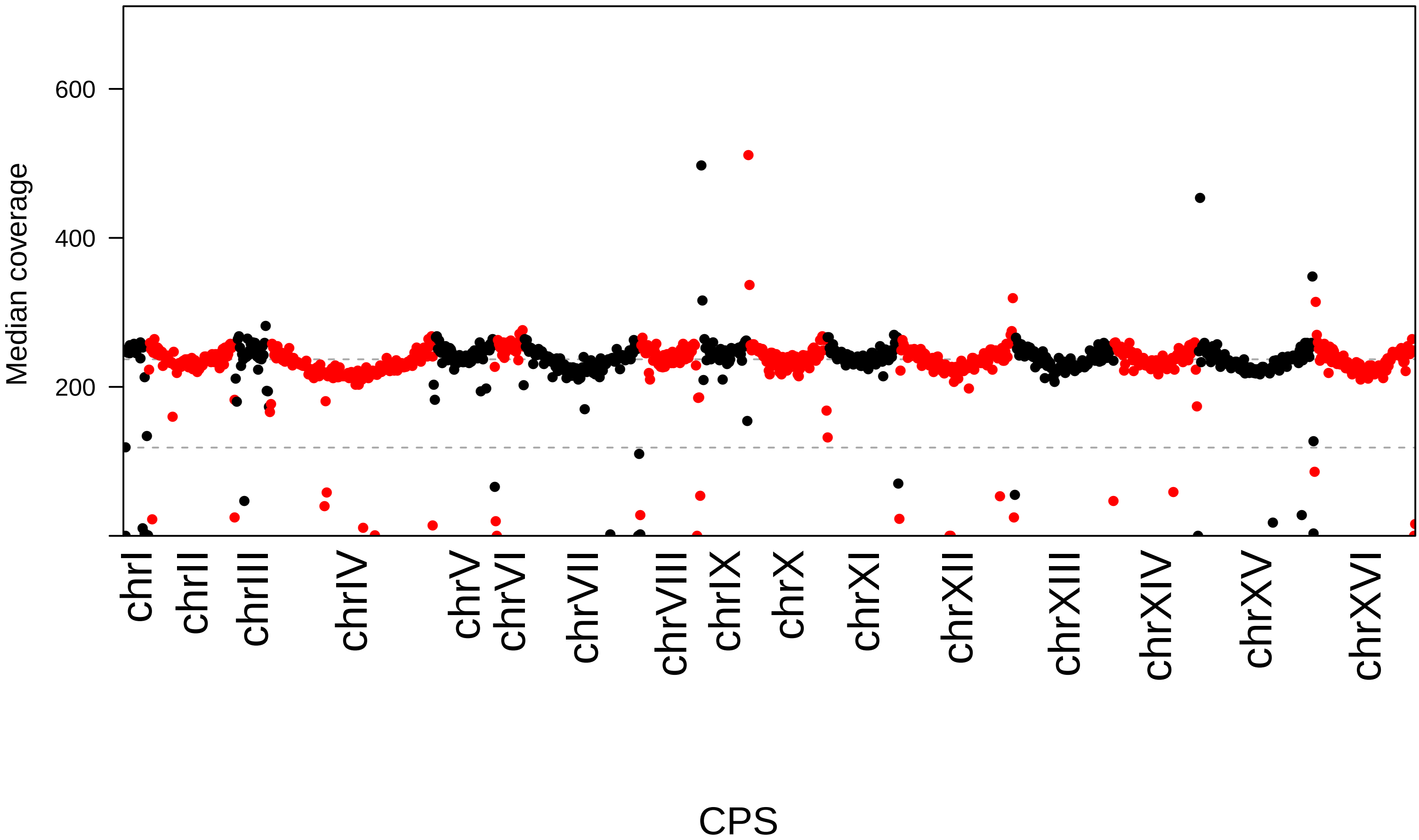

Supplementary Figure S2

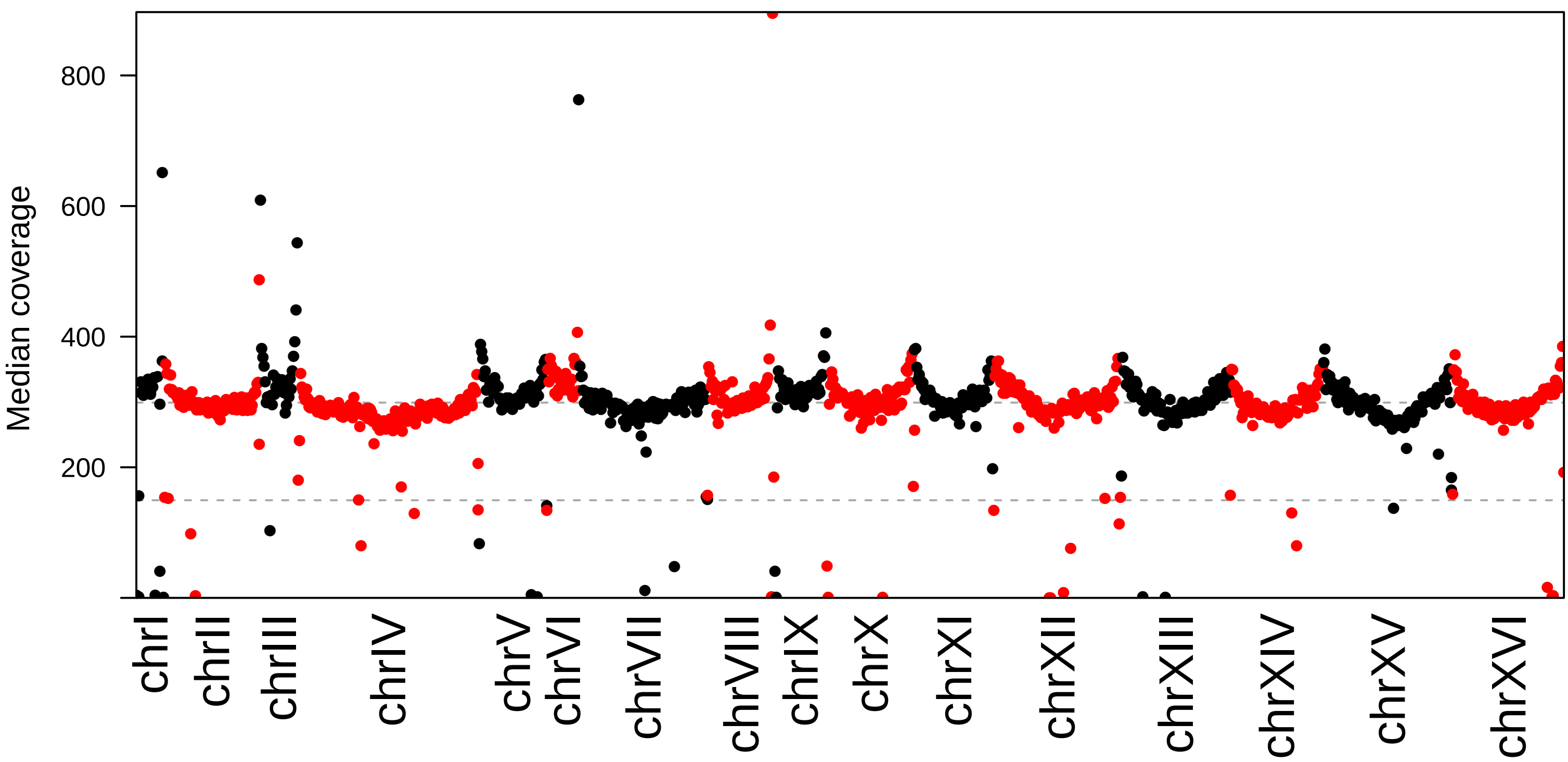

CQD

Supplementary Figure S2

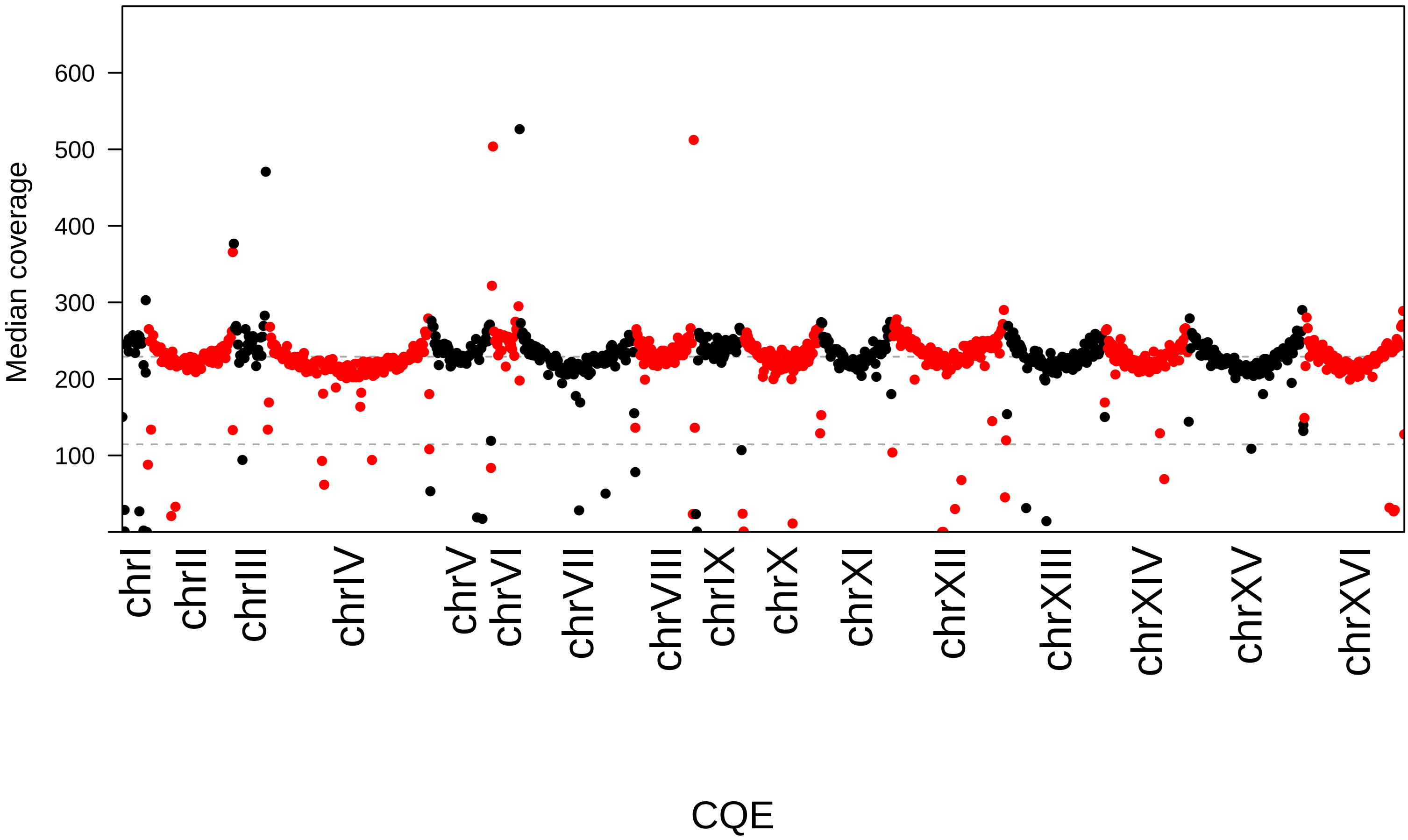

Supplementary Figure S2

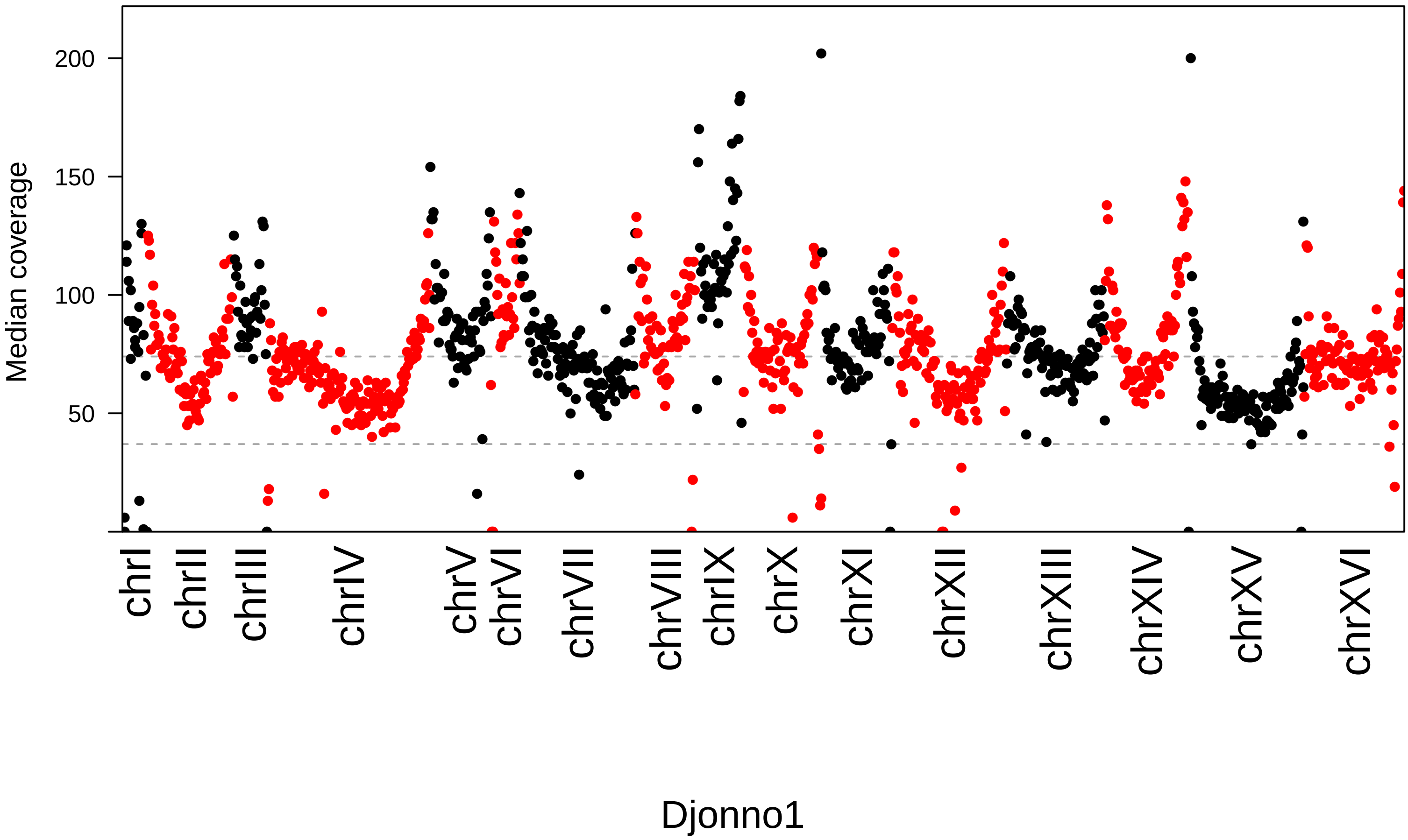

Supplementary Figure S2

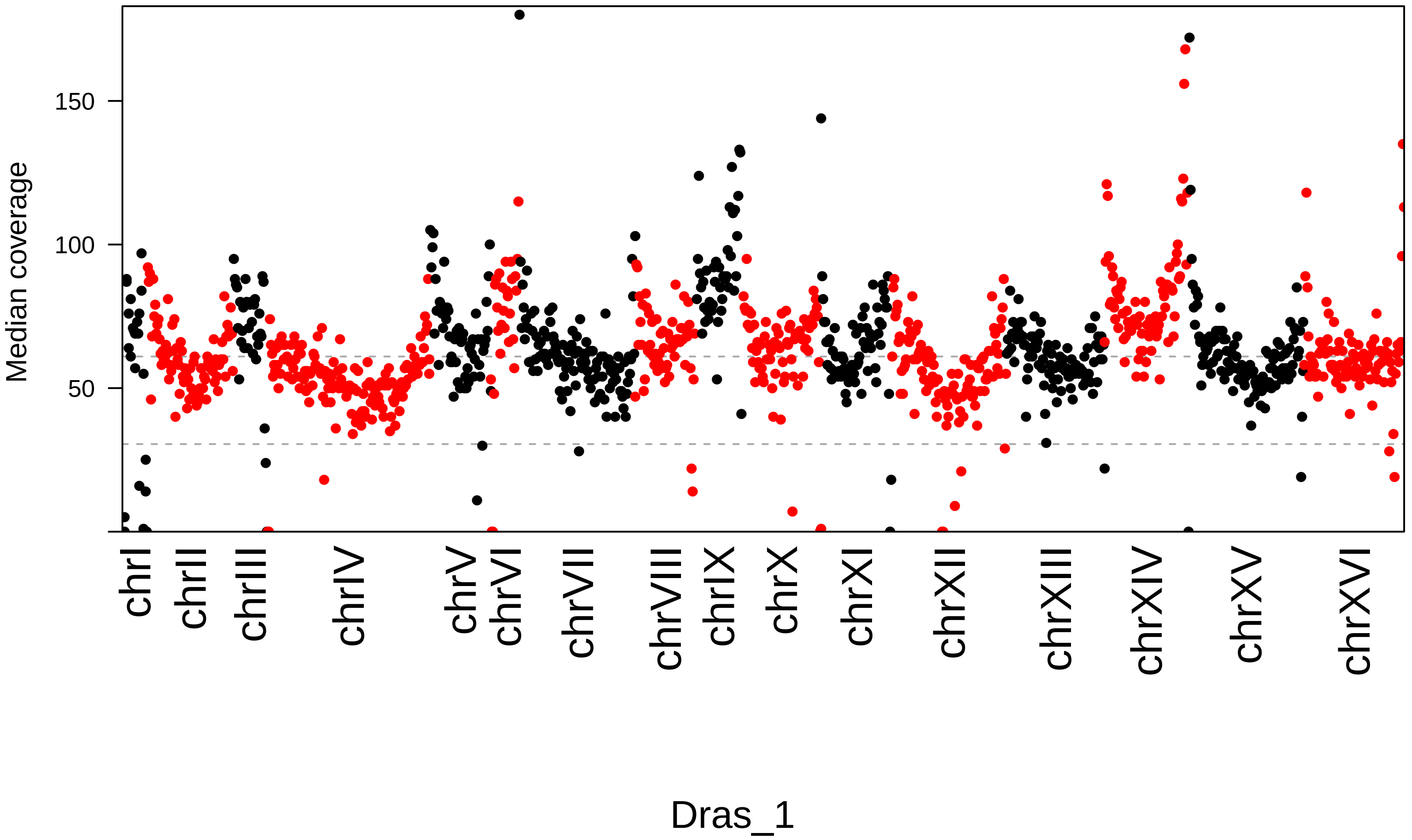

Supplementary Figure S2

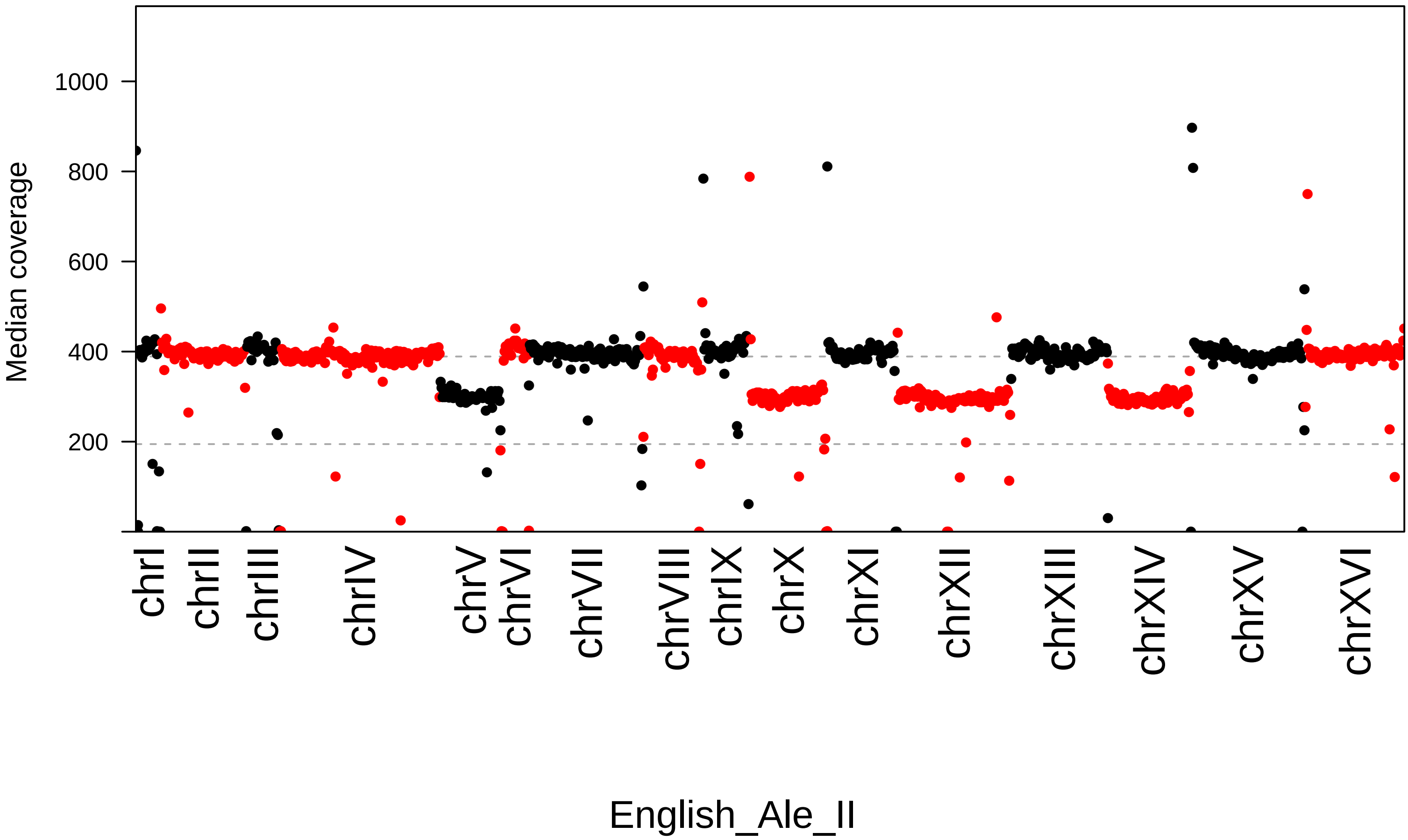

Supplementary Figure S2

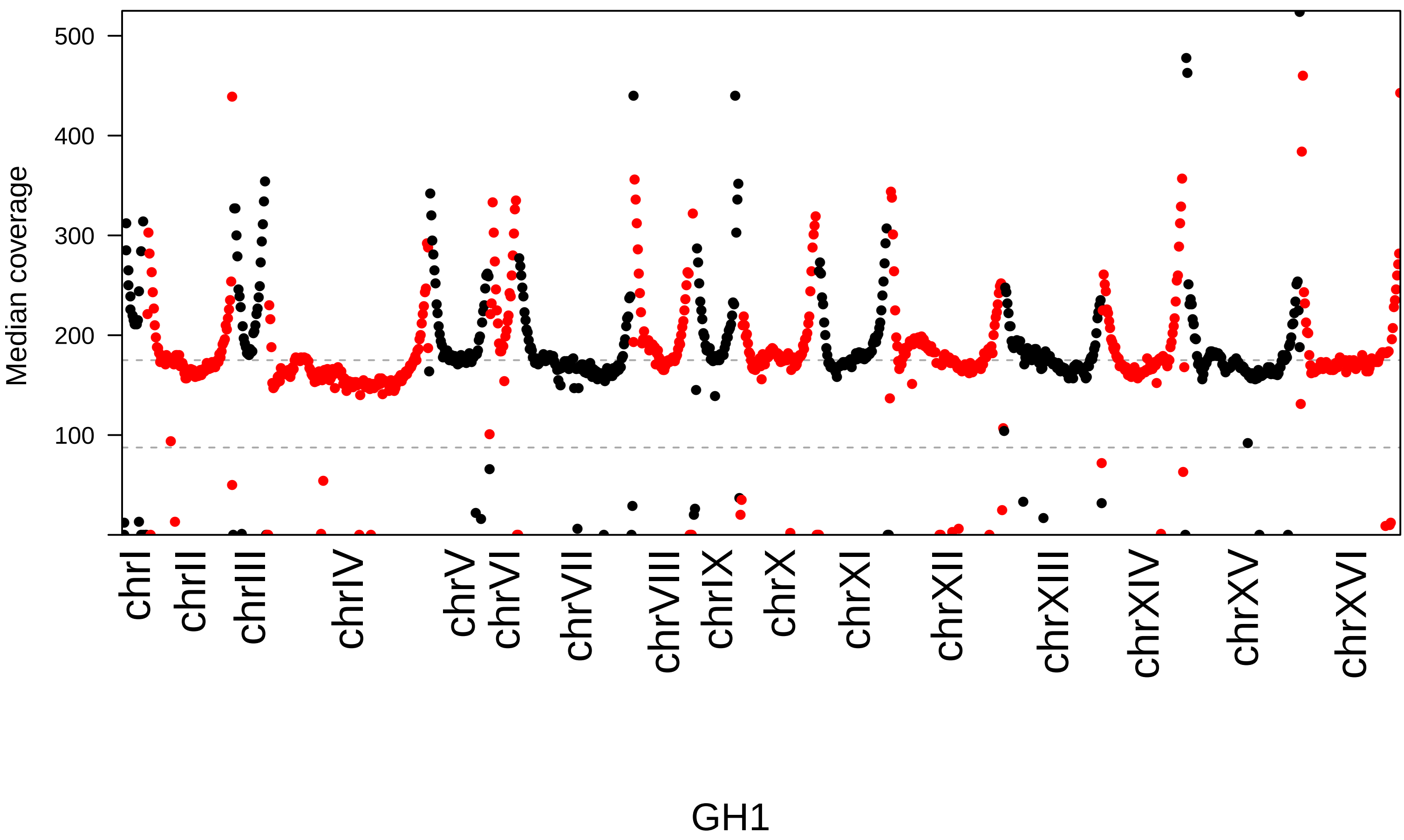

Supplementary Figure S2

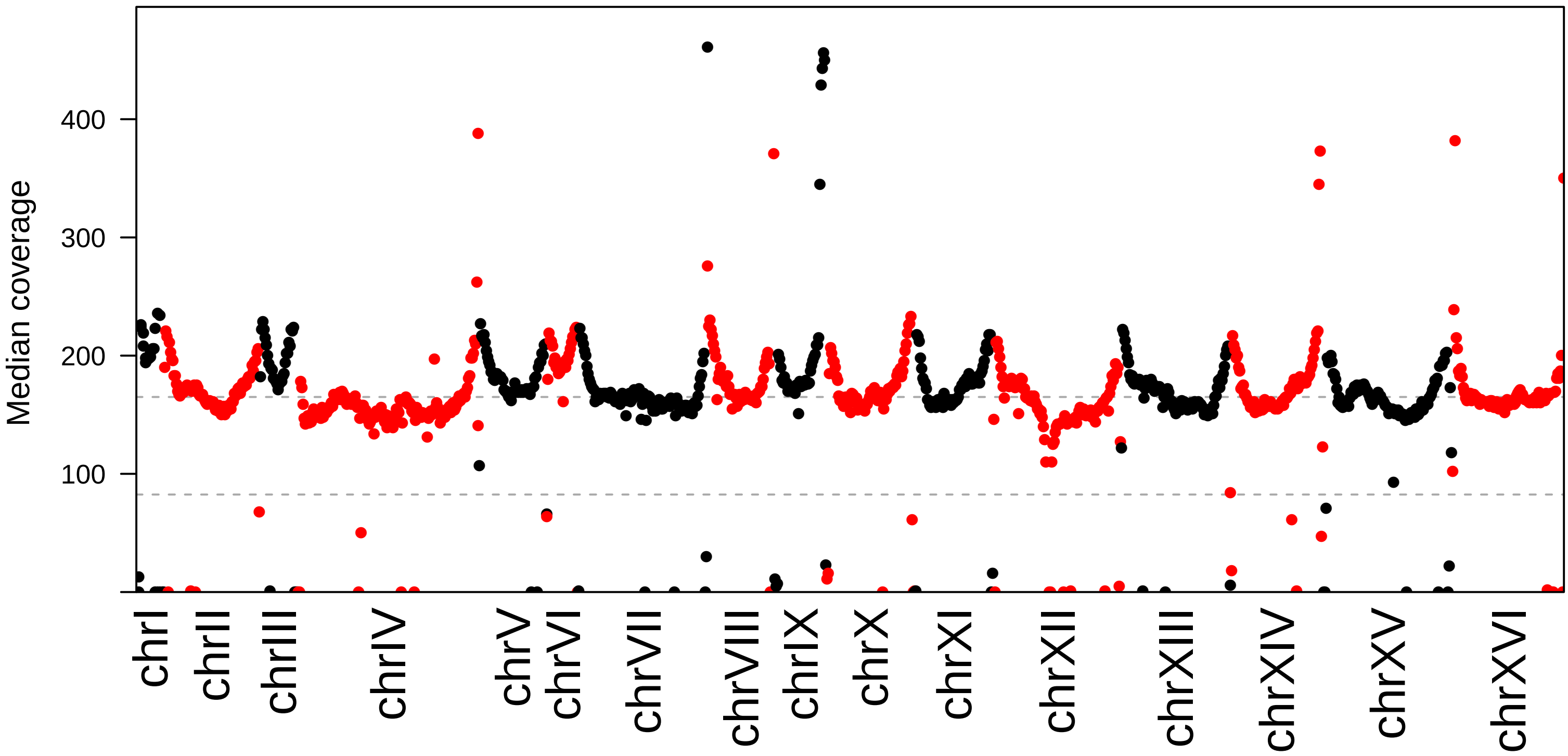

GH2

Supplementary Figure S2

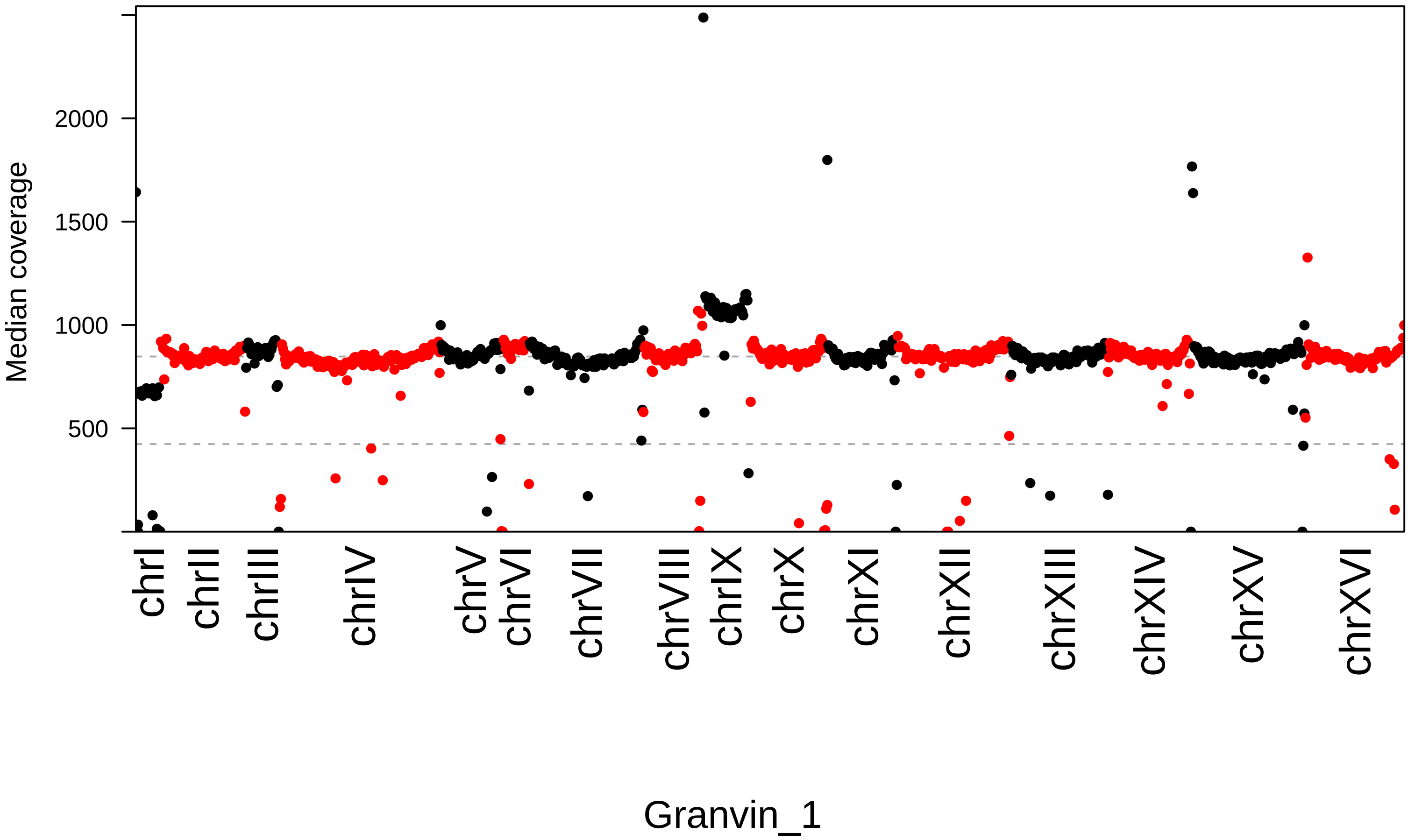

Supplementary Figure S2

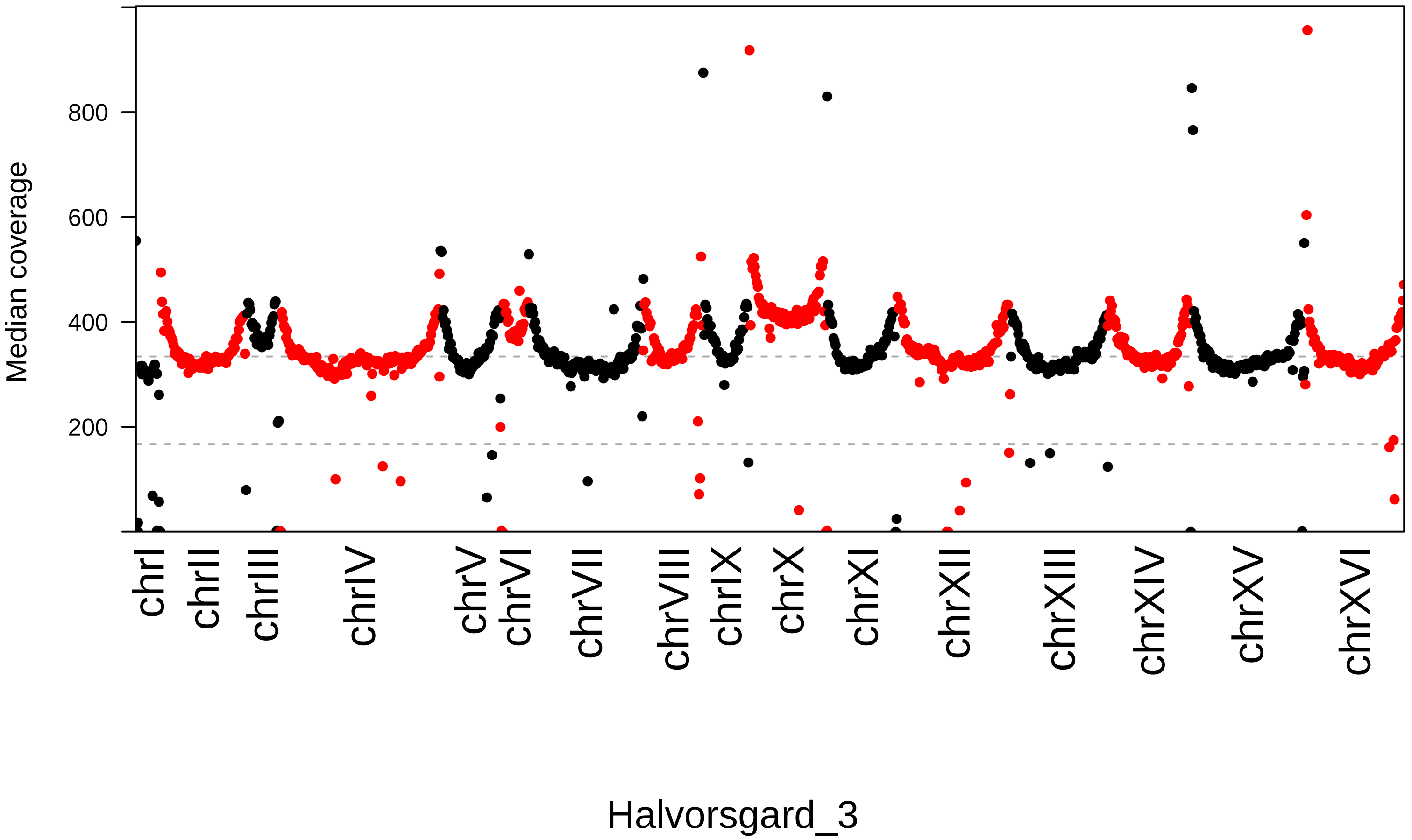

Supplementary Figure S2

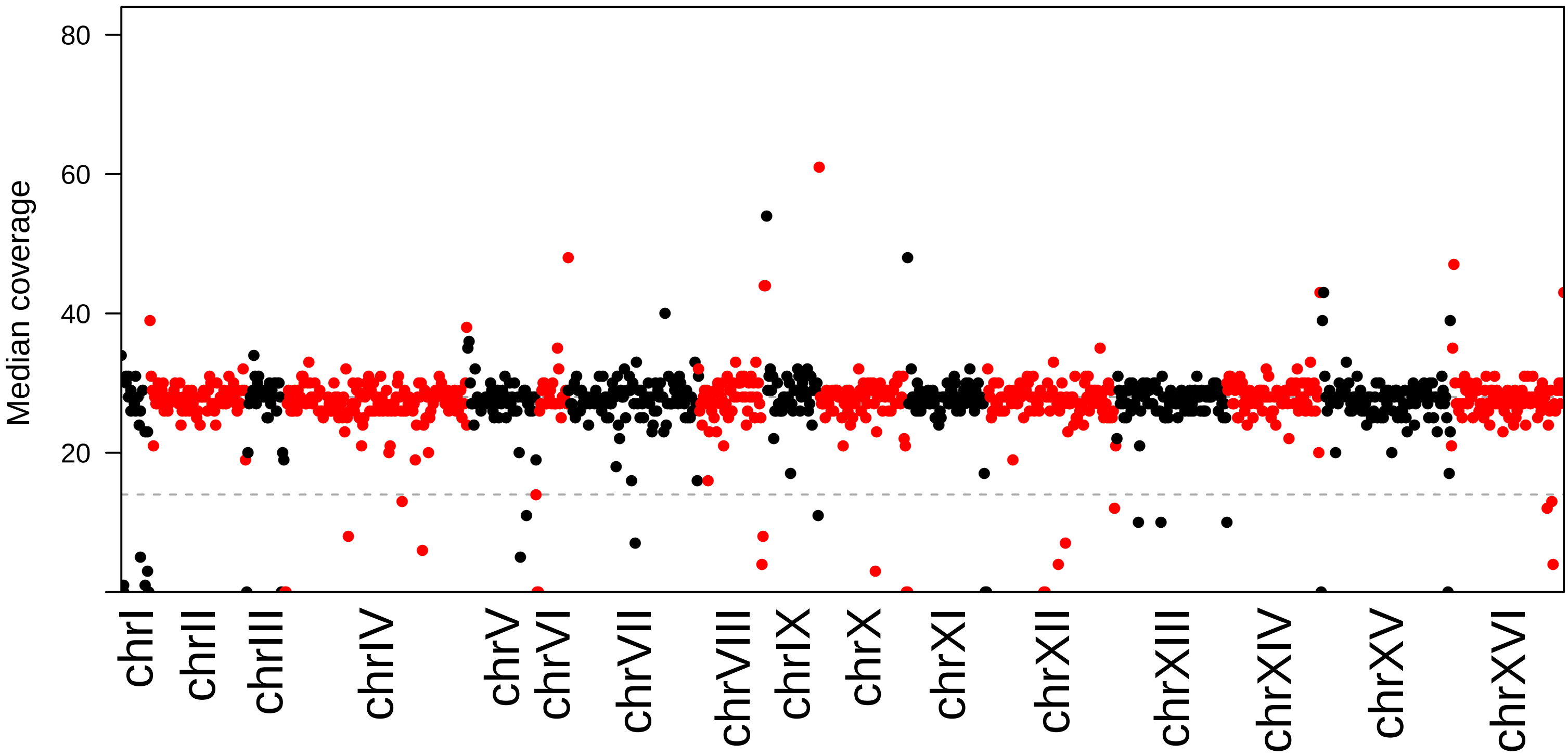

Halvorsgard_6

Supplementary Figure S2

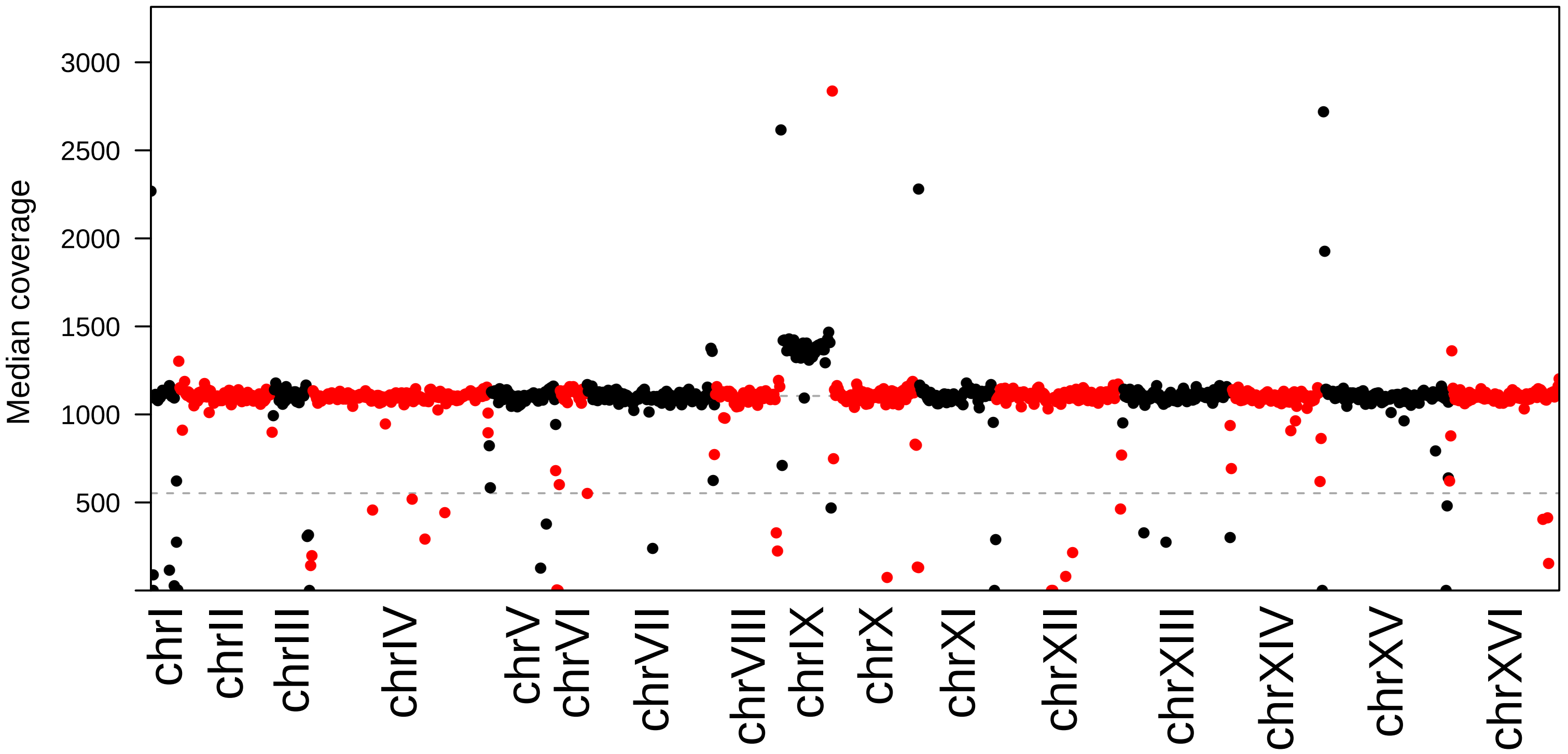

Hornindal_1

Supplementary Figure S2

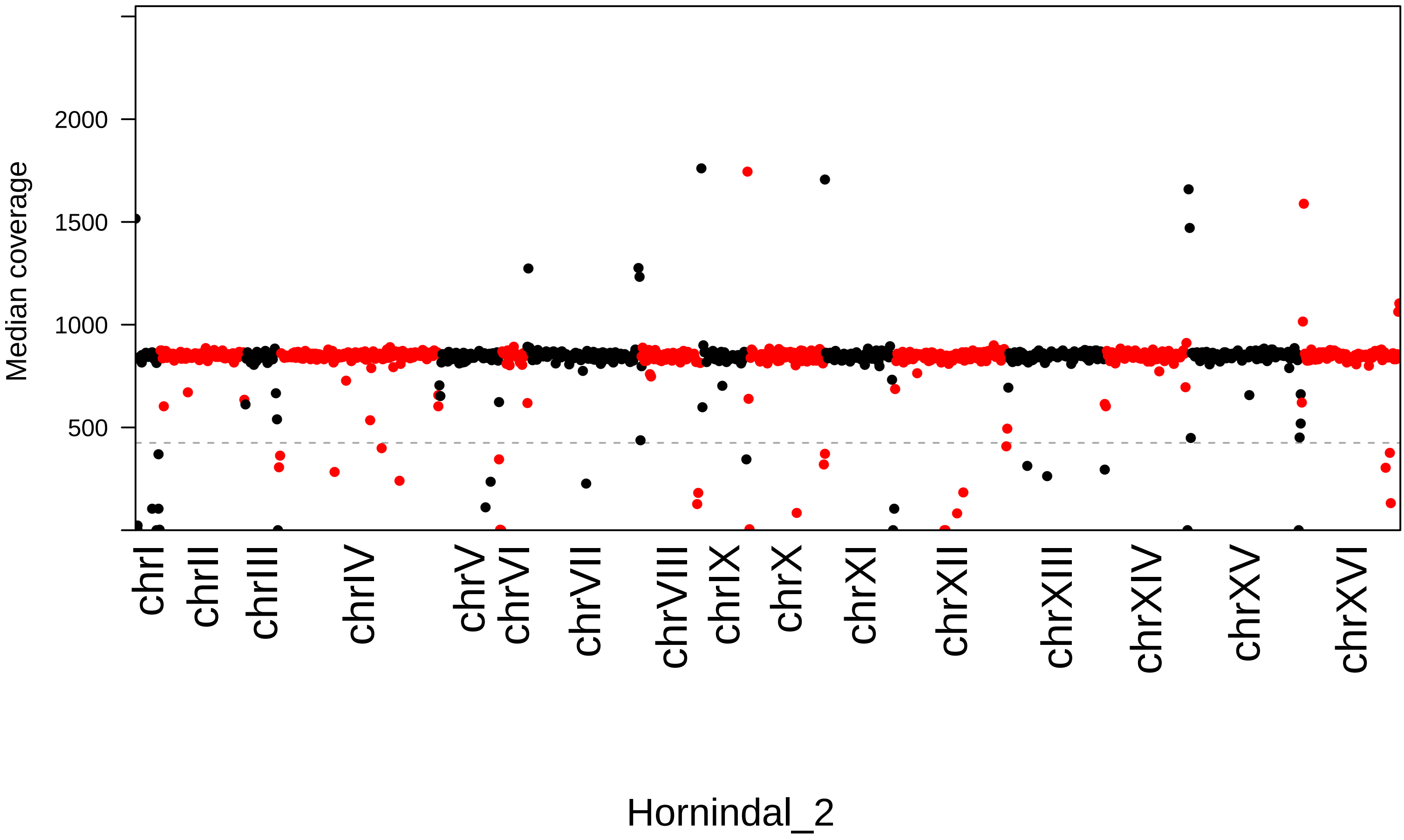

Supplementary Figure S2

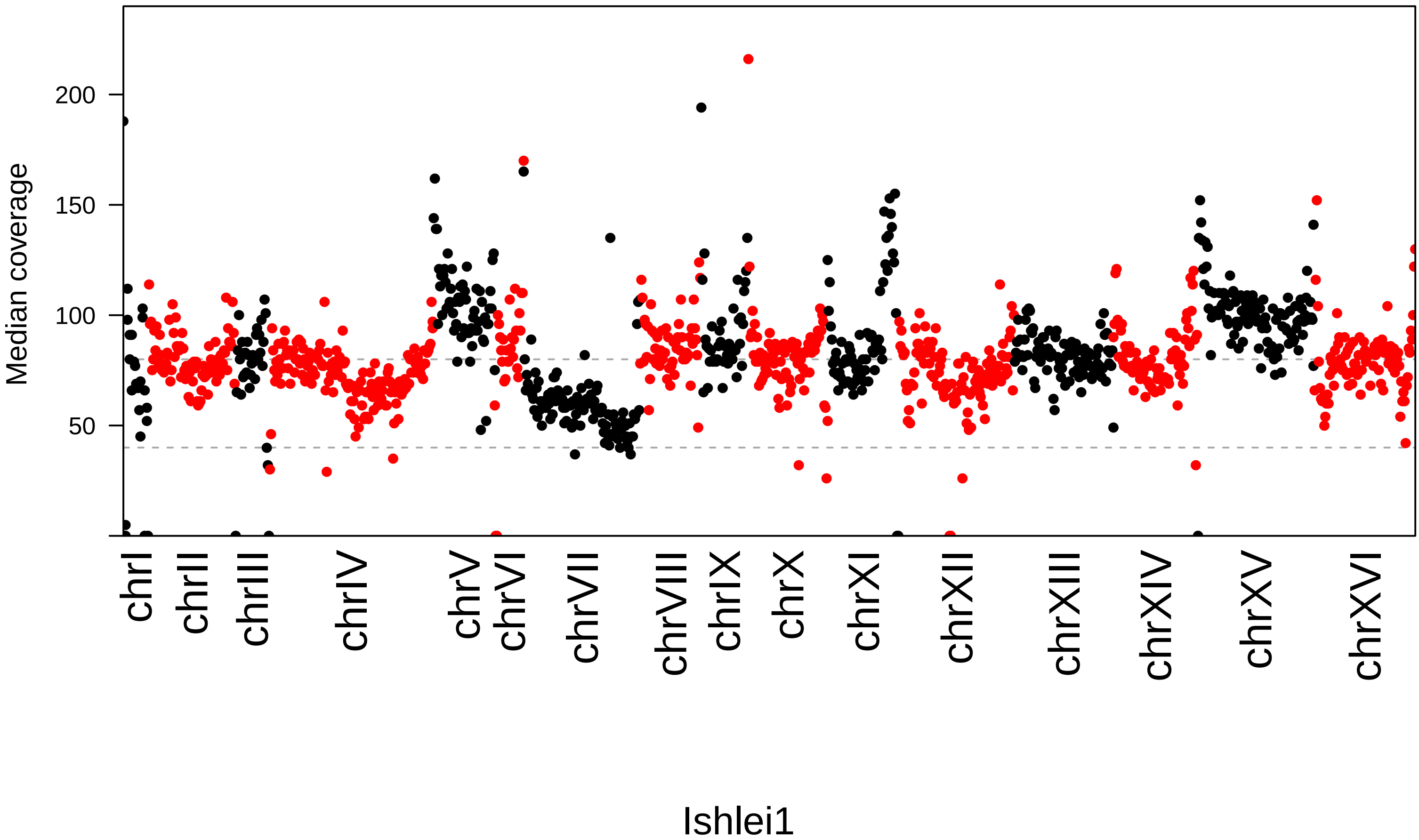

Supplementary Figure S2

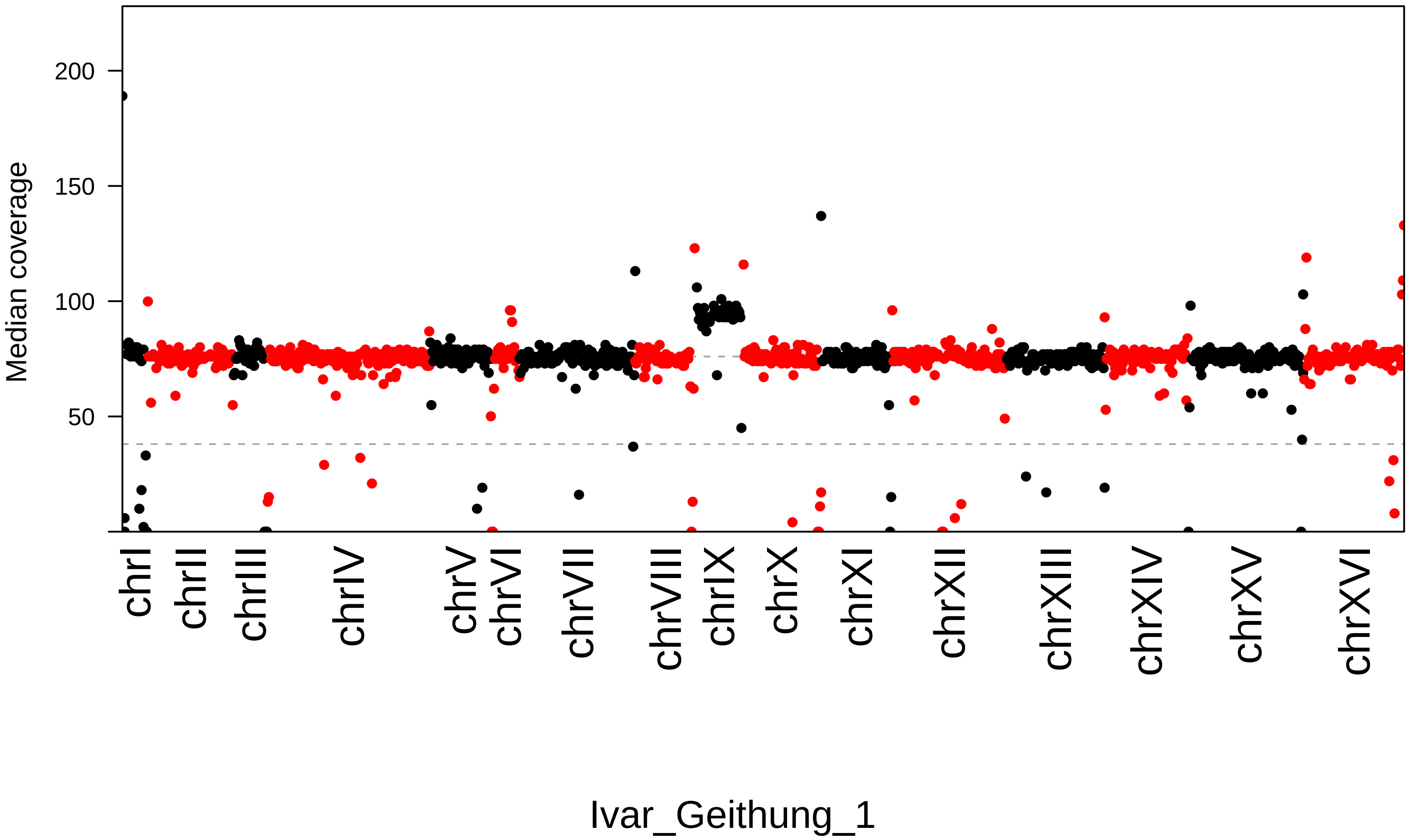

Supplementary Figure S2

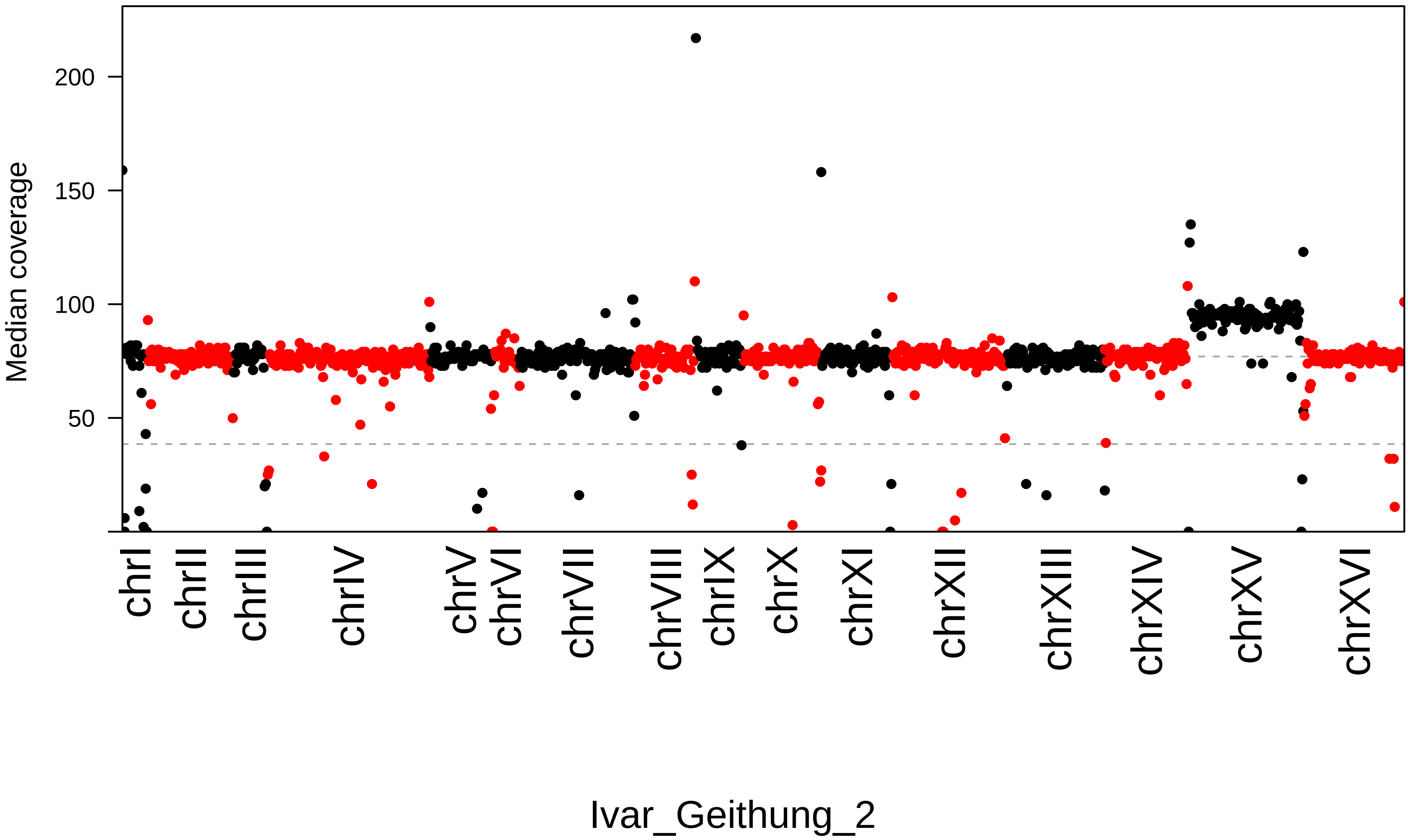

Supplementary Figure S2

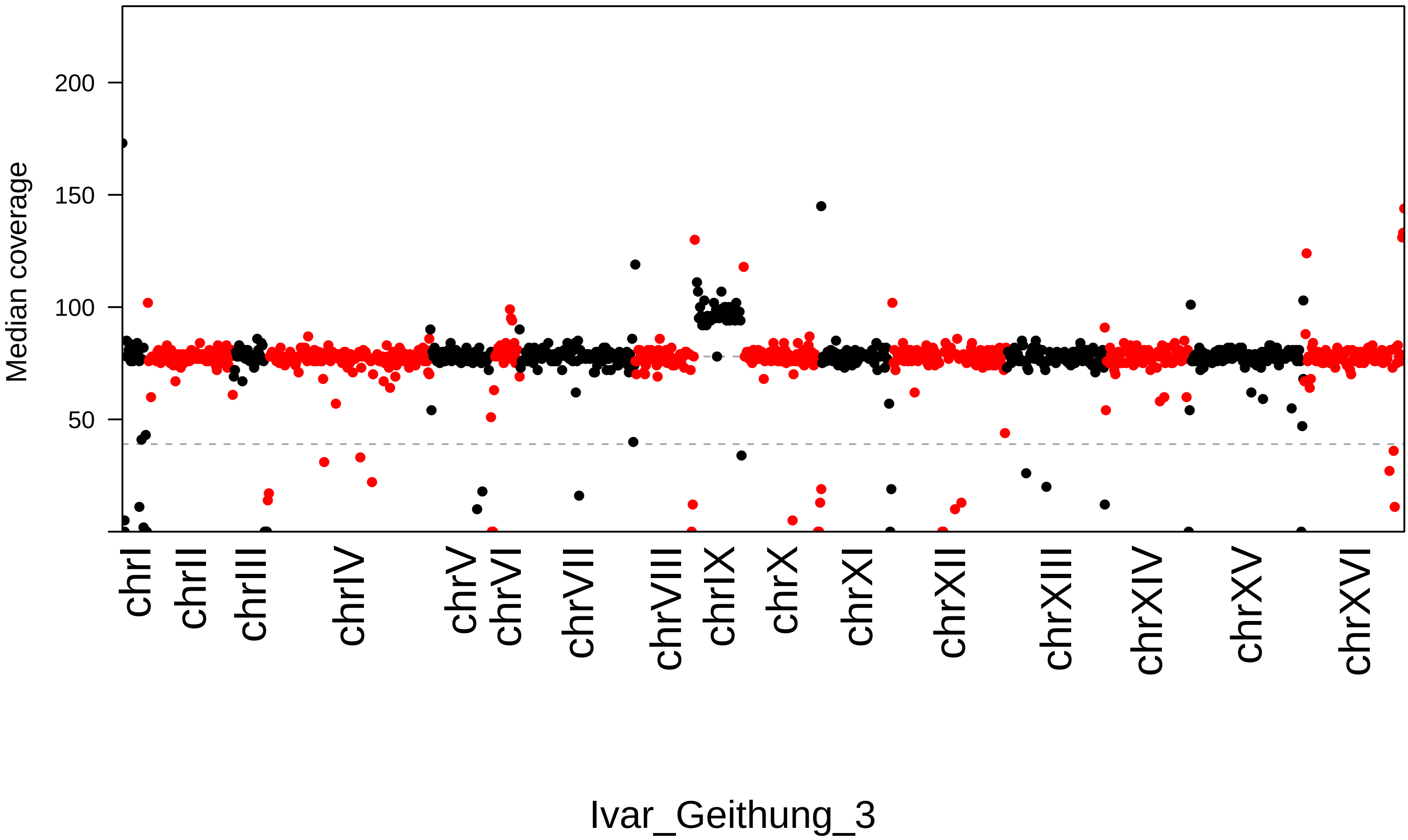

Supplementary Figure S2

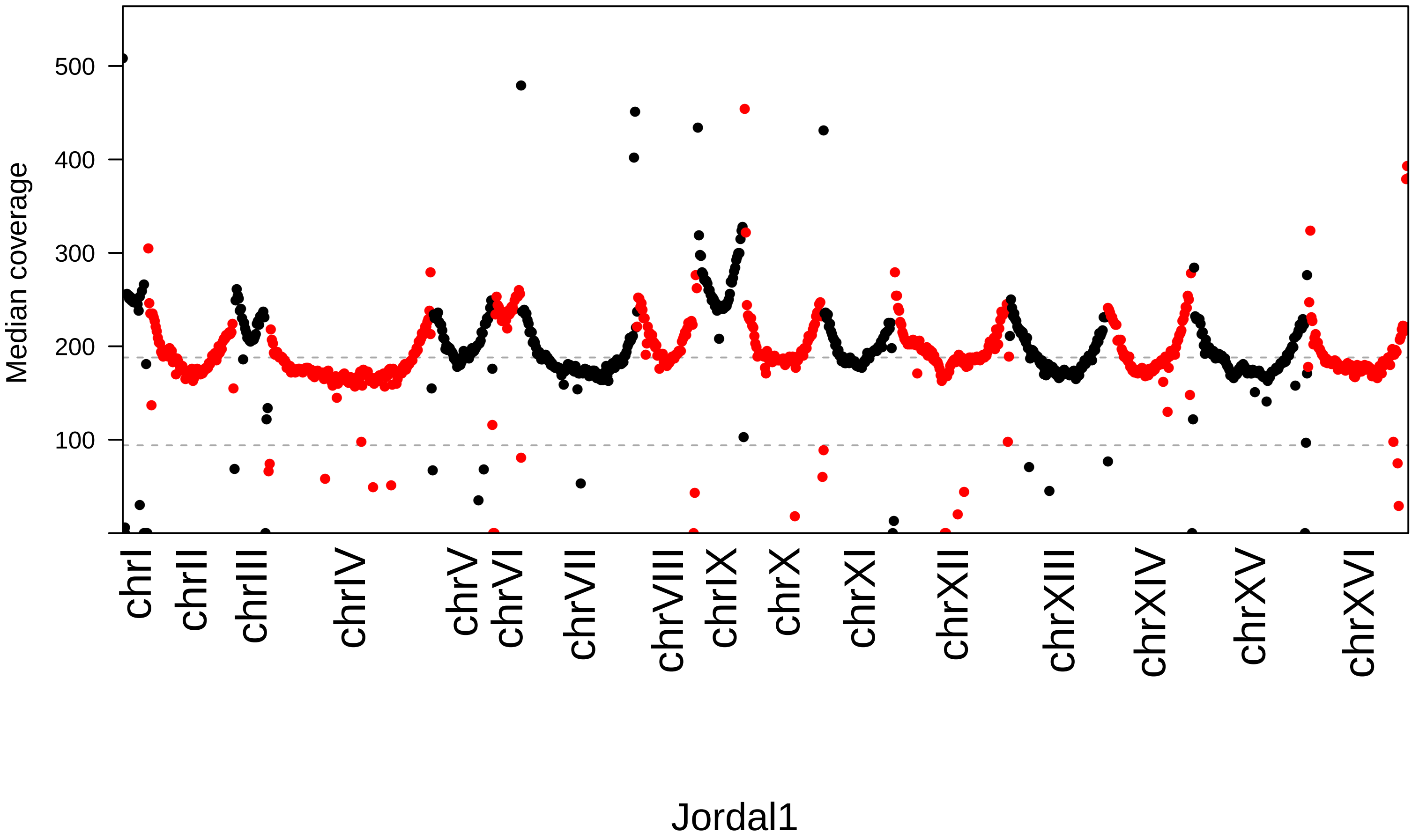

Supplementary Figure S2

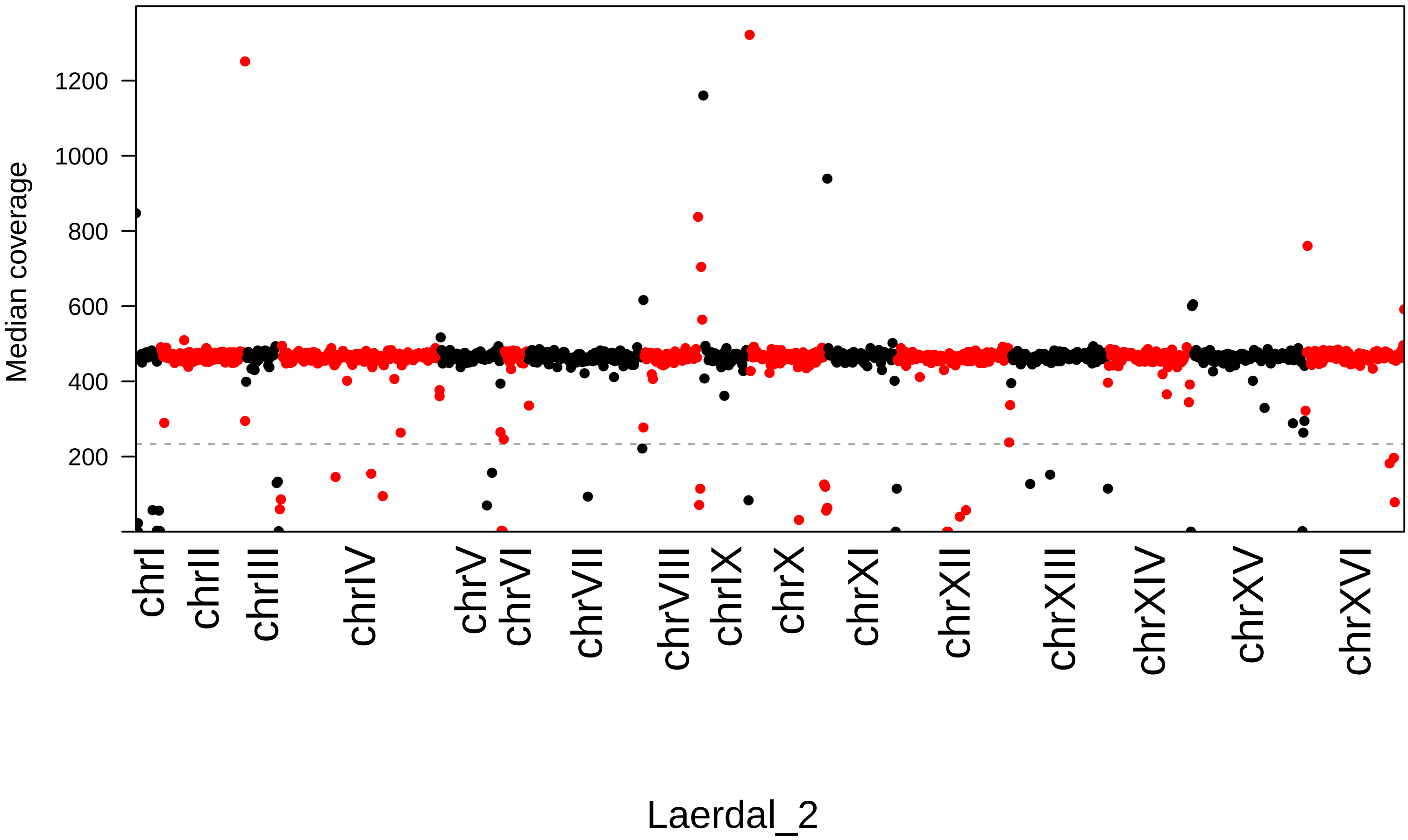

Supplementary Figure S2

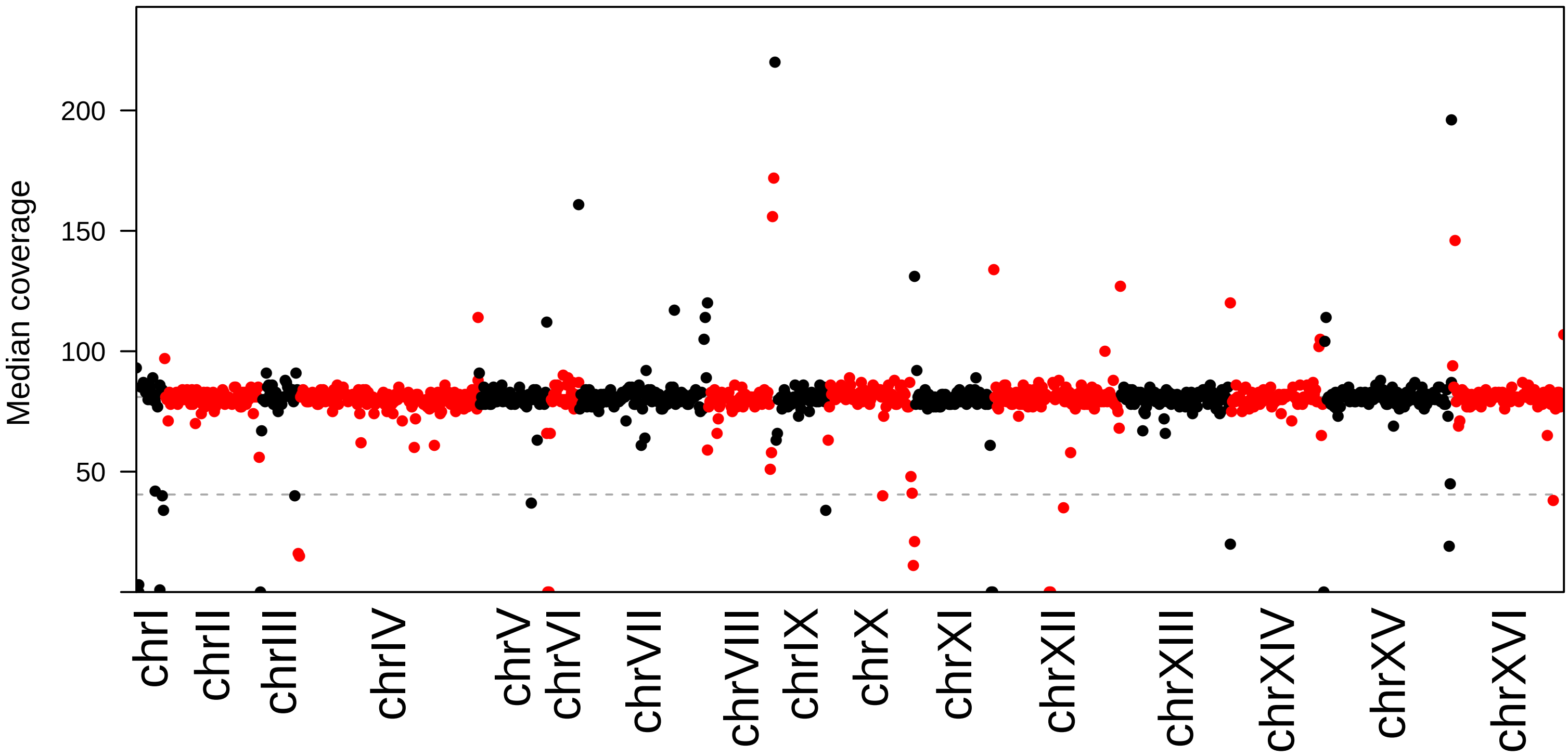

Lostegard_1

Supplementary Figure S2

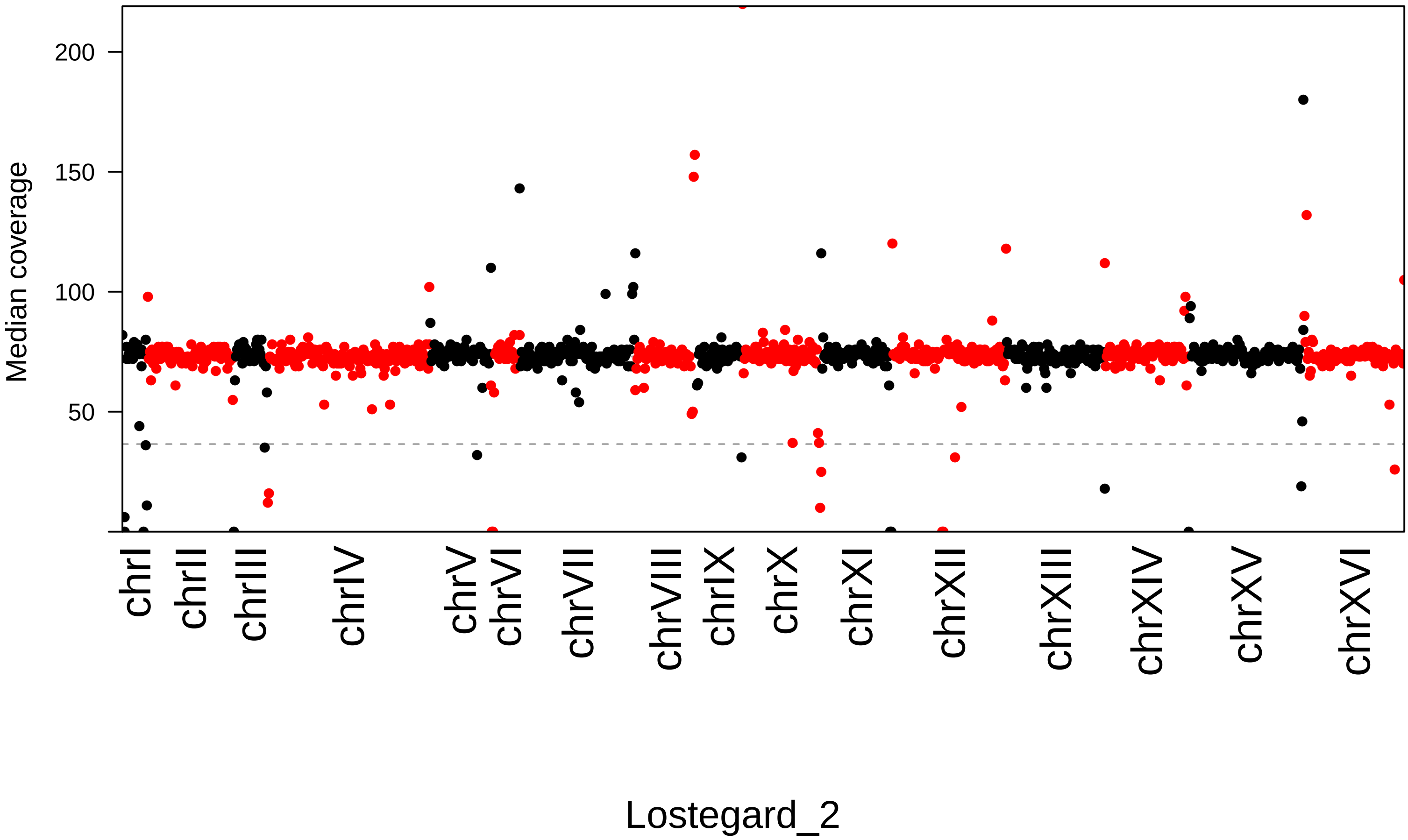

Supplementary Figure S2

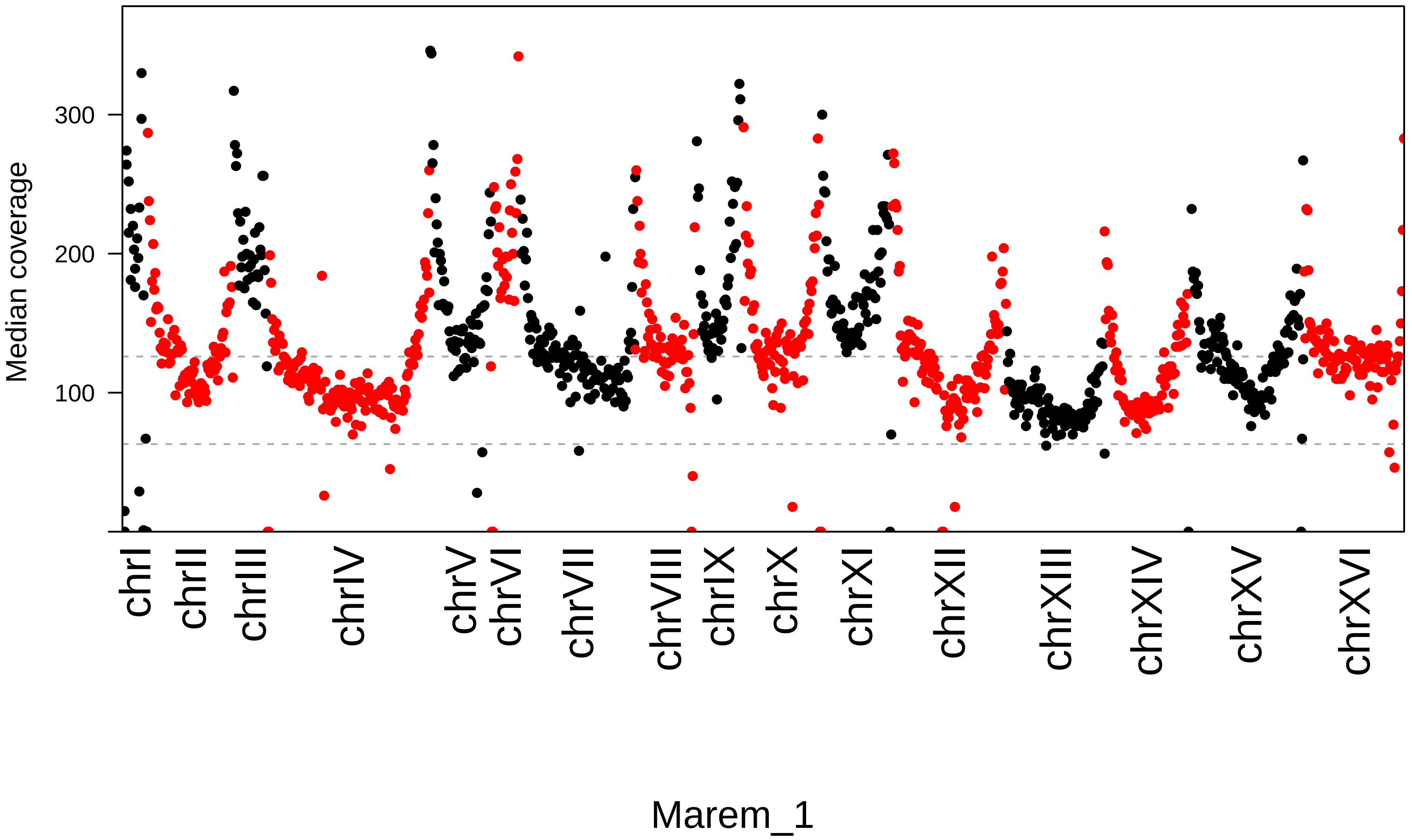

Supplementary Figure S2

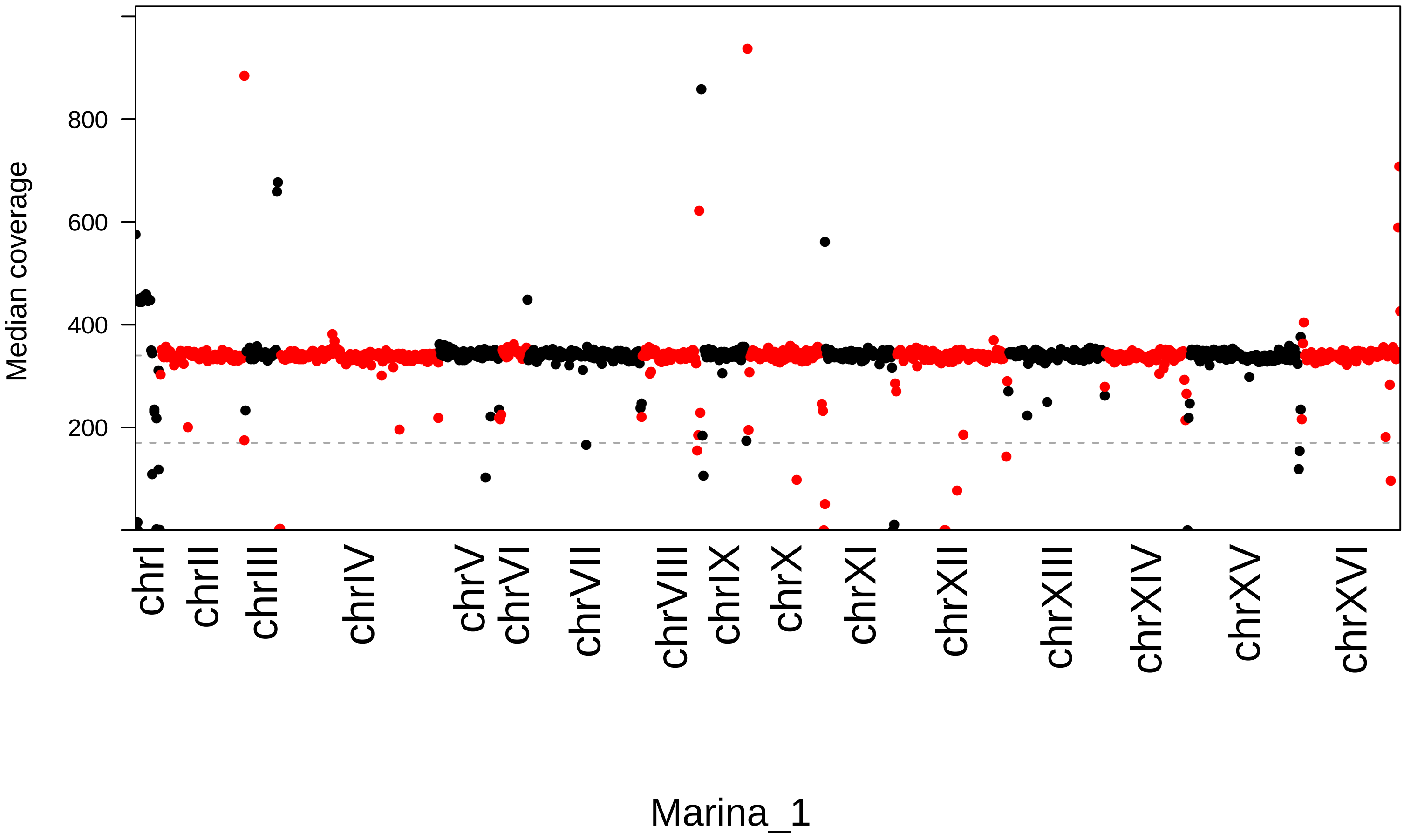

Supplementary Figure S2

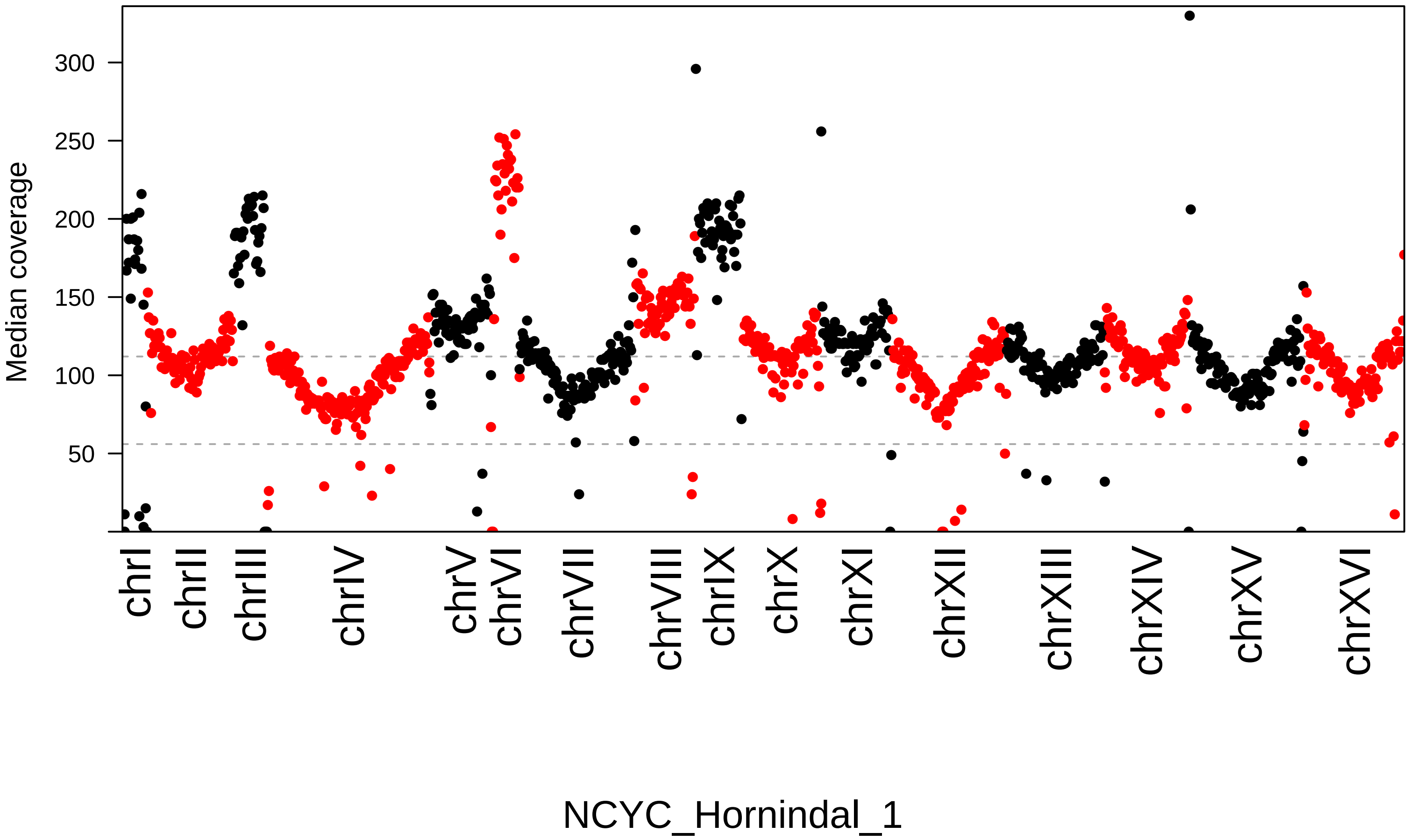

Supplementary Figure S2

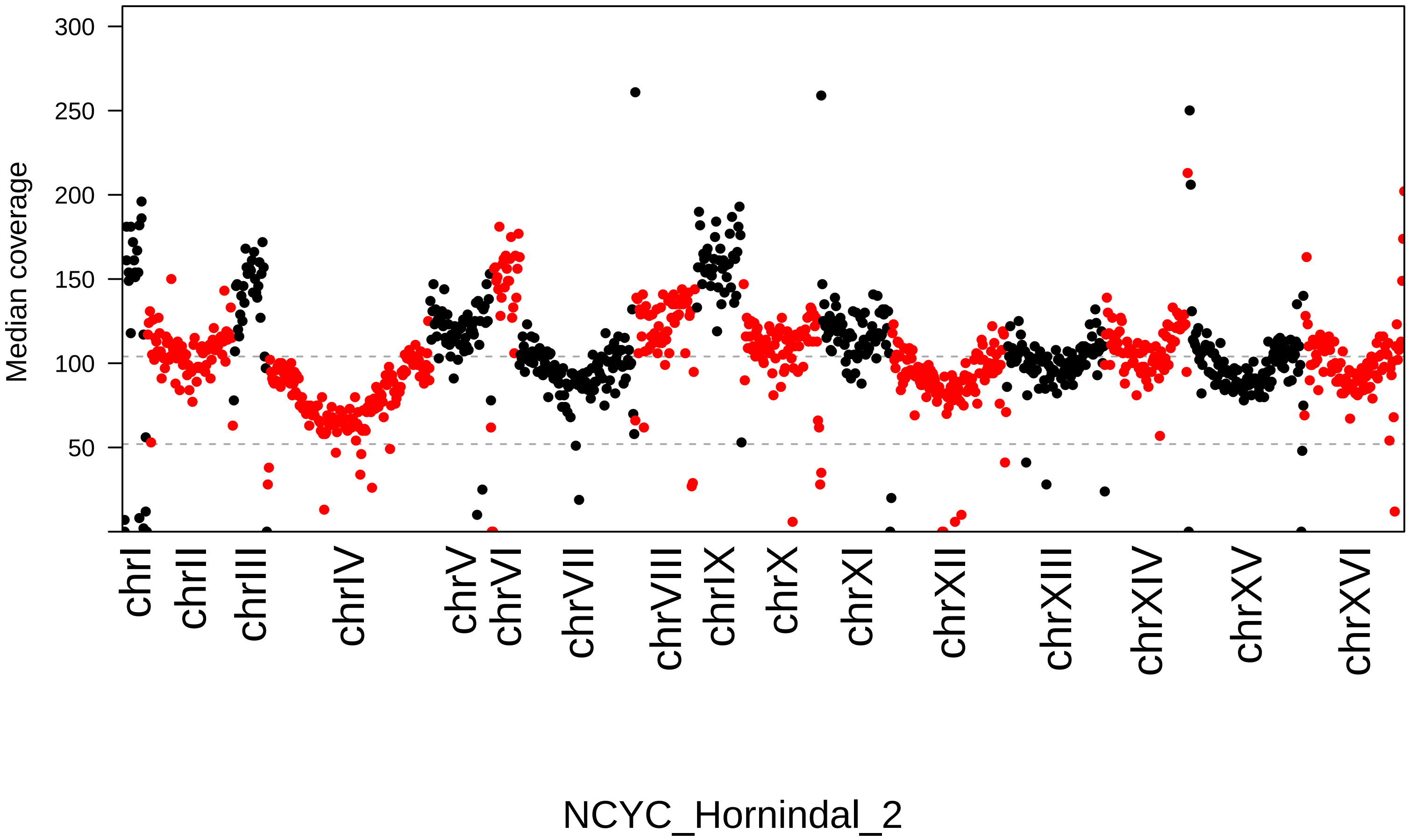

Supplementary Figure S2

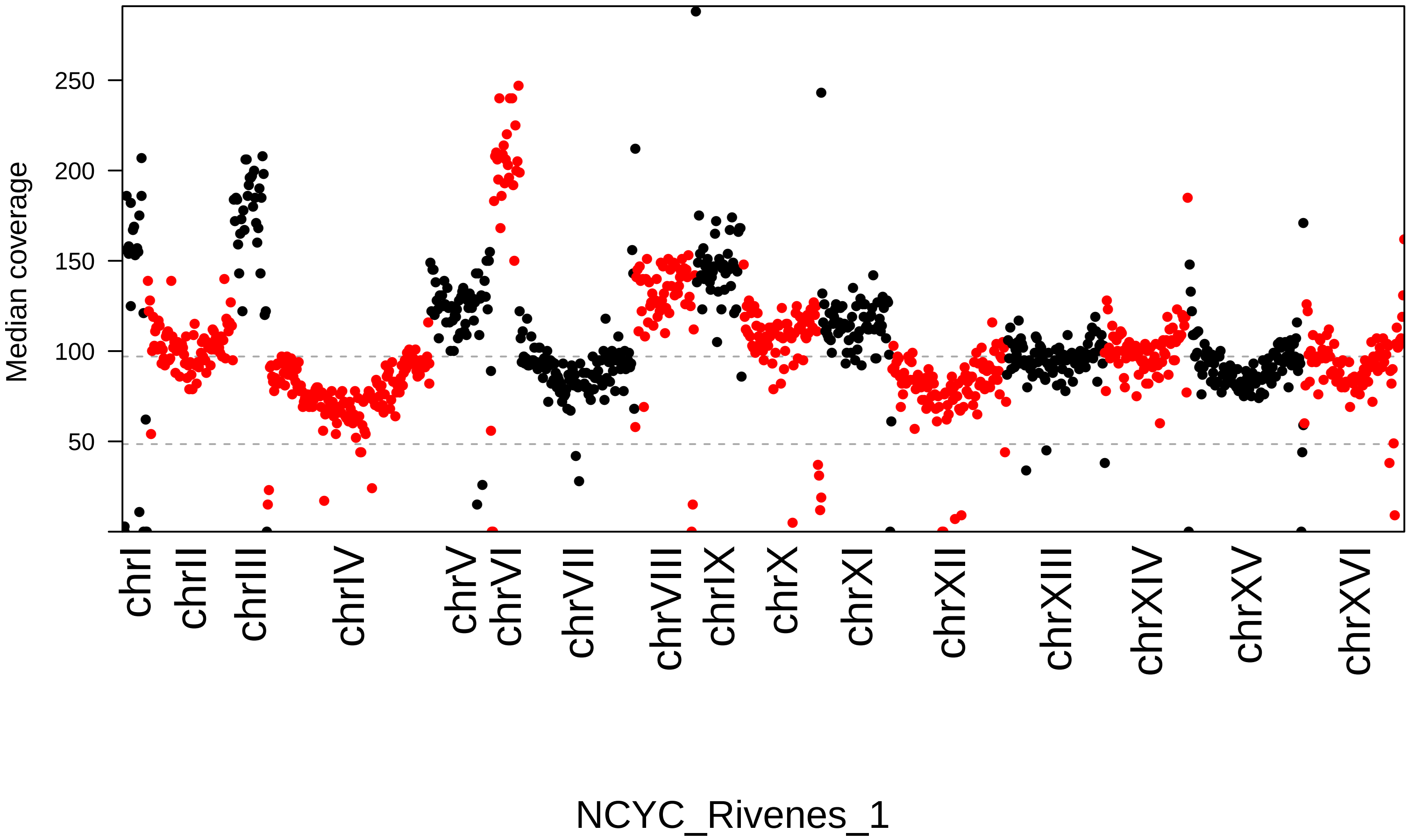

Supplementary Figure S2

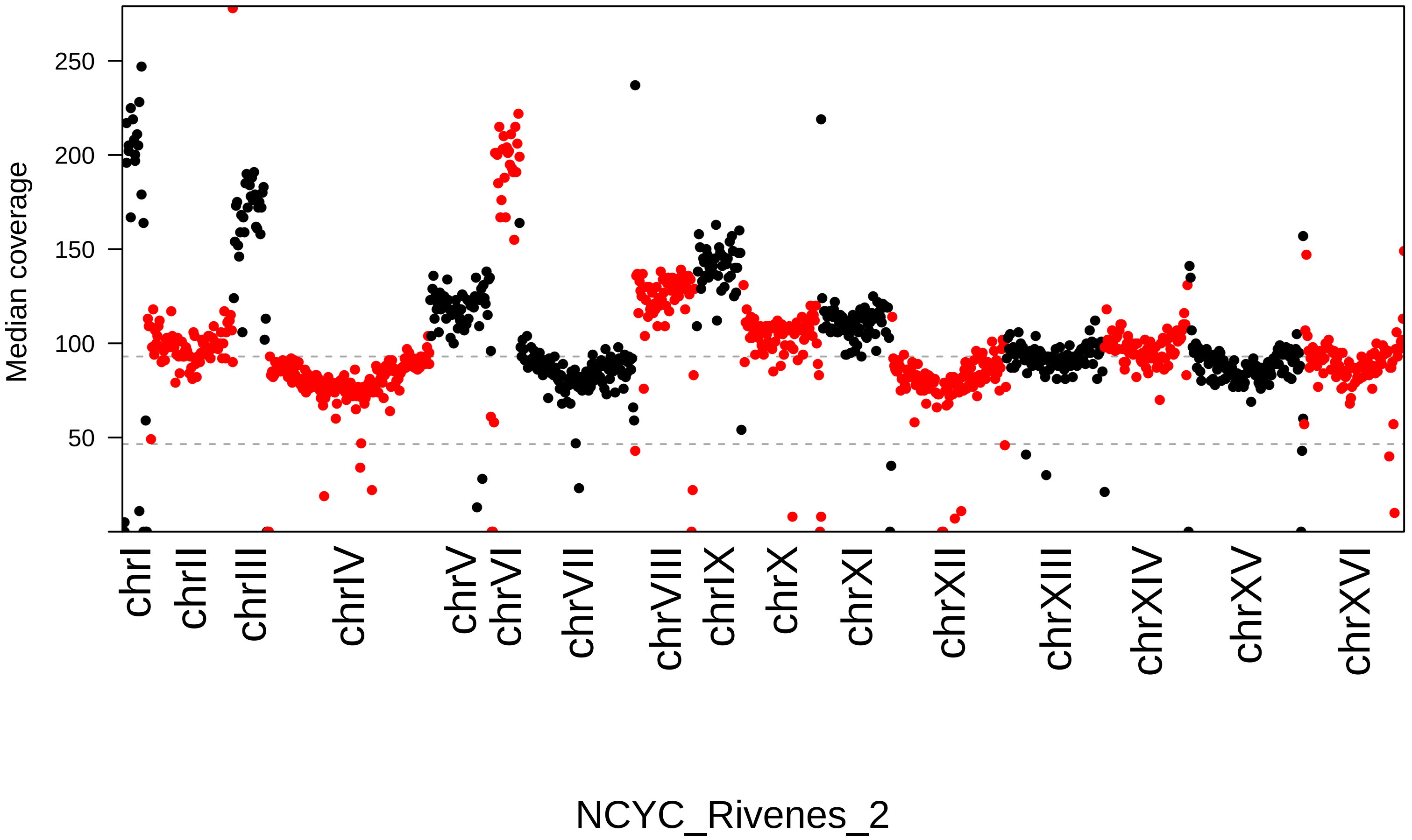

Supplementary Figure S2

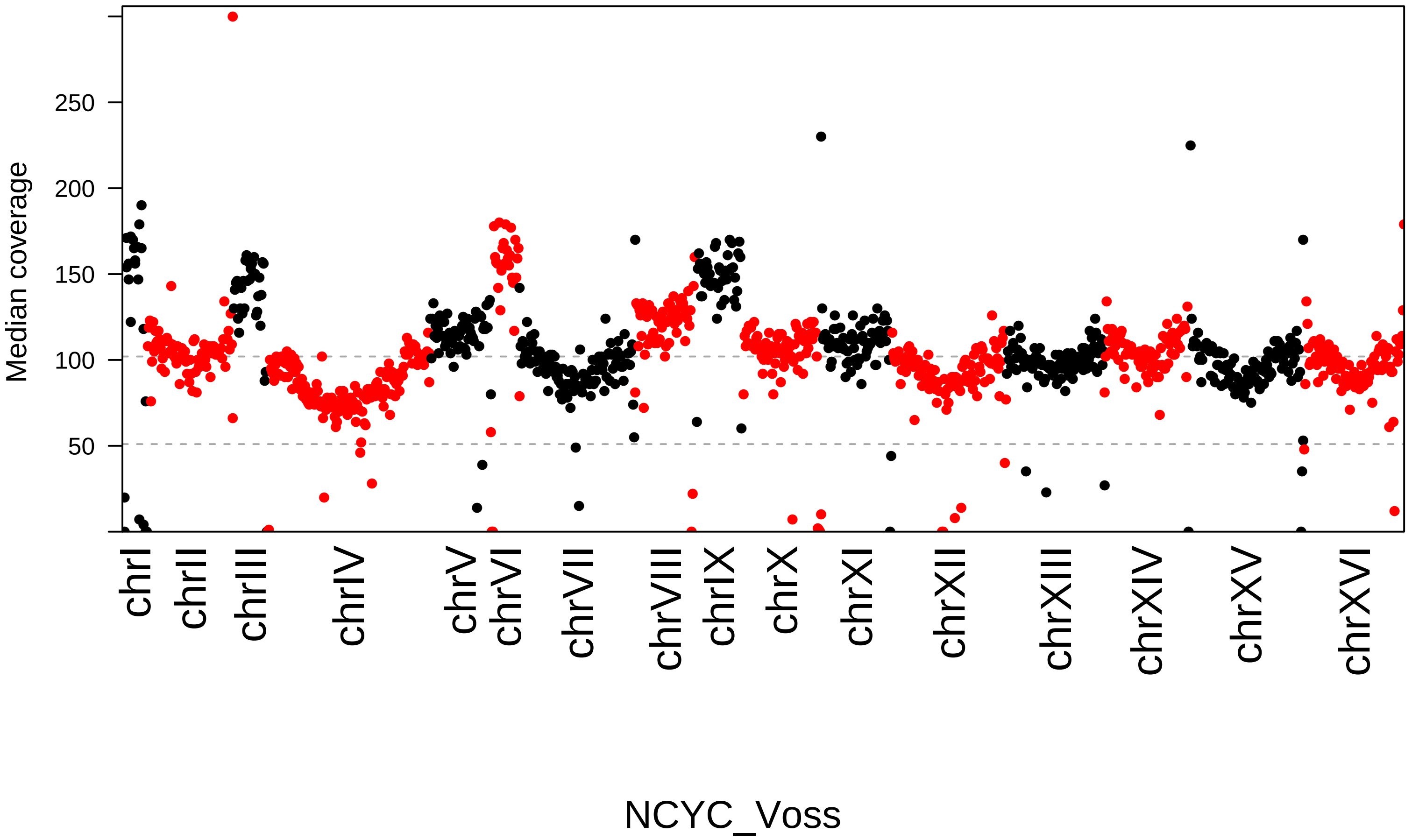

Supplementary Figure S2

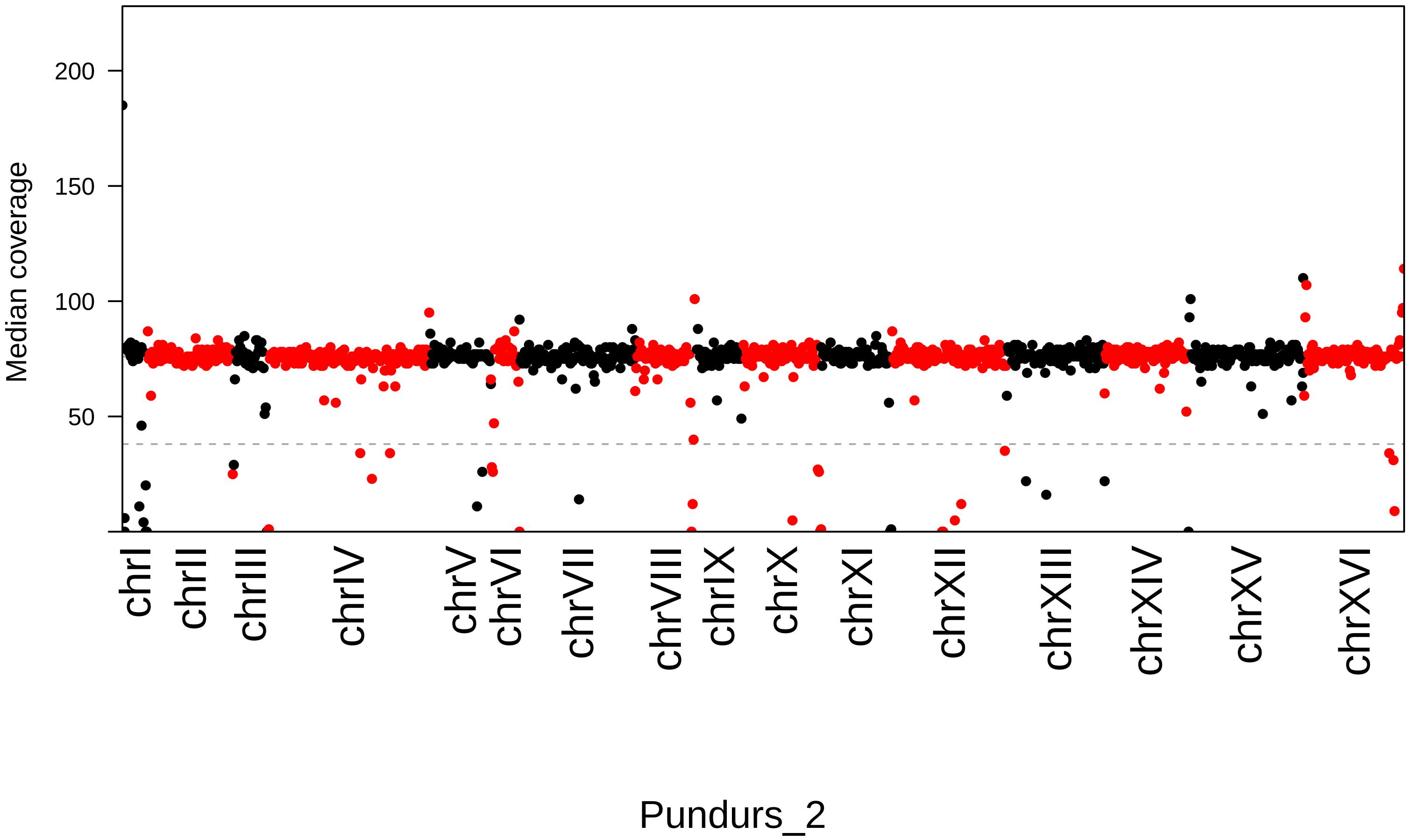

Supplementary Figure S2

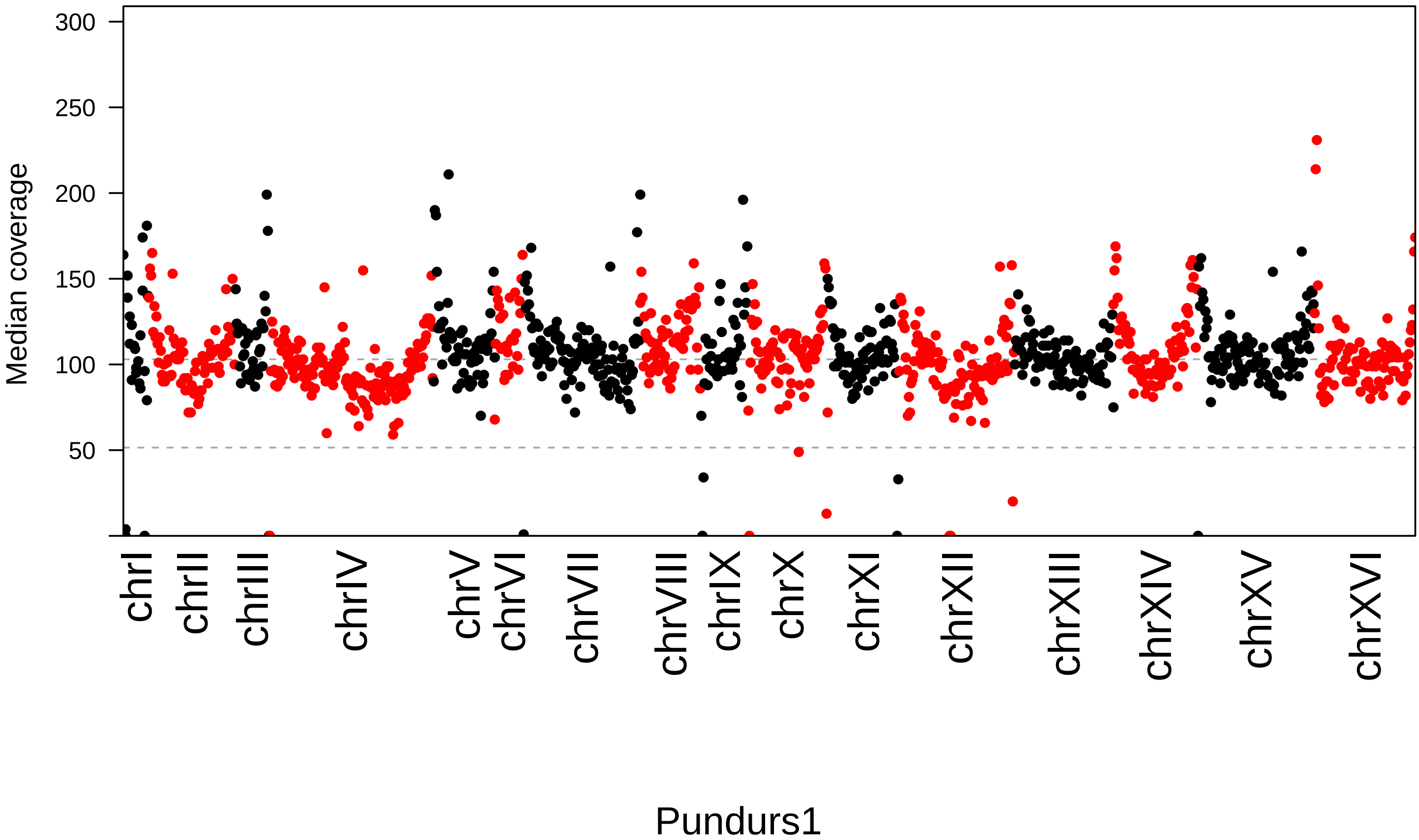

Supplementary Figure S2

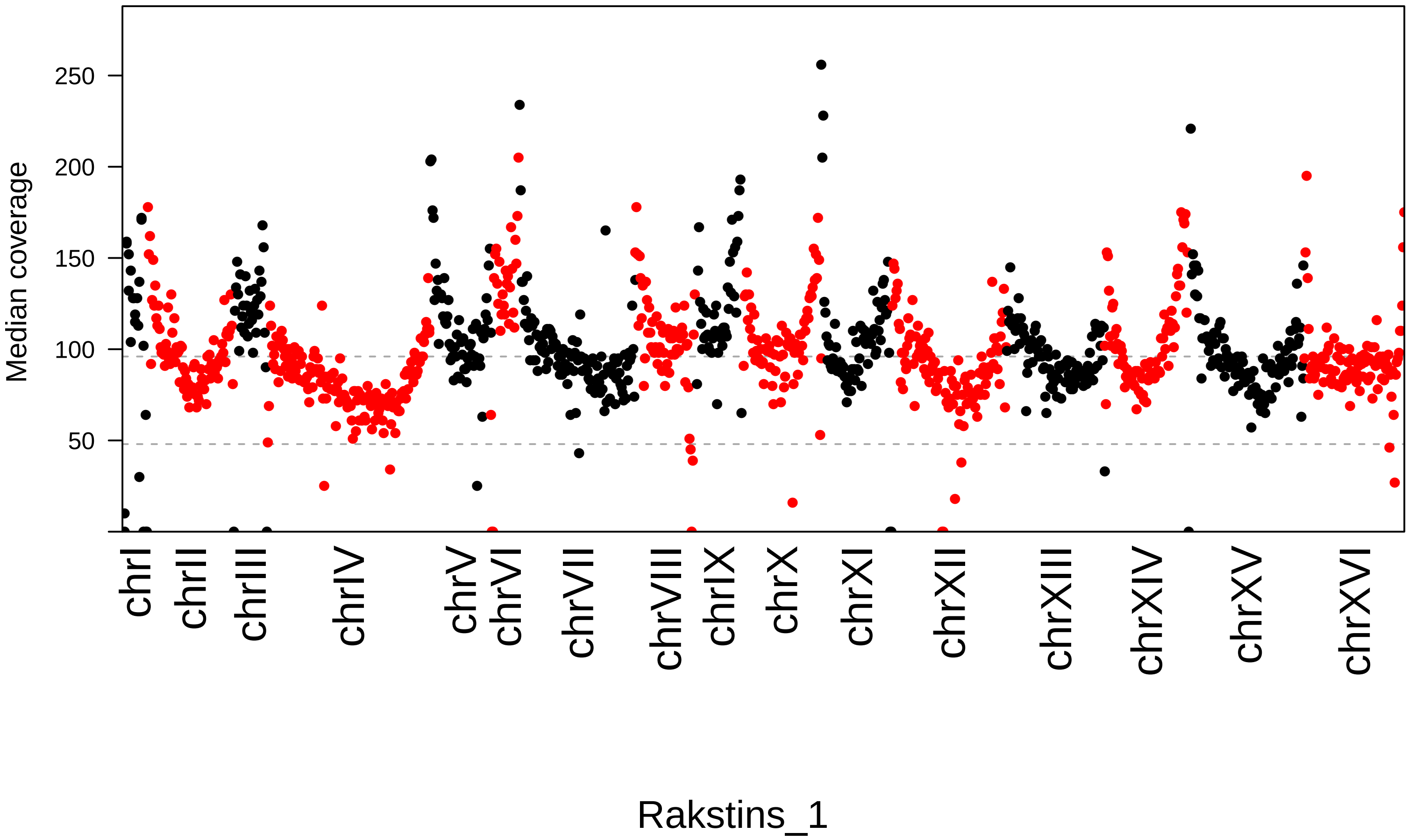

Supplementary Figure S2

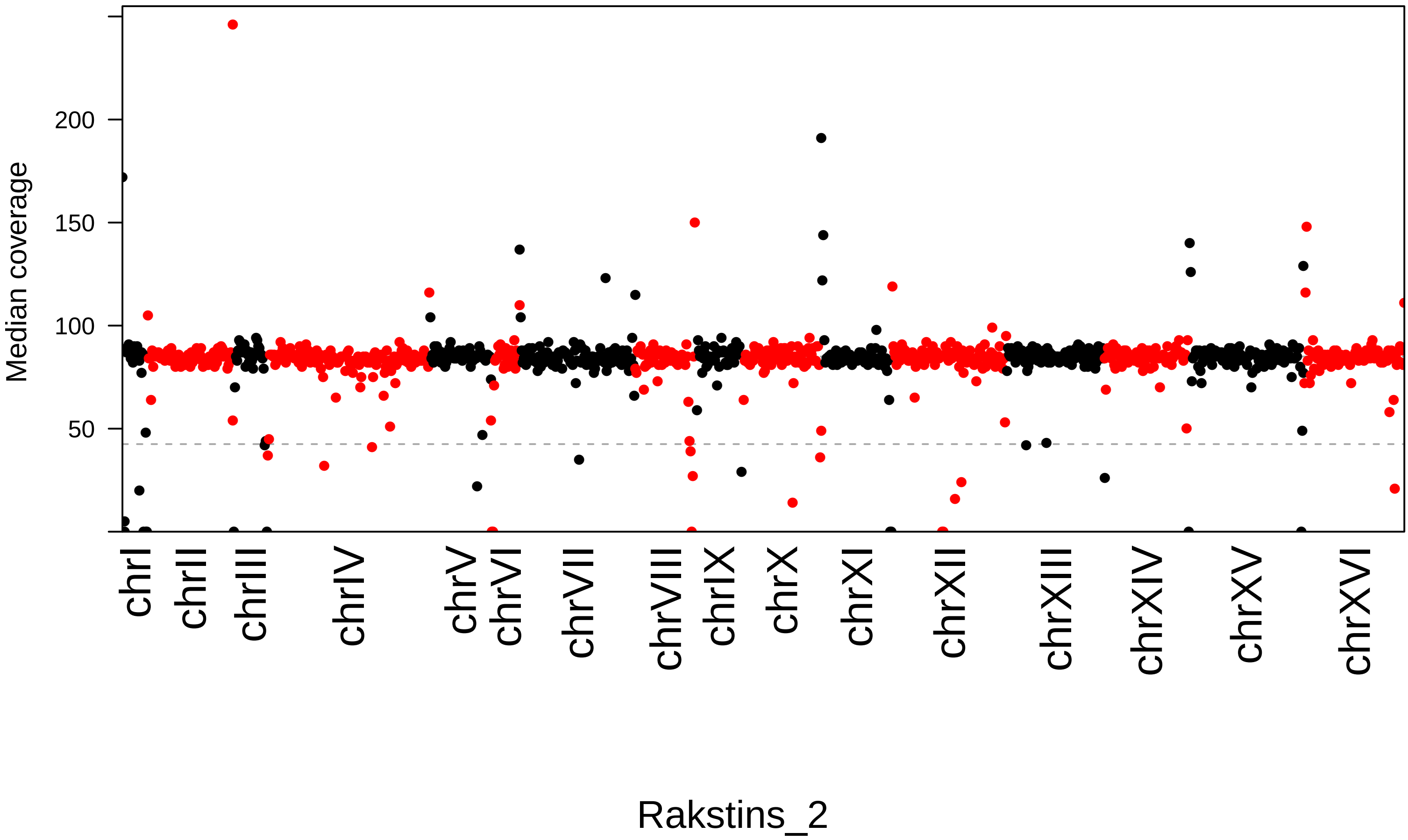

Supplementary Figure S2

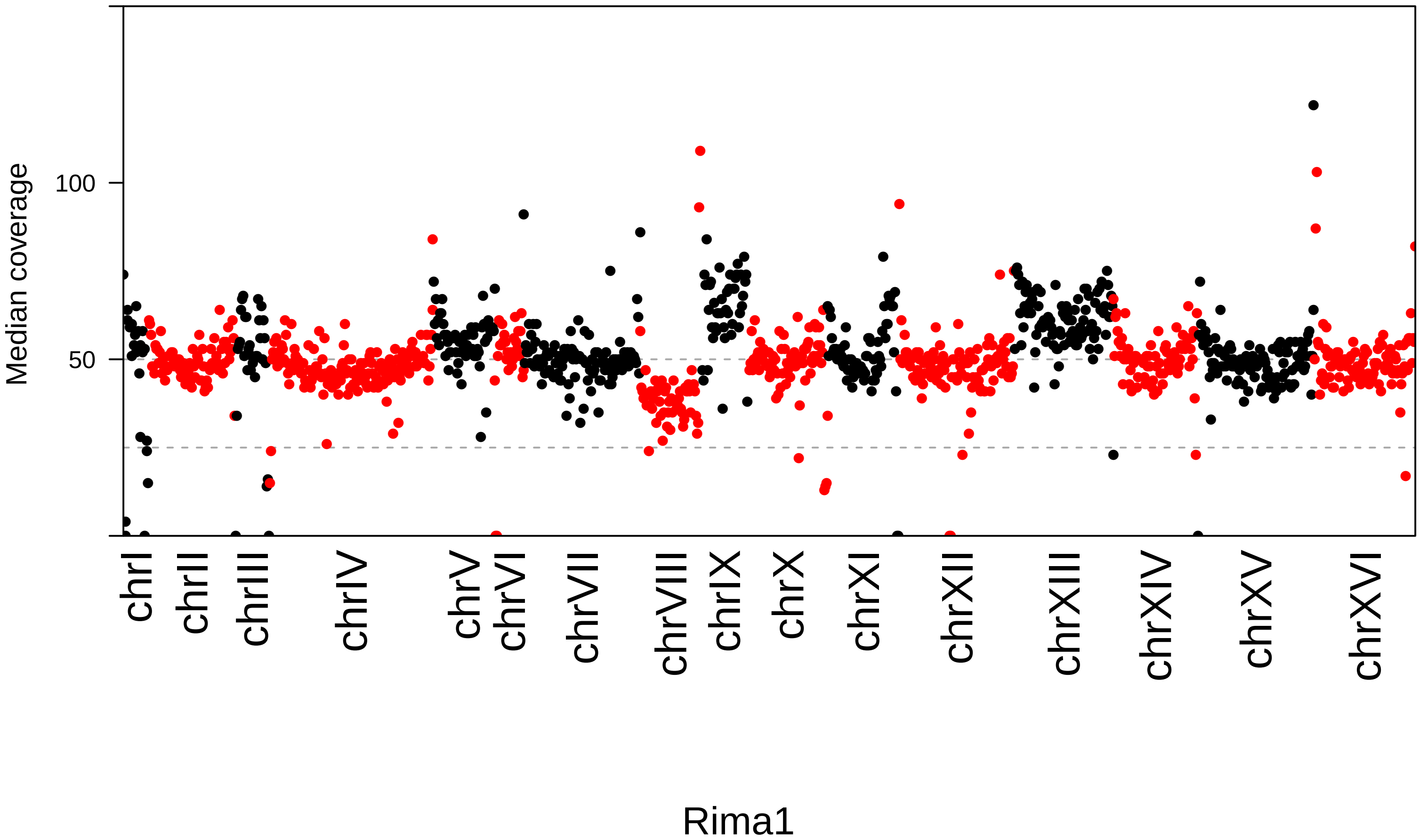

Supplementary Figure S2

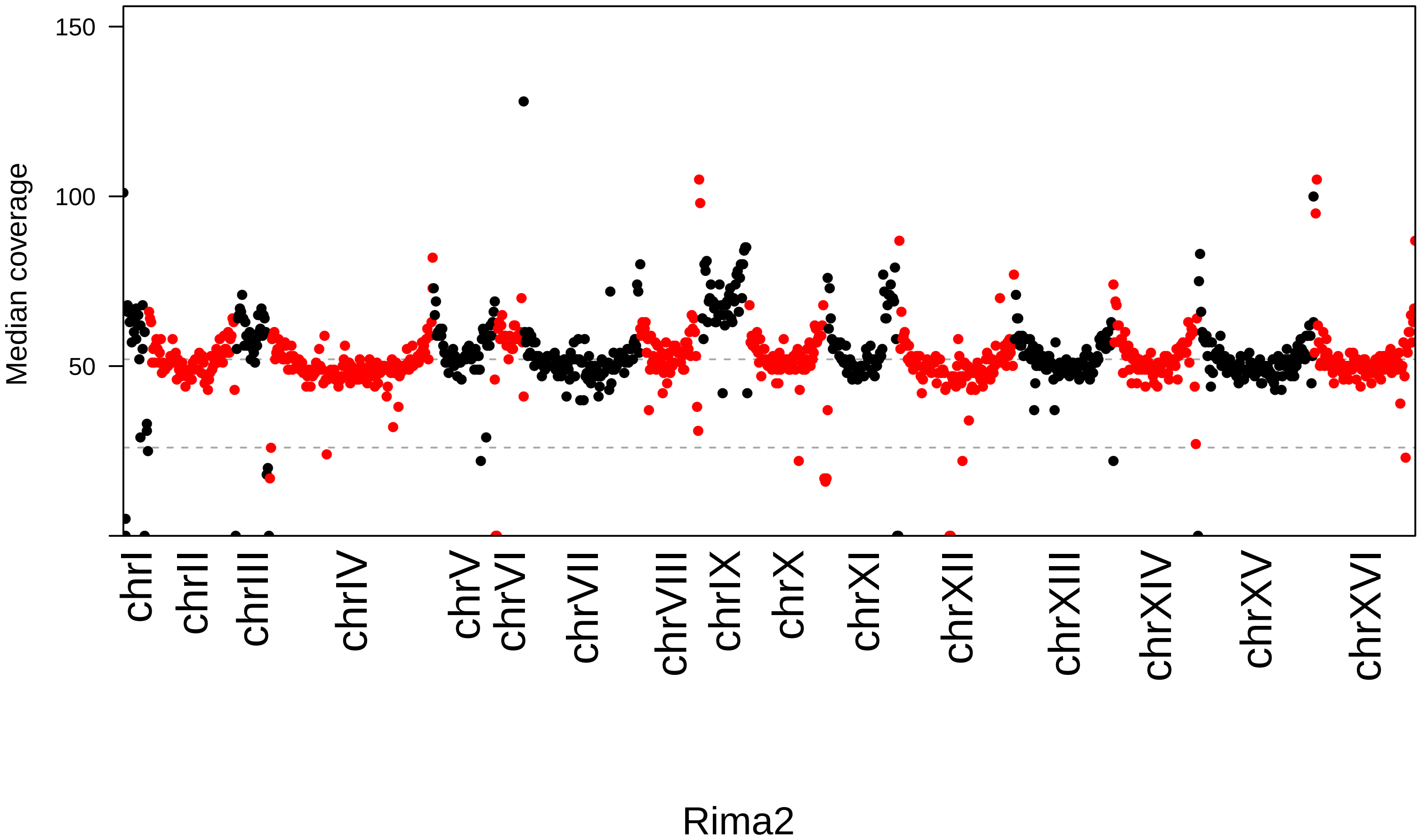

Supplementary Figure S2

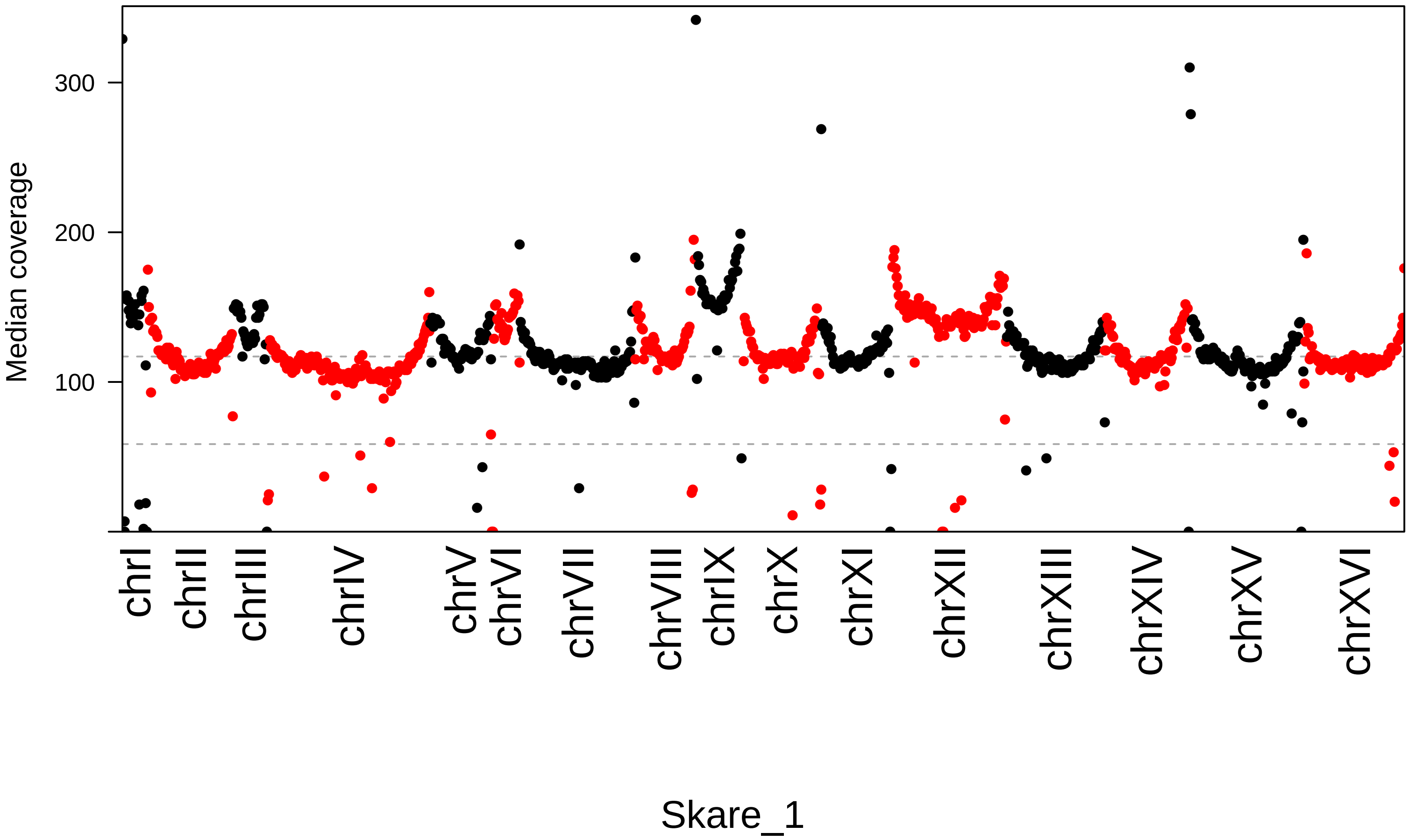

Supplementary Figure S2

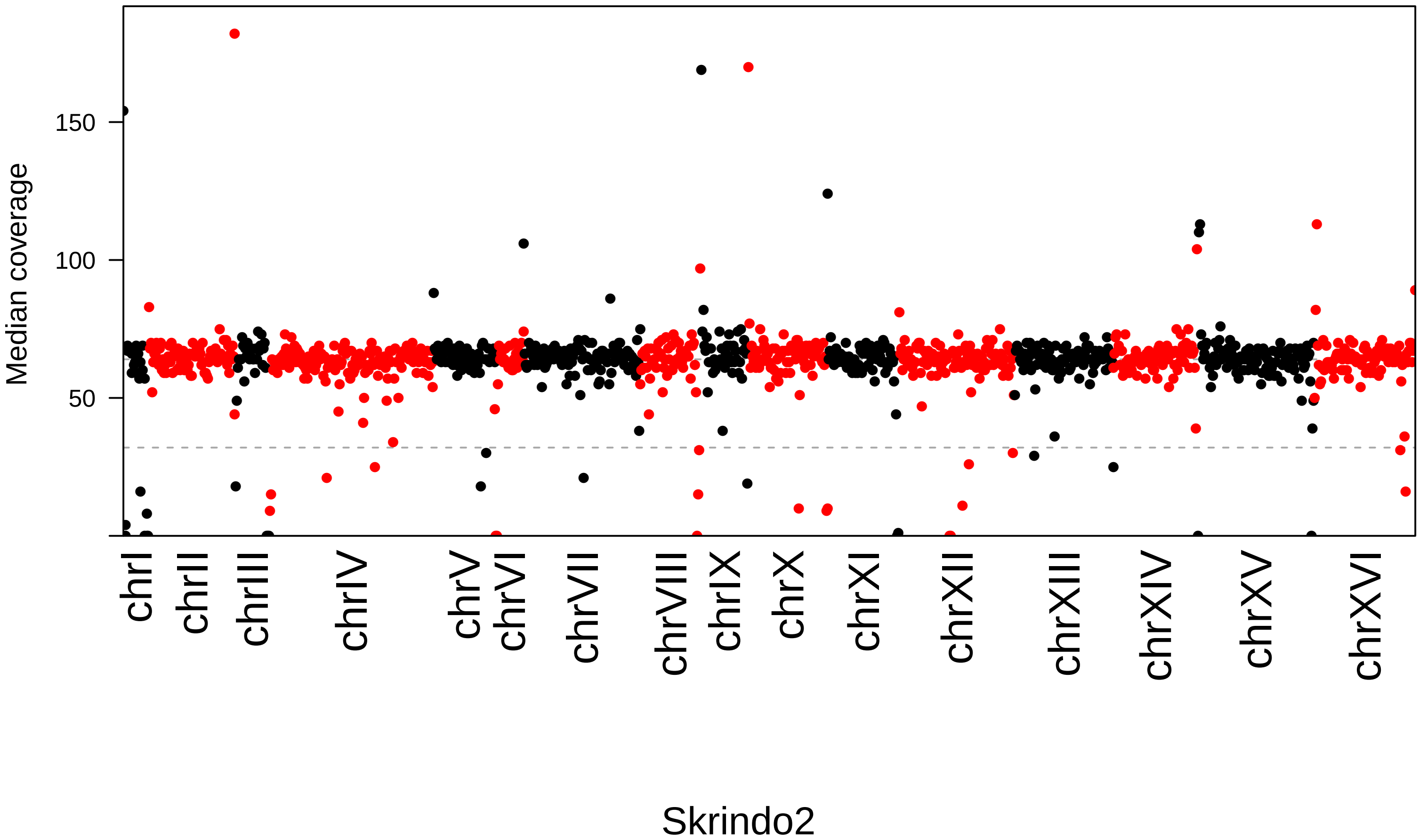

Supplementary Figure S2

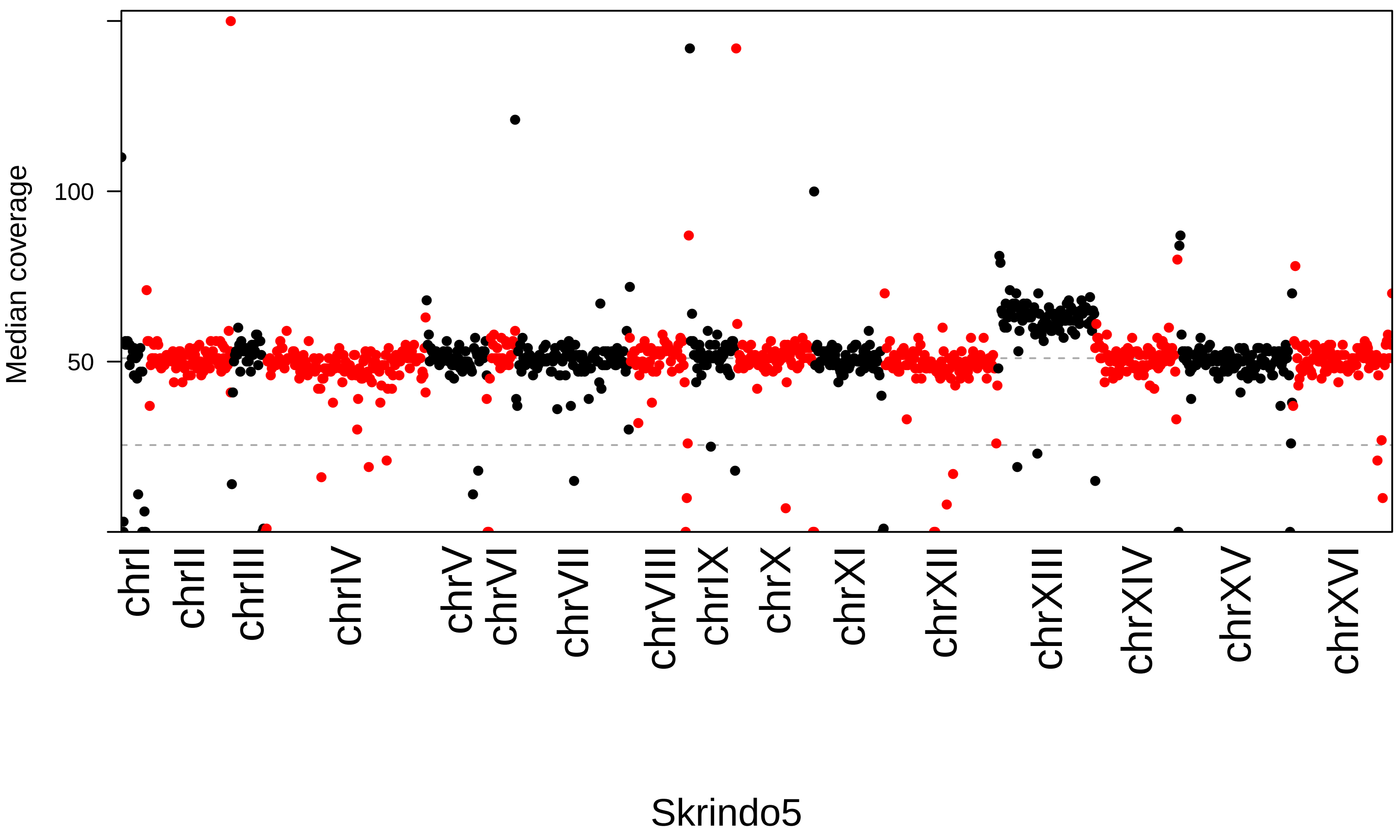

Supplementary Figure S2

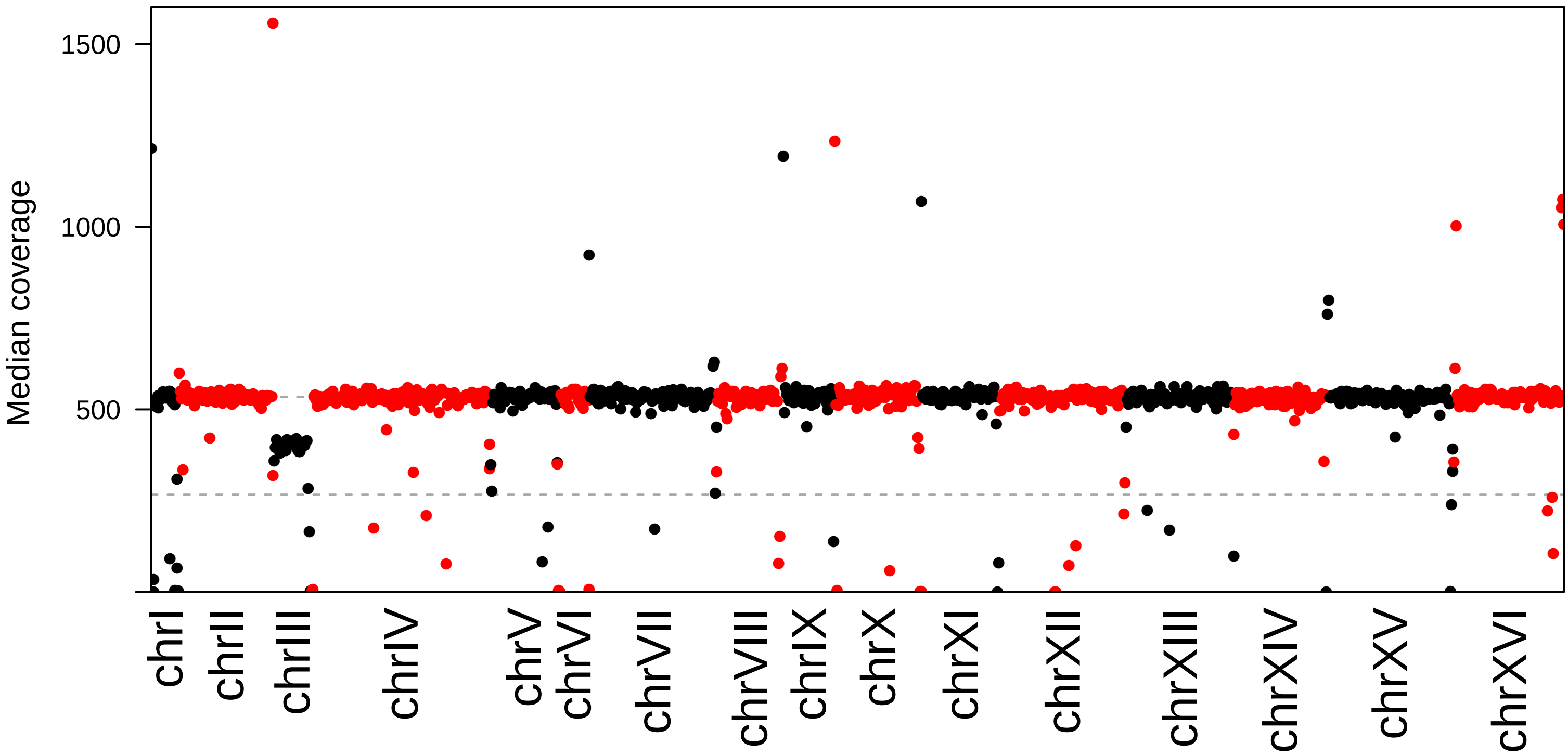

Stordal_Ebbegarden_1

Supplementary Figure S2

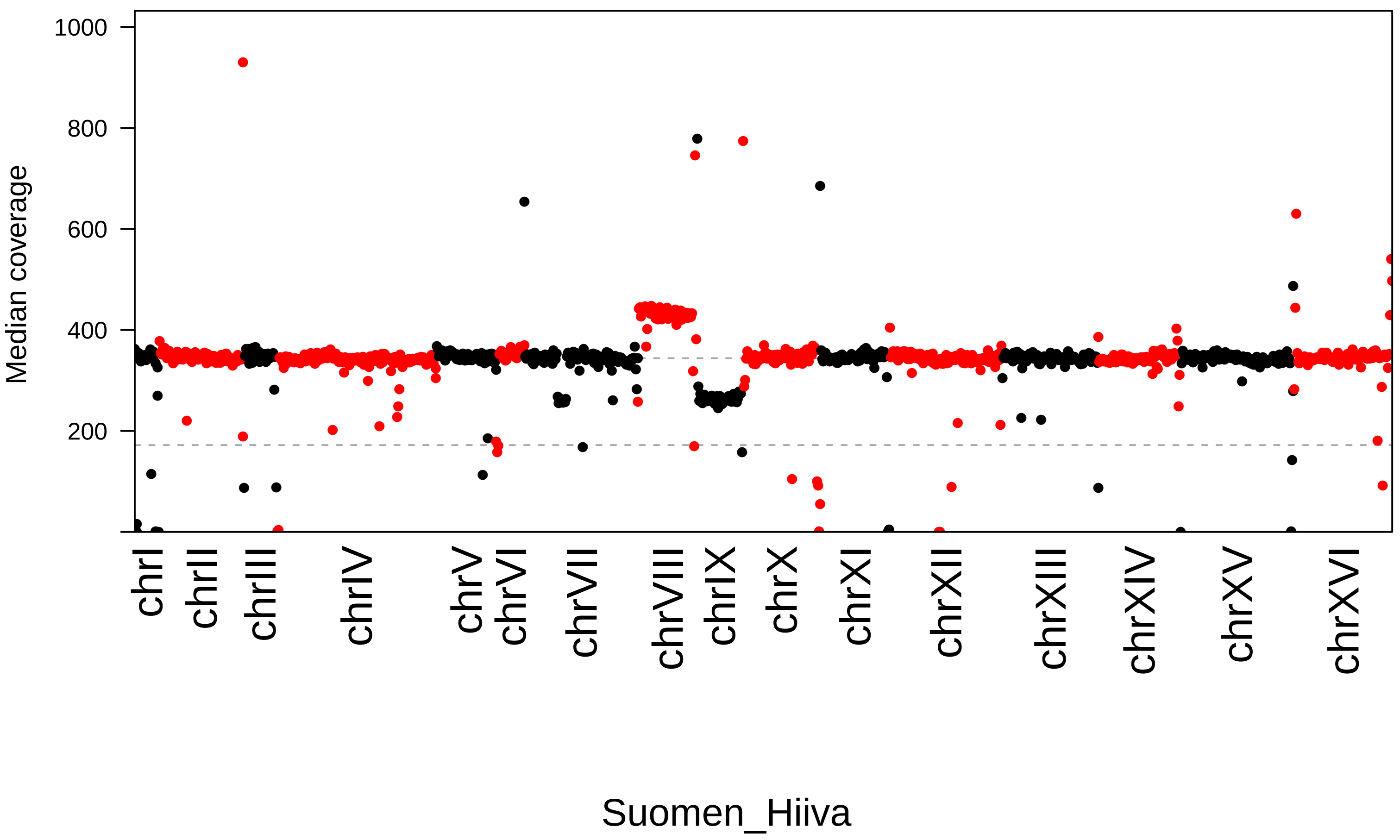

Supplementary Figure S2

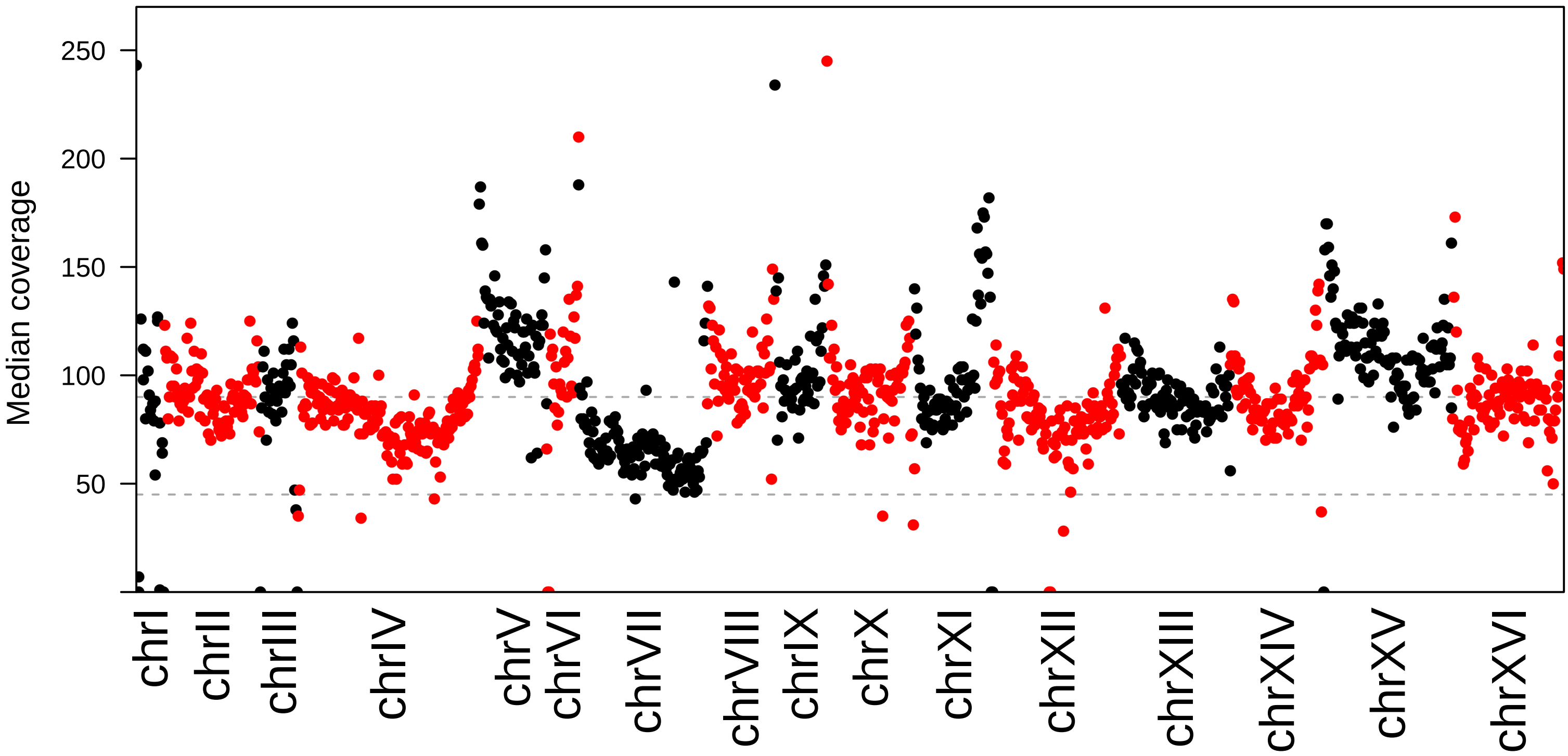

Tinn_1

Supplementary Figure S2

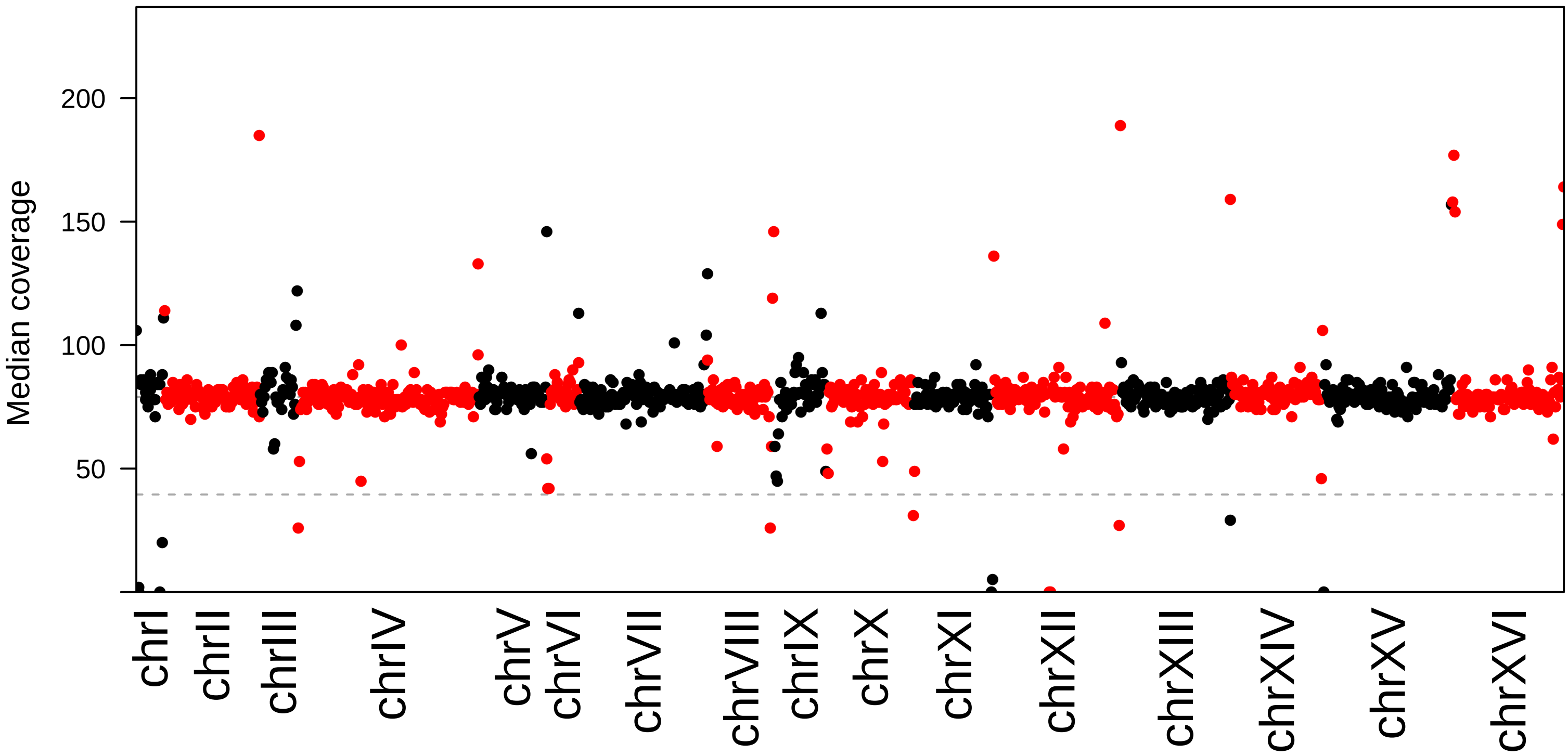

Tinn_2

Supplementary Figure S2

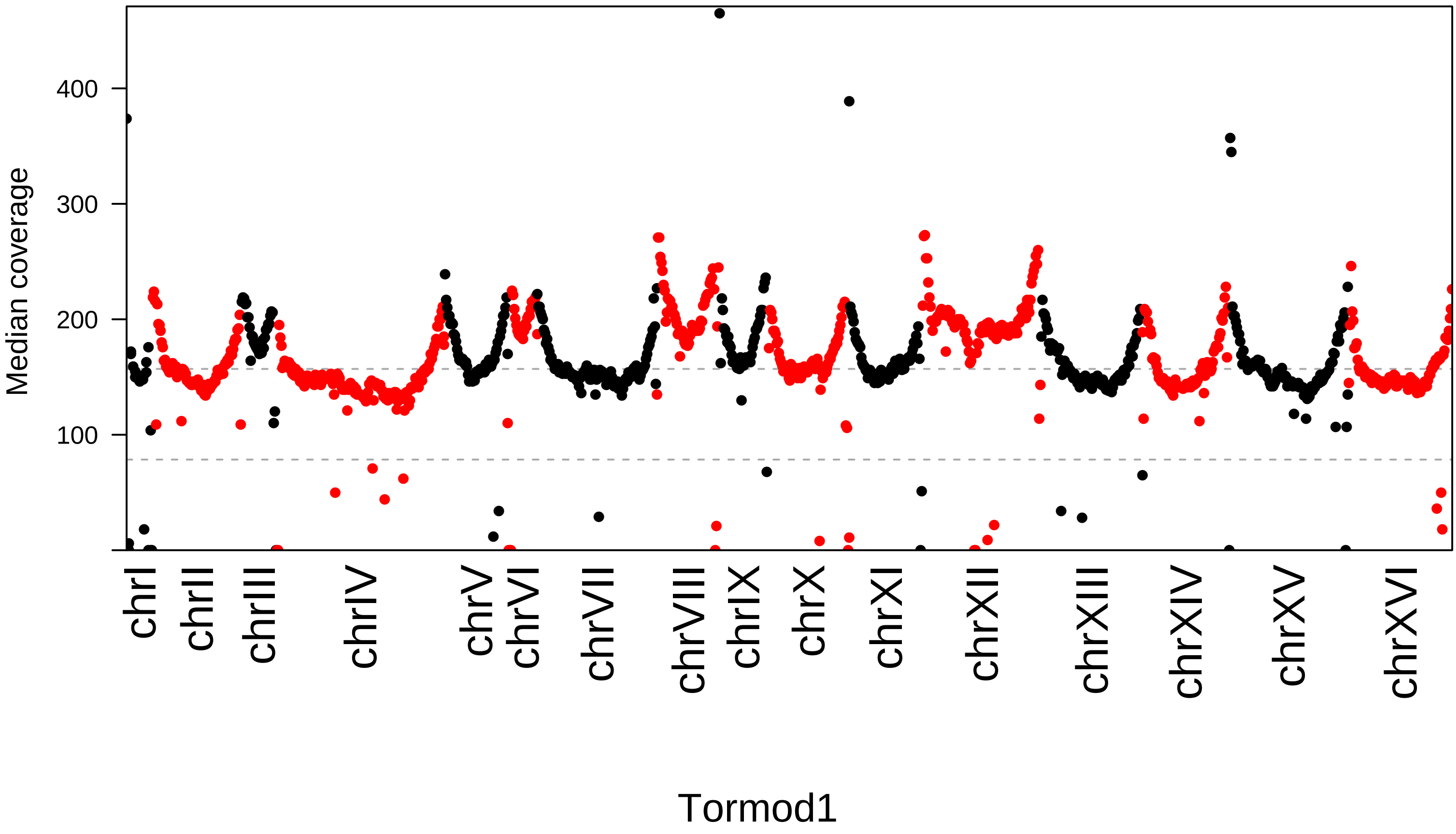

Supplementary Figure S2

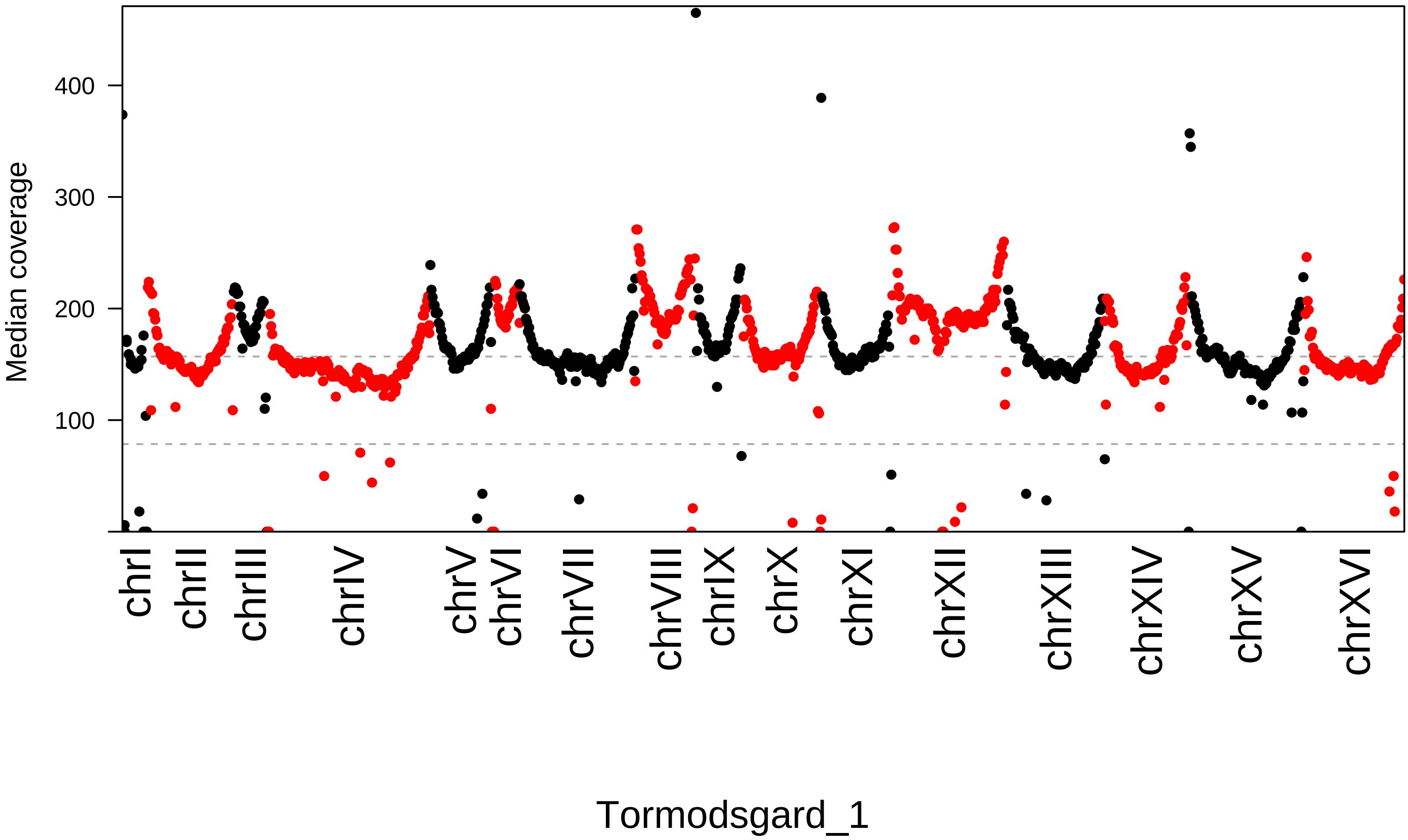

Supplementary Figure S2

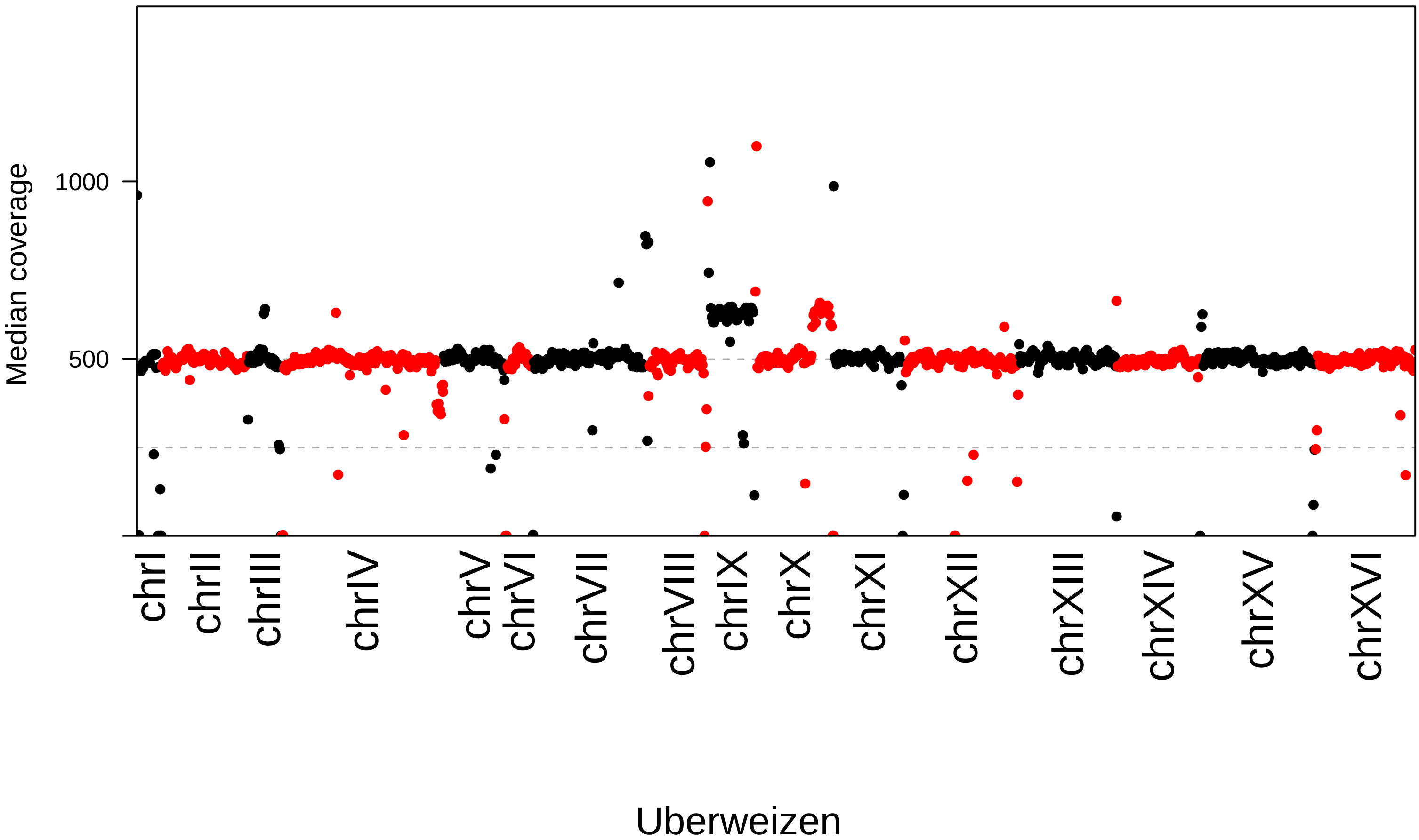

Supplementary Figure S2

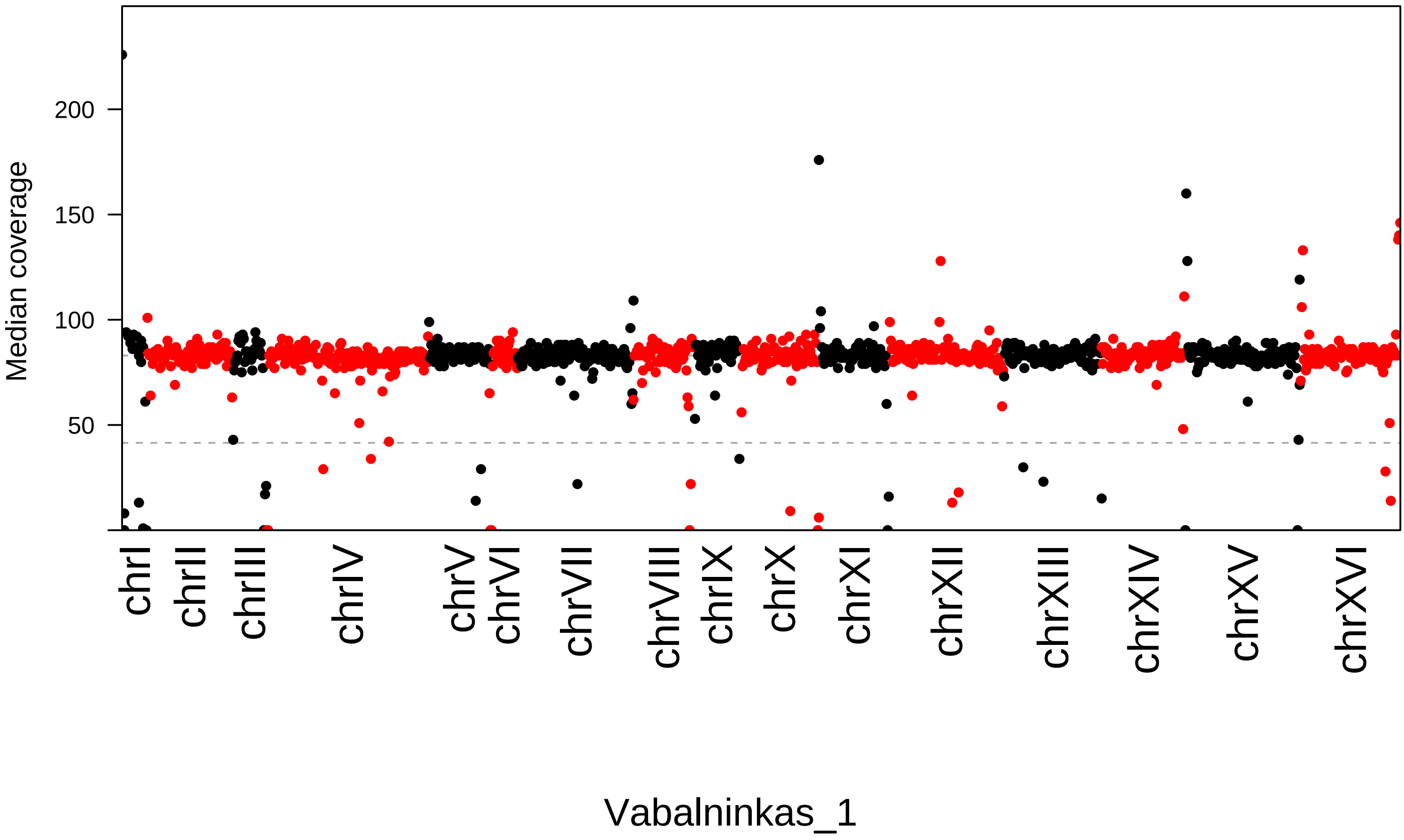

Supplementary Figure S2

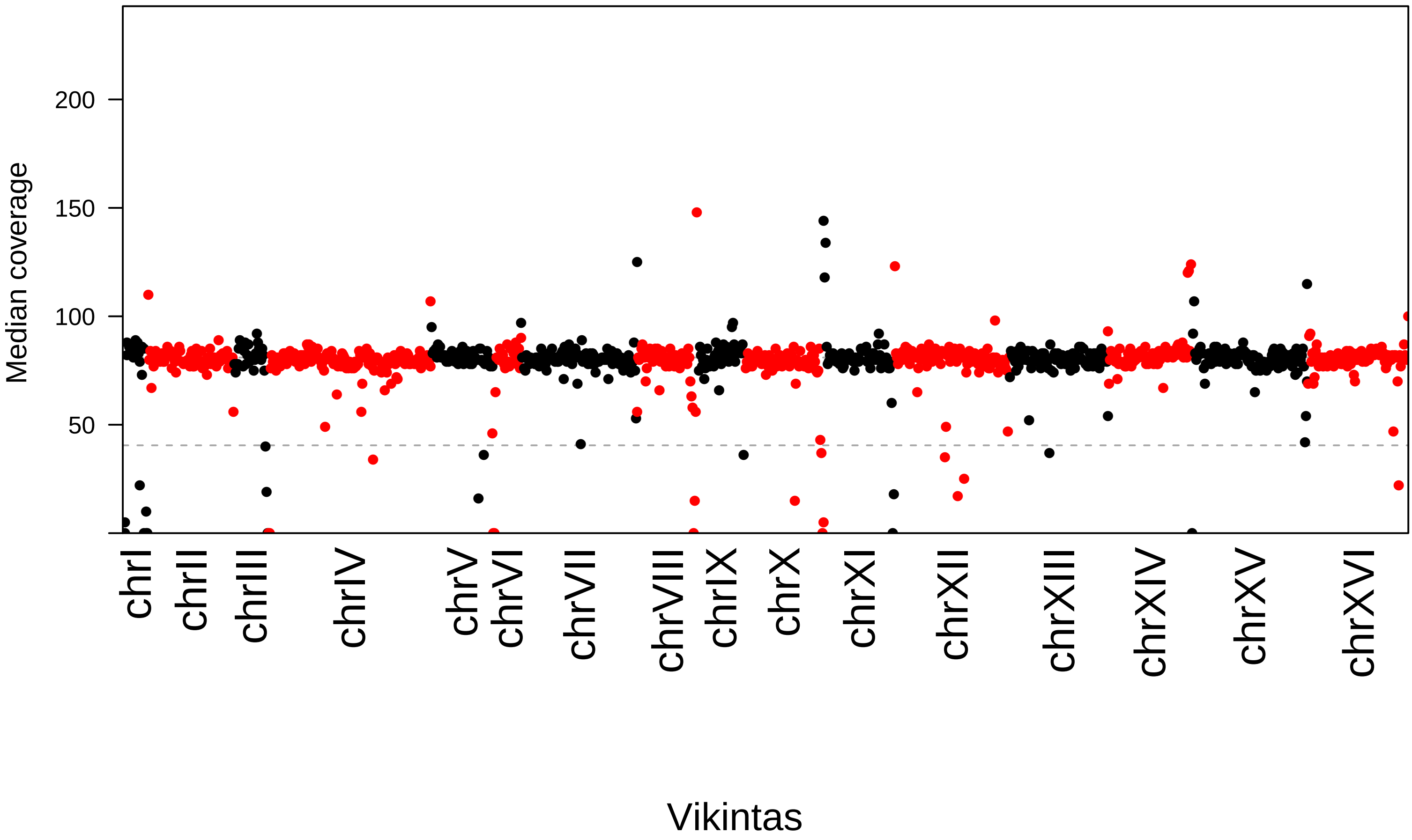

Supplementary Figure S2

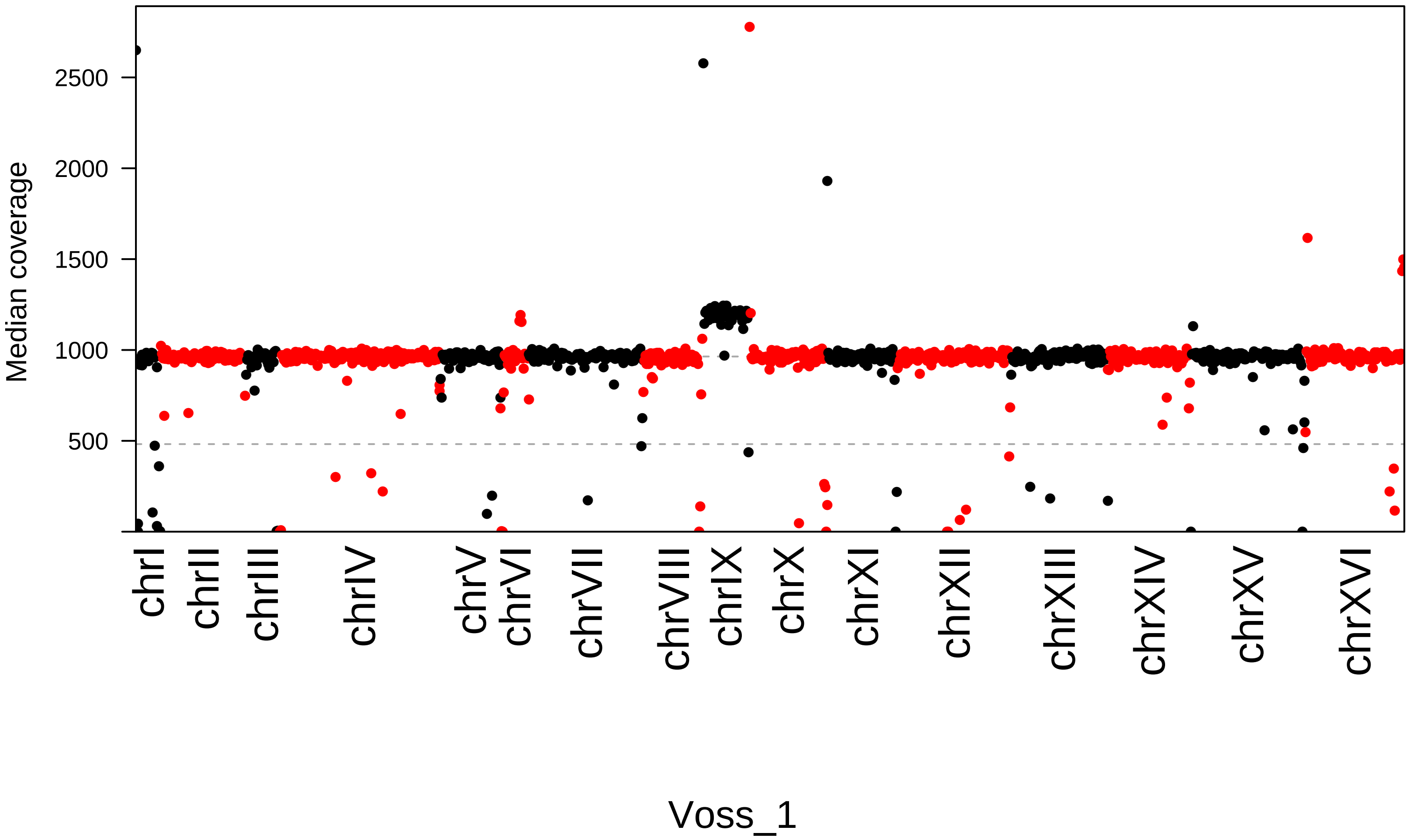

Supplementary Figure S2

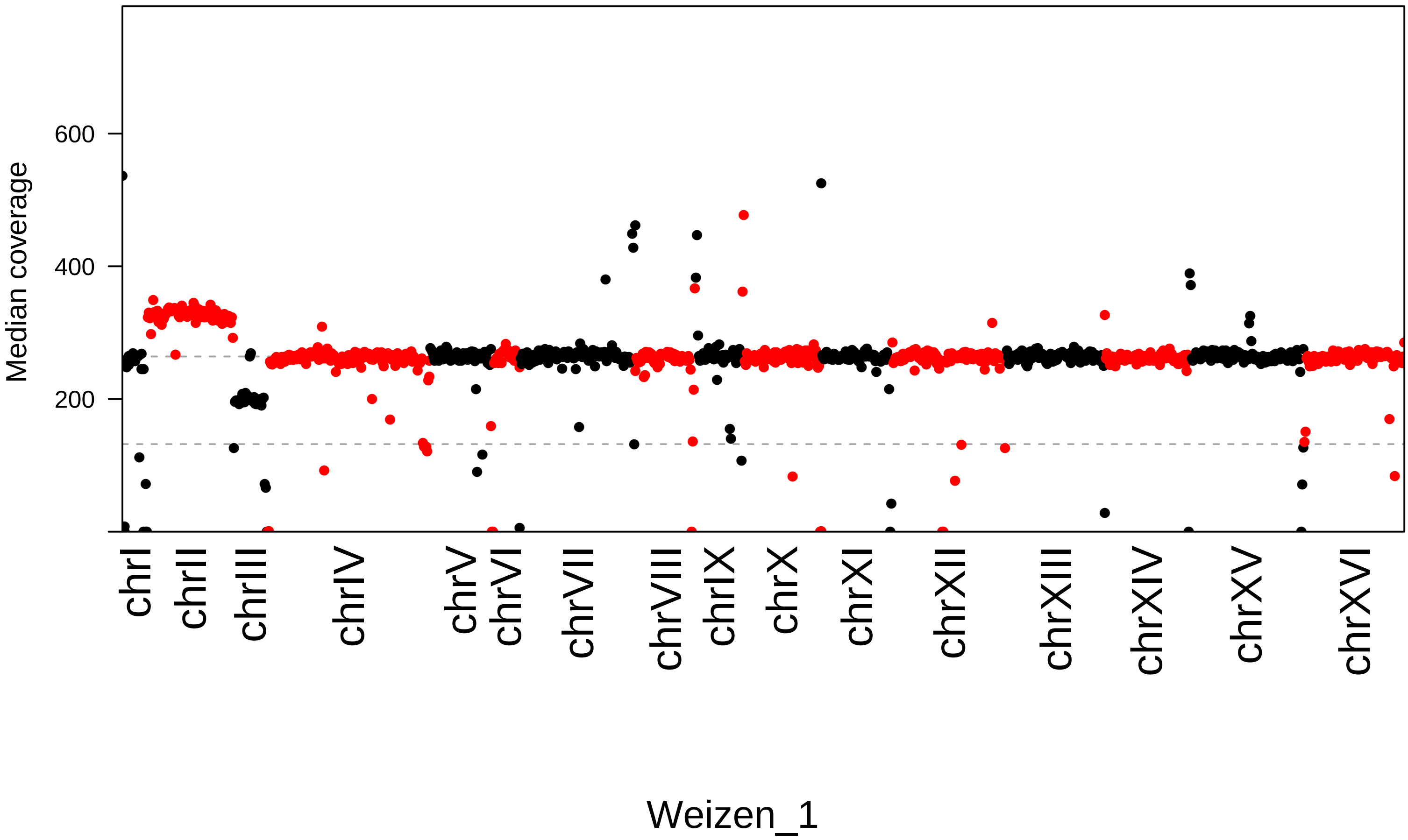

Supplementary Figure S2

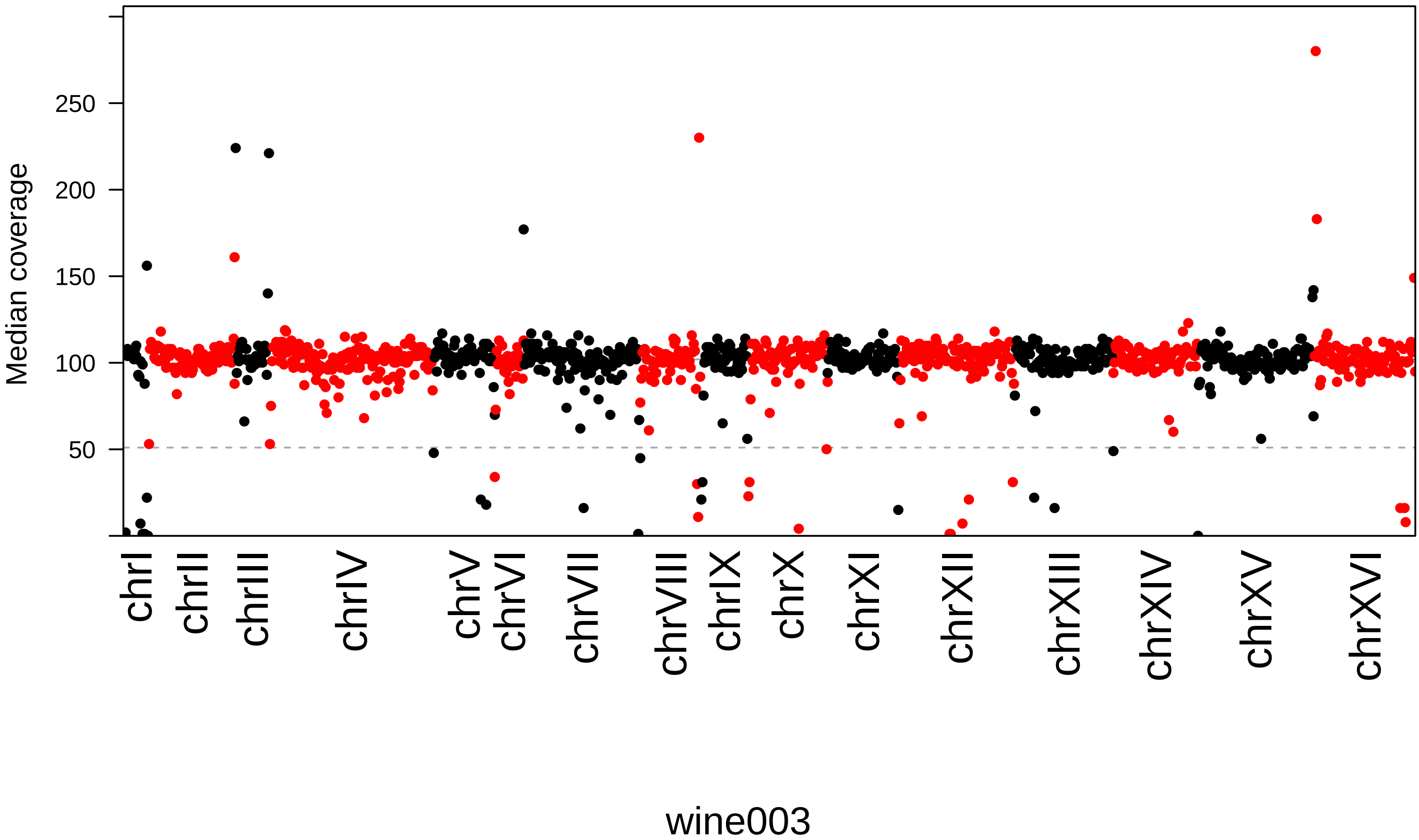

Supplementary Figure S2

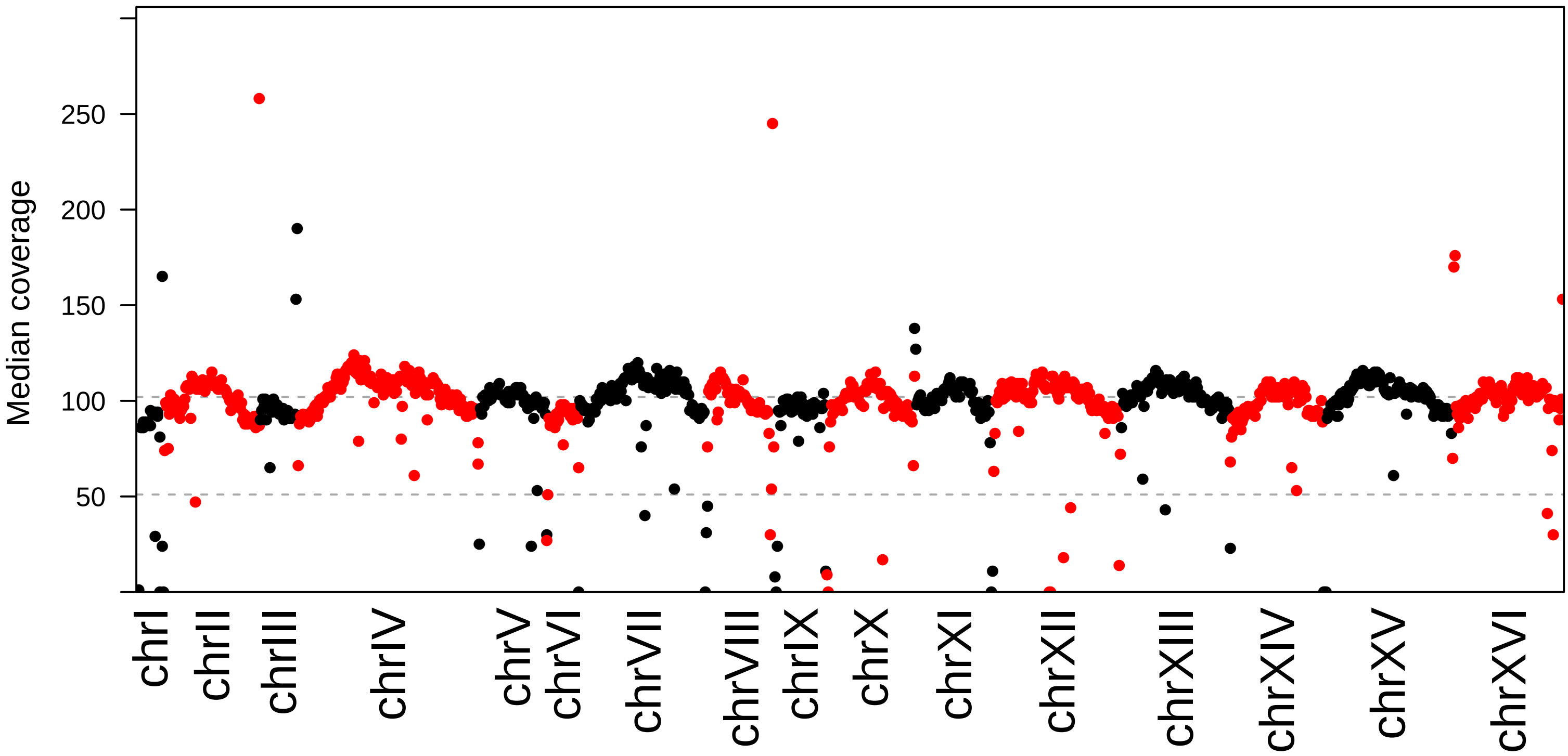

wine005

Supplementary Figure S3

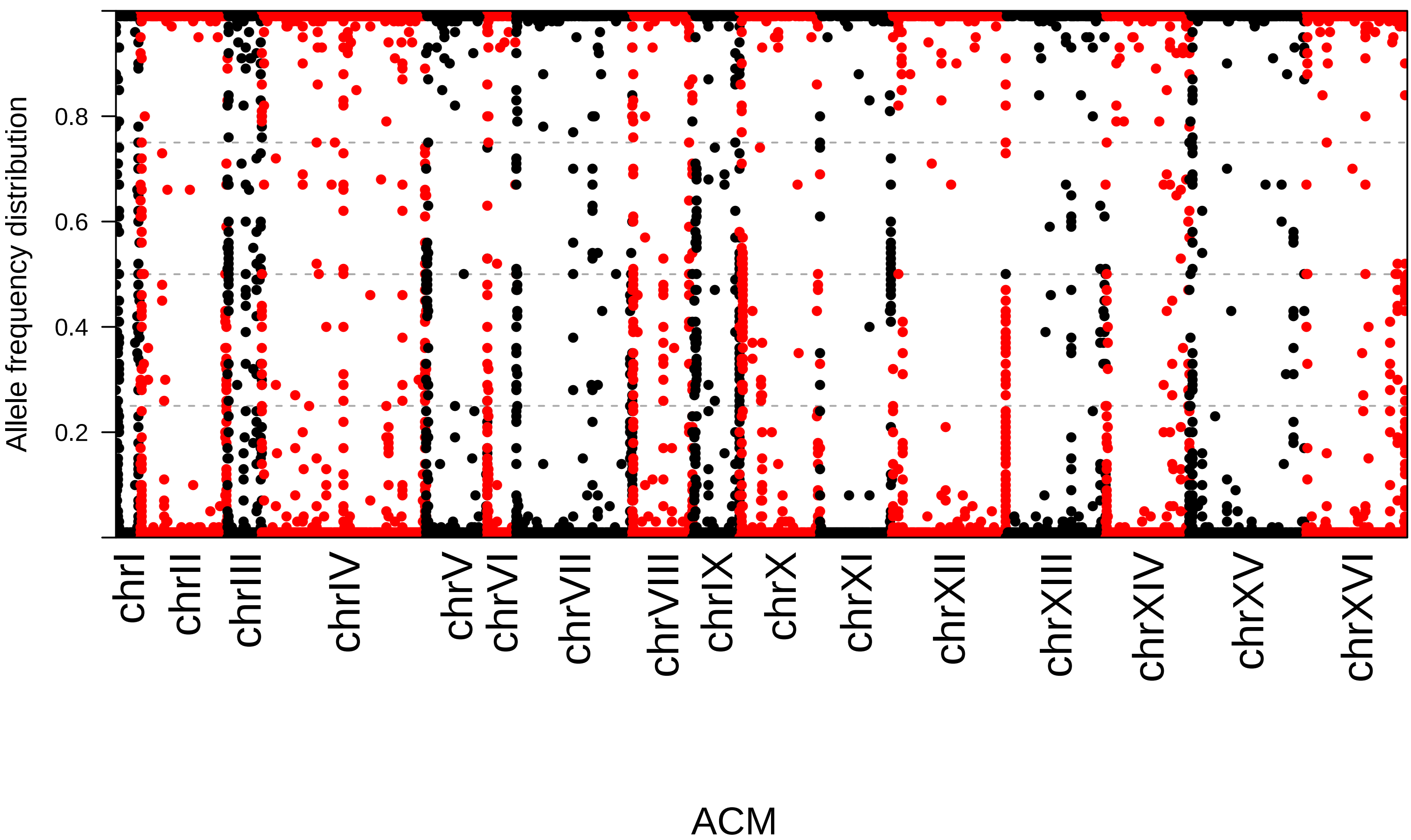

Supplementary Figure S3

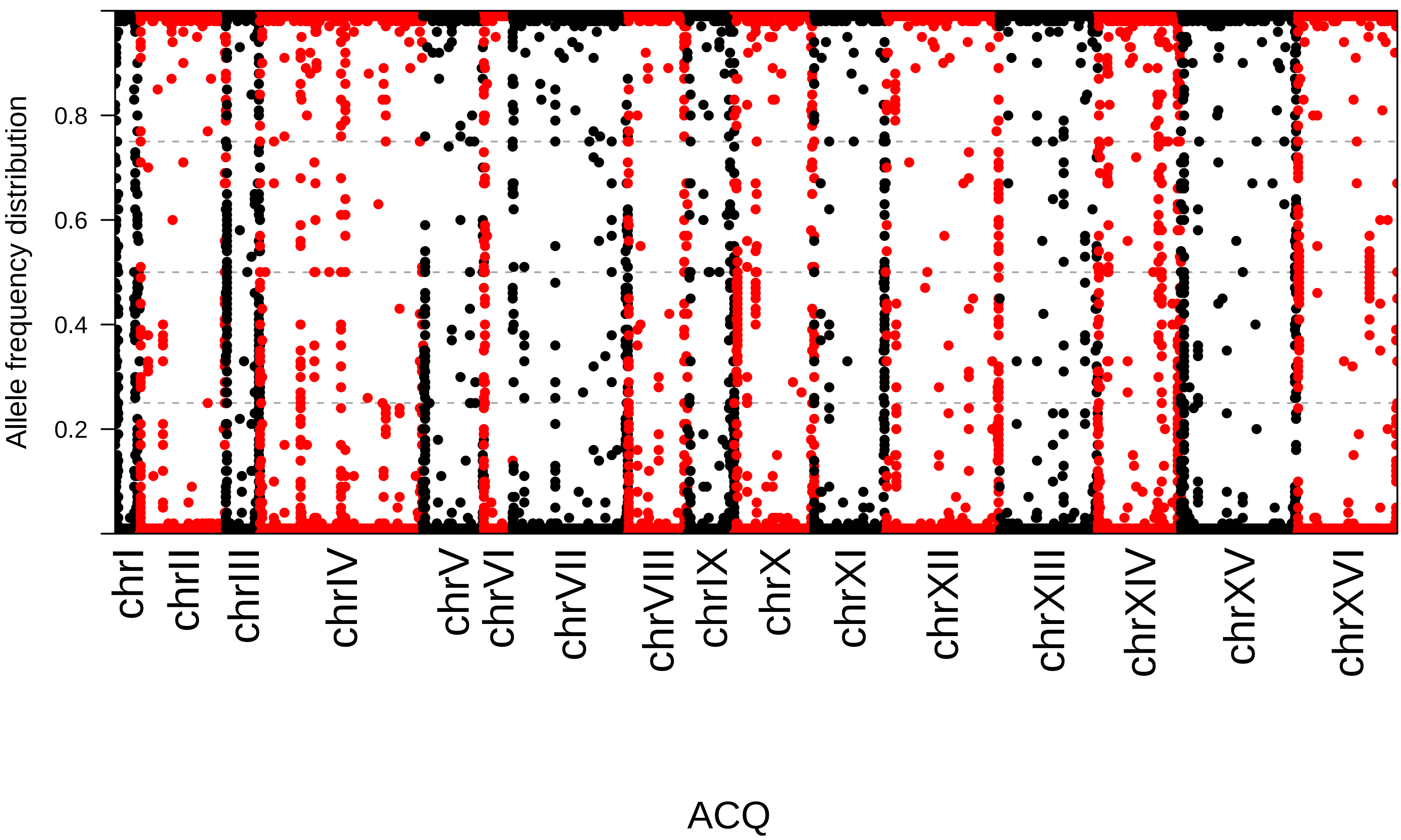

Supplementary Figure S3

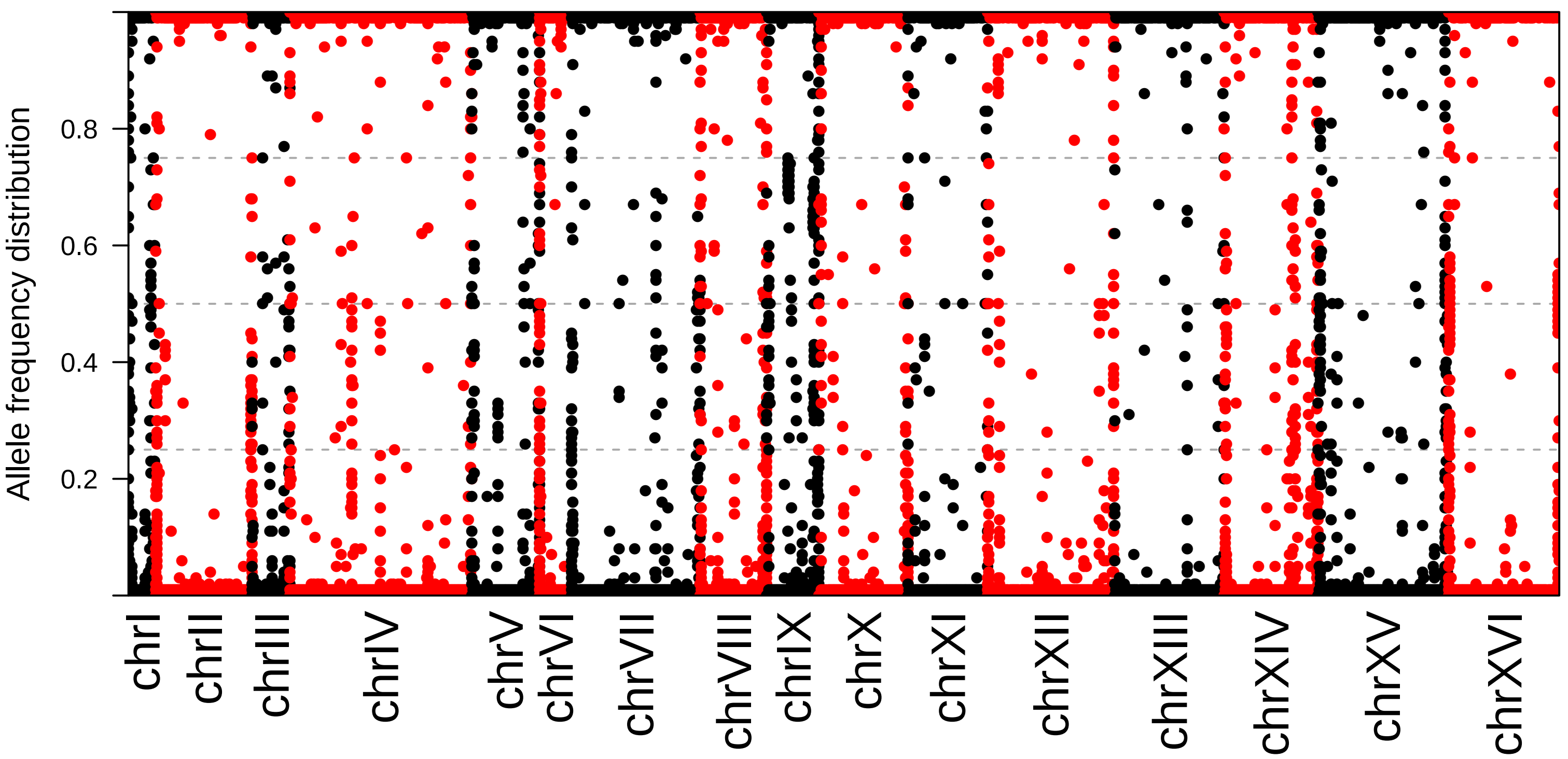

AEA

Supplementary Figure S3

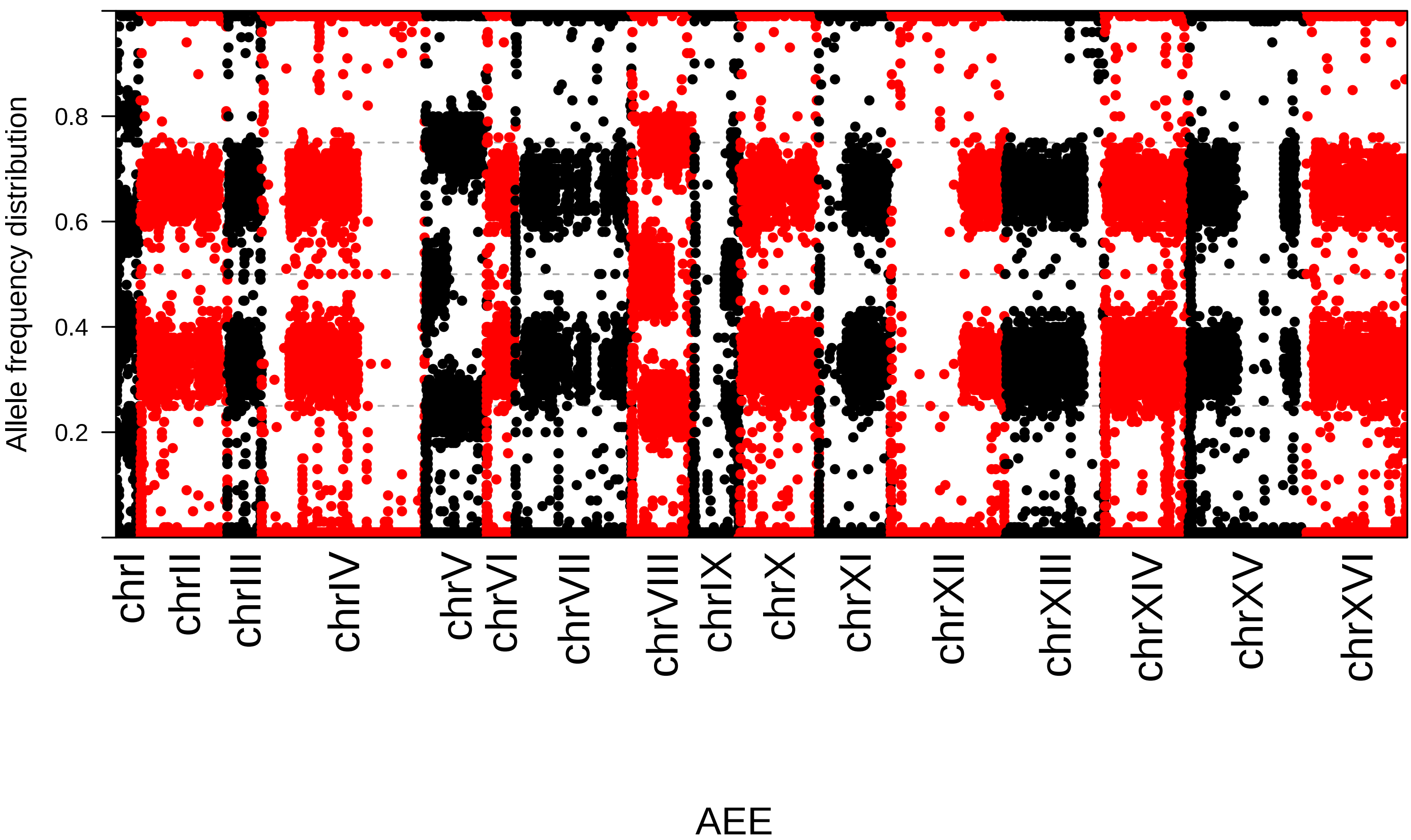

Supplementary Figure S3

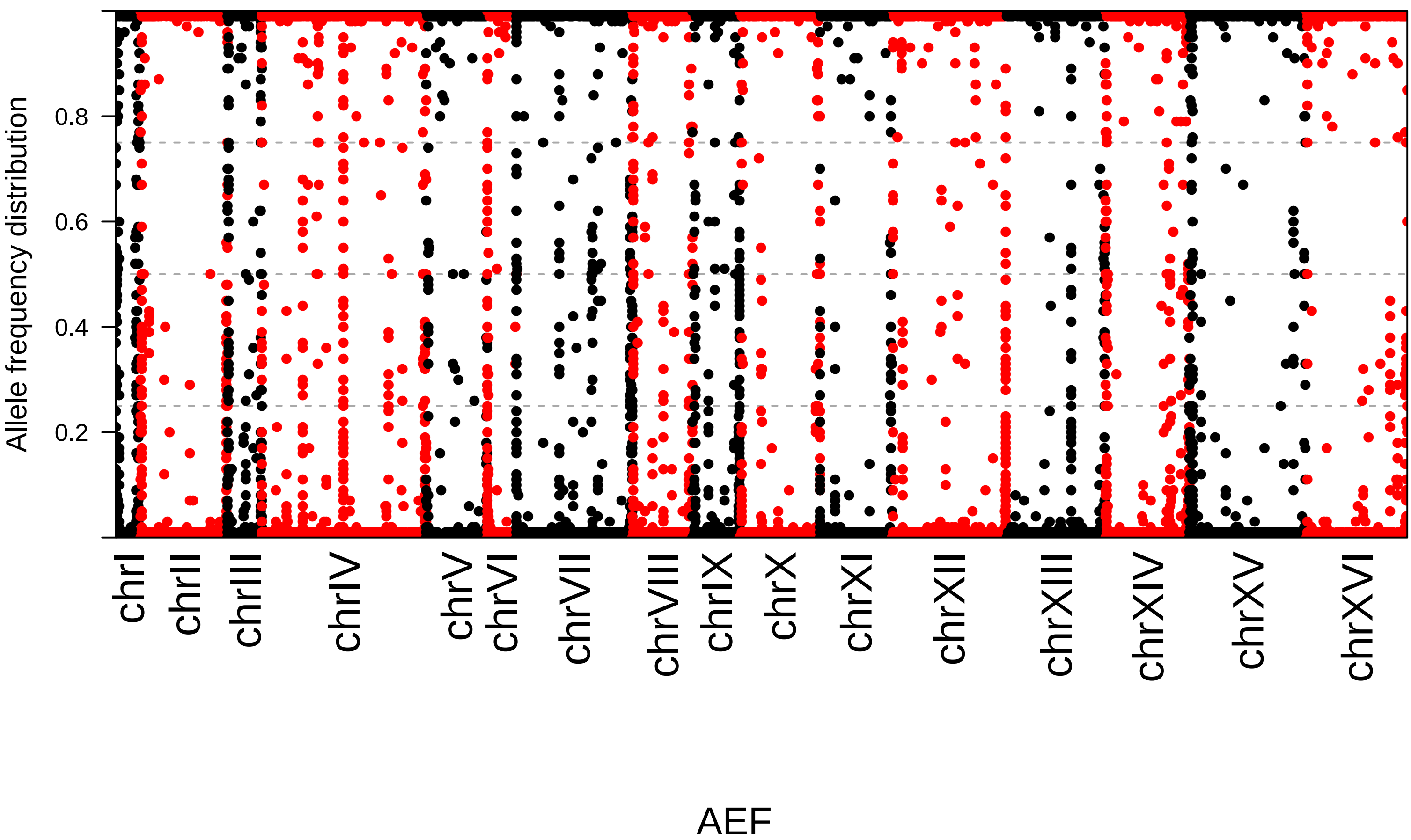

Supplementary Figure S3

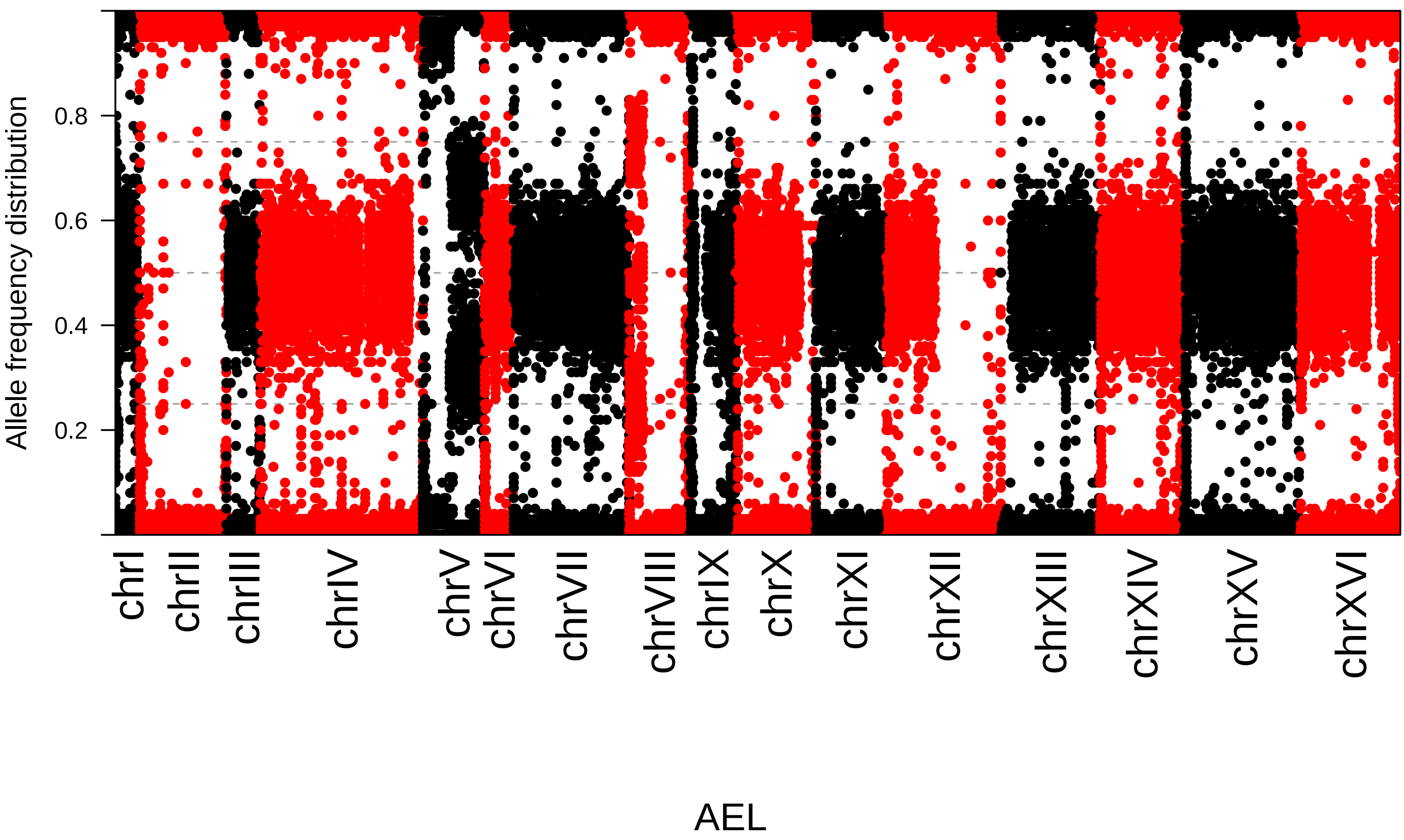

Supplementary Figure S3

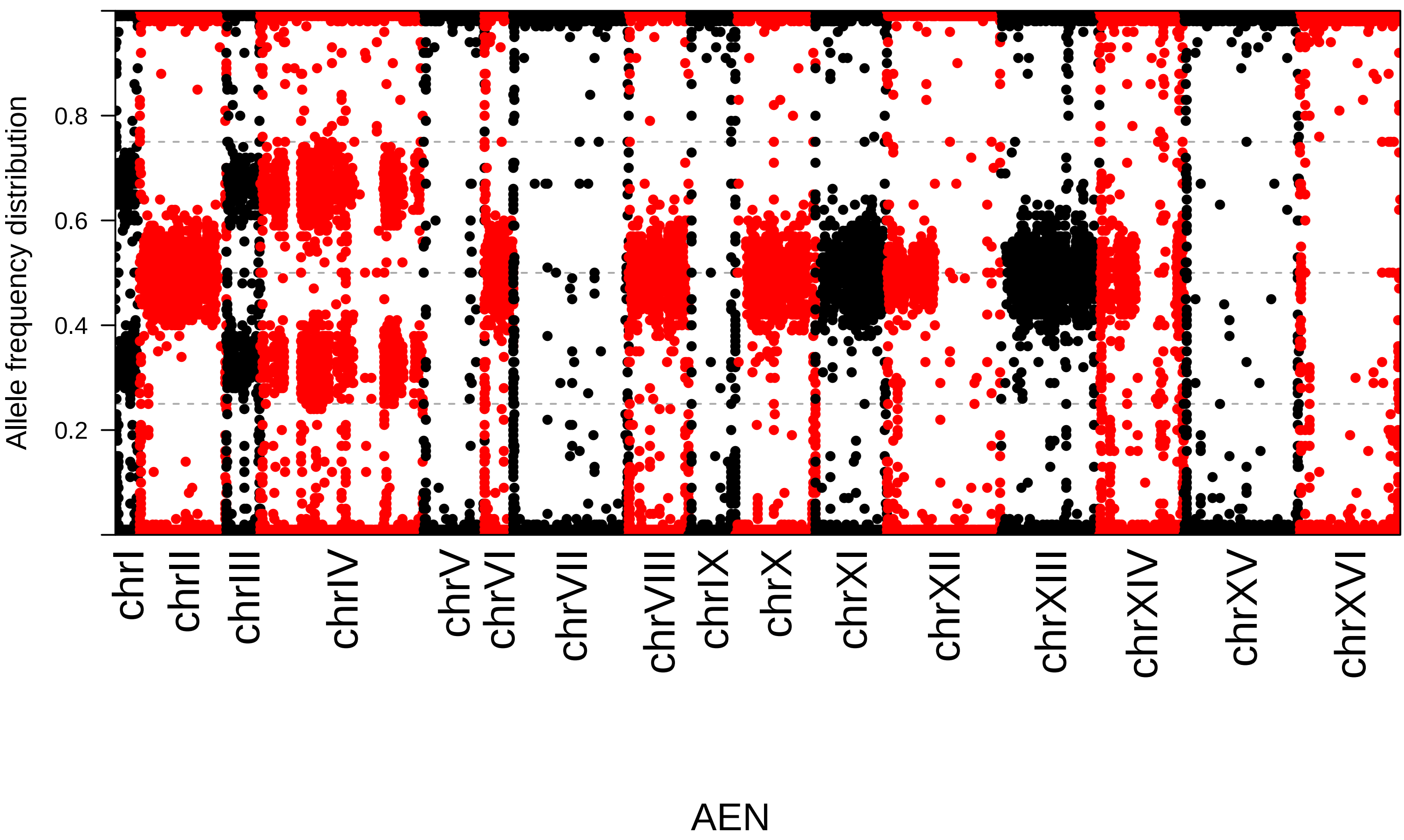

Supplementary Figure S3

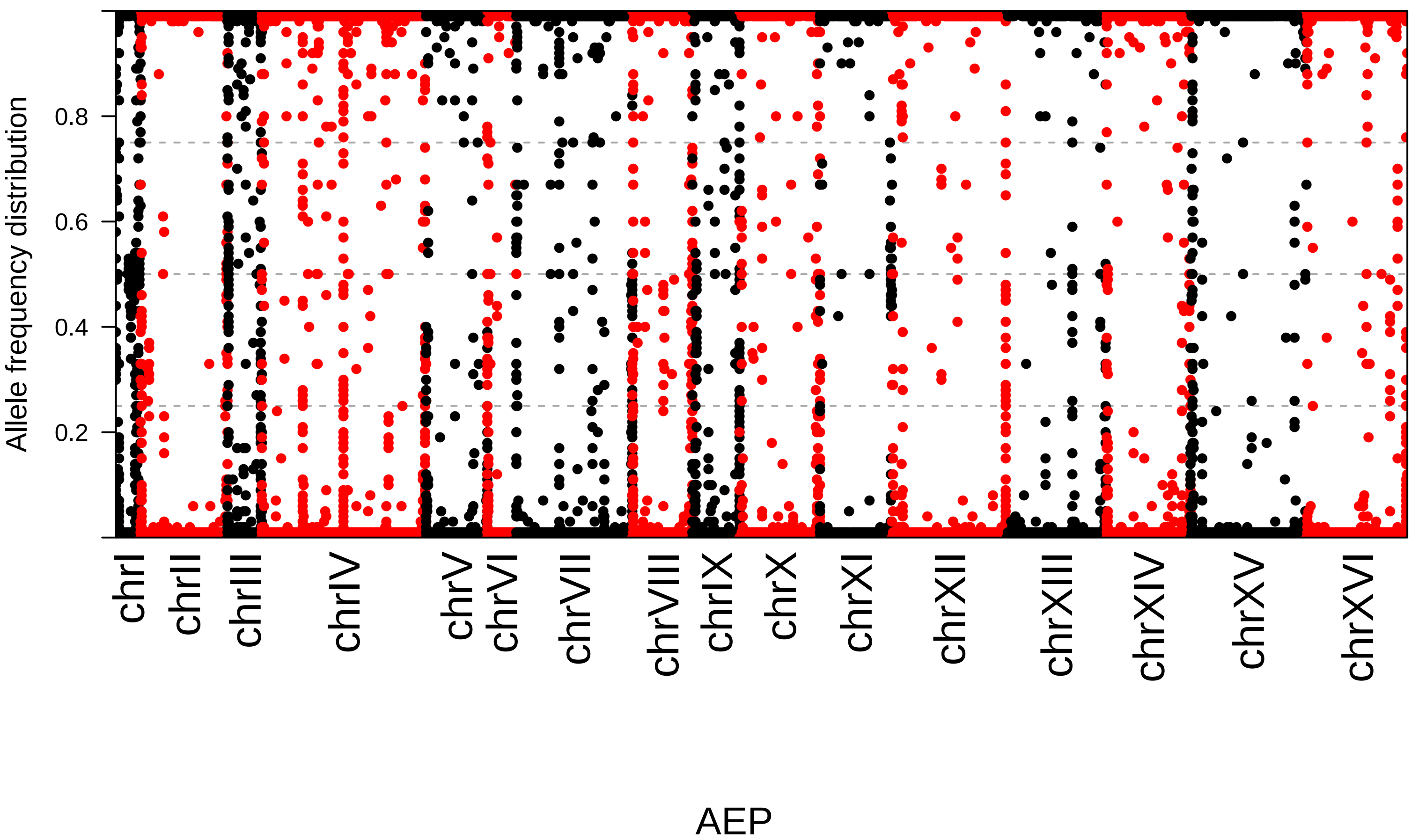

Supplementary Figure S3

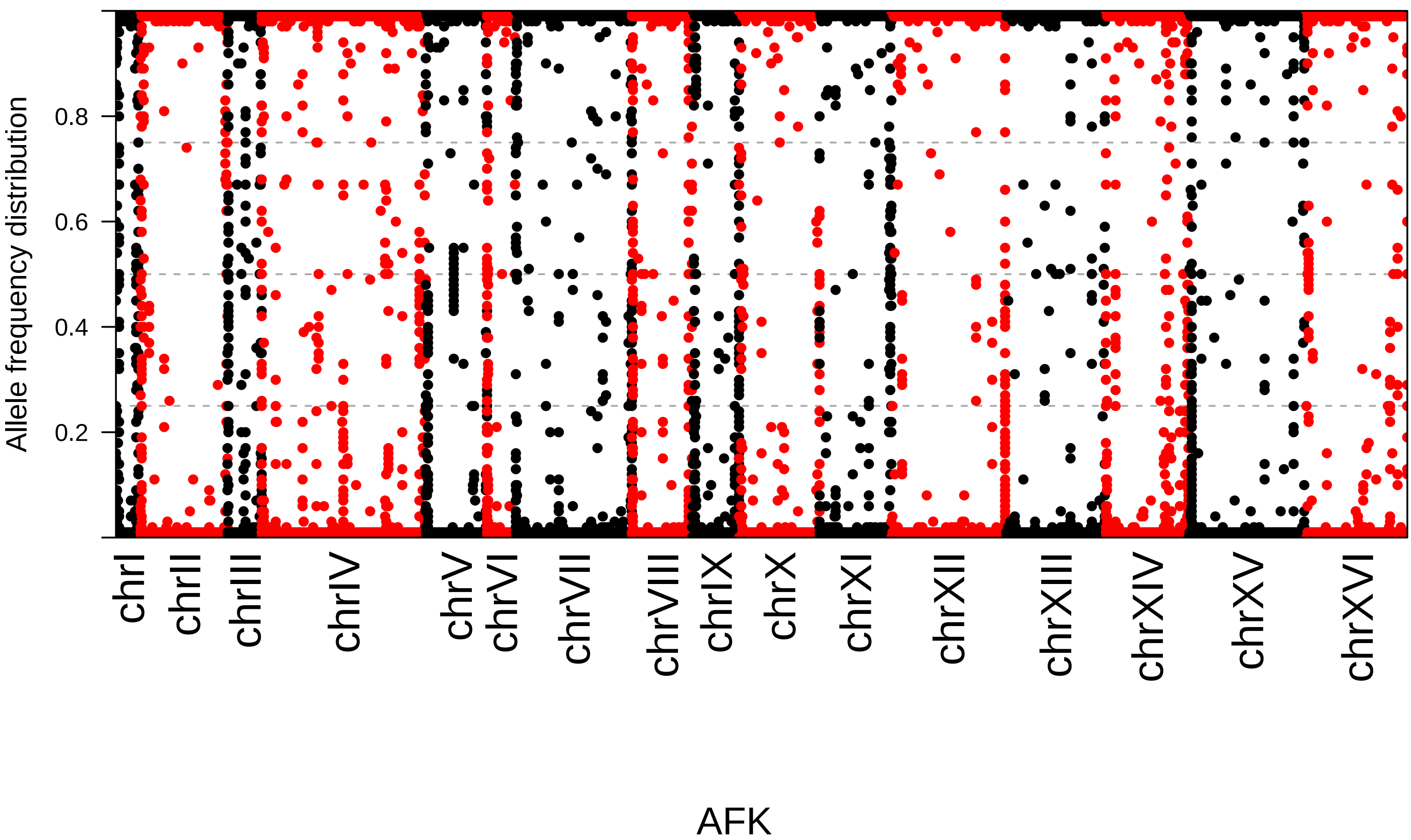

Supplementary Figure S3

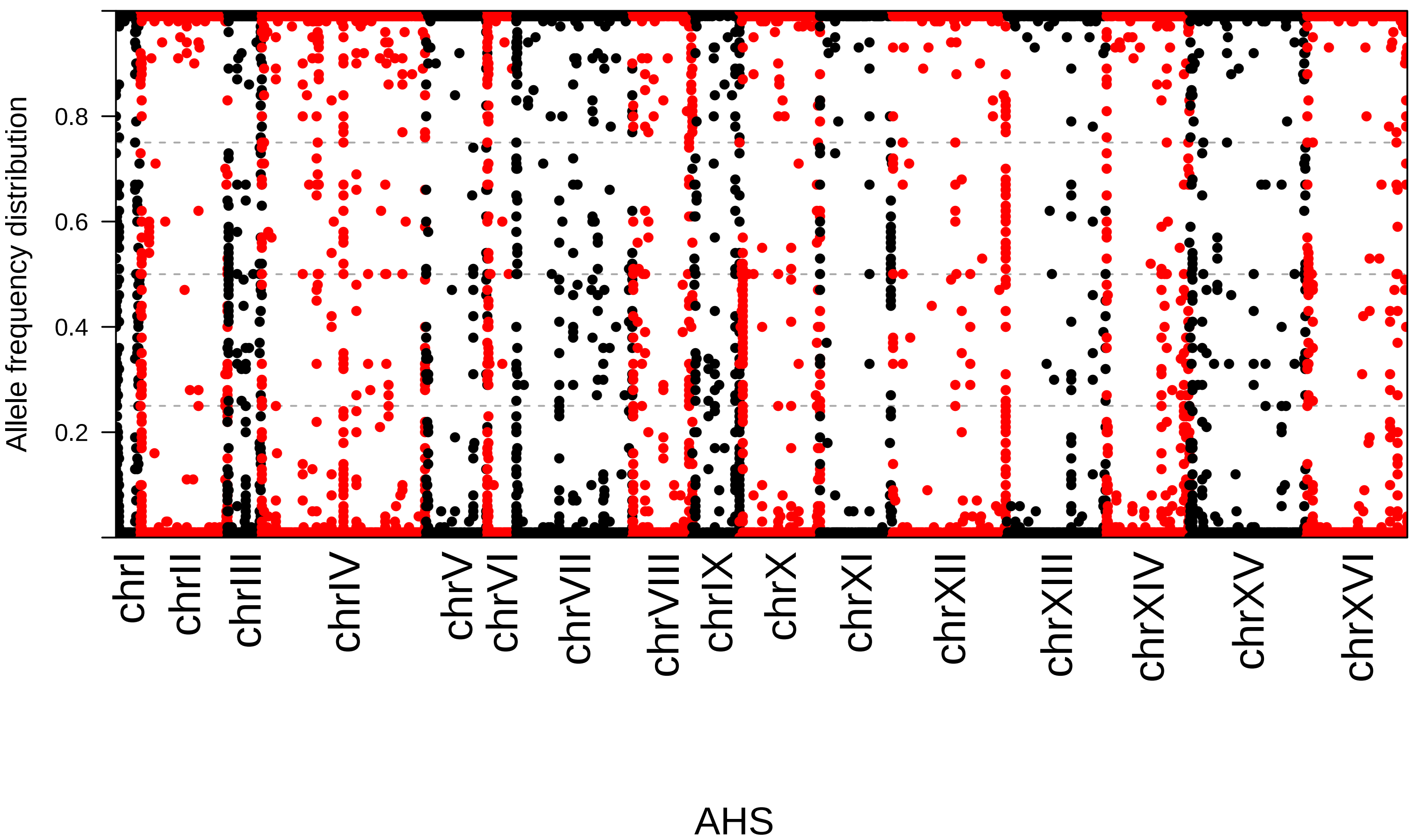

Supplementary Figure S3

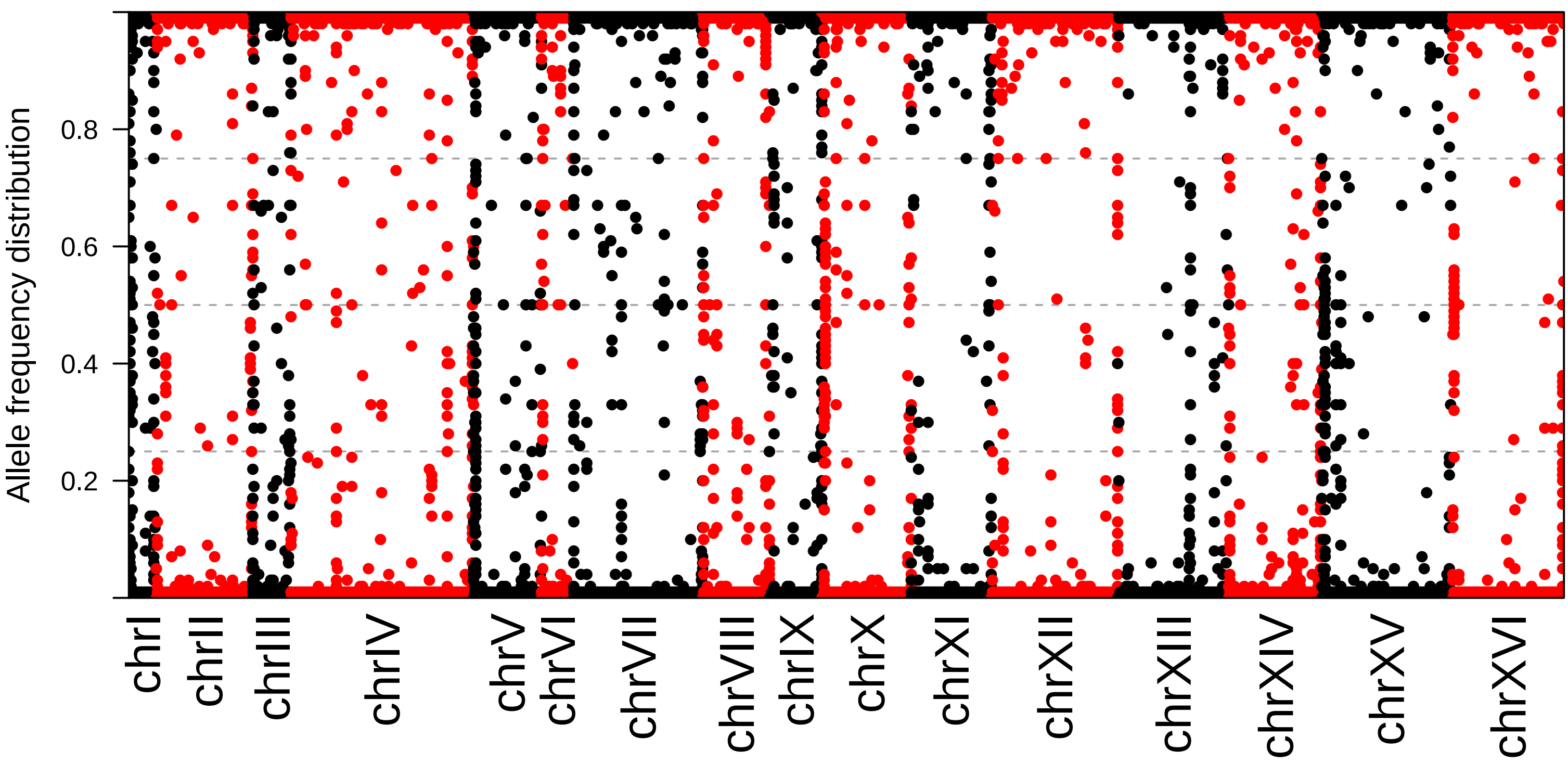

AKM

Supplementary Figure S3

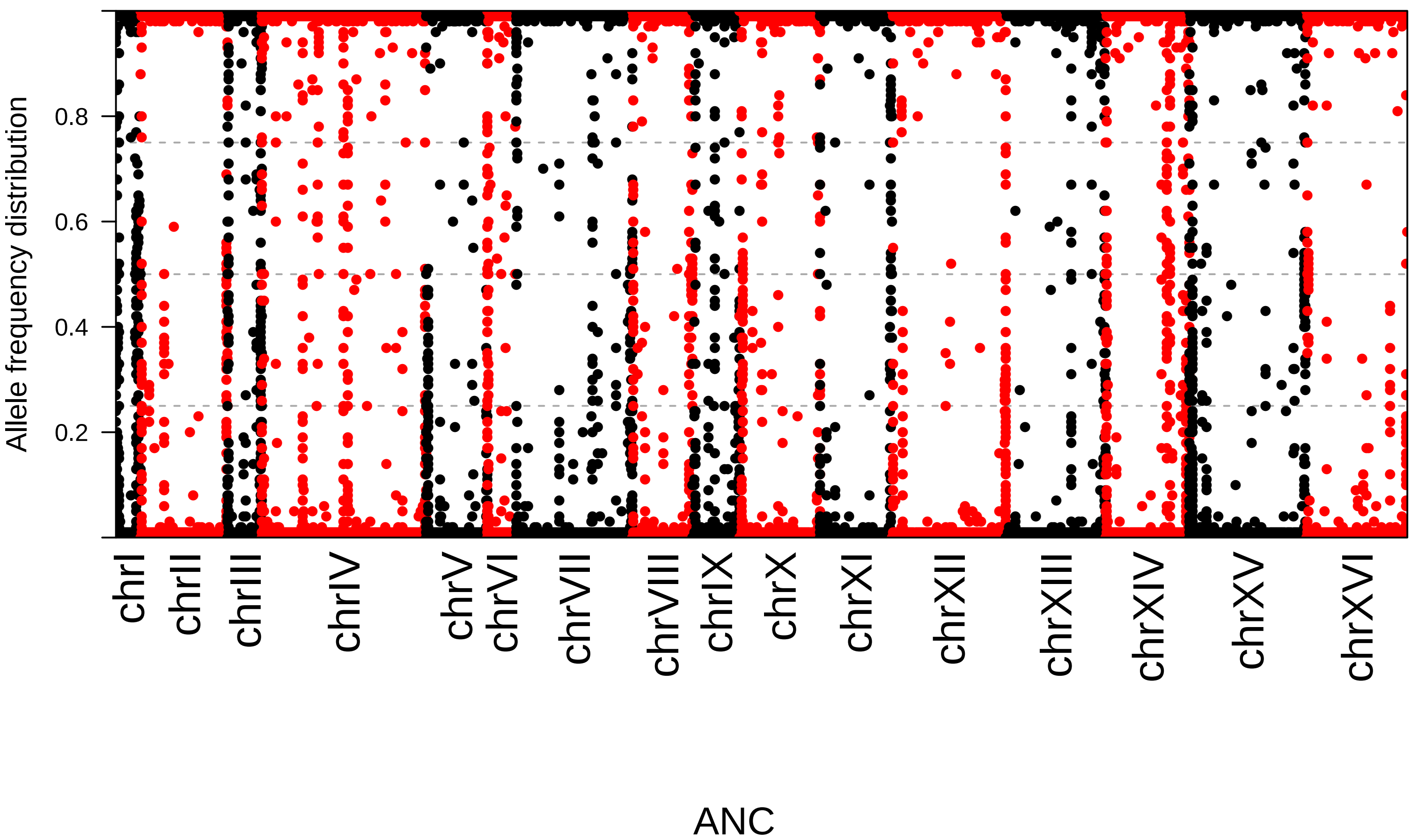

Supplementary Figure S3

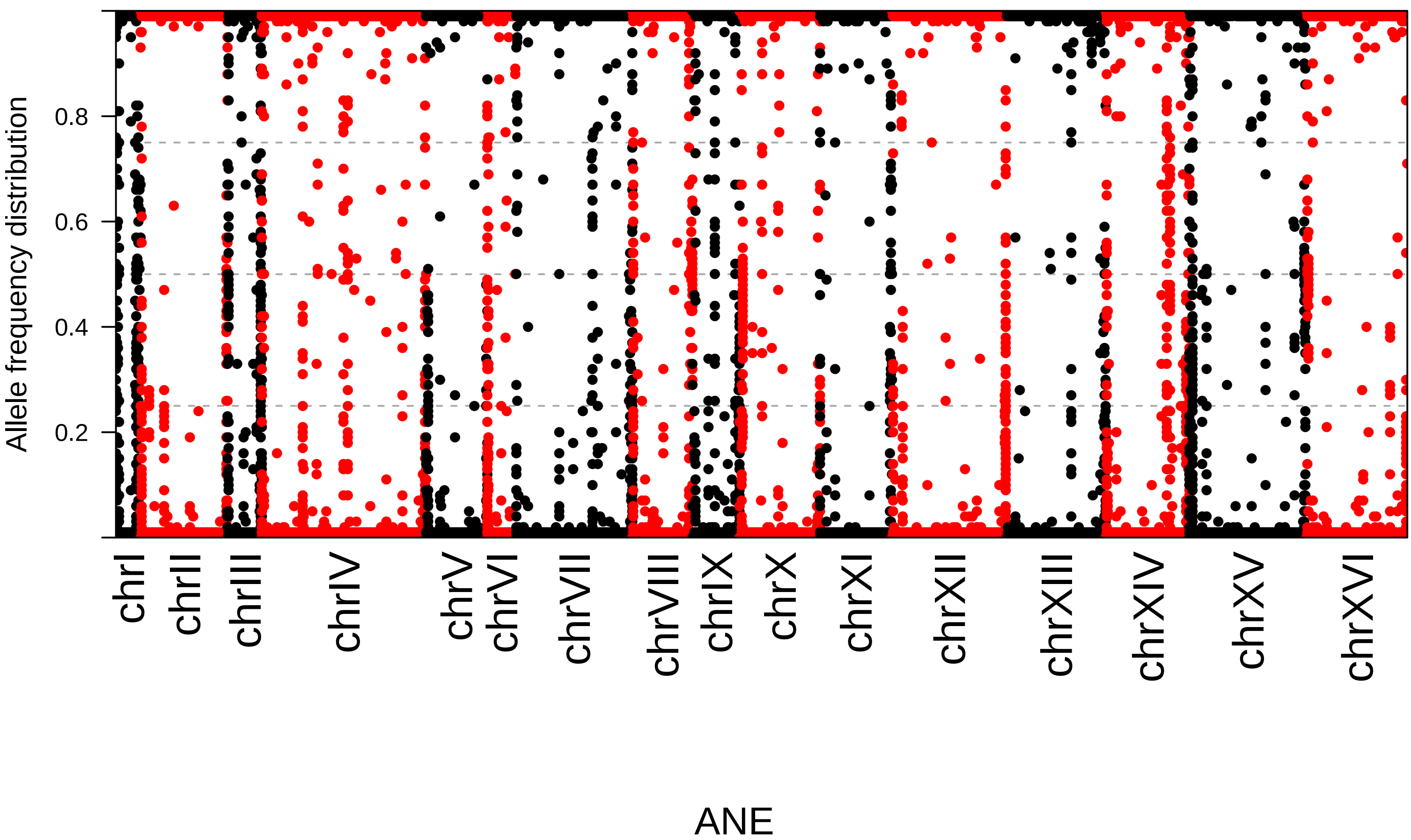

Supplementary Figure S3

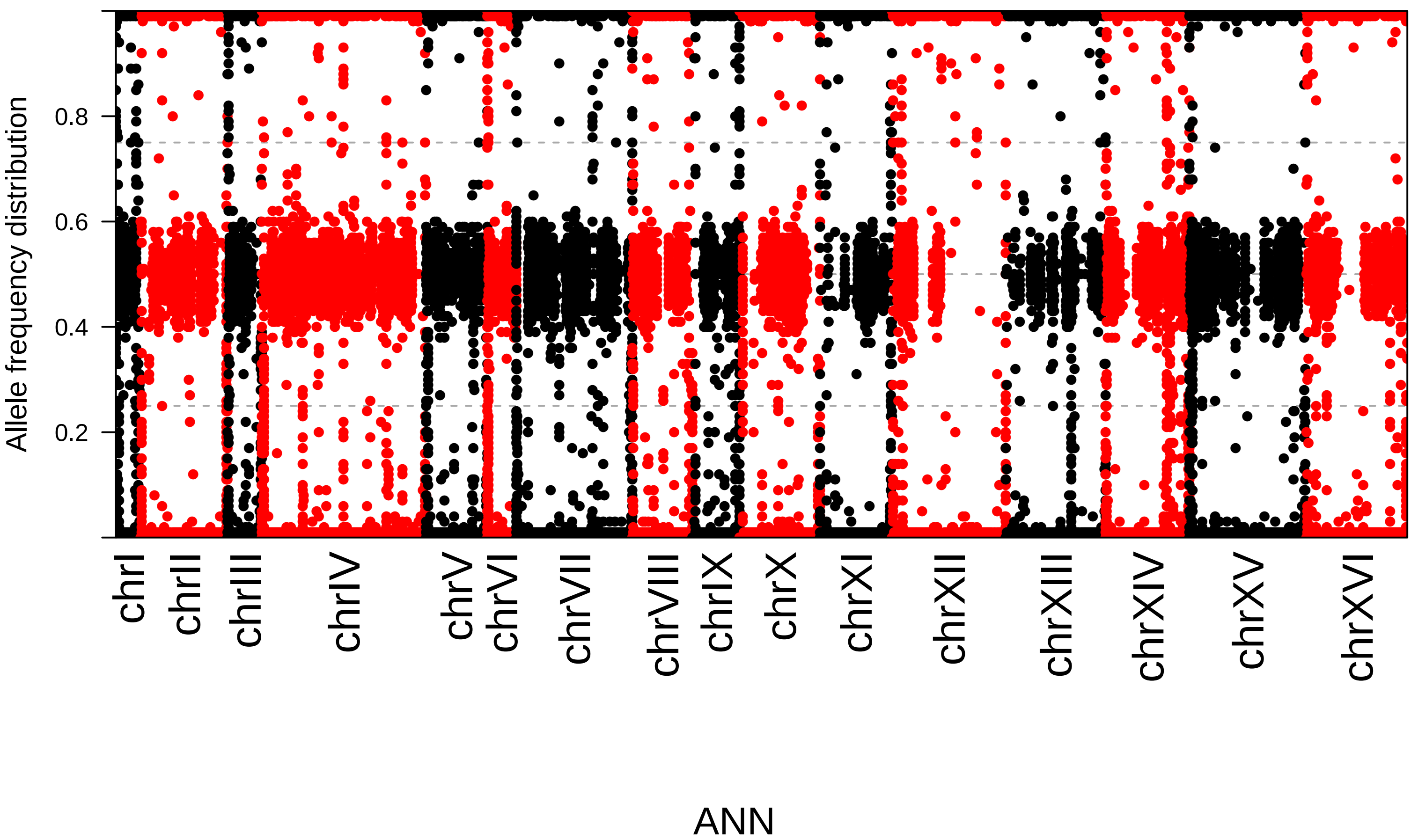

Supplementary Figure S3

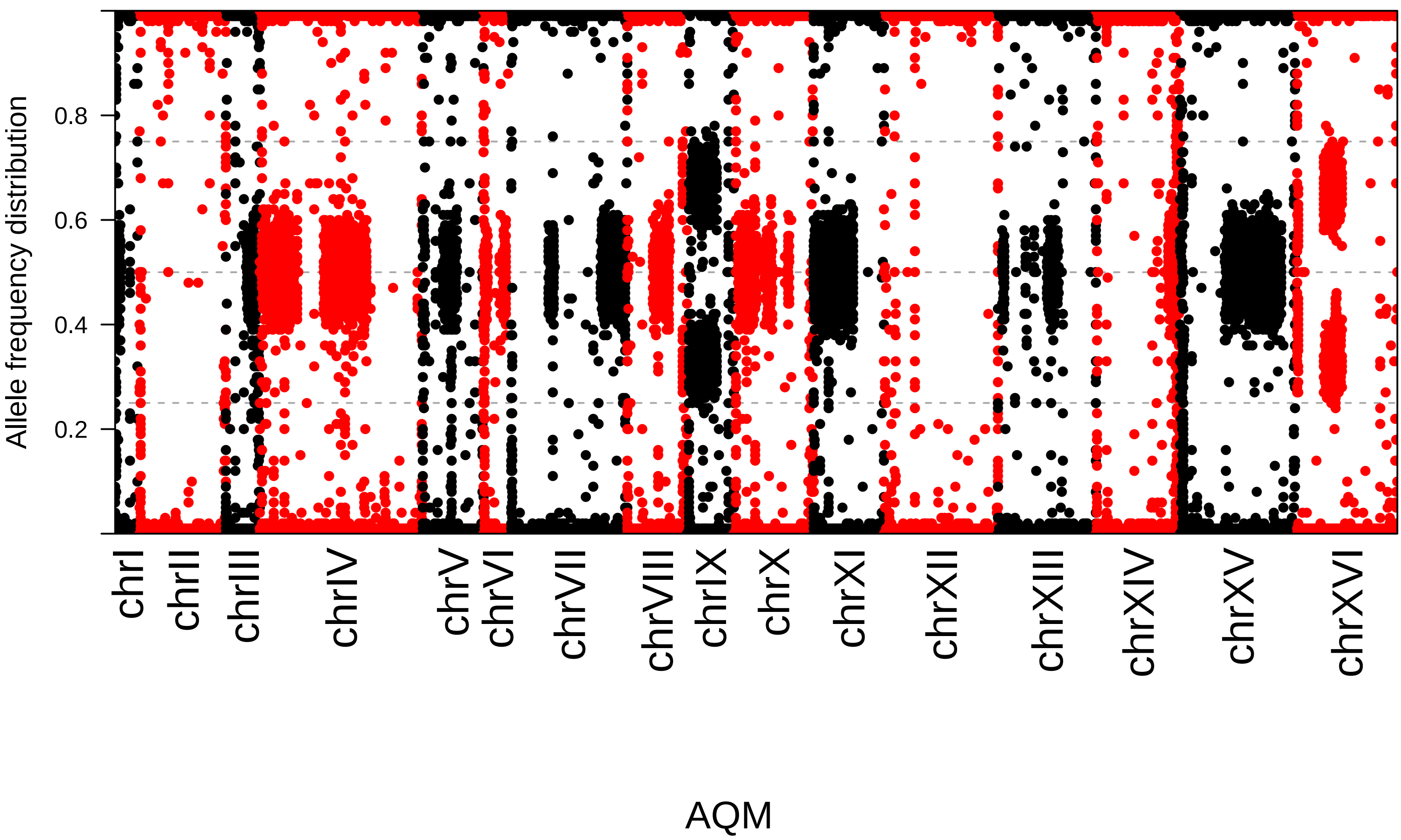

Supplementary Figure S3

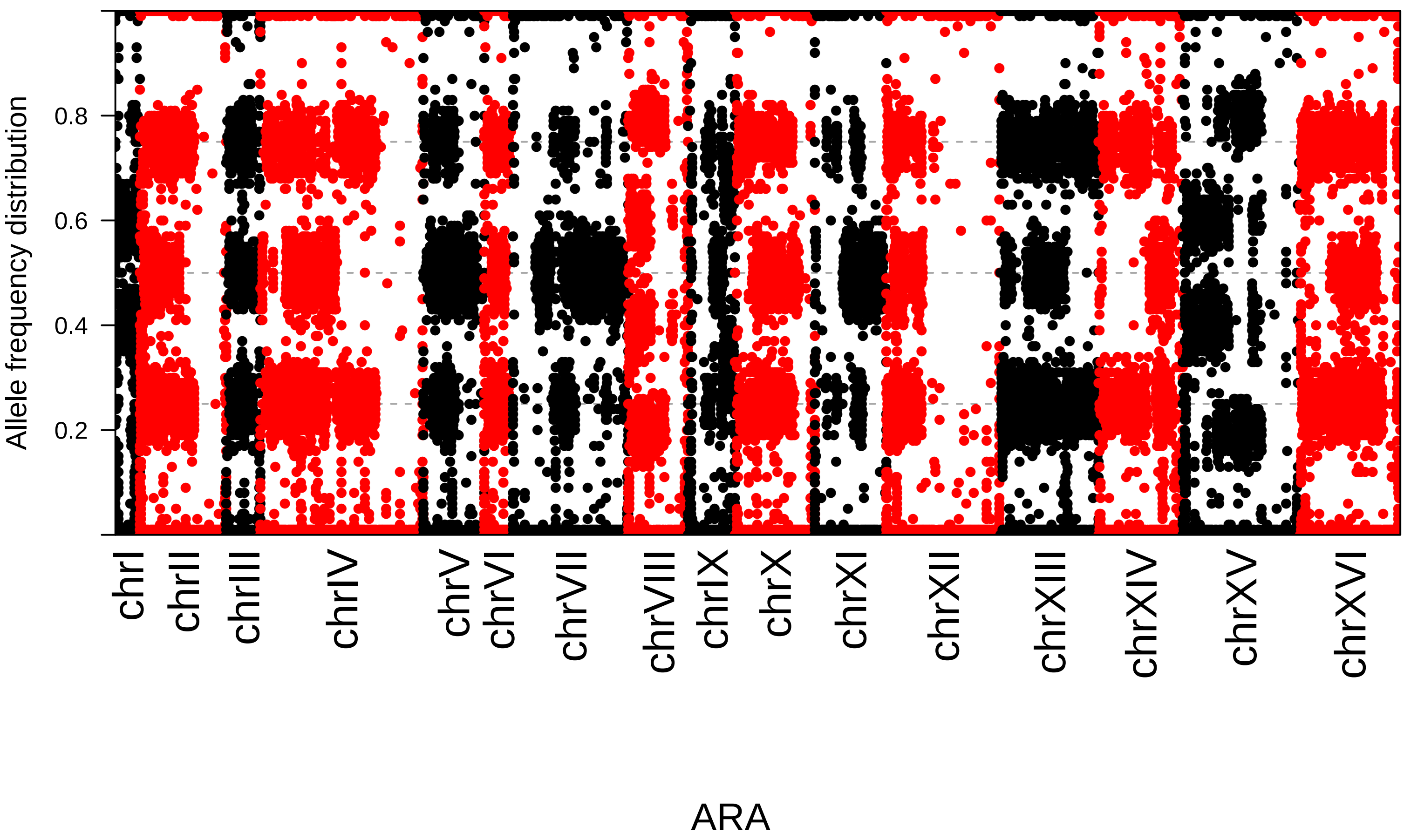

Supplementary Figure S3

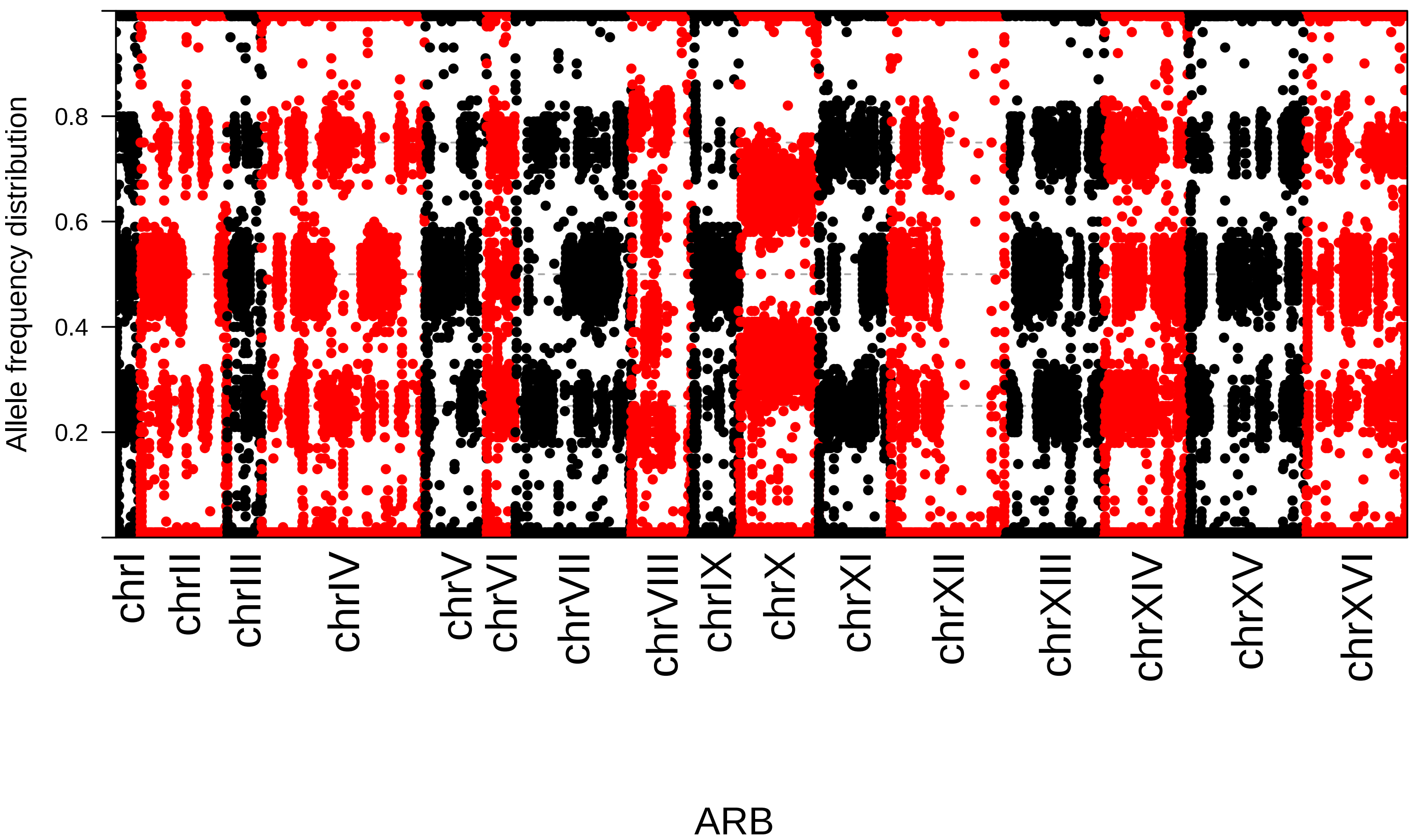

Supplementary Figure S3

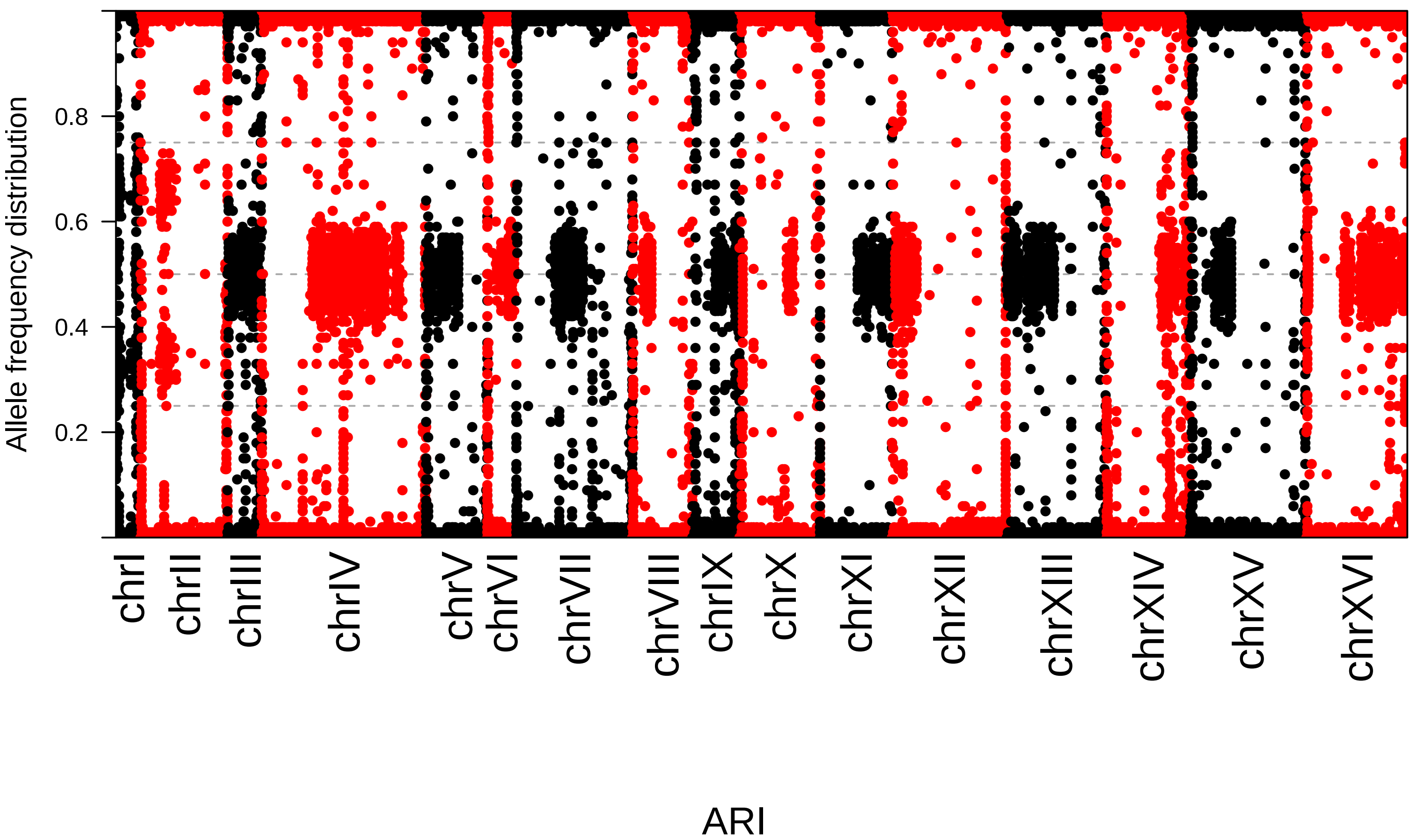

Supplementary Figure S3

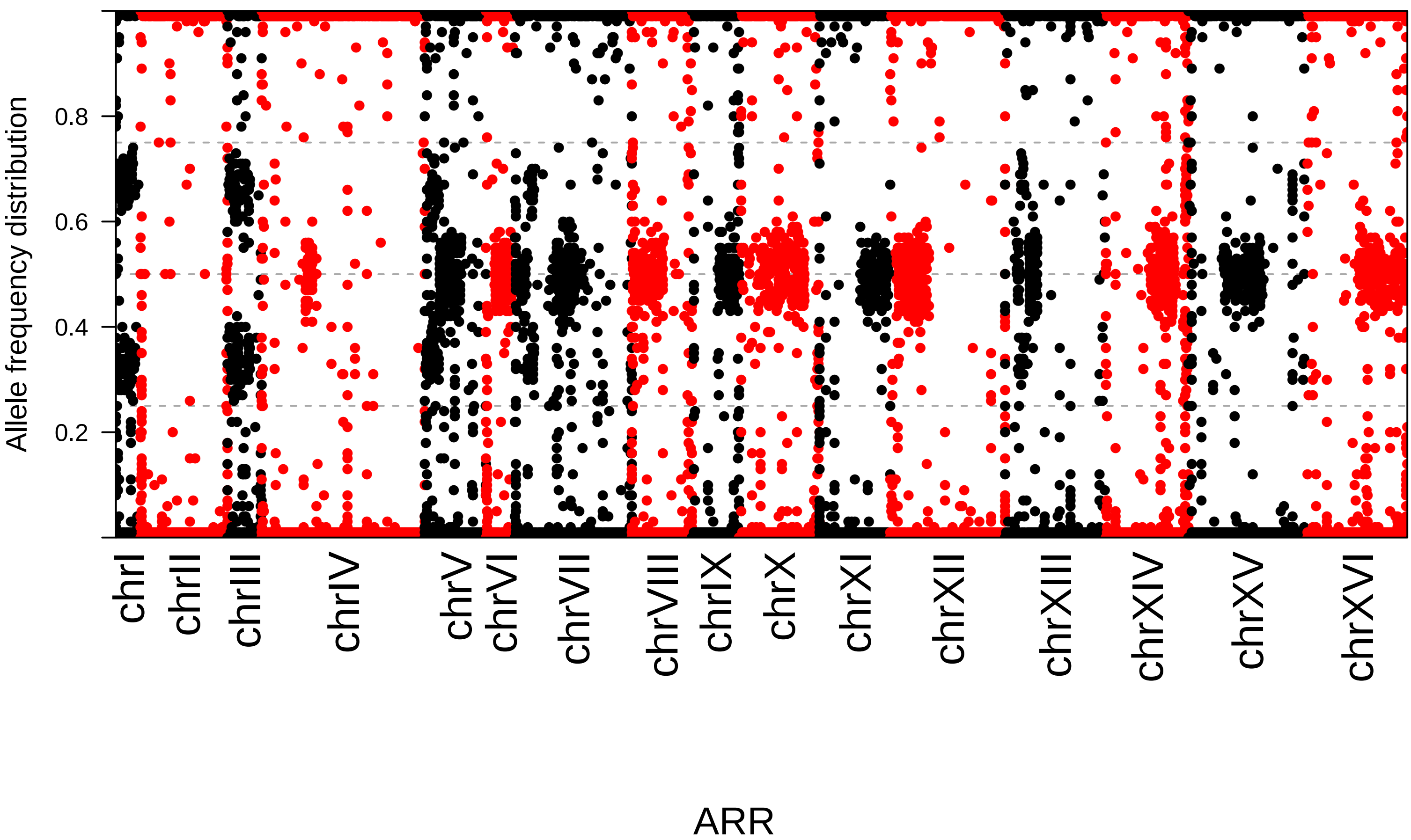

Supplementary Figure S3

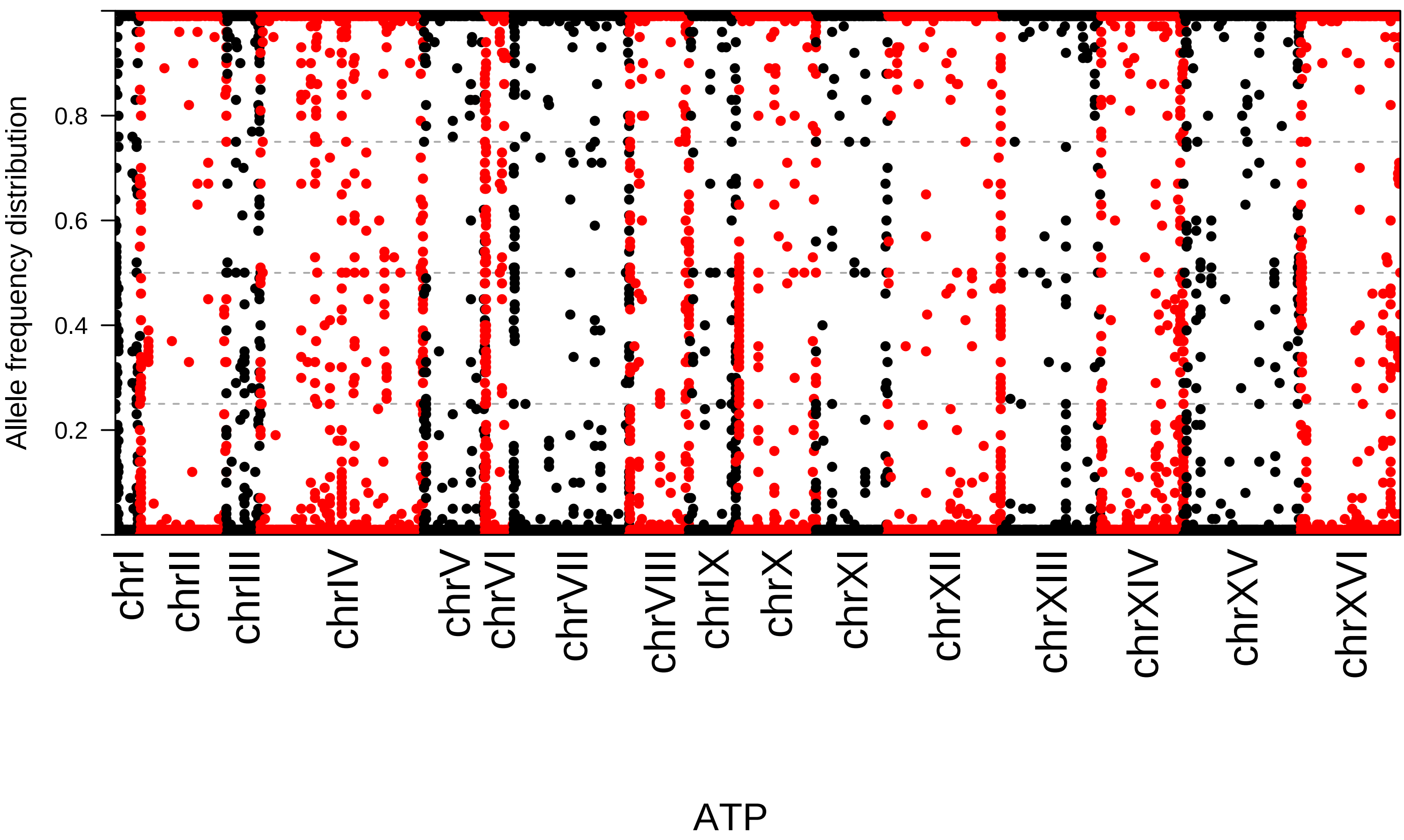

Supplementary Figure S3

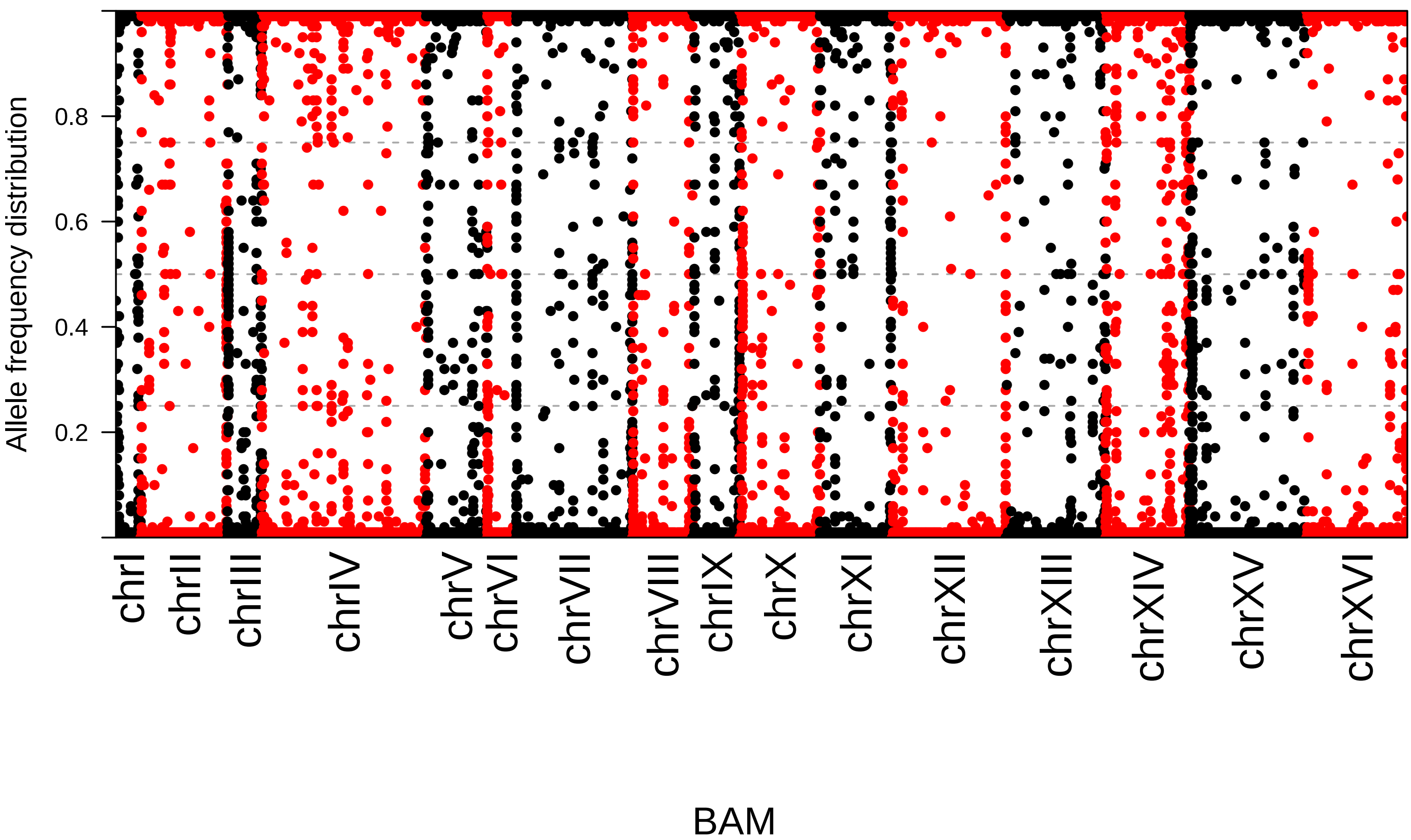

Supplementary Figure S3

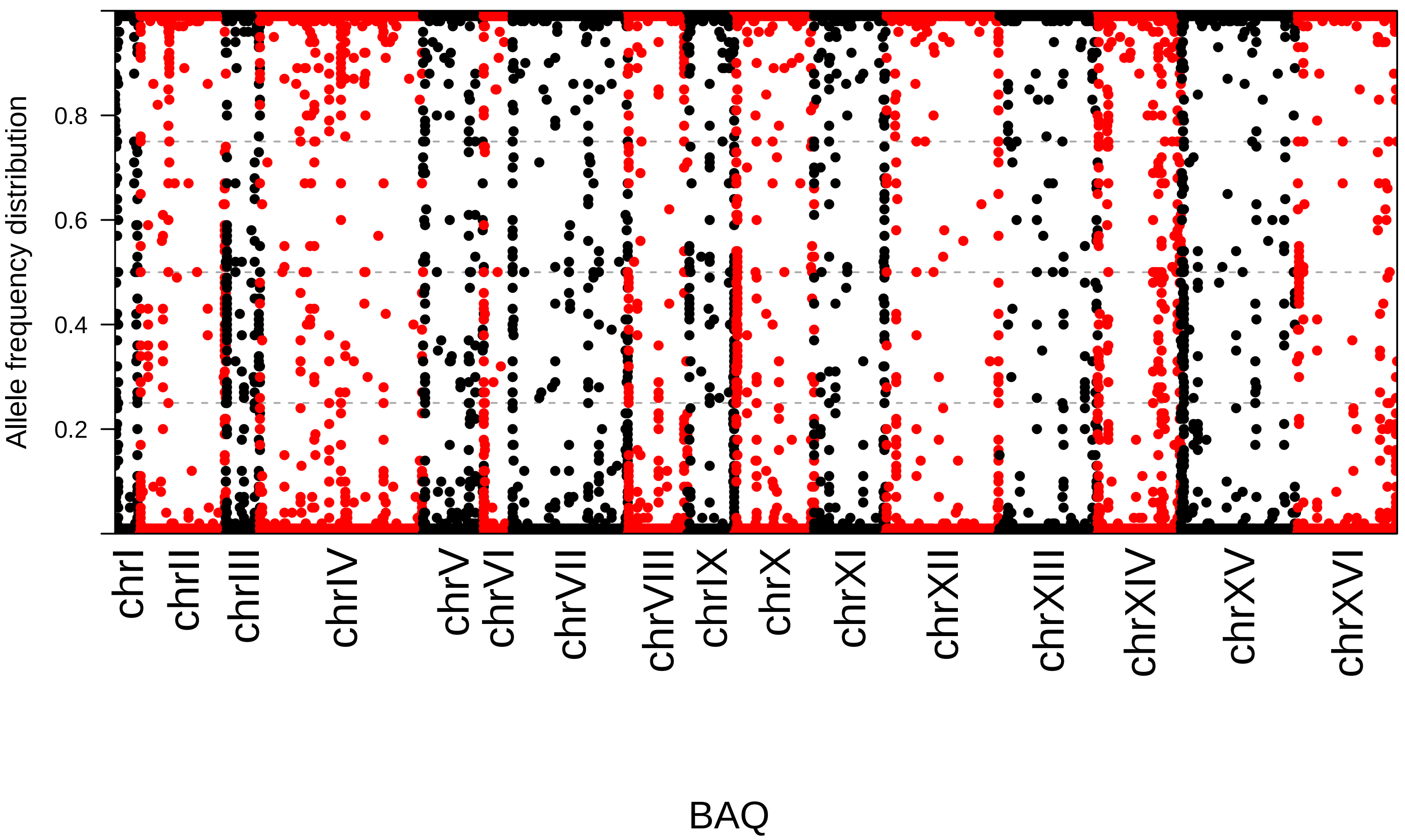

Supplementary Figure S3

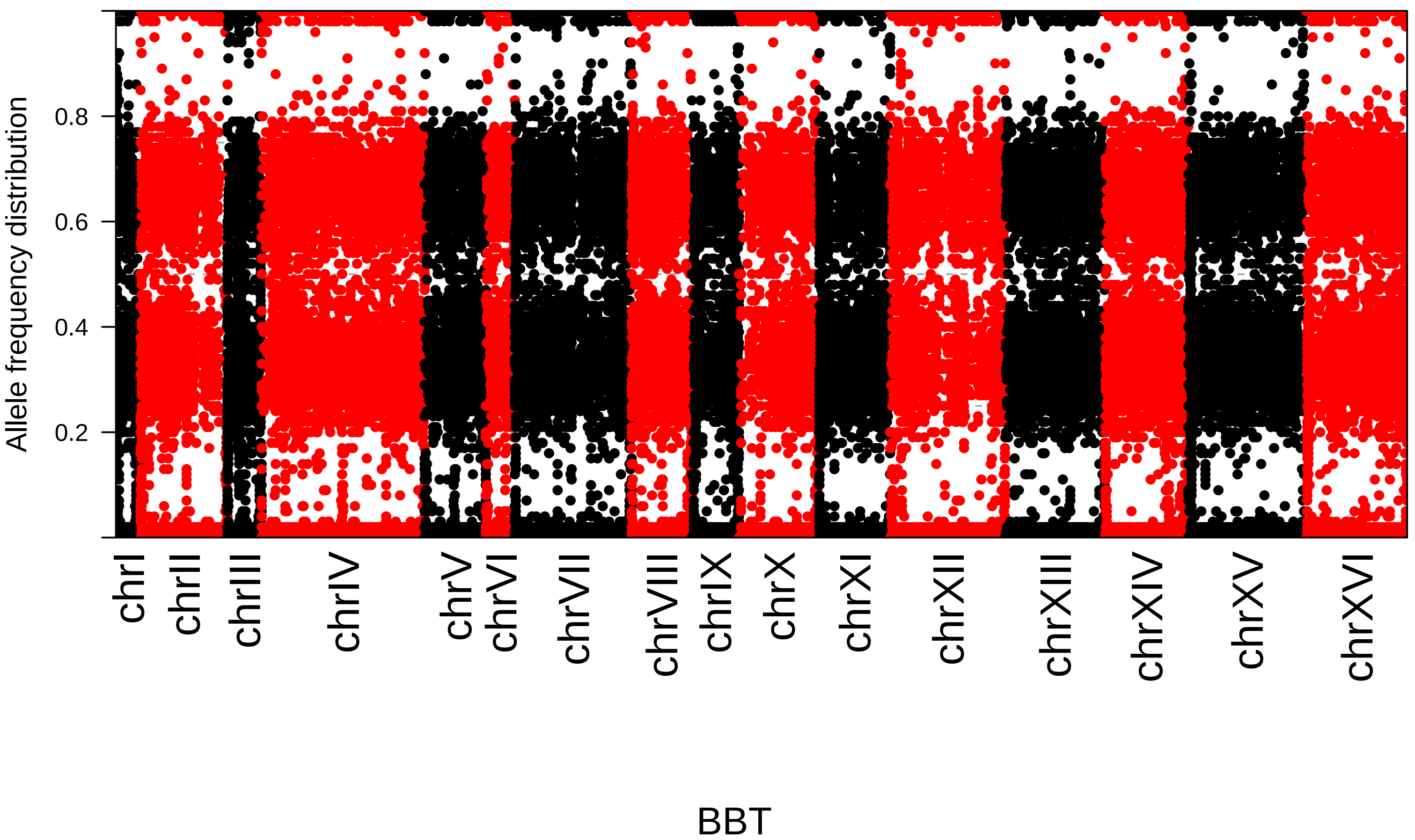

Supplementary Figure S3

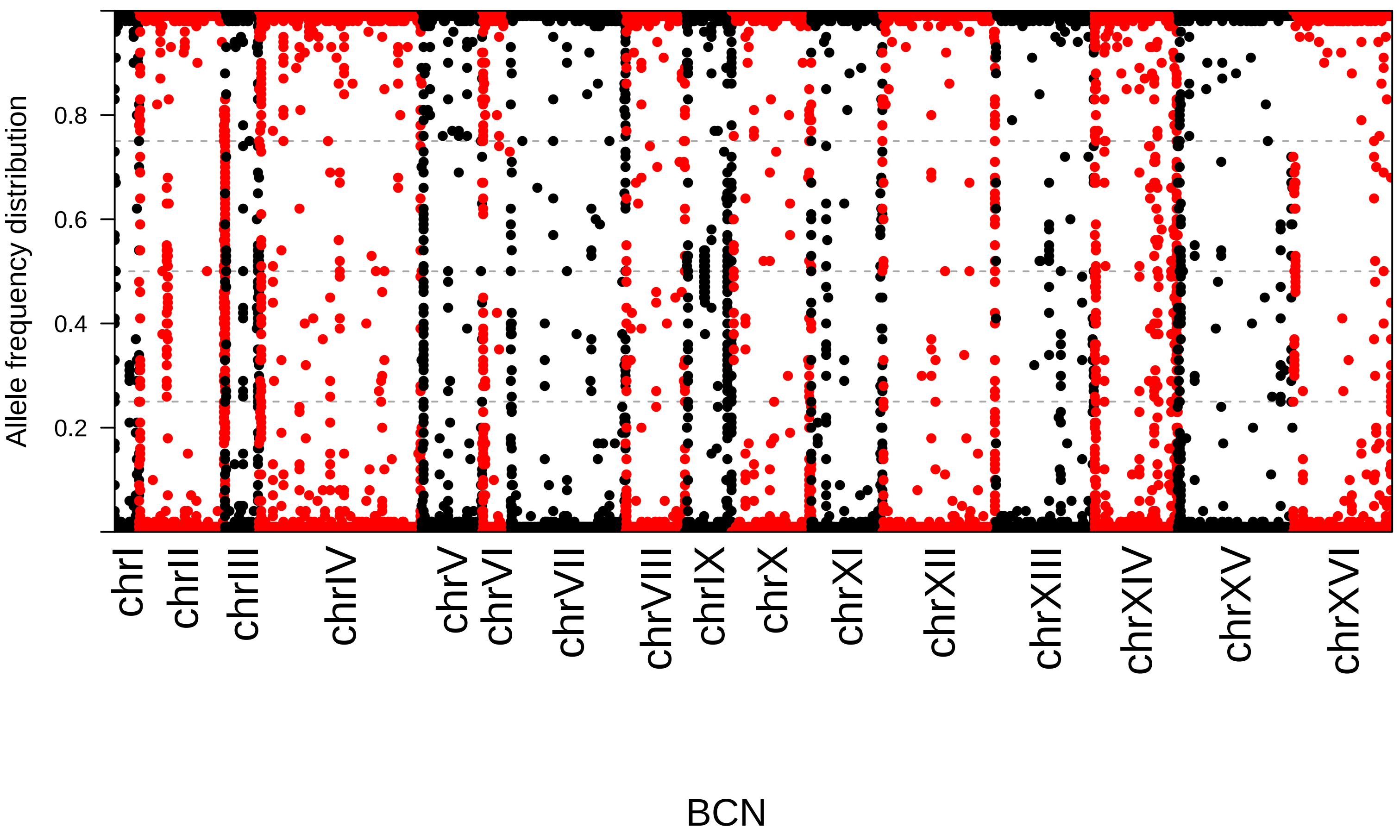

Supplementary Figure S3

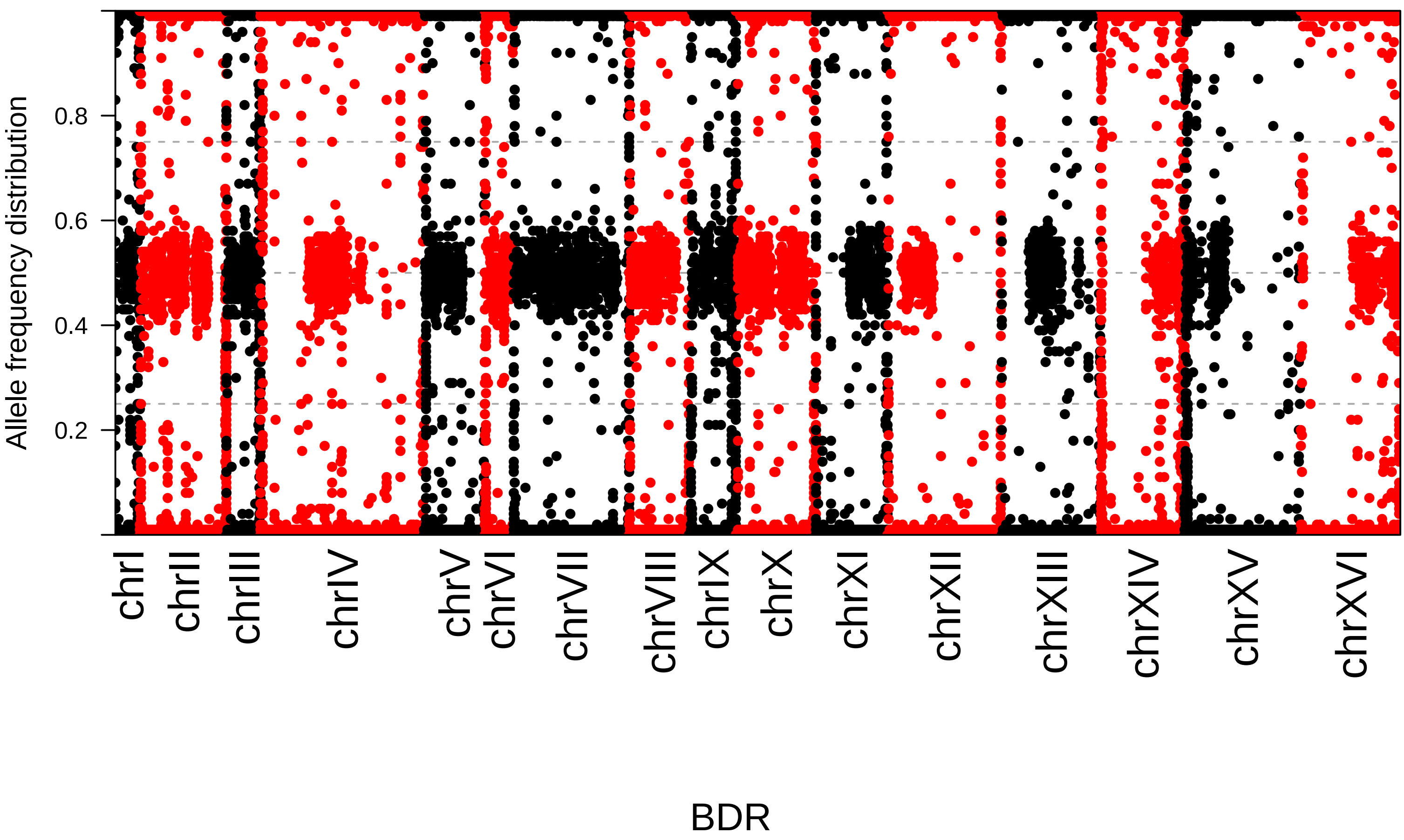

Supplementary Figure S3

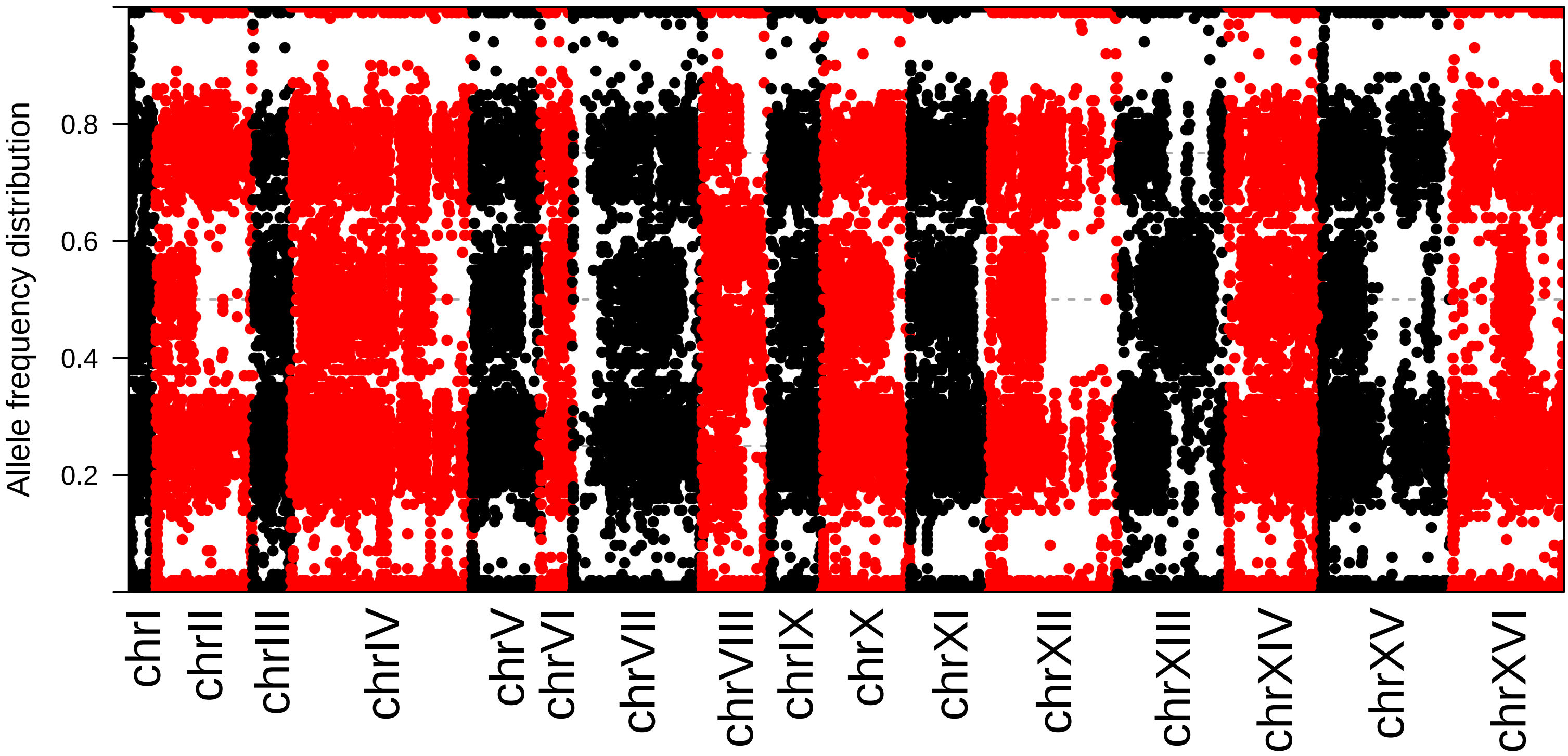

beer022

Supplementary Figure S3

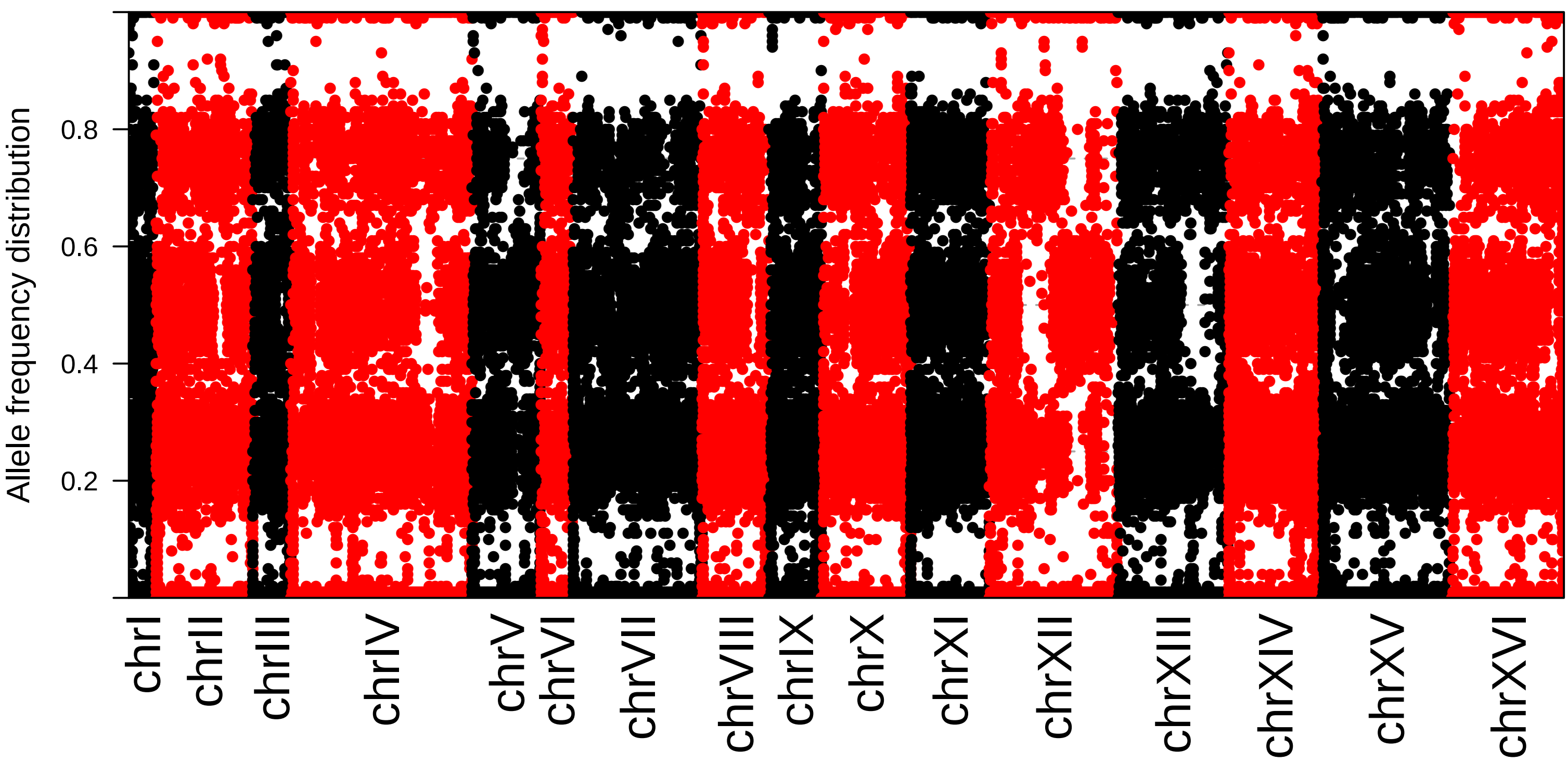

beer028

Supplementary Figure S3

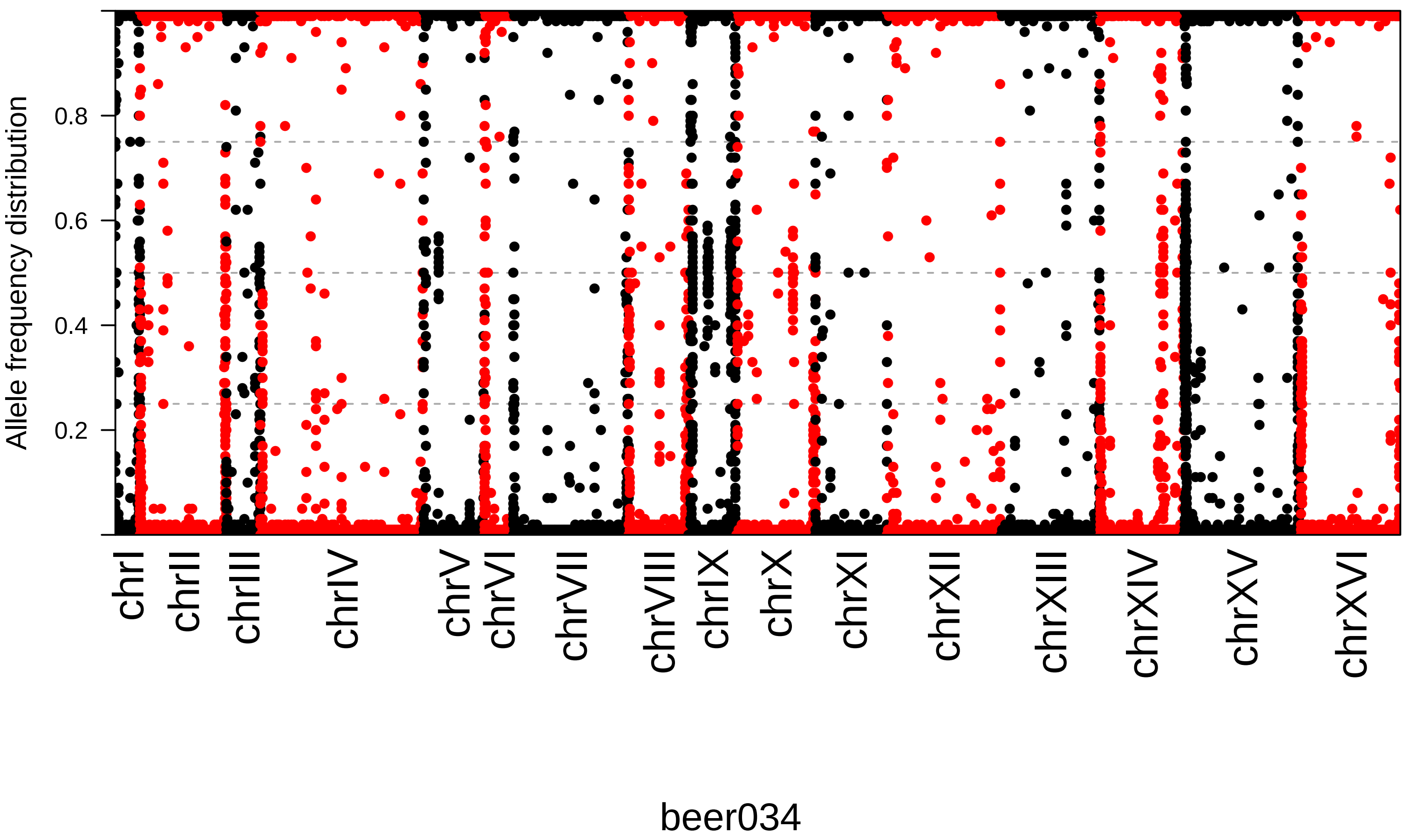

Supplementary Figure S3

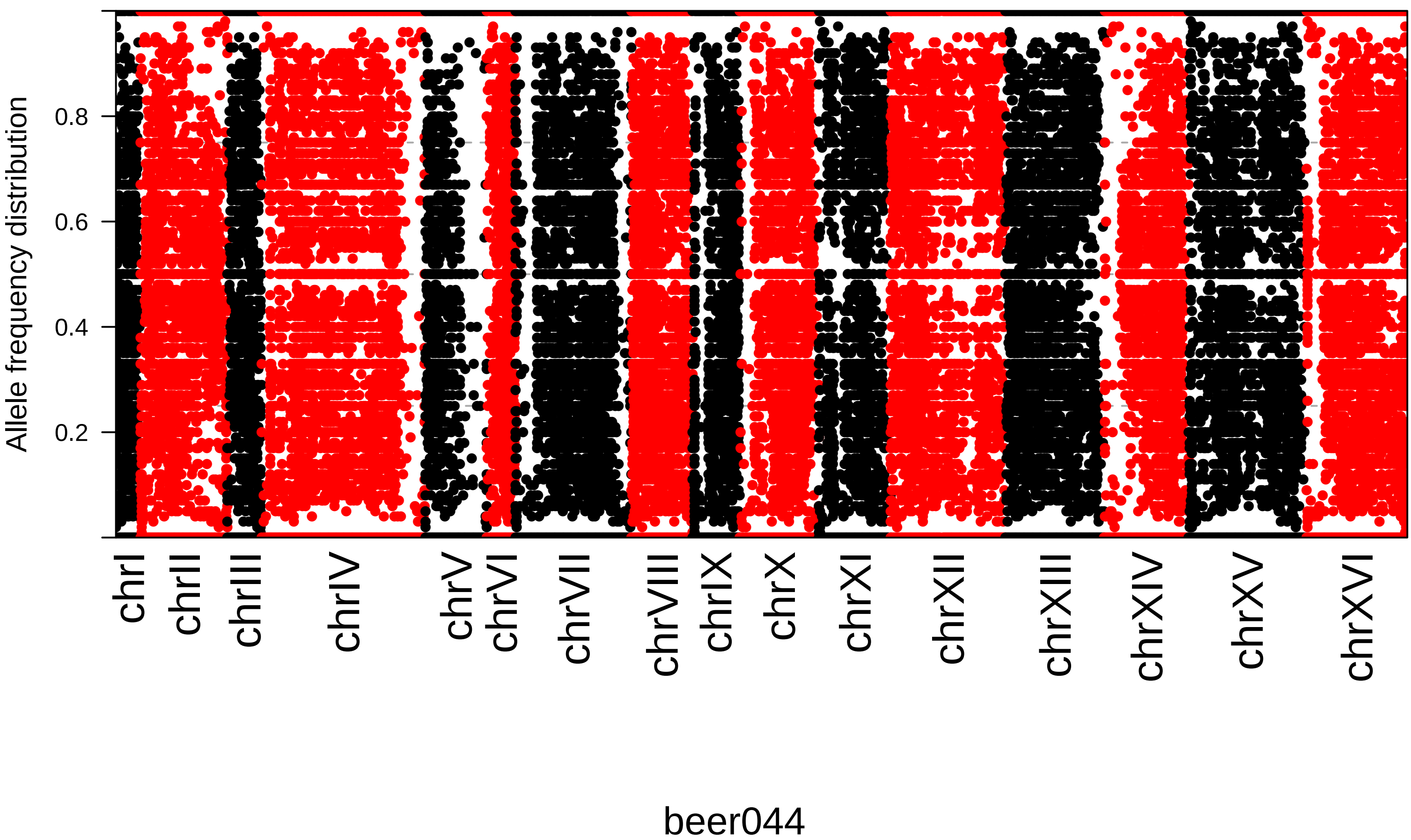

Supplementary Figure S3

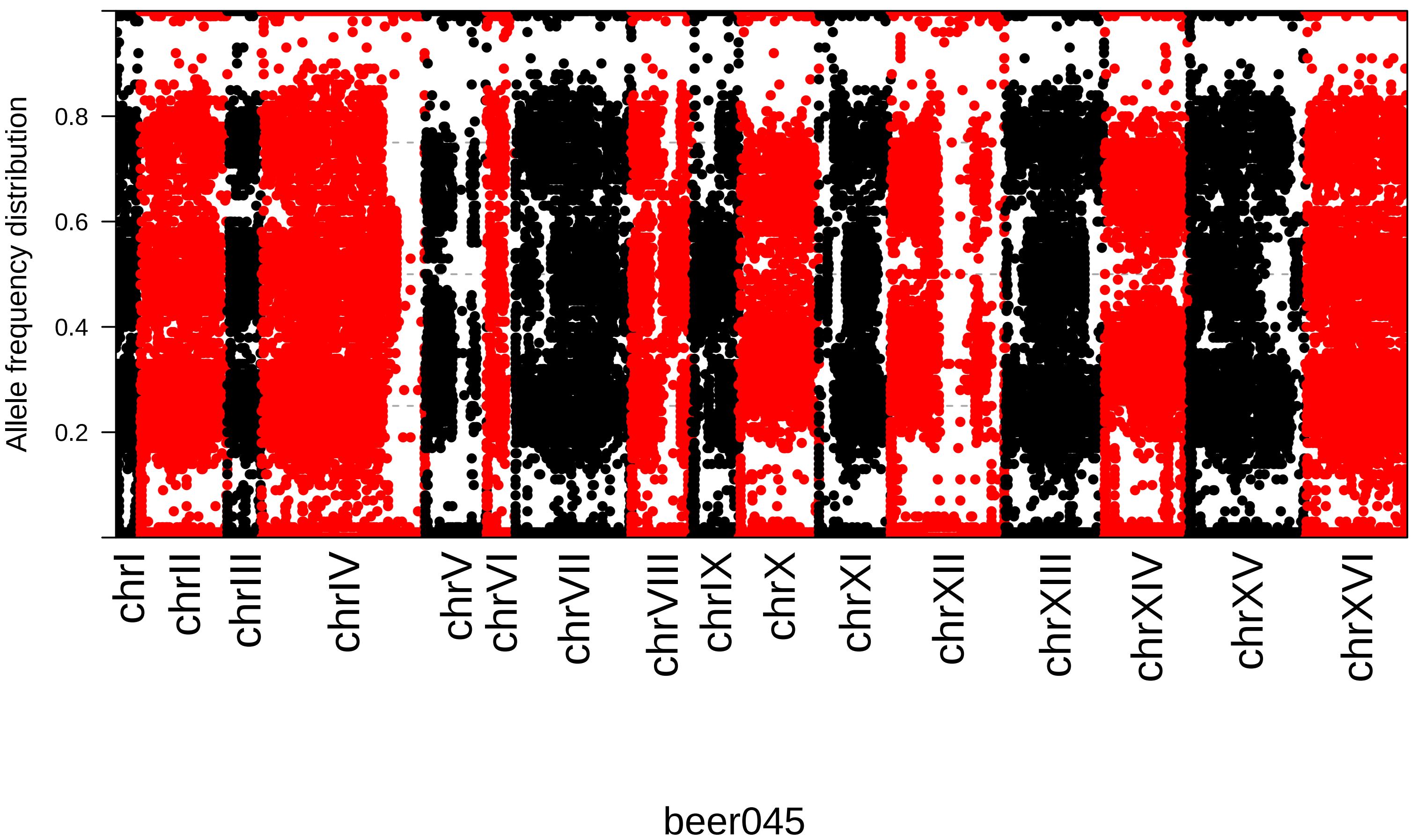

Supplementary Figure S3

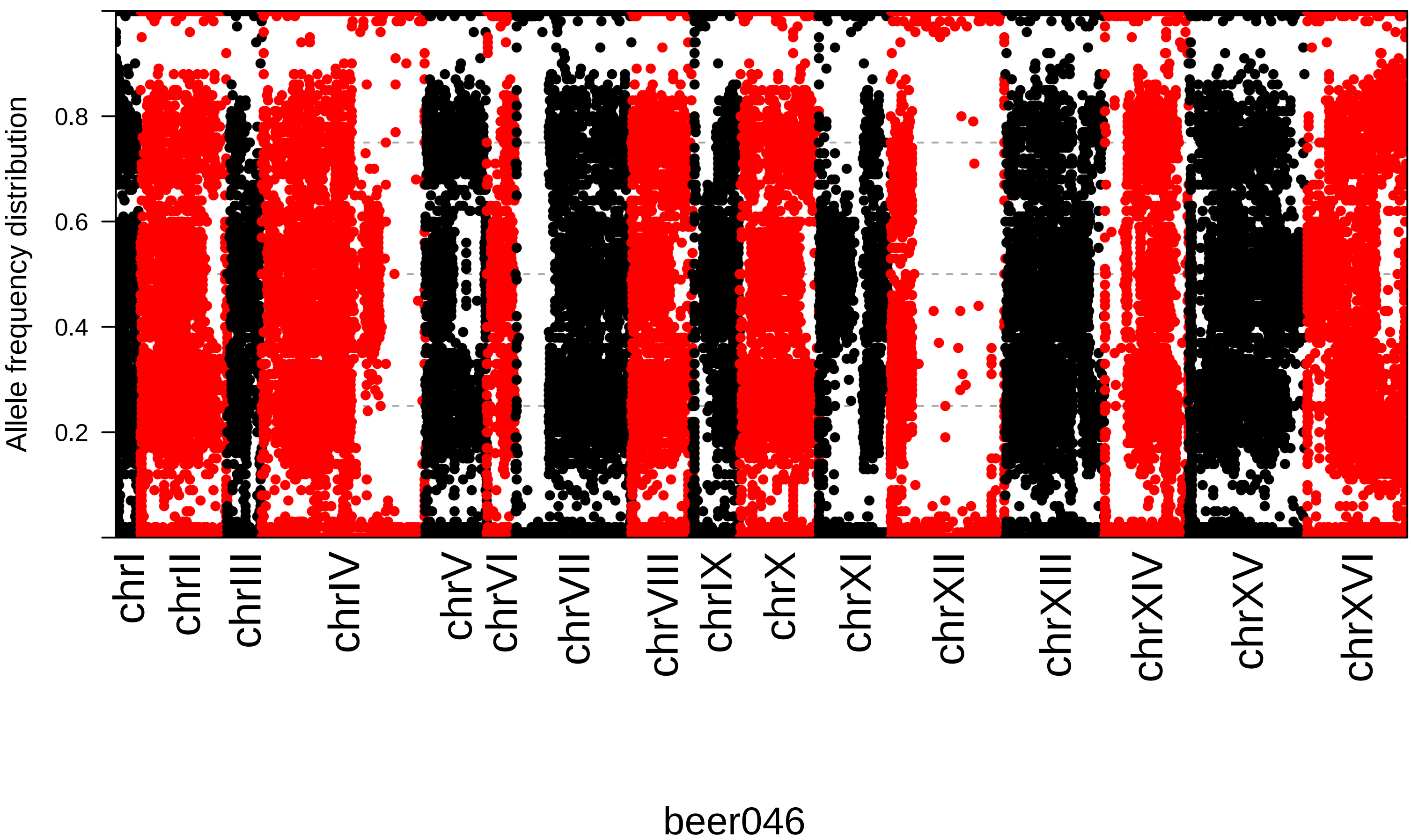

Supplementary Figure S3

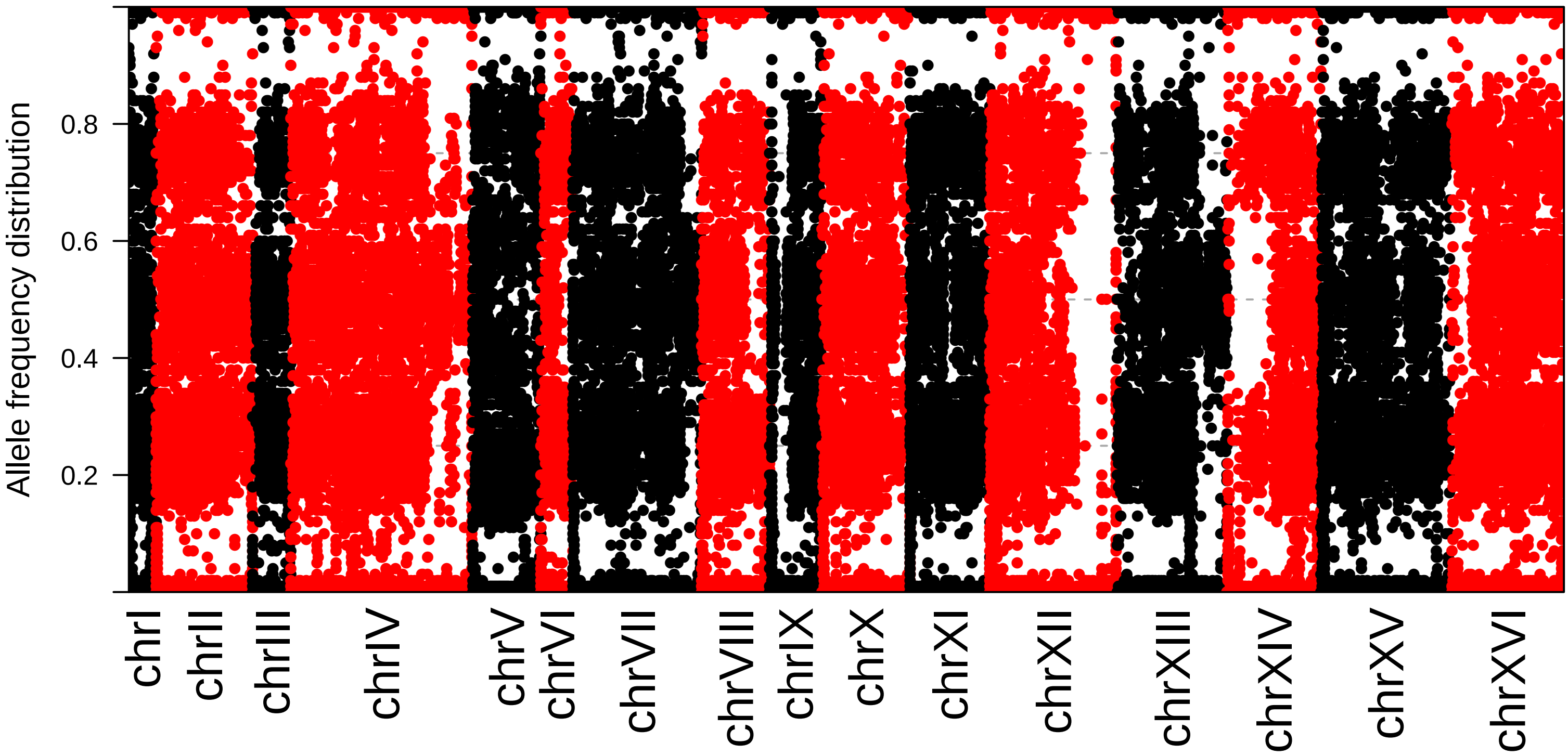

beer079

Supplementary Figure S3

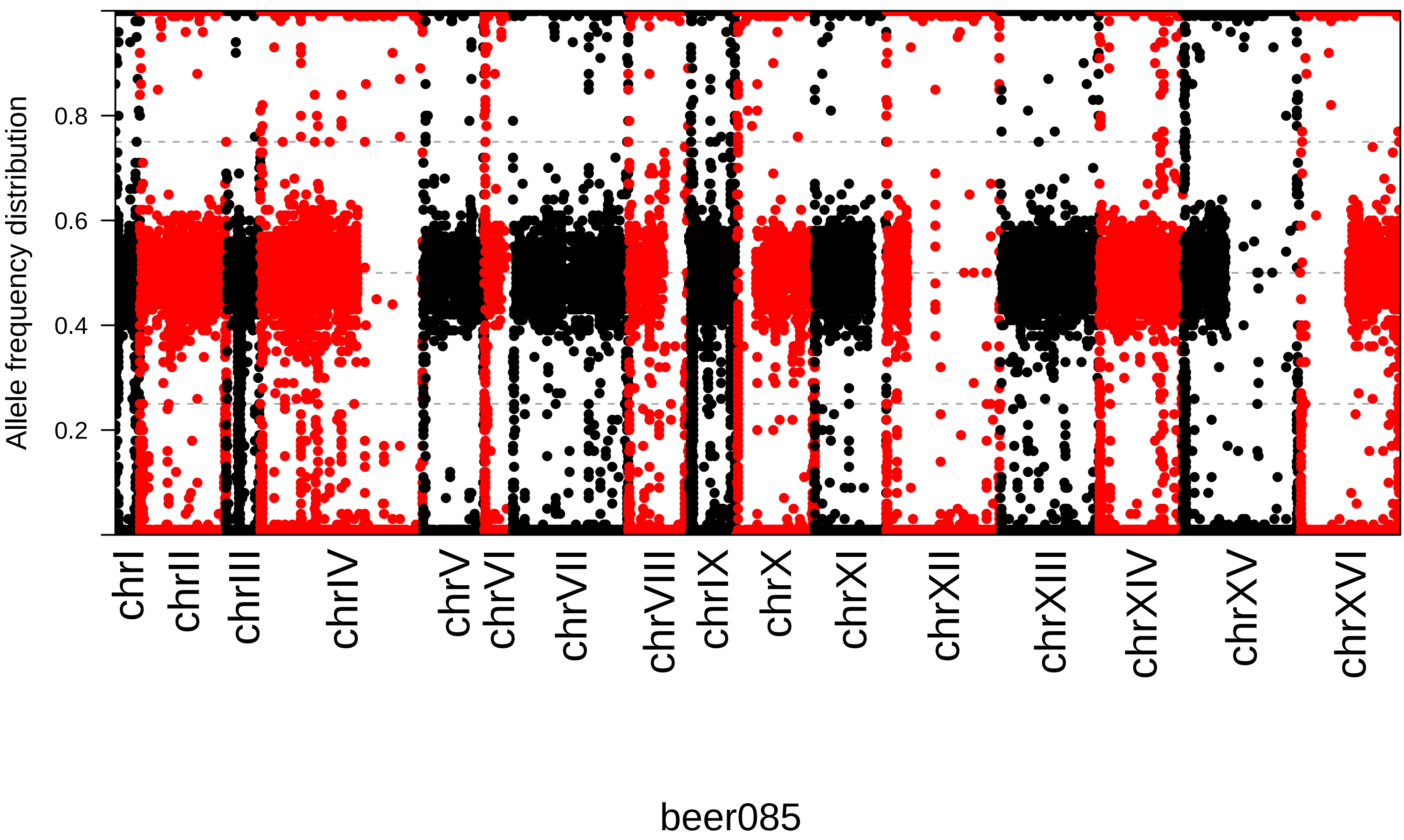

Supplementary Figure S3

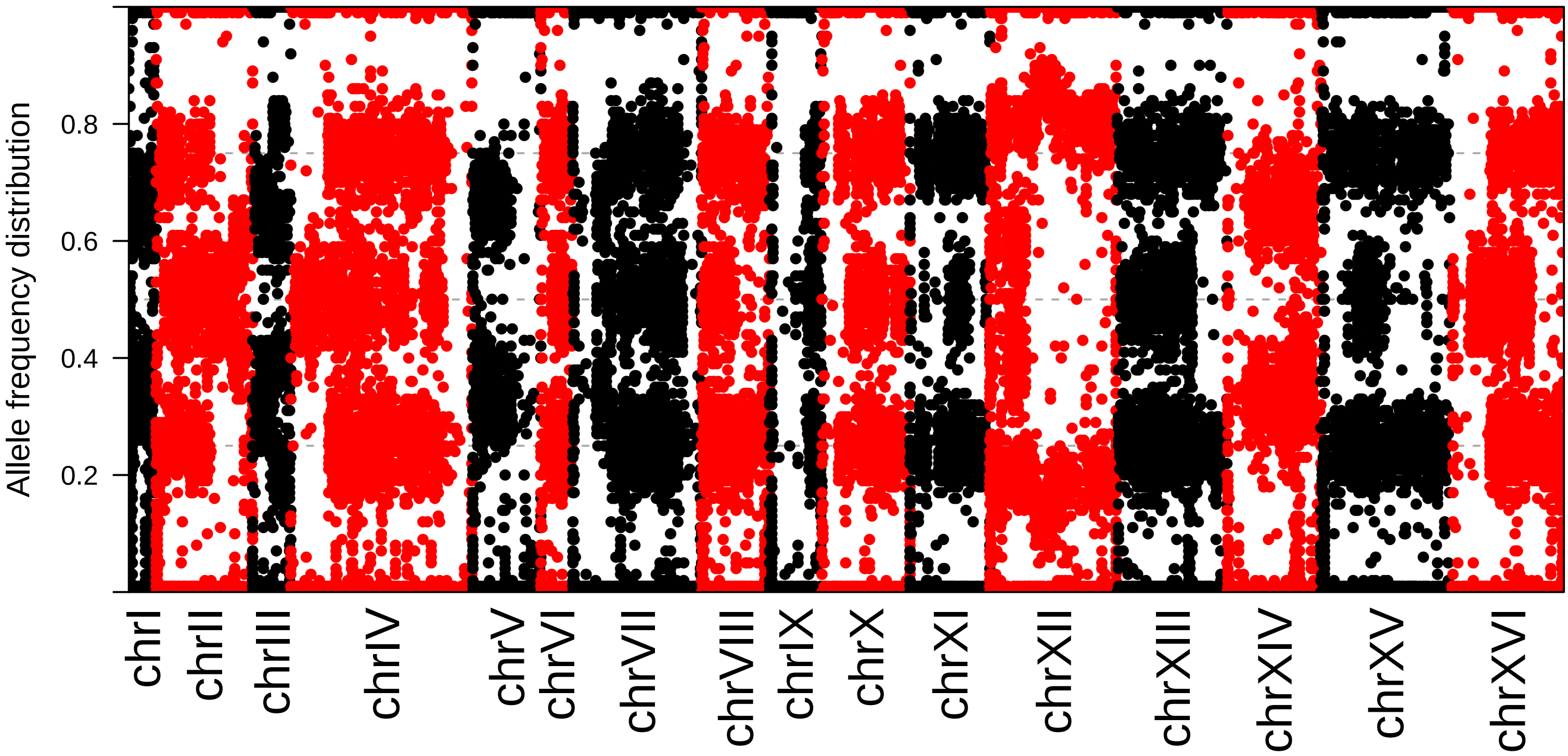

beer098

Supplementary Figure S3

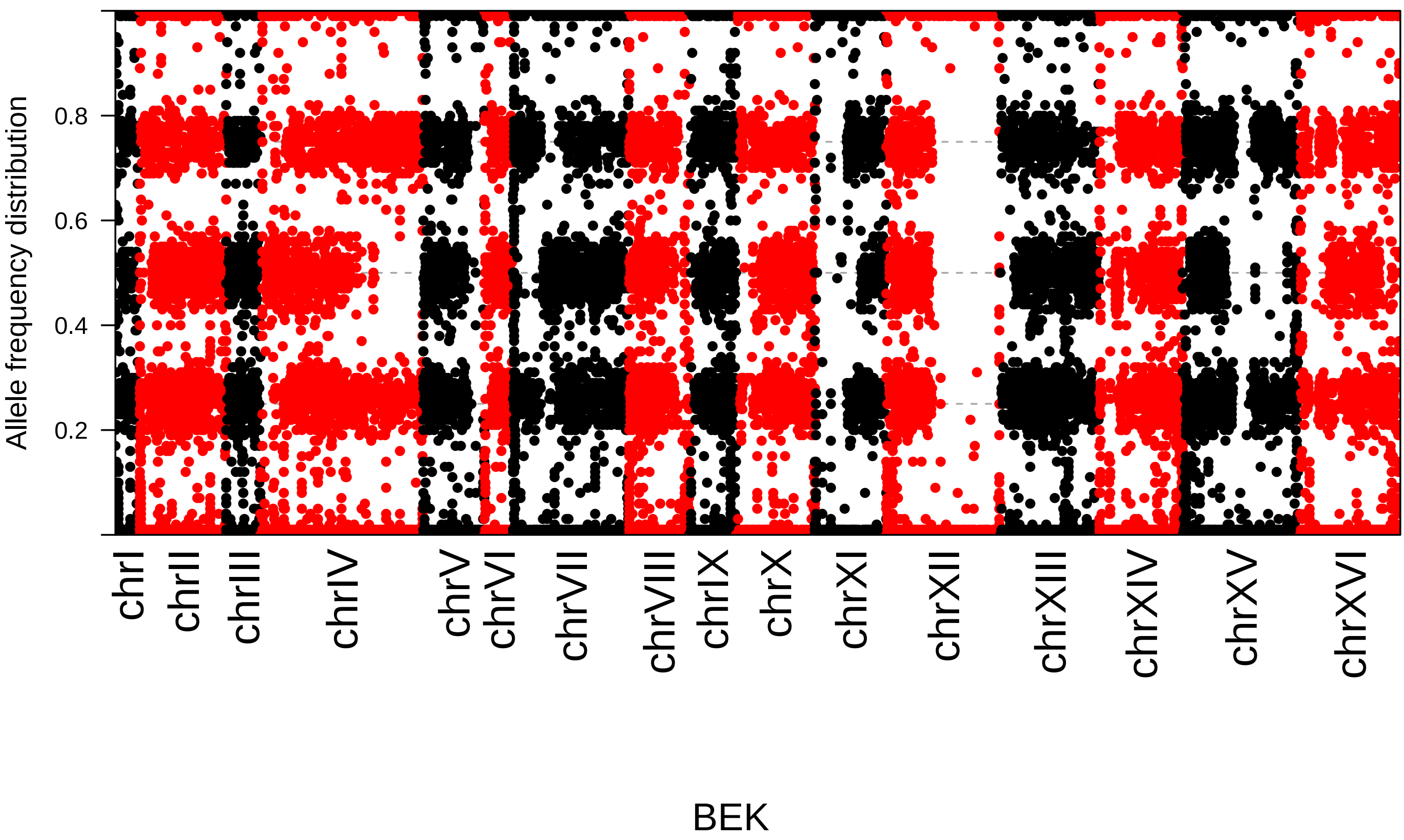

Supplementary Figure S3

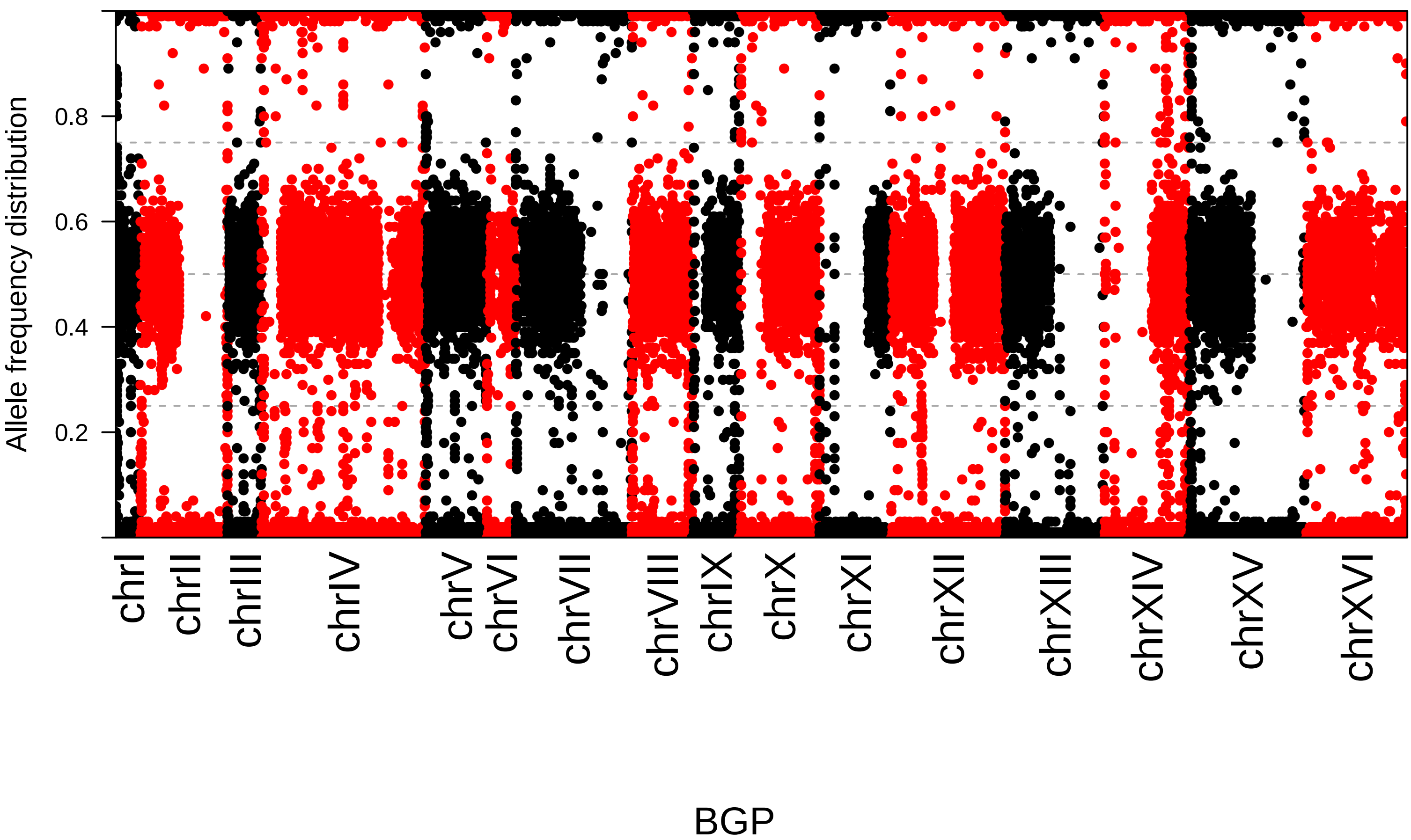

Supplementary Figure S3

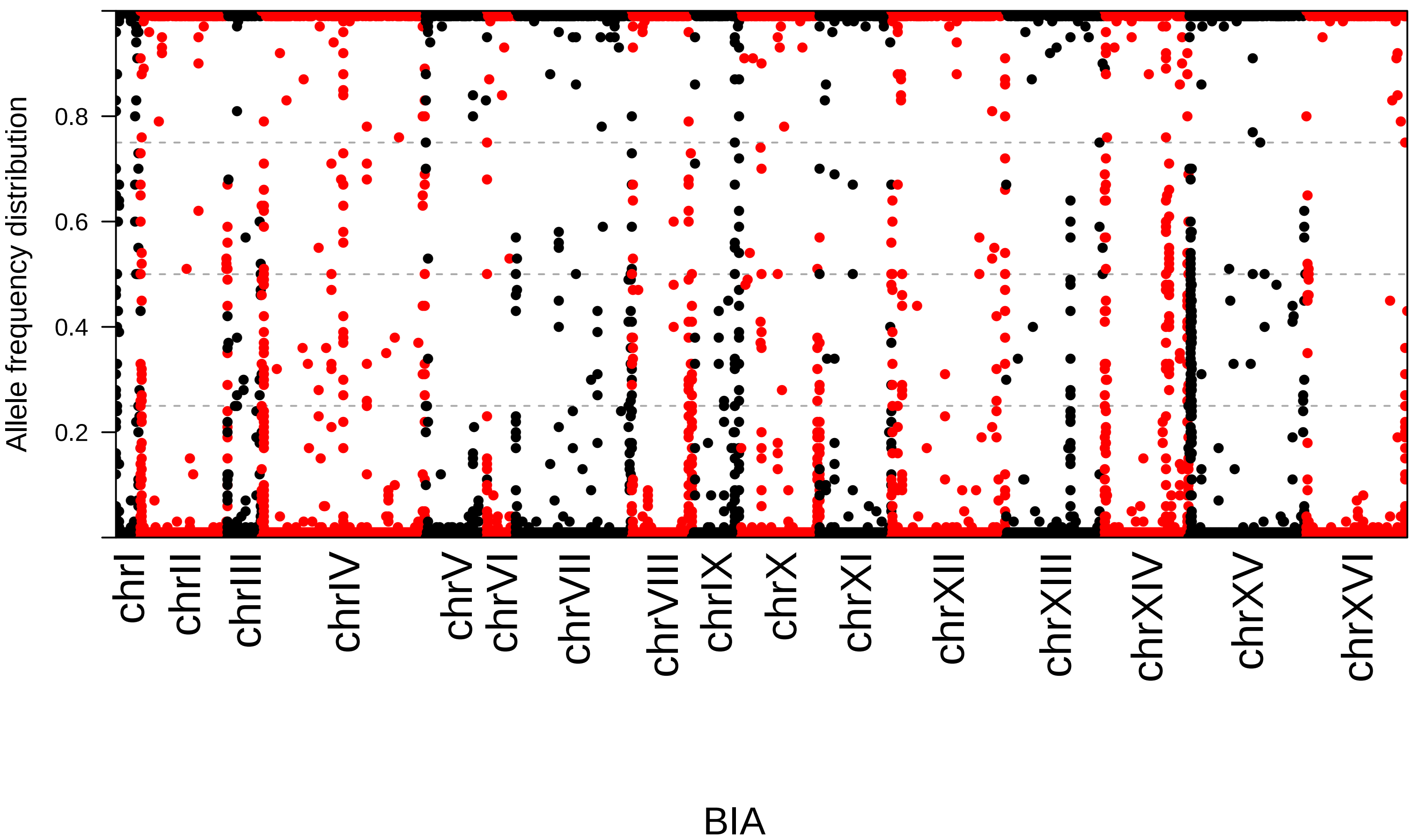

Supplementary Figure S3

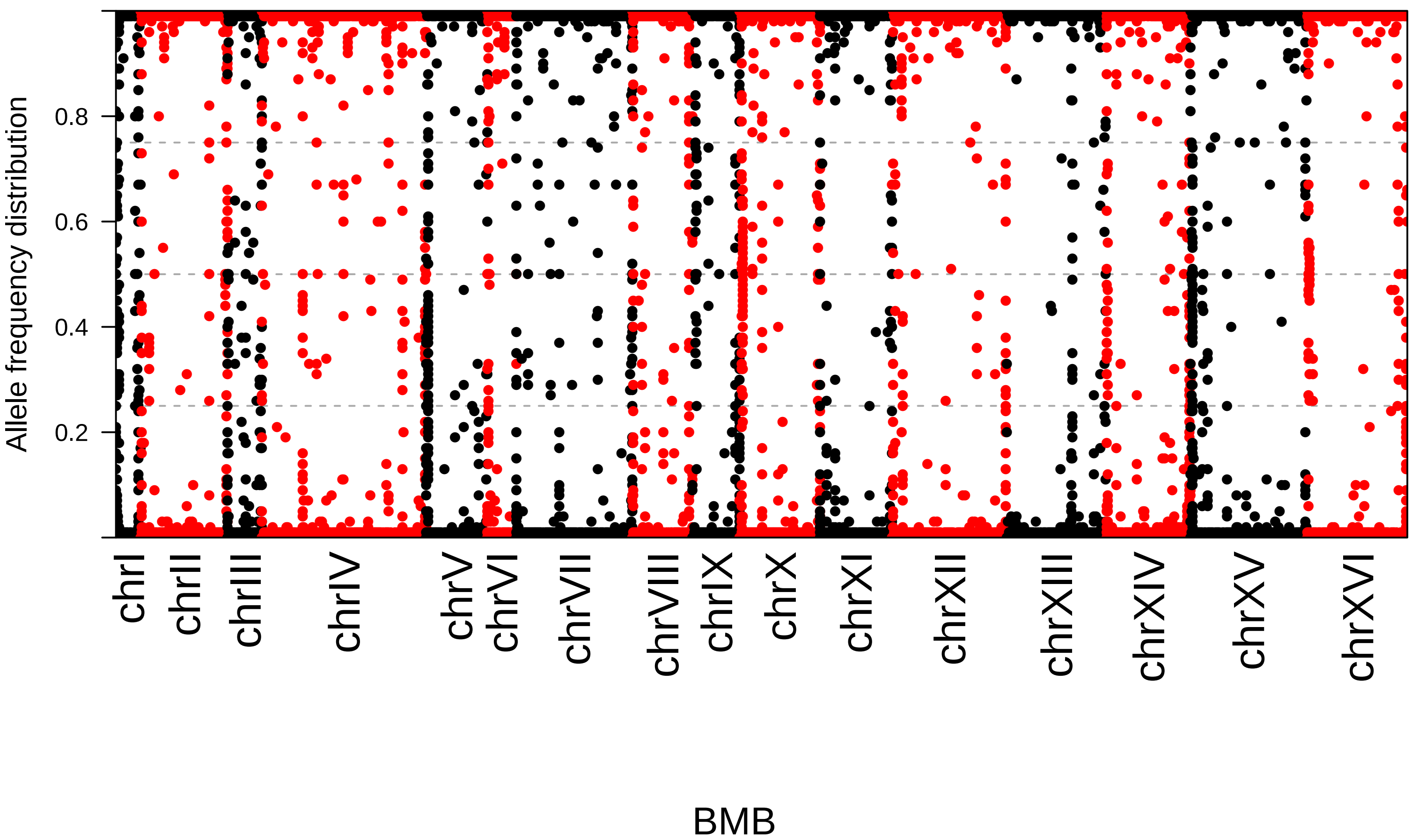

Supplementary Figure S3

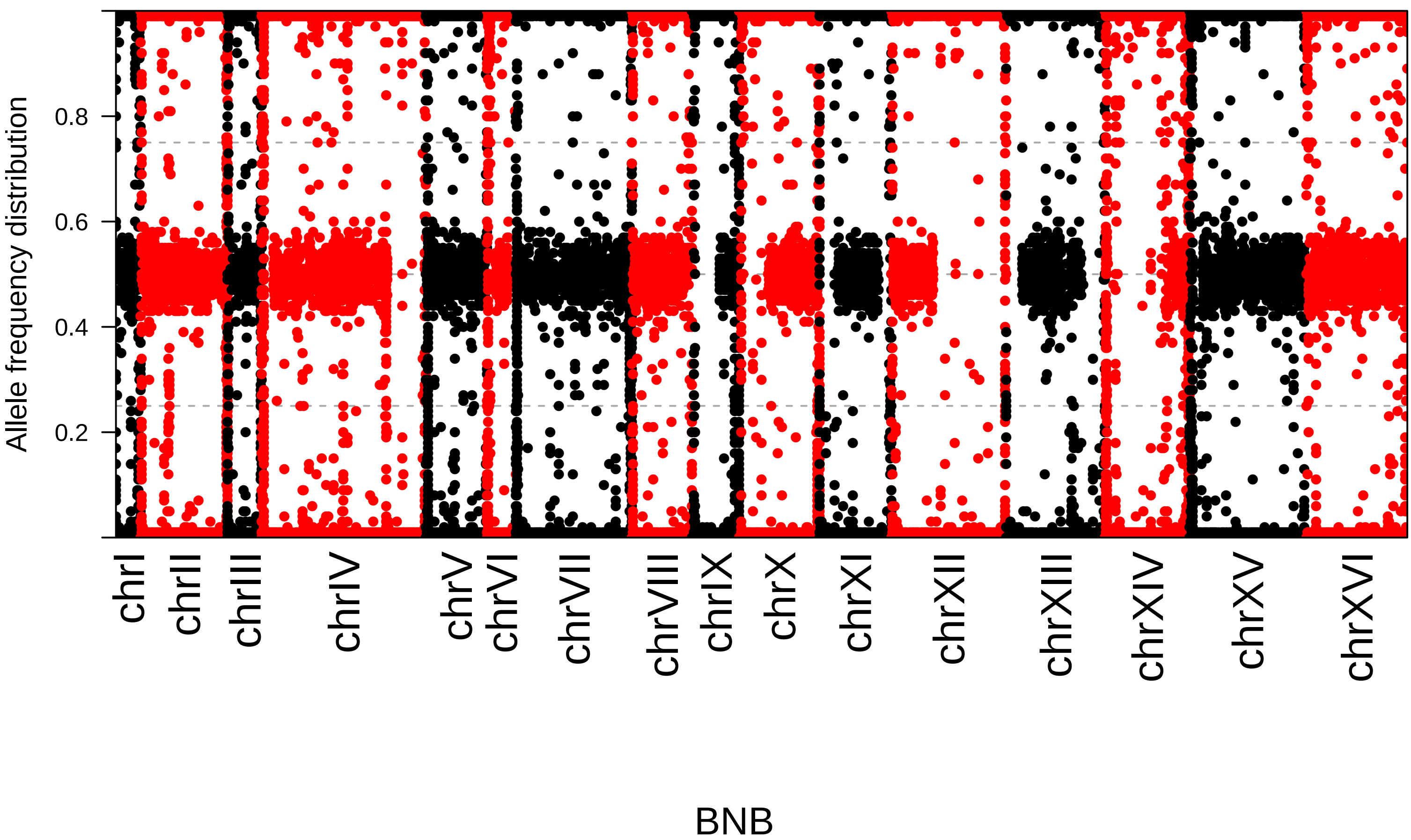

Supplementary Figure S3

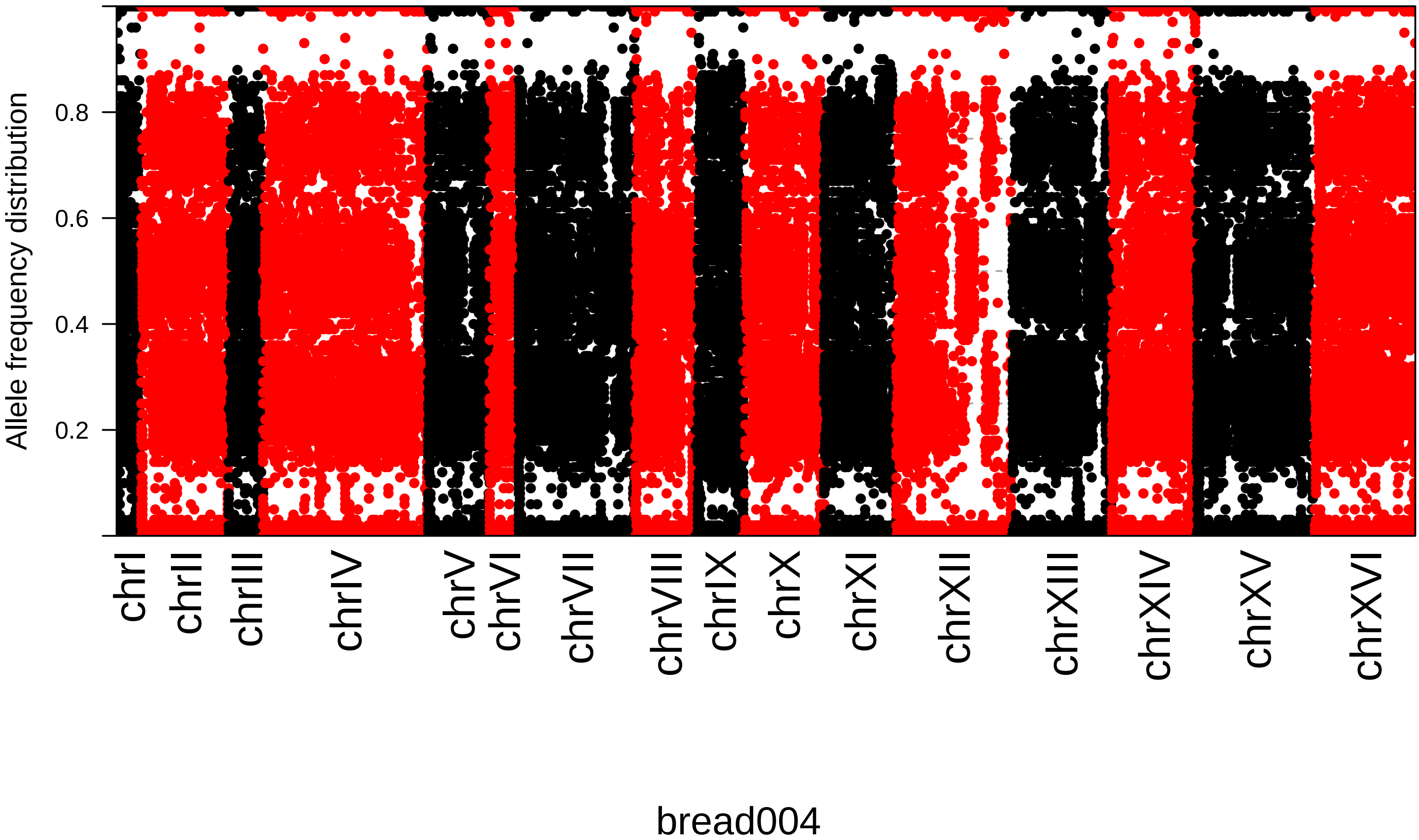

Supplementary Figure S3

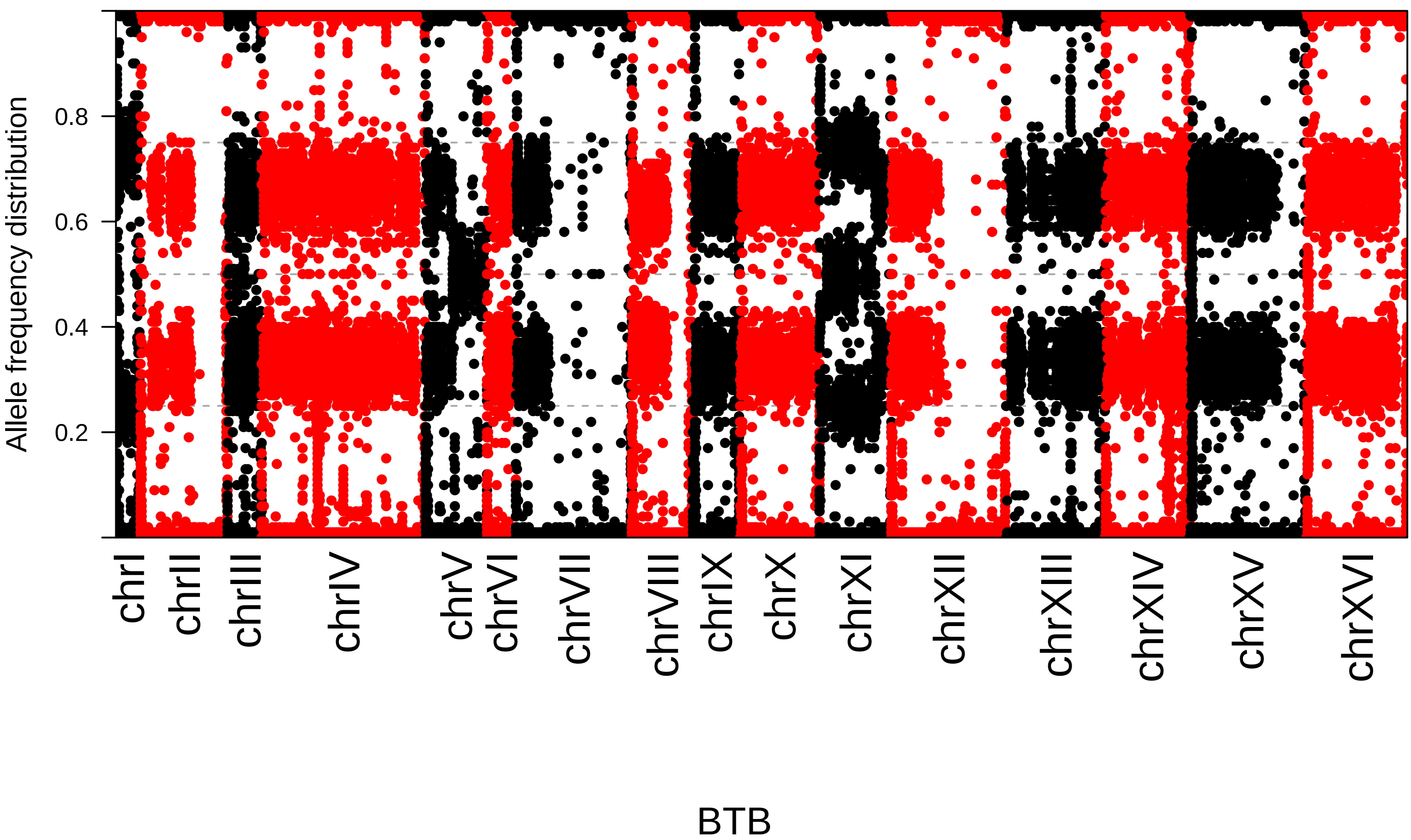

Supplementary Figure S3

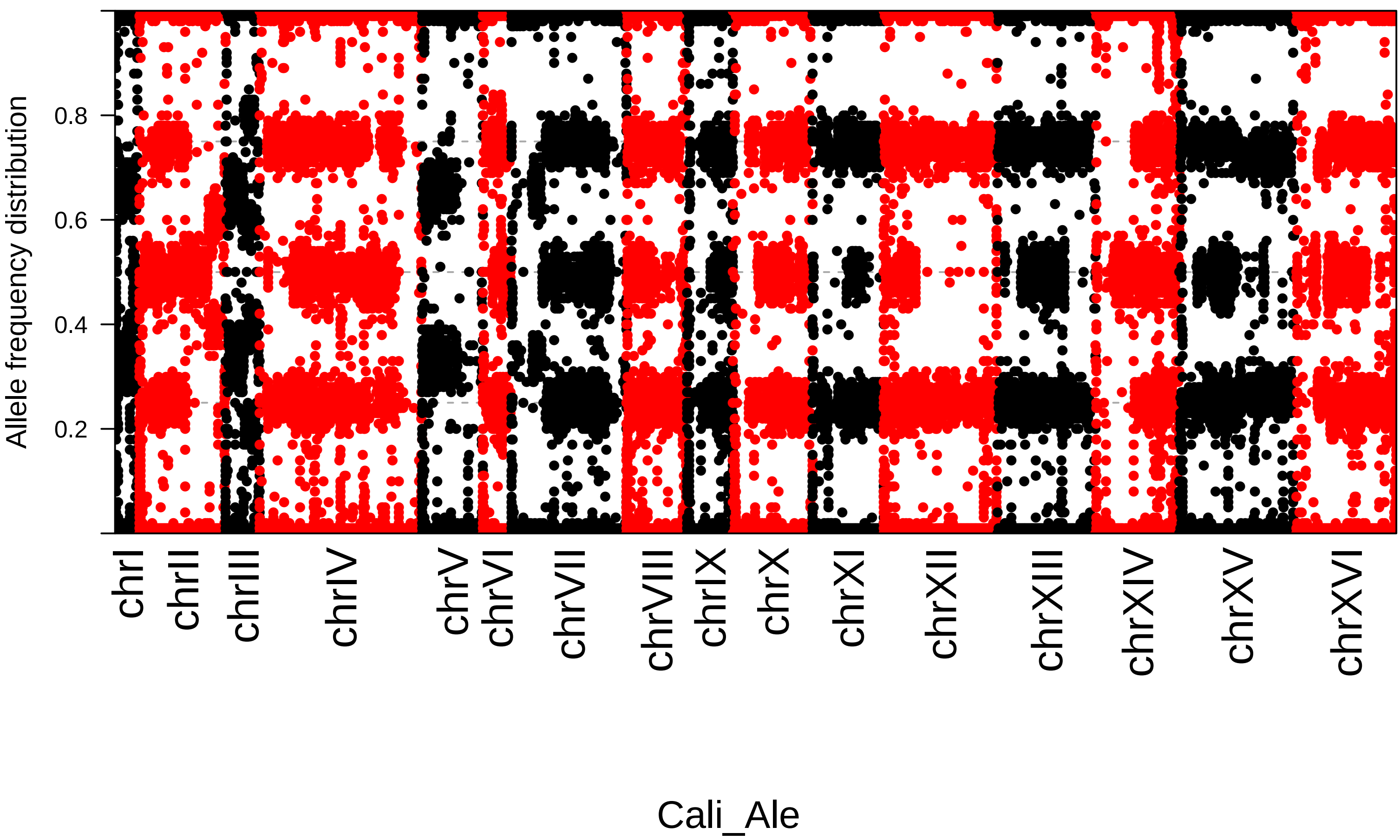

Supplementary Figure S3

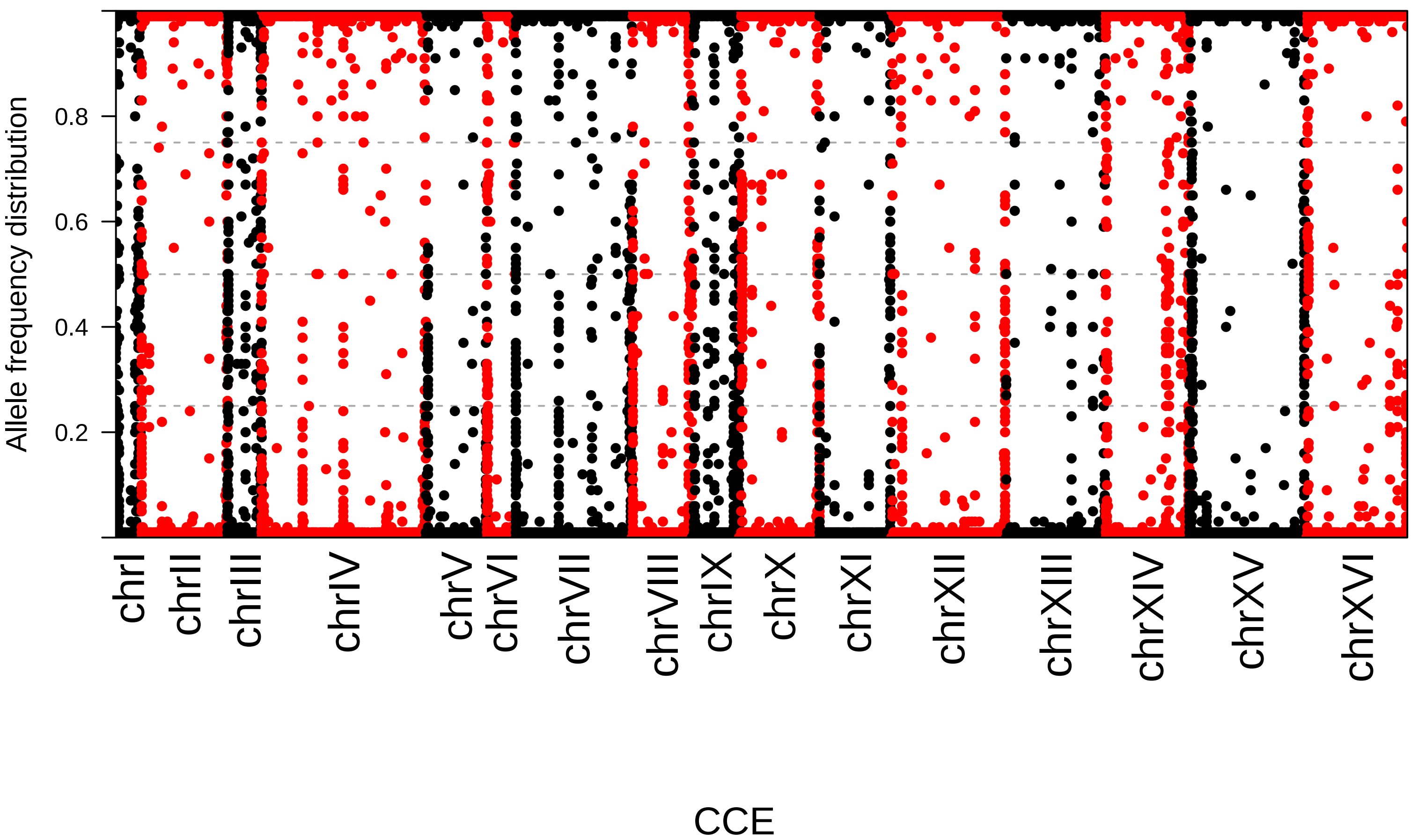

Supplementary Figure S3

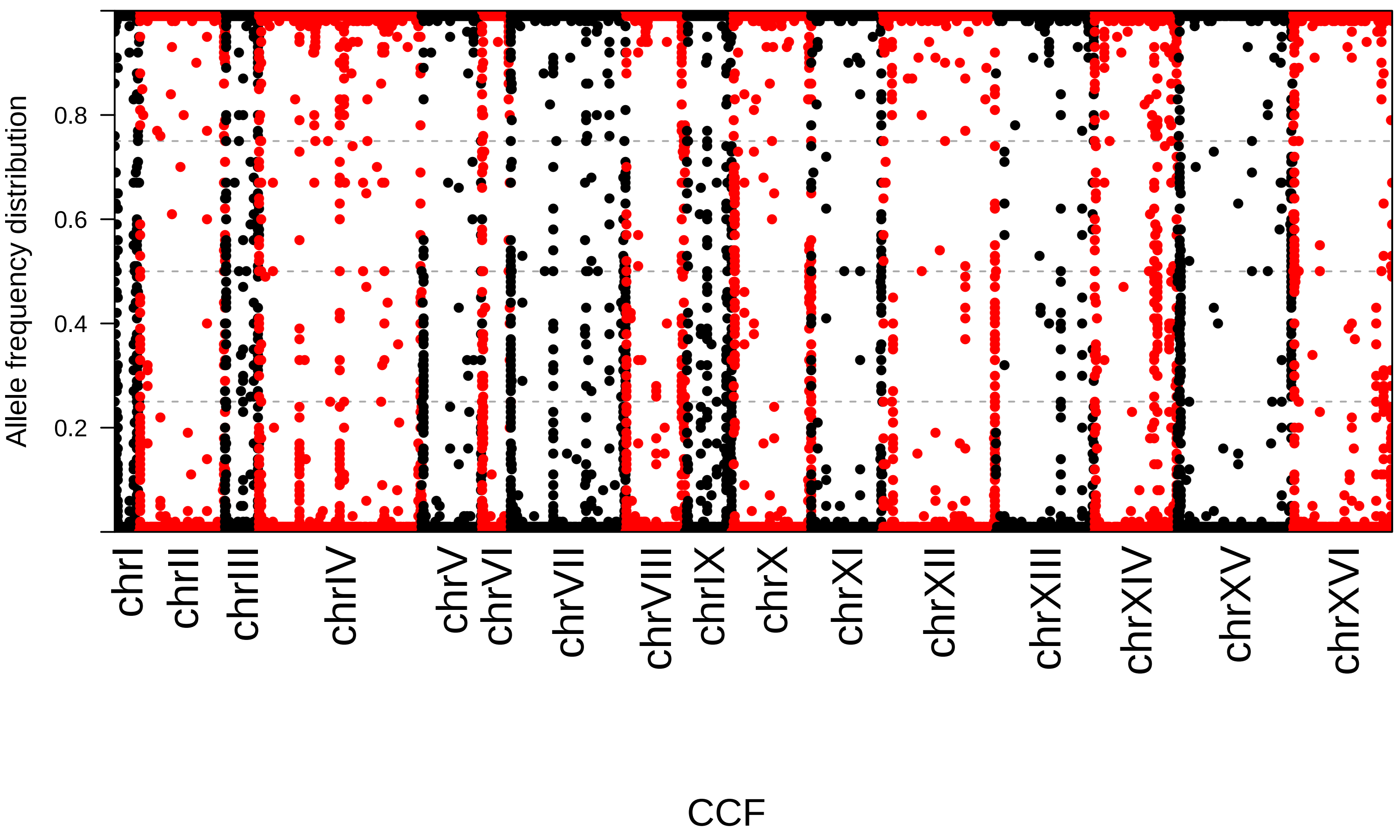

Supplementary Figure S3

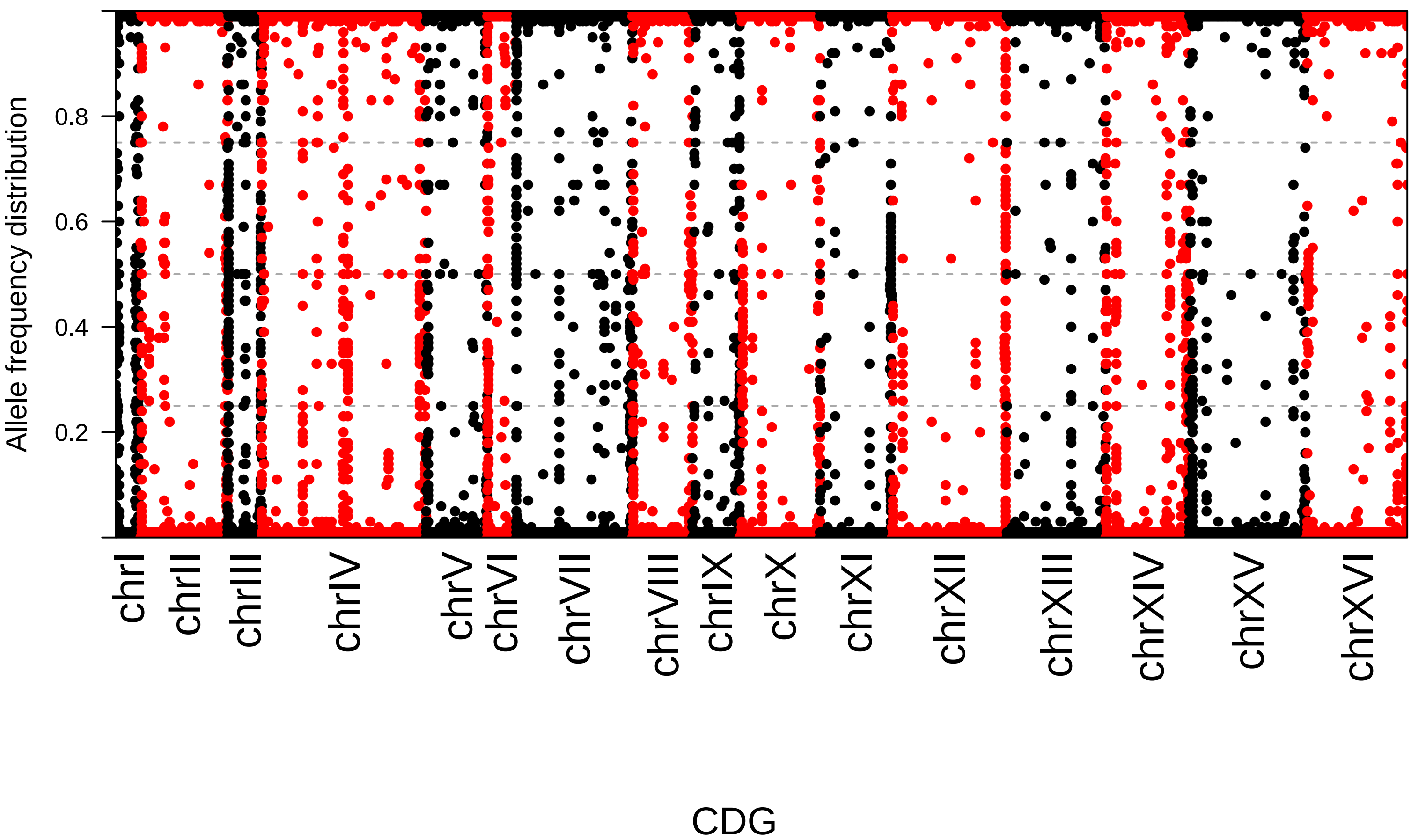

Supplementary Figure S3

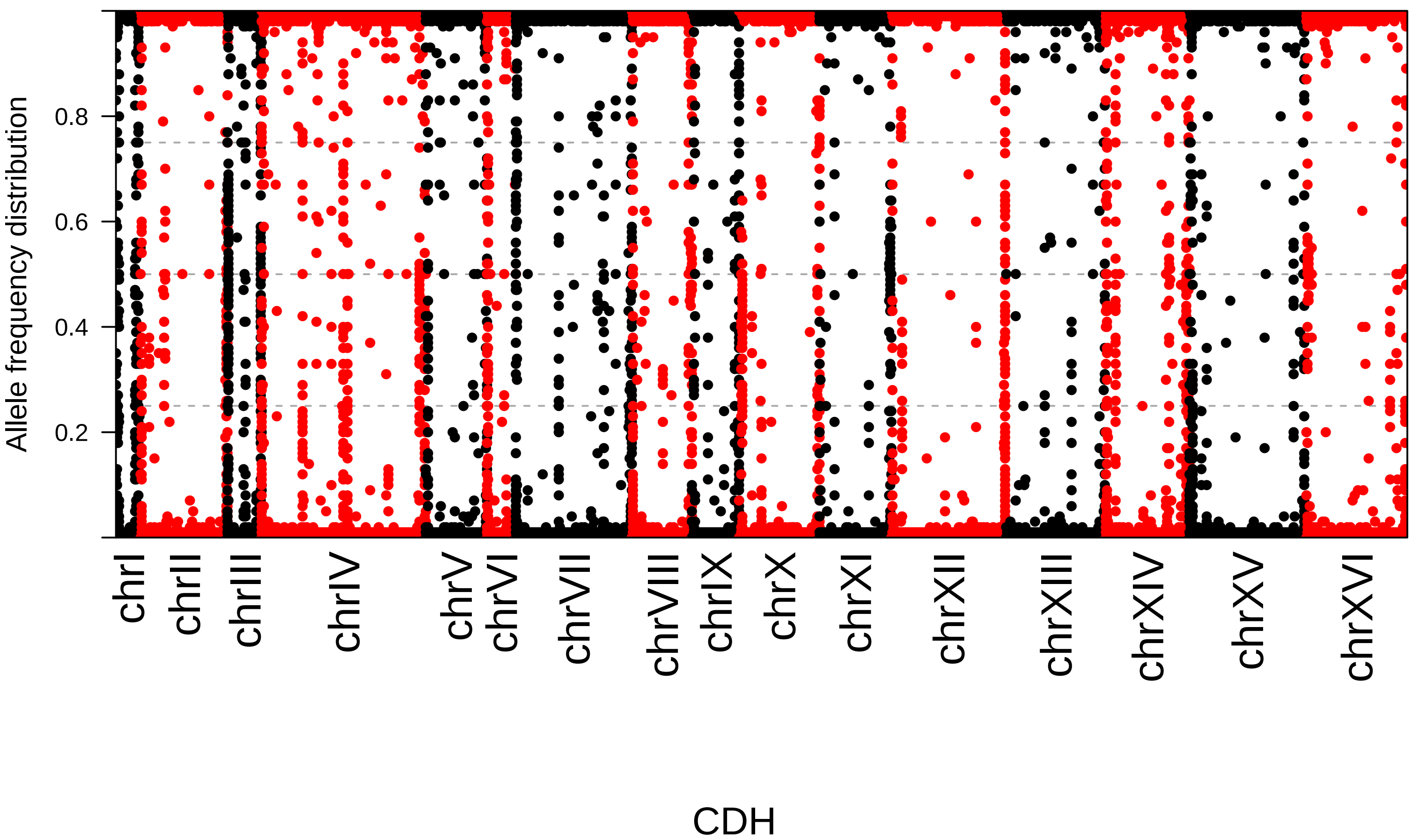

Supplementary Figure S3

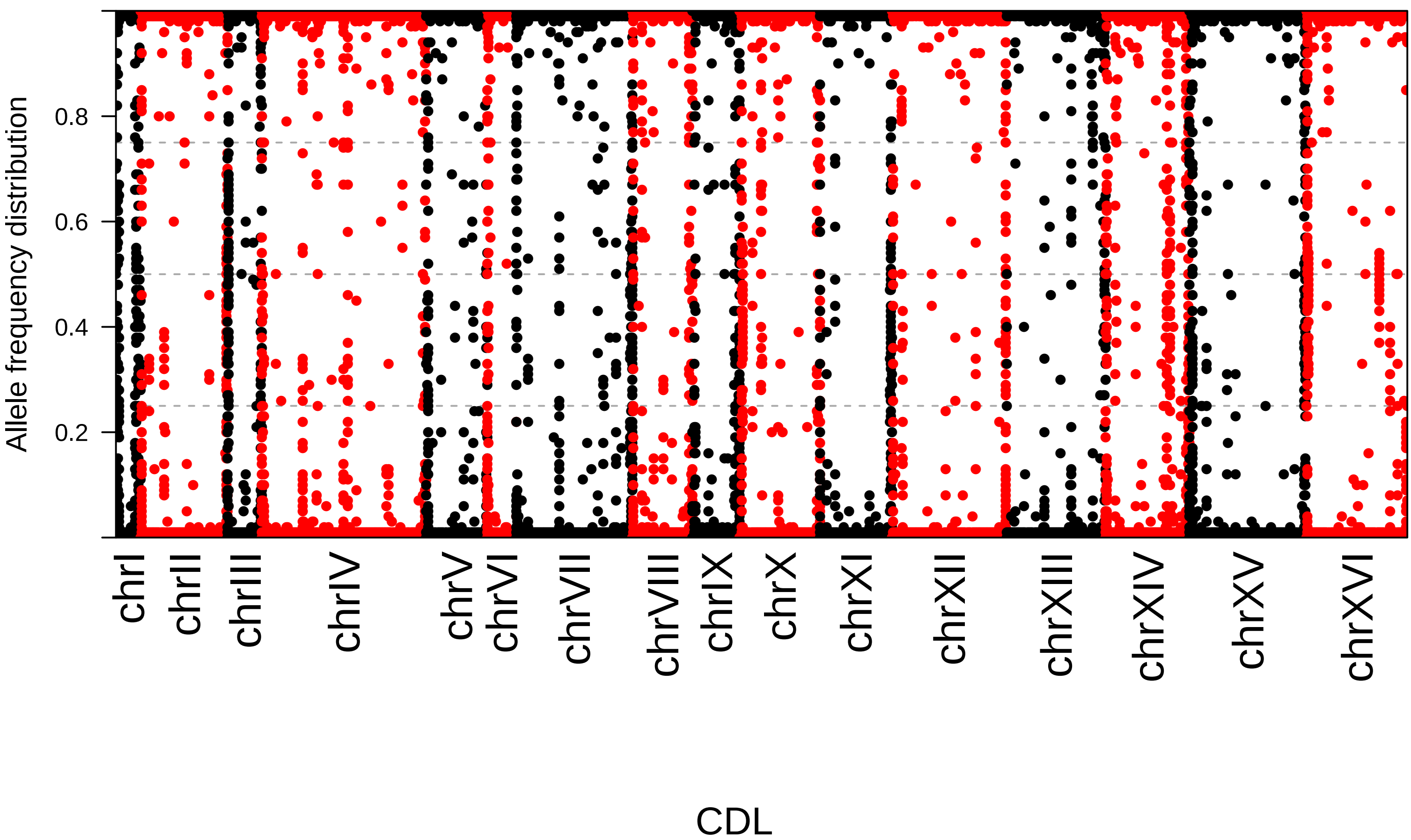

Supplementary Figure S3

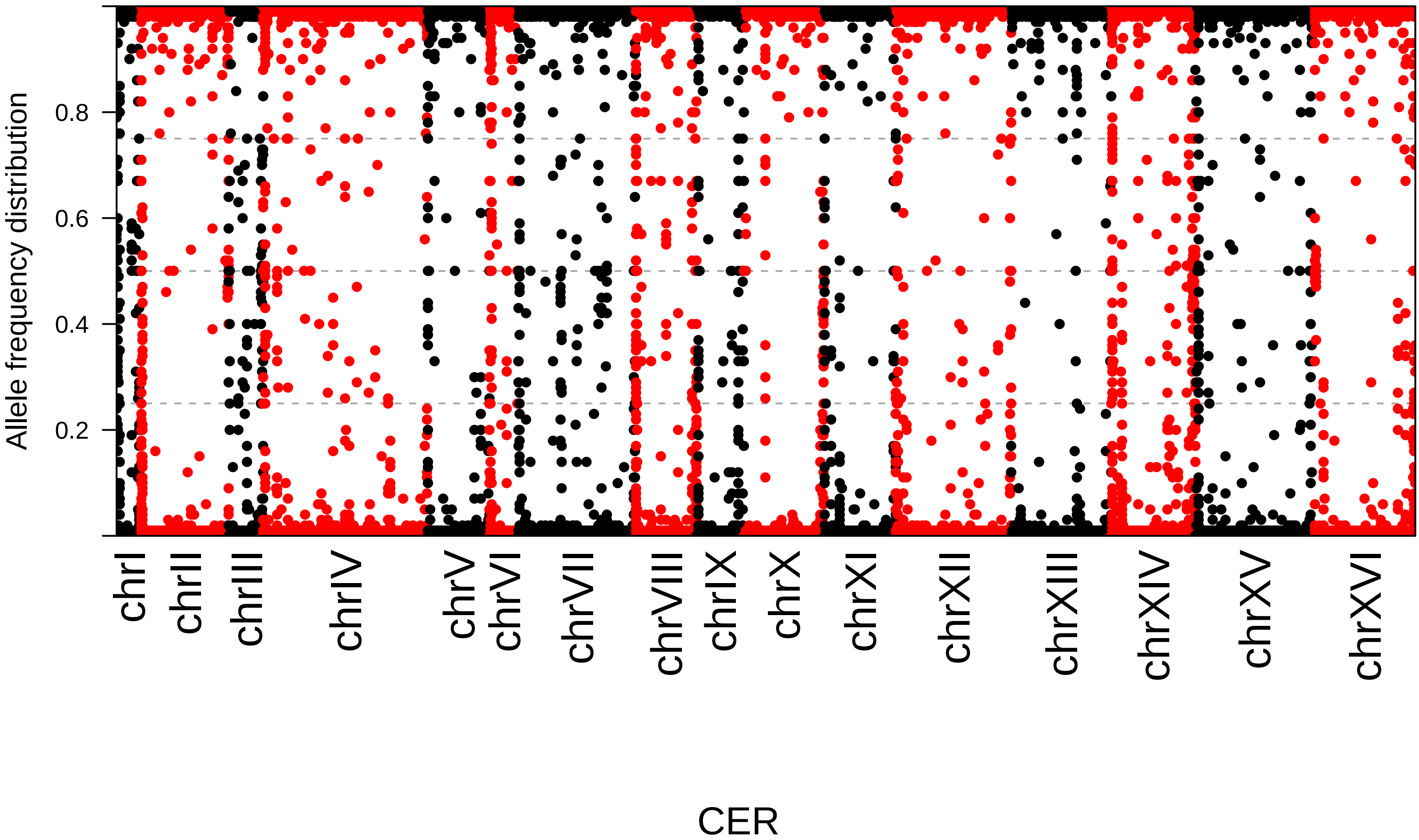

Supplementary Figure S3

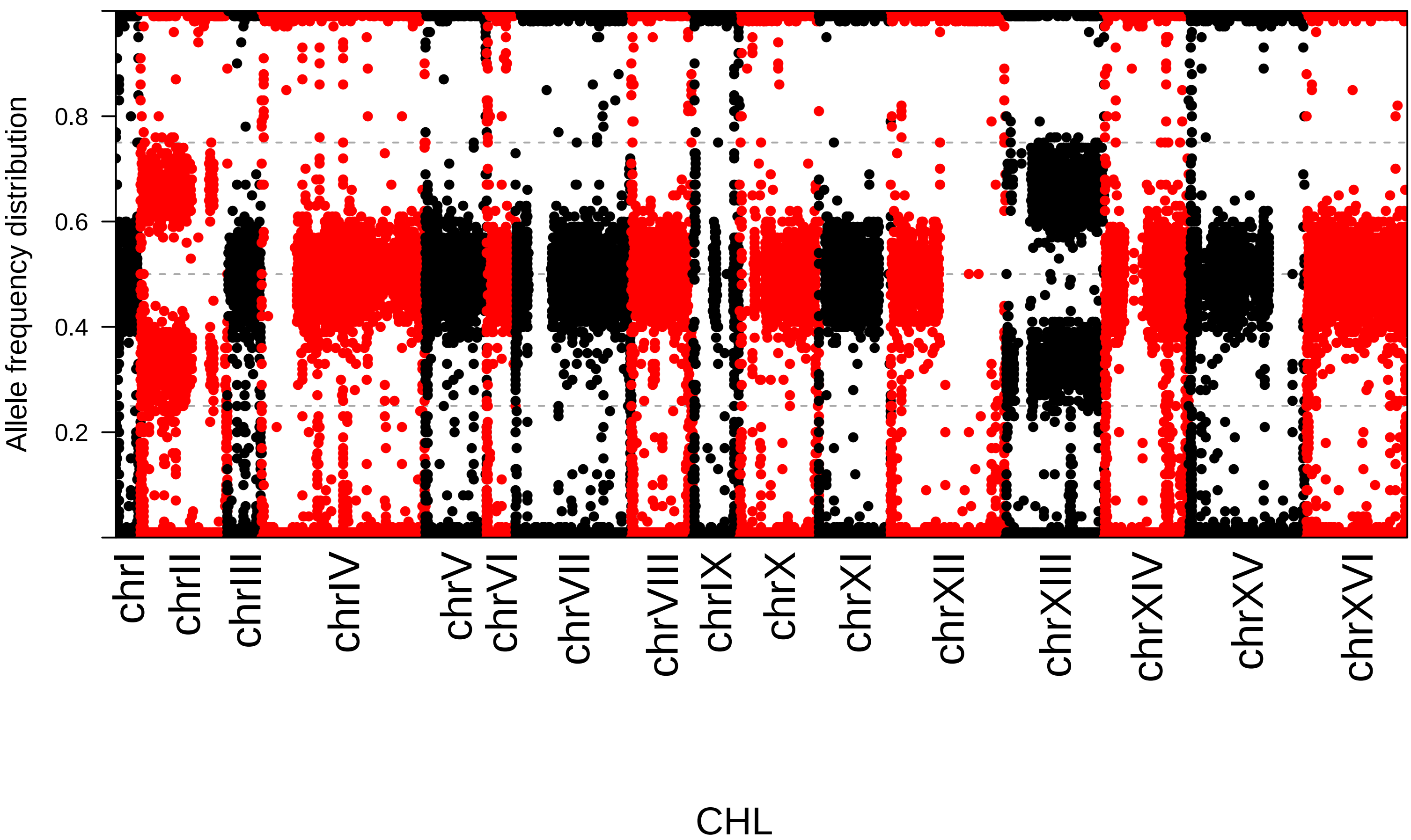

Supplementary Figure S3

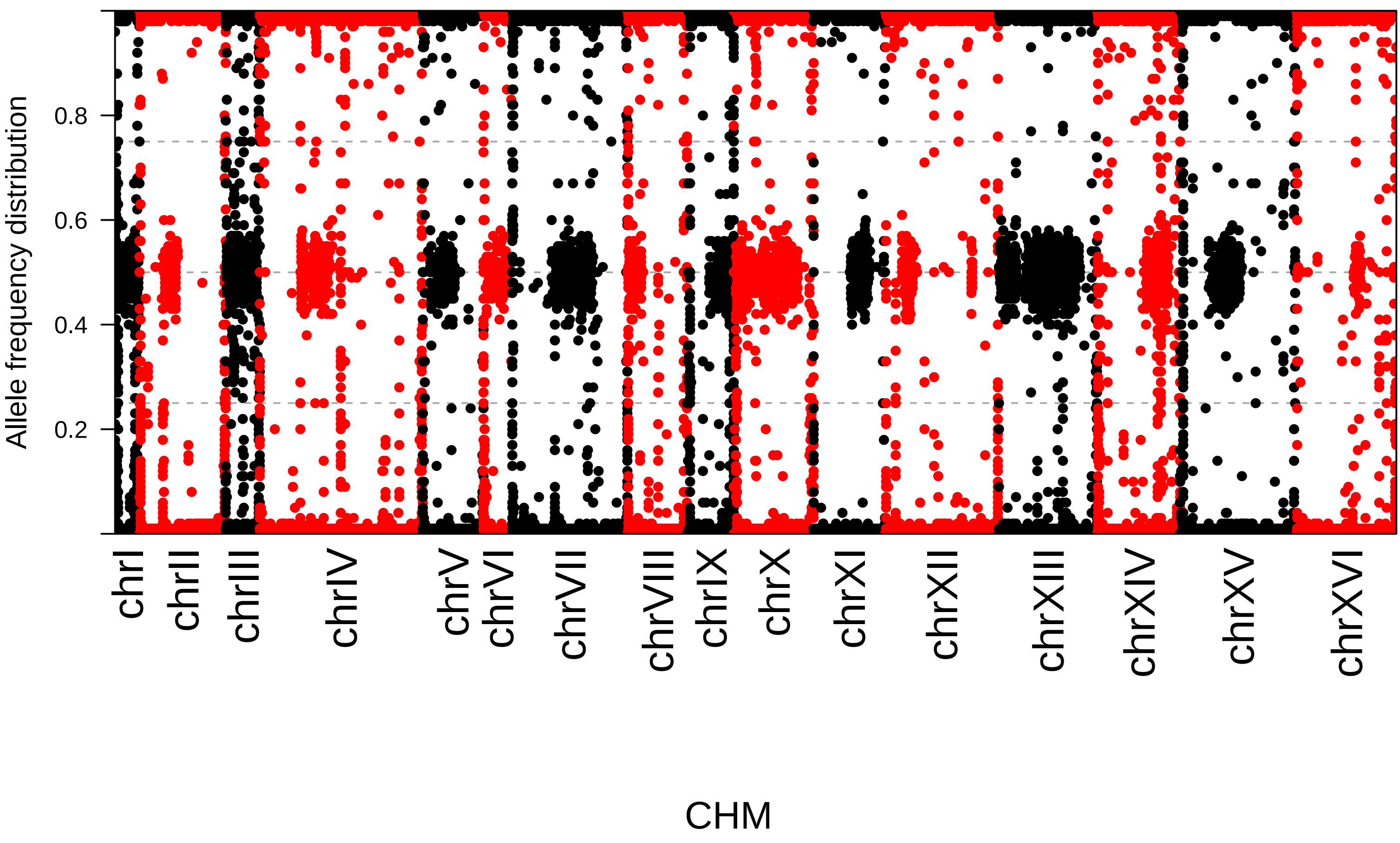

Supplementary Figure S3

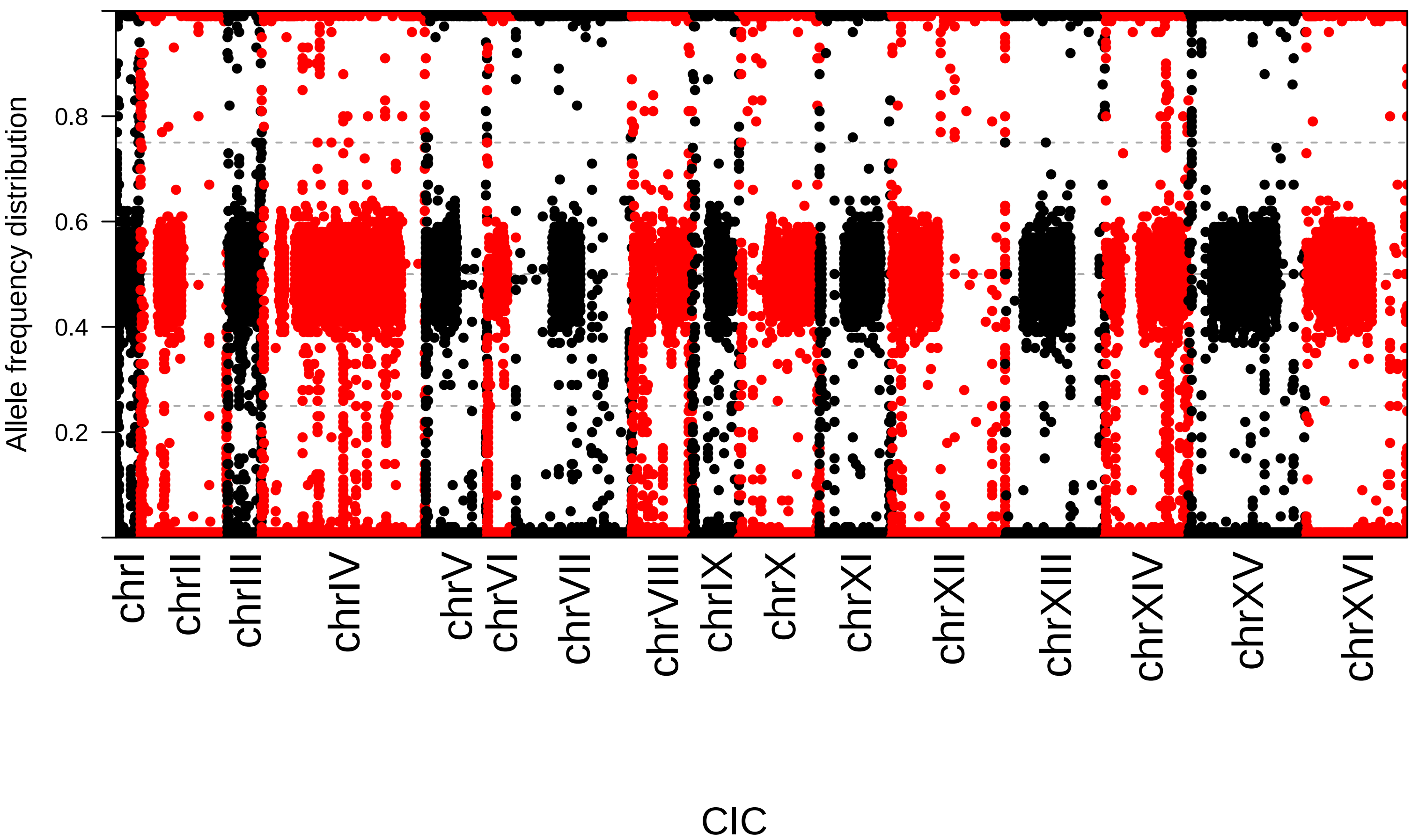

Supplementary Figure S3

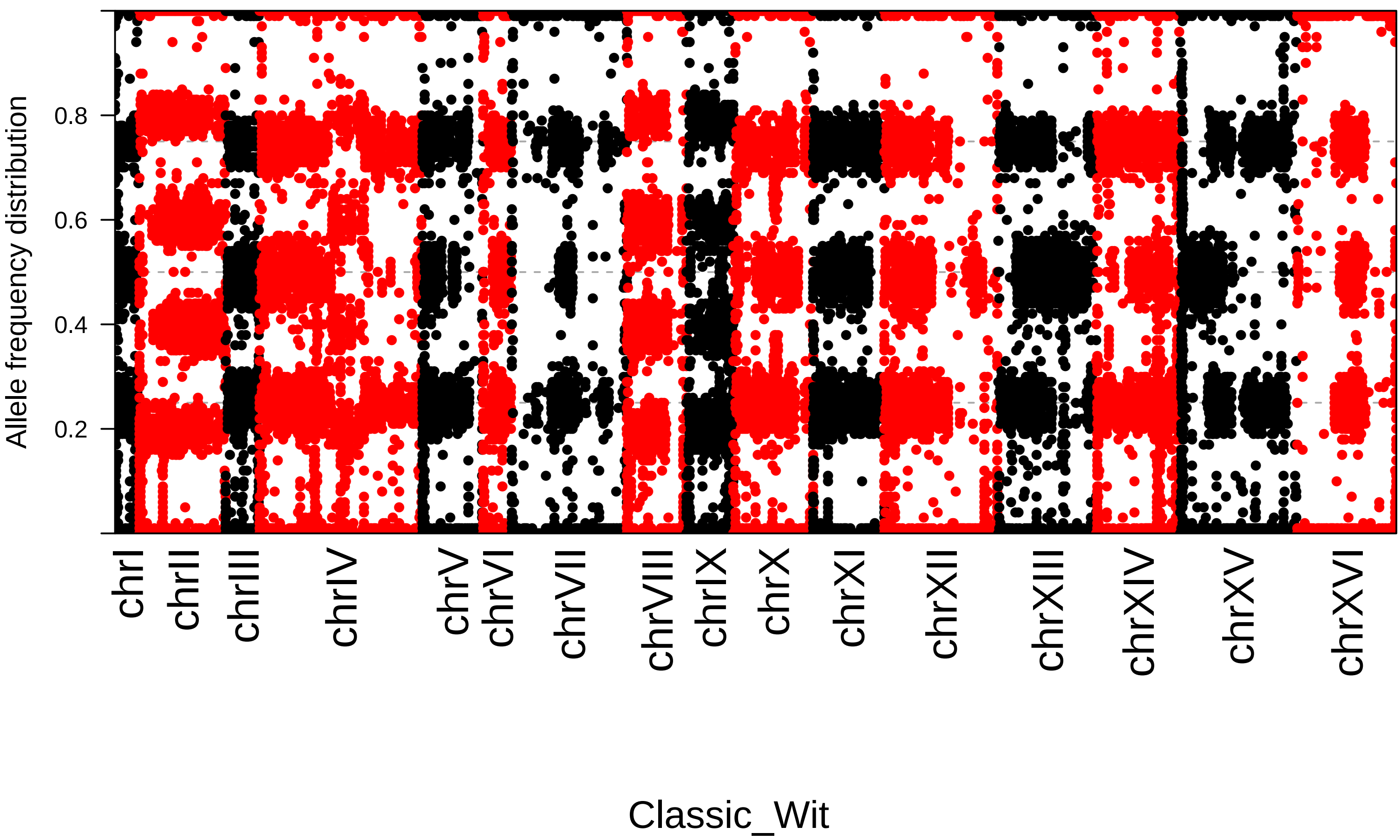

Supplementary Figure S3

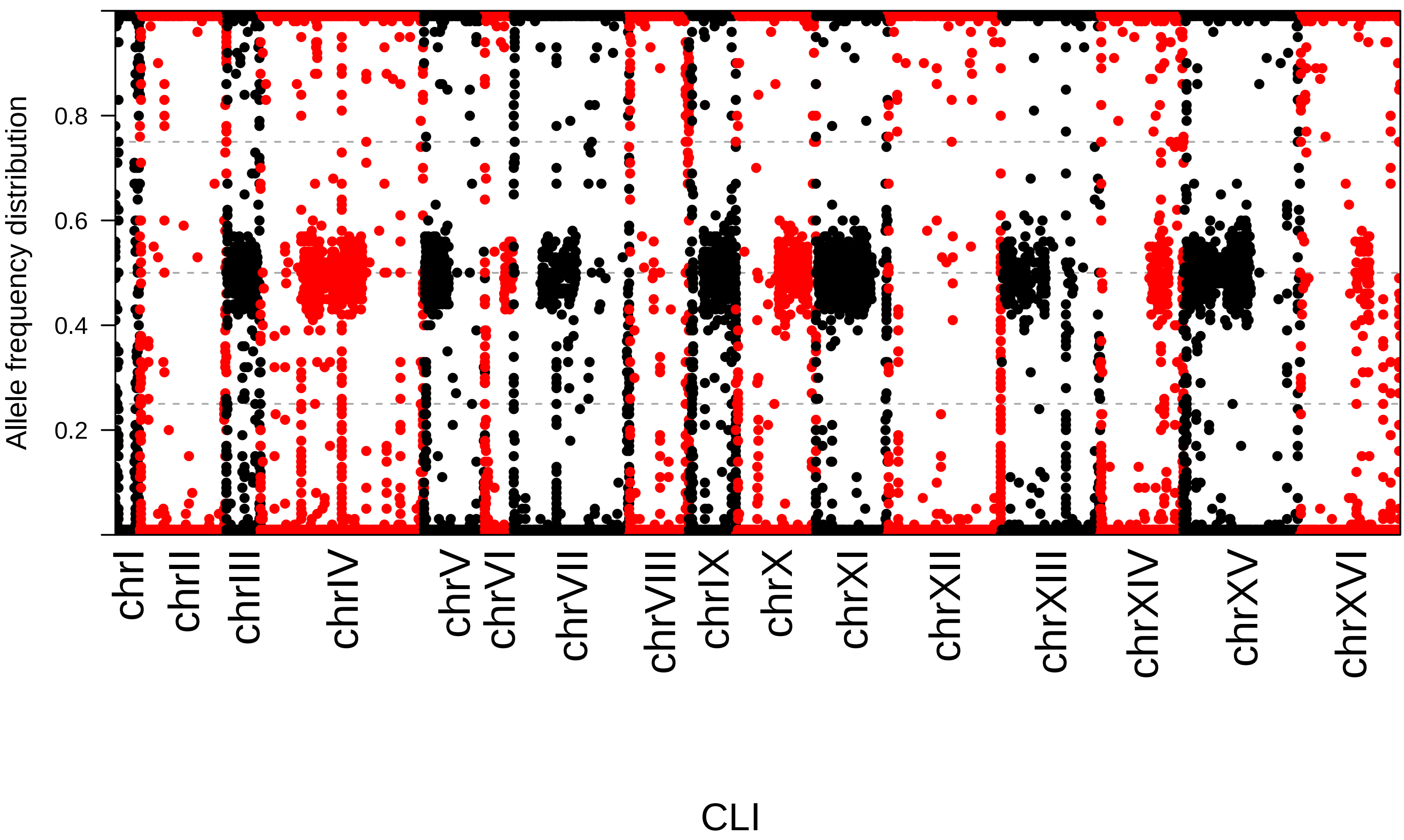

Supplementary Figure S3

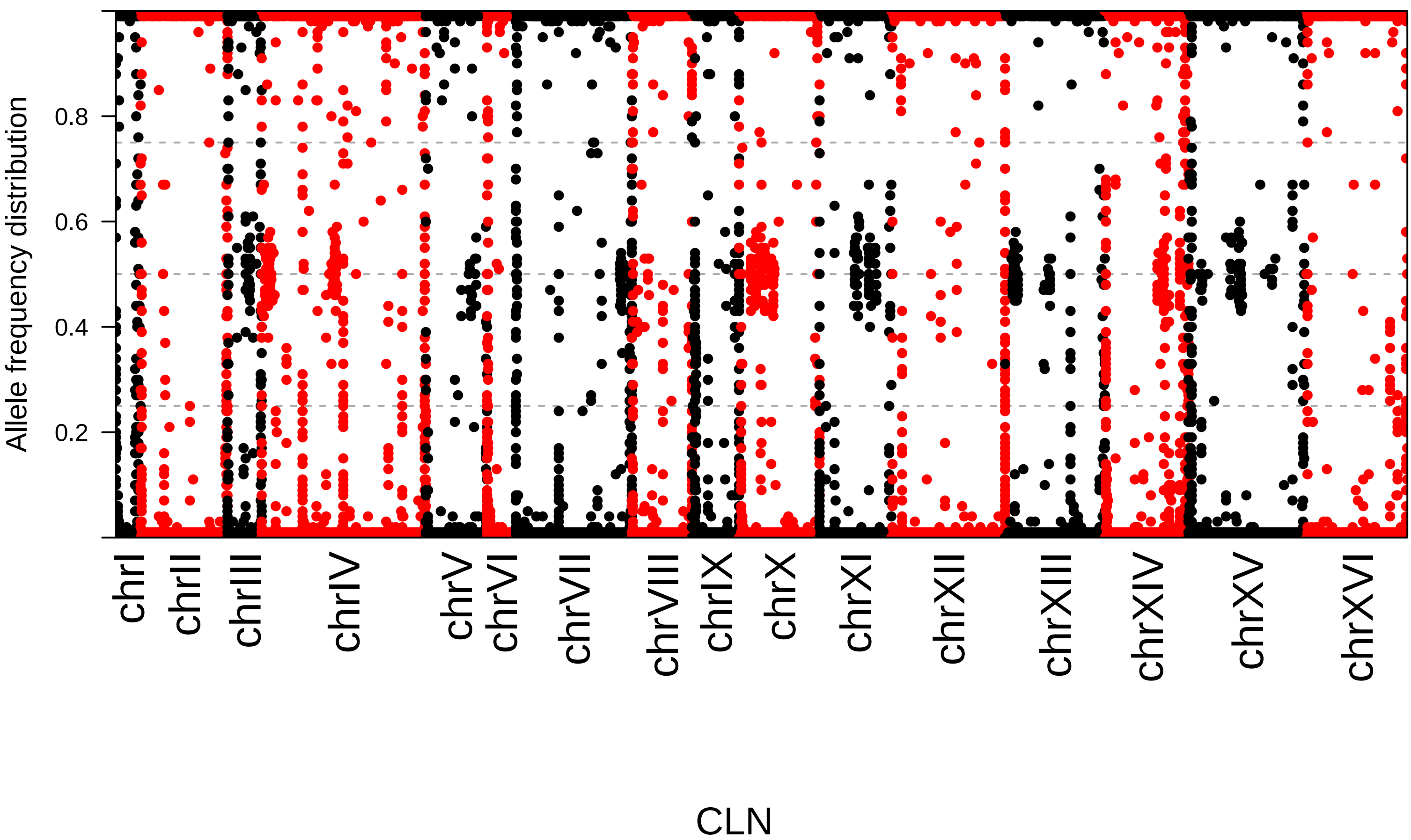

Supplementary Figure S3

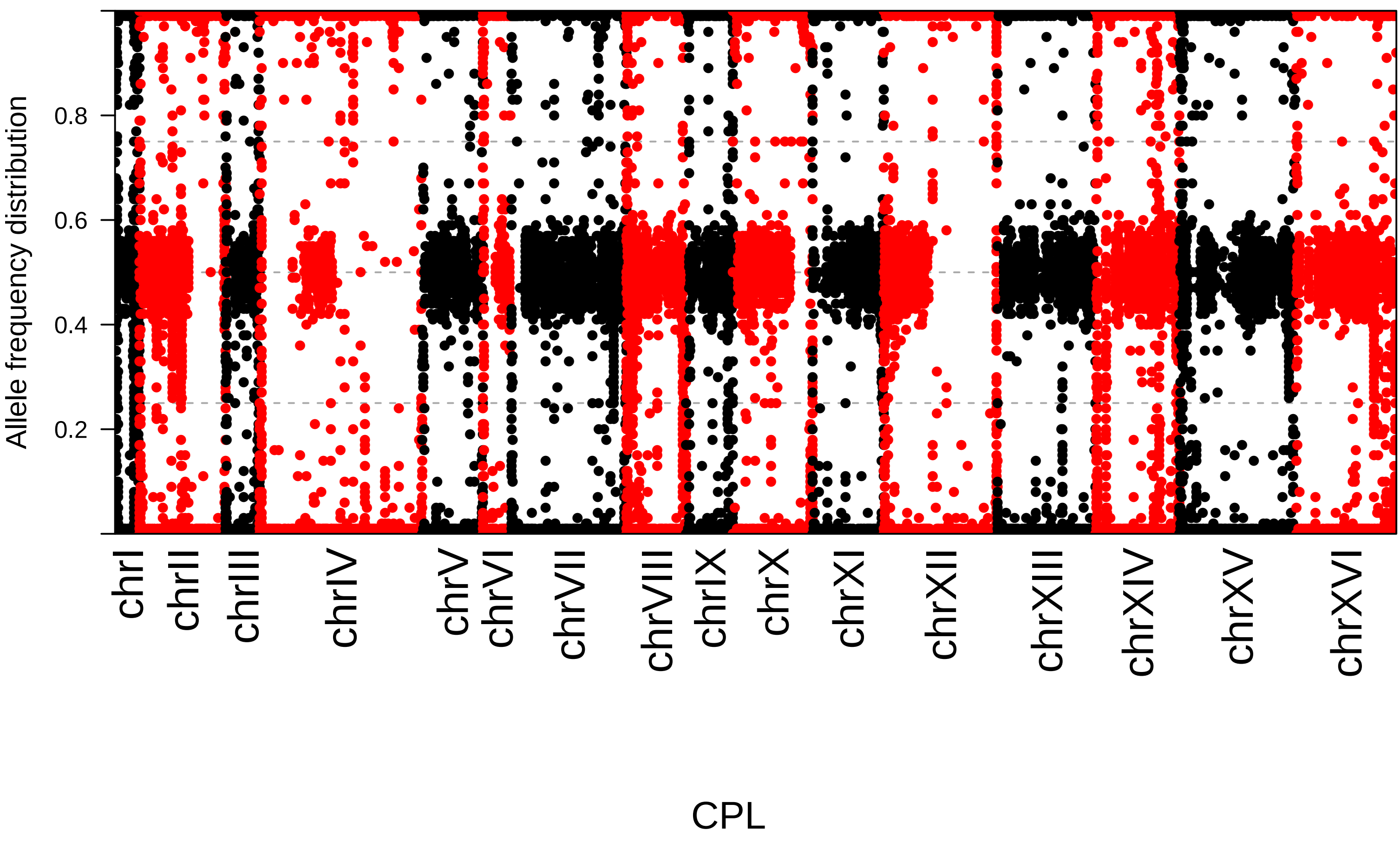

Supplementary Figure S3

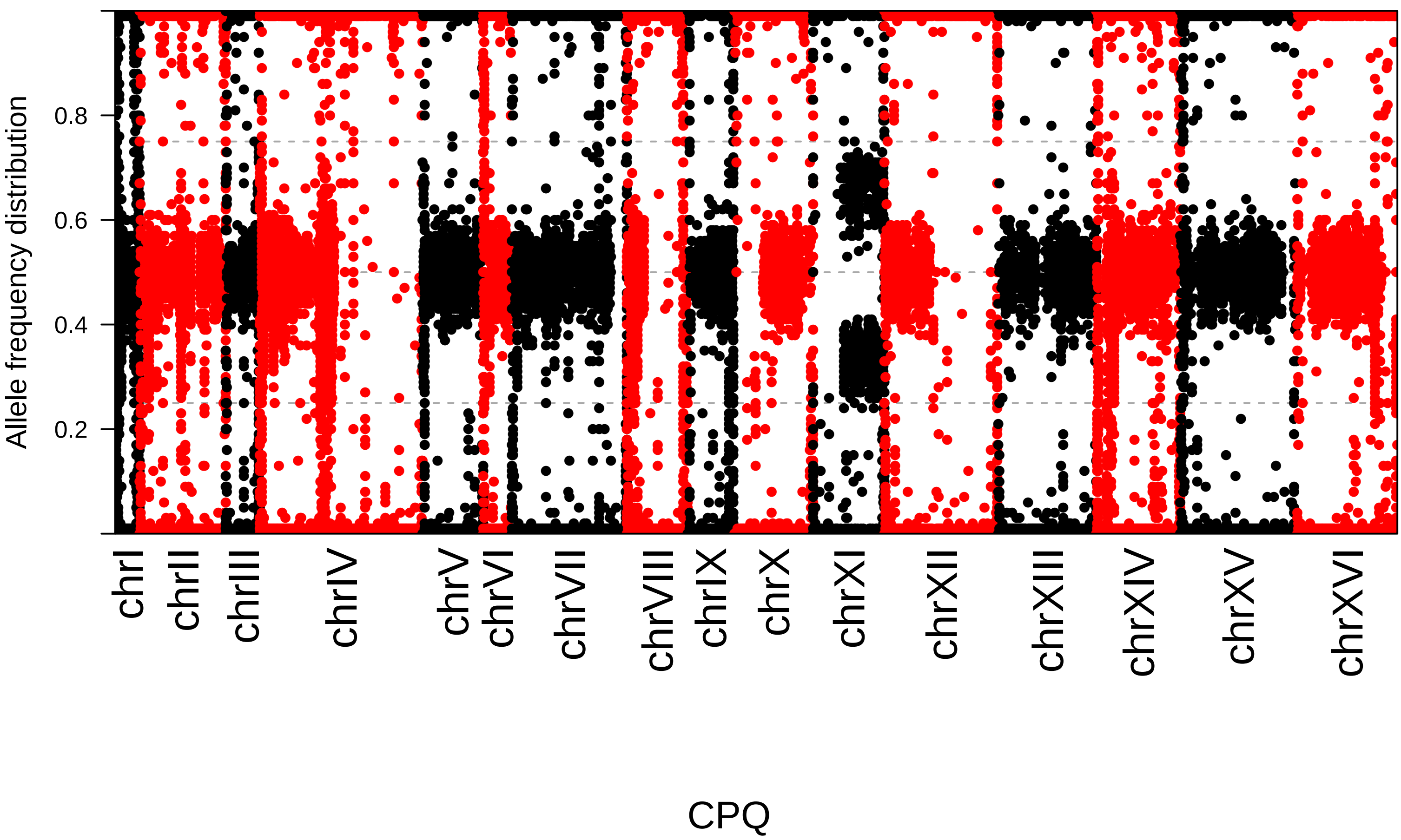

Supplementary Figure S3

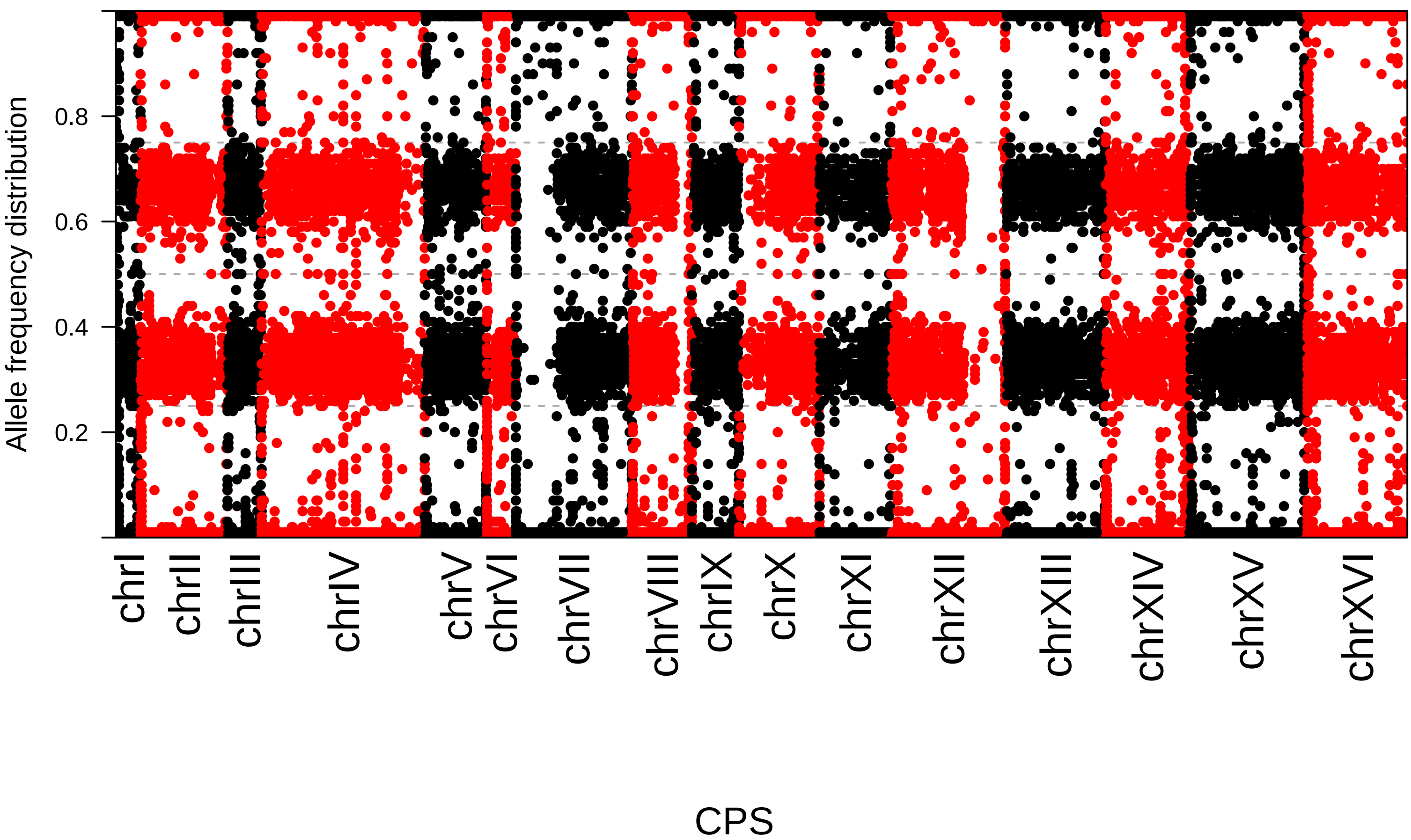

Supplementary Figure S3

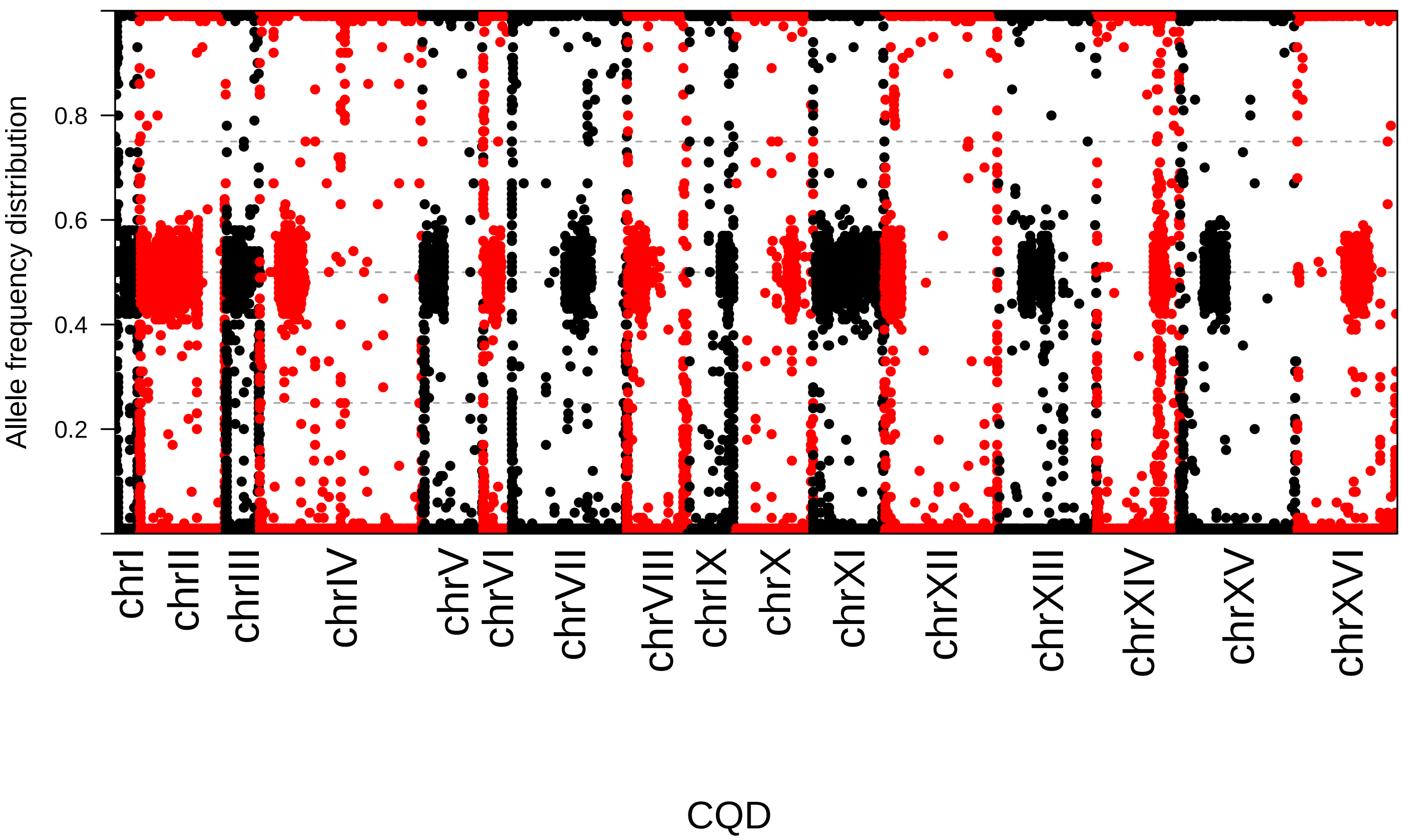

Supplementary Figure S3

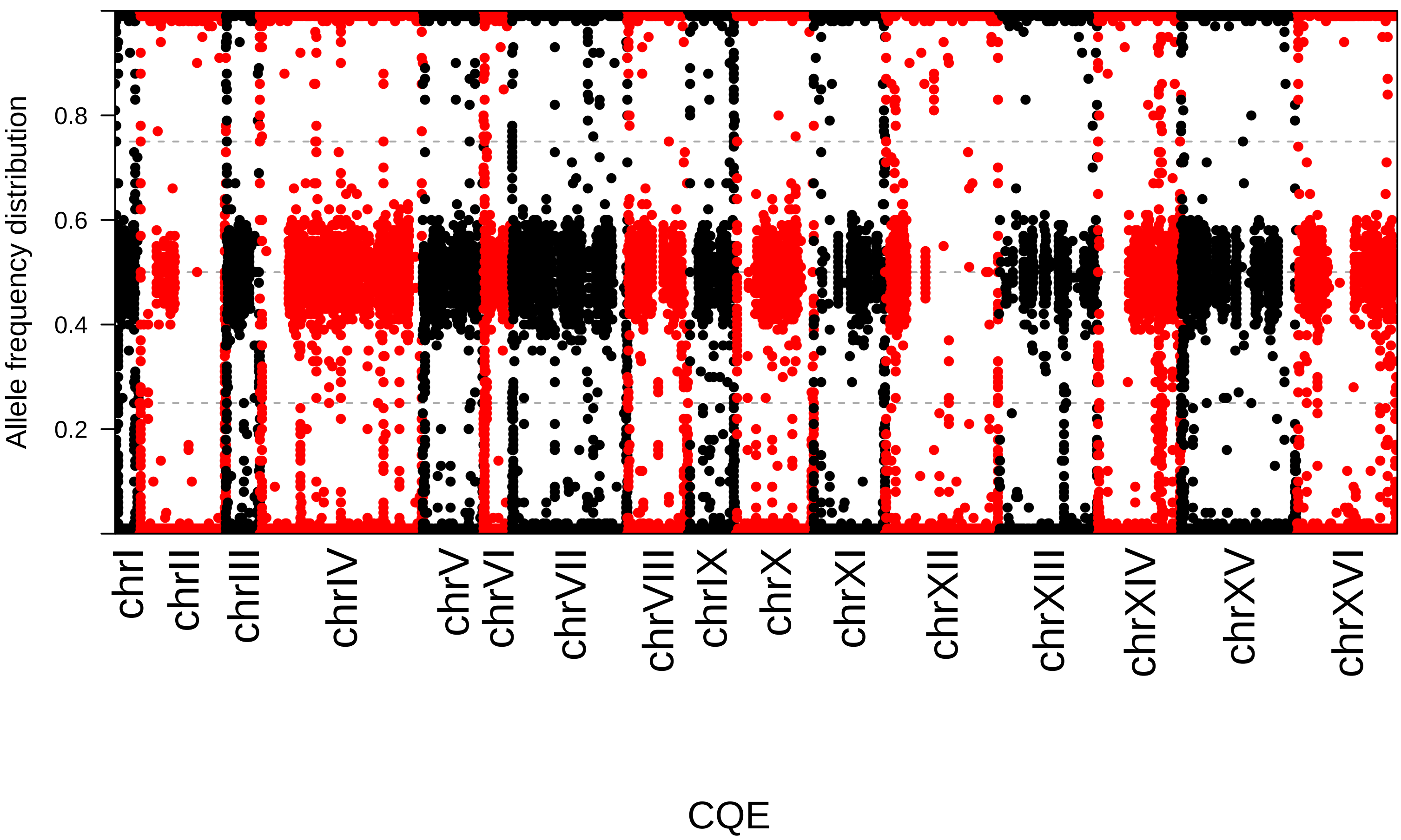

Supplementary Figure S3

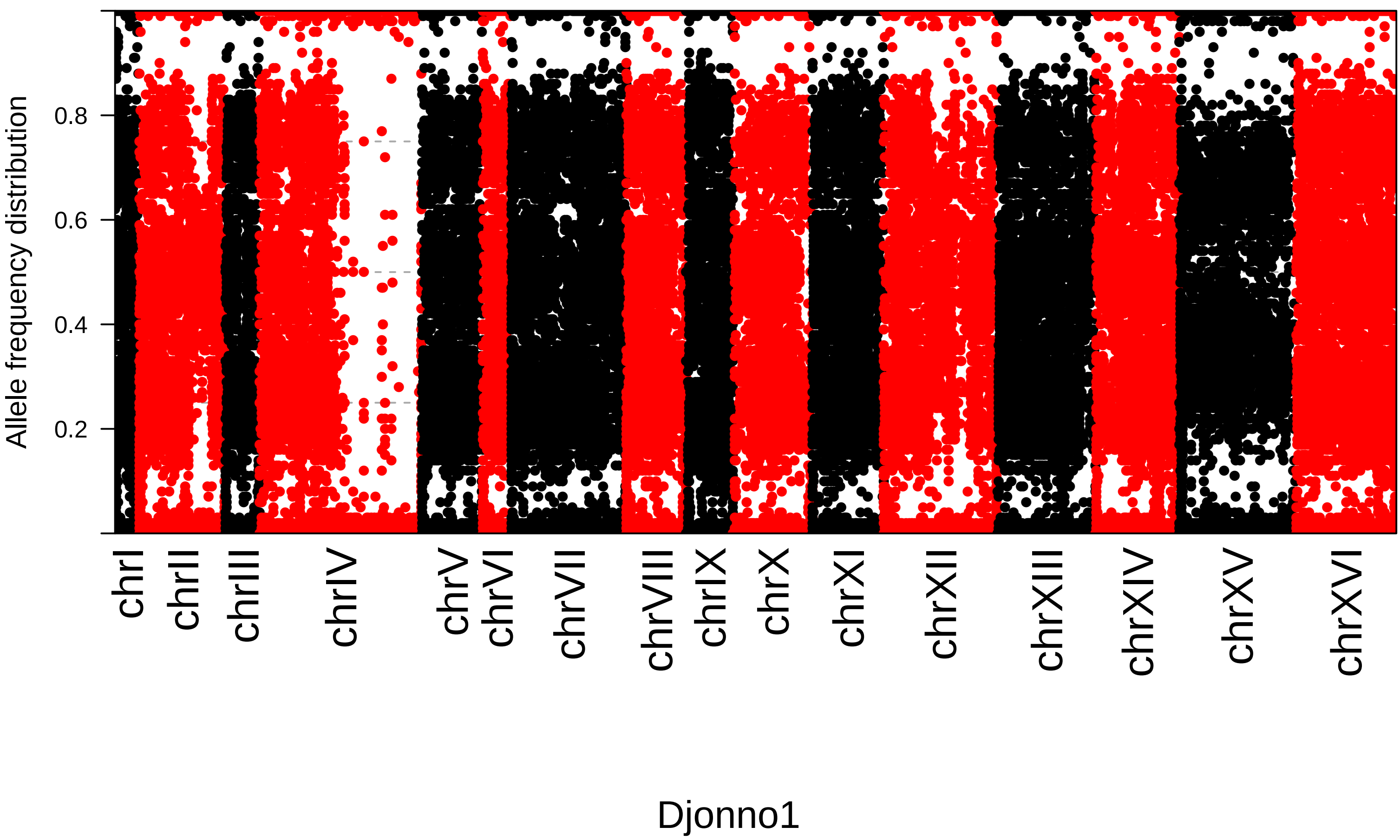

Supplementary Figure S3

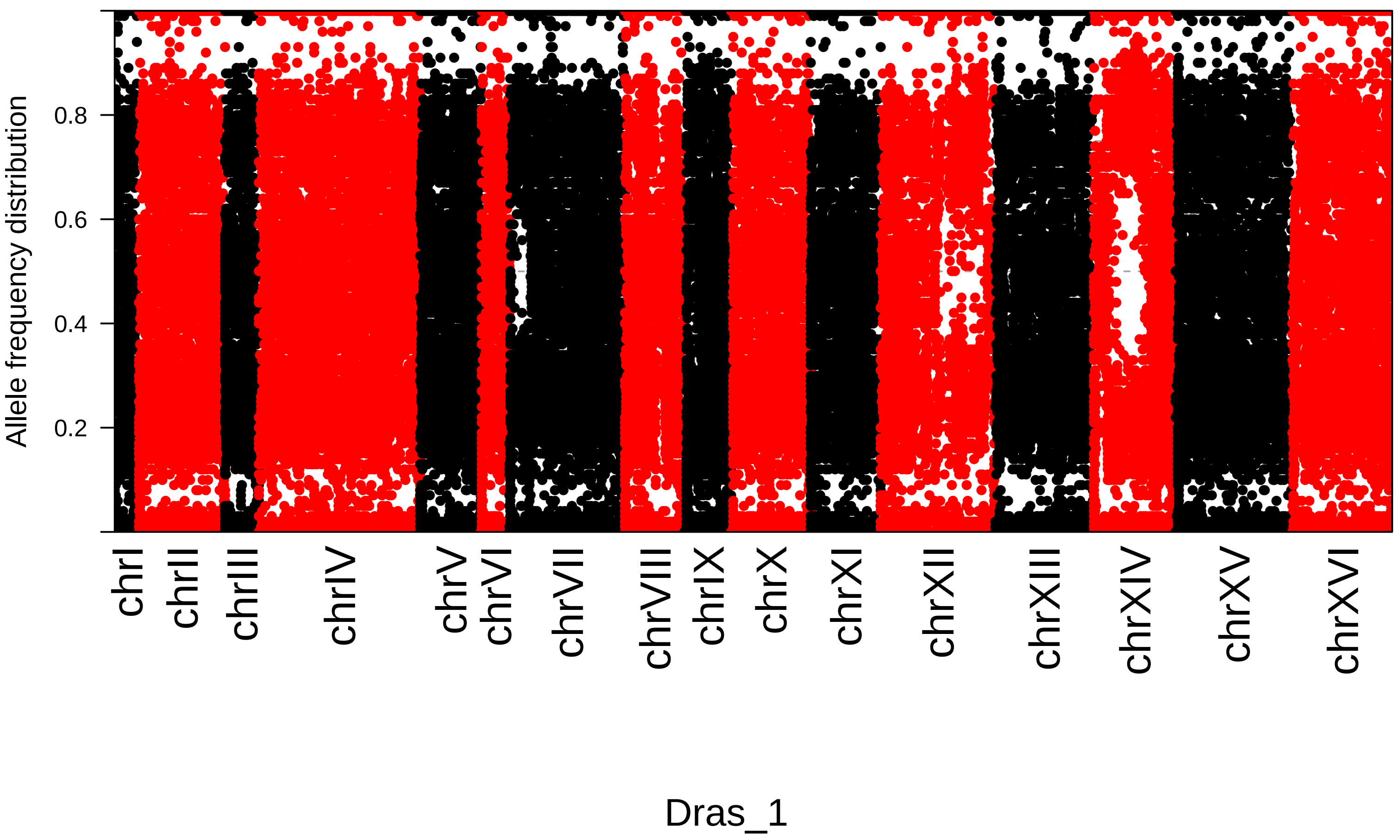

Supplementary Figure S3

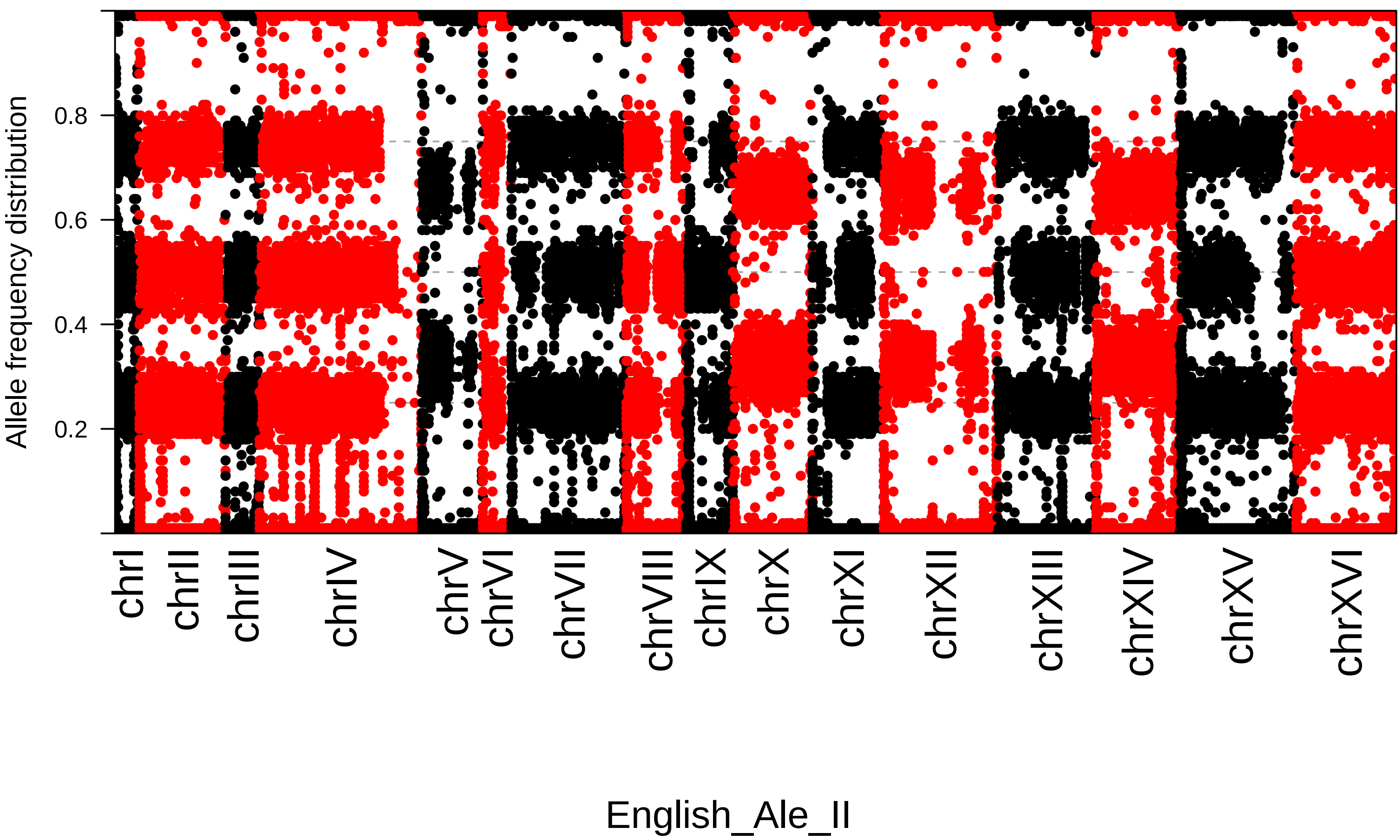

Supplementary Figure S3

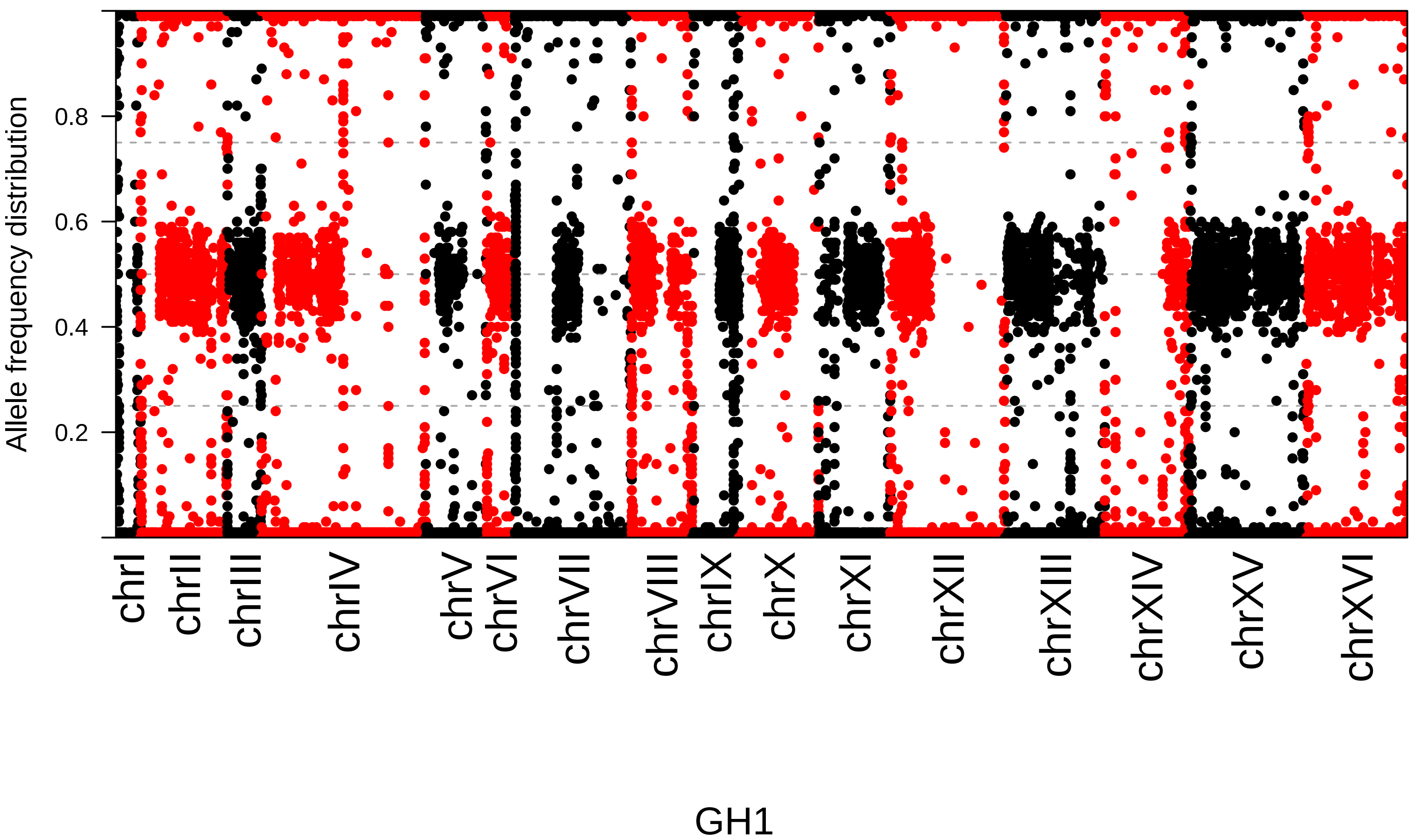

Supplementary Figure S3

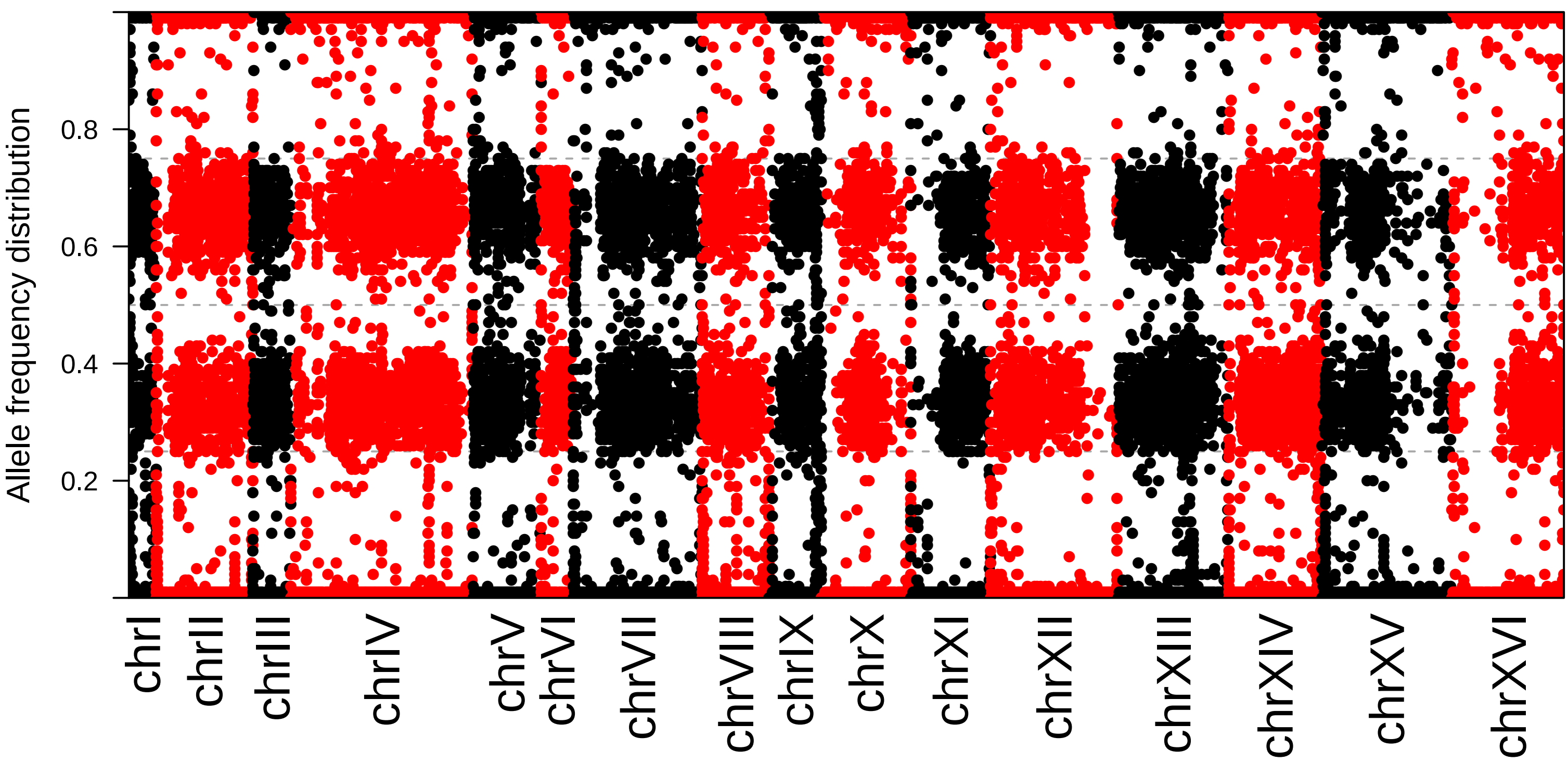

GH2

Supplementary Figure S3

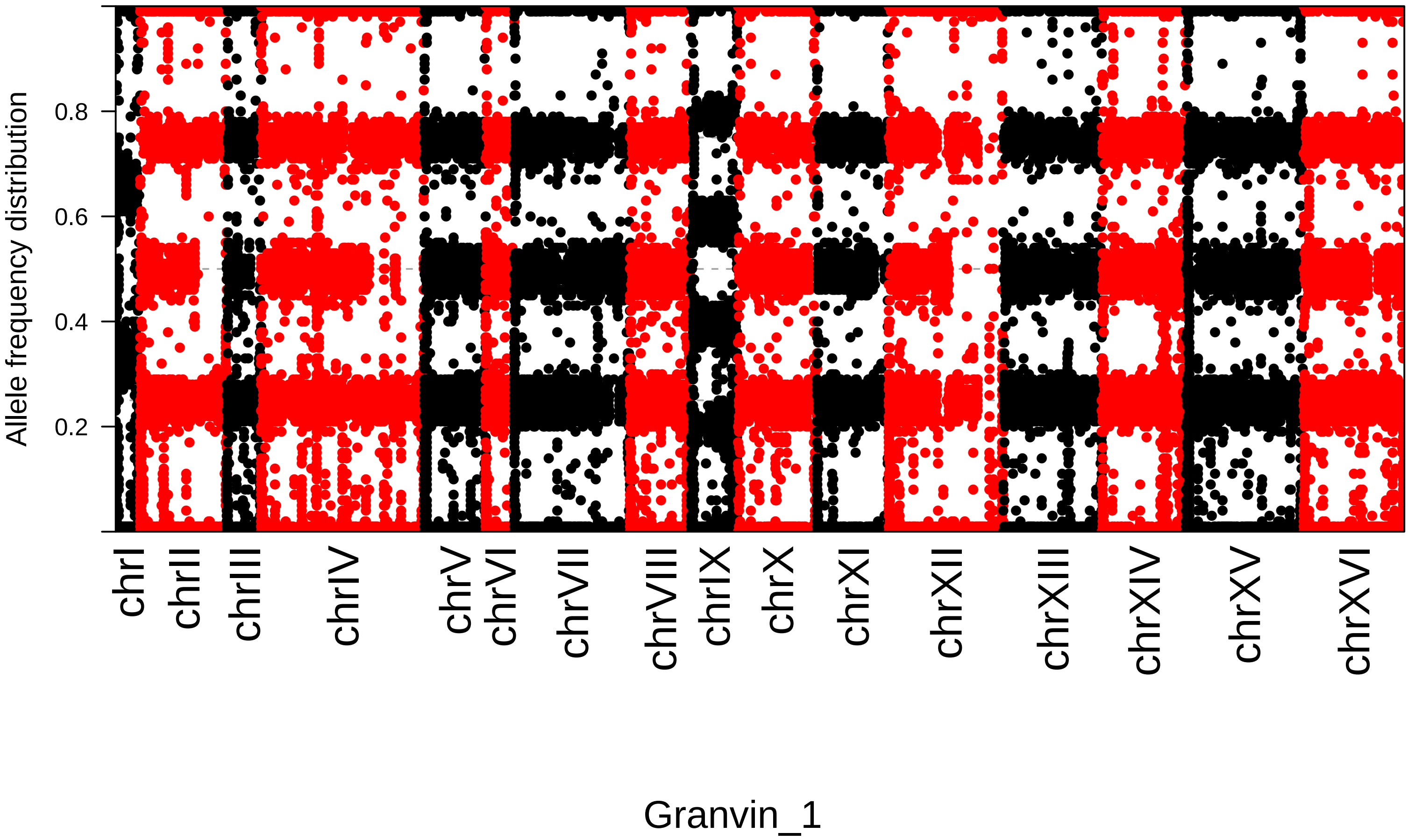

Supplementary Figure S3

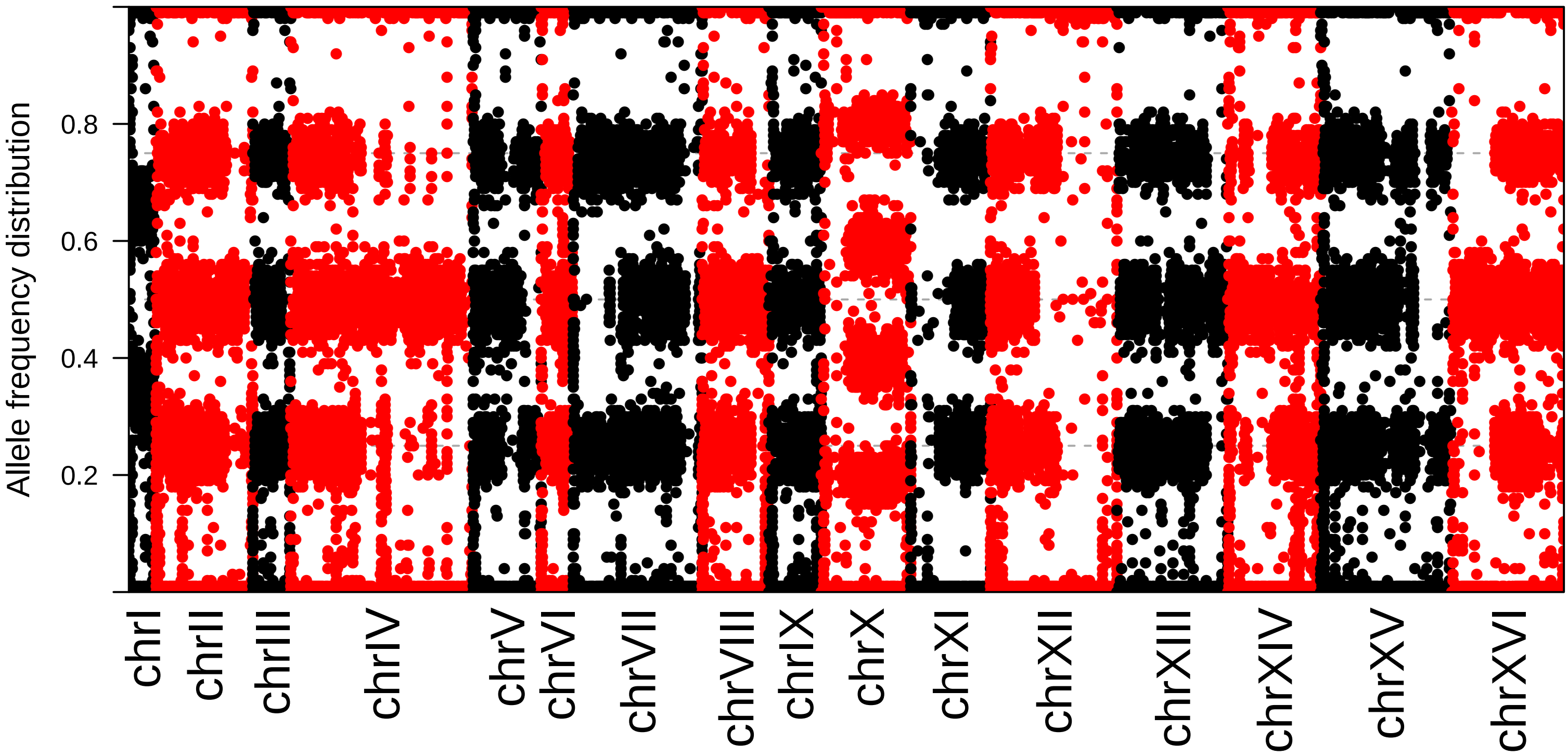

Halvorsgard_3

Supplementary Figure S3

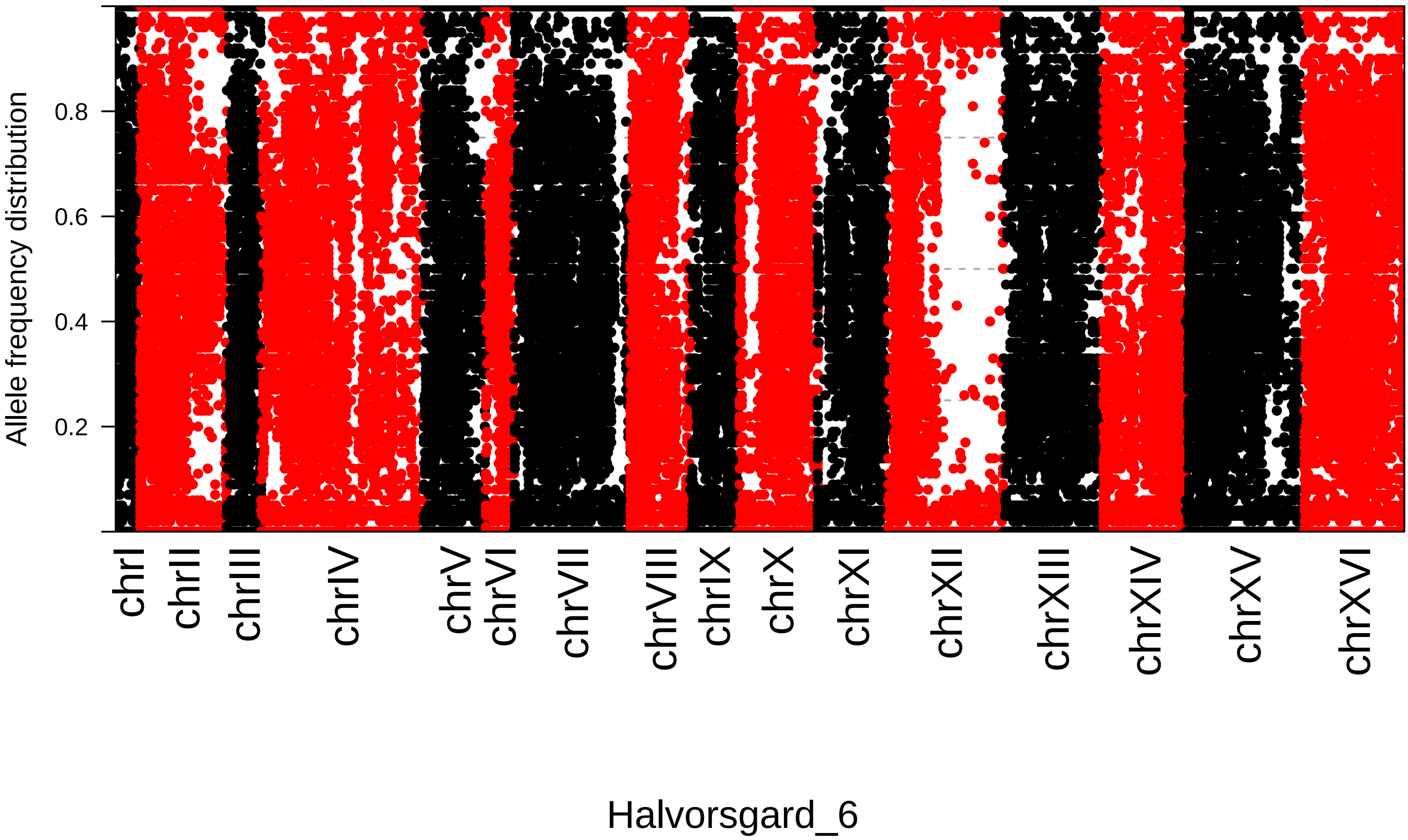

Supplementary Figure S3

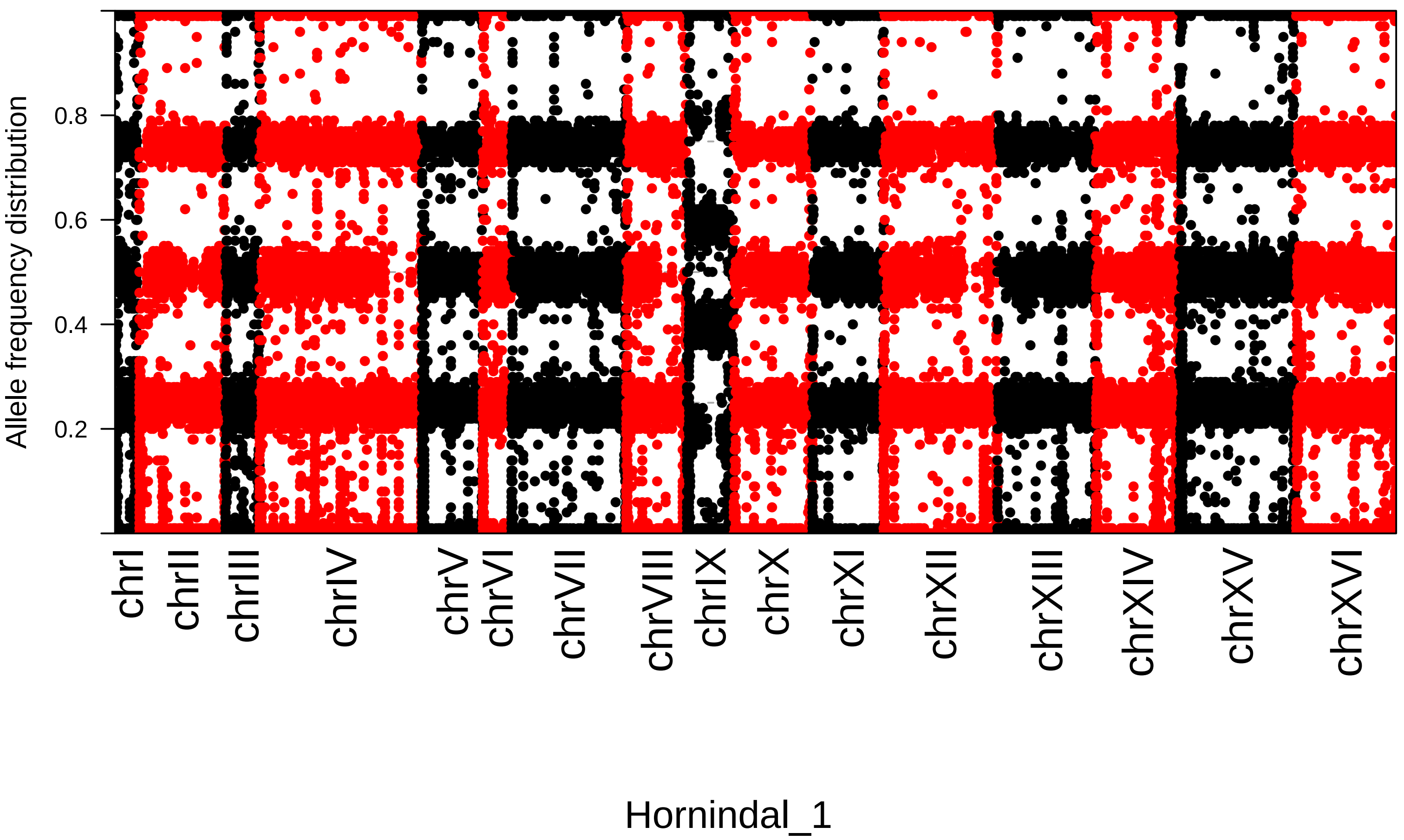

Supplementary Figure S3

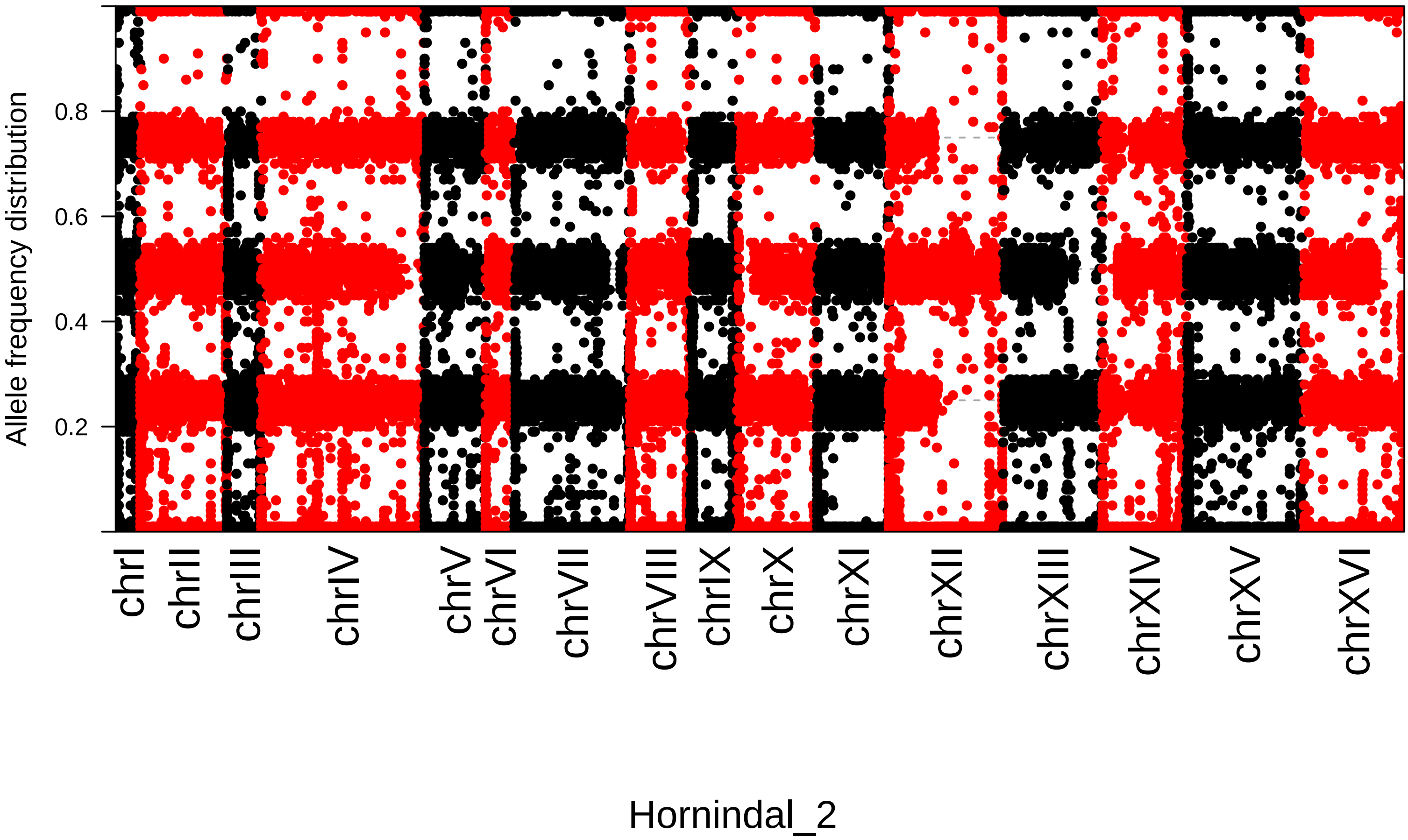

Supplementary Figure S3

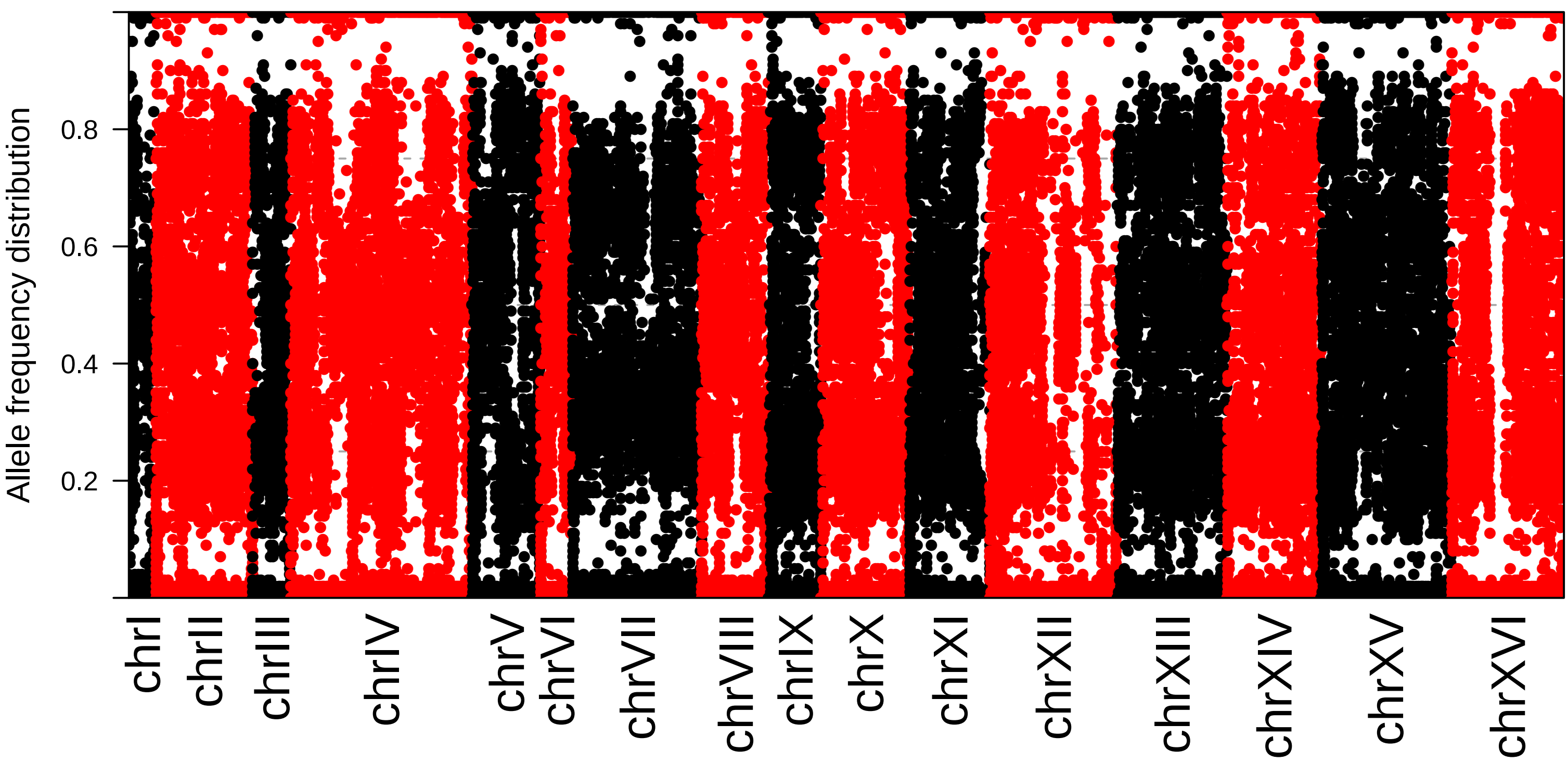

Ishlei1

Supplementary Figure S3

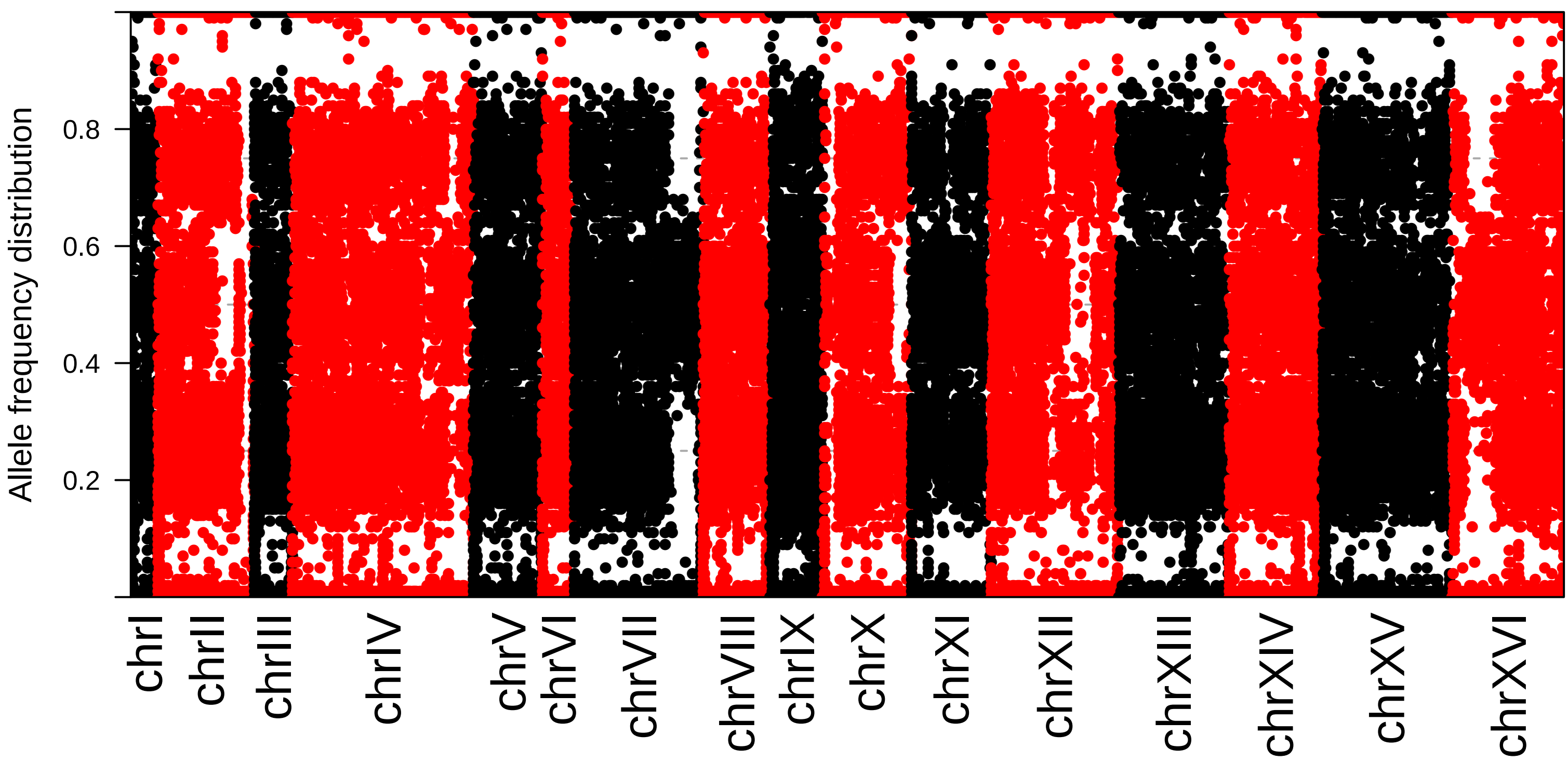

Ivar_Geithung_1

Supplementary Figure S3

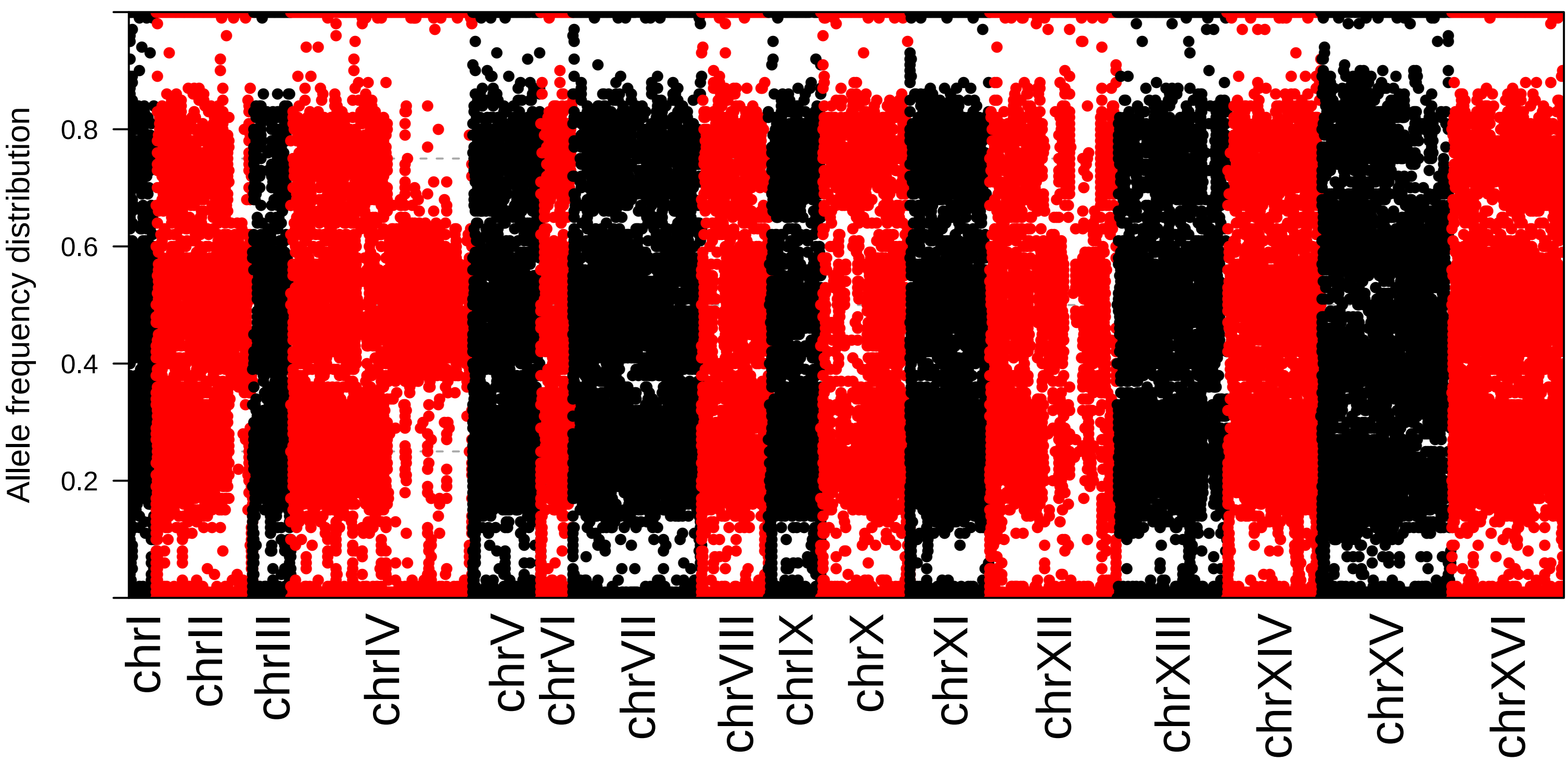

Ivar_Geithung_2

Supplementary Figure S3

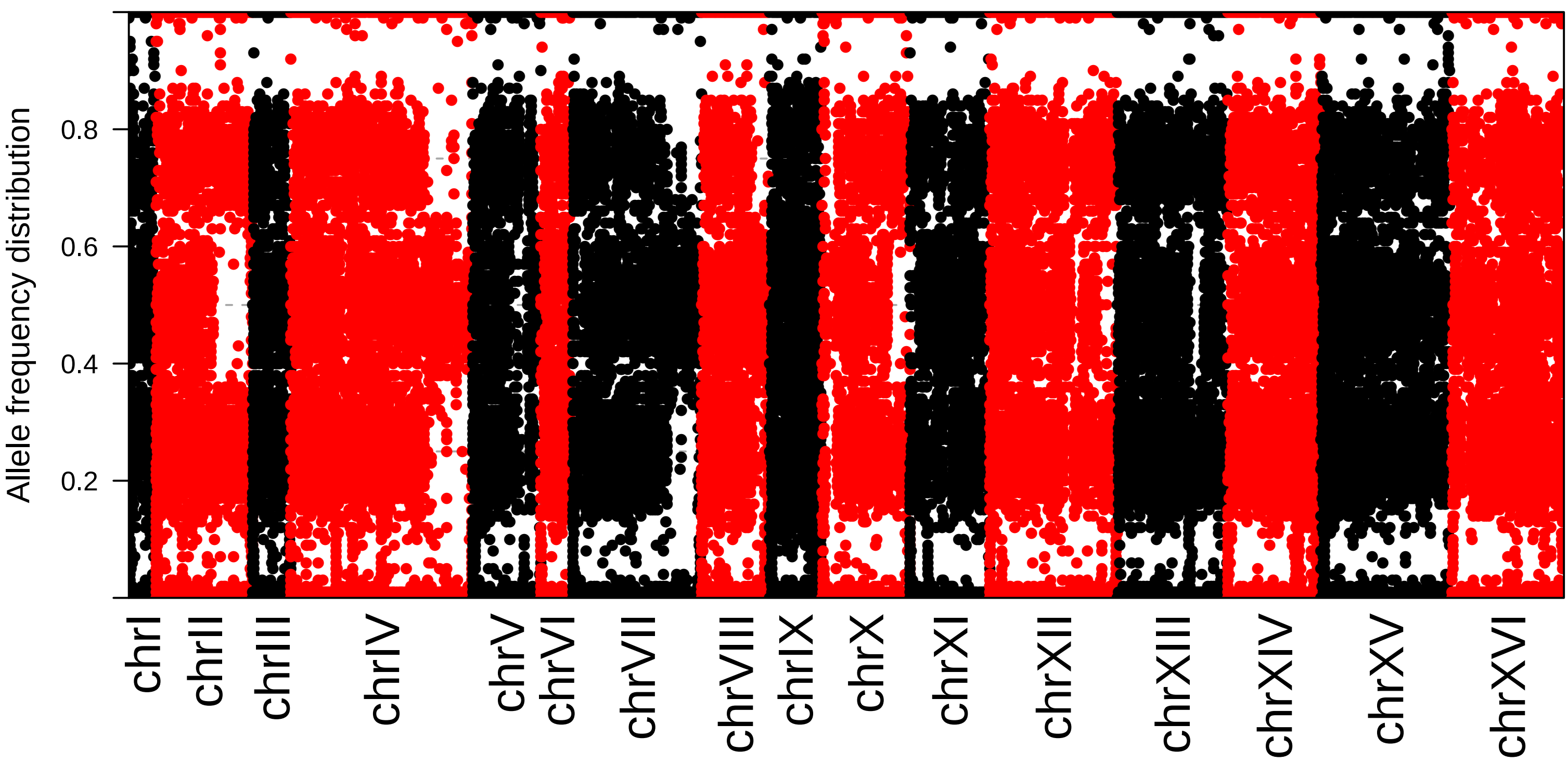

Ivar_Geithung_3

Supplementary Figure S3

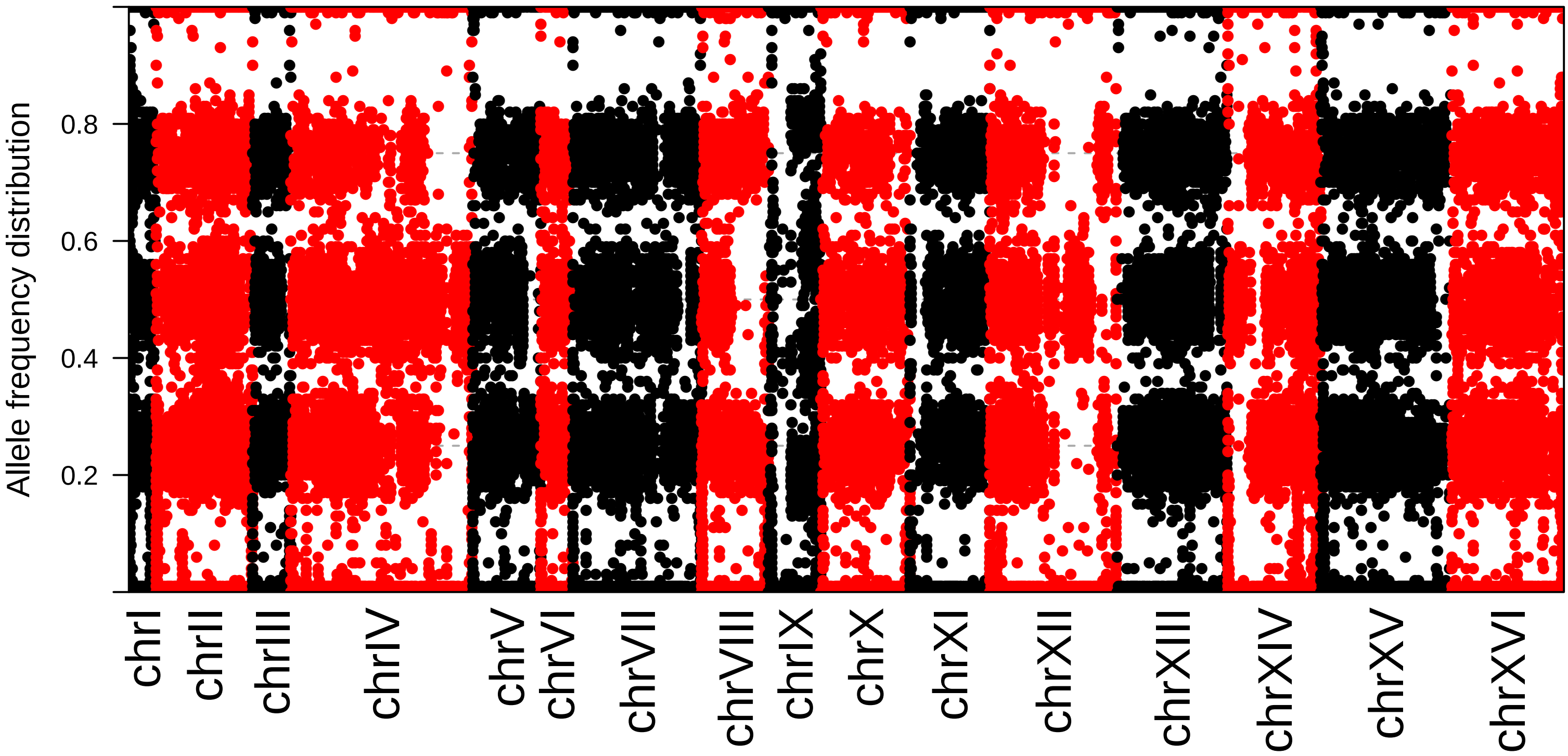

Jordal1

Supplementary Figure S3

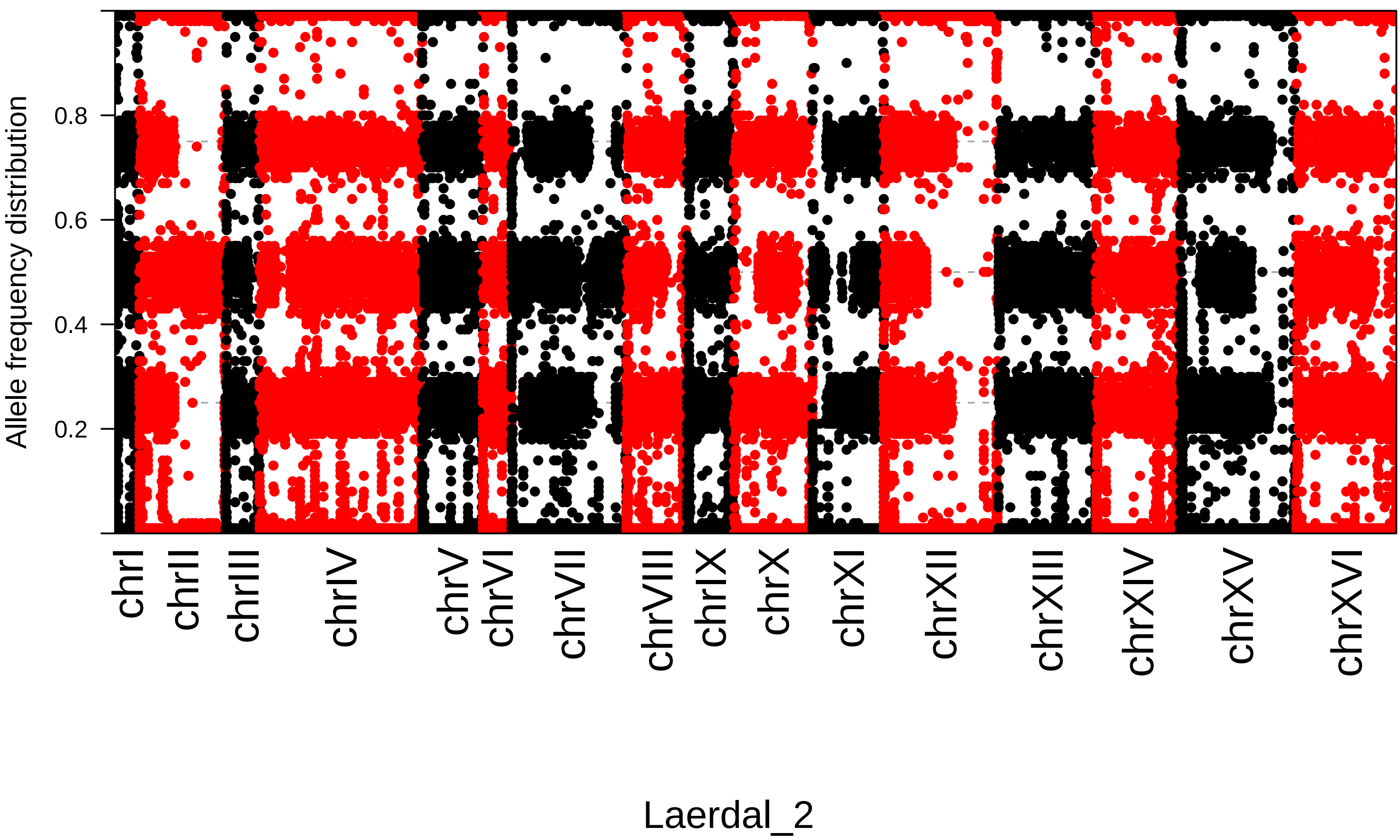

Supplementary Figure S3

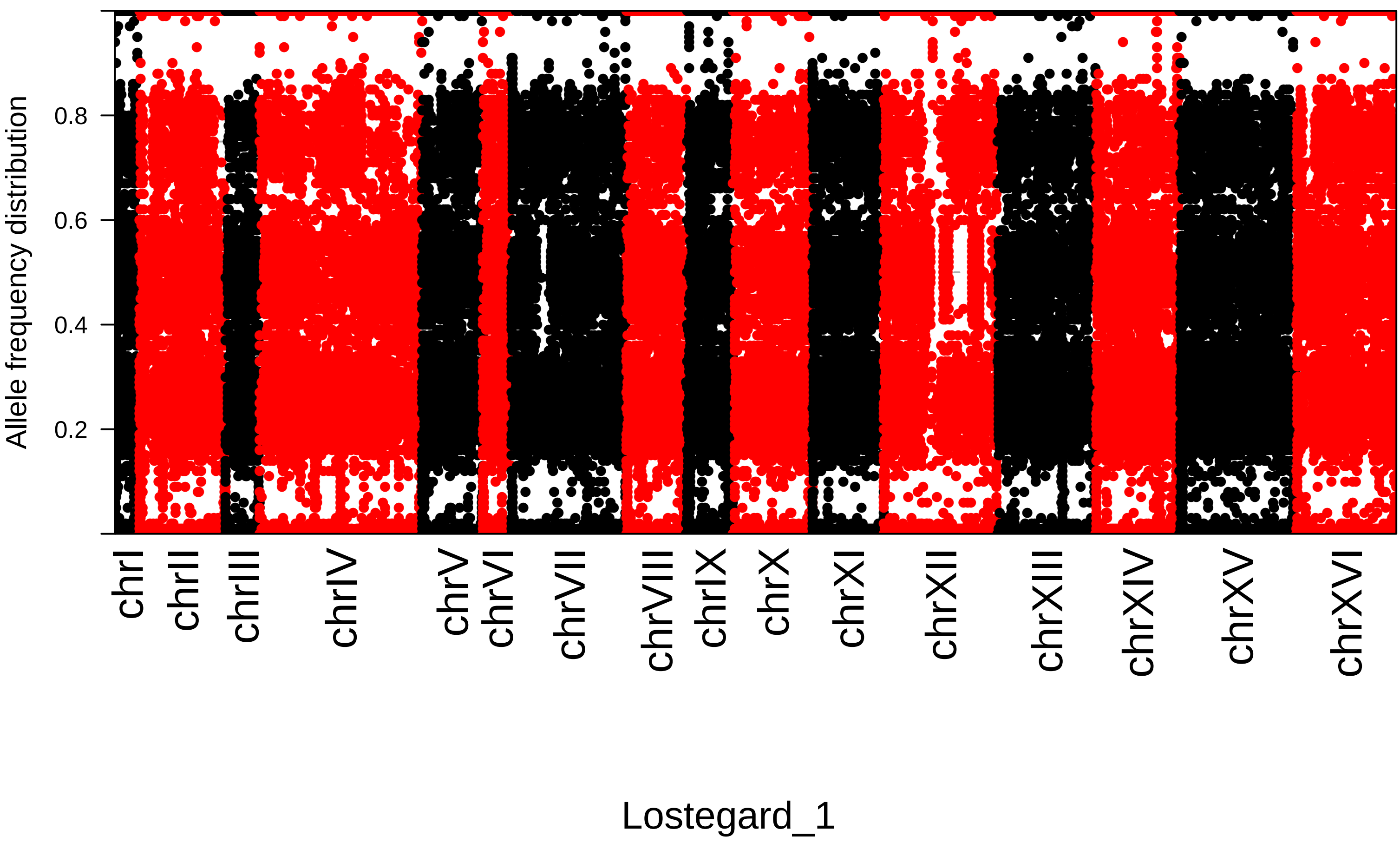

Supplementary Figure S3

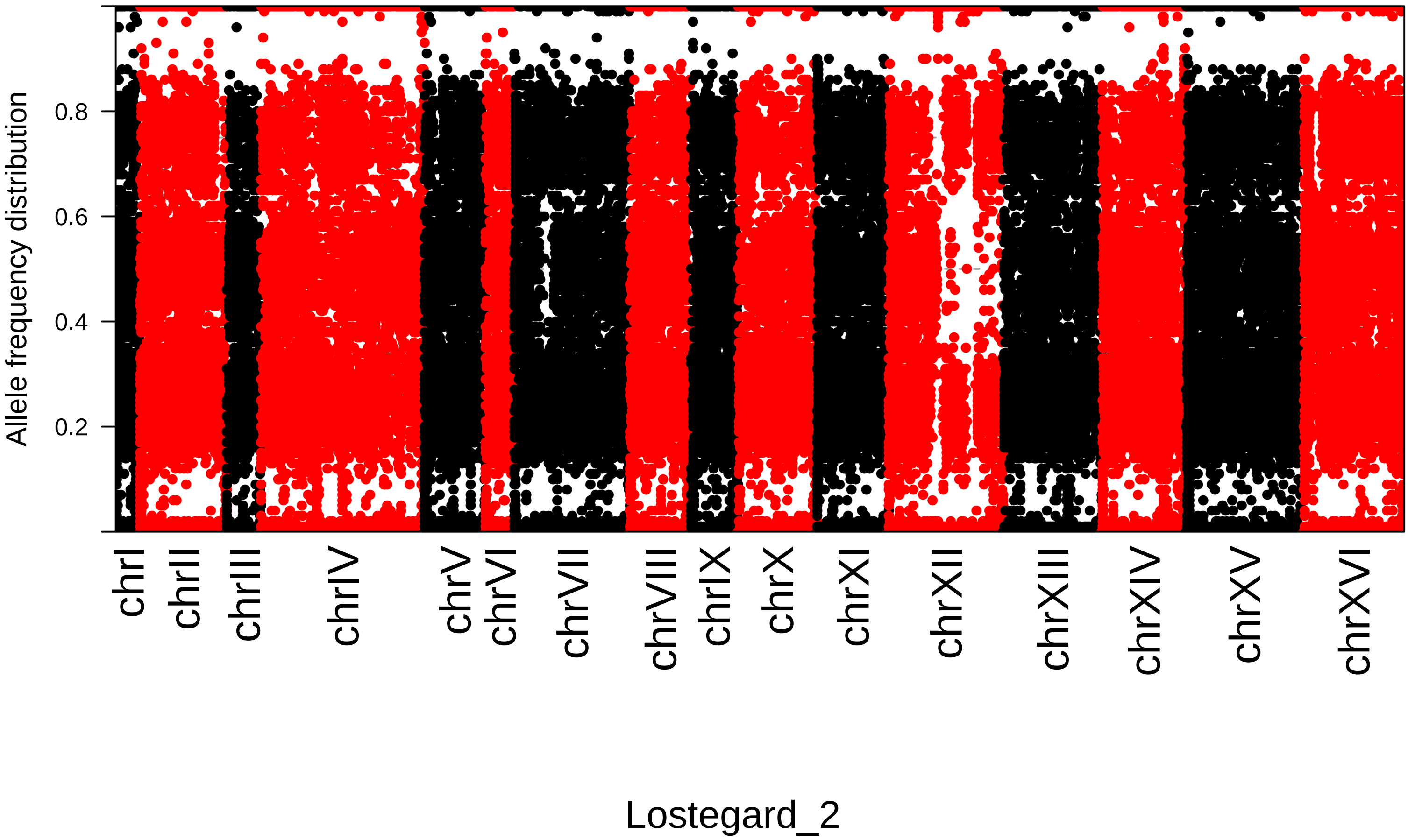

Supplementary Figure S3

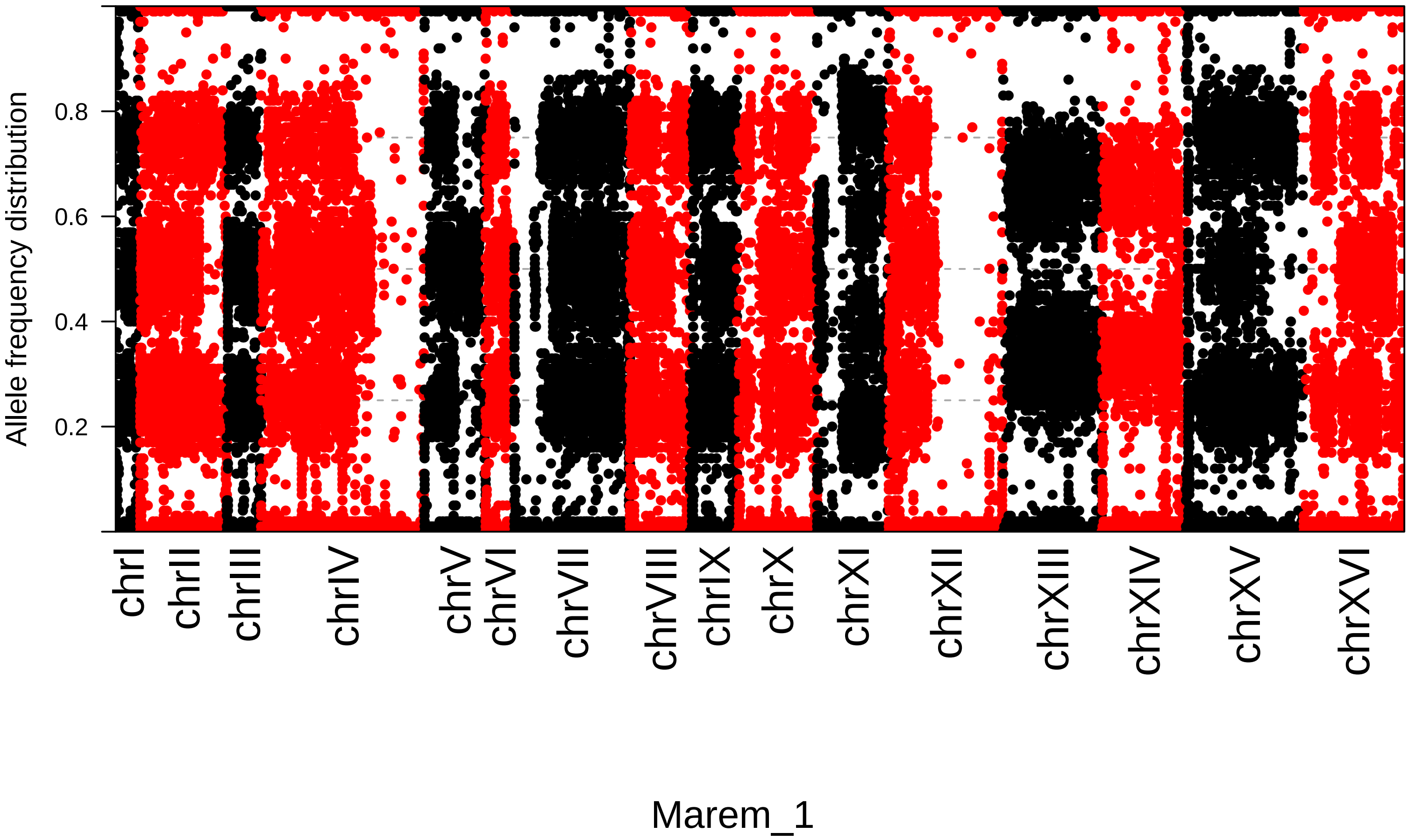

Supplementary Figure S3

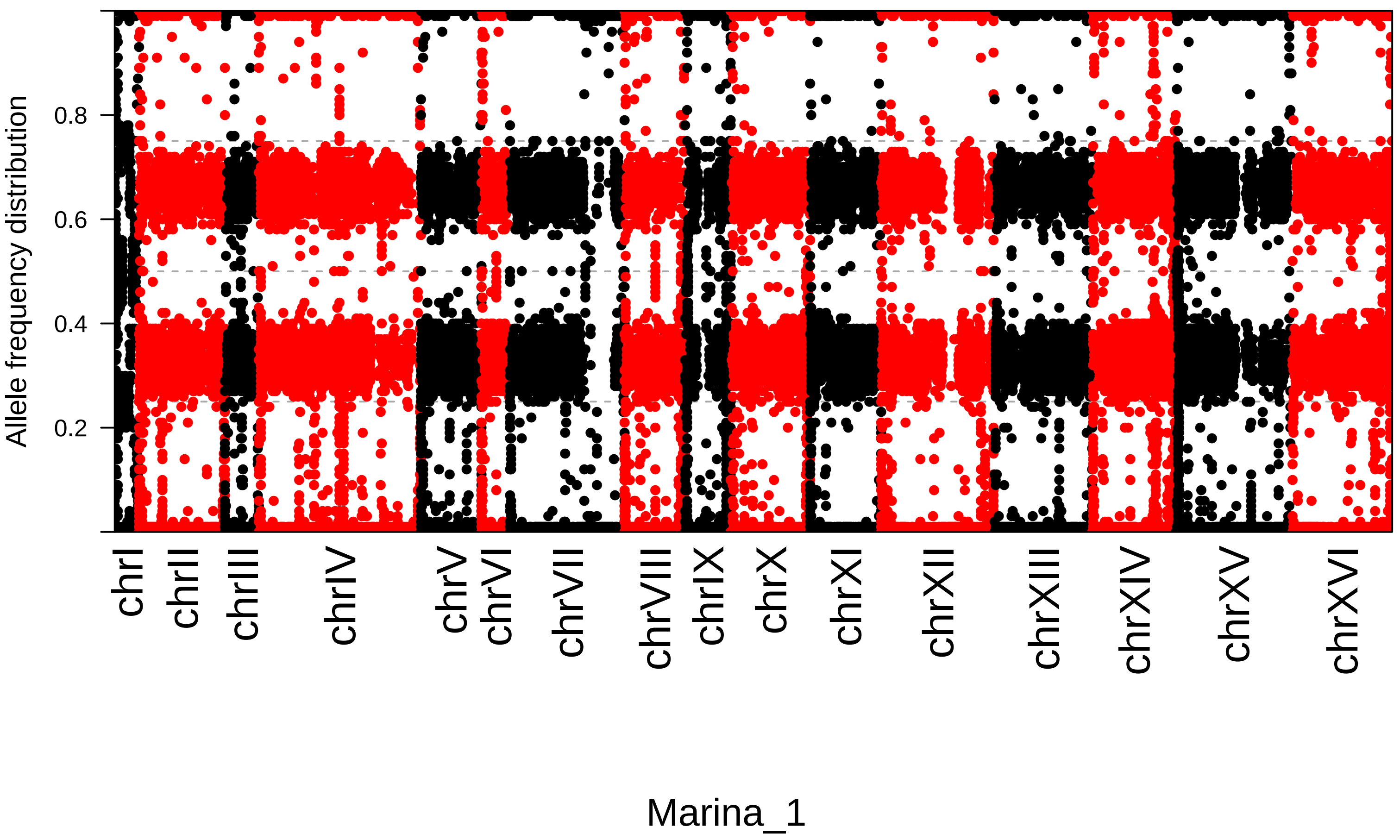

Supplementary Figure S3

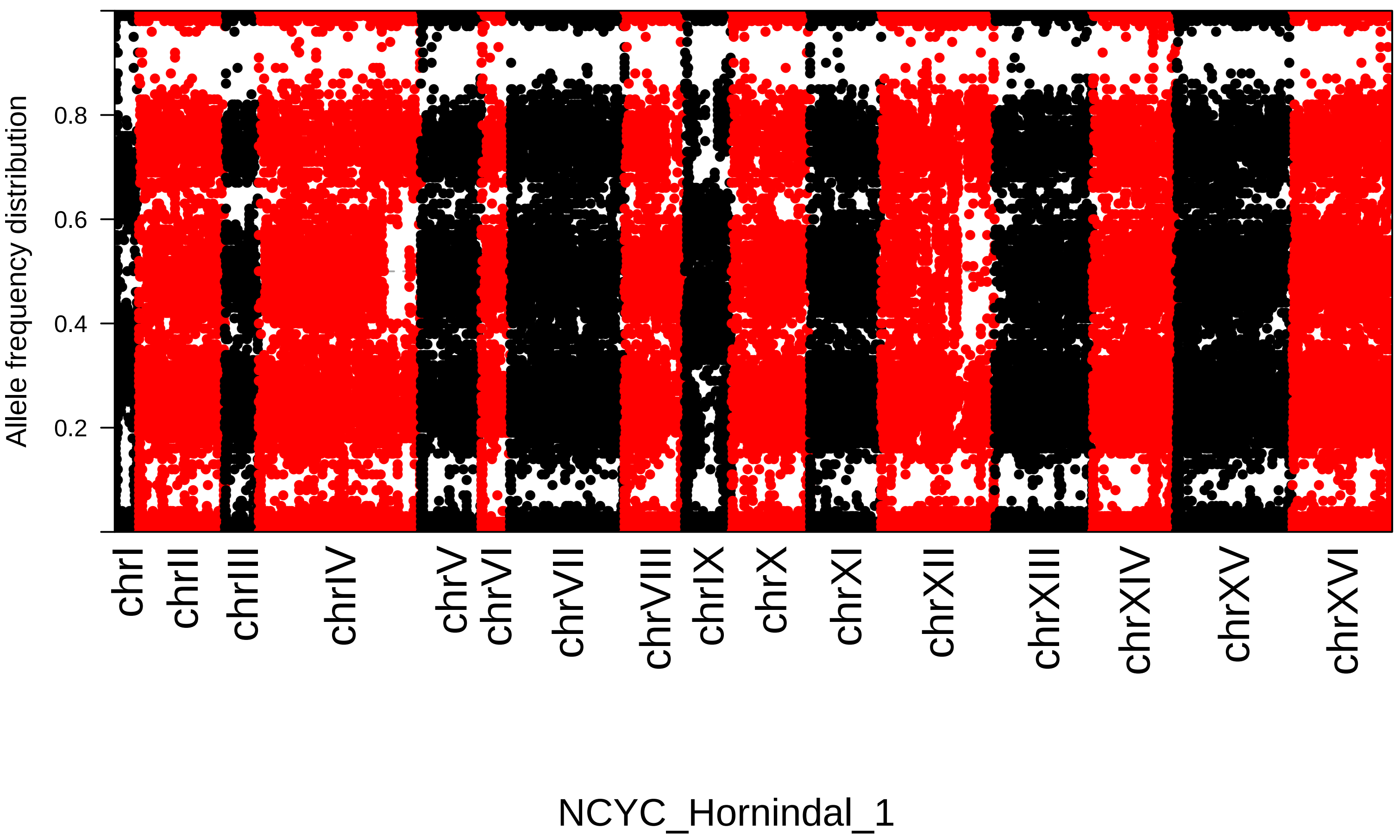

Supplementary Figure S3

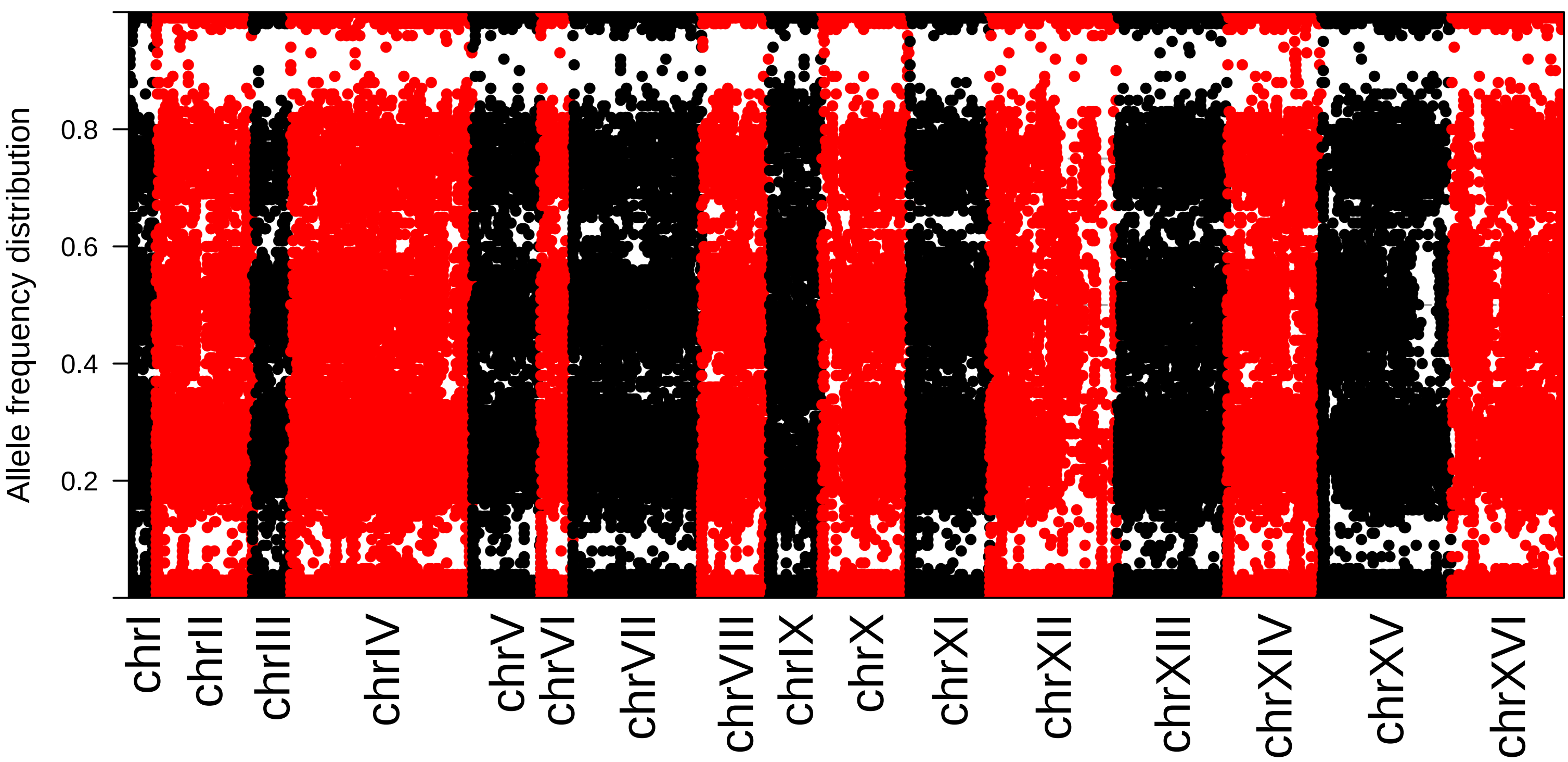

NCYC_Hornindal_2

Supplementary Figure S3

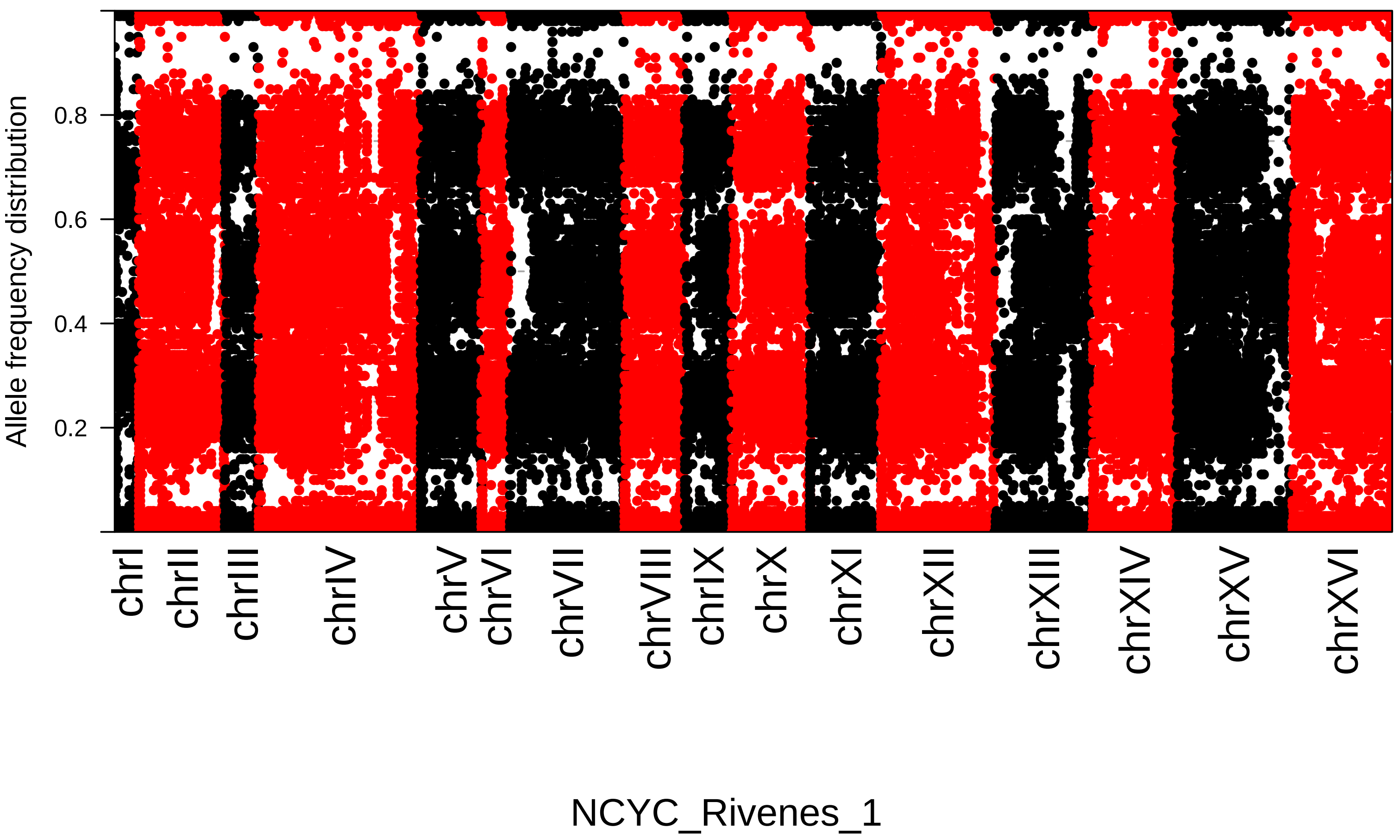

Supplementary Figure S3

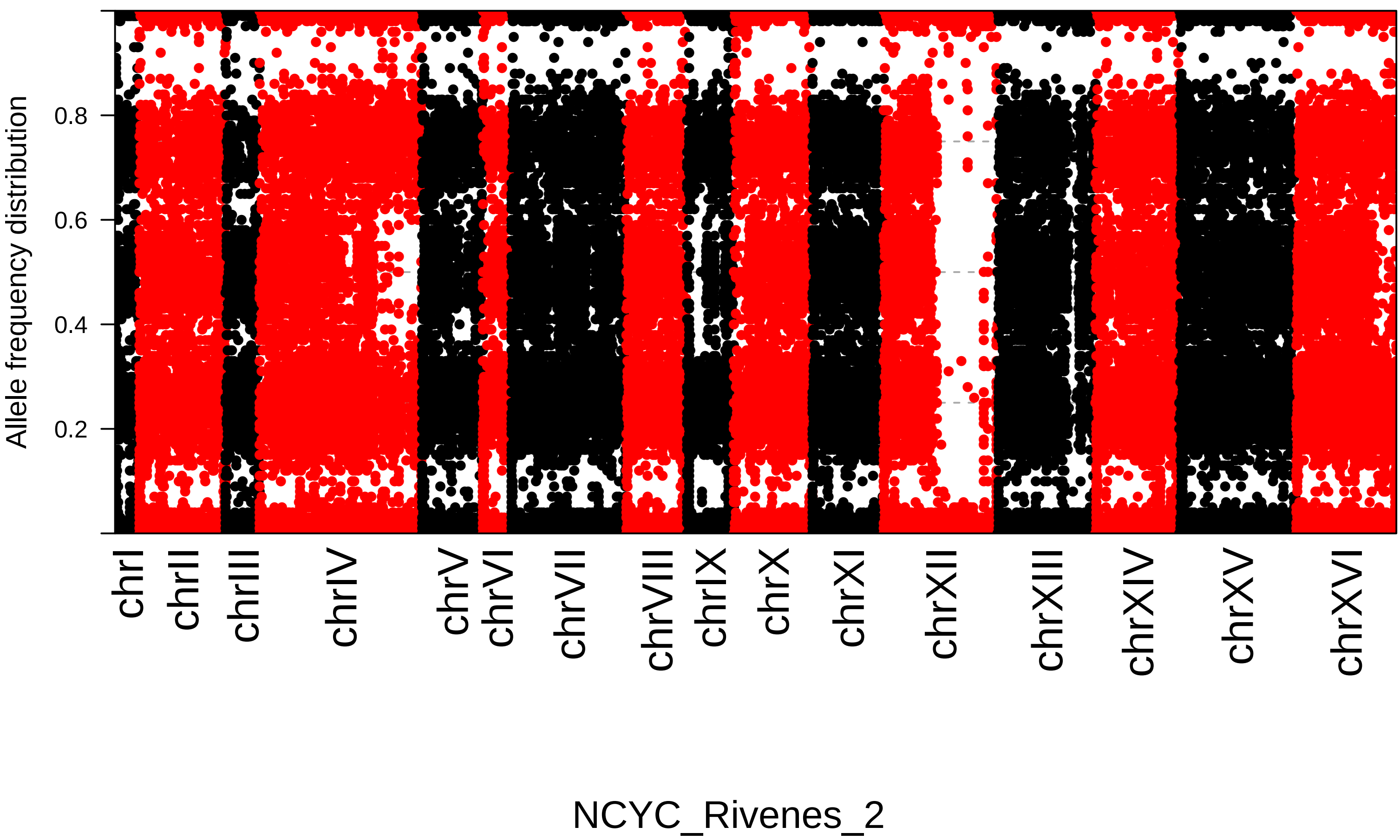

Supplementary Figure S3

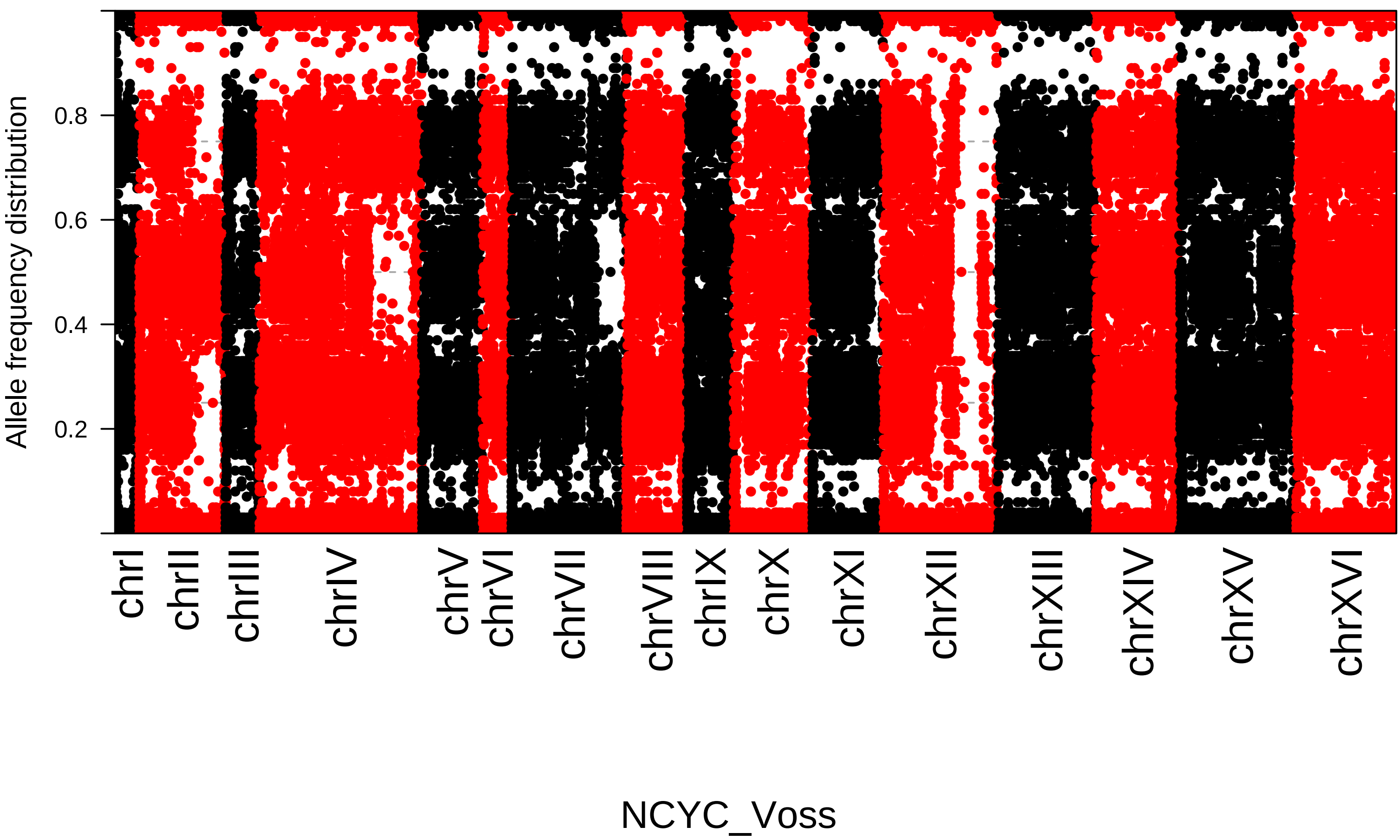

Supplementary Figure S3

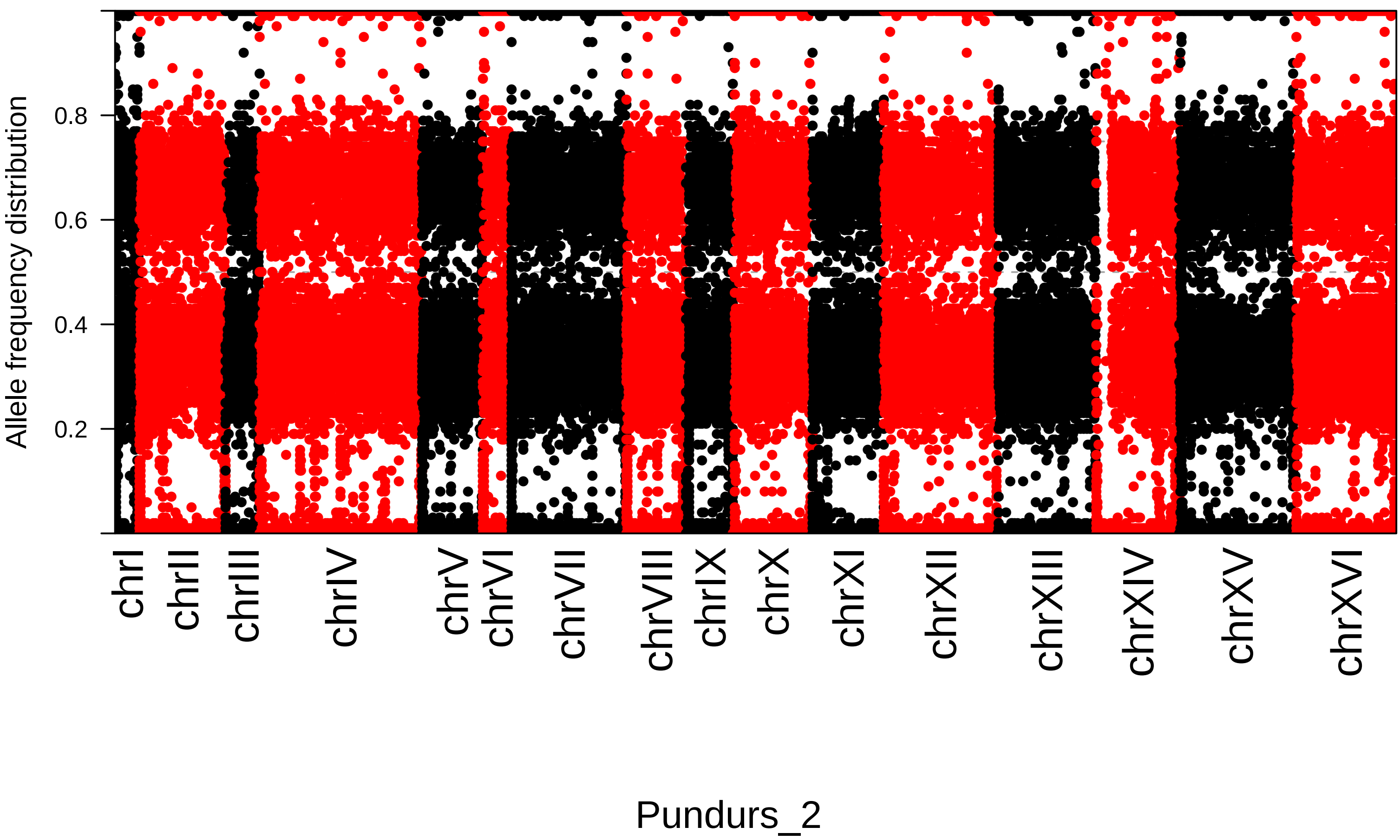

Supplementary Figure S3

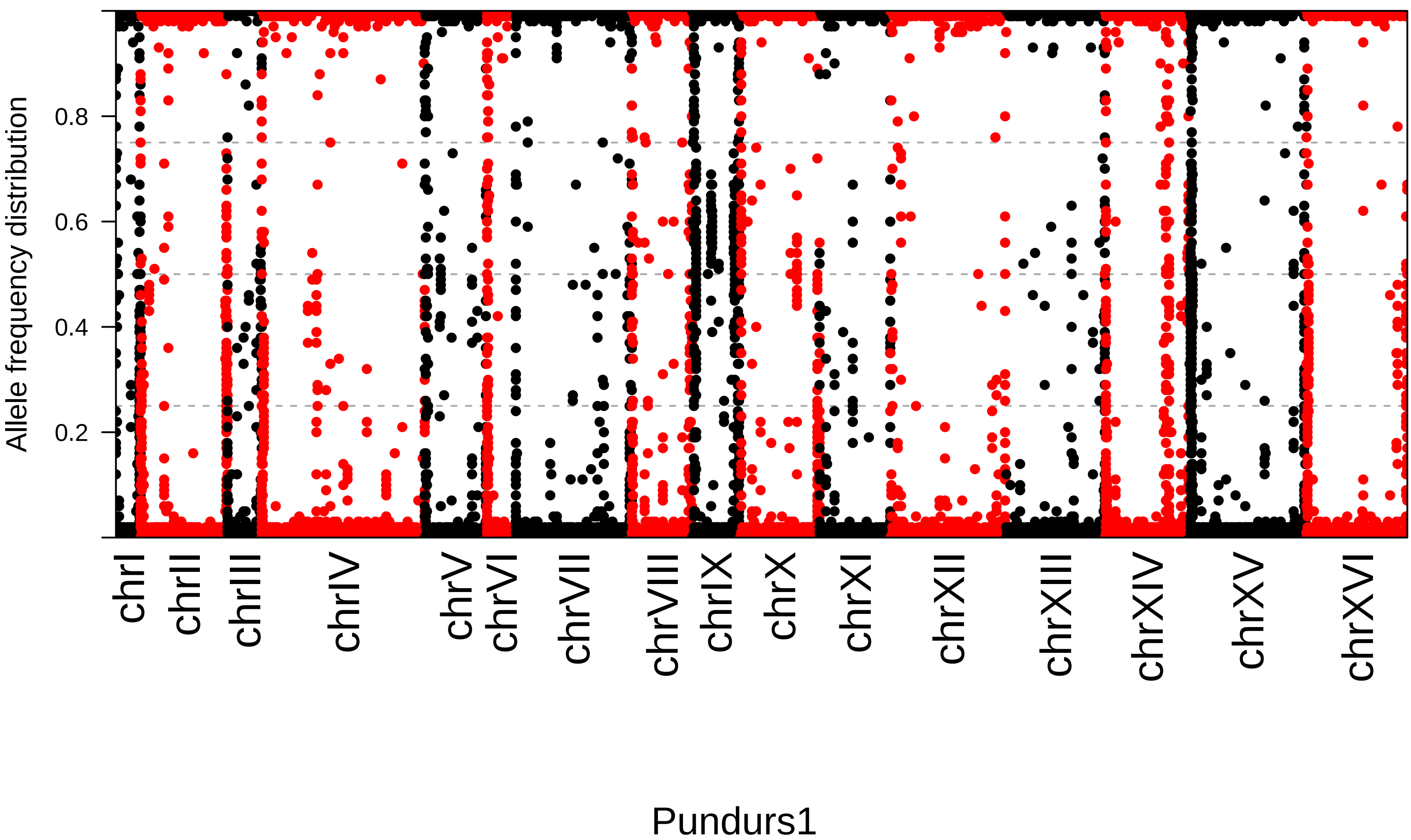

Supplementary Figure S3

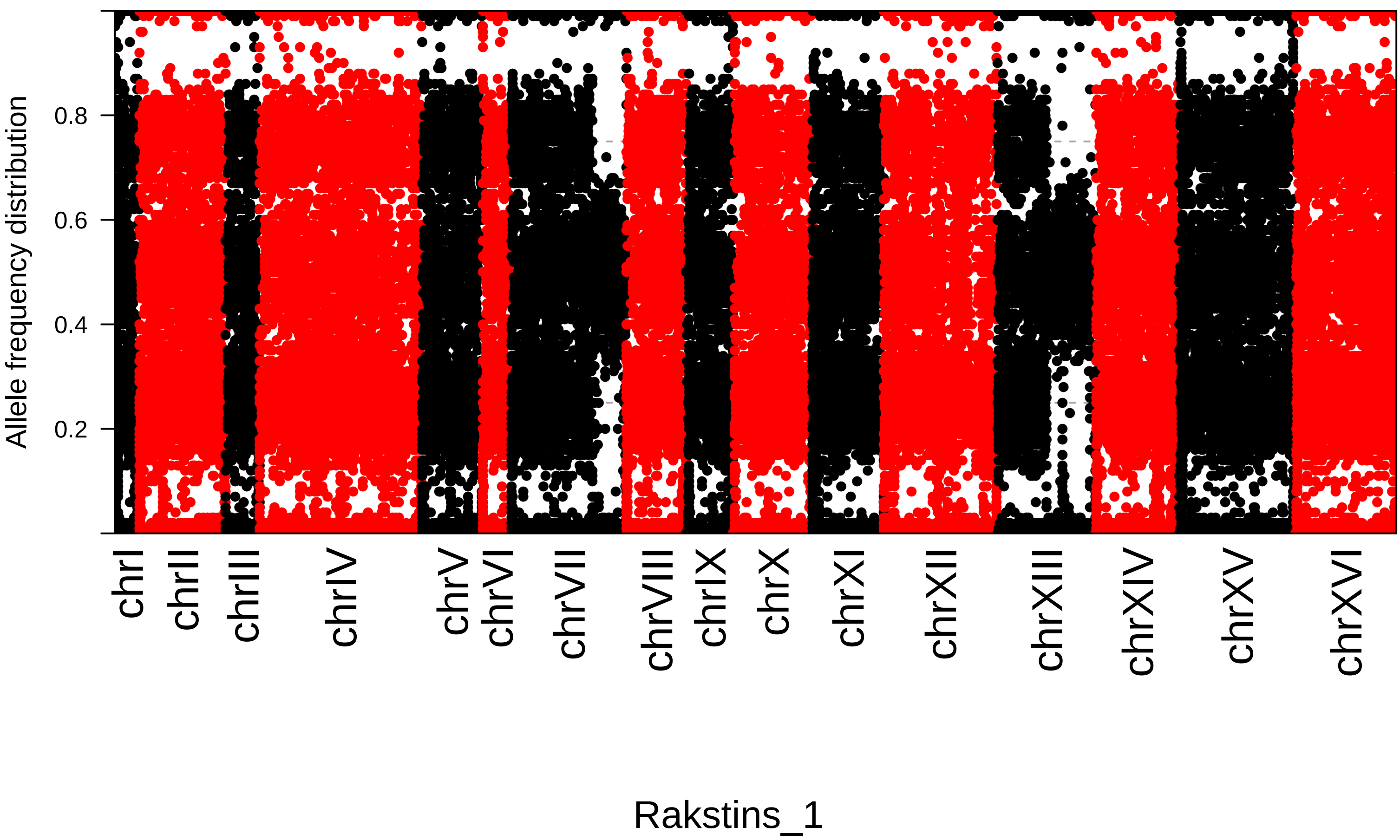

Supplementary Figure S3

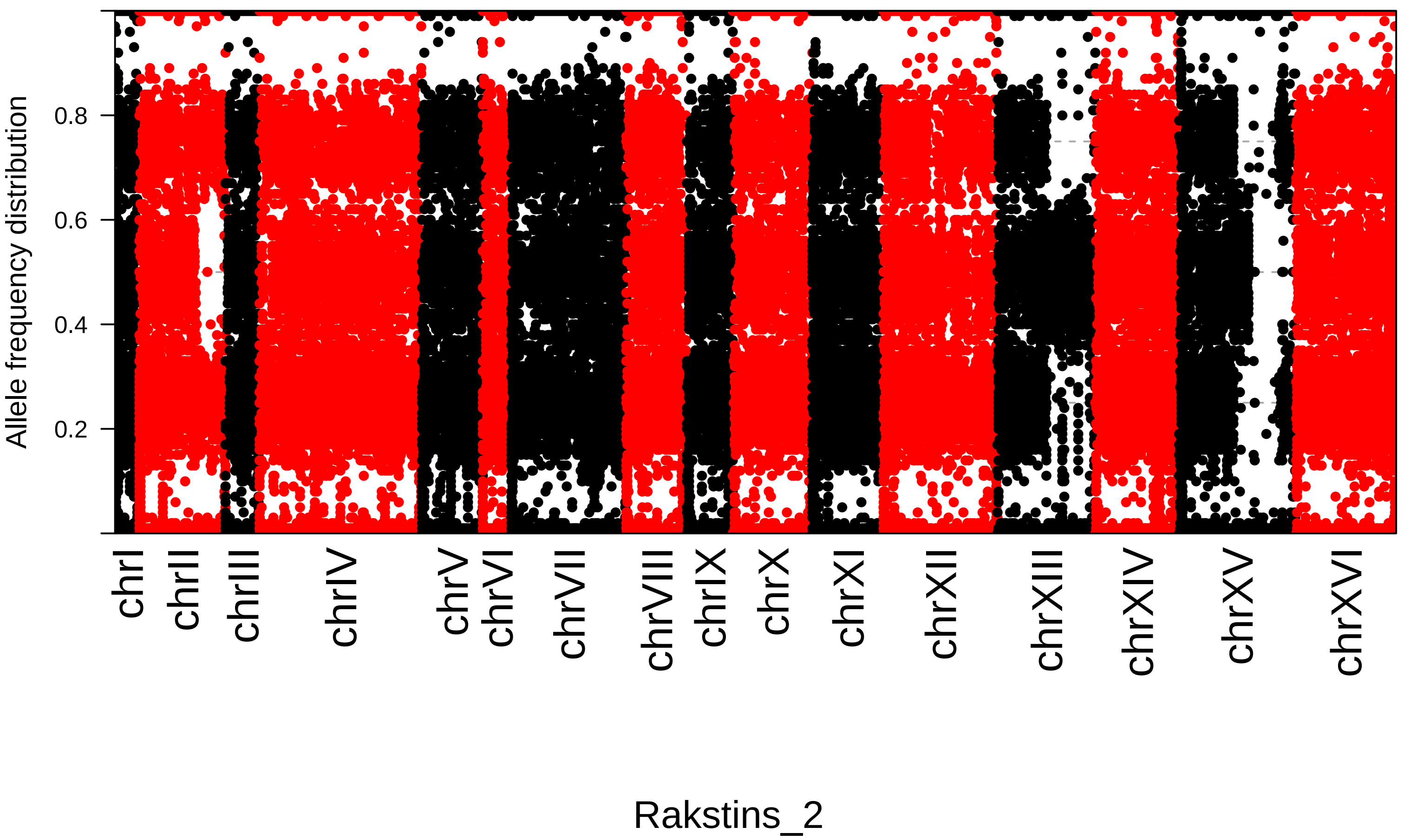

Supplementary Figure S3

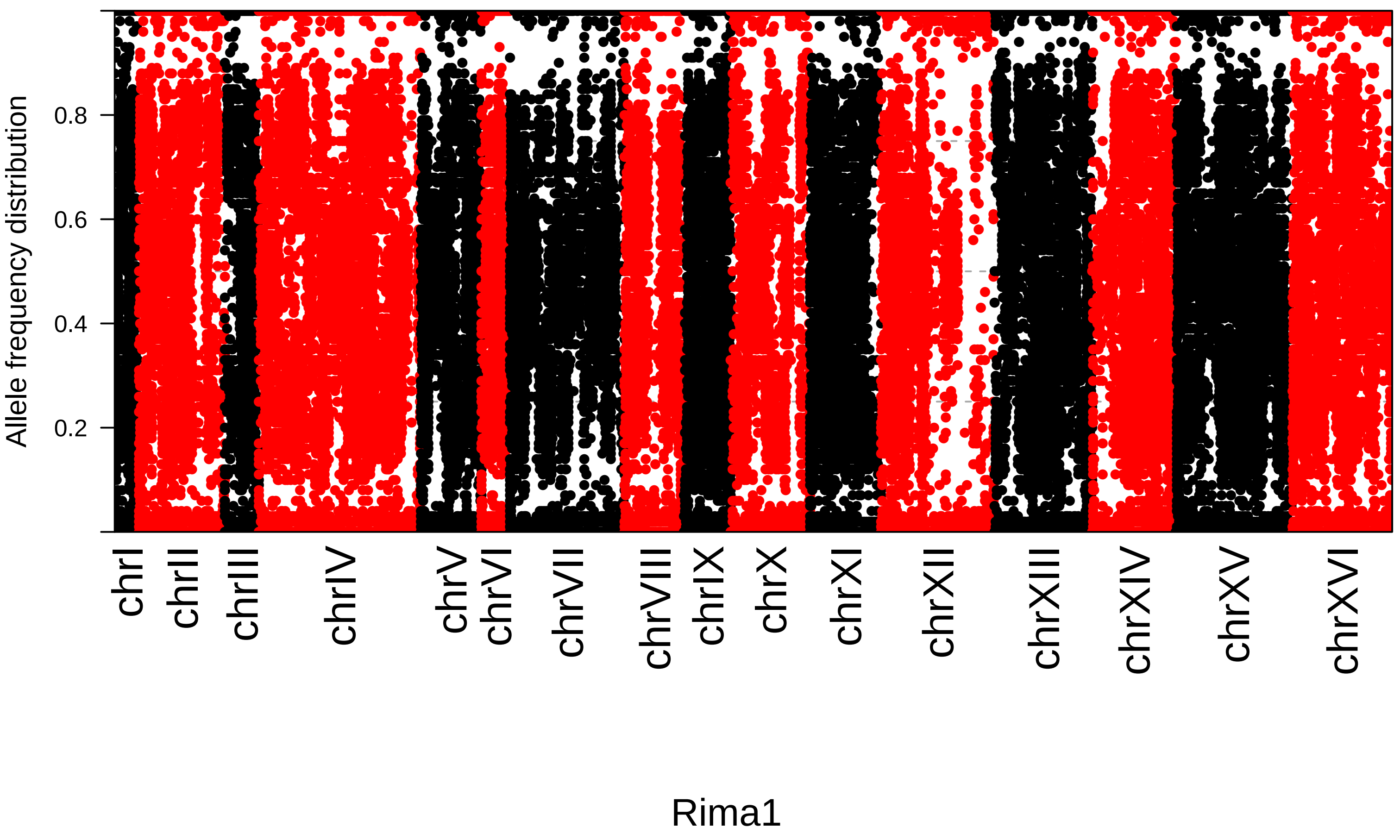

Supplementary Figure S3

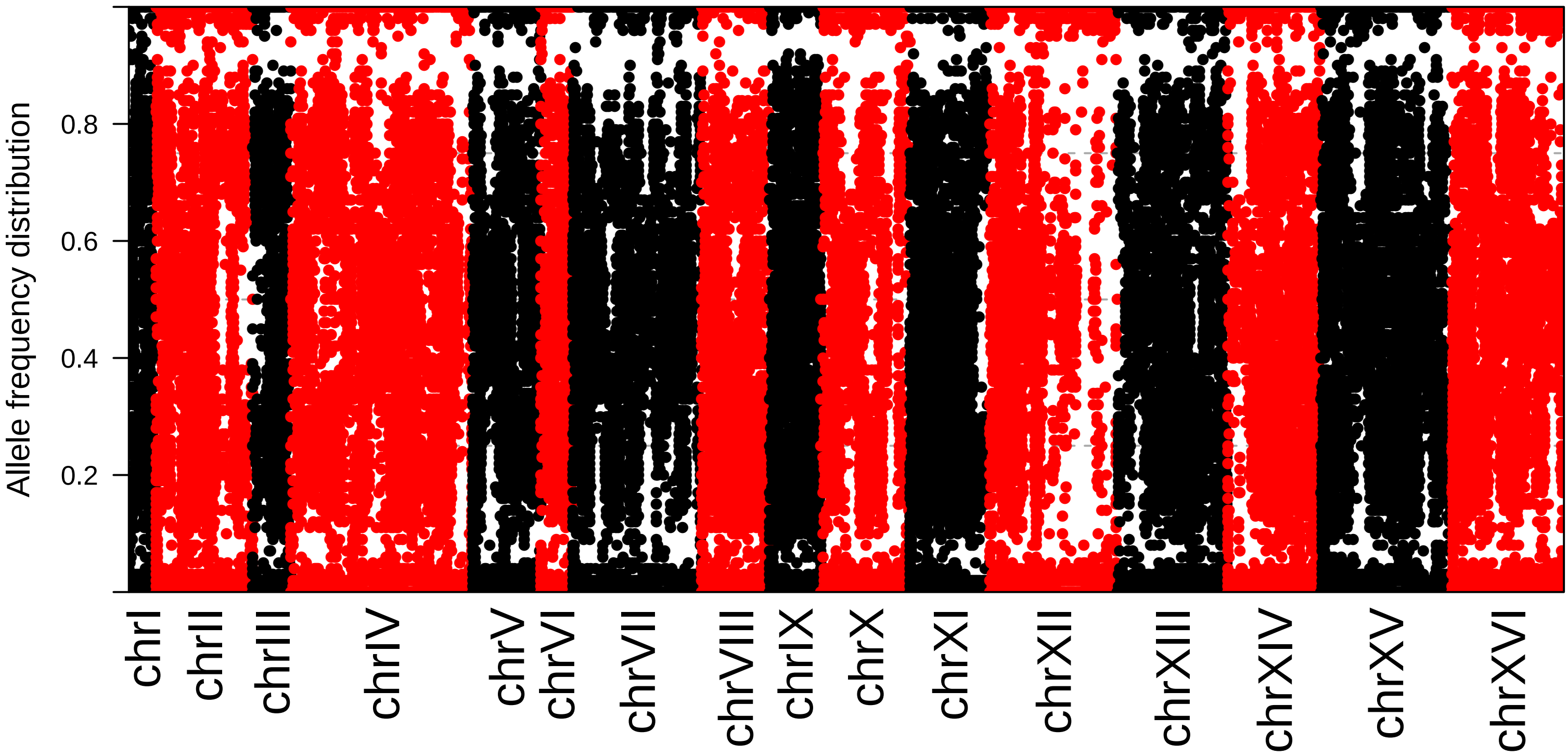

Rima2

Supplementary Figure S3

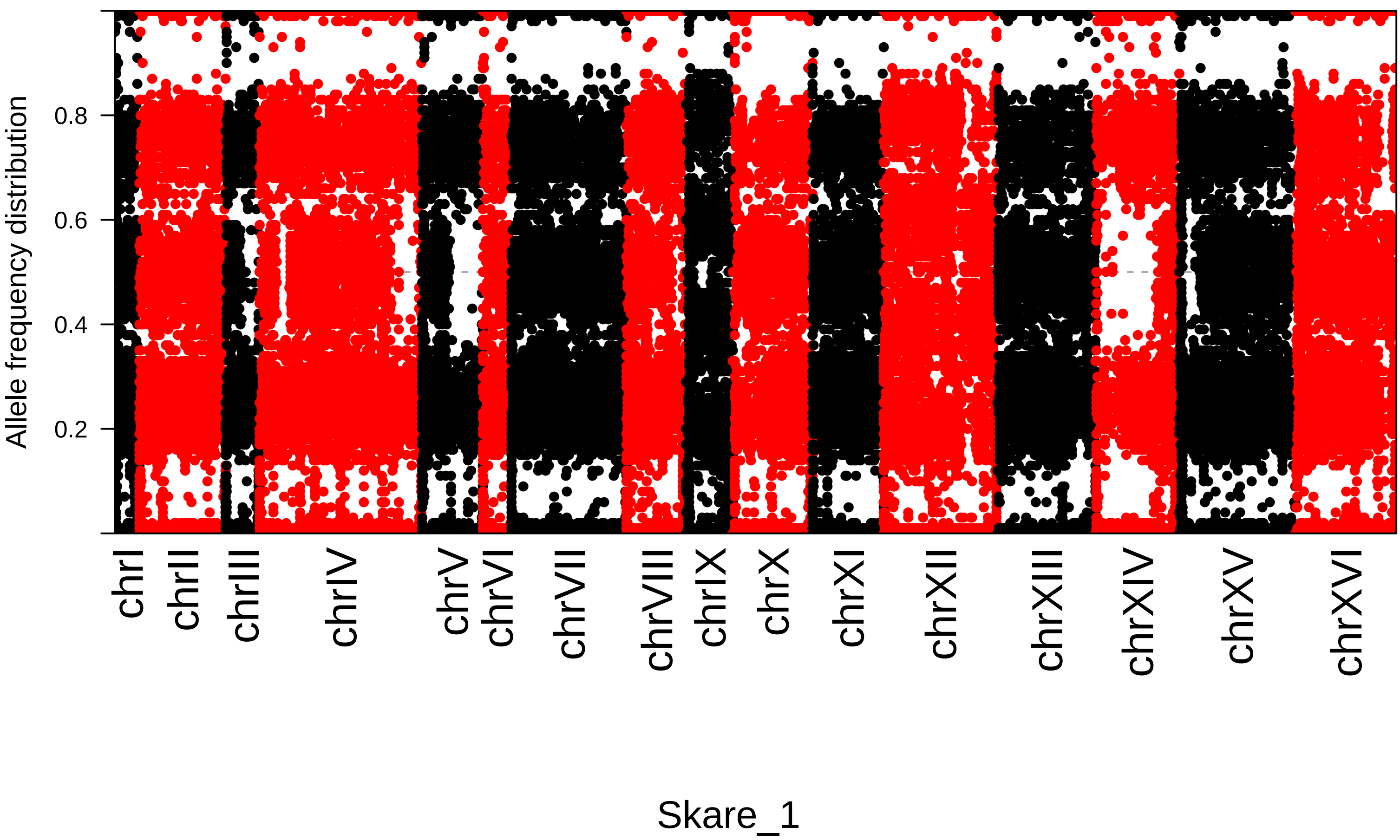

Supplementary Figure S3

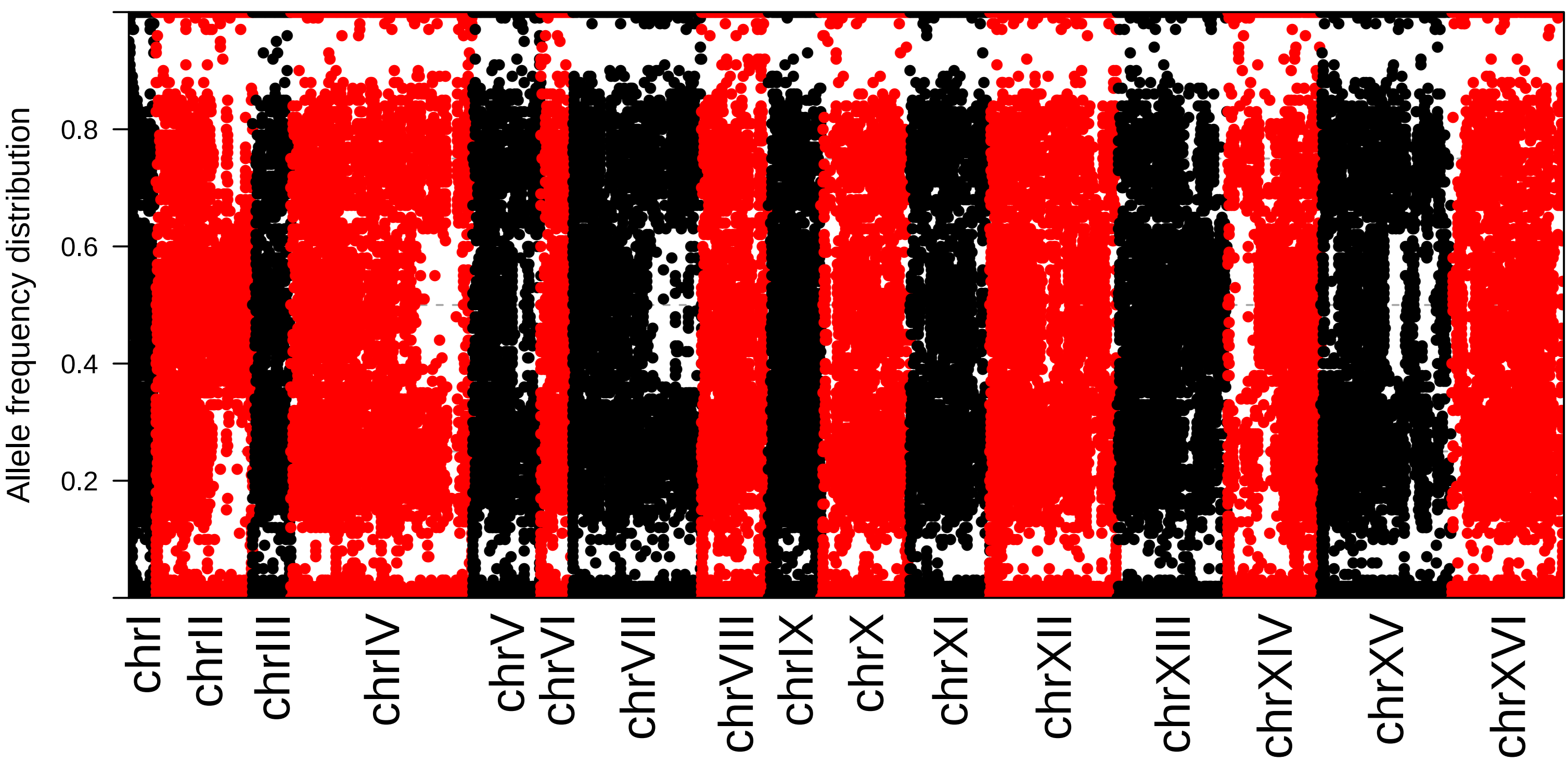

Skrindo2

Supplementary Figure S3

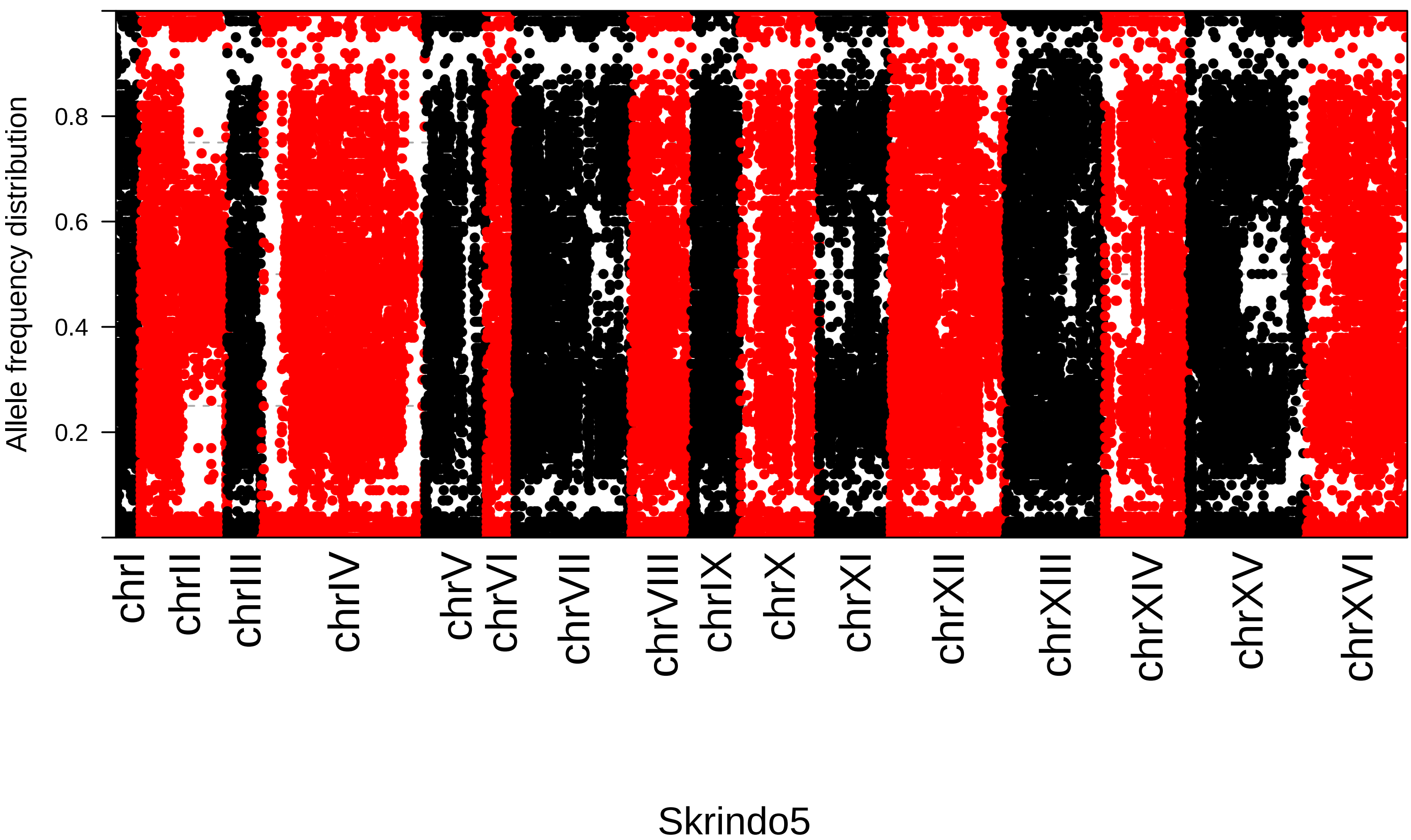

Supplementary Figure S3

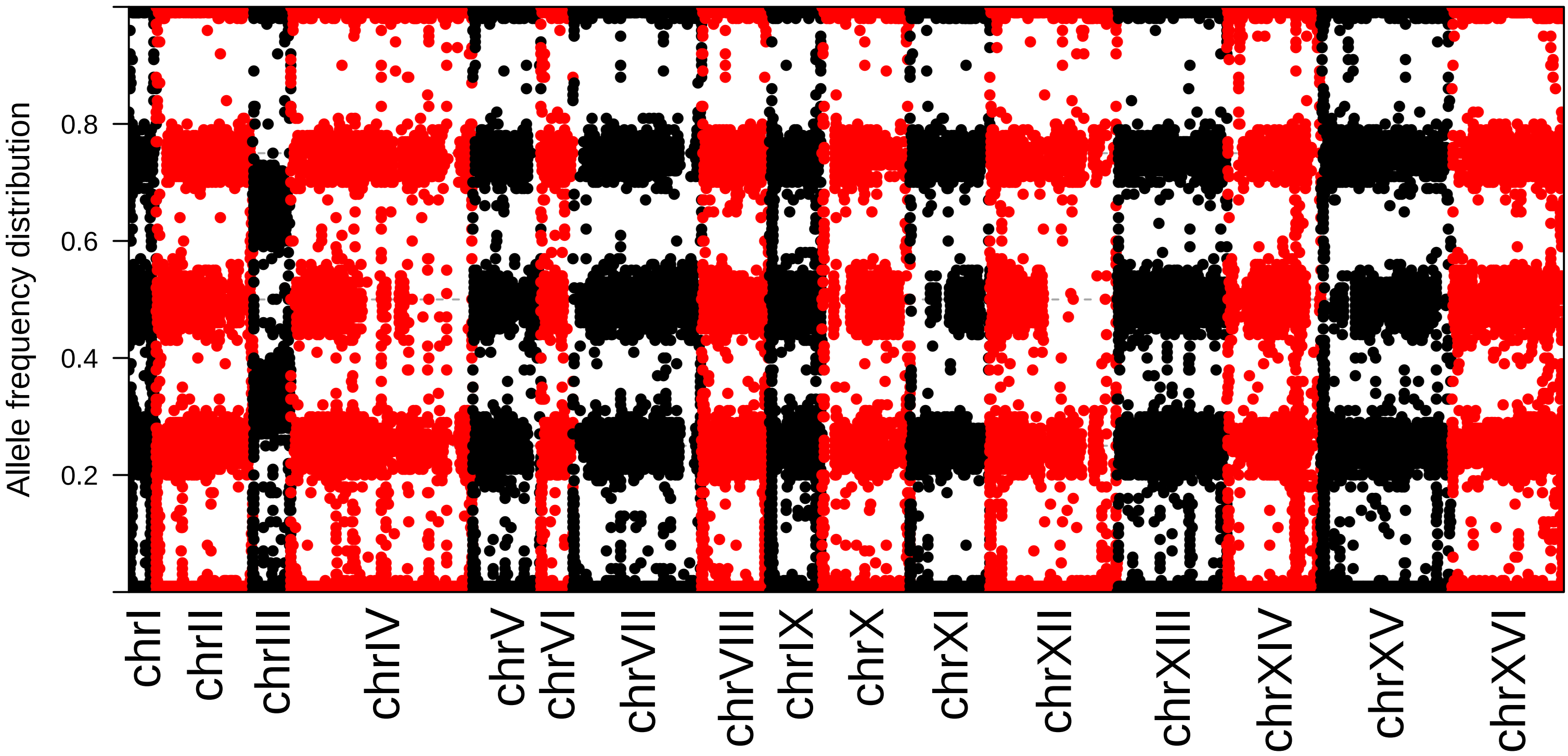

Stordal_Ebbegarden_1

Supplementary Figure S3

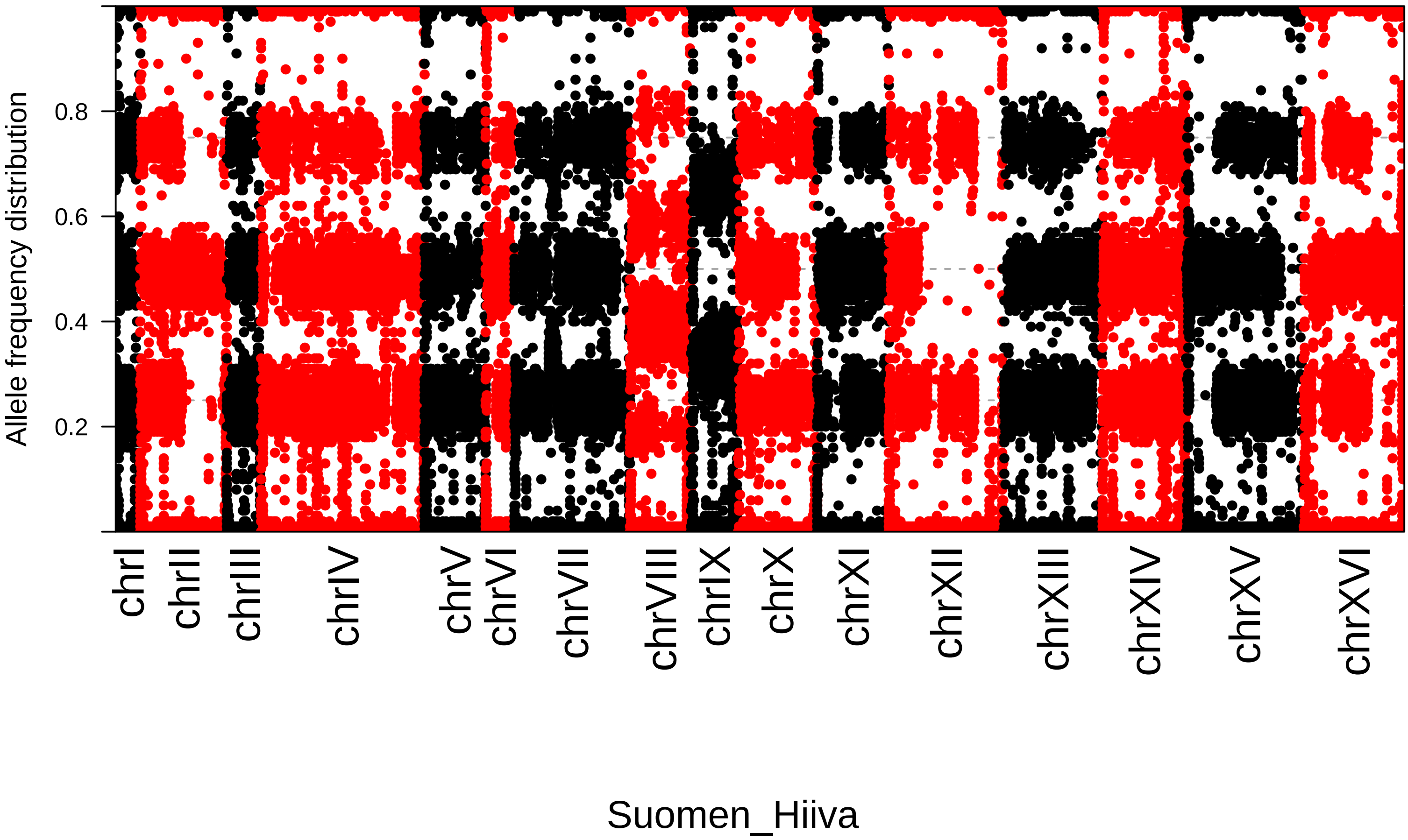

Supplementary Figure S3

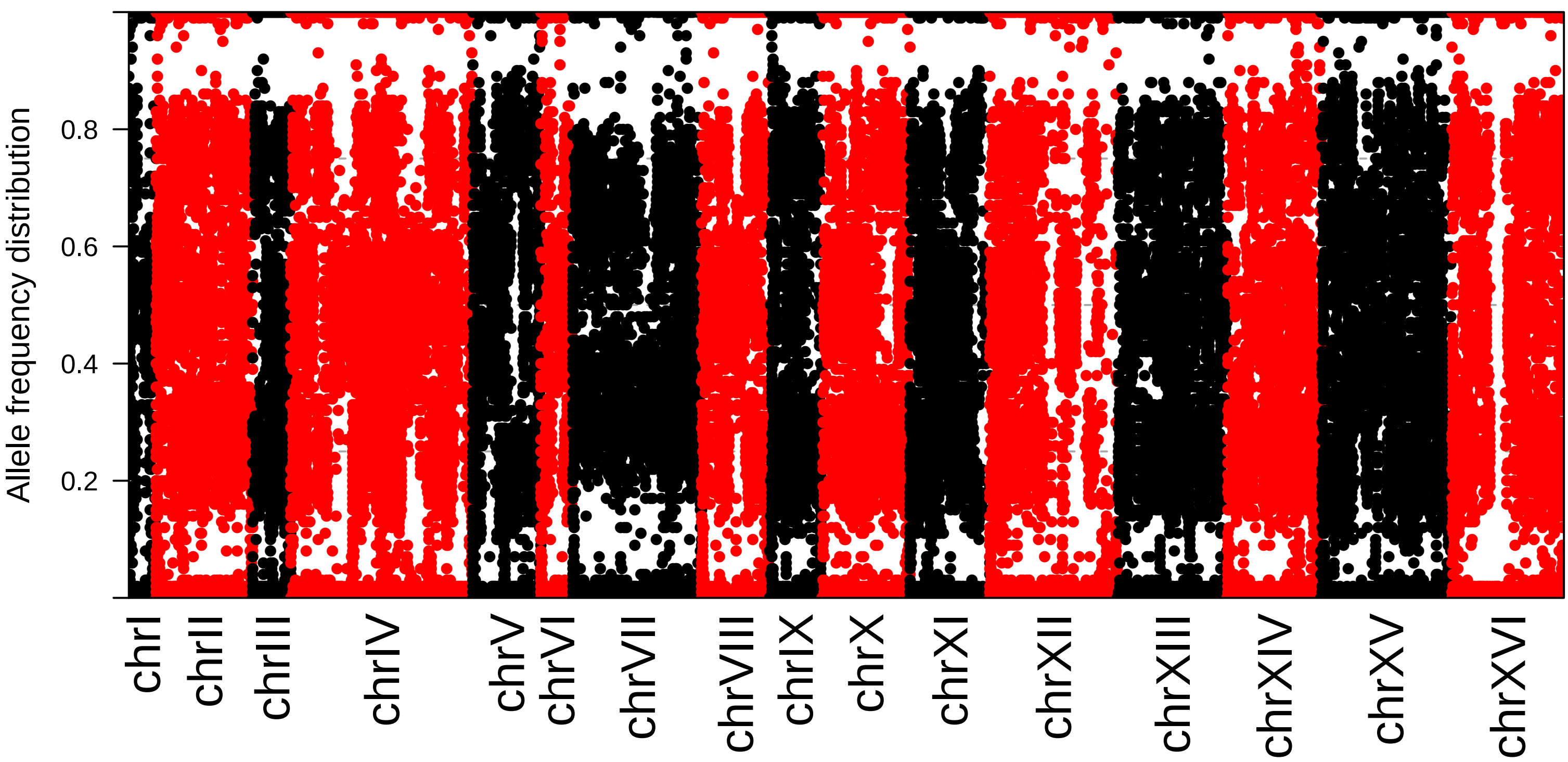

Tinn_1

Supplementary Figure S3

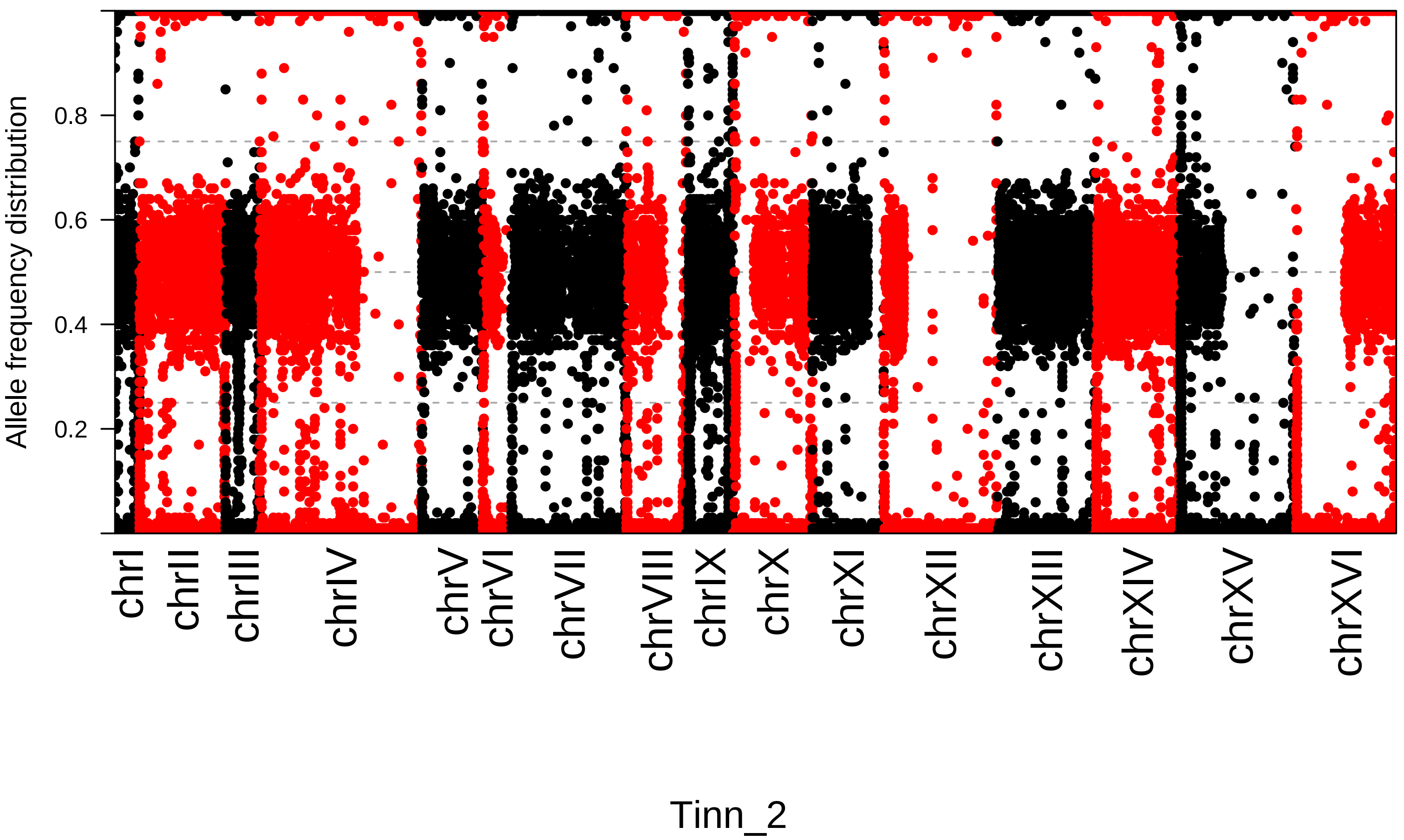

Supplementary Figure S3

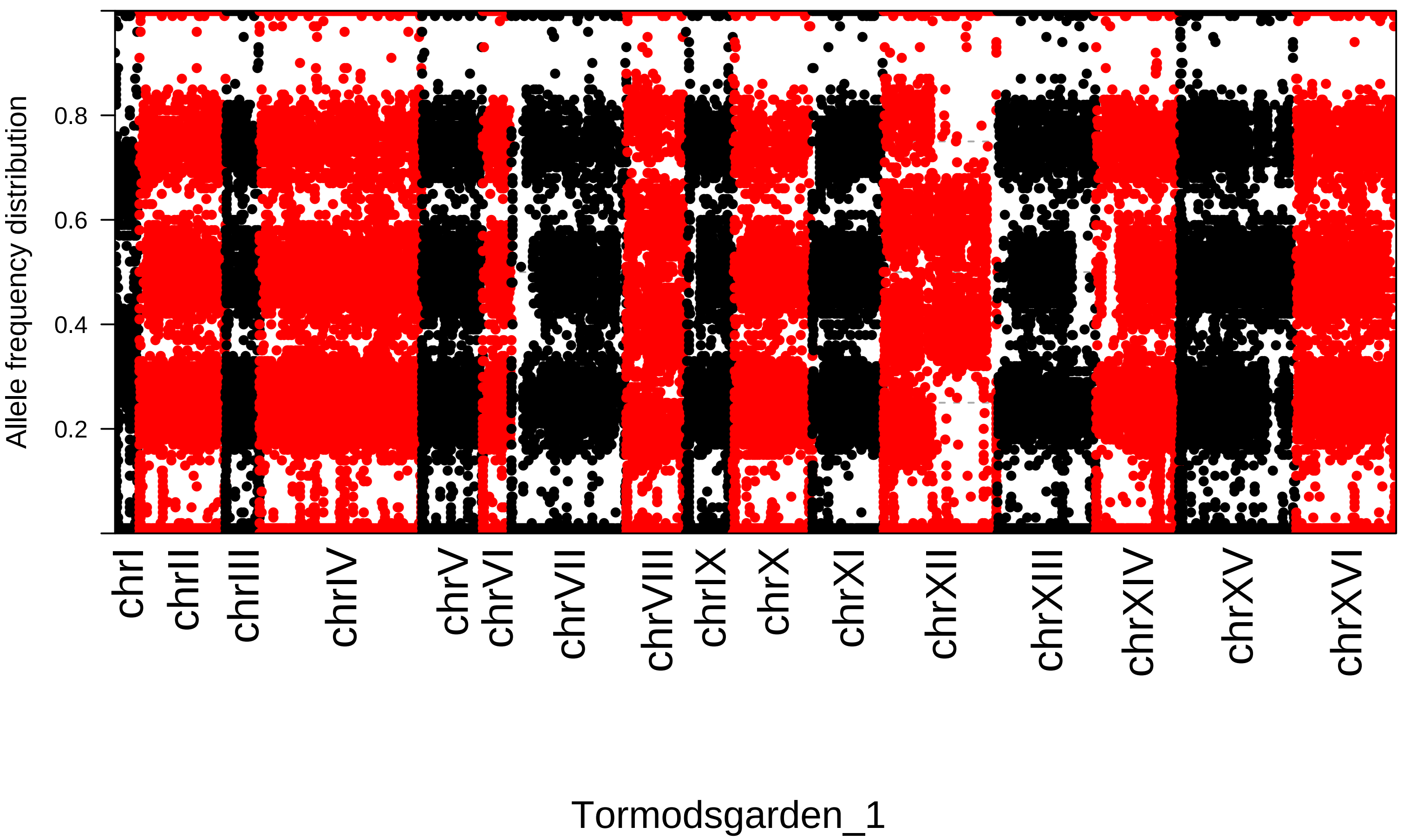

Supplementary Figure S3

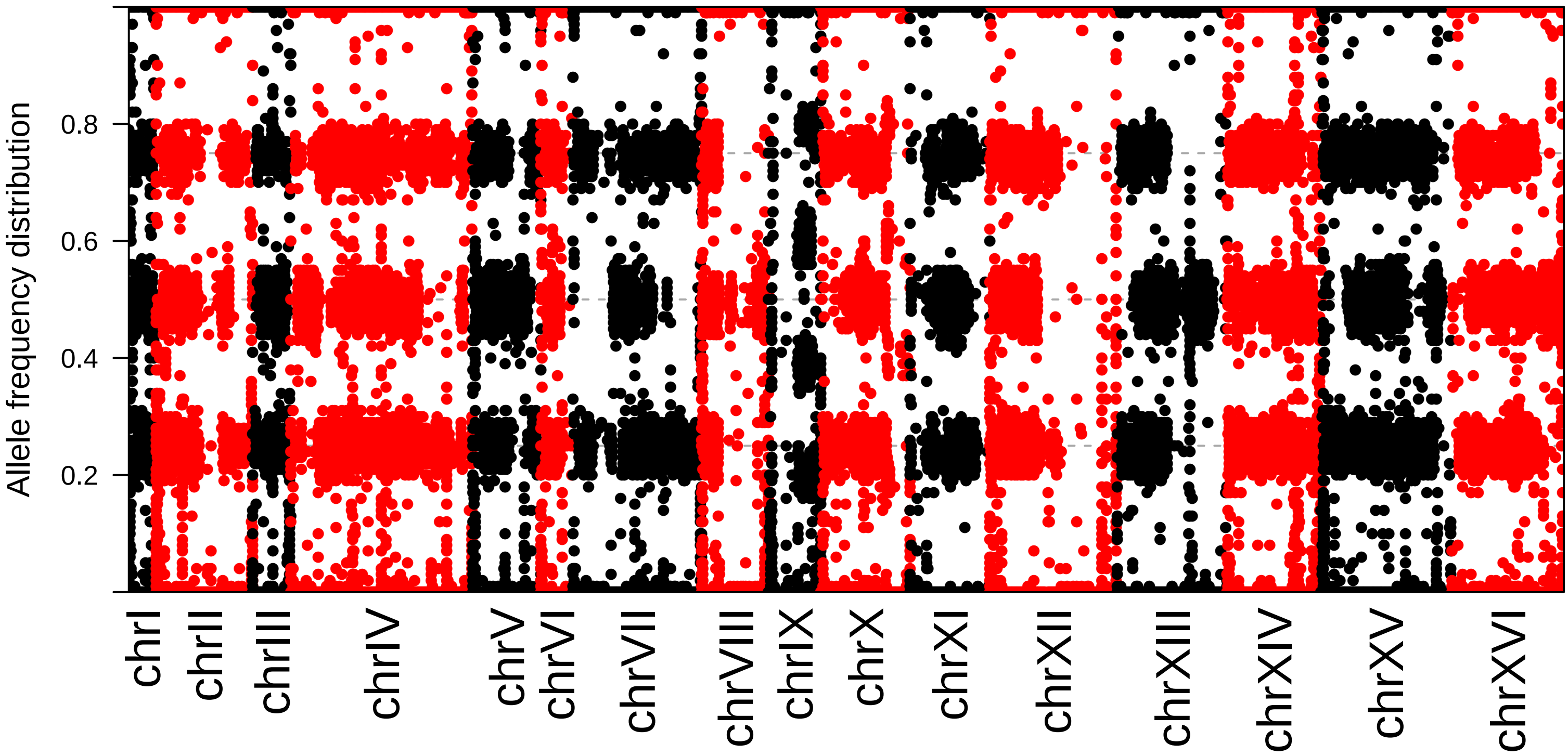

Uberweizen

Supplementary Figure S3

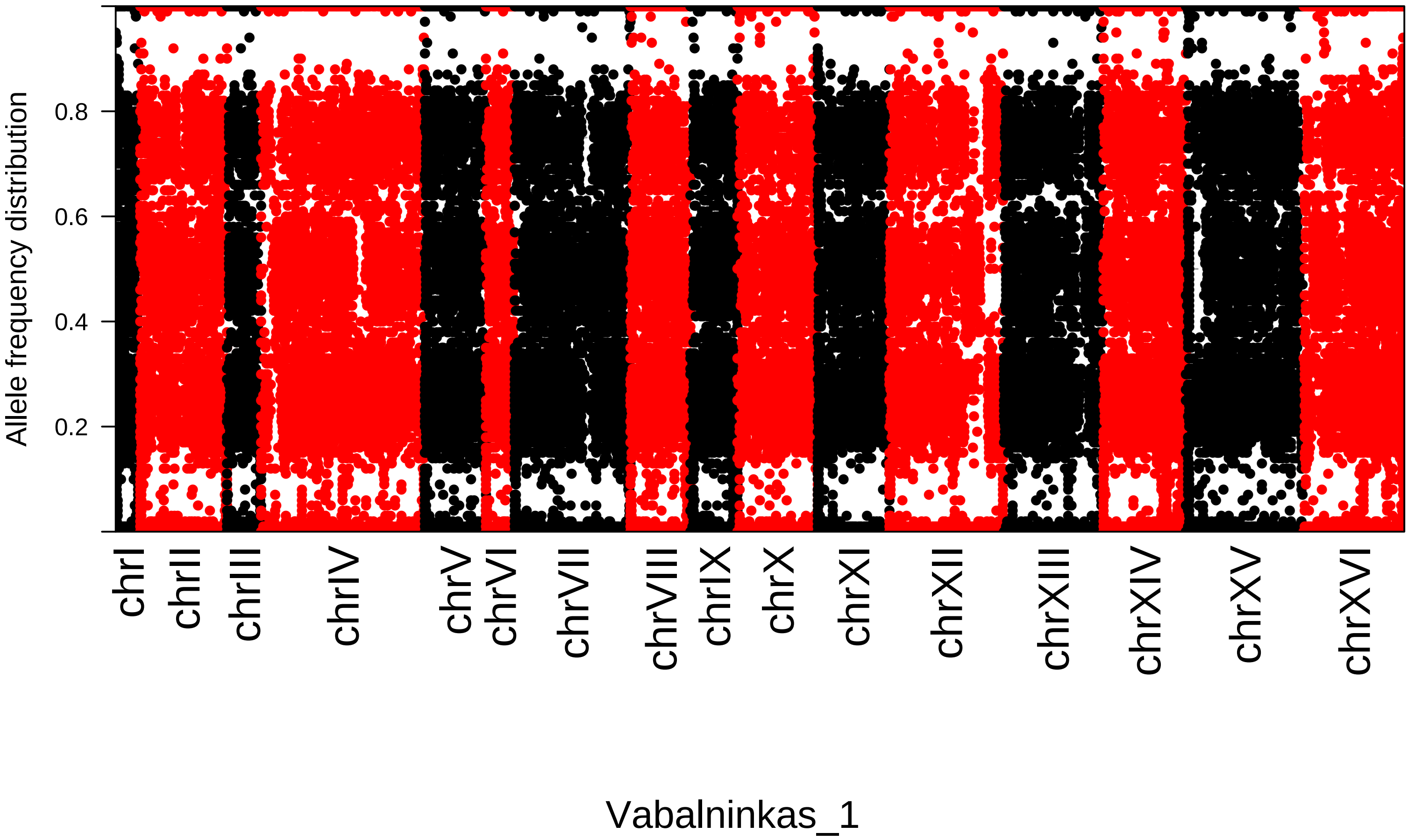

Supplementary Figure S3

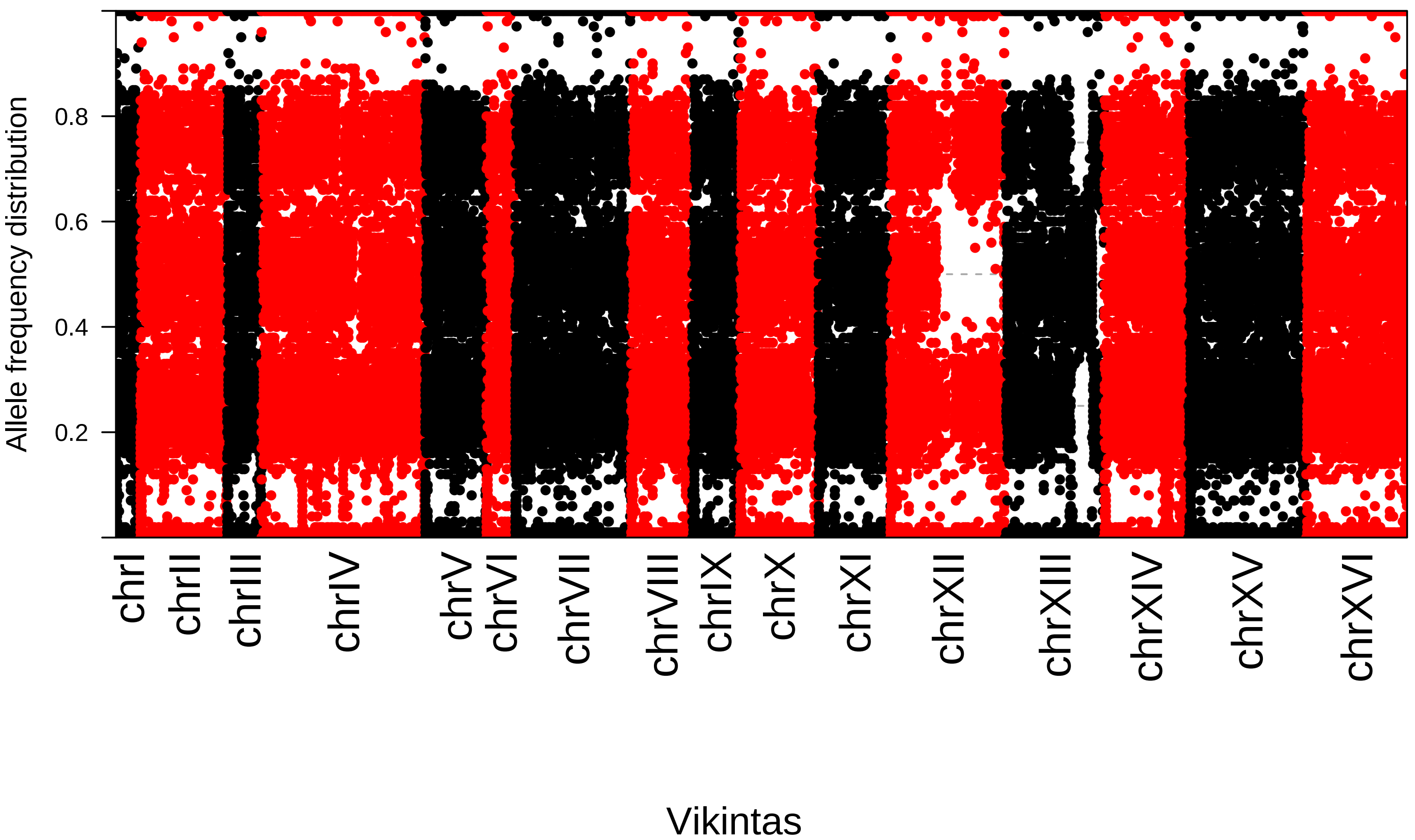

Supplementary Figure S3

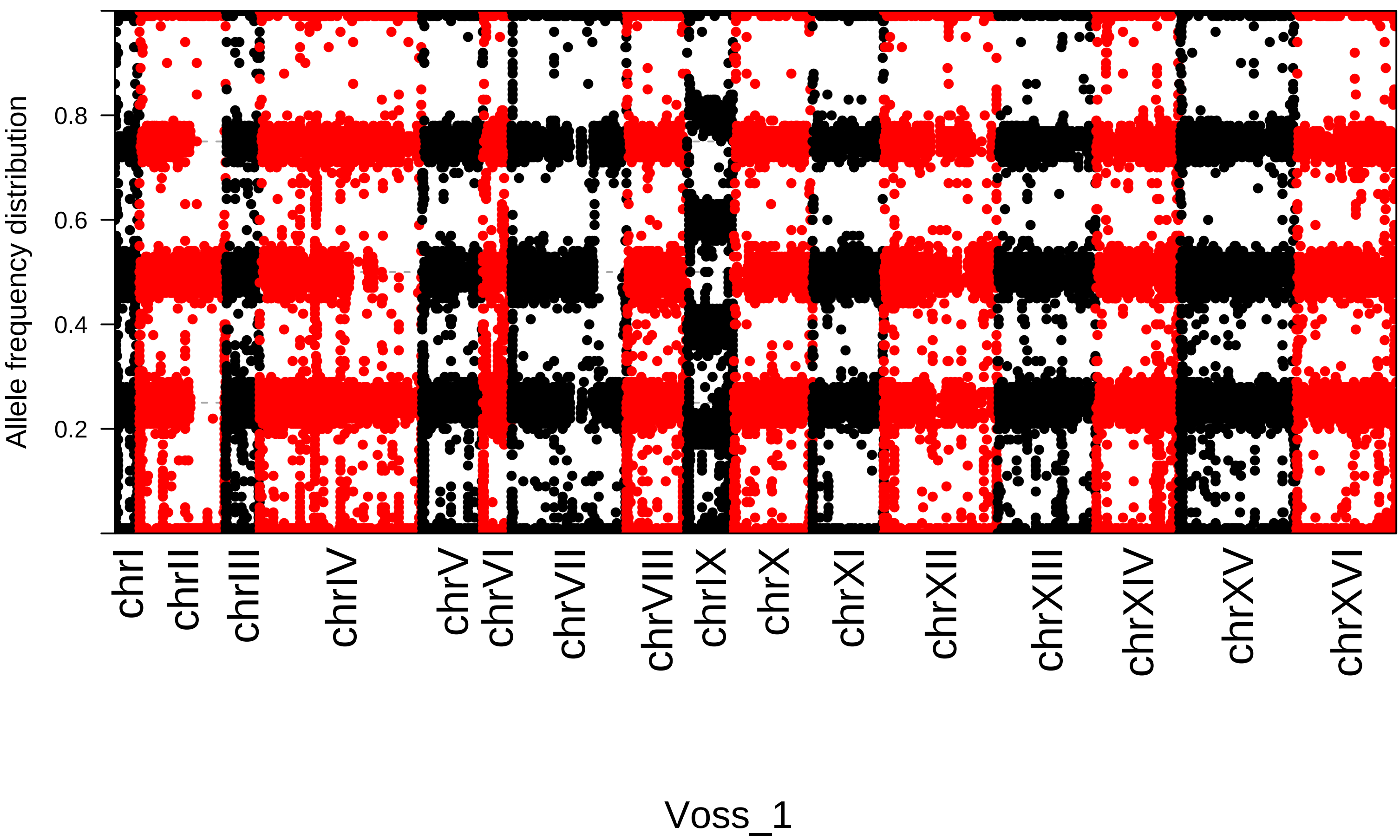

Supplementary Figure S3

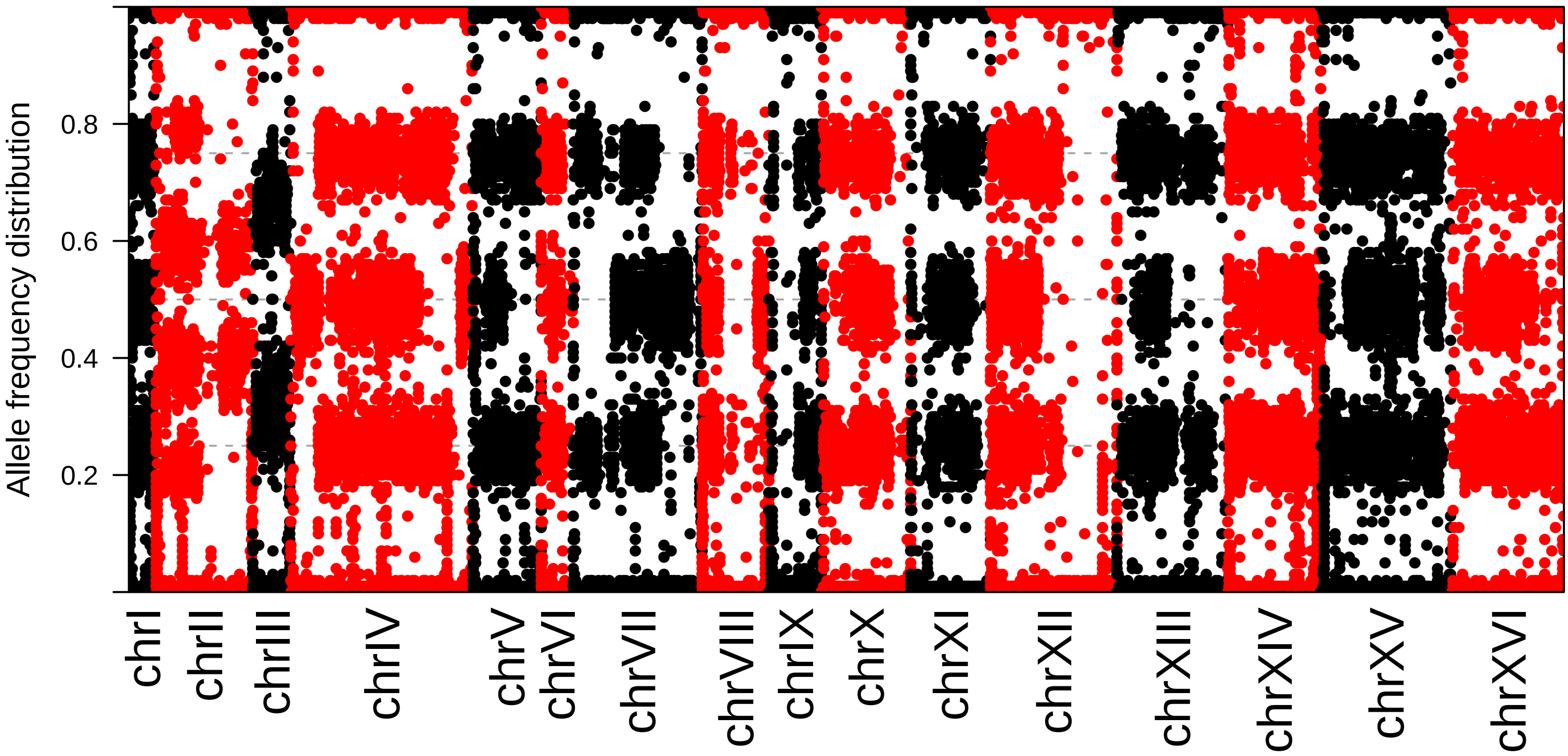

Weizen_1

Supplementary Figure S3

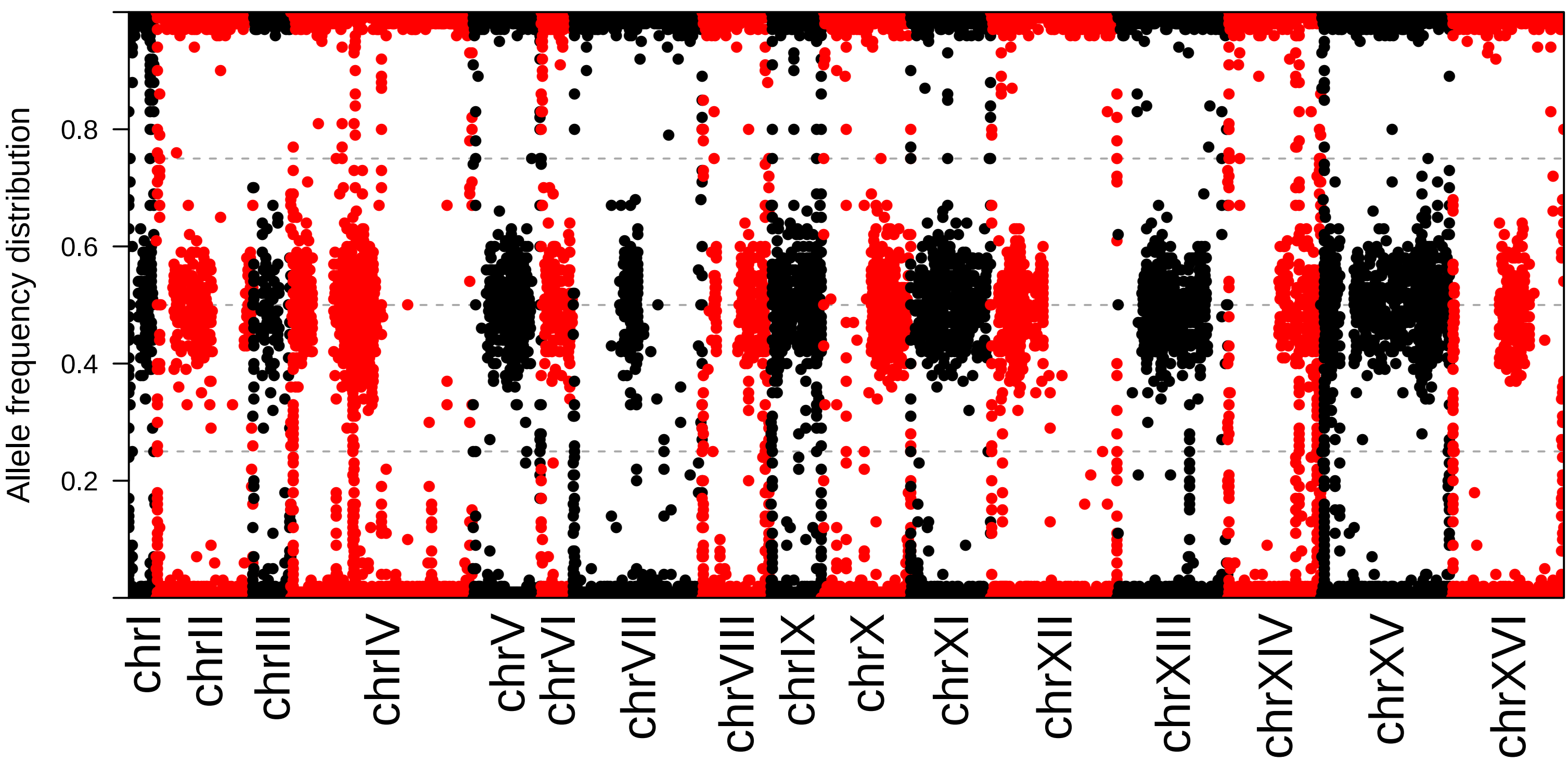

wine003

Supplementary Figure S3

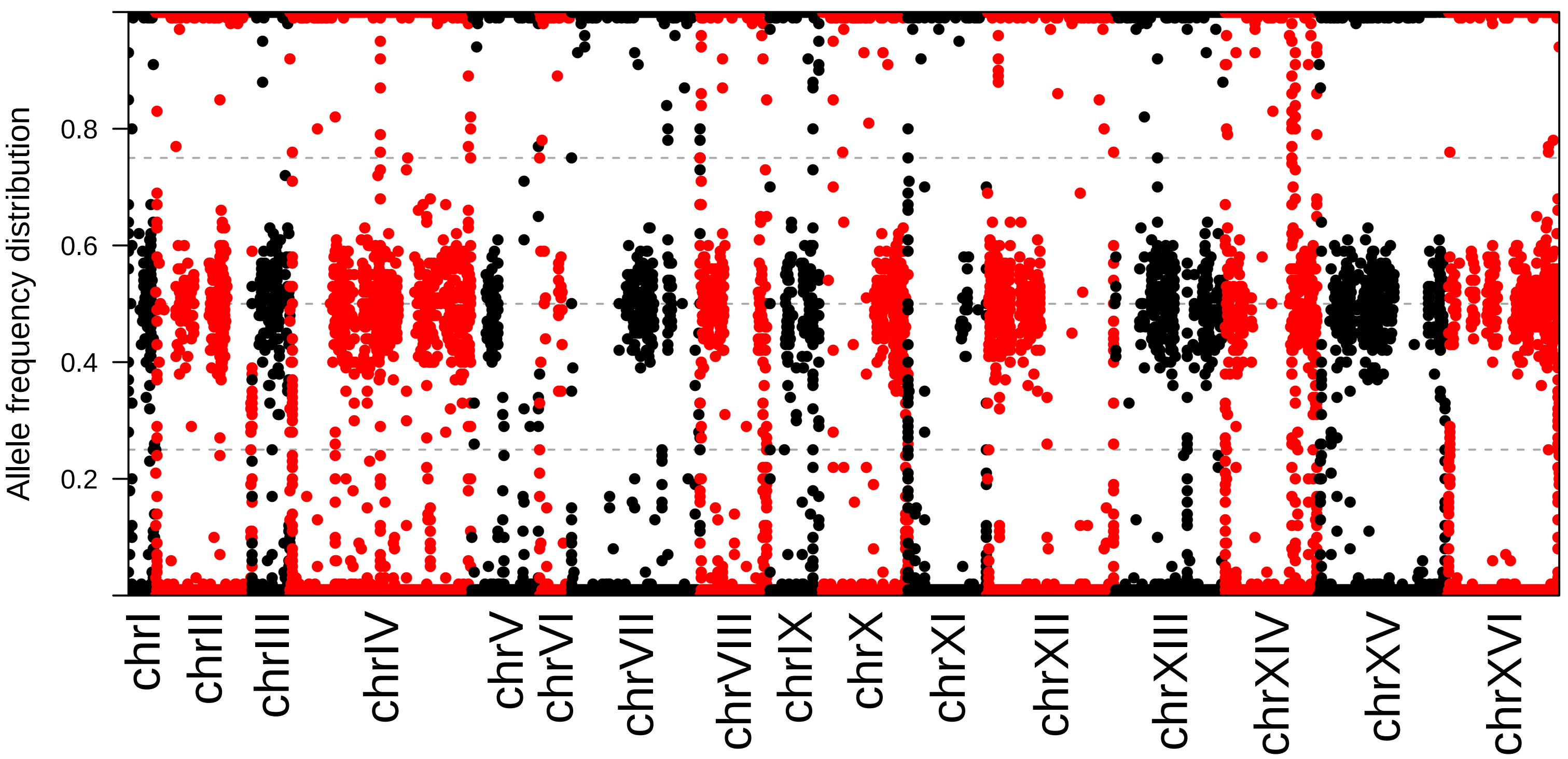

wine005

Supplementary Figure S4A

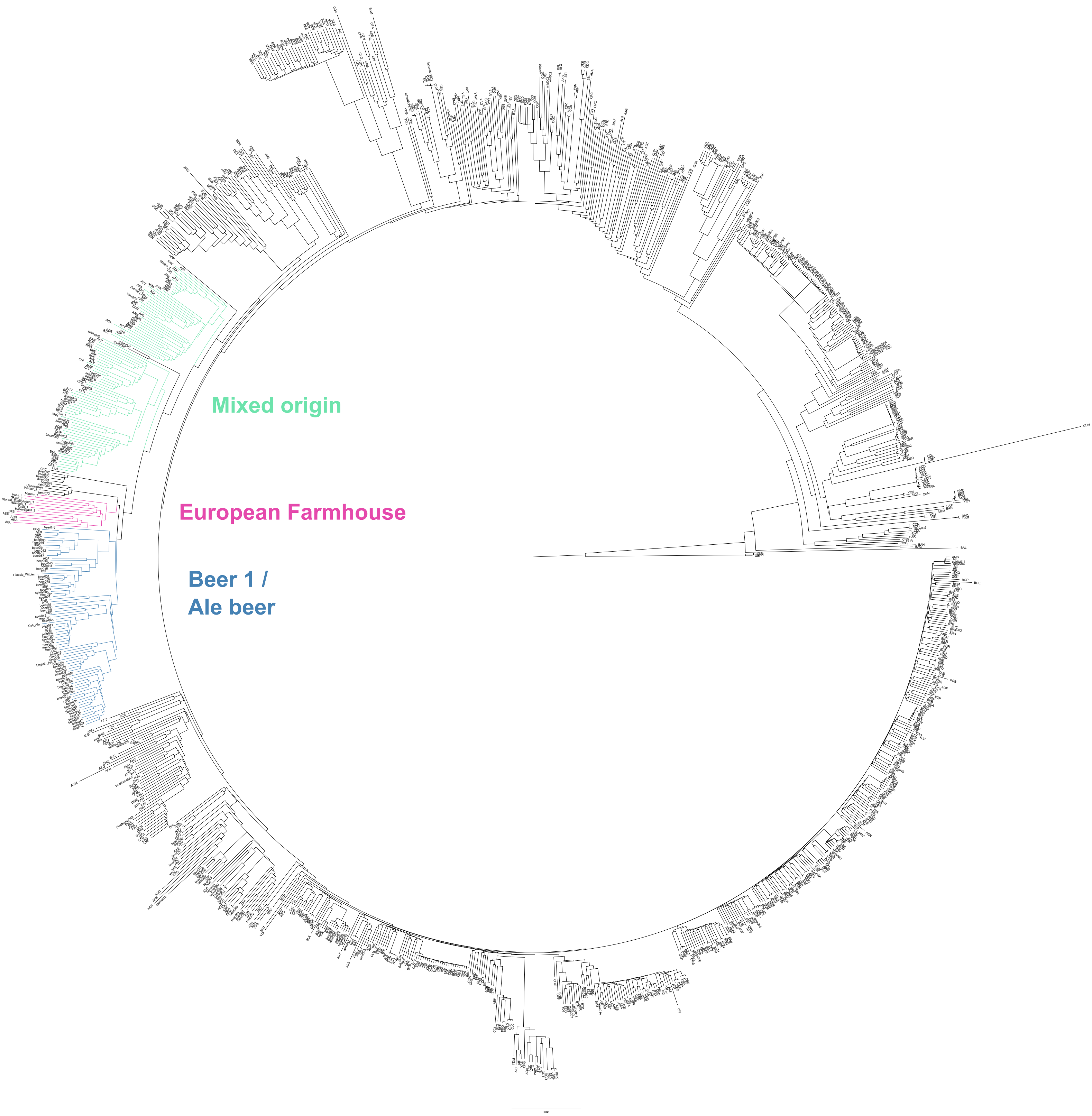

Supplementary Figure S4B

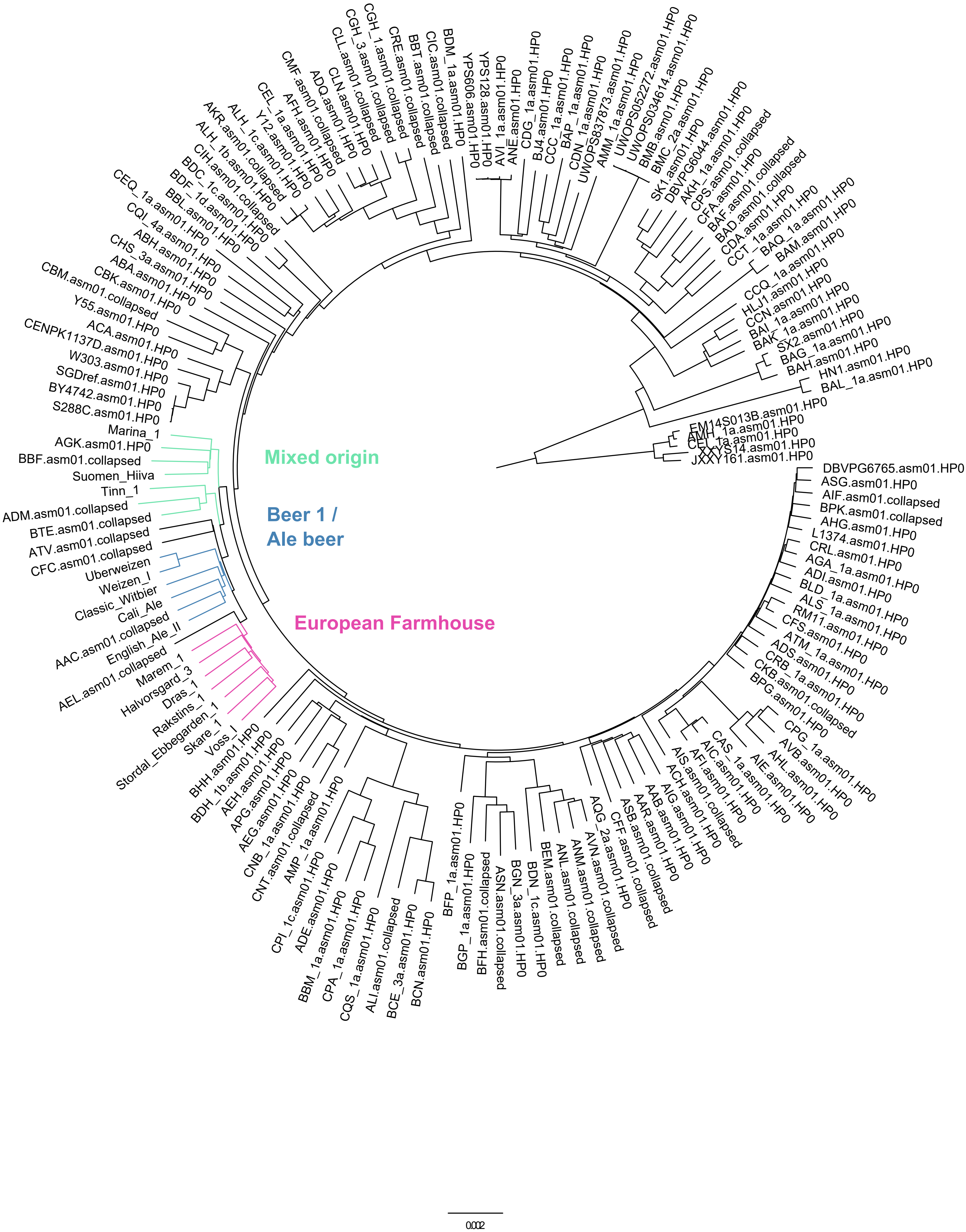

Supplementary Figure S5A

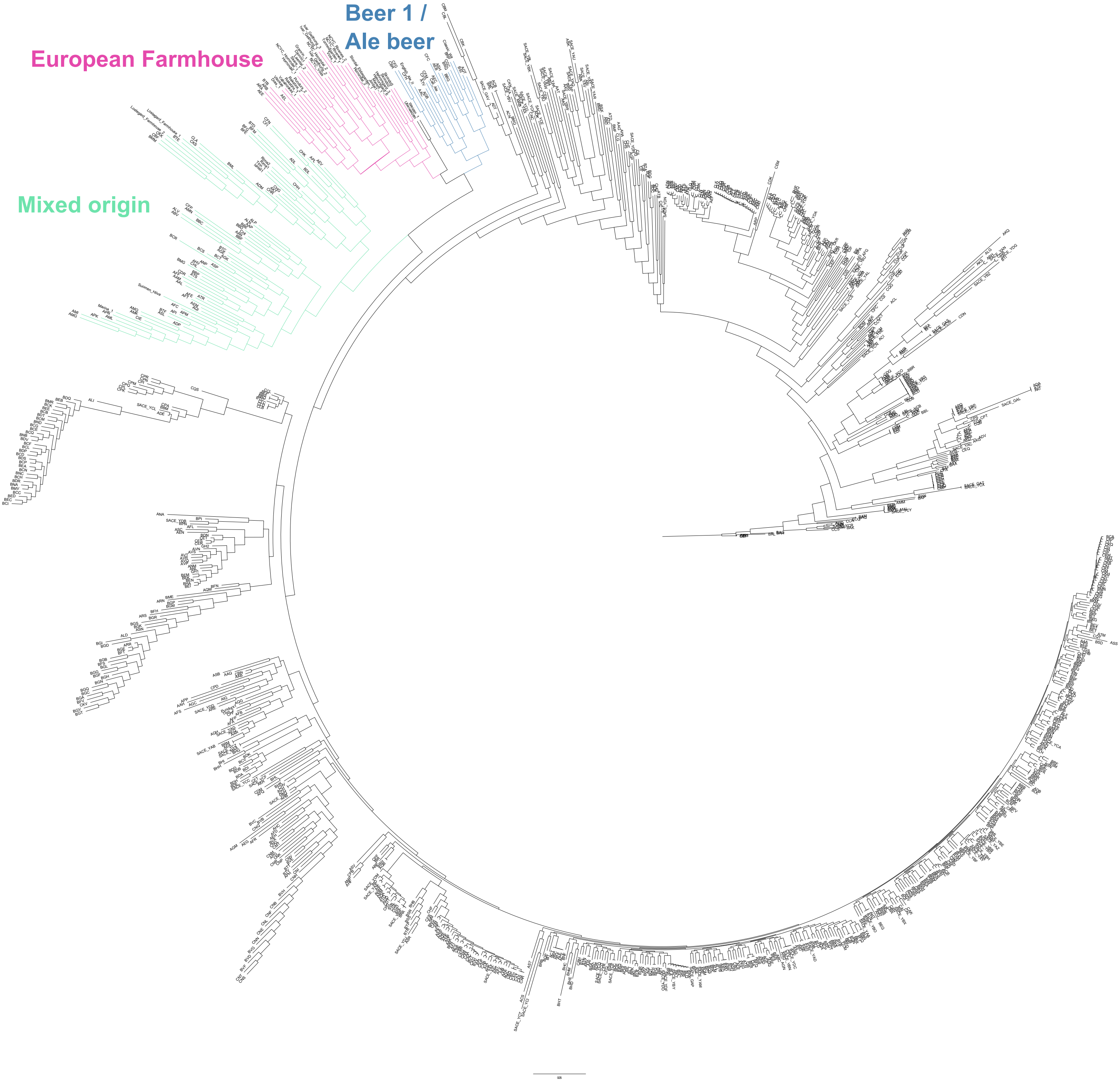

Supplementary Figure S5B

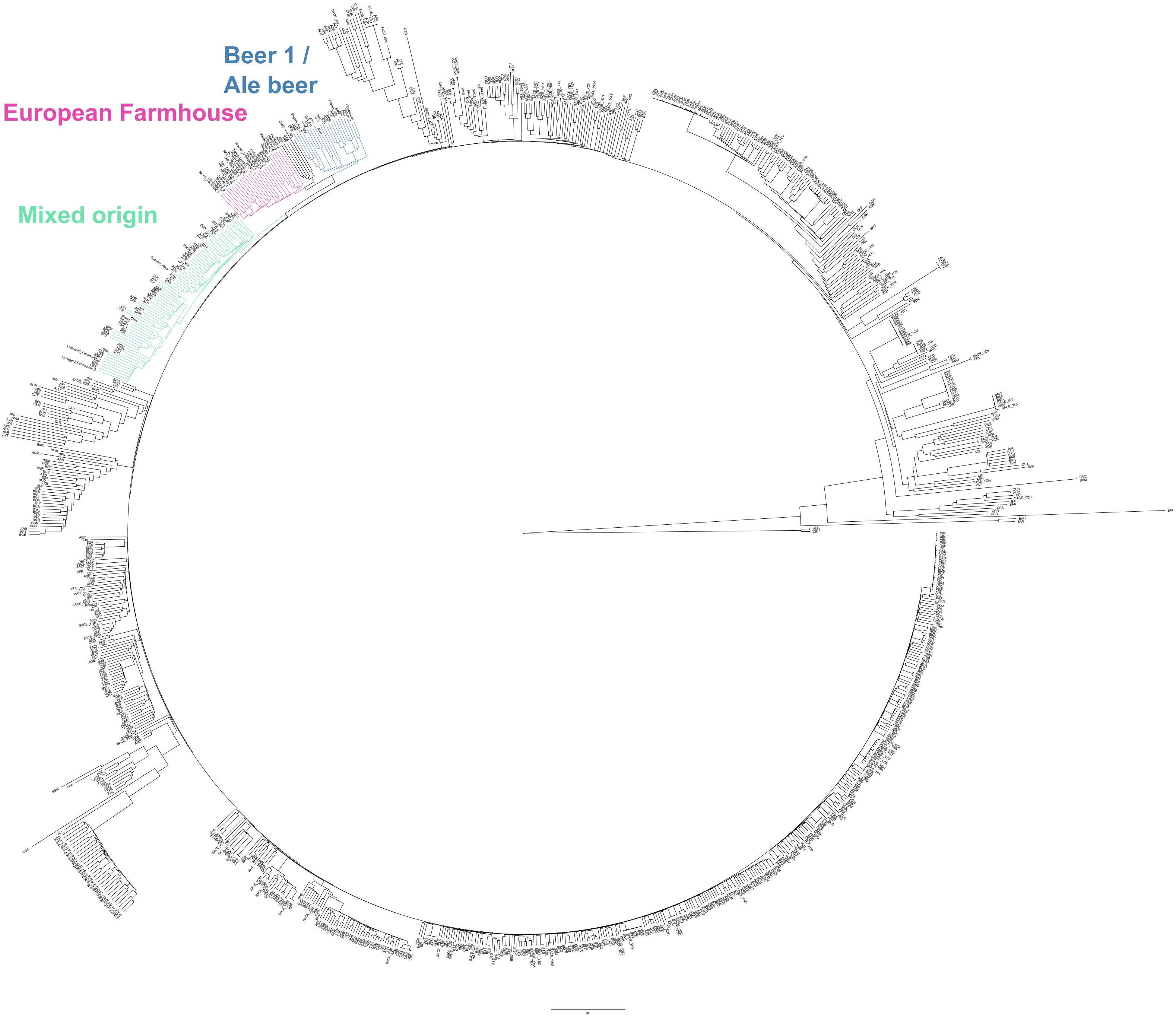

Supplementary Figure S6

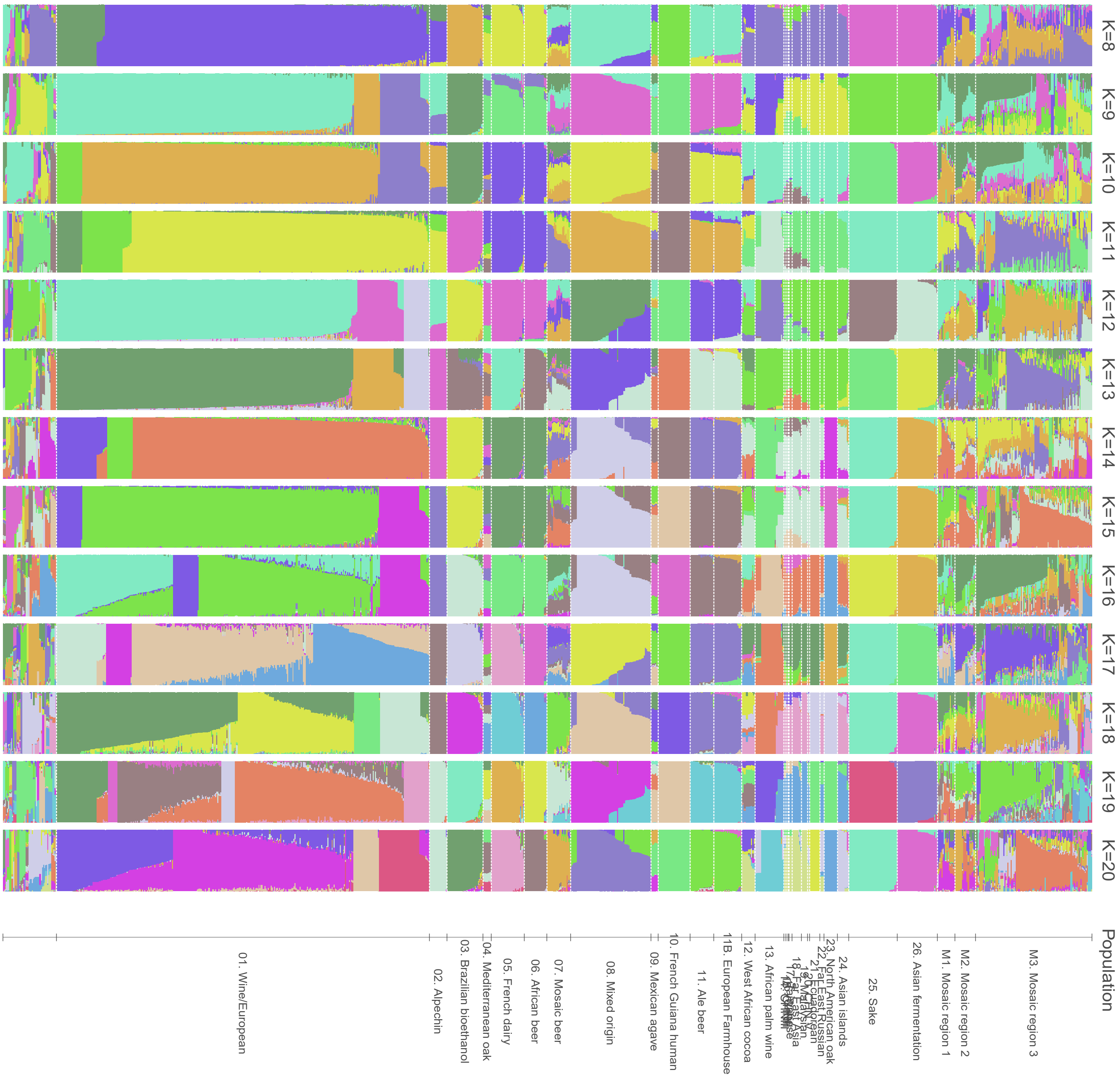

Supplementary Figure S7

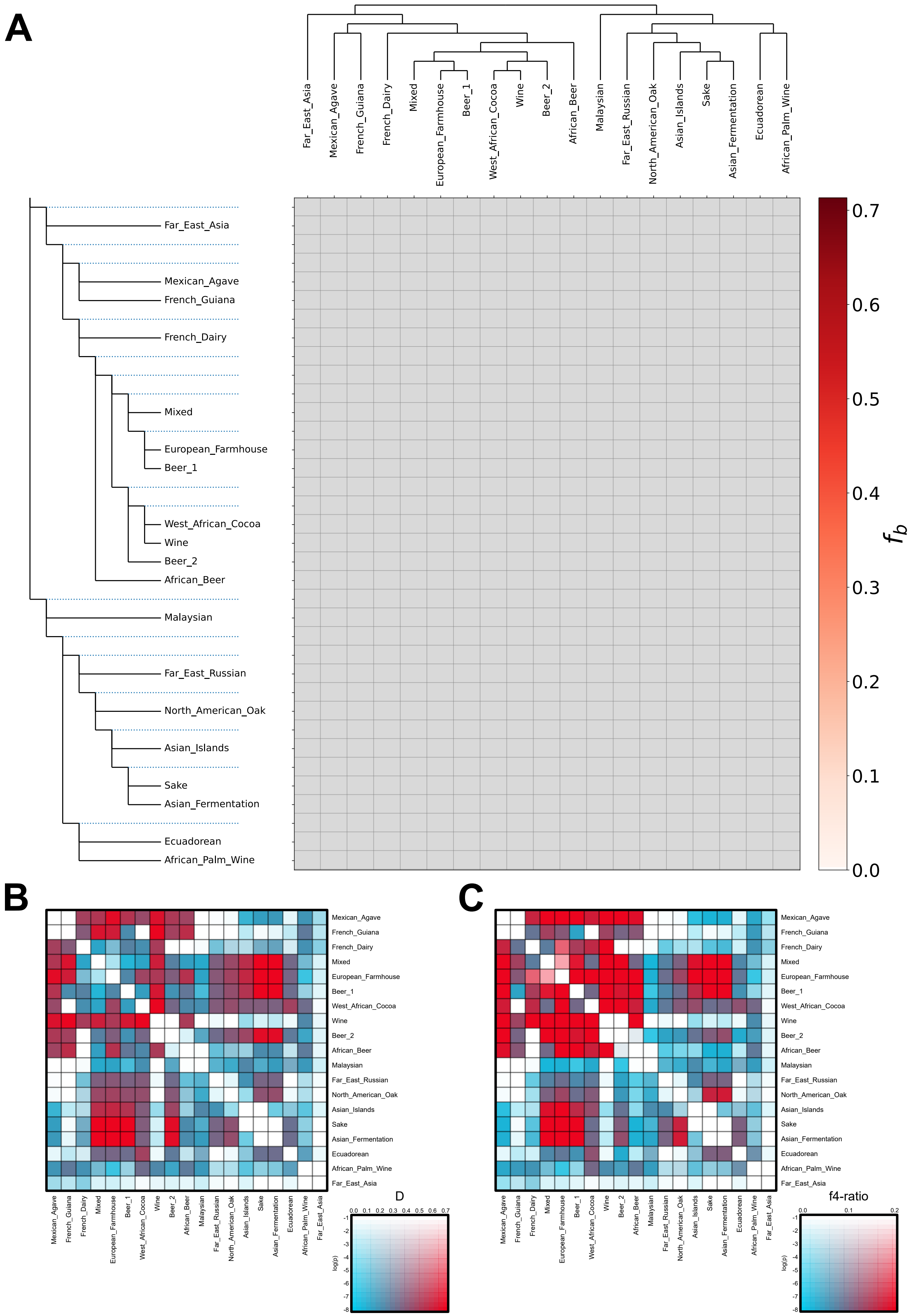

Supplementary Figure S8

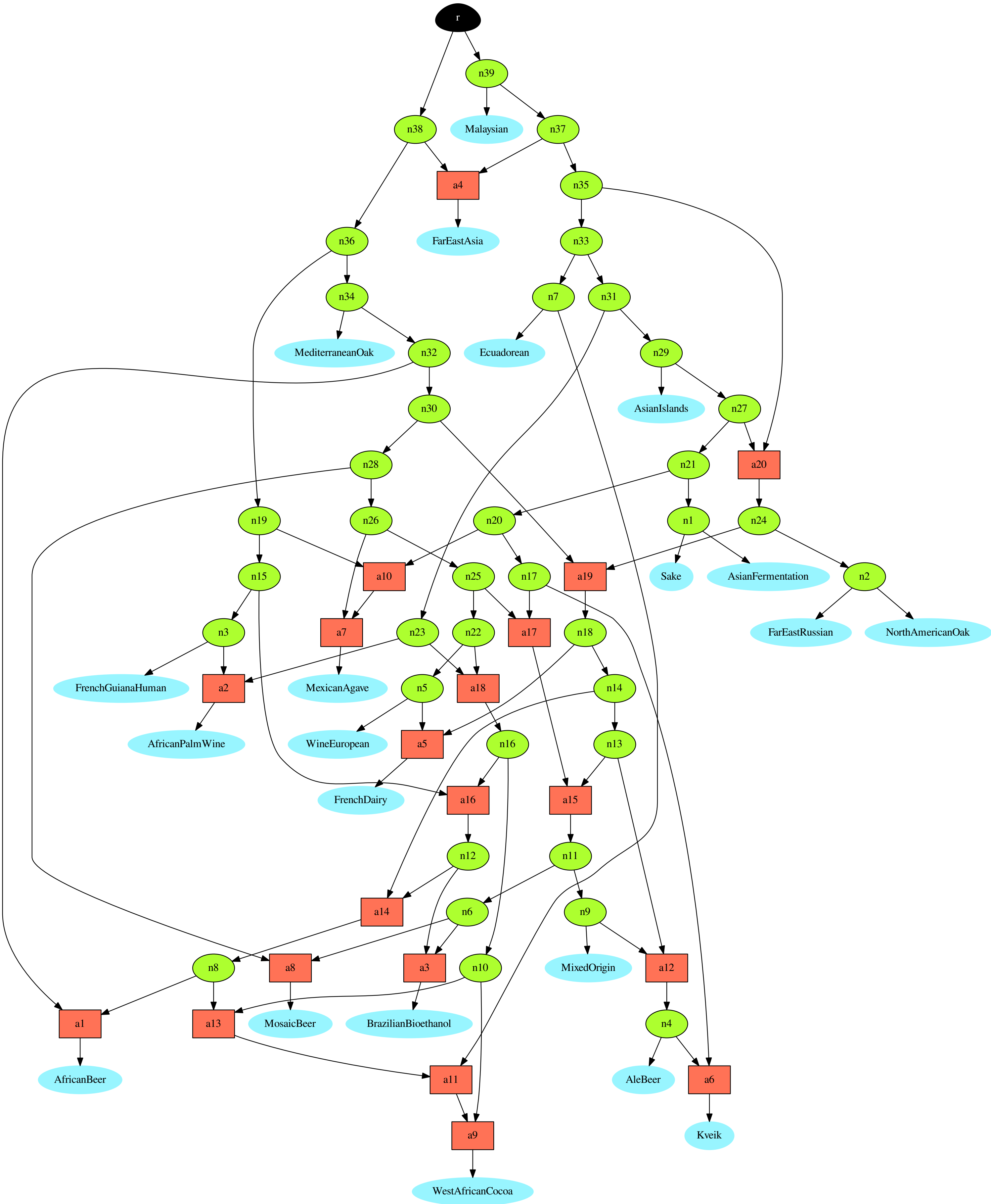

Supplementary Figure S9

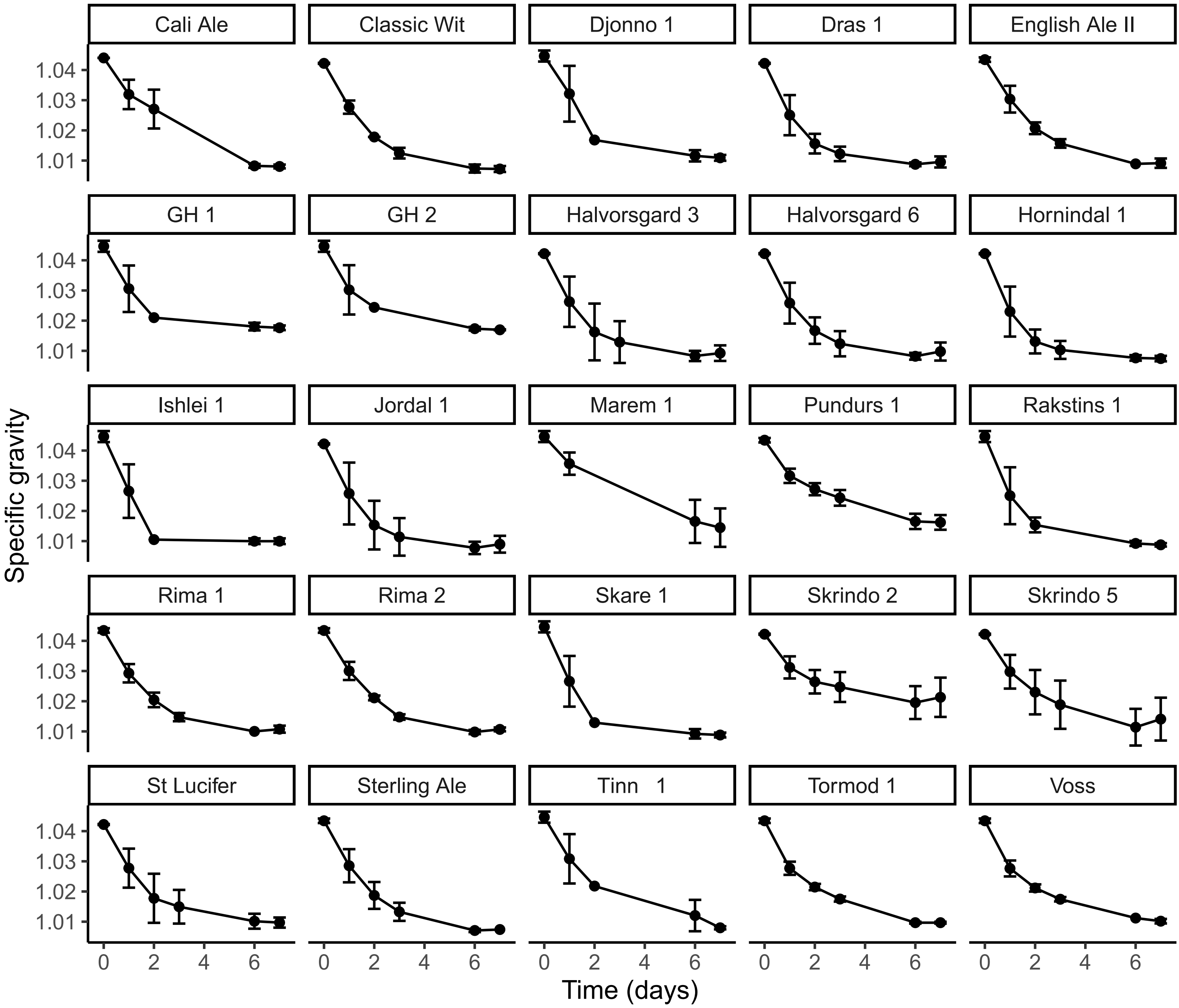

Supplementary Figure S10

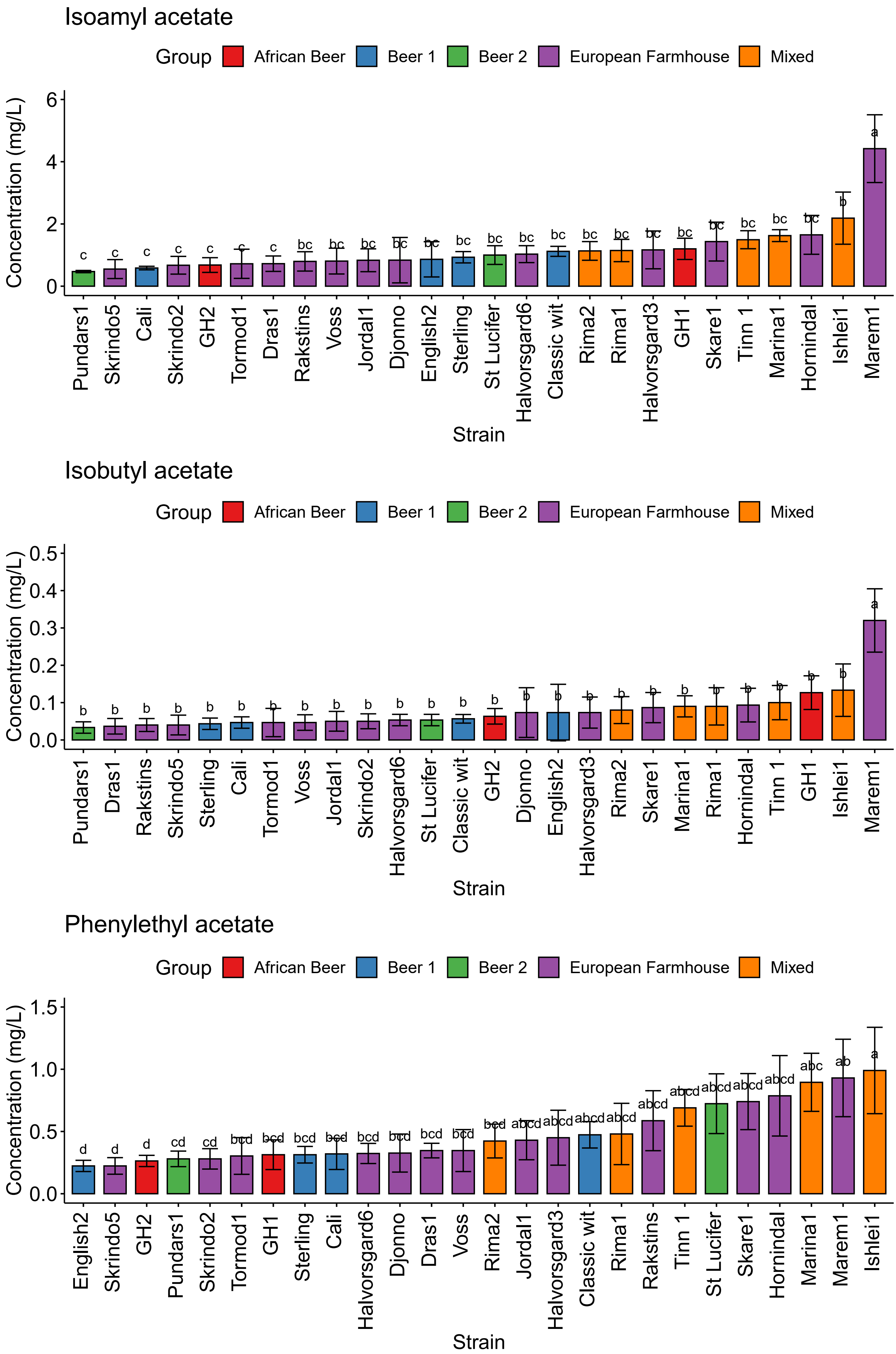

Supplementary Figure S11

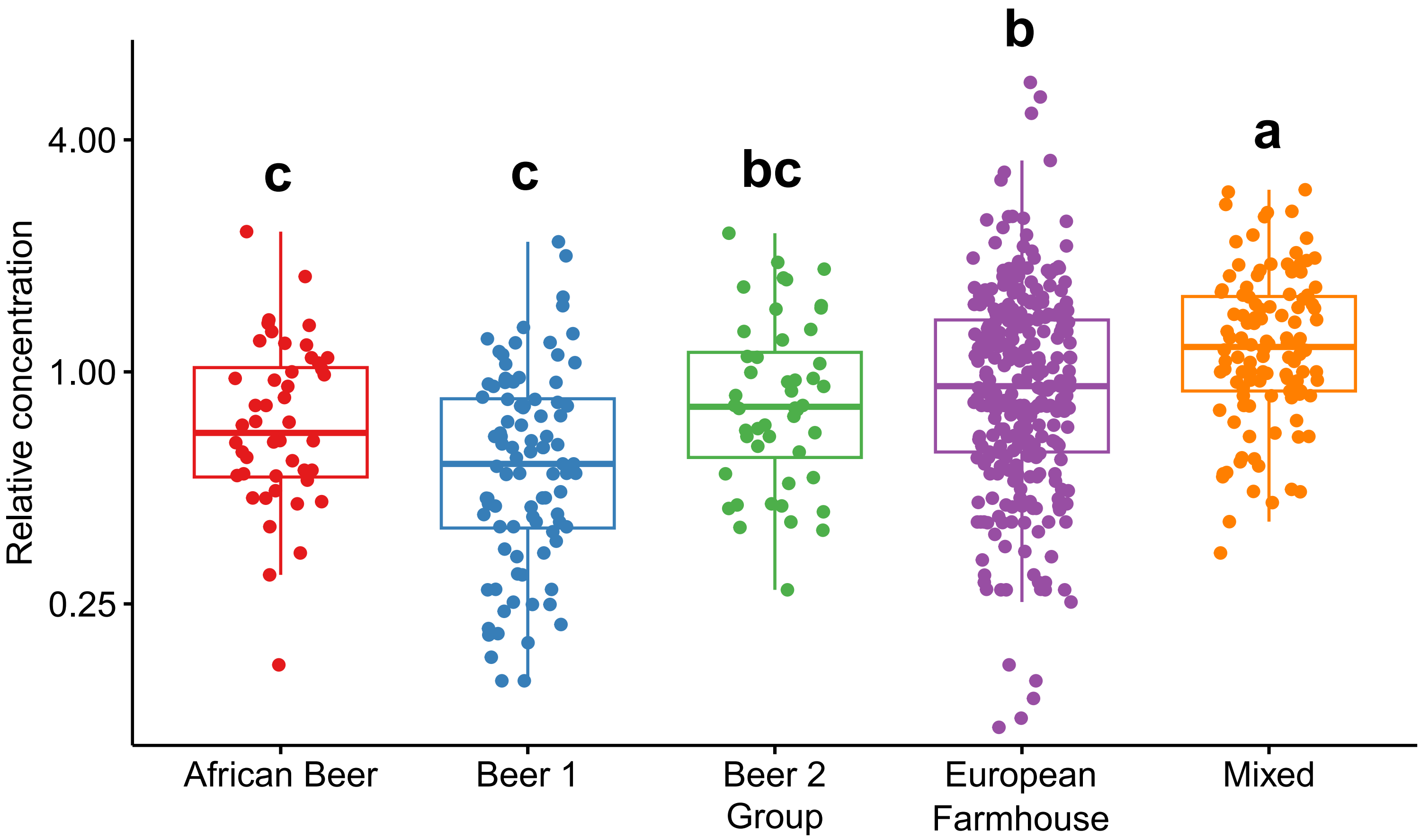

Supplementary Figure S12

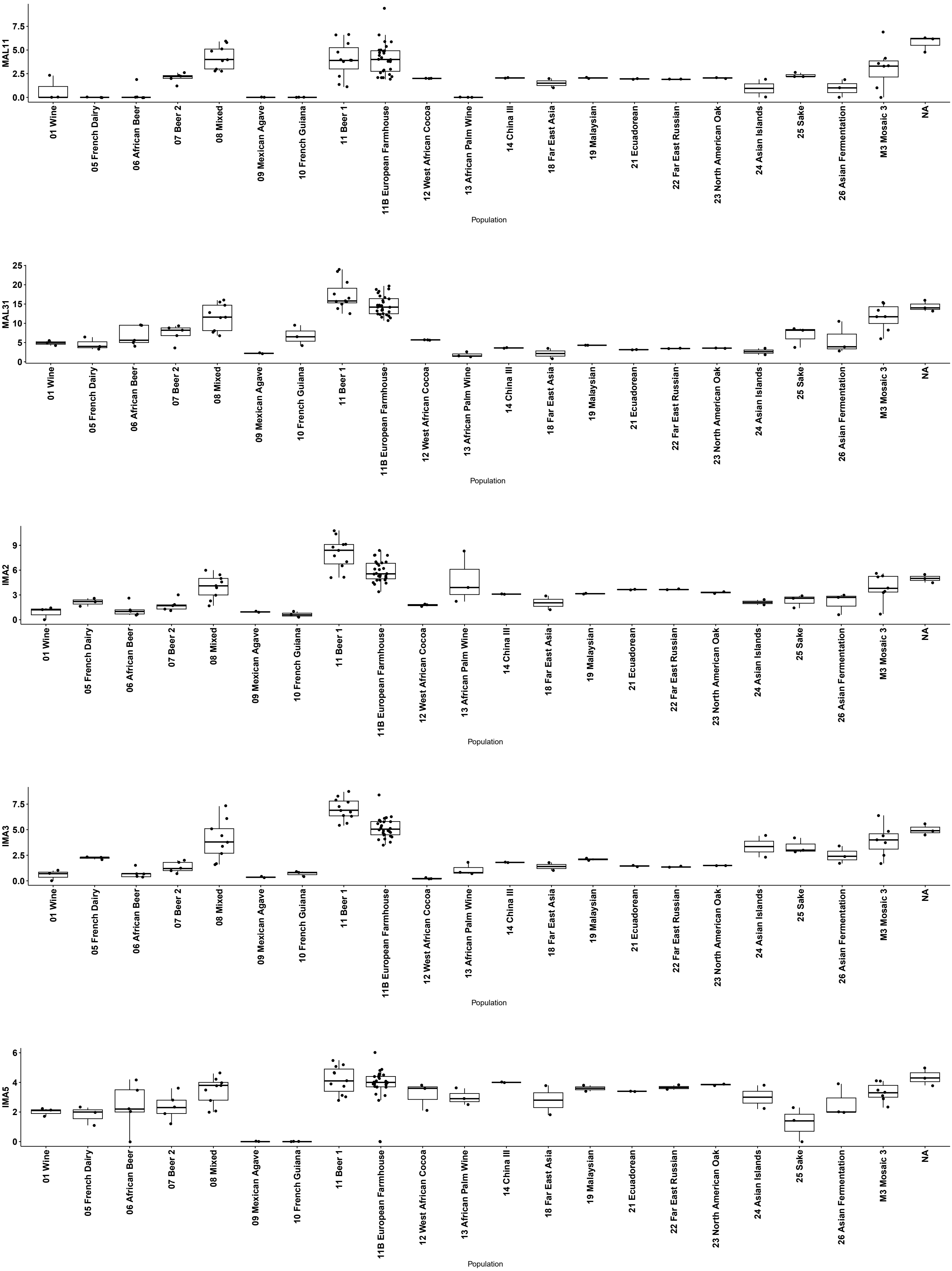

Supplementary Figure S13

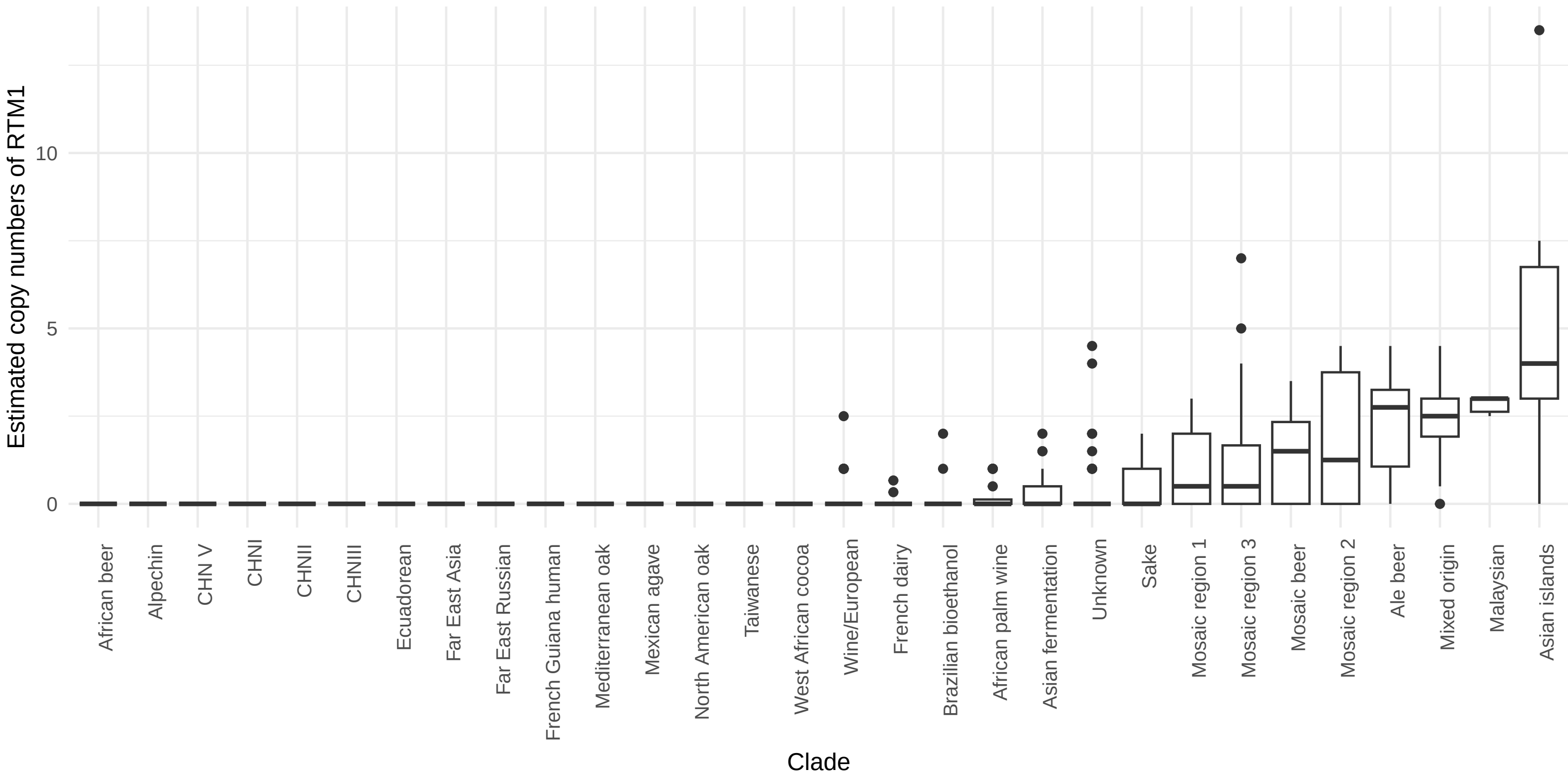

Supplementary Figure S14

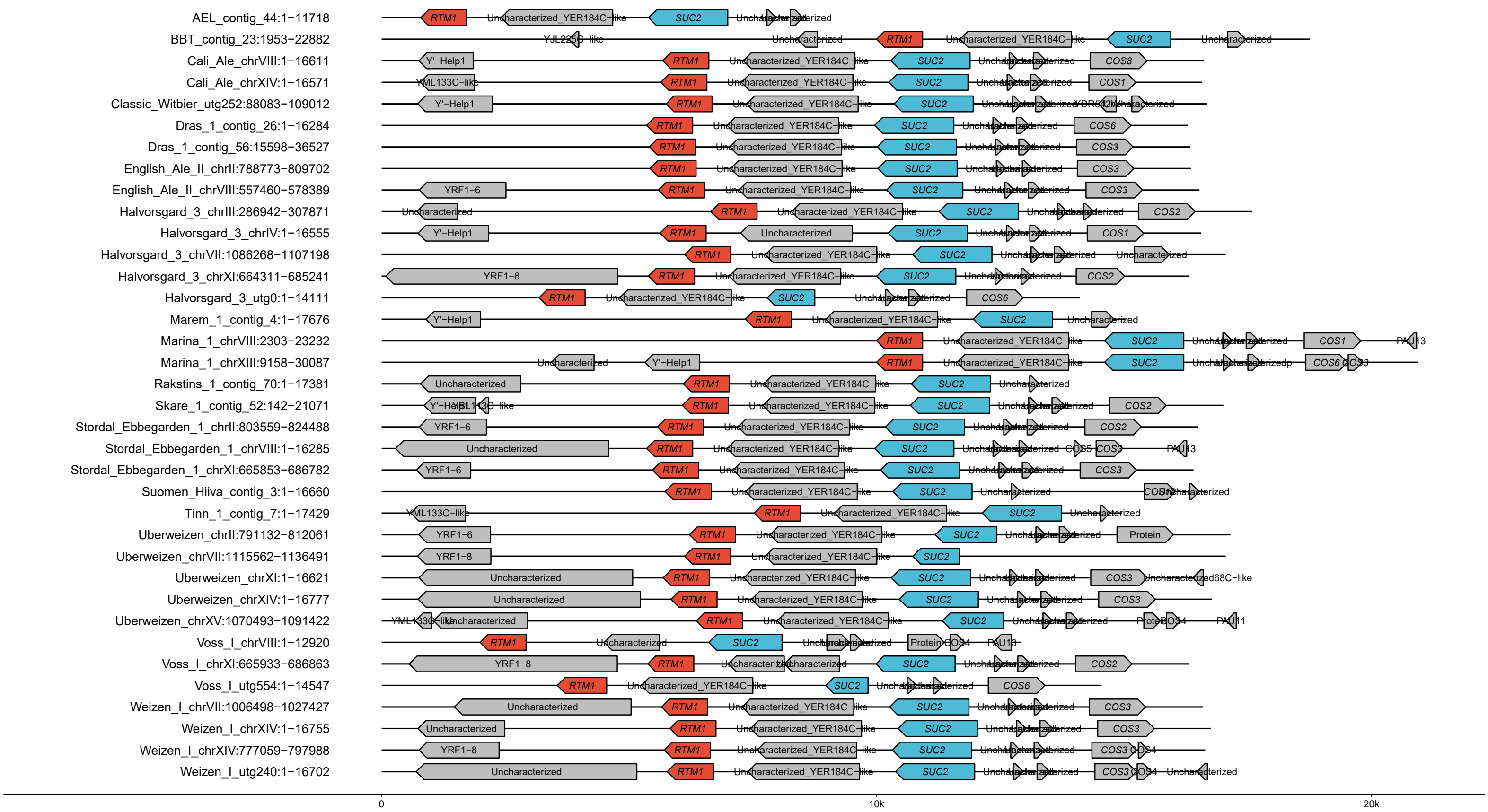

Supplement: Supplementary file 1 [file 253_2024_13267_MOESM1_ESM.pdf]
